# Identification of a Seven-Differentially Expressed Gene-Based Recurrence-Free Survival Model for Melanoma Patients

**DOI:** 10.1155/2022/3915112

**Published:** 2022-07-14

**Authors:** Yong Dong, Qian Miao, Da Li

**Affiliations:** ^1^Department of Medical Oncology, Sir Run Run Shaw Hospital, College of Medicine, Zhejiang University, Hangzhou, 310000 Zhejiang, China; ^2^Department of Medical Oncology, Quzhou People's Hospital, Quzhou, 324000 Zhejiang, China

## Abstract

Melanoma is a malignant tumor that originates in melanocytes of the skin or mucous membrane, which has a high mortality rate and worse prognosis. Therefore, perspective prognosis evaluation seems more important for patients' treatment. Gene expression profiles of melanoma were downloaded from The Cancer Genome Atlas (TCGA) and Gene Expression Omnibus (GEO) databases, respectively. 130 consistent differentially expressed genes (DEGs) were identified between melanoma and nevus tissues from two GEO cohorts. Prognostic genes were identified by univariate analysis, and 20 of them were regarded to be associated with the recurrence-free survival (RFS) of melanoma patients. Then, the LASSO Cox regression analysis chose seven of them to establish a seven-DEG-based RFS predicting signature. We demonstrated that this model was more powerful to predict RFS risk than other individual clinical features and was able to independently predict the RFS outcomes in different subsets of patients. We attempted to search for the underlying mechanisms by analyzing the coexpression genes of the seven candidates, and the pathway enrichment analyses indicated that immune response-related pathways might play a critical role in melanoma progression. Finally, we establish a robust seven-DEG-based RFS predicting signature, which will facilitate the personalized treatment of melanoma patients.

## 1. Introduction

**Figure 1:**
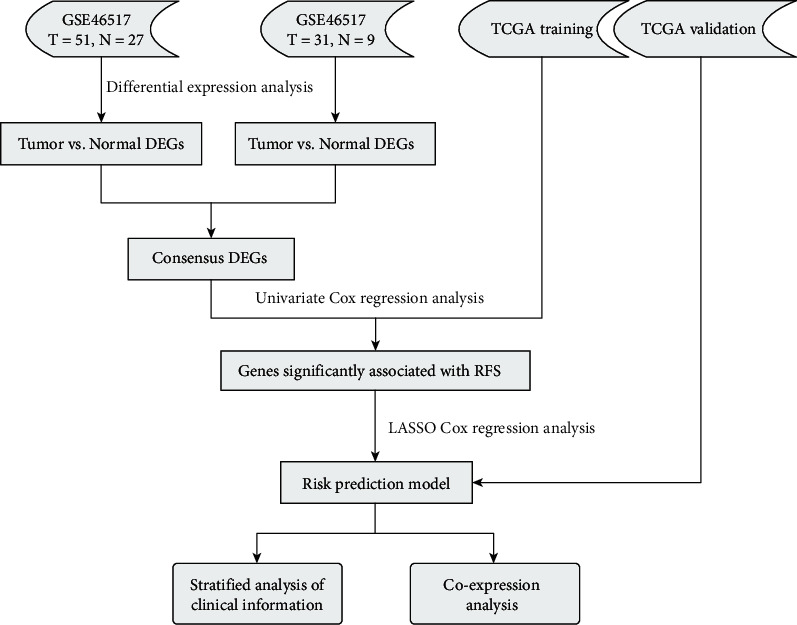
Flow diagram of this study.

Melanoma is caused by genetic mutations in melanocytes [1]. There will be 106,110 new cases of invasive melanoma in the United States in 2021, with 7,180 melanoma-related deaths. According to GLOBOCAN, the global number of melanoma cases in 2020 is 324,635, representing 1.7% of all cancers and 0.6% of 57,043 melanoma deaths or cancer-related mortality [2]. The classical prognostic factors which had been considered to be effective indicators for patients with melanoma are age and American Joint Committee on Cancer (AJCC) stage [3–5]. Besides, melanoma-specific indicators, such as Clark level [6], were also proved to provide prognostic information. Nevertheless, all the clinicopathological prognostic indicators are established basing on cancer instead of its underlying molecular subclassifications. That is why these indicators commonly are not useful in predicting clinical outcomes. Researchers tried to identify biomarkers at diverse biological levels to better predict the prognosis of melanoma patients; for instance, high expression of HIF stimulates the migration of melanoma cells and it also is relating to an unfavorable prognosis [7]. Based on OS and recurrence-free survival (RFS) rates, studies had shown that methylation of *TNFRSF10D* can predict the clinical outcome of melanoma patients [8]. Copy number variations of the interferon cluster which is related to T cell infiltration are also associated with OS of melanoma patients [9]. However, none of these biomarkers had been used clinically. Because of the genetic heterogeneity of melanoma, the instability of biomarkers' performance across cohorts was one of the most important reasons. Currently, according to the robustness across datasets, the optimized panels of gene expression based-models are emphasized [10–13]. For instance, MammaPrint and Oncotype DX have been used clinically due to their high performance [14–16]. In this study, we focused on developing a model for melanoma patients using gene expression profiling and evaluating the model's earnings management in the training and validation subsets (Figure 1).

## 2. Materials and Methods

### 2.1. Dataset Description

Two gene expression profiles of melanoma as well as common nevus based on RNA-seq (GSE98394) and microarray (GSE46517), respectively, were downloaded from the Gene Expression Omnibus (GEO) website (https://www.ncbi.nlm.nih.gov/geo). In the RNA-seq dataset (GSE98394), the researchers examined transcriptome changes from benign states to early-, intermediate-, and late-stage tumors using a set of 78 treatment-naive melanocytic tumors consisting of primary melanomas of the skin (*n* = 51) and benign melanocytic lesions (*n* = 27) [17]. For the other dataset (GSE46517), the authors analyzed 31 primary melanoma samples as well as 9 nevus samples basing on microarray. These two datasets were used for melanoma-related gene extraction. The expression matrix along with the matched clinical records of cohort from The Cancer Genome Atlas (TCGA) were downloaded from the UCSC Xena website (https://xenabrowser.net/datapages/) [18]. Only patients from TCGA database with sufficient RFS were retained and randomly divided into the training and validation subsets with a ratio of 7 : 3.

### 2.2. Differentially Expressed Gene (DEG) Analysis

DEGs of the RNA-seq cohort (GSE98394) were identified using “DESeq2 [19], and the DEGs of microarray-based cohort (GSE46517) were identified using the “limma” package [20]. DEGs were determined based on the thresholds that the absolute log2(fold − change) > 1 and the *P* value < 0.05. Then, we overlapped the upregulated DEGs as well as downregulated DEGs from the two datasets, respectively, to obtain more convincing results. The Kyoto Encyclopedia of Genes and Genomes (KEGG) and Gene Ontology (GO) analyses were executed to find out key pathways involved in the pathogenesis of melanoma using clusterProfiler [21].

### 2.3. Feature Selection and Model Development

R (version 3.6.2) was used for all calculations in this paper. The “survival” package (v3.1-8) was used for univariate/multivariate Cox regression analyses. TCGA gene expression value (Transcripts Per Kilobase of exon model per Million mapped reads (TPM)) was reversed into log_2_ (TPM + 1) for downstream analysis. The univariate Cox regression analysis was carried out to find out the RFS-related gene candidates (*P* < 0.05) using the consistent DEGs from the two GEO datasets. The LASSO Cox regression analysis was performed to select genes with the most prediction power. Then, the selected genes were used to construct the prognostic model by multivariate Cox regression analysis and the risk scores of each patient were calculated. Based on the prediction weight of each marker gene, we construct the prediction model and the risk level of each sample could be quantitized as follows: *risk* *score* = 0.127 × *expression* (*DFNA*5) − 0.300 × *expression* (*TNFRSF*1*B*) − 0.134 × *expression* (*TMEM*158) + 0.302 × *expression* (*MMP*11) − 0.020 × *expression* (*MAGEA*6) + 0.035 × *expression* (*APOBEC*3*G*) − 0.070 × *expression* (*ABCA*8). As a result, patients from TCGA database were separated into low- and high-risk subgroups (risk score < 0 or risk score > 0, respectively). Samples from the validation dataset were also separated into these two different risk level groups following the prognostic model. Kastle–Meyer test was performed to determine the significance of RFS risks between these two subgroups. In addition, the receiver operating characteristic (ROC) curve was applied to evaluate the robustness of the prognostic model using the R package “pROC” (v1.16.2) [22].

### 2.4. Identification of Coexpression Genes and Pathway Enrichment Analyses

To explore potential mechanisms of how these key genes play a role in the progression of melanoma, we tried to identify the highly coexpressed genes (Pearson correlation coefficient more than 0.5 or less than -0.5) of them based on TCGA gene expression profile. The Cytoscape software was executed to visualize their interactions, and KEGG and GO analyses were performed to verify the relevant significant pathways [21].

## 3. Results

### 3.1. Identification of DEGs

**Figure 2:**
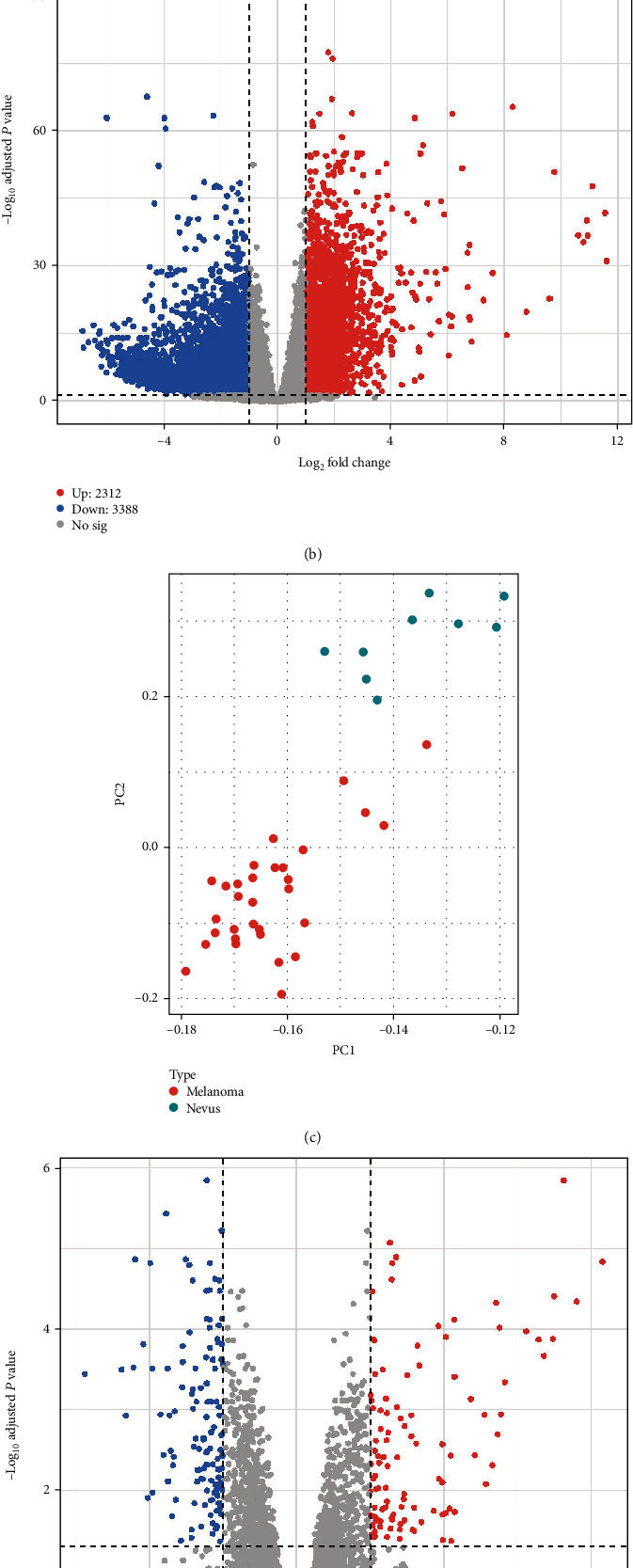
Identification of differentially expressed genes (DEGs). (a) Principal component analysis (PCA) distinguishing the melanoma and nevus tissues in the GSE98394 cohort; (b) the volcano plot displaying DEGs between melanoma and nevus tissues in the GSE98394 cohort; (c) PCA distinguishing the melanoma and nevus tissues in the GSE46517 cohort; (d) the volcano plot displaying DEGs between melanoma and nevus tissues in the GSE46517 cohort; (e) overlapping DEGs between two cohorts.

With the lack of normal tissues in TCGA dataset, we adopted two GEO datasets to find out key DEGs. The DEGs of the RNA-seq cohort (GSE98394) were identified using “DESeq2” [19], while the DEGs of the microarray cohort (GSE46517) were identified using the “limma” package [20]. Genes with |log_2_(*fold* − *change*)| > 1 and *P* value < 0.05 were considered statistically significant DEGs. For GSE98394 cohort, we found that 2,312 and 3,388 genes were upregulated and downregulated in melanoma tissues compared with normal controls, respectively (Figures 2(a) and 2(b) and Supplementary table 1). For the GSE46517 cohort, 107 genes were upregulated and 122 genes were downregulated in melanoma tissues compared with normal controls (Figures 2(c) and 2(d) and Supplementary table 2). In order to obtain more convincing DEG assembly, we overlapped the findings from two datasets. After that, there were 79 genes upregulated and 51 genes downregulated by comparing the melanoma tissues with normal controls (Figure 2(e) and Supplementary table 3). Interestingly, these DEGs were significantly enriched in activity of different enzymes and complement-related pathways (Figure S1).

### 3.2. Identification of Prognostic Genes and Establishing a Seven-Gene-Based RFS Predicting Signature

**Figure 3:**
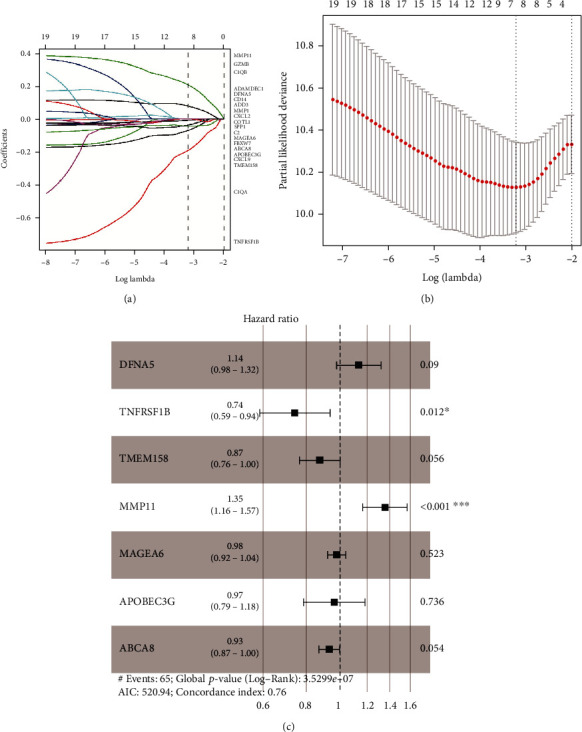
Seven-gene-based signature construction for recurrence-free survival (RFS) prediction. (a) LASSO coefficient against tried values of log lambdas; (b) 10-fold cross-validation for tuning parameter (lambda) selection via minimum criteria in the model; (c) forest plot showed results of multivariate cox analysis.

After extracting the 130 genes' expression profile from GEO datasets, we tried to establish a RFS-related prognostic model using separated training and testing dataset from TCGA (Table 1). RFS-related gene candidates were identified by univariate Cox regression analysis. In total, 20 genes were detected significantly associated with the RFS (Figure S2). Then, we executed the LASSO Cox regression analysis to extract key genes with the most RFS prediction power. These genes were then subjected to LASSO Cox regression analysis, and regression coefficients were calculated. The coefficient of each gene is plotted in Figure 3(a). To confirm the accuracy of this risk model, 10-fold cross-validation was performed to obtain the confidence interval under each lambda (Figure 3(b)). After this process, seven key genes including *DFNA5*, *TNFRSF1B*, *TMEM158*, *MMP11*, *MAGEA6*, *APOBEC3G*, and *ABCA8* were extracted. As a result, a seven-gene-based RFS predicting model was extracted which refers to the key gene expression using multivariate Cox regression: *risk* *score* = 0.127 × *expression* (*DFNA*5) − 0.300 × *expression* (*TNFRSF*1*B*) − 0.134 × *expression* (*TMEM*158) + 0.302 × *expression* (*MMP*11) − 0.020 × *expression* (*MAGEA*6) + 0.035 × *expression* (*APOBEC*3*G*) − 0.070 × *expression* (*ABCA*8) (Figure 3(c)). The patients with risk score < 0 and >0 were assigned into low- and high-risk subgroups, respectively.

### 3.3. Significance and Stability Assessment of the Seven-Gene-Based RFS Predicting Signature

**Figure 4:**
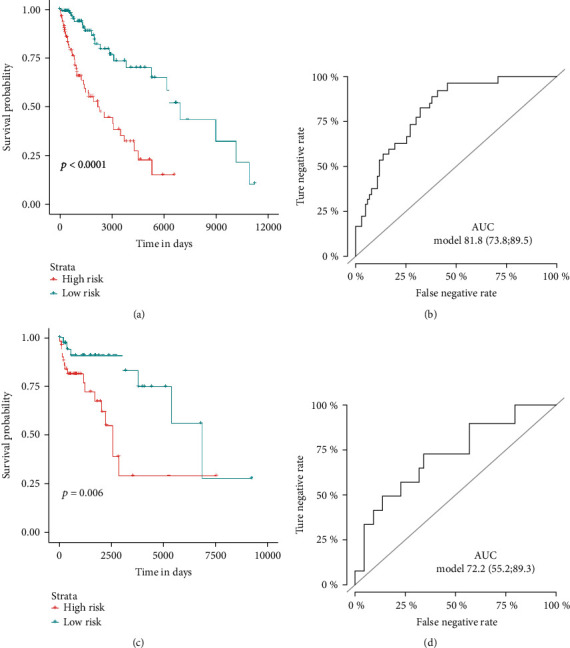
Assessment of the predictive value of the signature. (a) The K-M curve showed that the low- and high-risk sample groups from the training cohort separated by the seven-gene-based signature had significant PFS difference. (b) ROC curve showed that the signature obtained good predictive effect in the training cohort. (c) The K-M curve confirmed that the low- and high-risk sample groups from the training cohort separated by the seven-gene-based signature had significant PFS difference. (d) ROC curve showed that the signature obtained good predictive performance in the validation cohort. K-M: Kaplan-Meier; ROC: receiver operating characteristic.

Subsequently, we employed K-M analysis to validate the significance of the seven-gene-based RFS predicting signature, and results indicated that for these patients in the low-risk group, most have better survival outcomes than those in the high-risk subset in both the training (*P* < 0.001, Figure 4(a)) and validation cohorts (*P* = 0.006, Figure 4(c)). In addition, the sensitivity and stability of this model were proved by the ROC curve. We found that our model obtained a satisfying predictive value in the training cohort (*AUC* = 81.7, 95% CI: 73.8-89.5, Figure 4(b)) and a moderate predictive value in the validation cohort (AUC = 72.2, 95% CI: 55.2-89.3, Figure 4(d)).

### 3.4. Prediction Efficiency Comparison and Patient Subgroup Analyses

**Figure 5:**
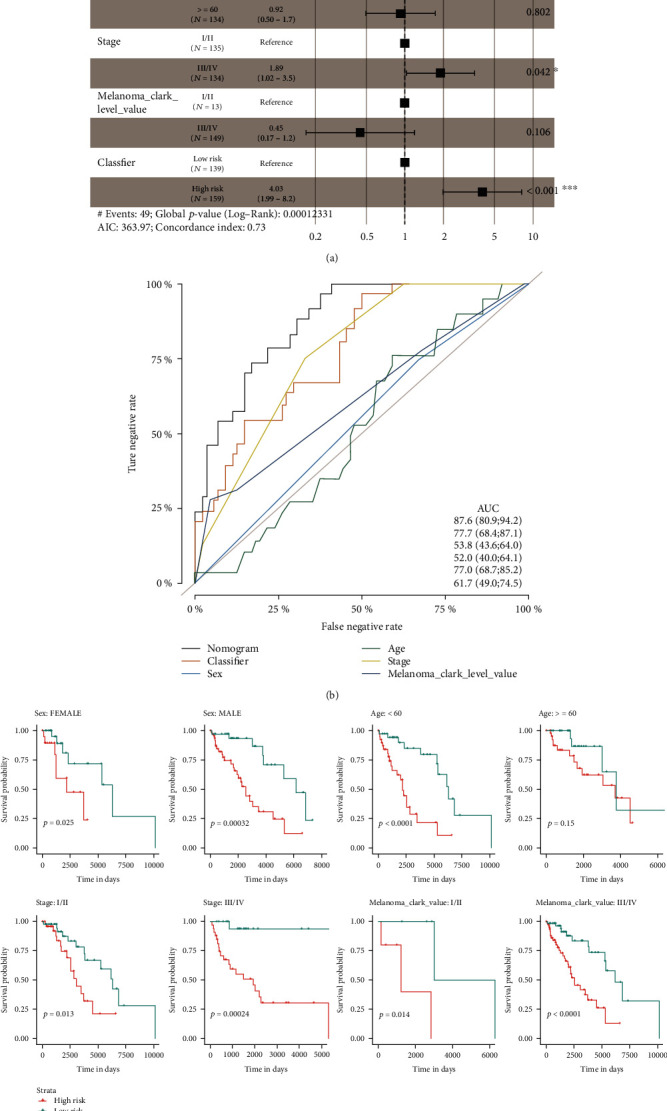
Multivariate analysis. (a) Multivariate Cox regression analysis showed the signature as an independent prognostic indicator. (b) The ROC curve showed that the seven-gene-based signature had better predictive performance than other clinical features. (c) Subgroup analyses confirmed the prediction value of the signature in different clinical subsets of melanoma patients.

We combined other clinicopathological features including patient age, gender, tumor stage, and melanoma Clark level value with people's prediction gene signature and carried out multivariant Cox regression analysis. The seven-gene-based signature was still statistically significant (*P* < 0.001) and provided a much higher predictive value (HR = 4.03, 95% CI: 1.99-8.2) than other clinical features, indicating the prediction signature was an independent prognostic factor for RFS prediction (Figure 5(a)). To further verify the prognostic prediction efficiency of our model, we performed the ROC analysis and compared the area under curve (AUC) with other individual clinicopathological features. As a result, our signature showed better predictive value (AUC = 77.7, 95% CI: 68.4-87.1) than melanoma Clark level value (AUC = 61.7, 95% CI: 49.0-74.5), stage (AUC = 77.0, 95% CI: 68.7-85.2), age (AUC = 52.0, 95% CI: 40.0-64.1), and sex (AUC = 53.8, 95% CI: 43.6-64.0) (Figure 5(b)). Notably, we found that the nomogram showed the best predictive value by combing the classifier with other features, with the AUC value of 87.6 (95% CI: 80.9-94.2).

We also performed subgroup analyses referring to different clinicopathological features, such as stage (I/II and III/IV) and sex (female and male). The results indicated that our signature was able to predict RFS outcomes for different sex (female and male), stage (I/II and III/IV), and melanoma Clark level value (I/II and III/IV) subsets, while it was only able to predict the RFS outcomes for patients' age less than 60 rather than ≥60 (Figure 5(c)). Overall, all the results support the significance and stability of the seven-gene-based RFS predicting signature.

### 3.5. Identification of Coexpression Genes and Pathway Enrichment

**Figure 6:**
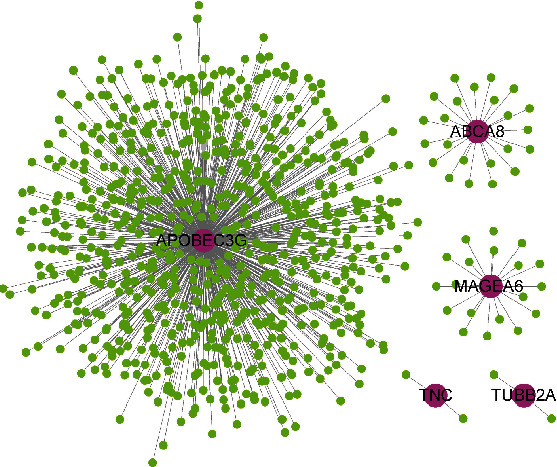
Interaction network between the seven genes and their corresponding coexpressed genes. Genes that have a Pearson correlation coefficient more than 0.5 or less than -0.5 were defined as coexpressed genes and are shown in this figure (green nodes).

**Figure 7:**
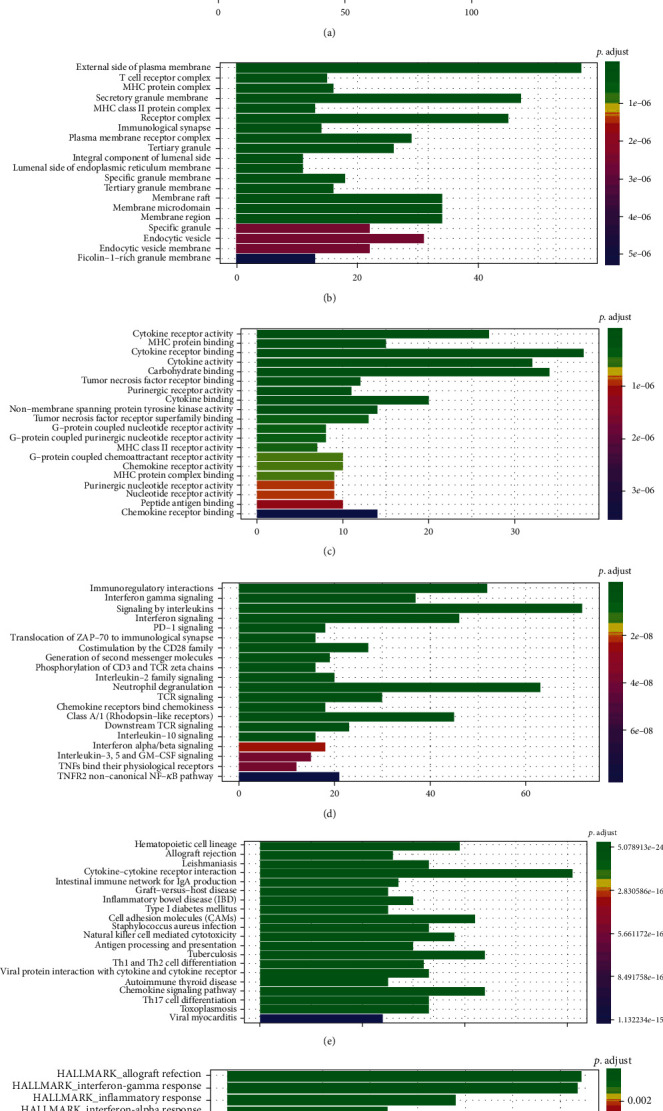
Pathway enrichment analyses. (a–f) GO-BP, GO-CC, GO-MF, KEGG, Reactome, and Hallmark pathway enrichment analyses for the coexpressed genes of the seven genes enrolled in the signature. GO: Gene Ontology; BP: biological process; CC: cellular component; MF: molecular function; KEGG: Kyoto Encyclopedia of Genes and Genomes.

To explore potential mechanisms on how these key genes influence the progression of melanoma, we performed the coexpression analysis based on TCGA gene expression profile. These genes, whose Pearson correlation coefficient was more than 0.5 or less than -0.5, were enrolled. Then, 783 highly coexpressed genes were merged from five of the seven key genes (Figure 6). Besides, the GO (Figures 7(a)–7(c)), KEGG (Figure 7(d)), Reactome (Figure 7(e)), and Hallmark (Figure 7(f)) pathway overrepresent enrichment analyses were performed to investigate the relevant significant pathways. We found that these highly coexpressed genes were playing a critical role in immune response processes, such as T cell activation, regulation of lymphocyte activation, cytokine receptor activity, and immunoregulatory interactions.

## 4. Discussion

Although AJCC staging system provides prognostic information for melanoma patients to some extent [23], remarkable prognostic heterogeneity exists, and several clinical prognosis predicting tools have been established to improve predictive value [24], with limited effects obtained. In the present study, we firstly identified the DEGs based on two cohorts. Many of the critical genes lead to tumorigenesis or progression when there is differential expression due to mutation, amplification, or loss. Thus, we tried to establish a DEG-based RFS predicting signature. With the help of LASSO Cox regression analysis, a seven-gene-based RFS predicting signature was set up by which the melanoma patients were assigned into low and high risk, and K-M and ROC curve analyses provide the significance and stability of our signature. Further, multivariate analysis confirmed the independence of our signature from clinicopathological features, and it worked even better the clinical stage. We explored the potential mechanisms of how these DEG candidates influence the progression of melanoma patients. We calculated the Pearson correlation coefficient between the seven DEGs and the whole mRNA profile; then, the pathway enrichment analyses were to classify the function of these coexpressed genes. Interestingly, we found that these genes were significantly enriched in immune response-related pathways, such as T cell activation, regulation of lymphocyte activation, cytokine receptor activity, and immunoregulatory interactions, indicating immune responses potentially promote the progression of melanoma.

Melanoma is described as one of the most immunogenic tumors, and many studies have been devoted to exploring the relationship between tumorigenesis and immune system [25]. And the results suggested that immunomodulatory mechanisms have been revealed leading to immune resistance and immunosuppression by mediating the disorder of melanoma recognition and attack by immune cells, favoring tumorigenesis and progression. Studies also confirmed the correlation between the proliferation of melanoma cells and the activity defective immune system, while others described that the variability of the antigenic repertoire is a pivotal factor for the immunosurveillance and progression of melanoma [26, 27]. These findings provide therapeutic advantages to conquer the immune evasion.

In our study, we found two independent prognostic indicators, the seven-DEG-based signature, and the AJCC staging system. Recently, an online predicting website was established based on the big AJCC melanoma staging dataset for melanoma patients with localized disease [28], by which each patient could obtain their 1-, 2-, 5-, and 10-year survival with 95% confidential interval (95% CI). Increasing evidence suggests that molecular profiling will add additional information supporting to the staging and prognosis prediction of melanoma patients. Hence, attributing to an improved cognition of disease at molecular level, improvements in targeting therapies for metastatic melanoma patients are obtained [29–33]. Nevertheless, biomarkers for the diagnosis, prognosis prediction, and guidance of treatment for melanoma patients are still lacking. In our study, we analyzed the DEGs from two cohorts and overlapped the findings of the two cohorts to obtain a more accurate and convincing DEG cluster. The LASSO Cox regression analysis was used to choose the stable and effective candidates. Thus, the final model we established based on seven DEGs is able to discriminate the melanoma patients with high and low risk. The K-M and ROC curve analyses confirmed its application value. Besides, the multivariate analysis supports its independent role in predicting RFS of melanoma patients.

To sum up, the prognostic model we developed based on the seven DEGs is robust in RFS outcome prediction of melanoma, and it also serves as an independent prognostic indicator for melanoma prognosis. Our signature offers a complement to clinicians with RFS information and will help the organization of future individualized therapy. Further multicenter-based large-scale studies are necessary to verify these findings, promoting its clinical application.

## Figures and Tables

**Table 1:** Clinical parameters of The Cancer Genome Atlas dataset.

		Training set	Testing set	*P*	SMD	Missing (%)
Total number		209	89			
Sex (%)	Male	125 (59.8%)	63 (70.8%)	0.096	0.232	0
	Female	84 (40.2%)	26 (29.2%)			
Age (%)	<60	117 (56.0%)	47 (52.8%)	0.707	0.064	0
	≥60	92 (44.0%)	42 (47.2%)			
Stage (%)	I/II	89 (46.4%)	46 (59.0%)	0.088	0.250	9.7
	III/IV	102 (53.4%)	32 (41.0%)			
Melanoma Clark level value (%)	I/II	9 (8.0%)	4 (8.0%)	1.000	0.001	45.6
	III/IV	103 (92.0%)	46 (92.0%)			

TCGA: The Cancer Genome Atlas; SMD: standard mean difference.

## Data Availability

The data used to support the findings of this study are included within the article.

## Supplementary Materials

Supplementary Materials Figure S1: functional enrichment result of overlapped DEGs between GSE98394 and GSE46517. Figure S2: the K-M curves show genes that significantly correlated with PFS. Grouping of samples is based on median gene expression.
