# Supplementary material for: Identification of a Seven-Differentially Expressed Gene-Based Recurrence-Free Survival Model for Melanoma Patients
Source: Dis Markers. 2022 Jul 14;2022:3915112. doi: 10.1155/2022/3915112 (PMC9303152; doi:10.1155/2022/3915112)
Supplement: Supplementary Materials — Figure S1: functional enrichment result of overlapped DEGs between GSE98394 and GSE46517. Figure S2: the K-M curves show genes that significantly correlated with PFS. Grouping of samples is based on median gene expression. [file 3915112.f1.zip › Supplementary table 1 (1).docx]

**ID**

**Supplementary table 1. The differentially expressed genes between melanoma and nevus tissues in GSE98394 cohort.**

**Base** **Mean**

**log2** **(FoldChange)** **lfcSE**

**stat**

***P-*** **value**

***P*** **-adjust**


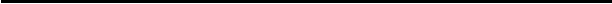


| A1BG 36 .760231 | -0 .343693205 | 0 . 18693 | -1 .8386 | 0 .06597 | 0 .09016 |
| --- | --- | --- | --- | --- | --- |
| A1CF 35 .32713206 | -3 .081868001 | 0 .35928 | -8 .5779 | 9 .66E-18 | 1 . 14E-16 |
| A2M 36759 .42273 | 2 .409016032 | 0 . 19016 | 12 .6682 | 8 .87E-37 | 7 .70E-35 |
| A2ML1 7206 .075283 | -0 .026631807 | 0 .26837 | -0 .0992 | 0 .92095 | 0 .93425 |
| A3GALT2 8 .093812334 | -0 .064910454 | 0 .29179 | -0 .2225 | 0 .82396 | 0 .85125 |
| A4GALT 504 .0795807 | 1 .018921209 | 0 . 18102 | 5 .62881 | 1 .81E-08 | 7 .00E-08 |
| A4GNT 14 .09842526 | -1 . 125424656 | 0 .31754 | -3 .5442 | 0 .00039 | 0 .00079 |
| AAAS 1660 .931957 | -0 . 17905023 | 0 .07664 | -2 .3362 | 0 .01948 | 0 .02947 |
| AACS 3195 . 174509 | 0 .097402141 | 0 . 18115 | 0 .53768 | 0 .5908 | 0 .64171 |
| AADAC 337 .3690647 | -2 .362410443 | 0 .35316 | -6 .6893 | 2 .24E-1 1 | 1 .26E-10 |
| AADACL2 3016 .096449 | -2 . 108551685 | 0 .28241 | -7 .4664 | 8 .24E-14 | 6 . 1 1E-13 |
| AADACL3 4071 .956282 | 1 .814076717 | 0 .79983 | 2 .26807 | 0 .02332 | 0 .03481 |
| AADACL4 8 .777710824 | -1 .329888002 | 0 .60048 | -2 .2147 | 0 .02678 | 0 .03955 |
| AADAT 337 .3361897 | -0 .910571621 | 0 . 13641 | -6 .6753 | 2 .47E-1 1 | 1 .38E-10 |
| AAED1 2468 .915729 | 0 .783220606 | 0 . 12426 | 6 .30322 | 2 .92E-10 | 1 .42E-09 |
| AAGAB 3093 .376462 | 0 .505498885 | 0 .08206 | 6 . 16038 | 7 .26E-10 | 3 .36E-09 |
| AAK1 1574 .889524 | 0 .349512022 | 0 . 1 1745 | 2 .97588 | 0 .00292 | 0 .00511 |
| AAMDC 459 .2967512 | -0 .860828628 | 0 . 14329 | -6 .0076 | 1 .88E-09 | 8 .24E-09 |
| AAMP 4276 .209646 | 0 .001163095 | 0 .07851 | 0 .01481 | 0 .98818 | 0 .98993 |
| AANAT 19 .05501293 | 0 . 135951848 | 0 .24542 | 0 .55395 | 0 .57961 | 0 .63099 |
| AAR2 2665 .678173 | -0 .02206478 | 0 .08197 | -0 .2692 | 0 .7878 | 0 .81905 |
| AARD 156 .4826541 | 1 .474785554 | 0 .34185 | 4 .31412 | 1 .60E-05 | 4 .00E-05 |
| AARS 4835 .203244 | 0 .743258177 | 0 . 12023 | 6 . 18197 | 6 .33E-10 | 2 .95E-09 |
| AARS2 1767 .411 193 | 0 .351000479 | 0 . 10847 | 3 .23599 | 0 .00121 | 0 .00226 |
| AARSD1 81 .54735037 | -0 .442120609 | 0 . 13812 | -3 .2011 | 0 .00137 | 0 .00253 |
| AASDH 2031 .004447 | -0 .562054944 | 0 .09445 | -5 .9508 | 2 .67E-09 | 1 . 15E-08 |
| AASDHPPT 4385 .873485 | -0 .398070216 | 0 . 10648 | -3 .7384 | 0 .00019 | 0 .00039 |
| AASS 3109 .380559 | -0 .834860031 | 0 . 13989 | -5 .9681 | 2 .40E-09 | 1 .04E-08 |
| AATK 403 .2962308 | 1 .437838148 | 0 . 19731 | 7 .28738 | 3 . 16E-13 | 2 .21E-12 |
| ABAT 536 .5197402 | 0 .281063586 | 0 . 13947 | 2 .01521 | 0 .04388 | 0 .06212 |
| ABCA1 19622 .8022 | 0 .34171262 | 0 . 1 1 1 16 | 3 .07406 | 0 .00211 | 0 .00378 |
| ABCA10 4801 . 17997 | -2 .285294165 | 0 .28585 | -7 .9946 | 1 .30E-15 | 1 . 19E-14 |
| ABCA12 10129 .95222 | -0 .939254801 | 0 .24579 | -3 .8213 | 0 .00013 | 0 .00029 |
| ABCA13 672 .680647 | 0 .208026782 | 0 .3123 | 0 .66611 | 0 .50534 | 0 .56003 |
| ABCA2 2228 .970955 | 0 . 19841604 | 0 . 13352 | 1 .48605 | 0 . 13727 | 0 . 17558 |
| ABCA3 911 .6122979 | 0 .722246911 | 0 . 16613 | 4 .34756 | 1 .38E-05 | 3 .48E-05 |
| ABCA4 199 .6119661 | -0 .033800759 | 0 .29746 | -0 . 1 136 | 0 .90953 | 0 .92496 |
| ABCA5 17608 .901 | -2 .023262289 | 0 . 17397 | -1 1 .63 | 2 .89E-31 | 1 .44E-29 |
| ABCA6 8642 .847856 | -1 .500251855 | 0 .25507 | -5 .8818 | 4 .06E-09 | 1 .70E-08 |
| ABCA7 983 . 1660912 | 1 .49599074 | 0 . 13875 | 10 .7818 | 4 .20E-27 | 1 .39E-25 |
| ABCA8 6992 .420839 | -1 .619977949 | 0 .26293 | -6 . 1611 | 7 .22E-10 | 3 .35E-09 |
| ABCA9 3225 .644196 | -1 .366651006 | 0 .25833 | -5 .2904 | 1 .22E-07 | 4 . 19E-07 |
| ABCB1 526 .7696049 | 0 .4951999 | 0 .22761 | 2 . 17565 | 0 .02958 | 0 .04327 |
| ABCB10 2175 .501503 | 0 .454735628 | 0 .09483 | 4 .79527 | 1 .62E-06 | 4 .68E-06 |
| ABCB11 78 .02292386 | -0 .882936973 | 0 .35522 | -2 .4856 | 0 .01293 | 0 .02016 |
| ABCB4 1 103 .08713 | 0 .760985346 | 0 .24167 | 3 . 14886 | 0 .00164 | 0 .00298 |
| ABCB5 1636 .203129 | 3 .039440976 | 0 .49024 | 6 . 1999 | 5 .65E-10 | 2 .65E-09 |
| ABCB6 96 . 1437696 | 0 .013432015 | 0 . 13372 | 0 . 10045 | 0 .91999 | 0 .93352 |
| ABCB7 1806 .957297 | -0 .845859067 | 0 .05368 | -15 .758 | 6 .05E-56 | 3 .32E-53 |
| ABCB8 1414 .857265 | 0 .861772011 | 0 .0955 | 9 .02336 | 1 .82E-19 | 2 .64E-18 |
| ABCB9 316 .5004811 | 0 .666053057 | 0 . 12322 | 5 .40532 | 6 .47E-08 | 2 .31E-07 |
| ABCC1 2976 .880613 | 0 .94495928 | 0 . 10421 | 9 .06812 | 1 .21E-19 | 1 .79E-18 |
| ABCC10 1605 .234053 | 0 .574624311 | 0 .09858 | 5 .8291 | 5 .57E-09 | 2 .30E-08 |

| ABCC11 ABCC12 ABCC2 ABCC3 ABCC4 ABCC5 ABCC6 ABCC8 ABCC9 ABCD1 ABCD2 ABCD3 ABCD4 ABCE1 ABCF1 ABCF2 ABCF3 ABCG1 ABCG2 ABCG4 ABCG5 ABCG8 ABHD1 ABHD10 ABHD11 ABHD12 ABHD12B ABHD13 ABHD14A | 236 .4383232 19 .30790576 1261 .040761 1029 .02862 2384 .856599 3798 .815261 79 .00187064 31 .66487244 3329 .361936 1 194 .376791 345 .2578612 7935 .094913 1549 . 194517 8225 .763152 2814 . 154456 4244 .334407 1961 .928648 2271 .673642 585 .4407417 55 .53344604 49 .58214914 6 .856063699 77 .40553333 3208 .806309 747 .8803405 2602 .016458 1647 .897815 3206 .519268 209 .3289278 |
| --- | --- |

ABHD14A-AC7 .502000012

| ABHD14B ABHD15 ABHD16A ABHD16B ABHD17A ABHD17B ABHD17C ABHD18 ABHD2 ABHD3 ABHD4 ABHD5 ABHD6 ABHD8 ABI1 ABI2 ABI3 ABI3BP ABL1 ABL2 ABLIM1 ABLIM2 ABLIM3 ABR ABRA ABRACL | 1720 .714449 845 .4448767 241 .0920974 151 .9084761 4052 .791056 2958 .425838 1039 .727267 1513 .501381 15930 .48162 1097 .51348 755 .0278667 4644 .445824 2267 .826865 328 .3205517 8585 .473048 511 1 .676982 311 .8825422 4372 .280106 3407 .867451 14076 .69177 12445 .65611 264 .8910628 1496 .889546 8236 .258539 59 .0362519 1783 .723472 |
| --- | --- |

-1 .797429547

-2 .76756674

2 .311672525

-0 .29476267

-0 .329104128

-0 .335725563

0 .322207032

-0 .337909376

1 .324407166

2 .585284168

0 .788133548

-0 .449718803

-0 .078569348

-0 . 144920927

0 .812165896

0 .569301345

0 .047231357

1 .00351808

-0 .601128557

1 . 161623056

-0 .267186135

-2 .073136614

-0 .794274513

-0 .3188745

0 .398357424

0 .306842156

-2 .275315404

-0 .466514016

0 .278531904

-0 .040058752

-0 . 147432159

1 .331459767

0 .255093068

1 . 143490308

0 .423698719

-0 .29963648

0 .419174935

0 .046329744

0 .775698123

0 .92793087

0 . 129643069

-0 . 168960552

-0 . 162994478

0 .713742411

-0 .373589658

-0 . 197484365

1 .58390186

0 .093799253

0 .952661003

1 .581469718

-0 .8022834

0 .000723123

-0 .622660957

0 .222073975

-1 . 1651509

0 . 149929054

0 .24732 0 .54708 0 .25913 0 . 1589 0 . 1 1529 0 . 10868 0 .23102 0 .51143 0 . 17796 0 . 19236 0 .23928 0 .09157 0 .0611 1 0 . 10846 0 . 13755 0 . 10906 0 .06397 0 . 13706 0 . 16331 0 .31468 0 .26187 0 .41248 0 . 14788 0 . 1 1638 0 . 12081 0 .09123 0 .23337 0 .09975 0 . 12568 0 .26462 0 . 10292 0 . 10164 0 . 1 1 136 0 . 13196 0 .09953 0 . 10791 0 . 12404 0 .08203 0 . 19881 0 . 10712 0 .09225 0 .24429 0 . 15696 0 . 1 1265 0 .08811 0 .08112 0 . 16441 0 .2161 0 . 1 1347 0 . 16555 0 . 19139 0 . 17569 0 . 16438 0 . 1 1 187 0 .21156 0 . 1551

-7 .2677 -5 .0588 8 .92097 -1 .8551 -2 .8545 -3 .0892 1 .39472 -0 .6607 7 .44218 13 .4396 3 .29384 -4 .9114 -1 .2857 -1 .3361 5 .90469 5 .22016 0 .73832 7 .32175 -3 .681 3 .69148 -1 .0203 -5 .0261 -5 .3711 -2 .7399 3 .29732 3 .36343 -9 .75 -4 .677 2 .21627 -0 . 1514 -1 .4325 13 .0994 2 .29068 8 .66531 4 .25694 -2 .7767 3 .37926 0 .56479 3 .90165 8 .66245 1 .40542 -0 .6916 -1 .0384 6 .33588 -4 .2402 -2 .4346 9 .63402 0 .43406 8 .39537 9 .55255 -4 . 1919 0 .00412 -3 .7879 1 .98508 -5 .5075 0 .96668

3 .66E-13 4 .22E-07 4 .62E-19 0 .06359 0 .00431 0 .00201 0 . 1631 0 .5088 9 .90E-14 3 .54E-41 0 .00099 9 .04E-07 0 . 19856 0 . 18151 3 .53E-09 1 .79E-07 0 .46032 2 .45E-13 0 .00023 0 .00022 0 .30759 5 .01E-07 7 .82E-08 0 .00615 0 .00098 0 .00077 1 .84E-22 2 .91E-06 0 .02667 0 .87967 0 . 152 3 .32E-39 0 .02198 4 .50E-18 2 .07E-05 0 .00549 0 .00073 0 .57222 9 .55E-05 4 .62E-18 0 . 1599 0 .48917 0 .29906 2 .36E-10 2 .23E-05 0 .01491 5 .74E-22 0 .66424 4 .64E-17 1 .27E-21 2 .77E-05 0 .99672 0 .00015 0 .04714 3 .64E-08 0 .3337

2 .54E-12 1 .33E-06 6 .35E-18 0 .08711 0 .00733 0 .0036 0 .20528 0 .56323 7 .27E-13 4 .59E-39 0 .00187 2 .72E-06 0 .24515 0 .2262 1 .49E-08 5 .95E-07 0 .51612 1 .73E-12 0 .00048 0 .00047 0 .36189 1 .56E-06 2 .76E-07 0 .01018 0 .00184 0 .00148 3 .67E-21 8 .08E-06 0 .0394 0 .89904 0 . 19247 3 .54E-37 0 .03296 5 .53E-17 5 . 10E-05 0 .00918 0 .0014 0 .62404 0 .00021 5 .67E-17 0 .20161 0 .54424 0 .35274 1 . 16E-09 5 .46E-05 0 .02298 1 .08E-20 0 .70924 5 .06E-16 2 .30E-20 6 .66E-05 0 .99739 0 .00032 0 .0663 1 .35E-07 0 .38867

| ABT1 2348 .359122 -0 .059356826 | 0 . 10511 -0 .5647 | 0 .57227 | 0 .62407 |
| --- | --- | --- | --- |
| ABTB1 995 .9187148 -0 .083245151 | 0 .09603 -0 .8669 | 0 .38601 | 0 .44237 |
| ABTB2 1459 .80972 0 .015495903 | 0 . 15485 0 . 10007 | 0 .92029 | 0 .93377 |
| AC003002 .6 16 .43169783 0 .088983892 | 0 . 18826 0 .47266 | 0 .63646 | 0 .68399 |
| AC004076 .7 7 .234378977 -0 . 12838455 | 0 .22794 -0 .5632 | 0 .57327 | 0 .62501 |
| AC004076 .9 9 .251767673 -0 .49862019 | 0 .29557 -1 .687 | 0 .0916 | 0 . 12179 |
| AC004381 .6 319 .6843783 -0 .032239264 | 0 .09138 -0 .3528 | 0 .72423 | 0 .76364 |
| AC005008 .2 3 .827126219 -3 .520152677 | 0 .63819 -5 .5158 | 3 .47E-08 | 1 .29E-07 |
| AC005481 .5 12 .08822652 -0 .621568219 | 0 .39702 -1 .5656 | 0 . 1 1745 | 0 . 15249 |
| AC005779 .2 1 .742506014 -1 .603816971 | 0 .52649 -3 .0462 | 0 .00232 | 0 .00412 |
| AC006116 .20189 .0204349 -0 .813421214 | 0 . 19677 -4 . 1339 | 3 .57E-05 | 8 .43E-05 |
| AC006126 .3 5 .656625959 -1 .046452805 | 0 .32009 -3 .2692 | 0 .00108 | 0 .00202 |
| AC006946 . 157 .608575621 -0 .876498607 | 0 .34506 -2 .5401 | 0 .01108 | 0 .01746 |
| AC007040 . 1 129 .59329557 0 . 175489441 | 0 .27616 0 .63546 | 0 .52513 | 0 .57921 |
| AC007192 .4 1 .494673648 -1 . 198512841 | 0 .59585 -2 .0114 | 0 .04428 | 0 .06264 |
| AC007557 . 1 0 .080215514 -0 .630660998 | 3 .0711 1 -0 .2054 | 0 .8373 | 0 .86227 |
| AC007731 . 1 1 1 .57280381 -0 . 147265662 | 0 .39548 -0 .3724 | 0 .70961 | 0 .75066 |
| AC007952 .5 10 .29286165 -1 .683118301 | 0 .39452 -4 .2662 | 1 .99E-05 | 4 .90E-05 |
| AC008060 .7 2 .804399656 -2 . 101065108 | 0 .90552 -2 .3203 | 0 .02032 | 0 .03067 |
| AC008132 . 1317 .39114218 -2 .779715539 | 0 .62831 -4 .4241 | 9 .68E-06 | 2 .49E-05 |
| AC008132 . 156 .989522331 -0 . 172256814 | 0 .83038 -0 .2074 | 0 .83566 | 0 .86084 |
| AC008271 . 1 8 .01920695 -2 .456807441 | 0 .74389 -3 .3027 | 0 .00096 | 0 .00181 |
| AC009403 .2 1 17 . 1534304 0 .334921307 | 0 . 10818 3 .09587 | 0 .00196 | 0 .00353 |
| AC009892 . 1**0** .894523386 -2 .576132278 | 0 .88562 -2 .9089 | 0 .00363 | 0 .00626 |
| AC010642 . 1 1 12 .0498901 -0 . 105671127 | 0 . 14981 -0 .7053 | 0 .48059 | 0 .53585 |
| AC011239 . 1 32 .60236701 -1 .006730234 | 0 .3098 -3 .2496 | 0 .00116 | 0 .00216 |
| AC011294 .3 203 .2298244 2 .34514576 | 0 .4233 5 .54012 | 3 .02E-08 | 1 . 13E-07 |
| AC011366 .3 1 .42507283 -3 .05263023 | 0 .67803 -4 .5022 | 6 .72E-06 | 1 .77E-05 |
| AC011551 .3 15 .01107755 -1 .203279159 | 0 .21966 -5 .4779 | 4 .31E-08 | 1 .57E-07 |
| AC011997 . 1 10 .27354334 -0 .375252622 | 0 .39003 -0 .9621 | 0 .33599 | 0 .39113 |
| AC012123 . 1 6 .521377838 -1 .41956515 | 0 .55096 -2 .5766 | 0 .00998 | 0 .01588 |
| AC013461 . 1 4940 .924243 0 . 17604042 | 0 . 1 1657 1 .51019 | 0 . 13099 | 0 . 16826 |
| AC015688 .3 0 .09301354 -0 .46094008 | 2 .20613 -0 .2089 | 0 .8345 | 0 .86005 |
| AC016757 .3 181 .8859596 -0 .036729676 | 0 .28135 -0 . 1305 | 0 .89613 | 0 .91327 |
| AC017104 .2 29 .08340877 -1 .469547643 | 0 .22237 -6 .6085 | 3 .88E-1 1 | 2 . 1 1E-10 |
| AC018816 .3 48 .63028984 -0 . 124003392 | 0 . 14187 -0 .8741 | 0 .38208 | 0 .43828 |
| AC021218 .2 13 .36392989 -0 .274758017 | 0 .65743 -0 .4179 | 0 .676 | 0 .71967 |
| AC023469 . 1 88 .07651609 2 .445876289 | 0 .55388 4 .41592 | 1 .01E-05 | 2 .58E-05 |
| AC023590 . 1 45 .26624839 -0 . 107499235 | 0 .25384 -0 .4235 | 0 .67194 | 0 .71606 |
| AC037459 .4 0 . 139637508 -0 .07119628 | 1 .70863 -0 .0417 | 0 .96676 | 0 .97173 |
| AC062017 . 1 49 .82388678 -1 . 100494773 | 0 . 17264 -6 .3746 | 1 .83E-10 | 9 . 15E-10 |
| AC064874 . 1 37 .57876755 -0 .329698498 | 0 .2299 -1 .4341 | 0 . 15154 | 0 . 19196 |
| AC066615 . 1 0 .720216379 0 . 132977336 | 0 .70664 0 . 18818 | 0 .85073 | 0 .87406 |
| AC069368 .3 5 . 122869582 0 . 183640848 | 0 .35604 0 .51579 | 0 .606 | 0 .65574 |
| AC074389 .6 13 .22579336 -0 .589549156 | 0 .39487 -1 .493 | 0 . 13543 | 0 . 17344 |
| AC079612 . 1 4 .286138794 -3 .548332883 | 0 .73176 -4 .849 | 1 .24E-06 | 3 .64E-06 |
| AC083864 .4 4 .784630988 -1 .916857328 | 0 .51893 -3 .6939 | 0 .00022 | 0 .00046 |
| AC090616 .2 32 .26219269 -0 .516224234 | 0 .28136 -1 .8347 | 0 .06654 | 0 .09084 |
| AC091801 . 1 9 .93198154 -1 .557701483 | 0 .51333 -3 .0345 | 0 .00241 | 0 .00428 |
| AC092675 .3 3 .854314154 -3 .224797489 | 0 .58777 -5 .4865 | 4 . 10E-08 | 1 .50E-07 |
| AC092687 .4 1 .28786565 -3 .823631824 | 0 .84366 -4 .5322 | 5 .84E-06 | 1 .55E-05 |
| AC093802 . 1 5 .45206864 -3 .729627376 | 0 .72521 -5 . 1428 | 2 .71E-07 | 8 .80E-07 |
| AC097381 . 1 21 .70924196 0 .356803108 | 0 .24464 1 .45851 | 0 . 1447 | 0 . 18405 |
| AC099552 .4 3 .95048876 -0 .939397431 | 0 .52153 -1 .8012 | 0 .07167 | 0 .09719 |
| AC102948 .2 1 .899808874 -4 .631212451 | 0 .98072 -4 .7223 | 2 .33E-06 | 6 .56E-06 |
| AC104532 .2 0 . 128958643 -0 . 184348372 | 1 .71336 -0 . 1076 | 0 .91432 | 0 .9287 |

AC104667 .3 56 .34153235 AC104794 .4 0 .919384488 AC106873 .4 44 .71637892 AC106876 .2 3 .577945499 AC108938 .5 45 .21889497 AC109829 . 1 10 .02336016 AC110781 .3 109 .4472142 AC112715 .2 7 .600339552 AC112721 . 1 21 .64247611 AC114783 . 1 2 .516878539 AC129492 .6 32 .56760896 AC131097 .4 16 .39859608 AC137932 .4 87 .36486496 AC138647 . 1 2 . 1 14900658 AC138969 .4 20 .32812727 AC144568 .2 0 .814482976 AC145676 .2 43 .6253322

| ACAA1 ACAA2 ACACB ACAD10 ACAD11 ACAD8 ACAD9 ACADL ACADM ACADS ACADSB ACADVL ACAN ACAP1 ACAP2 ACAP3 ACAT1 ACAT2 ACBD3 ACBD4 ACBD5 ACBD7 ACCS ACCSL ACD ACE ACE2 ACER1 ACER2 ACER3 ACHE ACIN1 ACKR1 ACKR2 ACKR3 ACKR4 ACLY ACMSD ACO1 | 1639 .877722 3080 .82948 2166 .043287 1522 .490418 47 .93030017 2936 .946676 3149 .573307 277 .9477541 3363 .551466 1398 .392739 2569 .043378 10678 .84349 225 .7924202 940 .6750278 14724 .32869 2821 .872373 4944 .65205 2273 .044004 4970 .087659 374 .8811237 3110 .41326 17 .89157554 1625 .076721 145 .4849752 649 .8057256 475 . 1931599 72 .83355867 766 . 1909641 327 . 1372663 3943 .30989 70 .87454298 5587 .291175 869 .6942156 172 .5560516 1025 .01257 1 128 .539204 10671 .03895 45 .58549596 4442 .721856 |
| --- | --- |

1 .435859534

-3 .226294408

-0 .275013691

-1 .062048894

1 .650080021

-1 . 158737621

-0 .055865113

-0 .397171983

2 .211 103758

-4 . 10272713

-0 .261830907

-0 .601601934

-0 .067949057

-1 .501970309

0 .221721369

0 .219050467

-0 .050030524

0 .42521803

0 .686887259

0 . 10167811

0 . 13001317

-1 .051772718

-0 .094915821

-0 .768628469

-2 .710284045

0 .390089553

0 .811037557

-0 .444670468

0 .271159511

2 .538319551

1 . 166194116

0 .039458939

0 . 17151028

-0 .689961108

0 .06152341

0 .40542612

-0 .053364098

-0 .041439763

0 .58194841

-0 .496806648

-1 .537876826

0 .006997751

2 .318844089

-0 .348214787

-1 . 182335759

-0 .676313563

0 .799741528

0 .810232806

0 . 100627007

1 .263871874

1 .092377622

0 .747703454

-1 .93817749

0 .980662172

-1 . 142993794

0 .462595406

0 .28479 0 .73656 0 .23118 0 .74888 0 .3268 0 .29754 0 .2254 0 .30325 0 .3049 0 .99162 0 .28788 0 .28533 0 . 15135 0 .485 0 .25189 0 .77565 0 .23293 0 . 10062 0 . 18206 0 . 18344 0 .08343 0 . 19018 0 . 14246 0 . 10522 0 .29849 0 . 18773 0 . 1 1862 0 .09266 0 . 1 1041 0 .29325 0 . 16665 0 .09246 0 .09841 0 . 12691 0 .24437 0 .08498 0 . 10092 0 .08173 0 .26871 0 . 14964 0 .29633 0 . 1 1484 0 . 1713 0 .28663 0 .31206 0 . 16957 0 . 10576 0 .21274 0 .09599 0 .23011 0 . 1971 0 . 19959 0 . 18016 0 .09193 0 .20634 0 . 1786

5 .04189 -4 .3802 -1 . 1896 -1 .4182 5 .04915 -3 .8945 -0 .2479 -1 .3097 7 .25186 -4 . 1374 -0 .9095 -2 . 1085 -0 .4489 -3 .0968 0 .88022 0 .28241 -0 .2148 4 .22589 3 .77293 0 .55428 1 .55839 -5 .5305 -0 .6663 -7 .3048 -9 .0799 2 .07797 6 .83744 -4 .7988 2 .45599 8 .65586 6 .99778 0 .42679 1 .74281 -5 .4365 0 .25176 4 .77091 -0 .5288 -0 .507 2 . 16571 -3 .3201 -5 . 1897 0 .06093 13 .5364 -1 .2149 -3 .7888 -3 .9884 7 .56218 3 .80856 1 .04826 5 .49259 5 .5422 3 .74621 -10 .758 10 .6675 -5 .5393 2 .59015

4 .61E-07 1 . 19E-05 0 .23421 0 . 15614 4 .44E-07 9 .84E-05 0 .80425 0 . 19028 4 . 1 1E-13 3 .51E-05 0 .36308 0 .03499 0 .65347 0 .00196 0 .37874 0 .77763 0 .82993 2 .38E-05 0 .00016 0 .57938 0 . 1 1914 3 . 19E-08 0 .50524 2 .78E-13 1 .09E-19 0 .03771 8 .06E-12 1 .60E-06 0 .01405 4 .89E-18 2 .60E-12 0 .66953 0 .08137 5 .43E-08 0 .80122 1 .83E-06 0 .59694 0 .61214 0 .03033 0 .0009 2 . 1 1E-07 0 .95141 9 .53E-42 0 .22442 0 .00015 6 .65E-05 3 .96E-14 0 .00014 0 .29452 3 .96E-08 2 .99E-08 0 .00018 5 .42E-27 1 .44E-26 3 .04E-08 0 .00959

1 .45E-06 3 .02E-05 0 .28383 0 . 19727 1 .40E-06 0 .00022 0 .83367 0 .23605 2 .83E-12 8 .32E-05 0 .41877 0 .05042 0 .69933 0 .00352 0 .43487 0 .80969 0 .8563 5 .79E-05 0 .00034 0 .63085 0 . 15446 1 . 19E-07 0 .55997 1 .95E-12 1 .62E-18 0 .054 4 .74E-1 1 4 .60E-06 0 .02175 5 .98E-17 1 .64E-1 1 0 .71385 0 . 10923 1 .96E-07 0 .83085 5 .24E-06 0 .64747 0 .66152 0 .04428 0 .00171 6 .95E-07 0 .95906 1 .29E-39 0 .27321 0 .00032 0 .00015 3 .08E-13 0 .0003 0 .34804 1 .45E-07 1 . 12E-07 0 .00038 1 .78E-25 4 .46E-25 1 . 14E-07 0 .01531

| ACO2 | 3295 . 162302 | 0 .446392657 | 0 . 10225 | 4 .36586 | 1 .27E-05 | 3 .21E-05 |
| --- | --- | --- | --- | --- | --- | --- |
| ACOD1 | 1 1 . 19731802 | -0 .373998071 | 0 .46003 | -0 .813 | 0 .41623 | 0 .47285 |
| ACOT1 | 505 . 1070793 | 0 .706231999 | 0 .31749 | 2 .22441 | 0 .02612 | 0 .03867 |
| ACOT11 | 730 .5630734 | -1 .247457982 | 0 . 15088 | -8 .2681 | 1 .36E-16 | 1 .41E-15 |
| ACOT12 | 19 .5441759 | -1 .871339898 | 0 .4279 | -4 .3733 | 1 .22E-05 | 3 . 1 1E-05 |
| ACOT13 | 1666 .741155 | -0 . 163814477 | 0 .07339 | -2 .2322 | 0 .0256 | 0 .03794 |
| ACOT2 | 1795 .947068 | 0 .821071958 | 0 . 14388 | 5 .7066 | 1 . 15E-08 | 4 .56E-08 |
| ACOT4 | 276 .657172 | 0 .459079686 | 0 .20139 | 2 .27952 | 0 .02264 | 0 .03385 |
| ACOT6 | 3 . 185770455 | -1 .794178818 | 0 .46628 | -3 .8479 | 0 .00012 | 0 .00026 |
| ACOT7 | 2699 .768351 | 1 . 143972478 | 0 . 17168 | 6 .66335 | 2 .68E-1 1 | 1 .49E-10 |
| ACOT8 | 1051 .941756 | -0 .431997382 | 0 .08151 | -5 .3001 | 1 . 16E-07 | 3 .98E-07 |
| ACOT9 | 2945 .357904 | 0 .596776427 | 0 .09846 | 6 .0614 | 1 .35E-09 | 6 .03E-09 |
| ACOX1 | 1 1548 .00775 | -0 . 149634022 | 0 .09571 | -1 .5634 | 0 . 1 1796 | 0 . 1531 |
| ACOX2 | 464 .7558075 | 0 .058083567 | 0 .30217 | 0 . 19222 | 0 .84757 | 0 .87147 |
| ACOX3 | 1617 .252932 | 0 . 141398378 | 0 .08011 | 1 .76501 | 0 .07756 | 0 . 10457 |
| ACOXL | 36 .37759986 | -0 .716483654 | 0 .41276 | -1 .7359 | 0 .08259 | 0 . 1 1076 |
| ACP1 | 4930 .74174 | -0 .300424769 | 0 .09854 | -3 .0489 | 0 .0023 | 0 .00409 |
| ACP2 | 1452 .761104 | 0 .947921093 | 0 . 1 1973 | 7 .91712 | 2 .43E-15 | 2 . 17E-14 |
| ACP5 | 5933 .552818 | 0 .517323022 | 0 .21386 | 2 .41899 | 0 .01556 | 0 .0239 |
| ACP6 | 1015 .541723 | -0 .250917851 | 0 . 15532 | -1 .6155 | 0 . 1062 | 0 . 13914 |
| ACP7 | 619 .8218407 | -0 . 137807929 | 0 .25803 | -0 .5341 | 0 .59328 | 0 .64406 |
| ACPP | 1909 .58777 | -0 .903554219 | 0 .22078 | -4 .0926 | 4 .27E-05 | 9 .97E-05 |
| ACPT | 47 .26706477 | -0 .426414776 | 0 . 16614 | -2 .5665 | 0 .01027 | 0 .01629 |
| ACR | 10 .81521227 | -0 .646297038 | 0 .37654 | -1 .7164 | 0 .08609 | 0 . 1 1502 |
| ACRBP | 45 . 12330335 | 0 .460718006 | 0 . 19629 | 2 .34712 | 0 .01892 | 0 .02869 |
| ACRC | 175 .5618505 | -0 .612467839 | 0 . 1 1251 | -5 .4436 | 5 .22E-08 | 1 .88E-07 |
| ACRV1 | 32 . 18140588 | -0 .276228188 | 0 . 19694 | -1 .4026 | 0 . 16073 | 0 .20257 |
| ACSBG1 | 3772 .921433 | 1 .29513373 | 0 .47738 | 2 .713 | 0 .00667 | 0 .01096 |
| ACSBG2 | 31 . 1 123927 | -1 .098823144 | 0 .2214 | -4 .963 | 6 .94E-07 | 2 . 12E-06 |
| ACSF2 | 1452 .279262 | -0 . 163367678 | 0 . 12734 | -1 .2829 | 0 . 19952 | 0 .24613 |
| ACSF3 | 1456 .720256 | 0 .56154828 | 0 .07175 | 7 .82629 | 5 .02E-15 | 4 .31E-14 |
| ACSL1 | 25205 .39101 | 0 .821193225 | 0 .26908 | 3 .0519 | 0 .00227 | 0 .00405 |
| ACSL3 | 1 1558 .69707 | 1 . 131411672 | 0 . 1608 | 7 .03611 | 1 .98E-12 | 1 .26E-1 1 |
| ACSL4 | 7637 .349106 | 0 .542612858 | 0 . 1 1332 | 4 .7885 | 1 .68E-06 | 4 .83E-06 |
| ACSL5 | 1329 .425762 | 1 . 1 17205045 | 0 . 18356 | 6 .0864 | 1 . 15E-09 | 5 .20E-09 |
| ACSL6 | 351 .7033662 | -1 .270849017 | 0 .22362 | -5 .6831 | 1 .32E-08 | 5 . 19E-08 |
| ACSM1 | 39 .05882053 | -0 .965254753 | 0 .30333 | -3 . 1822 | 0 .00146 | 0 .00268 |
| ACSM2A | 24 .61231959 | -4 .638007415 | 0 .60523 | -7 .6632 | 1 .81E-14 | 1 .46E-13 |
| ACSM2B | 24 .61323288 | -4 .674838802 | 0 .54324 | -8 .6054 | 7 .60E-18 | 9 .08E-17 |
| ACSM3 | 496 . 1 141906 | 0 .390417488 | 0 .38028 | 1 .02666 | 0 .30458 | 0 .35863 |
| ACSM4 | 33 .71844427 | -0 .584367671 | 0 .23787 | -2 .4566 | 0 .01402 | 0 .02171 |
| ACSM5 | 283 .6848559 | 1 .099418237 | 0 .22929 | 4 .79488 | 1 .63E-06 | 4 .68E-06 |
| ACSM6 | 238 .826411 1 | 0 .871271214 | 0 .44022 | 1 .97917 | 0 .0478 | 0 .06718 |
| ACSS1 | 2079 .866832 | -0 .338155469 | 0 . 1329 | -2 .5444 | 0 .01095 | 0 .01727 |
| ACSS2 | 3299 .71799 | -0 . 1 10231379 | 0 . 16705 | -0 .6599 | 0 .50933 | 0 .56375 |
| ACSS3 | 983 .7654128 | -0 . 130261504 | 0 .26871 | -0 .4848 | 0 .62785 | 0 .67629 |
| ACTA1 | 52 .55669714 | 3 .273893719 | 0 .42385 | 7 .7242 | 1 . 13E-14 | 9 .22E-14 |
| ACTA2 | 3646 . 147232 | 1 .869700082 | 0 .22452 | 8 .32756 | 8 .25E-17 | 8 .78E-16 |
| ACTB | 67253 .63571 | 1 .805791894 | 0 . 13266 | 13 .6123 | 3 .38E-42 | 4 .77E-40 |
| ACTBL2 | 59 .47253925 | -0 .683790559 | 0 .36296 | -1 .8839 | 0 .05958 | 0 .08212 |
| ACTC1 | 70 .45580325 | 1 .700618431 | 0 .48832 | 3 .4826 | 0 .0005 | 0 .00098 |
| ACTG1 | 94488 .71202 | 0 .322057117 | 0 . 1 1499 | 2 .80076 | 0 .0051 | 0 .00856 |
| ACTG2 | 296 .4097622 | 3 .002644188 | 0 .40038 | 7 .49947 | 6 .41E-14 | 4 .83E-13 |
| ACTL10 | 168 .7229787 | 0 .703554447 | 0 . 14502 | 4 .85138 | 1 .23E-06 | 3 .60E-06 |
| ACTL6A | 2807 .491087 | -0 . 128040927 | 0 . 10694 | -1 . 1974 | 0 .23116 | 0 .28062 |
| ACTL6B | 5 .407566521 | -2 .537612211 | 0 .50418 | -5 .0332 | 4 .82E-07 | 1 .51E-06 |

| ACTL7A 9 .97253072 | -1 .577940764 | 0 .30627 | -5 . 1521 | 2 .58E-07 | 8 .40E-07 |
| --- | --- | --- | --- | --- | --- |
| ACTL7B 5 .576225323 | -1 .327975816 | 0 .41515 | -3 . 1987 | 0 .00138 | 0 .00255 |
| ACTL8 7 .07911425 | -0 .039418343 | 0 .60703 | -0 .0649 | 0 .94822 | 0 .9566 |
| ACTL9 1 .752129262 | -0 .409182992 | 0 .65063 | -0 .6289 | 0 .52941 | 0 .58316 |
| ACTN1 6340 .914704 | 1 .608305667 | 0 . 13913 | 1 1 .5599 | 6 .58E-31 | 3 . 10E-29 |
| ACTN2 71 .35300419 | -0 .591660568 | 0 .28057 | -2 . 1088 | 0 .03496 | 0 .05038 |
| ACTN4 1 1066 .49228 | 0 .226293148 | 0 . 12683 | 1 .78422 | 0 .07439 | 0 . 10067 |
| ACTR10 3760 .639636 | -0 .377453529 | 0 .08297 | -4 .5492 | 5 .38E-06 | 1 .44E-05 |
| ACTR1A 6024 .506233 | 0 .238307292 | 0 .0729 | 3 .26912 | 0 .00108 | 0 .00202 |
| ACTR1B 4372 .068719 | -0 .210779806 | 0 . 10095 | -2 .0879 | 0 .0368 | 0 .05281 |
| ACTR2 15797 .85045 | 0 .551930047 | 0 .0921 | 5 .99275 | 2 .06E-09 | 9 .01E-09 |
| ACTR3 20699 .39141 | 0 . 106093012 | 0 .08605 | 1 .2329 | 0 .21761 | 0 .26569 |
| ACTR3B 500 .8275884 | 0 .034174637 | 0 .09979 | 0 .34248 | 0 .73199 | 0 .77013 |
| ACTR3C 439 .4754941 | -0 .614266469 | 0 . 16434 | -3 .7378 | 0 .00019 | 0 .00039 |
| ACTR5 885 .8221399 | -0 . 132608932 | 0 .0822 | -1 .6133 | 0 . 10667 | 0 . 13971 |
| ACTR6 2009 .896574 | -0 .453253869 | 0 . 1 1716 | -3 .8687 | 0 .00011 | 0 .00024 |
| ACTR8 3276 .666309 | -0 .046966851 | 0 .09506 | -0 .4941 | 0 .62124 | 0 .67026 |
| ACTRT1 2 .647069527 | -4 .376584601 | 0 .83342 | -5 .2514 | 1 .51E-07 | 5 .09E-07 |
| ACTRT2 0 .941096428 | -2 .864149691 | 0 .99627 | -2 .8749 | 0 .00404 | 0 .00692 |
| ACTRT3 137 .218617 | -0 . 129163491 | 0 . 15675 | -0 .824 | 0 .40992 | 0 .46643 |
| ACVR1 2437 .32012 | 0 .526561193 | 0 . 10044 | 5 .24229 | 1 .59E-07 | 5 .32E-07 |
| ACVR1B 1793 .568519 | -0 .029500956 | 0 . 13601 | -0 .2169 | 0 .82828 | 0 .85493 |
| ACVR1C 246 .4881179 | 1 .242963344 | 0 .23485 | 5 .29248 | 1 .21E-07 | 4 . 14E-07 |
| ACVR2A 3307 .480672 | -0 .874255184 | 0 . 1203 | -7 .2672 | 3 .67E-13 | 2 .54E-12 |
| ACVR2B 2350 .806102 | -0 .263946129 | 0 . 12452 | -2 . 1 196 | 0 .03404 | 0 .04916 |
| ACVRL1 2035 .082479 | 1 .426818369 | 0 . 1406 | 10 . 1482 | 3 .37E-24 | 8 . 14E-23 |
| ACY1 88 .864411 | 0 .042635401 | 0 . 13425 | 0 .31759 | 0 .7508 | 0 .78621 |
| ACY3 1 1 .20129565 | 0 .922132991 | 0 .39476 | 2 .33594 | 0 .01949 | 0 .02949 |
| ACYP1 687 .7748806 | -0 .321491544 | 0 . 12739 | -2 .5236 | 0 .01162 | 0 .01825 |
| ACYP2 845 .8652993 | -0 .201477896 | 0 .08848 | -2 .2772 | 0 .02278 | 0 .03405 |
| ADA 445 .9911753 | 1 .290284278 | 0 . 12392 | 10 .4122 | 2 . 18E-25 | 5 .97E-24 |
| ADAD1 10 .98414849 | -3 . 1075944 | 0 .68764 | -4 .5192 | 6 .21E-06 | 1 .64E-05 |
| ADAD2 29 .68731976 | -1 .350902854 | 0 .25649 | -5 .2669 | 1 .39E-07 | 4 .71E-07 |
| ADAL 720 .2803578 | -0 .075979383 | 0 . 1 1 185 | -0 .6793 | 0 .49697 | 0 .55182 |
| ADAM10 15039 .29951 | 0 .70351909 | 0 . 13758 | 5 . 1 1338 | 3 . 16E-07 | 1 .02E-06 |
| ADAM11 1 16 . 1673822 | 0 . 195996751 | 0 .2113 | 0 .92758 | 0 .35362 | 0 .40914 |
| ADAM12 1262 .51641 | 2 . 165378581 | 0 .22427 | 9 .65538 | 4 .66E-22 | 8 .86E-21 |
| ADAM15 8438 .347041 | 0 . 187185085 | 0 .08728 | 2 . 14472 | 0 .03198 | 0 .04643 |
| ADAM17 2860 .509588 | 0 . 134389184 | 0 .06497 | 2 .06838 | 0 .0386 | 0 .05517 |
| ADAM18 15 .42058604 | -4 .909104551 | 0 .94369 | -5 .202 | 1 .97E-07 | 6 .53E-07 |
| ADAM19 1532 .567475 | 2 .276836874 | 0 . 16372 | 13 .9073 | 5 .72E-44 | 9 .98E-42 |
| ADAM2 13 .79818987 | -3 .419984132 | 1 .07669 | -3 . 1764 | 0 .00149 | 0 .00273 |
| ADAM20 1 17 .5310694 | -1 .352011397 | 0 .22811 | -5 .9271 | 3 .08E-09 | 1 .31E-08 |
| ADAM21 45 .94529095 | -1 .345793041 | 0 . 18061 | -7 .4513 | 9 .24E-14 | 6 .81E-13 |
| ADAM22 1086 .712876 | -0 .517355583 | 0 . 16935 | -3 .0549 | 0 .00225 | 0 .00401 |
| ADAM23 2838 .095286 | -1 .784097698 | 0 .28838 | -6 . 1867 | 6 . 14E-10 | 2 .87E-09 |
| ADAM28 1059 .771723 | 0 .798533095 | 0 . 16735 | 4 .77155 | 1 .83E-06 | 5 .22E-06 |
| ADAM29 18 .02548959 | -2 .885131818 | 0 .61303 | -4 .7064 | 2 .52E-06 | 7 .05E-06 |
| ADAM30 6 .813899712 | -4 .800410194 | 0 .68352 | -7 .0231 | 2 . 17E-12 | 1 .38E-1 1 |
| ADAM32 632 .6117148 | -1 .764630061 | 0 .22816 | -7 .7342 | 1 .04E-14 | 8 .57E-14 |
| ADAM33 1 125 .809703 | 0 .591587003 | 0 .25104 | 2 .35654 | 0 .01845 | 0 .02801 |
| ADAM7 20 .51673564 | -3 .361683616 | 0 .55345 | -6 .0741 | 1 .25E-09 | 5 .58E-09 |
| ADAM8 1035 .564332 | 1 .911979261 | 0 . 16257 | 1 1 .7611 | 6 . 19E-32 | 3 .30E-30 |
| ADAM9 7330 .063285 | 0 .457231907 | 0 . 1076 | 4 .24922 | 2 . 15E-05 | 5 .26E-05 |
| ADAMDEC1 2141 .387912 | 5 . 130844945 | 0 .31266 | 16 .4104 | 1 .61E-60 | 1 .63E-57 |
| ADAMTS1 4533 .605661 | -0 . 127965465 | 0 . 15034 | -0 .8512 | 0 .39468 | 0 .45123 |

| ADAMTS10 ADAMTS12 ADAMTS13 ADAMTS14 ADAMTS15 ADAMTS16 ADAMTS17 ADAMTS18 ADAMTS19 ADAMTS2 ADAMTS20 ADAMTS3 ADAMTS4 ADAMTS5 ADAMTS6 ADAMTS7 ADAMTS8 ADAMTS9 ADAMTSL1 ADAMTSL2 ADAMTSL3 ADAMTSL4 | 723 . 1816233 1535 .336885 201 . 1612277 168 .3483956 752 .0671839 159 .4840549 425 .3260435 57 .75128963 980 .8666143 3643 .894926 39 .70197268 430 .3371376 822 .2213588 3522 .665653 275 .3149859 414 .578219 124 .6283631 1243 .300328 664 . 1421371 135 .2618661 424 .326343 668 .7813579 |
| --- | --- |

ADAMTSL4-A54 .93955613

| ADAMTSL5 ADAP1 ADAP2 ADAR ADARB1 ADARB2 ADAT1 ADAT2 ADAT3 ADCK1 ADCK2 ADCK3 ADCK4 ADCK5 ADCY1 ADCY10 ADCY2 ADCY3 ADCY4 ADCY5 ADCY6 ADCY7 ADCY8 ADCY9 ADCYAP1 | 85 .96133469 179 .7614364 1336 . 13534 20232 .53282 2824 .714842 48 . 10132842 1949 .525198 1031 . 134844 1 13 .6079984 531 .6658227 1732 .499685 2952 .596231 1534 .778645 646 .49272 1810 .991795 48 .53646732 1478 .243041 1895 .529005 533 .3407028 259 . 1 168637 2343 .745652 2074 .855756 78 .75369464 2182 .797456 250 .7473192 |
| --- | --- |

ADCYAP1R1373 .0542784

| ADD1 ADD2 ADD3 ADD3-AS1 ADGB ADGRA1 ADGRA2 | 9922 .404096 154 . 1404658 18165 .62441 162 .9553644 68 .46460015 6 .241861784 3388 .54565 |
| --- | --- |

0 .892935753

1 .543022235

0 .347318039

0 .860049217

1 .459734949

1 .822911087

0 .669138227

-0 .279383034

-6 .064521616

2 .810083356

-1 .820848371

-0 .746916915

2 .592420928

0 .434590518

0 .423986749

1 .550846062

-0 .843436577

0 .716525548

0 .7443074

2 .084627239

-1 .328170337

1 . 1 18150145

-0 .553382286

-0 .438754054

1 .361370552

1 . 152594112

0 .830296196

0 .663471851

-1 .537354121

0 .966497609

-0 .620332533

0 .556355193

0 .600249924

0 .392707863

-0 .352474907

0 .453394191

0 .675759139

2 .967547683

-1 .608009811

0 .740390465

0 .919847381

0 .774993374

0 .891439534

0 .591322225

1 .07293264

1 .474364086

0 .598002547

2 .392853262

0 .700975165

0 .329524119

0 .589521648

-1 .273621701

-3 .986914441

-2 .229500946

-2 .932763086

0 .780081083

0 . 19931 0 . 17856 0 . 1411 0 . 16195 0 .25981 0 .36815 0 . 17825 0 .3689 0 .26967 0 .20774 0 .60394 0 . 16676 0 .25382 0 .2014 0 . 16509 0 . 17832 0 .23698 0 . 14015 0 .24884 0 . 19715 0 .25663 0 . 14401 0 . 19137 0 . 15667 0 . 14514 0 . 1333 0 .09761 0 . 1 145 0 .29224 0 . 10015 0 . 13615 0 . 14377 0 . 10636 0 . 10906 0 .09007 0 .07627 0 . 10063 0 .30087 0 .25974 0 .26466 0 . 1 1298 0 . 19332 0 .23494 0 . 10416 0 . 14304 0 .4997 0 . 10779 0 .47335 0 .21398 0 .06989 0 .28581 0 . 14068 0 .23105 0 .28527 0 .61328 0 . 15919

4 .48011 8 .64172 2 .46158 5 .31061 5 .61842 4 .95155 3 .75401 -0 .7573 -22 .489 13 .5269 -3 .015 -4 .4789 10 .2136 2 . 15786 2 .56818 8 .6972 -3 .5591 5 . 1 1253 2 .99116 10 .5737 -5 . 1755 7 .76426 -2 .8917 -2 .8005 9 .3794 8 .64642 8 .50604 5 .79463 -5 .2606 9 .65087 -4 .5562 3 .86974 5 .64335 3 .60077 -3 .9133 5 .94455 6 .7156 9 .86324 -6 . 1908 2 .79748 8 . 14186 4 .00895 3 .79435 5 .67706 7 .5008 2 .9505 5 .54809 5 .05514 3 .27597 4 .71518 2 .06264 -9 .0534 -17 .256 -7 .8154 -4 .7821 4 .90025

7 .46E-06 5 .54E-18 0 .01383 1 .09E-07 1 .93E-08 7 .36E-07 0 .00017

0 .44885

#######

1 .09E-41 0 .00257 7 .50E-06 1 .72E-24 0 .03094 0 .01022 3 .40E-18 0 .00037 3 . 18E-07 0 .00278 3 .94E-26 2 .27E-07 8 .21E-15 0 .00383 0 .0051 6 .63E-21 5 .31E-18 1 .80E-17 6 .85E-09 1 .44E-07 4 .87E-22 5 .21E-06 0 .00011 1 .67E-08 0 .00032 9 . 1 1E-05 2 .77E-09 1 .87E-1 1 6 .01E-23 5 .98E-10 0 .00515 3 .89E-16 6 . 10E-05 0 .00015 1 .37E-08 6 .34E-14 0 .00317 2 .89E-08 4 .30E-07 0 .00105 2 .41E-06 0 .03915 1 .39E-19 1 .01E-66 5 .48E-15 1 .73E-06 9 .57E-07

1 .95E-05 6 .71E-17 0 .02144 3 .78E-07 7 .39E-08 2 .24E-06 0 .00037

0 .50468

#######

1 .45E-39 0 .00454 1 .96E-05 4 .29E-23 0 .04507 0 .01622 4 .23E-17 0 .00075 1 .02E-06 0 .00488 1 . 17E-24 7 .46E-07 6 .85E-14 0 .00658 0 .00857 1 . 12E-19 6 .46E-17 2 .07E-16 2 .80E-08 4 .86E-07 9 .23E-21 1 .39E-05 0 .00024 6 .46E-08 0 .00065 0 .0002 1 . 19E-08 1 .06E-10 1 .26E-21 2 .80E-09 0 .00864 3 .84E-15 0 .00014 0 .00032 5 .37E-08 4 .78E-13 0 .00552 1 .08E-07 1 .36E-06 0 .00198 6 .77E-06 0 .05591 2 .03E-18 1 .43E-63 4 .66E-14 4 .97E-06 2 .86E-06

| ADGRA3 | 5980 .588562 | -0 .680389979 | 0 . 12861 | -5 .2905 | 1 .22E-07 | 4 . 19E-07 |
| --- | --- | --- | --- | --- | --- | --- |
| ADGRB1 | 263 .0654174 | 1 .090478574 | 0 .29576 | 3 .68701 | 0 .00023 | 0 .00047 |
| ADGRB2 | 203 .8500556 | 1 .478862394 | 0 .20303 | 7 .28384 | 3 .24E-13 | 2 .26E-12 |
| ADGRB3 | 985 .497804 | -2 .920210332 | 0 .28814 | -10 . 135 | 3 .87E-24 | 9 .25E-23 |
| ADGRD1 | 691 .7726238 | 1 .01261401 | 0 .25965 | 3 .89998 | 9 .62E-05 | 0 .00021 |
| ADGRD2 | 16 .7695864 | -1 .070115658 | 0 .31821 | -3 .363 | 0 .00077 | 0 .00148 |
| ADGRE1 | 59 .00397336 | 0 .75269242 | 0 .29694 | 2 .5348 | 0 .01125 | 0 .01772 |
| ADGRE2 | 832 .8012321 | 2 .073925241 | 0 . 16371 | 12 .6683 | 8 .86E-37 | 7 .70E-35 |
| ADGRE3 | 36 . 12400427 | 0 .576435059 | 0 .47061 | 1 .22488 | 0 .22062 | 0 .26893 |
| ADGRE5 | 1923 .272013 | 1 . 193041089 | 0 . 1 1401 | 10 .4643 | 1 .26E-25 | 3 .54E-24 |
| ADGRF1 | 66 .23200382 | -0 .992986527 | 0 .44795 | -2 .2167 | 0 .02664 | 0 .03935 |
| ADGRF2 | 286 .4905685 | -1 .994011968 | 0 .2983 | -6 .6845 | 2 .32E-1 1 | 1 .29E-10 |
| ADGRF3 | 81 .61891133 | -0 .767591013 | 0 . 13433 | -5 .7141 | 1 . 10E-08 | 4 .38E-08 |
| ADGRF4 | 2076 .995406 | -1 .921672509 | 0 .26515 | -7 .2474 | 4 .25E-13 | 2 .92E-12 |
| ADGRF5 | 2527 .650556 | 1 . 102369554 | 0 . 14948 | 7 .37474 | 1 .65E-13 | 1 . 19E-12 |
| ADGRG1 | 7680 .745924 | 0 .202561385 | 0 . 15954 | 1 .26963 | 0 .20422 | 0 .25122 |
| ADGRG2 | 551 .7522625 | 0 . 1 1 1529621 | 0 .24032 | 0 .46408 | 0 .64259 | 0 .68946 |
| ADGRG3 | 48 .55532525 | 1 .273363168 | 0 .32738 | 3 .88952 | 0 .0001 | 0 .00022 |
| ADGRG4 | 34 . 17024849 | -4 . 146832476 | 0 .54863 | -7 .5585 | 4 .08E-14 | 3 . 16E-13 |
| ADGRG5 | 138 .6881789 | 2 . 1 13513484 | 0 .31185 | 6 .77729 | 1 .22E-1 1 | 7 .05E-1 1 |
| ADGRG6 | 4037 .40988 | -0 . 165343347 | 0 .20552 | -0 .8045 | 0 .4211 1 | 0 .4777 |
| ADGRG7 | 19 .23950446 | -2 .225463001 | 0 .48478 | -4 .5907 | 4 .42E-06 | 1 . 19E-05 |
| ADGRL1 | 1071 .903331 | 0 .733881848 | 0 . 14006 | 5 .23976 | 1 .61E-07 | 5 .39E-07 |
| ADGRL2 | 4604 .063195 | 0 .056285309 | 0 . 1642 | 0 .34279 | 0 .73176 | 0 .76993 |
| ADGRL3 | 3460 .384195 | -0 .358182519 | 0 .32543 | -1 . 1007 | 0 .27105 | 0 .32315 |
| ADGRL4 | 1637 .989587 | 1 .289633828 | 0 . 16339 | 7 .89293 | 2 .95E-15 | 2 .61E-14 |
| ADGRV1 | 1806 . 102512 | -3 .723686159 | 0 .3243 | -1 1 .482 | 1 .62E-30 | 7 .36E-29 |
| ADH1A | 39 . 13682292 | -1 .35157681 | 0 .30191 | -4 .4768 | 7 .58E-06 | 1 .98E-05 |
| ADH1B | 4182 .050215 | -0 .337251232 | 0 .41986 | -0 .8032 | 0 .42184 | 0 .4784 |
| ADH4 | 84 . 1 1660177 | -1 . 132839132 | 0 .29038 | -3 .9012 | 9 .57E-05 | 0 .00021 |
| ADH5 | 1 1 182 .35939 | -0 .321356154 | 0 . 10608 | -3 .0294 | 0 .00245 | 0 .00434 |
| ADH6 | 104 .6765566 | -1 .442634803 | 0 .21973 | -6 .5655 | 5 . 19E-1 1 | 2 .77E-10 |
| ADH7 | 193 .6103677 | 1 .415704694 | 0 .38855 | 3 .64355 | 0 .00027 | 0 .00055 |
| ADHFE1 | 260 .623594 | -0 .379608229 | 0 . 18283 | -2 .0762 | 0 .03787 | 0 .0542 |
| ADI1 | 4833 .795647 | 0 . 155571992 | 0 . 1019 | 1 .5267 | 0 . 12683 | 0 . 16337 |
| ADIG | 3 .686064576 | -2 .825253707 | 0 .42484 | -6 .6501 | 2 .93E-1 1 | 1 .62E-10 |
| ADIPOQ | 357 .7284751 | 3 .270002126 | 0 .63021 | 5 . 18872 | 2 . 12E-07 | 6 .98E-07 |
| ADIPOR1 | 9396 .051085 | -0 .018456775 | 0 . 10199 | -0 . 181 | 0 .8564 | 0 .87889 |
| ADIPOR2 | 7856 .788891 | 0 .415266862 | 0 .08951 | 4 .63931 | 3 .50E-06 | 9 .58E-06 |
| ADK | 3014 .887265 | -0 .625808247 | 0 .09813 | -6 .3771 | 1 .80E-10 | 9 .01E-10 |
| ADM | 1658 .88777 | 1 .58339411 1 | 0 .20388 | 7 .76633 | 8 .08E-15 | 6 .74E-14 |
| ADM2 | 30 .47176593 | 1 . 153543103 | 0 .27601 | 4 . 17933 | 2 .92E-05 | 7 .01E-05 |
| ADM5 | 50 .72909352 | 0 .397563918 | 0 . 18318 | 2 . 17031 | 0 .02998 | 0 .04381 |
| ADNP | 1 1066 .45537 | 0 . 160533073 | 0 .07991 | 2 .00902 | 0 .04453 | 0 .06298 |
| ADNP2 | 2937 .084019 | 0 . 103650285 | 0 .06935 | 1 .49457 | 0 . 13503 | 0 . 17294 |
| ADO | 3003 .282003 | 0 .518982738 | 0 .09878 | 5 .25408 | 1 .49E-07 | 5 .02E-07 |
| ADORA1 | 73 .59515292 | 1 .243367567 | 0 .23169 | 5 .36643 | 8 .03E-08 | 2 .83E-07 |
| ADORA2A | 31 .98788522 | 0 .748346889 | 0 . 18935 | 3 .95222 | 7 .74E-05 | 0 .00017 |
| ADORA2B | 649 .990679 | -0 .562839774 | 0 . 13286 | -4 .2364 | 2 .27E-05 | 5 .55E-05 |
| ADPGK | 4544 .497517 | 0 .305762802 | 0 .09041 | 3 .38208 | 0 .00072 | 0 .00139 |
| ADPRH | 919 .576998 | 1 .242823594 | 0 . 10717 | 1 1 .597 | 4 .27E-31 | 2 .09E-29 |
| ADPRHL1 | 91 .75842519 | 0 .304623237 | 0 .22332 | 1 .36404 | 0 . 17256 | 0 .21606 |
| ADPRHL2 | 1935 .516519 | 0 .467576748 | 0 . 10393 | 4 .49894 | 6 .83E-06 | 1 .80E-05 |
| ADPRM | 856 .9549439 | -0 .691327922 | 0 . 10743 | -6 .4353 | 1 .23E-10 | 6 .26E-10 |
| ADRA1A | 89 .58958785 | 1 .232925567 | 0 .44097 | 2 .79596 | 0 .00517 | 0 .00868 |
| ADRA1B | 28 .64820042 | 1 .726952131 | 0 .41492 | 4 . 16209 | 3 . 15E-05 | 7 .52E-05 |

| ADRA1D ADRA2A ADRA2C ADRB1 ADRB2 ADRB3 ADRM1 ADSL ADSS ADSSL1 ADTRP | 13 .5391774 618 .4511809 399 .6630917 47 .53888933 793 .2105895 6 . 158061813 2447 .06896 4557 .016748 6106 .898249 270 .638753 1740 .483272 |
| --- | --- |

AE000662 .924 . 1 16024803

AEBP1 13219 .22801 AEBP2 4331 .30485 AEN 3254 .973927 AES 2833 .08363 AF011889 .5 72 .630894 AF165138 .7 13 . 13485503 AFAP1 2052 .517227 AFAP1L1 1712 .941569 AFAP1L2 3615 .736526 AFF1 10100 .34717 AFF2 475 . 1231883 AFF3 1913 .668943 AFF4 16385 .64453 AFG3L2 5183 . 154672 AFM 10 .58540709 AFMID 957 .472057 AFP 10 .93942151 AFTPH 6241 .300198 AGA 3272 .268017 AGAP1 2233 .781754 AGAP2 289 .5330399 AGAP2-AS1 142 .5835902 AGAP3 2864 .706185 AGAP4 66 .73536297 AGAP5 18 .08059159 AGAP6 335 .0769373 AGAP9 7 .023872735 AGBL1 32 .51279468 AGBL2 88 .9910965 AGBL3 530 . 1543412 AGBL4 88 . 14893518 AGBL5 1484 .342944 AGER 102 .5891122 AGFG1 9143 .870376 AGFG2 955 . 1615195 AGGF1 4539 .858253 AGK 2500 .034795 AGL 4623 .476745 AGMAT 131 .3764703 AGMO 189 .4205443 AGO1 5537 . 166187 AGO2 1965 .57782 AGO3 7072 .570206 AGO4 3831 .853457

1 .046828089

2 . 150292938

1 . 134091532

0 .504090156

-0 .404349874

-0 .864043744

0 .610577335

-0 .267817344

-0 .004877235

-0 . 193345793

-0 .055775994

-1 .33385545

1 .788096987

-0 .09962976

1 .253799683

0 .094836793

-1 .241453725

-1 .643752146

0 .257574377

1 .306170964

-1 .033150562

-0 . 157165653

-0 .678458279

-0 .604573298

0 . 150051988

0 .069205854

-5 .378023938

-0 .299999263

-4 .95194197

-0 .331866198

-0 .322024537

0 .36800258

1 .514090081

0 .275640879

0 .890439848

-0 .560190629

-0 .576298201

0 .077253417

-0 .593421363

-0 .952713913

-1 .01721765

-0 .677374159

-2 . 1 16145093

-0 .244419679

-0 .061303243

-0 . 195089402

-0 .345843104

0 . 1 1363443

-0 . 1 13504034

0 .029440181

1 .886708723

-0 .215198135

0 . 199195079

0 .204093962

-0 .410308406

-0 . 17457918

0 .30617 3 .41908

0 .24555 8 .75689

0 .29583 3 .83365

0 .32117 1 .56956

0 . 19063 -2 . 1212

0 .45403 -1 .9031

0 . 12229 4 .99266

0 . 13937 -1 .9216

0 .09315 -0 .0524

0 . 14448 -1 .3382

0 .32306 -0 . 1726

0 .33206 -4 .0169

0 . 16244 1 1 .0077

0 .09978 -0 .9984

0 . 16761 7 .48051

0 .09585 0 .98946

0 . 19958 -6 .2203

0 .46357 -3 .5459

0 . 1 1294 2 .28066

0 . 15917 8 .20614

0 . 16104 -6 .4155

0 .08533 -1 .8418

0 .23108 -2 .936

0 .20411 -2 .962

0 .07196 2 .08533

0 .08207 0 .84323

0 .60095 -8 .9492

0 . 10153 -2 .9549

0 .73957 -6 .6957

0 .0828 -4 .0079

0 . 13513 -2 .383

0 . 10641 3 .45827

0 . 1734 8 .73189

0 . 16676 1 .65294

0 . 10957 8 . 1264

0 .29853 -1 .8765

0 .21308 -2 .7046

0 . 14526 0 .53185

0 .27012 -2 . 1969

0 .41772 -2 .2808

0 . 17492 -5 .8153

0 . 14594 -4 .6416

0 .26576 -7 .9625

0 .08821 -2 .7709

0 . 14078 -0 .4355

0 .07881 -2 .4754

0 . 14052 -2 .4611

0 .08492 1 .33807

0 . 10799 -1 .0511

0 . 10975 0 .26825

0 . 1751 10 .7753

0 .30887 -0 .6967

0 .09166 2 . 17323

0 .09511 2 . 14593

0 . 10949 -3 .7473

0 .08295 -2 . 1045

0 .00063 2 .01E-18 0 .00013 0 . 1 1652 0 .03391 0 .05703 5 .96E-07 0 .05466 0 .95824 0 . 18082 0 .86293 5 .90E-05 3 .51E-28 0 .31806 7 .40E-14 0 .32244 4 .96E-10 0 .00039 0 .02257 2 .28E-16 1 .40E-10 0 .06551 0 .00332 0 .00306 0 .03704 0 .3991 3 .58E-19 0 .00313 2 . 15E-1 1 6 . 13E-05 0 .01717 0 .00054 2 .50E-18 0 .09834 4 .42E-16 0 .06059 0 .00684 0 .59483 0 .02803 0 .02256 6 .05E-09 3 .46E-06 1 .69E-15 0 .00559 0 .66323 0 .01331 0 .01385 0 . 18087 0 .29323 0 .78851 4 .50E-27 0 .48597 0 .02976 0 .03188 0 .00018 0 .03533

0 .00122 2 .58E-17 0 .00027 0 . 15142 0 .049 0 .07887 1 .84E-06 0 .07588 0 .96488 0 .22547 0 .88451 0 .00013 1 .29E-26 0 .37256 5 .52E-13 0 .37731 2 .34E-09 0 .00079 0 .03376 2 .30E-15 7 .08E-10 0 .08958 0 .00577 0 .00533 0 .05312 0 .45571 4 .99E-18 0 .00545 1 .20E-10 0 .00014 0 .0262 0 .00107 3 . 16E-17 0 . 12969 4 .33E-15 0 .0834 0 .01122 0 .64555 0 .04119 0 .03376 2 .49E-08 9 .48E-06 1 .53E-14 0 .00933 0 .70836 0 .02069 0 .02147 0 .22552 0 .34673 0 .81961 1 .49E-25 0 .54109 0 .04351 0 .04631 0 .00038 0 .05087

| AGPAT1 | 618 . 1401313 | 0 .501106767 | 0 . 13819 | 3 .62629 | 0 .00029 | 0 .00059 |
| --- | --- | --- | --- | --- | --- | --- |
| AGPAT2 | 2790 .289321 | 0 .452979665 | 0 . 13315 | 3 .40201 | 0 .00067 | 0 .0013 |
| AGPAT3 | 5356 .097148 | 0 .958188426 | 0 . 16222 | 5 .90659 | 3 .49E-09 | 1 .47E-08 |
| AGPAT4 | 1018 .675449 | -0 .378346192 | 0 . 13679 | -2 .7659 | 0 .00568 | 0 .00947 |
| AGPAT5 | 3256 .374071 | 0 .422333407 | 0 . 14364 | 2 .94024 | 0 .00328 | 0 .0057 |
| AGPS | 7949 .281349 | 0 .059251147 | 0 . 1 1558 | 0 .51264 | 0 .6082 | 0 .65786 |
| AGR2 | 176 .0908205 | -0 .589427447 | 0 .38035 | -1 .5497 | 0 . 12122 | 0 . 15684 |
| AGR3 | 20 .95807977 | -4 .390924073 | 0 .55898 | -7 .8553 | 3 .99E-15 | 3 .47E-14 |
| AGRN | 6077 . 160686 | 1 .54552643 | 0 . 13053 | 1 1 .84 | 2 .43E-32 | 1 .36E-30 |
| AGRP | 20 .78898767 | -0 . 159495189 | 0 . 18967 | -0 .8409 | 0 .4004 | 0 .45697 |
| AGT | 132 .3990597 | 3 .369541826 | 0 .40156 | 8 .39112 | 4 .82E-17 | 5 .24E-16 |
| AGTPBP1 | 4305 .977629 | -0 .311 106062 | 0 .09467 | -3 .2863 | 0 .00102 | 0 .00191 |
| AGTR1 | 345 . 1084952 | 0 .431811432 | 0 .29151 | 1 .48128 | 0 . 13853 | 0 . 17706 |
| AGTR2 | 8 .309828431 | -5 . 161841656 | 0 .81339 | -6 .3461 | 2 .21E-10 | 1 .09E-09 |
| AGTRAP | 1707 . 137063 | 0 .901533491 | 0 . 10292 | 8 .75982 | 1 .96E-18 | 2 .52E-17 |
| AGXT | 18 .39328436 | -0 .534541783 | 0 .37826 | -1 .4132 | 0 . 15761 | 0 . 19896 |
| AGXT2 | 13 .26816772 | -4 .868790172 | 0 .61041 | -7 .9763 | 1 .51E-15 | 1 .37E-14 |
| AHCTF1 | 8081 .314919 | 0 . 185185491 | 0 .08188 | 2 .26172 | 0 .02371 | 0 .03535 |
| AHCY | 4591 . 193594 | 0 .292289002 | 0 . 148 | 1 .97492 | 0 .04828 | 0 .06777 |
| AHCYL1 | 5322 .666879 | 0 .255182264 | 0 .09101 | 2 .80393 | 0 .00505 | 0 .00849 |
| AHCYL2 | 17049 .69777 | -2 .259976202 | 0 .20908 | -10 .809 | 3 . 1 1E-27 | 1 .04E-25 |
| AHDC1 | 875 .0693115 | 0 .501177262 | 0 . 1952 | 2 .56752 | 0 .01024 | 0 .01625 |
| AHI1 | 3012 .211907 | -0 .653731003 | 0 . 13121 | -4 .9822 | 6 .29E-07 | 1 .93E-06 |
| AHNAK | 372727 .2019 | -0 .577716511 | 0 . 1547 | -3 .7344 | 0 .00019 | 0 .0004 |
| AHNAK2 | 52373 .05241 | -1 .0418242 | 0 .21083 | -4 .9415 | 7 .75E-07 | 2 .35E-06 |
| AHR | 31994 .30568 | 0 .378586556 | 0 . 1 1918 | 3 . 17659 | 0 .00149 | 0 .00273 |
| AHRR | 880 .7185402 | 0 . 170441543 | 0 .21073 | 0 .80881 | 0 .41863 | 0 .47529 |
| AHSA1 | 3061 .346319 | 0 .006955418 | 0 . 12848 | 0 .05414 | 0 .95683 | 0 .96365 |
| AHSA2 | 2593 .369034 | -0 .598563521 | 0 . 1289 | -4 .6436 | 3 .42E-06 | 9 .40E-06 |
| AHSG | 6 . 142445354 | -2 . 198754022 | 0 .48686 | -4 .5162 | 6 .30E-06 | 1 .67E-05 |
| AHSP | 7 .972855269 | 0 .452182419 | 0 .57216 | 0 .79031 | 0 .42935 | 0 .48586 |
| AICDA | 21 .75849823 | -0 .276597058 | 0 .55964 | -0 .4942 | 0 .62113 | 0 .67018 |
| AIDA | 3343 .080402 | -0 .060484819 | 0 .07951 | -0 .7607 | 0 .44684 | 0 .50264 |
| AIF1 | 1028 .942944 | 1 .370821438 | 0 . 19881 | 6 .89527 | 5 .38E-12 | 3 .25E-1 1 |
| AIF1L | 6299 .599765 | 0 . 187427345 | 0 . 19371 | 0 .96757 | 0 .33326 | 0 .38825 |
| AIFM1 | 1586 .526558 | 0 .853395259 | 0 .09863 | 8 .65218 | 5 .05E-18 | 6 . 16E-17 |
| AIFM2 | 1006 .450337 | 0 .449602417 | 0 . 10848 | 4 . 1446 | 3 .40E-05 | 8 .08E-05 |
| AIFM3 | 78 .89753633 | -0 .499620016 | 0 . 1385 | -3 .6074 | 0 .00031 | 0 .00063 |
| AIG1 | 2194 .648639 | -1 .336958014 | 0 . 10988 | -12 . 167 | 4 .65E-34 | 3 .08E-32 |
| AIM1 | 10650 .45132 | -0 .017152764 | 0 . 18187 | -0 .0943 | 0 .92486 | 0 .93732 |
| AIM1L | 850 .5084854 | -0 .422605008 | 0 .25226 | -1 .6753 | 0 .09388 | 0 . 12444 |
| AIM2 | 384 .7922218 | 3 .507756562 | 0 .35194 | 9 .96697 | 2 . 13E-23 | 4 .67E-22 |
| AIMP1 | 3477 .497049 | -0 .286996414 | 0 .09532 | -3 .0109 | 0 .00261 | 0 .00459 |
| AIMP2 | 941 .5173543 | 0 .359051675 | 0 . 1307 | 2 .74709 | 0 .00601 | 0 .00998 |
| AIP | 3007 .43332 | -0 .464783958 | 0 . 15537 | -2 .9915 | 0 .00278 | 0 .00487 |
| AIPL1 | 6 .706444289 | -4 . 147173951 | 0 .65363 | -6 .3449 | 2 .23E-10 | 1 . 10E-09 |
| AIRE | 20 .33635558 | -1 .234932775 | 0 .21963 | -5 .6229 | 1 .88E-08 | 7 .22E-08 |
| AJAP1 | 260 .4078222 | 0 .503848126 | 0 .25478 | 1 .97761 | 0 .04797 | 0 .06739 |
| AJUBA | 2900 .248481 | -0 .631631949 | 0 . 15795 | -3 .999 | 6 .36E-05 | 0 .00014 |
| AK1 | 175 .9053387 | 0 .993172517 | 0 . 10149 | 9 .78553 | 1 .30E-22 | 2 .62E-21 |
| AK2 | 9650 .015894 | 0 .380172675 | 0 . 10317 | 3 .6849 | 0 .00023 | 0 .00048 |
| AK3 | 6766 .367495 | -0 .326802815 | 0 . 1 1689 | -2 .7958 | 0 .00518 | 0 .00869 |
| AK4 | 2512 .9148 | -0 .627584584 | 0 . 17042 | -3 .6826 | 0 .00023 | 0 .00048 |
| AK5 | 557 .9326141 | 0 .392624169 | 0 .24002 | 1 .63583 | 0 . 10188 | 0 . 13395 |
| AK6 | 3957 .338868 | -0 .357643226 | 0 . 12254 | -2 .9186 | 0 .00352 | 0 .00608 |
| AK7 | 212 .9789352 | -1 .680533081 | 0 .22304 | -7 .5348 | 4 .89E-14 | 3 .75E-13 |

| AK8 1 1 1 . 1474035 | -0 .534020577 | 0 . 16861 | -3 . 1672 | 0 .00154 | 0 .00281 |
| --- | --- | --- | --- | --- | --- |
| AK9 607 .2942279 | -1 .047483737 | 0 . 12632 | -8 .2924 | 1 . 1 1E-16 | 1 . 17E-15 |
| AKAIN1 1 . 141532088 | -3 .822010175 | 0 .94585 | -4 .0408 | 5 .33E-05 | 0 .00012 |
| AKAP1 1430 .602683 | 0 .213144689 | 0 . 1 1263 | 1 .89248 | 0 .05843 | 0 .08065 |
| AKAP10 2966 .422818 | 0 .038700378 | 0 .04914 | 0 .78759 | 0 .43094 | 0 .48737 |
| AKAP11 10540 .64603 | 0 .065902193 | 0 .08966 | 0 .73498 | 0 .46235 | 0 .51812 |
| AKAP12 7748 .454053 | -0 .537581317 | 0 . 19505 | -2 .7561 | 0 .00585 | 0 .00974 |
| AKAP13 17610 .32993 | -0 . 172776307 | 0 .09209 | -1 .8762 | 0 .06062 | 0 .08343 |
| AKAP14 12 .5467452 | -1 .233102201 | 0 .25632 | -4 .8107 | 1 .50E-06 | 4 .35E-06 |
| AKAP17A 1875 .219866 | 0 .758311436 | 0 .0751 | 10 .0976 | 5 .66E-24 | 1 .33E-22 |
| AKAP2 128 . 1475381 | 0 . 172434214 | 0 . 1 1364 | 1 .5174 | 0 . 12917 | 0 . 16617 |
| AKAP3 93 . 13954244 | -1 .779199835 | 0 . 16831 | -10 .571 | 4 .05E-26 | 1 .20E-24 |
| AKAP4 7 .805864754 | -6 .020909654 | 0 .76394 | -7 .8813 | 3 .24E-15 | 2 .85E-14 |
| AKAP5 887 .4547409 | 0 .523509205 | 0 . 19091 | 2 .74212 | 0 .0061 | 0 .01012 |
| AKAP6 2409 .829413 | 0 .382054285 | 0 .26455 | 1 .44417 | 0 . 14869 | 0 . 18869 |
| AKAP7 736 .3730782 | -0 .288738348 | 0 . 12024 | -2 .4013 | 0 .01634 | 0 .02501 |
| AKAP8 2034 . 12201 | 0 .303623394 | 0 .06466 | 4 .69592 | 2 .65E-06 | 7 .41E-06 |
| AKAP8L 1514 .416781 | 0 .327277783 | 0 .08685 | 3 .76839 | 0 .00016 | 0 .00035 |
| AKAP9 22765 .55268 | -0 .481581741 | 0 . 15304 | -3 . 1468 | 0 .00165 | 0 .003 |
| AKIP1 1 139 .941789 | 0 .71797353 | 0 . 1 151 | 6 .2379 | 4 .43E-10 | 2 . 10E-09 |
| AKIRIN1 3065 .50581 | 0 .959688129 | 0 . 1 1092 | 8 .65194 | 5 .06E-18 | 6 . 17E-17 |
| AKIRIN2 1312 .468636 | 0 .442399954 | 0 .07816 | 5 .66009 | 1 .51E-08 | 5 .89E-08 |
| AKNA 2468 .443402 | 1 .350120932 | 0 . 16169 | 8 .35028 | 6 .81E-17 | 7 .31E-16 |
| AKNAD1 1 16 .7453938 | -2 .015911766 | 0 . 18171 | -1 1 .094 | 1 .34E-28 | 5 . 12E-27 |
| AKR1A1 4869 .064721 | 0 .31344576 | 0 . 1 1616 | 2 .69842 | 0 .00697 | 0 .01142 |
| AKR1B1 3713 .377714 | 0 .411895139 | 0 . 17087 | 2 .41058 | 0 .01593 | 0 .02441 |
| AKR1B10 870 .2282699 | 2 .413812353 | 0 .29952 | 8 .05901 | 7 .69E-16 | 7 .21E-15 |
| AKR1B15 123 .3607573 | 1 .628148047 | 0 .2736 | 5 .9508 | 2 .67E-09 | 1 . 15E-08 |
| AKR1C1 2230 .905661 | -1 .366453237 | 0 .24019 | -5 .6891 | 1 .28E-08 | 5 .02E-08 |
| AKR1C2 1319 .498878 | -0 . 15618655 | 0 .2506 | -0 .6233 | 0 .53312 | 0 .58677 |
| AKR1C3 1298 .211 105 | -0 .441883301 | 0 .23257 | -1 .9 | 0 .05743 | 0 .07937 |
| AKR1C4 18 .08286518 | -2 .236680933 | 0 .32256 | -6 .9342 | 4 .08E-12 | 2 .50E-1 1 |
| AKR1D1 12 .61495321 | -1 .910565508 | 0 .54527 | -3 .5039 | 0 .00046 | 0 .00091 |
| AKR1E2 580 .6074049 | -1 .606201711 | 0 .21461 | -7 .4841 | 7 .20E-14 | 5 .38E-13 |
| AKR7A2 2674 .871548 | 0 . 197338821 | 0 . 10828 | 1 .82254 | 0 .06837 | 0 .09313 |
| AKR7A3 85 .43288707 | 0 .632848914 | 0 .21764 | 2 .9078 | 0 .00364 | 0 .00627 |
| AKT1 5677 .601122 | 0 .623976393 | 0 . 10159 | 6 . 14215 | 8 . 14E-10 | 3 .75E-09 |
| AKT1S1 597 .0424934 | 0 .489321264 | 0 .09587 | 5 . 10413 | 3 .32E-07 | 1 .07E-06 |
| AKT2 5531 .407411 | 0 . 145304385 | 0 .07049 | 2 .06131 | 0 .03927 | 0 .05608 |
| AKT3 9364 .868969 | -0 .580507657 | 0 . 1 1671 | -4 .9738 | 6 .56E-07 | 2 .01E-06 |
| AKTIP 2052 .589507 | -0 .675041504 | 0 .0798 | -8 .4588 | 2 .70E-17 | 3 .03E-16 |
| AL356053 . 1 421 .3035101 | -1 .491585994 | 0 . 1639 | -9 . 1006 | 8 .98E-20 | 1 .35E-18 |
| AL928654 .7 22 .00696323 | -0 .358378211 | 0 .29442 | -1 .2172 | 0 .22352 | 0 .27224 |
| ALAD 2402 .681215 | -0 . 189081732 | 0 .09556 | -1 .9788 | 0 .04784 | 0 .06724 |
| ALAS1 2718 .357802 | 0 .708786672 | 0 . 1 1033 | 6 .42426 | 1 .33E-10 | 6 .71E-10 |
| ALAS2 22 .67281177 | -1 .381218452 | 0 .27476 | -5 .027 | 4 .98E-07 | 1 .55E-06 |
| ALB 28 .28278214 | -1 .393775147 | 0 .38384 | -3 .6311 | 0 .00028 | 0 .00058 |
| ALCAM 6638 . 148078 | 0 .694492981 | 0 . 16976 | 4 .09094 | 4 .30E-05 | 0 .0001 |
| ALDH16A1 1791 .703085 | 1 .36188294 | 0 .08437 | 16 . 1418 | 1 .30E-58 | 1 .04E-55 |
| ALDH18A1 5143 .288999 | 0 .088398042 | 0 .08553 | 1 .03351 | 0 .30137 | 0 .35506 |
| ALDH1A1 5818 .281323 | -0 .630484398 | 0 .2211 1 | -2 .8515 | 0 .00435 | 0 .00739 |
| ALDH1A2 323 .8954792 | -0 . 131881397 | 0 .22504 | -0 .586 | 0 .55786 | 0 .61032 |
| ALDH1A3 3465 .220453 | 1 . 174846963 | 0 .29367 | 4 .00051 | 6 .32E-05 | 0 .00014 |
| ALDH1B1 1309 .353328 | 1 .536898744 | 0 . 17177 | 8 .94723 | 3 .65E-19 | 5 .07E-18 |
| ALDH1L1 224 .6694758 | 0 .715790747 | 0 .37441 | 1 .9118 | 0 .0559 | 0 .07743 |
| ALDH1L2 2703 .877095 | -0 .834502406 | 0 . 19613 | -4 .2548 | 2 .09E-05 | 5 . 14E-05 |

| ALDH2 ALDH3A1 ALDH3A2 ALDH3B1 ALDH3B2 ALDH4A1 ALDH5A1 ALDH6A1 ALDH7A1 ALDH8A1 ALDH9A1 ALDOA ALDOB ALDOC ALG1 ALG10 ALG10B ALG11 ALG12 ALG13 ALG14 ALG1L ALG2 ALG3 ALG5 ALG6 ALG8 ALG9 ALK ALKBH1 ALKBH2 ALKBH3 ALKBH4 ALKBH5 ALKBH6 ALKBH7 ALKBH8 ALLC ALMS1 ALOX12 | 3457 .408555 944 .2914229 15237 .57027 730 .8526343 2882 .961882 747 .9446996 1292 .806461 3004 .799405 3043 .690409 62 .03748525 8323 .298018 15233 .7049 43 .87386203 1366 .73214 1053 .565276 583 .7156212 847 .8941332 868 .6599483 725 .285392 2858 .697641 1516 . 123392 54 .84024788 2062 .517522 800 .7887617 1409 .73966 2551 .969538 2295 .572464 710 .6691313 338 .8907115 909 .9805544 1297 .009224 1878 .250183 549 .2069217 3798 .883868 69 .39940052 2752 .271406 1298 .083006 10 .42613921 7119 .354963 249 .4953754 |
| --- | --- |

ALOX12-AS1229 .8565514

| ALOX12B ALOX15 ALOX15B ALOX5 ALOX5AP ALOXE3 ALPI ALPK1 ALPK2 ALPK3 ALPL ALPP ALPPL2 ALS2 ALS2CL | 660 .0485367 313 .5901209 2611 .937597 911 .4923896 713 .352501 622 .7808214 2 .945926671 3179 . 12083 496 . 1776104 204 .8446851 157 .6946872 3 .05841758 2 .089443024 3711 .936956 3322 .349321 |
| --- | --- |

-0 .229295501

-1 .622248423

-1 .379505542

0 .519037764

0 . 180809731

0 .922781691

0 .002474171

0 . 157569539

-0 .407231449

-0 .673772497

-0 . 154618164

0 .807309309

-1 .857428171

-1 .205012307

0 .378970514

-0 . 19561813

-0 .413168087

-0 .245115388

0 .271464479

-0 .456807282

-0 .403304894

-1 .054036598

0 .457818285

0 .233343498

-0 .044496967

-0 .392957687

0 .272879083

-0 .401837421

-0 .971634691

-0 . 185507882

-0 .560136382

-0 .478285016

0 .388488183

0 .677921873

0 . 132938166

-0 . 101683634

-0 .613812358

-2 .791310405

-0 .461999777

-0 .917941683

-0 .290382723

-0 .088395312

0 .915362123

2 .326754358

1 . 101266775

1 .009418372

-1 . 195539507

-2 .251123522

-0 .275968692

-0 .521631912

1 .664516031

1 .788643334

-2 .878137015

-2 .812620028

-0 .364331415

-0 .958767613

0 . 18924 -1 .2116

0 .28913 -5 .6108

0 . 16006 -8 .6185

0 . 12709 4 .08407

0 .29412 0 .61475

0 . 12727 7 .25077

0 . 15835 0 .01562

0 . 10457 1 .50684

0 .08535 -4 .7713

0 .20708 -3 .2536

0 . 1 1 1 13 -1 .3913

0 . 15384 5 .2478

0 .24332 -7 .6336

0 . 14221 -8 .4737

0 .09112 4 . 15903

0 .09296 -2 . 1044

0 .09551 -4 .3257

0 .07735 -3 . 1691

0 .06799 3 .99299

0 .07014 -6 .5128

0 . 1 1533 -3 .4969

0 .25353 -4 . 1575

0 .09019 5 .07624

0 . 1 1404 2 .04614

0 . 12004 -0 .3707

0 . 1027 -3 .8262

0 . 15784 1 .72887

0 .09635 -4 . 1706

0 . 17548 -5 .5371

0 .07691 -2 .412

0 . 13819 -4 .0534

0 .08424 -5 .6775

0 . 1 1329 3 .42925

0 .07823 8 .66576

0 . 10866 1 .22338

0 . 17039 -0 .5968

0 .06978 -8 .7962

0 .45282 -6 . 1643

0 .08313 -5 .5572

0 . 14498 -6 .3315

0 . 12085 -2 .4028

0 .29987 -0 .2948

0 .4767 1 .92019

0 .58147 4 .0015

0 . 13497 8 . 1596

0 . 19735 5 . 1 1481

0 .27975 -4 .2736

0 .73873 -3 .0473

0 .09489 -2 .9082

0 .31007 -1 .6823

0 .2058 8 .08819

0 . 19934 8 .97292

0 .70053 -4 . 1085

0 .73182 -3 .8433

0 .07119 -5 . 1 179

0 . 19842 -4 .8319

0 .22565 2 .01E-08 6 .78E-18 4 .43E-05 0 .53872 4 . 14E-13 0 .98753 0 . 13185 1 .83E-06 0 .00114 0 . 16414 1 .54E-07 2 .28E-14 2 .38E-17 3 .20E-05 0 .03535 1 .52E-05 0 .00153 6 .52E-05 7 .38E-1 1 0 .00047 3 .22E-05 3 .85E-07 0 .04074 0 .71088 0 .00013 0 .08383 3 .04E-05 3 .07E-08 0 .01586 5 .05E-05 1 .37E-08 0 .00061 4 .48E-18 0 .22119 0 .55066 1 .41E-18 7 .08E-10 2 .74E-08 2 .43E-10 0 .01627 0 .76816 0 .05483 6 .29E-05 3 .36E-16 3 . 14E-07 1 .92E-05 0 .00231 0 .00364 0 .09251 6 .06E-16 2 .89E-19 3 .98E-05 0 .00012 3 .09E-07 1 .35E-06

0 .27452 7 .71E-08 8 . 13E-17 0 .0001 0 .59202 2 .85E-12 0 .98944 0 . 16926 5 .23E-06 0 .00213 0 .20652 5 . 18E-07 1 .82E-13 2 .69E-16 7 .62E-05 0 .05089 3 .81E-05 0 .0028 0 .00015 3 .85E-10 0 .00093 7 .67E-05 1 .22E-06 0 .05801 0 .7518 0 .00028 0 . 1 1228 7 .27E-05 1 . 15E-07 0 .02432 0 .00012 5 .36E-08 0 .00118 5 .51E-17 0 .26957 0 .60327 1 .84E-17 3 .28E-09 1 .03E-07 1 . 19E-09 0 .02491 0 .80133 0 .07611 0 .00014 3 .33E-15 1 .01E-06 4 .76E-05 0 .00411 0 .00627 0 . 12284 5 .80E-15 4 .08E-18 9 .35E-05 0 .00026 9 .97E-07 3 .94E-06

| ALS2CR11 934 .7712548 | -1 .560173005 | 0 .24344 -6 .4088 | 1 .47E-10 | 7 .39E-10 |
| --- | --- | --- | --- | --- |
| ALS2CR12 180 .016865 | -0 .615159667 | 0 . 18262 -3 .3685 | 0 .00076 | 0 .00145 |
| ALX1 1426 .466516 | -1 .480892308 | 0 .24562 -6 .0291 | 1 .65E-09 | 7 .27E-09 |
| ALX3 3 .906400057 | 0 .632903778 | 0 .59775 1 .05881 | 0 .28969 | 0 .34292 |
| ALX4 294 .9820335 | -0 .412058023 | 0 .272 -1 .5149 | 0 . 1298 | 0 . 1669 |
| ALYREF 1901 .550843 | 0 .649621699 | 0 . 10841 5 .99233 | 2 .07E-09 | 9 .02E-09 |
| AMACR 228 .6597922 | 0 .267961709 | 0 . 10653 2 .51541 | 0 .01189 | 0 .01865 |
| AMBN 9 .073074694 | -2 .83327511 | 0 .51656 -5 .4849 | 4 . 14E-08 | 1 .51E-07 |
| AMBP 19 .08280331 | -1 .059788744 | 0 .29544 -3 .5872 | 0 .00033 | 0 .00068 |
| AMBRA1 3456 .273174 | -0 . 175958781 | 0 .08005 -2 . 1981 | 0 .02794 | 0 .04107 |
| AMD1 14509 .43401 | -1 .286898836 | 0 .08859 -14 .527 | 8 .21E-48 | 2 .07E-45 |
| AMDHD1 141 .5784181 | -0 .416609432 | 0 .21519 -1 .936 | 0 .05286 | 0 .07361 |
| AMDHD2 992 .6675448 | 0 .816547093 | 0 . 12203 6 .6912 | 2 .21E-1 1 | 1 .24E-10 |
| AMELX 14 .08094969 | -0 .355726464 | 0 .29939 -1 . 1882 | 0 .23477 | 0 .2844 |
| AMELY 0 . 14688501 | 0 .31231288 | 2 .3469 0 . 13307 | 0 .89413 | 0 .91157 |
| AMER1 942 .2578879 | -0 . 192504203 | 0 . 1 1556 -1 .6658 | 0 .09574 | 0 . 12664 |
| AMER2 222 .4685851 | 1 .703590592 | 0 .49809 3 .42028 | 0 .00063 | 0 .00122 |
| AMER3 7 .472749687 | -3 .445918214 | 0 .76852 -4 .4839 | 7 .33E-06 | 1 .92E-05 |
| AMFR 10297 .37007 | 0 .051587168 | 0 .09629 0 .53577 | 0 .59212 | 0 .64296 |
| AMH 26 .60781263 | 0 .444987606 | 0 .25727 1 .72962 | 0 .0837 | 0 . 1 1211 |
| AMHR2 13 .84492522 | -1 .383040336 | 0 .39657 -3 .4875 | 0 .00049 | 0 .00096 |
| AMIGO1 430 .8334901 | -0 .516050717 | 0 . 16955 -3 .0436 | 0 .00234 | 0 .00415 |
| AMIGO2 489 .2410524 | 1 .373028131 | 0 . 18713 7 .33723 | 2 . 18E-13 | 1 .55E-12 |
| AMIGO3 0 .496788669 | -0 .744641873 | 0 .72632 -1 .0252 | 0 .30526 | 0 .35934 |
| AMMECR1 2488 .618522 | -0 . 163689634 | 0 .09675 -1 .6919 | 0 .09066 | 0 . 12066 |
| AMMECR1L 3332 .071693 | 0 .585433886 | 0 .09125 6 .41544 | 1 .40E-10 | 7 .08E-10 |
| AMN 101 .4506959 | -0 .450394911 | 0 . 19466 -2 .3138 | 0 .02068 | 0 .03116 |
| AMN1 552 .9095909 | -0 .934692813 | 0 . 13866 -6 .7411 | 1 .57E-1 1 | 8 .95E-1 1 |
| AMOT 1954 .091908 | -1 .090011219 | 0 . 16854 -6 .4674 | 9 .97E-1 1 | 5 . 12E-10 |
| AMOTL1 1 1613 .04574 | -0 .389726452 | 0 . 1025 -3 .802 | 0 .00014 | 0 .00031 |
| AMOTL2 4699 . 10965 | -0 . 161440533 | 0 . 14292 -1 . 1296 | 0 .25865 | 0 .30998 |
| AMPD1 16 .50159034 | -1 .384619865 | 0 .50658 -2 .7333 | 0 .00627 | 0 .01037 |
| AMPD2 1616 .494299 | 0 .311496972 | 0 . 10272 3 .03261 | 0 .00242 | 0 .0043 |
| AMPD3 1079 . 147685 | 0 .080066134 | 0 . 10816 0 .74027 | 0 .45913 | 0 .51494 |
| AMPH 270 .5009518 | 0 .961085136 | 0 .27222 3 .53049 | 0 .00041 | 0 .00083 |
| AMT 583 .3268535 | -0 .778171988 | 0 . 15777 -4 .9324 | 8 . 12E-07 | 2 .46E-06 |
| AMTN 13 .83341891 | -0 .533732954 | 0 .54959 -0 .9711 | 0 .33148 | 0 .38648 |
| AMY1A 0 .504373828 | -2 .946294302 | 1 .71575 -1 .7172 | 0 .08594 | 0 . 1 1485 |
| AMY1B 1 . 16121602 | -0 .922545088 | 0 .55503 -1 .6622 | 0 .09648 | 0 . 12752 |
| AMY1C 0 .492645252 | -2 .926801832 | 1 .4524 -2 .0152 | 0 .04389 | 0 .06213 |
| AMY2A 18 .02839645 | -2 .367356036 | 0 .26413 -8 .9627 | 3 . 17E-19 | 4 .44E-18 |
| AMY2B 1674 .698981 | -1 .871519918 | 0 . 17378 -10 .769 | 4 .80E-27 | 1 .58E-25 |
| AMZ1 137 .9300602 | 0 .899337913 | 0 .20479 4 .39149 | 1 . 13E-05 | 2 .87E-05 |
| AMZ2 3006 .468011 | -0 .064635691 | 0 . 12509 -0 .5167 | 0 .60535 | 0 .65522 |
| ANAPC1 4128 .585008 | -0 . 102401107 | 0 .05915 -1 .7313 | 0 .08339 | 0 . 1 1 176 |
| ANAPC10 1004 .32931 | -0 .506370603 | 0 .095 -5 .3304 | 9 .80E-08 | 3 .41E-07 |
| ANAPC11 3396 .677317 | 0 . 130786078 | 0 . 1411 0 .92694 | 0 .35396 | 0 .40951 |
| ANAPC13 3078 .06834 | -0 .365106582 | 0 . 10276 -3 .553 | 0 .00038 | 0 .00077 |
| ANAPC15 218 .7555031 | -0 .368703792 | 0 . 1292 -2 .8537 | 0 .00432 | 0 .00735 |
| ANAPC16 5882 .591144 | -0 .71585349 | 0 . 1069 -6 .6962 | 2 . 14E-1 1 | 1 .20E-10 |
| ANAPC2 1313 .976168 | 0 .401881479 | 0 .07763 5 . 17682 | 2 .26E-07 | 7 .42E-07 |
| ANAPC4 4041 .286739 | -0 .548699833 | 0 .09766 -5 .6184 | 1 .93E-08 | 7 .40E-08 |
| ANAPC5 14132 . 10774 | -0 . 135005535 | 0 .07659 -1 .7627 | 0 .07795 | 0 . 10505 |
| ANAPC7 2645 . 194809 | 0 .086868391 | 0 .06 1 .4477 | 0 . 1477 | 0 . 18756 |
| ANG 220 . 1685252 | -1 .570646885 | 0 . 12378 -12 .689 | 6 .77E-37 | 6 .01E-35 |
| ANGEL1 1617 .841183 | 0 .60641289 | 0 .09521 6 .36899 | 1 .90E-10 | 9 .47E-10 |

| ANGEL2 ANGPT1 ANGPT2 ANGPT4 ANGPTL1 ANGPTL2 ANGPTL3 ANGPTL4 ANGPTL5 ANGPTL6 ANGPTL7 ANGPTL8 ANHX ANK1 ANK2 ANK3 ANKAR ANKDD1A ANKDD1B ANKEF1 ANKFN1 ANKFY1 ANKH ANKHD1 | 4716 .638826 896 .6203705 2267 .205836 33 .39043349 1913 .417334 3100 .386602 552 .3248373 793 .4163912 253 .0293272 35 . 15897284 6633 .204356 23 .79922093 4 .381632526 135 .7153547 17784 .20065 4451 .83472 1248 .849494 707 .4006532 161 .0883871 439 .998471 343 .5712426 7706 .862885 5099 .96139 620 .2473318 |
| --- | --- |

ANKHD1-EIF350 .9913078

| ANKIB1 | 7333 .4668 |
| --- | --- |
| ANKK1 | 89 .83388314 |
| ANKLE1 | 33 . 18722217 |
| ANKLE2 | 6231 .666133 |
| ANKMY1 | 739 . 1209334 |
| ANKMY2 | 2385 .801051 |
| ANKRA2 | 1482 .894095 |
| ANKRD1 | 20 . 1013064 |
| ANKRD10 | 1 1246 . 18725 |
| ANKRD11 | 3679 . 100746 |
| ANKRD12 | 10565 .62027 |
| ANKRD13A | 7326 .902236 |
| ANKRD13B | 172 .2424616 |
| ANKRD13C | 4610 .739021 |
| ANKRD13D | 1675 .627291 |
| ANKRD16 | 446 .4490854 |
| ANKRD17 | 14449 .64279 |
| ANKRD18A | 191 .9911281 |
| ANKRD18B | 218 .5768842 |
| ANKRD2 | 28 .611 10004 |

ANKRD20A2 10 .76525558 ANKRD20A4 167 .3926724

| ANKRD22 ANKRD23 ANKRD24 ANKRD26 ANKRD27 ANKRD28 ANKRD29 ANKRD30A ANKRD30B | 1389 .603475 75 .63964676 32 .51470649 2499 . 12581 4421 .788983 25927 .66051 470 .4425659 30 .89646516 135 . 1007007 |
| --- | --- |

0 .054112792

2 . 124981823

-0 .263195769

0 .216373263

-0 .67127021

1 . 1 12163851

-1 .216257133

1 . 185966196

-0 .854715906

0 .511914868

-2 .664506717

0 .286245905

-3 .24712383

1 . 155343101

-1 . 138121561

-1 .078607809

-1 .535380225

0 .529339616

-1 . 17236561

-0 .692562961

-1 .851980049

-0 .232008829

0 . 187685694

-0 .575096433

-0 .661882063

-0 .057104115

-0 .773494738

0 .467902256

0 . 121787622

-0 .010690479

-0 .470690022

-0 .586345963

-1 .571854268

-0 .052116783

0 .58868149

-0 .431336724

-0 .339474229

0 .400596326

-0 .460911015

0 .662222724

-0 .258746641

0 .027069376

-1 .761648931

-1 .36711 1 157

-1 .267143991

-1 .004458238

-1 .828954867

-0 .6428882

-0 .735504726

-0 .322399037

-0 .699542461

-0 .309636649

-0 .252954984

1 .05411 1838

-2 .268842099

1 .340271224

0 .08913 0 .60713

0 .22265 9 .5439

0 .20509 -1 .2833

0 .29612 0 .73068

0 .23947 -2 .8031

0 . 15788 7 .04453

0 . 18529 -6 .5641

0 .23375 5 .07371

0 .35924 -2 .3792

0 . 17982 2 .84675

0 .39462 -6 .7521

0 .25613 1 . 1 1759

0 .71754 -4 .5254

0 .20953 5 .51388

0 . 14422 -7 .8916

0 .24898 -4 .3321

0 . 13983 -10 .98

0 . 12656 4 . 18266

0 . 17973 -6 .5228

0 . 16745 -4 . 136

0 .25203 -7 .3484

0 .08854 -2 .6203

0 . 13384 1 .40232

0 . 15265 -3 .7675

0 . 15899 -4 . 1631

0 .07635 -0 .748

0 .2772 -2 .7903

0 . 19217 2 .43488

0 .05838 2 .08608

0 .09896 -0 . 108

0 . 12147 -3 .8749

0 .07444 -7 .8768

0 .28629 -5 .4904

0 . 14476 -0 .36

0 . 13582 4 .33435

0 . 10859 -3 .9722

0 .06037 -5 .6235

0 . 15504 2 .58382

0 .08289 -5 .5605

0 .07031 9 .41877

0 .08538 -3 .0306

0 .06926 0 .39081

0 .37355 -4 .716

0 .26053 -5 .2475

0 .22932 -5 .5257

0 .37612 -2 .6705

0 .33312 -5 .4904

0 .22013 -2 .9206

0 . 15512 -4 .7416

0 .21703 -1 .4855

0 . 1 1825 -5 .9156

0 .07323 -4 .2283

0 . 16403 -1 .5421

0 . 19827 5 .31649

0 .64959 -3 .4928

0 .48694 2 .75242

0 .54377 1 .38E-21 0 . 19938 0 .46497 0 .00506 1 .86E-12 5 .23E-1 1 3 .90E-07 0 .01735 0 .00442 1 .46E-1 1 0 .26374 6 .03E-06 3 .51E-08 2 .98E-15 1 .48E-05 4 .75E-28 2 .88E-05 6 .90E-1 1 3 .53E-05 2 .01E-13 0 .00879 0 . 16082 0 .00016 3 . 14E-05 0 .45448 0 .00527 0 .0149 0 .03697 0 .91397 0 .00011 3 .36E-15 4 .01E-08 0 .71882 1 .46E-05 7 . 12E-05 1 .87E-08 0 .00977 2 .69E-08 4 .56E-21 0 .00244 0 .69593 2 .41E-06 1 .54E-07 3 .28E-08 0 .00757 4 .01E-08 0 .00349 2 . 12E-06 0 . 13742 3 .31E-09 2 .35E-05 0 . 12305 1 .06E-07 0 .00048 0 .00592

0 .59681 2 .49E-20 0 .24603 0 .52076 0 .00851 1 .20E-1 1 2 .80E-10 1 .24E-06 0 .02645 0 .0075 8 .32E-1 1 0 .31538 1 .60E-05 1 .30E-07 2 .64E-14 3 .71E-05 1 .72E-26 6 .92E-05 3 .62E-10 8 .36E-05 1 .43E-12 0 .01413 0 .20266 0 .00035 7 .50E-05 0 .51035 0 .00883 0 .02296 0 .05304 0 .92854 0 .00023 2 .95E-14 1 .47E-07 0 .75873 3 .68E-05 0 .00016 7 .20E-08 0 .01557 1 .01E-07 7 .81E-20 0 .00433 0 .73798 6 .75E-06 5 . 19E-07 1 .22E-07 0 .01233 1 .47E-07 0 .00604 5 .99E-06 0 . 17574 1 .40E-08 5 .74E-05 0 . 15901 3 .66E-07 0 .00095 0 .00984

| ANKRD30BL 4394 .640811 | 2 .72124208 | 0 .24368 1 1 . 1672 | 5 .90E-29 | 2 .35E-27 |
| --- | --- | --- | --- | --- |
| ANKRD31 190 .5038252 | -1 . 1 14993039 | 0 . 18609 -5 .9918 | 2 .08E-09 | 9 .05E-09 |
| ANKRD33 4 .88522732 | -2 .447606755 | 0 .64926 -3 .7698 | 0 .00016 | 0 .00035 |
| ANKRD33B 158 .5128902 | -0 . 152142106 | 0 .24168 -0 .6295 | 0 .52901 | 0 .58285 |
| ANKRD34B 17 .45597095 | -0 .872063817 | 0 .42883 -2 .0336 | 0 .042 | 0 .05964 |
| ANKRD34C 17 .06754852 | -1 .473724755 | 0 .53493 -2 .755 | 0 .00587 | 0 .00977 |
| ANKRD35 1004 .310839 | -0 .881594826 | 0 .25853 -3 .4101 | 0 .00065 | 0 .00126 |
| ANKRD36 7039 .779909 | -1 .652591239 | 0 . 16995 -9 .7239 | 2 .39E-22 | 4 .69E-21 |
| ANKRD36C 5759 .258551 | -1 .263731229 | 0 . 16076 -7 .8607 | 3 .82E-15 | 3 .33E-14 |
| ANKRD37 557 .3206636 | -0 .446928721 | 0 . 15559 -2 .8724 | 0 .00407 | 0 .00696 |
| ANKRD39 489 .0975127 | -0 .310613435 | 0 .09831 -3 . 1596 | 0 .00158 | 0 .00288 |
| ANKRD40 3523 .927447 | 0 .539289794 | 0 .082 6 .57645 | 4 .82E-1 1 | 2 .59E-10 |
| ANKRD42 797 . 1584491 | -0 .401379274 | 0 .06579 -6 . 1011 | 1 .05E-09 | 4 .77E-09 |
| ANKRD44 4721 .582114 | 0 .375654015 | 0 . 1 1231 3 .34488 | 0 .00082 | 0 .00157 |
| ANKRD45 94 .03564165 | -1 .372197502 | 0 .32642 -4 .2038 | 2 .63E-05 | 6 .34E-05 |
| ANKRD46 2390 .325095 | -0 .670830987 | 0 . 12168 -5 .5131 | 3 .53E-08 | 1 .31E-07 |
| ANKRD49 1633 .063377 | -0 .754623474 | 0 .09057 -8 .3315 | 7 .98E-17 | 8 .52E-16 |
| ANKRD50 13399 .5826 | -0 .796762536 | 0 . 12164 -6 .55 | 5 .75E-1 1 | 3 .05E-10 |
| ANKRD52 3260 . 177367 | 0 .850705072 | 0 . 1 1552 7 .36421 | 1 .78E-13 | 1 .28E-12 |
| ANKRD53 55 .75472101 | -1 .330247027 | 0 . 16926 -7 .859 | 3 .87E-15 | 3 .38E-14 |
| ANKRD54 1352 .477654 | 0 .230992002 | 0 . 12593 1 .83432 | 0 .06661 | 0 .09091 |
| ANKRD55 89 .49484407 | -1 .766136372 | 0 . 1983 -8 .9065 | 5 .27E-19 | 7 .21E-18 |
| ANKRD6 830 .7241409 | -0 .430103175 | 0 . 10103 -4 .2573 | 2 .07E-05 | 5 .09E-05 |
| ANKRD60 2 .32334906 | -2 .886345346 | 0 .74259 -3 .8869 | 0 .0001 | 0 .00022 |
| ANKRD61 129 .8620945 | 0 .042132269 | 0 . 1879 0 .22423 | 0 .82258 | 0 .84992 |
| ANKRD62 18 . 1 1755635 | -3 .523723369 | 0 .47156 -7 .4725 | 7 .87E-14 | 5 .84E-13 |
| ANKRD63 0 .616868959 | -1 .55816611 1 | 0 .97441 -1 .5991 | 0 . 1098 | 0 . 14347 |
| ANKRD65 139 .5866164 | 0 . 101179599 | 0 .2055 0 .49235 | 0 .62247 | 0 .67144 |
| ANKRD66 17 .57632412 | -2 .057102725 | 0 .54099 -3 .8025 | 0 .00014 | 0 .00031 |
| ANKRD7 88 .25781818 | -0 .519736134 | 0 .29871 -1 .7399 | 0 .08187 | 0 . 10988 |
| ANKRD9 661 .4485232 | 1 . 1 10977303 | 0 . 17484 6 .35409 | 2 . 10E-10 | 1 .04E-09 |
| ANKS1A 1 1021 .36493 | -0 .954788981 | 0 .20456 -4 .6676 | 3 .05E-06 | 8 .43E-06 |
| ANKS1B 450 .8922779 | -0 .499301374 | 0 .2338 -2 . 1356 | 0 .03272 | 0 .0474 |
| ANKS3 873 .6737804 | 0 .056043355 | 0 .0793 0 .70675 | 0 .47972 | 0 .535 |
| ANKS4B 14 . 13004078 | -2 .828527147 | 0 .35022 -8 .0765 | 6 .67E-16 | 6 .33E-15 |
| ANKS6 965 .5085738 | 0 .426981555 | 0 . 10686 3 .99561 | 6 .45E-05 | 0 .00015 |
| ANKUB1 128 .0318768 | -0 .686070798 | 0 . 15689 -4 .3729 | 1 .23E-05 | 3 . 1 1E-05 |
| ANKZF1 1904 .691165 | -0 .555389809 | 0 .09784 -5 .6765 | 1 .37E-08 | 5 .38E-08 |
| ANLN 2257 .684182 | 1 . 1 13527201 | 0 . 19016 5 .8556 | 4 .75E-09 | 1 .98E-08 |
| ANO1 1902 .838208 | -0 .779872322 | 0 . 19687 -3 .9614 | 7 .45E-05 | 0 .00017 |
| ANO10 2359 .494446 | 0 .020446093 | 0 .08173 0 .25017 | 0 .80246 | 0 .83186 |
| ANO2 199 .578541 | 0 .405770781 | 0 .21542 1 .88361 | 0 .05962 | 0 .08217 |
| ANO3 180 . 1849068 | -0 .869182387 | 0 .31871 -2 .7271 | 0 .00639 | 0 .01054 |
| ANO4 499 . 134914 | 2 .064129883 | 0 .36468 5 .6601 | 1 .51E-08 | 5 .89E-08 |
| ANO5 806 .8497773 | -1 .409353896 | 0 .30337 -4 .6457 | 3 .39E-06 | 9 .31E-06 |
| ANO6 1 1867 .25488 | 0 . 169806175 | 0 .07924 2 . 1429 | 0 .03212 | 0 .04663 |
| ANO7 57 .71443136 | -0 . 1 13601006 | 0 . 18765 -0 .6054 | 0 .54491 | 0 .59786 |
| ANO8 731 .6336995 | 0 .052478522 | 0 . 16201 0 .32391 | 0 .746 | 0 .78196 |
| ANO9 1256 .031767 | -0 .717217688 | 0 .21309 -3 .3659 | 0 .00076 | 0 .00147 |
| ANOS1 1 193 .478449 | 0 .682529723 | 0 .20141 3 .38871 | 0 .0007 | 0 .00136 |
| ANP32A 4169 .348542 | -0 .000692041 | 0 .08967 -0 .0077 | 0 .99384 | 0 .99478 |
| ANP32B 528 .00936 | 0 . 1 15375546 | 0 . 13141 0 .87798 | 0 .37996 | 0 .43616 |
| ANP32D 2 .087761225 | -3 .469047127 | 0 .65747 -5 .2764 | 1 .32E-07 | 4 .49E-07 |
| ANP32E 2810 .76291 | 0 .039350697 | 0 . 10665 0 .36898 | 0 .71214 | 0 .75286 |
| ANPEP 1096 .592199 | 1 .492229777 | 0 . 14305 10 .4315 | 1 .78E-25 | 4 .93E-24 |
| ANTXR1 8220 .606391 | 0 . 192090907 | 0 . 13333 1 .44074 | 0 . 14966 | 0 . 18976 |

| ANTXR2 ANXA1 ANXA10 ANXA11 ANXA13 ANXA2 ANXA2R ANXA3 ANXA4 ANXA5 ANXA6 ANXA7 ANXA9 AOAH AOC1 AOC2 AOC3 AOX1 | 3609 .822302 34029 .40102 22 .7574233 4974 .888049 19 .93871261 75748 .5819 231 .4038837 1 199 .834907 5293 .246696 32810 .41621 12267 .68555 5288 .069066 1281 .557215 1036 . 142186 154 .2343795 60 .56061729 825 .350394 2529 .409055 |
| --- | --- |

AP000322 .5351 . 17859413 AP000679 .2 16 . 10052193 AP000708 . 1 9 . 138168532 AP001631 . 104 .286605291 AP002884 .2 0 .346141034 AP003068 .2353 .31979156

AP003774 .4 20 .711029 AP006621 .5 136 . 1791661 AP1AR 3333 .003703 AP1B1 5839 .018569 AP1G1 10270 .33644 AP1G2 1 144 .880013 AP1M1 1704 .351403 AP1M2 764 .8636831 AP1S1 3591 .837802 AP1S2 19867 .85979 AP1S3 770 .4832757 AP2A1 3609 . 150044 AP2A2 2480 .982759 AP2B1 7791 . 143364 AP2M1 10309 .26134 AP2S1 3518 . 1 14893 AP3B1 6991 .86864 AP3B2 127 .4616107 AP3D1 5339 .631699 AP3M1 2240 .044331 AP3M2 4692 .874097 AP3S1 3449 .551076 AP3S2 1 142 .684131 AP4B1 868 .8065688 AP4E1 3206 . 18719 AP4M1 816 .2190205 AP4S1 662 .8668132 AP5B1 846 .8354483 AP5M1 2932 . 191392 AP5S1 975 .5742604 AP5Z1 2404 .699005 APAF1 2561 . 162632

0 .235796896

-0 . 17143692

-0 .854541685

0 .090141322

-1 .296196934

0 . 197469964

0 .379025283

0 .684298162

-0 .546561671

0 . 100363082

-0 . 180184501

-0 .085987534

-1 .270410785

2 .270943305

0 .893333704

0 .014222135

2 .364218374

1 .269415431

0 .202778677

-0 .293108893

-2 .877357947

-0 .846574683

0 .850961621

-0 .059756257

0 .411 141571

0 .08798833

-0 . 167361334

0 .857044938

0 . 140442185

-0 .293337217

1 .224170359

-1 .063785275

0 .468129717

0 .750670933

0 . 185000592

0 .584338269

0 .574730019

0 .406950067

0 .208698763

0 .583396197

0 .211561728

-0 .3067369

0 .894311687

0 . 105676539

-0 . 104988268

-0 .881992047

0 .53630639

-0 . 126286329

0 .030592118

0 . 176628779

-0 .562136561

0 .636347089

-0 .20598844

0 .785776762

0 .909003566

0 .435435831

0 . 12685 0 . 1766 0 .26109 0 . 10112 0 .36272 0 .09888 0 . 12781 0 .20482 0 .08409 0 . 14959 0 . 1 1981 0 .07603 0 . 15554 0 . 18425 0 .21065 0 . 17281 0 .23571 0 .26092 0 .23564 0 .29982 0 .40922 0 .48601 1 . 16947 0 . 17871 0 .27951 0 . 1 189 0 . 1 1671 0 .09949 0 .0926 0 . 10384 0 .09867 0 .2627 0 . 15705 0 .22275 0 . 1474 0 .08826 0 .08884 0 .09109 0 . 10664 0 . 14308 0 .06635 0 .20399 0 .08177 0 .06474 0 . 15386 0 .0947 0 .08291 0 .06955 0 .06318 0 .09218 0 .0704 0 . 1057 0 .06721 0 . 10811 0 . 1063 0 . 10006

1 .85885 -0 .9708 -3 .273 0 .89145 -3 .5735 1 .99701 2 .96542 3 .34101 -6 .4995 0 .67093 -1 .504 -1 . 131 -8 . 1675 12 .3251 4 .24081 0 .0823 10 .03 4 .8651 0 .86054 -0 .9776 -7 .0312 -1 .7419 0 .72765 -0 .3344 1 .47095 0 .74 -1 .434 8 .61402 1 .51659 -2 .8249 12 .4071 -4 .0494 2 .98073 3 .37003 1 .25513 6 .62085 6 .4695 4 .4678 1 .95705 4 .07748 3 . 18835 -1 .5037 10 .9372 1 .63236 -0 .6823 -9 .3137 6 .4689 -1 .8159 0 .48423 1 .91607 -7 .9849 6 .0204 -3 .0647 7 .26844 8 .55142 4 .35189

0 .06305 0 .33166 0 .00106 0 .37269 0 .00035 0 .04582 0 .00302 0 .00083 8 .06E-1 1 0 .50227 0 . 13259 0 .25807 3 . 15E-16 6 .64E-35 2 .23E-05 0 .93441 1 . 12E-23 1 . 14E-06 0 .38949 0 .32826 2 .05E-12 0 .08153 0 .46683 0 .7381 0 . 14131 0 .4593 0 . 15158 7 .05E-18 0 . 12937 0 .00473 2 .39E-35 5 . 13E-05 0 .00288 0 .00075 0 .20943 3 .57E-1 1 9 .83E-1 1 7 .90E-06 0 .05034 4 .55E-05 0 .00143 0 . 13266 7 .65E-28 0 . 1026 0 .49502 1 .23E-20 9 .87E-1 1 0 .06939 0 .62822 0 .05536 1 .41E-15 1 .74E-09 0 .00218 3 .64E-13 1 .22E-17 1 .35E-05

0 .08644 0 .38664 0 .002 0 .42864 0 .00071 0 .06465 0 .00527 0 .00159 4 . 19E-10 0 .55716 0 . 17007 0 .30939 3 . 13E-15 4 .68E-33 5 .45E-05 0 .94535 2 .54E-22 3 .38E-06 0 .44577 0 .38319 1 .31E-1 1 0 . 10943 0 .52261 0 .77524 0 . 18012 0 .51503 0 . 19198 8 .44E-17 0 . 16641 0 .00799 1 .79E-33 0 .00012 0 .00504 0 .00145 0 .25681 1 .95E-10 5 .05E-10 2 .06E-05 0 .07042 0 .00011 0 .00263 0 . 17015 2 .71E-26 0 . 1348 0 .55001 2 .03E-19 5 .07E-10 0 .09437 0 .67665 0 .07674 1 .28E-14 7 .65E-09 0 .00389 2 .52E-12 1 .42E-16 3 .41E-05

| APBA1 782 .5602276 | -1 . 123753929 | 0 . 16619 | -6 .7618 | 1 .36E-1 1 | 7 .80E-1 1 |
| --- | --- | --- | --- | --- | --- |
| APBA2 1394 .892089 | 1 .218889002 | 0 .24021 | 5 .07434 | 3 .89E-07 | 1 .23E-06 |
| APBA3 560 .6075465 | 0 .634259771 | 0 .08588 | 7 .38528 | 1 .52E-13 | 1 . 10E-12 |
| APBB1 1756 . 196449 | -0 . 100410521 | 0 . 10439 | -0 .9619 | 0 .33609 | 0 .39122 |
| APBB1IP 461 .8636478 | 2 .281519553 | 0 . 19368 | 1 1 .7796 | 4 .98E-32 | 2 .70E-30 |
| APBB2 4684 . 184947 | -0 .420072616 | 0 . 1 1806 | -3 .5581 | 0 .00037 | 0 .00075 |
| APBB3 717 .8568894 | 0 .042018131 | 0 .09109 | 0 .4613 | 0 .64458 | 0 .69128 |
| APC 7412 .98117 | -0 .299112461 | 0 .07367 | -4 .0603 | 4 .90E-05 | 0 .00011 |
| APC2 189 .0165231 | 1 . 191802839 | 0 . 12505 | 9 .53038 | 1 .57E-21 | 2 .83E-20 |
| APCDD1 10195 .7671 | -0 .632680583 | 0 .229 | -2 .7628 | 0 .00573 | 0 .00955 |
| APCDD1L 74 .79899076 | -0 .508583686 | 0 . 19197 | -2 .6493 | 0 .00807 | 0 .01306 |
| APCS 1 .960217749 | -3 .233265958 | 1 .00326 | -3 .2227 | 0 .00127 | 0 .00236 |
| APEH 5680 .676474 | 0 .304307171 | 0 .09924 | 3 .06624 | 0 .00217 | 0 .00387 |
| APEX1 2540 .565708 | -0 .258944783 | 0 . 15396 | -1 .6819 | 0 .09258 | 0 . 12292 |
| APEX2 1054 .883059 | 0 .2074511 12 | 0 .06103 | 3 .39907 | 0 .00068 | 0 .00131 |
| APH1A 8284 . 167357 | 0 . 169449322 | 0 . 10303 | 1 .64462 | 0 . 10005 | 0 . 1317 |
| APH1B 4454 .910829 | -1 .296702531 | 0 . 15135 | -8 .5678 | 1 .05E-17 | 1 .24E-16 |
| API5 5317 .853327 | -0 . 103752265 | 0 .05869 | -1 .7678 | 0 .0771 | 0 . 10401 |
| APIP 1201 .969566 | -0 .48216781 | 0 . 1 1306 | -4 .2648 | 2 .00E-05 | 4 .93E-05 |
| APITD1 121 .7032236 | 0 .631372445 | 0 . 16278 | 3 .87858 | 0 .00011 | 0 .00023 |
| APLF 1859 .592924 | -1 .77094689 | 0 . 12077 | -14 .664 | 1 .09E-48 | 2 .88E-46 |
| APLN 183 . 1240566 | 2 .381098478 | 0 .3231 | 7 .36951 | 1 .71E-13 | 1 .23E-12 |
| APLNR 836 .2748745 | 1 .907624717 | 0 . 17841 | 10 .6924 | 1 . 10E-26 | 3 .46E-25 |
| APLP1 39 .91610862 | 0 .772079284 | 0 .27523 | 2 .80518 | 0 .00503 | 0 .00846 |
| APLP2 16864 .54469 | 0 .290791978 | 0 . 10162 | 2 .86149 | 0 .00422 | 0 .00719 |
| APMAP 8939 .596221 | 1 .075990638 | 0 . 16149 | 6 .66288 | 2 .69E-1 1 | 1 .49E-10 |
| APOA1 13 .06769086 | 0 . 192644165 | 0 .3232 | 0 .59605 | 0 .55115 | 0 .60367 |
| APOA2 3 .585236226 | -0 . 150014113 | 0 .42508 | -0 .3529 | 0 .72415 | 0 .7636 |
| APOA4 1 .204175069 | -2 .595535008 | 1 .06279 | -2 .4422 | 0 .0146 | 0 .02253 |
| APOA5 4 .731792497 | -1 .238479858 | 0 .49266 | -2 .5139 | 0 .01194 | 0 .01872 |
| APOB 297 .8914899 | -1 .034230604 | 0 .31828 | -3 .2495 | 0 .00116 | 0 .00216 |
| APOBEC1 3 .017428881 | -4 .708084566 | 0 .85401 | -5 .5129 | 3 .53E-08 | 1 .31E-07 |
| APOBEC2 107 . 1297866 | -0 .464221316 | 0 . 19197 | -2 .4182 | 0 .0156 | 0 .02394 |
| APOBEC3A 195 .8462936 | 3 .006294748 | 0 .391 | 7 .68877 | 1 .49E-14 | 1 .20E-13 |
| APOBEC3B 94 . 15176334 | 1 .500628515 | 0 .21545 | 6 .965 | 3 .28E-12 | 2 .04E-1 1 |
| APOBEC3C 1211 .363121 | 0 .495390923 | 0 . 1 198 | 4 . 13525 | 3 .55E-05 | 8 .39E-05 |
| APOBEC3D 157 . 1288228 | 1 .379769742 | 0 . 17648 | 7 .81806 | 5 .36E-15 | 4 .57E-14 |
| APOBEC3F 198 .8208204 | 0 .098208022 | 0 . 13037 | 0 .75329 | 0 .45128 | 0 .50714 |
| APOBEC3G 1023 .219344 | 1 . 190670576 | 0 . 19048 | 6 .25103 | 4 .08E-10 | 1 .95E-09 |
| APOBEC3H 37 .27583629 | 1 .6291644 | 0 .26617 | 6 . 12066 | 9 .32E-10 | 4 .25E-09 |
| APOBEC4 46 . 18417734 | -0 .933628436 | 0 .2144 | -4 .3546 | 1 .33E-05 | 3 .37E-05 |
| APOBR 239 .7905236 | 1 .983498698 | 0 .20555 | 9 .64994 | 4 .92E-22 | 9 .30E-21 |
| APOC1 4964 .444494 | 0 .54651392 | 0 .34216 | 1 .59724 | 0 . 1 1021 | 0 . 14393 |
| APOC2 2 .510007351 | -0 .218105651 | 0 .4494 | -0 .4853 | 0 .62744 | 0 .67589 |
| APOC3 2 . 109702234 | -1 .472548607 | 0 .70498 | -2 .0888 | 0 .03673 | 0 .05273 |
| APOC4 0 .263831876 | -1 .72354097 | 1 .47633 | -1 . 1675 | 0 .24303 | 0 .29347 |
| APOD 13530 .25666 | 0 .549215763 | 0 .29171 | 1 .88274 | 0 .05974 | 0 .08232 |
| APOE 61667 .67894 | 1 .228689525 | 0 .22098 | 5 .56021 | 2 .69E-08 | 1 .01E-07 |
| APOF 12 .88240414 | -2 .672632833 | 0 .29252 | -9 . 1365 | 6 .45E-20 | 9 .81E-19 |
| APOH 4 .450571407 | -4 .258536772 | 0 .77453 | -5 .4982 | 3 .84E-08 | 1 .41E-07 |
| APOL1 1702 .817066 | 2 .594132762 | 0 .20922 | 12 .3993 | 2 .64E-35 | 1 .95E-33 |
| APOL2 2630 .596976 | 1 .632143104 | 0 . 13422 | 12 . 16 | 5 .08E-34 | 3 .35E-32 |
| APOL3 1830 .607847 | 1 .441238892 | 0 . 19542 | 7 .37497 | 1 .64E-13 | 1 . 19E-12 |
| APOL4 567 .4031488 | 1 .228496701 | 0 .20779 | 5 .91211 | 3 .38E-09 | 1 .43E-08 |
| APOL5 4 .447074766 | -1 .583317739 | 0 .53478 | -2 .9607 | 0 .00307 | 0 .00535 |
| APOL6 15053 .75897 | 2 .056573012 | 0 . 18738 | 10 .9757 | 5 .00E-28 | 1 .81E-26 |

| APOLD1 2815 .57314 | 2 .601374525 | 0 .24571 | 10 .587 | 3 .43E-26 | 1 .02E-24 |
| --- | --- | --- | --- | --- | --- |
| APOM 144 .9919562 | -0 .462437557 | 0 . 10324 | -4 .479 | 7 .50E-06 | 1 .96E-05 |
| APOO 398 .833103 | 0 . 142326618 | 0 . 19977 | 0 .71246 | 0 .47618 | 0 .53167 |
| APOOL 776 .3203545 | -0 .323210558 | 0 .06963 | -4 .6415 | 3 .46E-06 | 9 .49E-06 |
| APOOP5 3 . 1 15931731 | -3 .516900614 | 0 .62868 | -5 .5941 | 2 .22E-08 | 8 .46E-08 |
| APOPT1 838 .7927662 | -0 .335023605 | 0 .09358 | -3 .5799 | 0 .00034 | 0 .0007 |
| APP 22760 .76156 | -0 .272013777 | 0 . 14472 | -1 .8795 | 0 .06017 | 0 .08289 |
| APPBP2 7022 .505561 | -0 . 177474854 | 0 .09474 | -1 .8733 | 0 .06103 | 0 .08393 |
| APPL1 1 1 124 .242 | -0 .403446941 | 0 . 10305 | -3 .9152 | 9 .03E-05 | 0 .0002 |
| APPL2 6266 .457558 | -0 .571768966 | 0 .08869 | -6 .4469 | 1 . 14E-10 | 5 .83E-10 |
| APRT 5555 .743687 | -0 .379586692 | 0 . 12599 | -3 .0128 | 0 .00259 | 0 .00457 |
| APTX 1747 .3497 | -0 .409715013 | 0 .07604 | -5 .3879 | 7 . 13E-08 | 2 .53E-07 |
| AQP1 18728 .20484 | -1 .28813315 | 0 .2003 | -6 .4312 | 1 .27E-10 | 6 .43E-10 |
| AQP10 13 .75790148 | -1 . 122405752 | 0 .28798 | -3 .8975 | 9 .72E-05 | 0 .00021 |
| AQP11 311 .8262878 | -0 . 19057647 | 0 . 14213 | -1 .3409 | 0 . 17997 | 0 .22451 |
| AQP12A 0 .617220255 | -1 . 143349697 | 1 .09344 | -1 .0456 | 0 .29572 | 0 .34929 |
| AQP12B 1 . 191162006 | -0 .215393368 | 0 .92679 | -0 .2324 | 0 .81622 | 0 .84416 |
| AQP2 10 .44906351 | 0 .023468139 | 0 .44601 | 0 .05262 | 0 .95804 | 0 .96472 |
| AQP3 9538 .291929 | -0 .088852745 | 0 .25977 | -0 .342 | 0 .73232 | 0 .77035 |
| AQP4 106 .6563038 | -1 .896570353 | 0 .30008 | -6 .3203 | 2 .61E-10 | 1 .28E-09 |
| AQP5 983 .2402072 | 6 . 174026811 | 0 .35473 | 17 .4048 | 7 .59E-68 | 1 .46E-64 |
| AQP6 26 .55070146 | 0 .852580279 | 0 .40312 | 2 . 1 1496 | 0 .03443 | 0 .04968 |
| AQP7 62 .45587326 | 0 . 142427009 | 0 .35966 | 0 .396 | 0 .6921 | 0 .73445 |
| AQP8 10 .40294658 | -0 .06652747 | 0 .3908 | -0 . 1702 | 0 .86482 | 0 .88621 |
| AQP9 1 126 .093707 | -1 .221282735 | 0 .23107 | -5 .2853 | 1 .25E-07 | 4 .29E-07 |
| AQR 6617 .675191 | -0 . 1 12714072 | 0 .0728 | -1 .5483 | 0 . 12156 | 0 . 15723 |
| AR 1848 .557564 | -0 .2509881 | 0 .26064 | -0 .963 | 0 .33556 | 0 .39067 |
| ARAF 937 .860252 | 0 .201018332 | 0 .07623 | 2 .63699 | 0 .00836 | 0 .01351 |
| ARAP1 5329 .784112 | 0 .664098122 | 0 .08604 | 7 .71825 | 1 . 18E-14 | 9 .64E-14 |
| ARAP2 10349 .81386 | -0 .482775451 | 0 . 14646 | -3 .2962 | 0 .00098 | 0 .00185 |
| ARAP3 828 .7999785 | 0 .518506565 | 0 . 15414 | 3 .36397 | 0 .00077 | 0 .00148 |
| ARC 54 .34296181 | 1 .099427345 | 0 .26501 | 4 . 14863 | 3 .34E-05 | 7 .95E-05 |
| ARCN1 9443 .692615 | 0 .428185061 | 0 .07409 | 5 .77937 | 7 .50E-09 | 3 .05E-08 |
| AREG 44 .71713781 | 1 . 198027914 | 0 .34714 | 3 .45115 | 0 .00056 | 0 .00109 |
| AREL1 3113 . 142553 | 0 .353492882 | 0 .08029 | 4 .40258 | 1 .07E-05 | 2 .74E-05 |
| ARF1 8828 .772968 | 0 .237622468 | 0 . 10222 | 2 .32467 | 0 .02009 | 0 .03035 |
| ARF3 6201 .781457 | 0 .479724756 | 0 .08096 | 5 .92514 | 3 . 12E-09 | 1 .33E-08 |
| ARF4 10838 .61809 | 0 .764394231 | 0 . 12532 | 6 .0996 | 1 .06E-09 | 4 .82E-09 |
| ARF5 5021 .985122 | -0 .053554117 | 0 .09587 | -0 .5586 | 0 .57643 | 0 .62806 |
| ARF6 3841 .670305 | 1 .047692004 | 0 .09977 | 10 .5009 | 8 .55E-26 | 2 .46E-24 |
| ARFGAP1 4272 .681052 | 1 .32296985 | 0 . 13074 | 10 . 1 19 | 4 .55E-24 | 1 .08E-22 |
| ARFGAP2 6065 .084695 | -0 .400026613 | 0 .05471 | -7 .3113 | 2 .65E-13 | 1 .86E-12 |
| ARFGAP3 3399 .514485 | 0 .21674263 | 0 .09707 | 2 .23295 | 0 .02555 | 0 .03787 |
| ARFGEF1 9948 .547664 | -0 .085149431 | 0 .09418 | -0 .9041 | 0 .36592 | 0 .42169 |
| ARFGEF2 13885 .53986 | 0 .469064196 | 0 .09309 | 5 .03866 | 4 .69E-07 | 1 .47E-06 |
| ARFGEF3 1978 .656193 | -0 .31511 1306 | 0 .23369 | -1 .3484 | 0 . 17752 | 0 .22176 |
| ARFIP1 4896 .758786 | -0 .538652894 | 0 . 10192 | -5 .2853 | 1 .25E-07 | 4 .29E-07 |
| ARFIP2 2301 .420081 | -0 . 12615305 | 0 .0921 | -1 .3698 | 0 . 17075 | 0 .21408 |
| ARFRP1 2167 .631076 | 0 .361577194 | 0 .08314 | 4 .34886 | 1 .37E-05 | 3 .46E-05 |
| ARG1 3448 .876092 | -1 . 175502593 | 0 .2625 | -4 .4781 | 7 .53E-06 | 1 .97E-05 |
| ARG2 371 .4142029 | -0 .208242701 | 0 . 16911 | -1 .2314 | 0 .21816 | 0 .26624 |
| ARGFX 3 .559142915 | -3 .405921623 | 0 .8989 | -3 .789 | 0 .00015 | 0 .00032 |
| ARGLU1 8369 .231069 | -0 .222338274 | 0 . 12236 | -1 .817 | 0 .06921 | 0 .09416 |
| ARHGAP1 2739 .781446 | 0 .963702714 | 0 . 10568 | 9 . 1 1872 | 7 .60E-20 | 1 . 15E-18 |
| ARHGAP10 3704 .794822 | -1 .08981372 | 0 . 1 1318 | -9 .6289 | 6 .04E-22 | 1 . 13E-20 |
| ARHGAP11A1394 .433613 | 1 . 161152089 | 0 . 14847 | 7 .82053 | 5 .26E-15 | 4 .49E-14 |

ARHGAP11B481 . 1218172

ARHGAP12 8057 .596245 ARHGAP15 1441 .515243 ARHGAP17 2064 .455892 ARHGAP18 6287 .680116 ARHGAP19 1290 .639332

ARHGAP19-S9 .606204172

| ARHGAP20 | 1439 .080677 |
| --- | --- |
| ARHGAP21 | 8674 .919987 |
| ARHGAP22 | 364 .0416018 |
| ARHGAP24 | 2982 .84594 |
| ARHGAP25 | 980 .8676443 |
| ARHGAP26 | 2125 .731081 |
| ARHGAP27 | 1499 .379539 |
| ARHGAP28 | 1 128 .211687 |
| ARHGAP29 | 6567 .757693 |
| ARHGAP30 | 2407 .568402 |
| ARHGAP31 | 7650 .395339 |
| ARHGAP32 | 7203 .02151 |
| ARHGAP33 | 174 .5484447 |
| ARHGAP35 | 6801 .527444 |
| ARHGAP36 | 8 .656473883 |
| ARHGAP39 | 446 .053188 |
| ARHGAP4 | 1458 .00979 |
| ARHGAP40 | 393 .314382 |
| ARHGAP42 | 5448 .461489 |
| ARHGAP44 | 161 .0017639 |
| ARHGAP45 | 2572 .947517 |
| ARHGAP5 | 26479 .429 |
| ARHGAP6 | 457 .3215412 |
| ARHGAP8 | 79 .70977485 |
| ARHGAP9 | 561 .9165492 |
| ARHGDIA | 2150 .264732 |
| ARHGDIB | 3663 .617211 |
| ARHGDIG | 10 .35039392 |
| ARHGEF1 | 2808 .597552 |
| ARHGEF10 | 2866 .012456 |

ARHGEF10L 3839 .445602

| ARHGEF11 ARHGEF12 ARHGEF15 ARHGEF16 ARHGEF17 ARHGEF18 ARHGEF19 ARHGEF2 ARHGEF25 ARHGEF26 ARHGEF28 ARHGEF3 ARHGEF33 ARHGEF35 ARHGEF37 ARHGEF38 ARHGEF39 ARHGEF4 | 6829 .052119 29316 .47201 288 .5390276 263 .099491 1938 .695542 1094 .76803 820 .21403 3753 .902484 389 .0674342 1662 .943932 3563 .203269 2005 .531104 229 .2824839 212 .4303602 5608 .070264 1 10 .7925287 194 . 1 164998 2193 .216283 |
| --- | --- |

0 .332163418

-0 .959052965

0 .833077042

0 .545369562

0 .557233856

0 .052615179

-1 .429039481

-1 .882058997

0 .210470693

1 .20687902

-1 . 194718825

1 .325419726

0 .609972018

0 .61168673

0 .506198516

-0 .748284325

1 .665956146

-0 .818606881

-0 .64600017

0 .025627872

0 .053492289

-3 .077724754

1 .677044846

1 .653940369

-0 .9412211 12

-1 .086086893

-0 .443957906

2 .423304708

-0 .781350083

0 .202650351

1 .376948215

1 .739956451

0 .815494103

1 .218002265

0 .849814459

1 . 106010377

-0 . 1 17544043

0 .259791689

0 .96092324

-0 .34825636

1 . 104524242

-0 .482999066

0 .760173367

0 .806444604

-0 .450013766

1 . 162491464

0 .510393823

-1 .013842398

-1 . 167240846

0 .668354546

-1 . 189126975

-0 .411268639

-1 .866042435

-0 .417345179

0 .276510016

-0 .460452573

0 . 12763 0 .09358 0 . 17392 0 . 1 1936 0 . 1379 0 . 13704 0 .33465 0 . 19845 0 . 1 1 124 0 . 13085 0 . 16551 0 . 17319 0 . 10343 0 . 1 1859 0 . 18808 0 . 1528 0 . 14153 0 . 1294 0 . 14696 0 . 17233 0 .09879 0 .65393 0 . 18663 0 . 13045 0 .30663 0 . 16429 0 . 1789 0 . 15003 0 . 10381 0 . 17772 0 .29714 0 . 16937 0 . 1 1817 0 . 17307 0 .4468 0 .08936 0 . 1 1371 0 .08617 0 . 12913 0 .08494 0 . 17751 0 . 19237 0 . 14269 0 .07127 0 . 14864 0 .09975 0 . 12754 0 . 17462 0 . 19106 0 . 10574 0 . 19403 0 .24251 0 .2006 0 .25253 0 . 1 1644 0 .23452

2 .60265 -10 .249 4 .79003 4 .56919 4 .04087 0 .38394 -4 .2703 -9 .4839 1 .89207 9 .22324 -7 .2186 7 .65289 5 .89754 5 . 15782 2 .69142 -4 .8973 1 1 .7708 -6 .326 -4 .3959 0 . 14871 0 .54149 -4 .7065 8 .98571 12 .6782 -3 .0695 -6 .6109 -2 .4815 16 . 1526 -7 .5269 1 . 14025 4 .63396 10 .2731 6 .90086 7 .0375 1 .90199 12 .3768 -1 .0337 3 .0148 7 .4416 -4 . 1002 6 .22227 -2 .5107 5 .3275 1 1 .3151 -3 .0276 1 1 .6544 4 .00182 -5 .806 -6 . 1094 6 .32082 -6 . 1284 -1 .6959 -9 .3023 -1 .6527 2 .37472 -1 .9634

0 .00925 1 .20E-24 1 .67E-06 4 .90E-06 5 .33E-05 0 .70102 1 .95E-05 2 .45E-21 0 .05848 2 .88E-20 5 .25E-13 1 .97E-14 3 .69E-09 2 .50E-07 0 .00711 9 .72E-07 5 .52E-32 2 .52E-10 1 . 10E-05 0 .88178 0 .58817 2 .52E-06 2 .57E-19 7 .81E-37 0 .00214 3 .82E-1 1 0 .01308 1 .09E-58 5 .20E-14 0 .25418 3 .59E-06 9 .32E-25 5 . 17E-12 1 .96E-12 0 .05717 3 .49E-35 0 .30126 0 .00257 9 .95E-14 4 . 13E-05 4 .90E-10 0 .01205 9 .96E-08 1 . 10E-29 0 .00246 2 . 18E-31 6 .29E-05 6 .40E-09 1 .00E-09 2 .60E-10 8 .87E-10 0 .08991 1 .37E-20 0 .0984 0 .01756 0 .0496

0 .01482 3 .04E-23 4 .79E-06 1 .32E-05 0 .00012 0 .74272 4 .82E-05 4 .32E-20 0 .08072 4 .55E-19 3 .57E-12 1 .57E-13 1 .55E-08 8 . 16E-07 0 .01165 2 .90E-06 2 .96E-30 1 .23E-09 2 .82E-05 0 .90083 0 .63918 7 .05E-06 3 .66E-18 6 .90E-35 0 .00383 2 .08E-10 0 .02037 9 .50E-56 3 .97E-13 0 .30522 9 .82E-06 2 .41E-23 3 . 13E-1 1 1 .25E-1 1 0 .07906 2 .57E-33 0 .35498 0 .00454 7 .29E-13 9 .67E-05 2 .31E-09 0 .01887 3 .46E-07 4 .74E-28 0 .00436 1 .09E-29 0 .00014 2 .62E-08 4 .55E-09 1 .27E-09 4 .06E-09 0 . 1 1973 2 .26E-19 0 . 12974 0 .02674 0 .06949

| ARHGEF40 5259 .273038 0 .326782364 | 0 . 13379 2 .44244 | 0 .01459 | 0 .02252 |
| --- | --- | --- | --- |
| ARHGEF5 709 .6841925 -0 .763647004 | 0 . 179 -4 .2663 | 1 .99E-05 | 4 .90E-05 |
| ARHGEF6 3870 . 1 10617 0 .010512197 | 0 . 12632 0 .08322 | 0 .93368 | 0 .94486 |
| ARHGEF7 4790 .503512 -0 .046118773 | 0 .09301 -0 .4959 | 0 .61999 | 0 .66914 |
| ARHGEF9 2250 .355468 -0 .922071079 | 0 .0947 -9 .7363 | 2 . 1 1E-22 | 4 . 18E-21 |
| ARID1A 3586 .993832 0 .671550464 | 0 . 12643 5 .31147 | 1 .09E-07 | 3 .76E-07 |
| ARID1B 7828 .042926 -0 .272197778 | 0 .08255 -3 .2973 | 0 .00098 | 0 .00185 |
| ARID2 6729 .841688 -0 . 150695432 | 0 .07184 -2 .0978 | 0 .03592 | 0 .05166 |
| ARID3A 572 .5116269 1 .602082083 | 0 . 10588 15 . 1308 | 1 .01E-51 | 3 .60E-49 |
| ARID3B 318 .8057728 0 .525030504 | 0 . 12825 4 .09371 | 4 .25E-05 | 9 .92E-05 |
| ARID3C 9 .802244464 -1 .269274695 | 0 .28482 -4 .4565 | 8 .33E-06 | 2 . 16E-05 |
| ARID4A 3541 .810893 -0 .576297646 | 0 .0943 -6 . 1 1 12 | 9 .89E-10 | 4 .50E-09 |
| ARID4B 5257 . 171191 -0 .013257225 | 0 .09237 -0 . 1435 | 0 .88587 | 0 .9044 |
| ARID5A 966 .8610936 1 .990034102 | 0 . 12933 15 .3876 | 1 .98E-53 | 7 .45E-51 |
| ARID5B 1 1246 .98745 -0 .806813933 | 0 . 1 1462 -7 .0393 | 1 .93E-12 | 1 .24E-1 1 |
| ARIH1 8113 .735523 -0 . 160903549 | 0 .06029 -2 .6688 | 0 .00761 | 0 .01239 |
| ARIH2 4819 .386757 0 .356913827 | 0 .06747 5 .29027 | 1 .22E-07 | 4 . 19E-07 |
| ARIH2OS 124 .7202167 -0 .212857898 | 0 .09687 -2 . 1973 | 0 .028 | 0 .04115 |
| ARL1 6136 .467684 -0 .465034781 | 0 .08853 -5 .2529 | 1 .50E-07 | 5 .05E-07 |
| ARL10 2080 .280607 -0 .099523369 | 0 . 14169 -0 .7024 | 0 .48242 | 0 .5376 |
| ARL11 188 . 1501269 1 .392836387 | 0 . 17234 8 .08206 | 6 .37E-16 | 6 .07E-15 |
| ARL13A 60 .695898 -0 .818665212 | 0 . 18732 -4 .3704 | 1 .24E-05 | 3 . 15E-05 |
| ARL13B 1263 .665898 -0 .215958371 | 0 .07879 -2 .7411 | 0 .00612 | 0 .01015 |
| ARL14 32 .63828455 -0 .611607775 | 0 .29578 -2 .0678 | 0 .03866 | 0 .05525 |
| ARL14EP 3210 .776265 -0 .651393134 | 0 .0869 -7 .4963 | 6 .56E-14 | 4 .93E-13 |
| ARL14EPL 3 .214576322 -4 .480105273 | 0 .80552 -5 .5618 | 2 .67E-08 | 1 .01E-07 |
| ARL15 3171 .305859 -0 .701641045 | 0 . 10952 -6 .4066 | 1 .49E-10 | 7 .49E-10 |
| ARL16 1053 .23436 0 .232589821 | 0 .09547 2 .43618 | 0 .01484 | 0 .02289 |
| ARL17A 203 .6301525 -0 .752154285 | 0 . 16736 -4 .4942 | 6 .98E-06 | 1 .83E-05 |
| ARL17B 96 .03718387 -0 .760270278 | 0 . 16628 -4 .5722 | 4 .83E-06 | 1 .30E-05 |
| ARL2 1528 .629252 -0 .307592185 | 0 . 1373 -2 .2403 | 0 .02507 | 0 .03721 |
| ARL2-SNX150 .278296313 -1 .386983957 | 1 . 1 1832 -1 .2402 | 0 .21489 | 0 .26281 |
| ARL2BP 3825 .62528 0 .0967421 | 0 .09822 0 .98493 | 0 .32466 | 0 .37955 |
| ARL3 964 .7600355 -0 .649762904 | 0 . 10396 -6 .2498 | 4 . 1 1E-10 | 1 .96E-09 |
| ARL4A 4811 .862584 -0 .722281215 | 0 . 13872 -5 .2069 | 1 .92E-07 | 6 .37E-07 |
| ARL4C 1826 .251458 1 .616575042 | 0 . 13084 12 .3558 | 4 .53E-35 | 3 .29E-33 |
| ARL4D 791 .4185645 -0 . 122662238 | 0 . 16922 -0 .7249 | 0 .46853 | 0 .52429 |
| ARL5A 15107 .50824 -0 .458467278 | 0 . 1 1339 -4 .0432 | 5 .27E-05 | 0 .00012 |
| ARL5B 5621 .994128 -0 .285549081 | 0 . 1055 -2 .7066 | 0 .0068 | 0 .011 16 |
| ARL5C 26 .9707529 -0 .565413244 | 0 .26283 -2 . 1512 | 0 .03146 | 0 .04578 |
| ARL6 615 .5128841 -0 .77532042 | 0 .08896 -8 .7153 | 2 .90E-18 | 3 .64E-17 |
| ARL6IP1 8223 .506075 0 .03898517 | 0 .09603 0 .40596 | 0 .68477 | 0 .7278 |
| ARL6IP4 39 .94478708 -0 .005138086 | 0 . 13743 -0 .0374 | 0 .97018 | 0 .97444 |
| ARL6IP5 1 1394 .87715 -0 .36381008 | 0 . 10921 -3 .3312 | 0 .00086 | 0 .00165 |
| ARL6IP6 1666 .959082 -0 .838366053 | 0 .08732 -9 .6013 | 7 .90E-22 | 1 .47E-20 |
| ARL8A 1360 .472918 0 .656515208 | 0 .09166 7 . 16272 | 7 .91E-13 | 5 .28E-12 |
| ARL8B 10933 .59325 0 .00693002 | 0 .08211 0 .0844 | 0 .93274 | 0 .94416 |
| ARL9 67 .39763221 -1 . 153670206 | 0 .22316 -5 . 1697 | 2 .35E-07 | 7 .68E-07 |
| ARMC1 2475 .588011 0 .019381699 | 0 .08946 0 .21665 | 0 .82848 | 0 .85505 |
| ARMC10 3031 .405571 0 .038347845 | 0 .09893 0 .38763 | 0 .69829 | 0 .74015 |
| ARMC12 15 .70271918 -0 .569595484 | 0 .28268 -2 .015 | 0 .04391 | 0 .06215 |
| ARMC2 356 .4060759 -0 .465408616 | 0 . 10942 -4 .2534 | 2 . 1 1E-05 | 5 . 17E-05 |
| ARMC3 71 . 13678738 -1 .537997063 | 0 .36627 -4 . 199 | 2 .68E-05 | 6 .47E-05 |
| ARMC4 60 .08186213 -0 .308593432 | 0 .40655 -0 .759 | 0 .44782 | 0 .50361 |
| ARMC5 509 .2830895 0 .845505908 | 0 .0913 9 .26058 | 2 .03E-20 | 3 .27E-19 |
| ARMC6 1492 .490404 0 .727078362 | 0 . 10087 7 .20818 | 5 .67E-13 | 3 .84E-12 |

| ARMC7 ARMC8 ARMC9 ARMCX1 ARMCX2 ARMCX3 ARMCX4 ARMCX5 ARMCX6 ARMS2 ARMT1 ARNT ARNT2 ARNTL ARNTL2 ARPC1A ARPC1B ARPC2 ARPC3 ARPC4 | 417 . 1533947 3960 .523168 2057 .285649 3854 .813851 1558 .594426 4085 .065918 1225 .496811 1273 .319868 829 .8289068 4 .546272908 2572 .016234 4108 . 140002 4688 .724893 1268 .815372 1943 .384694 6890 .067958 7369 .27925 14964 . 17667 6046 .888608 5556 .520661 |
| --- | --- |

ARPC4-TTLL0 .268250925

| ARPC5 ARPC5L ARPIN ARPP19 ARPP21 ARR3 ARRB1 ARRB2 ARRDC1 | 15818 .97771 1880 .092551 801 .6729684 18514 . 19774 49 .03140778 81 .69155023 3329 .958646 972 .3766341 1054 .572751 |
| --- | --- |

ARRDC1-AS 273 .2027741

| ARRDC2 ARRDC3 ARRDC4 ARRDC5 ARSA ARSB ARSD ARSE ARSF ARSG ARSH ARSI ARSJ ARSK ART1 ART3 ART4 ART5 ARTN ARV1 ARVCF ARX AS3MT ASAH1 ASAH2 | 1523 . 154863 24077 . 15696 6796 .557672 27 .63248959 907 .2607235 2893 .805998 3552 .877255 322 .8367776 159 .437537 950 .3808592 17 .68753983 618 .8933837 606 .3221853 756 . 1048212 6 .631418256 177 .3543442 1 13 .2057087 29 .27051346 24 .09481283 1033 .618639 715 .5451294 13 .51712922 6 .461491998 34925 .32611 43 .42870025 |
| --- | --- |

0 .358711457

-0 .348015017

1 .546443423

-1 .324474987

-0 . 109861147

-0 .016722561

-0 .562097025

-0 .535876828

-0 .40994918

-2 . 145261873

-0 .265411289

-0 . 106242341

-0 .05574677

-0 .476736341

0 .234041005

0 . 106469817

1 .574260424

0 .04866066

0 .071056908

0 .491438096

-0 .377495196

0 .644249057

0 .224230799

0 . 185423097

0 .208079725

-2 . 165859589

-0 .994208548

0 .329720939

1 .576027064

0 .353801061

0 .391583576

0 .679809338

-1 .288915403

-0 .282944622

0 .86663429

0 .644961051

0 .872916062

-0 .496995247

-0 .534604525

-0 .63829991

0 .295844578

-0 .410260017

1 .298454708

0 .646043274

-0 .526102602

-1 .323735983

-1 .643184121

-0 . 187357401

-1 .438241851

0 .557382652

-0 .24691967

0 .440970854

1 .07160237

-0 .791378385

0 .035350846

-0 .875349732

0 .07437 0 .06155 0 . 16781 0 . 12912 0 . 18005 0 . 1 1517 0 . 1378 0 .09713 0 . 12503 0 .42494 0 . 15388 0 .06653 0 . 18663 0 . 12902 0 . 14656 0 . 12635 0 . 18838 0 .07627 0 . 12456 0 .09556 1 .20779 0 . 12411 0 . 10951 0 . 10375 0 .0775 0 .48106 0 .22174 0 .09275 0 . 12566 0 .0914 0 .09072 0 . 10595 0 . 1368 0 .09508 0 .28 0 . 10874 0 . 1 1895 0 .09751 0 .23326 0 .33659 0 . 12742 0 .38039 0 .2898 0 . 16225 0 .09576 0 .33313 0 .30583 0 .21628 0 .25497 0 .23899 0 . 10611 0 . 10805 0 .47593 0 .29916 0 . 17204 0 .21358

4 .82343 -5 .6544 9 .21523 -10 .258 -0 .6102 -0 . 1452 -4 .0792 -5 .5172 -3 .2788 -5 .0484 -1 .7248 -1 .597 -0 .2987 -3 .6951 1 .59688 0 .84263 8 .35677 0 .638 0 .57047 5 . 14267 -0 .3126 5 . 19114 2 .04764 1 .78727 2 .68493 -4 .5022 -4 .4836 3 .55493 12 .5425 3 .87112 4 .3162 6 .41642 -9 .4222 -2 .9757 3 .09515 5 .93105 7 .33834 -5 .0966 -2 .2919 -1 .8964 2 .32185 -1 .0785 4 .48054 3 .98183 -5 .4938 -3 .9736 -5 .3729 -0 .8663 -5 .6409 2 .3322 -2 .3271 4 .08125 2 .25159 -2 .6453 0 .20548 -4 .0985

1 .41E-06 1 .56E-08 3 . 1 1E-20 1 .09E-24 0 .54176 0 .88456 4 .52E-05 3 .44E-08 0 .00104 4 .45E-07 0 .08455 0 . 1 1027 0 .76516 0 .00022 0 . 1 1029 0 .39944 6 .45E-17 0 .52347 0 .56836 2 .71E-07 0 .75462 2 .09E-07 0 .04059 0 .07389 0 .00725 6 .72E-06 7 .34E-06 0 .00038 4 .37E-36 0 .00011 1 .59E-05 1 .40E-10 4 .42E-21 0 .00292 0 .00197 3 .01E-09 2 . 16E-13 3 .46E-07 0 .02191 0 .05791 0 .02024 0 .2808 7 .45E-06 6 .84E-05 3 .93E-08 7 .08E-05 7 .75E-08 0 .38634 1 .69E-08 0 .01969 0 .01996 4 .48E-05 0 .02435 0 .00816 0 .8372 4 . 16E-05

4 . 10E-06 6 .08E-08 4 .89E-19 2 .80E-23 0 .59485 0 .90314 0 .00011 1 .28E-07 0 .00196 1 .40E-06 0 . 1 1317 0 . 14398 0 .79864 0 .00046 0 . 144 0 .45604 6 .94E-16 0 .57761 0 .62032 8 .81E-07 0 .78922 6 .90E-07 0 .05781 0 . 10006 0 .01186 1 .77E-05 1 .92E-05 0 .00076 3 .51E-34 0 .00024 3 .97E-05 7 .04E-10 7 .57E-20 0 .00511 0 .00354 1 .28E-08 1 .54E-12 1 . 1 1E-06 0 .03286 0 .07999 0 .03056 0 .33362 1 .95E-05 0 .00015 1 .45E-07 0 .00016 2 .74E-07 0 .44267 6 .55E-08 0 .02977 0 .03016 0 .0001 0 .03623 0 .01321 0 .86224 9 .73E-05

| ASAH2B | 194 .8663404 | -0 .260693973 | 0 . 12814 -2 .0345 | 0 .0419 | 0 .05952 |
| --- | --- | --- | --- | --- | --- |
| ASAP1 | 7768 . 173413 | -0 .252436354 | 0 . 10866 -2 .3232 | 0 .02017 | 0 .03046 |
| ASAP2 | 2312 .66242 | -0 . 1 18546619 | 0 . 10322 -1 . 1485 | 0 .25078 | 0 .30174 |
| ASAP3 | 2878 .95468 | -0 .664161805 | 0 . 1 1997 -5 .536 | 3 .09E-08 | 1 . 16E-07 |
| ASB1 | 2113 .986323 | 0 . 157928754 | 0 .0741 2 . 13125 | 0 .03307 | 0 .04786 |
| ASB10 | 2 .738898339 | -1 .783506515 | 0 .75862 -2 .351 | 0 .01872 | 0 .0284 |
| ASB11 | 871 .817852 | -1 . 177870593 | 0 .45602 -2 .583 | 0 .0098 | 0 .0156 |
| ASB12 | 1 .039838899 | -2 .560398906 | 0 .64174 -3 .9898 | 6 .61E-05 | 0 .00015 |
| ASB13 | 1878 .452824 | 0 .519942234 | 0 . 10831 4 .80038 | 1 .58E-06 | 4 .57E-06 |
| ASB14 | 270 .2564617 | -0 .766338462 | 0 . 14824 -5 . 1696 | 2 .35E-07 | 7 .68E-07 |
| ASB15 | 16 .66732208 | -2 .544334102 | 0 .41454 -6 . 1377 | 8 .37E-10 | 3 .85E-09 |
| ASB16 | 51 .77107081 | 0 .428058515 | 0 . 1916 2 .2341 | 0 .02548 | 0 .03777 |
| ASB17 | 5 .229284535 | -2 .413620489 | 0 .61887 -3 .9001 | 9 .62E-05 | 0 .00021 |
| ASB18 | 30 .96015433 | -2 .01354832 | 0 .42177 -4 .774 | 1 .81E-06 | 5 . 16E-06 |
| ASB2 | 291 .8408022 | 2 .95439318 | 0 .21835 13 .5306 | 1 .03E-41 | 1 .38E-39 |
| ASB3 | 323 .2727462 | -0 .513224594 | 0 .08698 -5 .9008 | 3 .62E-09 | 1 .52E-08 |
| ASB4 | 68 .01998565 | 0 .200554555 | 0 .38503 0 .52088 | 0 .60245 | 0 .65267 |
| ASB5 | 15 .6476594 | -2 .760338793 | 0 .5509 -5 .0106 | 5 .43E-07 | 1 .68E-06 |
| ASB6 | 1 108 .067191 | 0 .201694246 | 0 .09681 2 .08335 | 0 .03722 | 0 .05336 |
| ASB7 | 1853 .882094 | 0 .254083145 | 0 .06829 3 .72066 | 0 .0002 | 0 .00042 |
| ASB8 | 2588 .877871 | -0 .227367832 | 0 .05939 -3 .8287 | 0 .00013 | 0 .00028 |
| ASB9 | 1345 .703288 | -1 .002416684 | 0 .2427 -4 . 1302 | 3 .62E-05 | 8 .56E-05 |
| ASCC1 | 1252 .992162 | -0 .347682535 | 0 .05028 -6 .9144 | 4 .70E-12 | 2 .86E-1 1 |
| ASCC2 | 2018 .22919 | 0 .379831609 | 0 . 1 1546 3 .28972 | 0 .001 | 0 .00189 |
| ASCC3 | 9506 . 130343 | -0 .02586401 | 0 .0968 -0 .2672 | 0 .78931 | 0 .82032 |
| ASCL1 | 8 .479809516 | -0 .631236141 | 0 .59255 -1 .0653 | 0 .28675 | 0 .33988 |
| ASCL2 | 137 .6874161 | 0 .210967495 | 0 . 18106 1 . 16519 | 0 .24394 | 0 .29438 |
| ASCL3 | 6 .258946509 | -0 .736159274 | 0 .3439 -2 . 1406 | 0 .03231 | 0 .04687 |
| ASCL4 | 66 .96438659 | 1 .572777315 | 0 .38952 4 .03777 | 5 .40E-05 | 0 .00012 |
| ASCL5 | 6 .529245276 | 0 .303507034 | 0 .32062 0 .94663 | 0 .34383 | 0 .39935 |
| ASF1A | 2542 .69182 | -0 .758908236 | 0 .09848 -7 .7062 | 1 .30E-14 | 1 .05E-13 |
| ASF1B | 427 .3748266 | 2 .243457641 | 0 .20402 10 .9964 | 3 .98E-28 | 1 .46E-26 |
| ASGR1 | 99 . 1899192 | -0 .370390191 | 0 . 19461 -1 .9032 | 0 .05701 | 0 .07886 |
| ASGR2 | 46 .58054094 | -0 .261523434 | 0 .21809 -1 . 1992 | 0 .23046 | 0 .27983 |
| ASH1L | 14410 .71063 | -0 .479410391 | 0 .0823 -5 .8252 | 5 .70E-09 | 2 .35E-08 |
| ASH2L | 3665 . 186024 | -0 .248365173 | 0 .09196 -2 .7009 | 0 .00691 | 0 .01134 |
| ASIC1 | 78 .84483122 | -0 .07019089 | 0 .23866 -0 .2941 | 0 .76868 | 0 .80174 |
| ASIC2 | 15 .37028281 | -1 . 1 19570021 | 0 .34929 -3 .2053 | 0 .00135 | 0 .0025 |
| ASIC3 | 40 .46632966 | 0 .419609368 | 0 . 18818 2 .22978 | 0 .02576 | 0 .03817 |
| ASIC4 | 25 .51188072 | -0 .71637276 | 0 .26773 -2 .6757 | 0 .00746 | 0 .01216 |
| ASIC5 | 7 .302450119 | -3 .047004534 | 0 .58318 -5 .2248 | 1 .74E-07 | 5 .81E-07 |
| ASIP | 68 .22735822 | 0 .634383793 | 0 .29357 2 . 16096 | 0 .0307 | 0 .04475 |
| ASL | 947 .3030735 | 0 .425813276 | 0 . 13296 3 .20252 | 0 .00136 | 0 .00252 |
| ASMT | 10 .58172959 | -0 .835983681 | 0 .30941 -2 .7019 | 0 .00689 | 0 .01131 |
| ASMTL | 1257 .248608 | -0 .05421924 | 0 .08492 -0 .6385 | 0 .52316 | 0 .57733 |
| ASNA1 | 1776 .200501 | 0 .055643727 | 0 . 14267 0 .39003 | 0 .69652 | 0 .73856 |
| ASNS | 816 .0767404 | -0 .535067169 | 0 . 12373 -4 .3245 | 1 .53E-05 | 3 .83E-05 |
| ASNSD1 | 5076 .233089 | -0 .599279664 | 0 . 12906 -4 .6435 | 3 .42E-06 | 9 .40E-06 |
| ASPA | 1414 .547153 | -0 .274269298 | 0 . 17641 -1 .5547 | 0 . 12001 | 0 . 15548 |
| ASPDH | 27 .66242136 | 0 .330143355 | 0 .25793 1 .27995 | 0 .20056 | 0 .24729 |
| ASPG | 270 .4057697 | -0 .831362188 | 0 .27609 -3 .0112 | 0 .0026 | 0 .00459 |
| ASPH | 14743 .01298 | 0 .208332217 | 0 .08822 2 .36137 | 0 .01821 | 0 .02768 |
| ASPHD1 | 60 .3329886 | 1 .707258437 | 0 .27316 6 .2501 | 4 . 10E-10 | 1 .96E-09 |
| ASPHD2 | 245 .7306413 | 0 .332235662 | 0 . 10464 3 . 17518 | 0 .0015 | 0 .00275 |
| ASPM | 3332 .753064 | 1 .239305917 | 0 . 17742 6 .98519 | 2 .84E-12 | 1 .78E-1 1 |
| ASPN | 2156 .960459 | 0 .277778234 | 0 .23902 1 . 16218 | 0 .24516 | 0 .29564 |

| ASPRV1 | 23697 .52188 | -1 . 140118669 | 0 .26773 | -4 .2584 | 2 .06E-05 | 5 .07E-05 |
| --- | --- | --- | --- | --- | --- | --- |
| ASPSCR1 | 1364 .452515 | 0 .725302834 | 0 .08968 | 8 .08802 | 6 .06E-16 | 5 .80E-15 |
| ASRGL1 | 259 .8911311 | 2 .681403521 | 0 .26097 | 10 .2747 | 9 . 17E-25 | 2 .38E-23 |
| ASS1 | 2654 . 188248 | -0 .92616049 | 0 .23751 | -3 .8995 | 9 .64E-05 | 0 .00021 |
| ASTE1 | 882 .8979 | -0 .730690246 | 0 .0577 | -12 .663 | 9 .48E-37 | 8 . 19E-35 |
| ASTL | 18 .94764047 | -0 .771015269 | 0 .28196 | -2 .7345 | 0 .00625 | 0 .01033 |
| ASTN1 | 79 .21523615 | -1 .793590457 | 0 .35641 | -5 .0324 | 4 .84E-07 | 1 .51E-06 |
| ASTN2 | 506 .8767117 | -0 .020012428 | 0 . 12595 | -0 . 1589 | 0 .87375 | 0 .89388 |
| ASUN | 2006 .626401 | -0 .02593171 | 0 .09578 | -0 .2707 | 0 .7866 | 0 .81789 |
| ASXL1 | 6553 .029659 | 0 .260066513 | 0 . 1 1281 | 2 .30534 | 0 .02115 | 0 .0318 |
| ASXL2 | 611 1 .314051 | 0 .2011947 | 0 .07418 | 2 .7124 | 0 .00668 | 0 .01098 |
| ASXL3 | 94 .82073319 | -0 .939334052 | 0 .3887 | -2 .4166 | 0 .01566 | 0 .02404 |
| ASZ1 | 1 1 .78752526 | -3 .08465386 | 0 .79287 | -3 .8905 | 0 .0001 | 0 .00022 |
| ATAD1 | 7608 .26073 | -0 .524632138 | 0 . 1 1013 | -4 .7636 | 1 .90E-06 | 5 .42E-06 |
| ATAD2 | 2488 .778877 | 1 .073348071 | 0 . 14206 | 7 .55577 | 4 . 16E-14 | 3 .22E-13 |
| ATAD2B | 5216 .080412 | -0 .567822195 | 0 . 10622 | -5 .3456 | 9 .01E-08 | 3 . 15E-07 |
| ATAD3A | 1 107 .606723 | 1 .54704398 | 0 . 13147 | 1 1 .7669 | 5 .78E-32 | 3 . 10E-30 |
| ATAD3B | 2596 .985407 | 1 .714861588 | 0 . 19561 | 8 .76682 | 1 .84E-18 | 2 .38E-17 |
| ATAD3C | 35 .40885374 | 0 .491038855 | 0 .23171 | 2 . 1 1923 | 0 .03407 | 0 .0492 |
| ATAD5 | 1368 .368883 | 0 .252658143 | 0 .09747 | 2 .59221 | 0 .00954 | 0 .01522 |
| ATAT1 | 266 .7267284 | 0 .067130684 | 0 . 14774 | 0 .45437 | 0 .64956 | 0 .69573 |
| ATCAY | 10 .32586779 | -2 .979127049 | 0 .5199 | -5 .7302 | 1 .00E-08 | 4 .00E-08 |
| ATE1 | 4724 .563406 | -0 .254693834 | 0 .06552 | -3 .8872 | 0 .0001 | 0 .00022 |
| ATF1 | 1747 .382771 | -0 .222919945 | 0 . 10736 | -2 .0764 | 0 .03785 | 0 .05418 |
| ATF2 | 4936 .885817 | -0 .370926324 | 0 .09054 | -4 .097 | 4 . 19E-05 | 9 .79E-05 |
| ATF3 | 781 .5156798 | 1 .350337829 | 0 .20861 | 6 .47299 | 9 .61E-1 1 | 4 .95E-10 |
| ATF4 | 13823 .8952 | -0 .447793786 | 0 . 12593 | -3 .5559 | 0 .00038 | 0 .00076 |
| ATF5 | 276 . 1200577 | 1 .317258158 | 0 . 12772 | 10 .3138 | 6 . 10E-25 | 1 .61E-23 |
| ATF6 | 9167 .639274 | 0 . 12141535 | 0 .06698 | 1 .81274 | 0 .06987 | 0 .09497 |
| ATF6B | 2252 .302942 | 0 .438890271 | 0 .08699 | 5 .04557 | 4 .52E-07 | 1 .42E-06 |
| ATF7 | 1572 .830446 | 0 .067701014 | 0 . 1022 | 0 .66243 | 0 .50769 | 0 .56217 |
| ATF7IP | 6541 . 165979 | -0 .214977165 | 0 .0955 | -2 .251 | 0 .02438 | 0 .03628 |
| ATF7IP2 | 1098 . 169397 | -0 .574145504 | 0 . 18358 | -3 . 1275 | 0 .00176 | 0 .00319 |
| ATG10 | 954 .425574 | -0 .859058856 | 0 .09936 | -8 .6462 | 5 .33E-18 | 6 .47E-17 |
| ATG101 | 1 191 . 140339 | 0 .071497791 | 0 . 12939 | 0 .5526 | 0 .58054 | 0 .63175 |
| ATG12 | 4693 .053454 | -0 .374936122 | 0 .09536 | -3 .9316 | 8 .44E-05 | 0 .00019 |
| ATG13 | 3227 .89821 | 0 .090879699 | 0 .05936 | 1 .53092 | 0 . 12579 | 0 . 16214 |
| ATG14 | 4048 .932732 | -0 .232361394 | 0 .0696 | -3 .3384 | 0 .00084 | 0 .00161 |
| ATG16L1 | 3096 .985343 | -0 . 153189199 | 0 .07298 | -2 .0991 | 0 .03581 | 0 .05151 |
| ATG16L2 | 1502 .043643 | -0 .260369125 | 0 . 14773 | -1 .7625 | 0 .07799 | 0 . 10508 |
| ATG2A | 1822 .613694 | 0 .603486683 | 0 . 1 1491 | 5 .25176 | 1 .51E-07 | 5 .08E-07 |
| ATG2B | 9569 .27142 | -0 .553793084 | 0 .09076 | -6 . 1018 | 1 .05E-09 | 4 .75E-09 |
| ATG3 | 3679 .980443 | -0 .245122127 | 0 .08101 | -3 .0258 | 0 .00248 | 0 .00439 |
| ATG4A | 1 141 .594282 | -0 .055134865 | 0 .08824 | -0 .6248 | 0 .53208 | 0 .58569 |
| ATG4B | 2210 .505892 | 0 .359999387 | 0 .08772 | 4 . 10411 | 4 .06E-05 | 9 .51E-05 |
| ATG4C | 1682 .89261 | -1 .398934826 | 0 . 1 1 152 | -12 .544 | 4 .26E-36 | 3 .44E-34 |
| ATG4D | 609 .0381817 | 0 .763708463 | 0 . 10294 | 7 .41885 | 1 . 18E-13 | 8 .62E-13 |
| ATG5 | 3368 .474345 | -0 .399424729 | 0 . 10006 | -3 .9918 | 6 .56E-05 | 0 .00015 |
| ATG7 | 3326 .286692 | -0 . 178229152 | 0 .06768 | -2 .6336 | 0 .00845 | 0 .01363 |
| ATG9A | 2591 .018094 | 0 .527852276 | 0 .09596 | 5 .50072 | 3 .78E-08 | 1 .39E-07 |
| ATG9B | 423 .5587431 | -0 .078433207 | 0 .27514 | -0 .2851 | 0 .77559 | 0 .80781 |
| ATHL1 | 1072 .019831 | 1 .052002488 | 0 . 13424 | 7 .83668 | 4 .63E-15 | 3 .97E-14 |
| ATIC | 2043 .695683 | -0 . 171742781 | 0 . 1 1505 | -1 .4927 | 0 . 13551 | 0 . 17352 |
| ATL1 | 1 162 .286736 | -1 .403010358 | 0 .0951 | -14 .754 | 2 .91E-49 | 8 .22E-47 |
| ATL2 | 6425 .431304 | -0 .41042268 | 0 . 1 1643 | -3 .5251 | 0 .00042 | 0 .00085 |
| ATL3 | 23236 .89568 | 0 .267565346 | 0 . 10828 | 2 .47099 | 0 .01347 | 0 .02092 |

| ATM ATMIN ATN1 ATOH1 ATOH7 ATOH8 ATOX1 ATP10A ATP10B ATP10D ATP11A ATP11AUN ATP11B ATP11C ATP12A ATP13A1 ATP13A2 ATP13A3 ATP13A4 ATP13A5 ATP1A1 ATP1A2 ATP1A3 ATP1A4 ATP1B1 ATP1B2 ATP1B3 ATP1B4 ATP23 ATP2A1 ATP2A2 ATP2A3 ATP2B1 ATP2B2 ATP2B3 ATP2B4 ATP2C1 ATP2C2 ATP4A ATP4B ATP5A1 ATP5B ATP5C1 ATP5D ATP5E ATP5EP2 ATP5F1 ATP5G1 ATP5G2 ATP5G3 ATP5H ATP5I ATP5J ATP5J2 | 24698 .06776 7522 . 146724 1 121 .951898 3 .344607072 10 .22224025 431 .0817323 2635 . 1 19883 2171 .301554 2695 .35241 4824 .431389 3271 .27089 8 . 124240353 14710 .78786 3572 .730897 1 109 .609998 1841 .856394 1415 .706178 1 1 148 .41238 1601 .809989 252 .5161545 22707 .48109 253 .9211 105 28 .92930479 27 . 1831873 3376 .452787 91 .37246078 7602 .717272 1 1 .86217192 490 . 1030685 67 .36942686 12556 .01952 1215 .536864 8793 .217377 67 .87490049 21 .63243658 17984 .96308 10744 .82789 817 .2304628 15 .71419869 51 .21963241 35343 .05436 15279 .60853 14108 .6713 1387 .608687 1 1995 .81222 17 .615166 12674 . 1 1 139 1764 .355835 7158 .644534 6380 .243284 5743 .837786 2200 .046444 1958 .742744 2115 .901511 |
| --- | --- |

ATP5J2-PTC 3 .250068907 ATP5L 3577 .432412

-0 . 100550343

-0 .092081575

0 .588375071

-1 .242918915

-1 .520328131

0 .072582044

0 .604659853

0 .21378394

-0 .625747305

-0 .501312396

0 .900066001

-1 .062803101

-0 .561193833

0 .333196352

0 .470103399

1 .058914497

1 .627391391

0 .628481775

-1 .213228768

-0 .946387311

0 .540433866

0 .931945066

0 .396630542

-1 .923457818

0 .788456395

0 . 189674432

0 .329090499

-4 .33846604

-0 .791260249

0 .049500129

0 .751229316

2 .09672828

-0 .33556834

-0 .991258807

-1 .090081399

-0 .29399906

-0 . 109517766

-0 .77203856

-1 .696654098

-3 .869927075

-0 .268045839

0 .443238492

-1 . 161440882

0 .571489167

0 . 178000579

0 .955022748

-0 .220059158

-0 . 12036998

-0 .926775685

0 .412155846

-0 .313203342

-0 . 126386647

-0 .21833059

0 .451082939

-0 .33896913

-0 .696836449

0 . 1 17 0 .06852 0 . 18677 0 .63674 0 .35058 0 .22059 0 . 12258 0 . 17821 0 . 18934 0 .08564 0 . 13576 0 .65374 0 .08027 0 .0914 0 .41018 0 .08756 0 . 1317 0 . 10466 0 .2811 1 0 .3349 0 . 16746 0 .33622 0 .33862 0 .36763 0 . 16463 0 .29285 0 .09016 0 .64846 0 . 10619 0 . 19425 0 .08743 0 . 1911 0 .09784 0 .25224 0 .49055 0 . 1 1 181 0 .07486 0 .22928 0 .21236 0 .3382 0 . 1 1225 0 .09966 0 . 12778 0 . 10879 0 . 17704 0 .45021 0 . 1 1852 0 . 16149 0 . 17067 0 . 14585 0 . 1401 0 . 16818 0 . 12588 0 . 17456 0 .37641 0 . 16134

-0 .8594 -1 .3438 3 . 15026 -1 .952 -4 .3366 0 .32904 4 .93295 1 . 19962 -3 .3049 -5 .8539 6 .62991 -1 .6257 -6 .9917 3 .64556 1 . 1461 12 .093 12 .3569 6 .0048 -4 .3158 -2 .8259 3 .22733 2 .77183 1 . 17132 -5 .232 4 .78927 0 .64768 3 .65021 -6 .6905 -7 .451 0 .25482 8 .59246 10 .9722 -3 .4297 -3 .9298 -2 .2221 -2 .6295 -1 .463 -3 .3672 -7 .9896 -1 1 .443 -2 .3879 4 .44758 -9 .0891 5 .25307 1 .00542 2 . 1213 -1 .8567 -0 .7454 -5 .4304 2 .82592 -2 .2355 -0 .7515 -1 .7344 2 .58412 -0 .9005 -4 .319

0 .3901 0 . 17901 0 .00163 0 .05094 1 .45E-05 0 .74212 8 . 10E-07 0 .23029 0 .00095 4 .80E-09 3 .36E-1 1 0 . 10401 2 .72E-12 0 .00027 0 .25175 1 . 15E-33 4 .47E-35 1 .92E-09 1 .59E-05 0 .00472 0 .00125 0 .00557 0 .24147 1 .68E-07 1 .67E-06 0 .51719 0 .00026 2 .22E-1 1 9 .26E-14 0 .79886 8 .51E-18 5 .20E-28 0 .0006 8 .50E-05 0 .02627 0 .00855 0 . 14345 0 .00076 1 .35E-15 2 .55E-30 0 .01694 8 .68E-06 9 .99E-20 1 .50E-07 0 .3147 0 .0339 0 .06336 0 .45605 5 .62E-08 0 .00471 0 .02538 0 .45235 0 .08285 0 .00976 0 .36783 1 .57E-05

0 .44636 0 .22343 0 .00297 0 .07119 3 .64E-05 0 .7787 2 .45E-06 0 .27968 0 .0018 1 .99E-08 1 .85E-10 0 . 13652 1 .71E-1 1 0 .00055 0 .30262 7 .33E-32 3 .26E-33 8 .38E-09 3 .97E-05 0 .00797 0 .00232 0 .00931 0 .29182 5 .61E-07 4 .81E-06 0 .57147 0 .00054 1 .25E-10 6 .82E-13 0 .82883 1 .01E-16 1 .87E-26 0 .00118 0 .00019 0 .03887 0 .01378 0 . 18261 0 .00146 1 .23E-14 1 . 13E-28 0 .02587 2 .25E-05 1 .49E-18 5 .05E-07 0 .36911 0 .04898 0 .08684 0 .51184 2 .02E-07 0 .00797 0 .03764 0 .50825 0 . 1 1 108 0 .01556 0 .42369 3 .92E-05

| ATP5L2 | 16 . 17604156 | -0 .24729595 | 0 .21569 | -1 . 1466 | 0 .25157 | 0 .3025 |
| --- | --- | --- | --- | --- | --- | --- |
| ATP5O | 1654 .805761 | -0 .594755268 | 0 . 14343 | -4 . 1468 | 3 .37E-05 | 8 .01E-05 |
| ATP5S | 1032 .987205 | -0 .296674727 | 0 .07401 | -4 .0088 | 6 . 10E-05 | 0 .00014 |
| ATP5SL | 1455 .620765 | -0 .076149395 | 0 .09878 | -0 .7709 | 0 .44075 | 0 .49667 |
| ATP6AP1 | 5870 .312627 | 0 .823650367 | 0 . 10185 | 8 .08728 | 6 . 10E-16 | 5 .84E-15 |
| ATP6AP1L | 270 .2791828 | -0 .554899615 | 0 . 1412 | -3 .9298 | 8 .50E-05 | 0 .00019 |
| ATP6AP2 | 14140 .48908 | 0 .064110909 | 0 . 10076 | 0 .63626 | 0 .52461 | 0 .57866 |
| ATP6V0A1 | 5048 .600201 | 0 .366445416 | 0 .07887 | 4 .64605 | 3 .38E-06 | 9 .30E-06 |
| ATP6V0A2 | 4071 .96396 | 0 .458014251 | 0 .07574 | 6 .04755 | 1 .47E-09 | 6 .53E-09 |
| ATP6V0A4 | 509 .7871858 | 1 .910106982 | 0 .33238 | 5 .74668 | 9 . 10E-09 | 3 .65E-08 |
| ATP6V0B | 6561 .934066 | 1 .488277173 | 0 . 16835 | 8 .84031 | 9 .55E-19 | 1 .27E-17 |
| ATP6V0C | 17 .58951461 | 0 . 188307701 | 0 . 19131 | 0 .9843 | 0 .32497 | 0 .37986 |
| ATP6V0D1 | 3792 .33013 | 1 . 173585709 | 0 . 12616 | 9 .30217 | 1 .38E-20 | 2 .26E-19 |
| ATP6V0D2 | 314 .3163046 | 3 .704886385 | 0 .45475 | 8 . 14699 | 3 .73E-16 | 3 .69E-15 |
| ATP6V0E1 | 9091 .253264 | 0 .071434058 | 0 . 13745 | 0 .51971 | 0 .60327 | 0 .65341 |
| ATP6V0E2 | 1 166 .296703 | 1 .515177006 | 0 . 1814 | 8 .35285 | 6 .66E-17 | 7 . 16E-16 |
| ATP6V1A | 15780 .97864 | 0 .424921005 | 0 . 10164 | 4 . 18063 | 2 .91E-05 | 6 .98E-05 |
| ATP6V1B1 | 211 .2914774 | 1 .52906866 | 0 .25685 | 5 .95327 | 2 .63E-09 | 1 . 13E-08 |
| ATP6V1B2 | 22094 .69244 | -0 .047808856 | 0 . 13135 | -0 .364 | 0 .71587 | 0 .75611 |
| ATP6V1C1 | 5935 .272178 | 0 .641227648 | 0 . 1 1059 | 5 .79809 | 6 .71E-09 | 2 .74E-08 |
| ATP6V1C2 | 1213 .814336 | -0 .599735978 | 0 .24264 | -2 .4717 | 0 .01345 | 0 .02088 |
| ATP6V1D | 4567 .32578 | -0 .013473239 | 0 . 10051 | -0 . 1341 | 0 .89336 | 0 .91093 |
| ATP6V1E1 | 8226 .452653 | 0 .315417232 | 0 . 12006 | 2 .62716 | 0 .00861 | 0 .01387 |
| ATP6V1E2 | 524 . 1832571 | -0 .069020941 | 0 . 1 1504 | -0 .6 | 0 .54852 | 0 .60135 |
| ATP6V1F | 2816 . 127832 | 0 .353068069 | 0 . 18996 | 1 .85865 | 0 .06308 | 0 .08647 |
| ATP6V1G1 | 8380 .348134 | -0 .216097084 | 0 . 13882 | -1 .5567 | 0 . 1 1955 | 0 . 15496 |
| ATP6V1G2 | 27 .57689731 | -0 .320133611 | 0 .20746 | -1 .5431 | 0 . 12281 | 0 . 1587 |
| ATP6V1G3 | 3 .689878584 | -4 .980470738 | 0 .86328 | -5 .7693 | 7 .96E-09 | 3 .22E-08 |
| ATP6V1H | 4760 .313034 | 0 .630251623 | 0 . 10166 | 6 . 19971 | 5 .66E-10 | 2 .65E-09 |
| ATP7A | 8469 .868205 | -0 .612714093 | 0 . 1 1 1 1 1 | -5 .5146 | 3 .50E-08 | 1 .29E-07 |
| ATP7B | 629 .2107705 | -0 . 103951975 | 0 . 16367 | -0 .6351 | 0 .52534 | 0 .57941 |
| ATP8A1 | 7579 .527685 | -2 .298525454 | 0 .2009 | -1 1 .441 | 2 .60E-30 | 1 . 15E-28 |
| ATP8A2 | 646 .5124315 | 0 .026119765 | 0 .26188 | 0 .09974 | 0 .92055 | 0 .93399 |
| ATP8B1 | 2514 .512271 | 0 .051382953 | 0 . 13822 | 0 .37176 | 0 .71007 | 0 .75107 |
| ATP8B2 | 7610 .64822 | -0 . 180676627 | 0 . 12857 | -1 .4053 | 0 . 15994 | 0 .20166 |
| ATP8B3 | 137 .8131972 | 1 .366649373 | 0 . 17971 | 7 .60486 | 2 .85E-14 | 2 .25E-13 |
| ATP8B4 | 1459 .939502 | 0 .621801848 | 0 . 14937 | 4 . 16292 | 3 . 14E-05 | 7 .50E-05 |
| ATP9A | 3743 .662188 | 0 .564107105 | 0 . 1621 | 3 .48007 | 0 .0005 | 0 .00099 |
| ATP9B | 4119 .580582 | -0 .329213485 | 0 .07494 | -4 .3933 | 1 . 12E-05 | 2 .85E-05 |
| ATPAF1 | 1784 .555391 | 0 .272099551 | 0 .0729 | 3 .73235 | 0 .00019 | 0 .0004 |
| ATPAF2 | 1 103 .787741 | -0 .686883799 | 0 .0949 | -7 .2379 | 4 .56E-13 | 3 . 12E-12 |
| ATPIF1 | 1527 .941772 | -0 . 151912412 | 0 . 14241 | -1 .0667 | 0 .28609 | 0 .33921 |
| ATR | 6569 .527481 | -0 .098548243 | 0 .08468 | -1 . 1637 | 0 .24453 | 0 .29499 |
| ATRAID | 4999 .296601 | -0 .23257508 | 0 . 1277 | -1 .8212 | 0 .06857 | 0 .09338 |
| ATRIP | 561 .4534685 | -0 .067705381 | 0 .06469 | -1 .0466 | 0 .29528 | 0 .34884 |
| ATRN | 9276 .770762 | 0 .420152274 | 0 .07611 | 5 .52025 | 3 .39E-08 | 1 .26E-07 |
| ATRNL1 | 3052 .470775 | -3 .440867204 | 0 .25914 | -13 .278 | 3 . 10E-40 | 3 .72E-38 |
| ATRX | 1 1798 .54964 | -0 .275304701 | 0 .08803 | -3 . 1273 | 0 .00176 | 0 .00319 |
| ATXN1 | 8475 .237279 | 0 . 1 17482142 | 0 .08018 | 1 .46522 | 0 . 14286 | 0 . 18195 |
| ATXN10 | 4986 .35156 | -0 .490603568 | 0 .07151 | -6 .8602 | 6 .88E-12 | 4 .09E-1 1 |
| ATXN1L | 4092 .617356 | 0 .460229464 | 0 . 1 1203 | 4 . 10812 | 3 .99E-05 | 9 .36E-05 |
| ATXN2 | 1690 . 161738 | -0 . 190003953 | 0 . 10468 | -1 .815 | 0 .06952 | 0 .09453 |
| ATXN2L | 1868 .330296 | 0 .862215604 | 0 . 12984 | 6 .64045 | 3 . 13E-1 1 | 1 .72E-10 |
| ATXN3 | 4569 .789463 | -0 .482305481 | 0 .09346 | -5 . 1603 | 2 .47E-07 | 8 .06E-07 |
| ATXN3L | 3 .809113322 | -1 .078948267 | 0 .93702 | -1 . 1515 | 0 .24954 | 0 .30041 |
| ATXN7 | 3166 .814284 | -0 .237127396 | 0 . 1028 | -2 .3068 | 0 .02107 | 0 .0317 |

| ATXN7L1 ATXN7L2 ATXN7L3 ATXN7L3B AUH AUNIP AUP1 AURKA AURKAIP1 AURKB AURKC AUTS2 AVEN AVIL AVL9  AVP AVPI1 AVPR1A AVPR1B AVPR2 AWAT1 AWAT2 AXDND1 AXIN1 AXIN2 AXL AZGP1 AZI2 AZIN1 AZIN1-AS1 AZIN2 AZU1  B2M B3GALNT1 B3GALNT2 B3GALT1 B3GALT2 B3GALT4 B3GALT5 | 879 . 1 107545 190 .5171636 2542 . 126133 8643 .226443 1361 .551862 218 .9627323 3924 .676312 903 .7022369 1968 .949537 400 .755403 57 .3664331 2278 .856215 797 .3678986 564 .4632068 5808 .579601 0 .716972237 1312 .360238 334 .8640907 4 .553190015 1 12 .5716424 623 .5728957 1396 .23439 122 .9043199 1799 .00663 1231 .879715 4035 .733542 7492 . 153753 2392 .992531 15090 .7287 282 .0240662 485 .0230483 5 .333315719 281001 .4144 1570 .856725 1481 .644015 281 .2586945 506 . 1 1 16855 869 .4881376 171 .4230435 |
| --- | --- |

B3GALT5-AS13 .58146339

| B3GALT6 B3GAT1 B3GAT2 B3GAT3 B3GLCT B3GNT2 B3GNT3 B3GNT4 B3GNT5 B3GNT6 B3GNT7 B3GNT8 B3GNT9 B3GNTL1 B4GALNT1 B4GALNT2 | 1208 .321938 145 .987921 164 .4920175 1239 .022693 3036 .406403 4181 .827202 109 .7940015 93 .70340605 1202 . 134548 3 .609857247 499 .7013421 80 .37962449 549 .5237815 475 .5381568 94 .91813077 55 .75288864 |
| --- | --- |

-0 . 167374466

0 .069856719

0 .817468143

-0 .219722688

-0 .219167377

1 .422265551

0 .522562876

2 .018390099

0 .572032731

1 .776767296

0 . 130210081

-0 .759312376

0 .27072433

-0 .56874965

0 .204190693

0 .060080771

-0 . 185462371

1 .25296552

-0 .902090853

-0 . 161156898

1 .665700295

1 .70633373

-0 .060984976

0 .906410139

-0 .289661352

1 .0018309

-0 .928534331

-0 .430868384

0 .286586691

-0 .706721175

0 .345681281

-0 . 153875439

0 .699178002

-0 .47214315

0 .265848516

1 .578812324

-0 .424824469

0 .276362804

1 . 15224977

0 .492643603

0 .49850782

0 . 1 16415081

-0 .74764245

0 .264518859

0 .620649572

0 .600113836

0 .780767324

-0 .519068671

-0 . 172490514

-2 .424185387

1 .518881835

0 . 1 17156885

1 .003521171

-0 .469570518

-0 .019204623

-1 .379478987

0 . 1 1016 0 . 1 1318 0 .06031 0 .06955 0 .08986 0 . 18213 0 .09098 0 .205 0 . 13491 0 .20148 0 . 14812 0 . 14379 0 . 10473 0 . 1 1478 0 .07504 0 .73388 0 . 14978 0 . 18205 0 .45649 0 . 16043 0 .62833 0 .72952 0 .39933 0 .081 0 . 18493 0 . 1436 0 .22492 0 .06232 0 . 10787 0 . 13079 0 . 10909 0 .399 0 . 18819 0 . 15483 0 .08407 0 .30444 0 . 14073 0 .09539 0 .35075 0 .46588 0 . 10521 0 .32708 0 . 14202 0 . 12 0 . 15108 0 . 12672 0 .29064 0 . 13564 0 . 12363 0 .59924 0 . 17072 0 . 16086 0 . 13922 0 . 10536 0 .26222 0 .40375

-1 .5194 0 .61723 13 .5549 -3 . 1591 -2 .4391 7 .80915 5 .74383 9 .84558 4 .24023 8 .81837 0 .87908 -5 .2807 2 .58503 -4 .9553 2 .72095 0 .08187 -1 .2382 6 .88252 -1 .9762 -1 .0045 2 .65097 2 .33899 -0 . 1527 1 1 . 1899 -1 .5664 6 .97678 -4 . 1283 -6 .9142 2 .65675 -5 .4035 3 . 16882 -0 .3857 3 .71527 -3 .0495 3 . 16226 5 . 18597 -3 .0187 2 .89725 3 .28511 1 .05746 4 .73833 0 .35592 -5 .2645 2 .20437 4 . 10814 4 .73572 2 .6864 -3 .8269 -1 .3952 -4 .0454 8 .89671 0 .72832 7 .20829 -4 .4566 -0 .0732 -3 .4166

0 . 12866 0 .53708 7 .41E-42 0 .00158 0 .01472 5 .76E-15 9 .26E-09 7 . 16E-23 2 .23E-05 1 . 16E-18 0 .37936 1 .29E-07 0 .00974 7 .22E-07 0 .00651 0 .93475 0 .21564 5 .88E-12 0 .04814 0 .31514 0 .00803 0 .01934 0 .87862 4 .57E-29 0 . 1 1727 3 .02E-12 3 .65E-05 4 .70E-12 0 .00789 6 .54E-08 0 .00153 0 .69975 0 .0002 0 .00229 0 .00157 2 . 15E-07 0 .00254 0 .00376 0 .00102 0 .2903 2 . 15E-06 0 .7219 1 .41E-07 0 .0275 3 .99E-05 2 . 18E-06 0 .00722 0 .00013 0 . 16295 5 .22E-05 5 .75E-19 0 .46642 5 .67E-13 8 .33E-06 0 .94162 0 .00063

0 . 16558 0 .59056 1 .02E-39 0 .00289 0 .02271 4 .88E-14 3 .71E-08 1 .49E-21 5 .46E-05 1 .53E-17 0 .43555 4 .40E-07 0 .01552 2 .20E-06 0 .01073 0 .9455 0 .26354 3 .53E-1 1 0 .06761 0 .36956 0 .013 0 .02927 0 .89824 1 .83E-27 0 . 15226 1 .89E-1 1 8 .62E-05 2 .86E-1 1 0 .0128 2 .33E-07 0 .0028 0 .7415 0 .00043 0 .00408 0 .00286 7 .09E-07 0 .00449 0 .00647 0 .00192 0 .34359 6 .09E-06 0 .76152 4 .77E-07 0 .04048 9 .36E-05 6 . 16E-06 0 .01181 0 .00028 0 .20514 0 .00012 7 .83E-18 0 .5222 3 .84E-12 2 . 16E-05 0 .95119 0 .00123

| B4GALNT3 | 1 171 .777981 | 0 .661193262 | 0 . 16994 | 3 .89079 | 9 .99E-05 | 0 .00022 |
| --- | --- | --- | --- | --- | --- | --- |
| B4GALNT4 | 463 .5112751 | 0 .408477201 | 0 .27923 | 1 .46289 | 0 . 1435 | 0 . 18265 |
| B4GALT1 | 4547 .614717 | 1 .456973629 | 0 .09897 | 14 .7212 | 4 .71E-49 | 1 .27E-46 |
| B4GALT2 | 2232 .066362 | 0 .721935204 | 0 . 12186 | 5 .92453 | 3 . 13E-09 | 1 .33E-08 |
| B4GALT3 | 1212 .415796 | -0 . 198636948 | 0 . 10073 | -1 .9721 | 0 .0486 | 0 .06818 |
| B4GALT4 | 1324 .008223 | -0 .331241322 | 0 .08329 | -3 .9771 | 6 .98E-05 | 0 .00016 |
| B4GALT5 | 5155 .251172 | 1 .502528349 | 0 . 16058 | 9 .35701 | 8 .20E-21 | 1 .37E-19 |
| B4GALT6 | 2365 .669815 | -0 .351289643 | 0 .23565 | -1 .4907 | 0 . 13603 | 0 . 17414 |
| B4GALT7 | 1242 .903968 | 0 .484021028 | 0 .08831 | 5 .481 | 4 .23E-08 | 1 .54E-07 |
| B4GAT1 | 1941 .327624 | -0 .063757051 | 0 . 13526 | -0 .4714 | 0 .63738 | 0 .68475 |
| B9D1 | 438 .704313 | -0 .411 191131 | 0 . 10292 | -3 .9953 | 6 .46E-05 | 0 .00015 |
| B9D2 | 156 .2222888 | 0 . 195998483 | 0 . 12418 | 1 .57838 | 0 . 1 1448 | 0 . 14903 |
| BAALC | 1880 . 17788 | 0 .572116277 | 0 .34443 | 1 .66107 | 0 .0967 | 0 . 12776 |
| BAALC-AS2 | 37 .27418161 | -0 .28751022 | 0 .26869 | -1 .0701 | 0 .2846 | 0 .33756 |
| BAAT | 87 .87776592 | 1 .9944205 | 0 .51699 | 3 .85773 | 0 .00011 | 0 .00025 |
| BABAM1 | 1 188 .392016 | 0 .347384446 | 0 . 10763 | 3 .22765 | 0 .00125 | 0 .00232 |
| BACE1 | 2540 .026233 | 0 . 15361387 | 0 . 10017 | 1 .53354 | 0 . 12514 | 0 . 16145 |
| BACE2 | 14353 .55268 | 0 . 184354339 | 0 . 16826 | 1 .09566 | 0 .27323 | 0 .32547 |
| BACH1 | 1 1497 .93486 | -0 .503399093 | 0 .08356 | -6 .0246 | 1 .69E-09 | 7 .46E-09 |
| BACH2 | 997 .4241647 | -0 .540805012 | 0 . 15044 | -3 .5949 | 0 .00032 | 0 .00066 |
| BAD | 1 167 .713498 | -0 . 151953158 | 0 . 12037 | -1 .2624 | 0 .20681 | 0 .25396 |
| BAG1 | 3536 .916167 | -0 .243871764 | 0 . 12367 | -1 .972 | 0 .04861 | 0 .06819 |
| BAG2 | 985 .4321521 | 0 .722688819 | 0 . 14399 | 5 .01914 | 5 . 19E-07 | 1 .61E-06 |
| BAG3 | 1515 .00453 | 0 .085880948 | 0 . 13782 | 0 .62316 | 0 .53318 | 0 .5868 |
| BAG4 | 4344 .72889 | -0 .416500092 | 0 . 10917 | -3 .815 | 0 .00014 | 0 .00029 |
| BAG5 | 4347 .936371 | -0 .044558883 | 0 .06719 | -0 .6632 | 0 .5072 | 0 .56176 |
| BAG6 | 5653 .83392 | 0 .743044325 | 0 . 10287 | 7 .22319 | 5 .08E-13 | 3 .46E-12 |
| BAHD1 | 1027 .882176 | 0 .770290988 | 0 .0913 | 8 .43682 | 3 .26E-17 | 3 .62E-16 |
| BAIAP2 | 2348 .261622 | -0 .386573464 | 0 . 10973 | -3 .5229 | 0 .00043 | 0 .00085 |
| BAIAP2L1 | 902 . 1951616 | -0 .311 185763 | 0 . 14084 | -2 .2095 | 0 .02714 | 0 .04002 |
| BAIAP2L2 | 153 .9285936 | 0 .897335615 | 0 . 19786 | 4 .53532 | 5 .75E-06 | 1 .53E-05 |
| BAIAP3 | 197 .5485579 | -1 .526188558 | 0 . 1718 | -8 .8834 | 6 .48E-19 | 8 .75E-18 |
| BAK1 | 1934 .223809 | 1 .006246678 | 0 . 12692 | 7 .92836 | 2 .22E-15 | 2 .00E-14 |
| BAMBI | 2325 .994891 | -0 . 170700862 | 0 .22427 | -0 .7611 | 0 .44657 | 0 .50241 |
| BANF1 | 705 .236755 | 0 . 143593206 | 0 . 16933 | 0 .84802 | 0 .39642 | 0 .45309 |
| BANF2 | 3 .511091753 | -2 .898916291 | 0 .6342 | -4 .571 | 4 .85E-06 | 1 .31E-05 |
| BANK1 | 318 . 1434259 | 0 .928335401 | 0 .27932 | 3 .32351 | 0 .00089 | 0 .00169 |
| BANP | 952 .9913171 | 0 .222347439 | 0 . 10041 | 2 .21445 | 0 .0268 | 0 .03957 |
| BAP1 | 5764 .319985 | -0 .008205133 | 0 .06823 | -0 . 1203 | 0 .90428 | 0 .9205 |
| BARD1 | 2393 .649299 | -0 .916296746 | 0 .08447 | -10 .848 | 2 .04E-27 | 6 .96E-26 |
| BARHL1 | 8 .385252435 | -0 .307532376 | 0 .28912 | -1 .0637 | 0 .28748 | 0 .34064 |
| BARHL2 | 3 .676490223 | -2 . 157116379 | 0 .72679 | -2 .968 | 0 .003 | 0 .00523 |
| BARX1 | 6 .210928903 | 0 .462248844 | 0 .54262 | 0 .85188 | 0 .39428 | 0 .45085 |
| BARX2 | 480 .7708053 | -0 .460277178 | 0 .29944 | -1 .5371 | 0 . 12426 | 0 . 16044 |
| BASP1 | 1 183 .746621 | 1 .775060657 | 0 . 14157 | 12 .5383 | 4 .61E-36 | 3 .68E-34 |
| BATF | 373 .8975547 | 1 .722136125 | 0 .20712 | 8 .31472 | 9 .20E-17 | 9 .73E-16 |
| BATF2 | 215 .4846469 | 1 .543661631 | 0 .24535 | 6 .29179 | 3 . 14E-10 | 1 .52E-09 |
| BATF3 | 143 .3952853 | 1 . 109967331 | 0 . 18343 | 6 .05112 | 1 .44E-09 | 6 .41E-09 |
| BAX | 1 139 .779498 | 0 .828005002 | 0 . 13304 | 6 .22392 | 4 .85E-10 | 2 .29E-09 |
| BAZ1A | 5634 .925563 | 0 .243338344 | 0 . 1005 | 2 .42123 | 0 .01547 | 0 .02376 |
| BAZ1B | 7141 .9911 1 | 0 .688754235 | 0 .09594 | 7 . 17904 | 7 .02E-13 | 4 .72E-12 |
| BAZ2A | 4801 .286549 | 0 .206294985 | 0 . 1 1241 | 1 .83517 | 0 .06648 | 0 .09078 |
| BAZ2B | 8522 .235404 | -0 .764068457 | 0 . 10861 | -7 .0351 | 1 .99E-12 | 1 .27E-1 1 |
| BBC3 | 665 .9087 | 1 .935591591 | 0 . 16196 | 1 1 .9513 | 6 .39E-33 | 3 .80E-31 |
| BBIP1 | 2221 .622773 | -0 .854687537 | 0 . 10525 | -8 . 1209 | 4 .63E-16 | 4 .53E-15 |
| BBOF1 | 192 .7126224 | -0 .446866095 | 0 . 10704 | -4 . 1747 | 2 .98E-05 | 7 . 15E-05 |

| BBOX1 BBS1 BBS10 BBS12 BBS2 BBS4 BBS5 BBS7 BBS9 BBX BCAM BCAN BCAP29 BCAP31 BCAR1 BCAR3 BCAS1 BCAS2 BCAS3 BCAS4 BCAT1 BCAT2 BCCIP BCDIN3D BCHE BCKDHA BCKDHB BCKDK BCL10 BCL11A BCL11B BCL2 BCL2A1 BCL2L1 BCL2L10 BCL2L11 BCL2L12 BCL2L13 BCL2L14 BCL2L15 BCL2L2 | 1905 .546256 70 .06490353 2567 .873338 938 .4720492 5996 .516184 1827 . 147527 449 .2871579 1574 .801847 2634 .960816 10115 .45 915 .6160579 8471 .228824 6583 .233235 8801 .47086 1974 .057622 1639 .569067 108 .666096 1894 .874954 2466 .207066 184 .0005137 2391 .492297 1223 .994557 1560 . 1 14169 222 .6626791 5419 .085907 1 12 .0063451 1707 .396473 1556 .358777 211 1 .780353 1836 .745816 3407 .299391 5287 .705377 2238 .243163 2281 .335034 142 .3664618 1484 .469897 85 .41680889 6792 .926963 105 .769422 65 .66641646 2965 .543104 |
| --- | --- |

BCL2L2-PAB19 .75583799

| BCL3  BCL6  BCL6B  BCL7A  BCL7B  BCL7C  BCL9  BCL9L  BCLAF1  BCO1  BCO2  BCOR  BCORL1  BCR | 282 . 1349151 2484 .328822 418 .9513249 864 .6471305 745 .2718572 1565 .599019 1070 .775024 1028 . 14509 19794 .22203 27 . 19469312 162 .3458854 2265 .605868 375 .4084067 3198 .008734 |
| --- | --- |

-1 .2061499

-0 .613469718

-0 .857840807

-0 .358911068

-1 .503719192

-0 .00138558

0 .88845769

-0 .583559341

-1 .007188566

-0 .430459602

1 . 10517808

-0 .708699754

0 . 1 1992396

0 .318570887

1 .501515954

-0 .004835777

-0 . 144227269

-0 .459303155

-0 .515860274

0 .286341044

1 . 199658704

0 .422597132

-0 .759680979

-0 . 1 17637996

-1 .517626809

0 .202653838

-0 . 173301564

0 . 164741882

0 . 109754909

-0 .536250078

-0 . 173226008

-0 .248666052

1 .526985168

0 .576747172

-1 .430903978

1 .513275554

0 .661100585

0 .077391881

0 .341966522

-0 .250804379

-0 .203045476

0 . 153135572

1 .422192252

-0 .449974064

1 . 1 17849283

0 .383879768

0 .261590348

-0 . 150804108

0 .054071465

0 .837900234

-0 .290408073

-2 .035837302

-0 .973032648

0 .461489164

0 .258649295

0 .28942566

0 .24883 0 . 14307 0 . 10565 0 . 14443 0 . 1 1593 0 .08746 0 . 1376 0 .09005 0 .09032 0 .08559 0 .2011 1 0 .45437 0 . 12816 0 .09868 0 . 13313 0 . 1 1582 0 .27125 0 .09422 0 .09269 0 .21169 0 .21137 0 .21434 0 .07707 0 . 10562 0 .22897 0 . 1 1883 0 . 1 1858 0 . 10603 0 .07561 0 .2461 0 .21809 0 . 1 1804 0 .33388 0 .08847 0 .27213 0 . 1 1024 0 . 14212 0 .07727 0 . 19067 0 .20005 0 .07689 0 .2321 0 . 16786 0 . 14363 0 . 15414 0 . 1 1426 0 . 1 1628 0 . 10596 0 . 1 1275 0 . 17843 0 .08971 0 .28342 0 . 17331 0 .09957 0 . 14172 0 . 10543

-4 .8474 -4 .288 -8 . 1 199 -2 .485 -12 .971 -0 .0158 6 .45681 -6 .4807 -1 1 . 152 -5 .0293 5 .49531 -1 .5597 0 .93575 3 .22821 1 1 .2787 -0 .0418 -0 .5317 -4 .8748 -5 .5657 1 .35262 5 .67559 1 .97166 -9 .8567 -1 . 1 138 -6 .6281 1 .70542 -1 .4615 1 .5537 1 .45157 -2 . 179 -0 .7943 -2 . 1066 4 .5734 6 .51877 -5 .2582 13 .7271 4 .6517 1 .00153 1 .79348 -1 .2537 -2 .6407 0 .65978 8 .47271 -3 . 1328 7 .25238 3 .35957 2 .2496 -1 .4232 0 .47956 4 .69588 -3 .2373 -7 . 1832 -5 .6144 4 .63468 1 .82506 2 .74514

1 .25E-06 1 .80E-05 4 .67E-16 0 .01296 1 .78E-38 0 .98736 1 .07E-10 9 . 13E-1 1 7 .02E-29 4 .92E-07 3 .90E-08 0 . 1 1882 0 .3494 0 .00125 1 .67E-29 0 .9667 0 .59493 1 .09E-06 2 .61E-08 0 . 17618 1 .38E-08 0 .04865 6 .41E-23 0 .26538 3 .40E-1 1 0 .08812 0 . 14388 0 . 12026 0 . 14662 0 .02933 0 .42703 0 .03515 4 .80E-06 7 .09E-1 1 1 .45E-07 6 .98E-43 3 .29E-06 0 .31657 0 .0729 0 .20995 0 .00827 0 .50939 2 .40E-17 0 .00173 4 . 10E-13 0 .00078 0 .02447 0 . 15466 0 .63154 2 .65E-06 0 .00121 6 .81E-13 1 .97E-08 3 .57E-06 0 .06799 0 .00605

3 .67E-06 4 .48E-05 4 .55E-15 0 .02019 1 .77E-36 0 .98937 5 .47E-10 4 .71E-10 2 .76E-27 1 .54E-06 1 .43E-07 0 . 15412 0 .40489 0 .00232 6 .98E-28 0 .97173 0 .64561 3 .23E-06 9 .86E-08 0 .22026 5 .41E-08 0 .06823 1 .34E-21 0 .31704 1 .87E-10 0 . 1 1756 0 . 18311 0 . 15573 0 . 18632 0 .04294 0 .48354 0 .05063 1 .29E-05 3 .71E-10 4 .92E-07 1 .08E-40 9 .06E-06 0 .37108 0 .0988 0 .25738 0 .01337 0 .56379 2 .71E-16 0 .00314 2 .82E-12 0 .0015 0 .03639 0 . 19559 0 .67954 7 .41E-06 0 .00225 4 .58E-12 7 .56E-08 9 .79E-06 0 .09266 0 .01003

| BCS1L | 816 .643059 | -0 .344758745 | 0 . 10523 | -3 .2763 | 0 .00105 | 0 .00198 |
| --- | --- | --- | --- | --- | --- | --- |
| BDH1 | 1788 .756245 | 0 .076815493 | 0 . 14387 | 0 .53393 | 0 .59339 | 0 .64409 |
| BDH2 | 2060 . 123325 | -0 .764910196 | 0 . 1 1745 | -6 .5126 | 7 .38E-1 1 | 3 .85E-10 |
| BDKRB1 | 34 .66806709 | -1 .045107389 | 0 .21862 | -4 .7805 | 1 .75E-06 | 5 .01E-06 |
| BDKRB2 | 659 .7326779 | 0 .276609436 | 0 . 15747 | 1 .75658 | 0 .07899 | 0 . 1063 |
| BDNF | 472 .3792635 | -1 .995047666 | 0 . 19939 | -10 .006 | 1 .43E-23 | 3 .20E-22 |
| BDP1 | 9752 .580758 | -0 .496965339 | 0 .0926 | -5 .3669 | 8 .01E-08 | 2 .82E-07 |
| BEAN1 | 294 .7743319 | 1 . 180478983 | 0 .23976 | 4 .92369 | 8 .49E-07 | 2 .56E-06 |
| BECN1 | 3203 .646439 | -0 .331755007 | 0 .09618 | -3 .4495 | 0 .00056 | 0 .0011 |
| BEGAIN | 144 . 1234758 | -0 .980768199 | 0 .21386 | -4 .586 | 4 .52E-06 | 1 .22E-05 |
| BEND2 | 13 .4182757 | -5 .643660996 | 0 .77137 | -7 .3164 | 2 .55E-13 | 1 .79E-12 |
| BEND3 | 425 .5855497 | 0 .805283597 | 0 . 1276 | 6 .311 12 | 2 .77E-10 | 1 .35E-09 |
| BEND4 | 34 .68016484 | -1 . 120590721 | 0 .46294 | -2 .4206 | 0 .01549 | 0 .02379 |
| BEND5 | 300 . 1463491 | -1 .040396979 | 0 . 1381 | -7 .5336 | 4 .93E-14 | 3 .78E-13 |
| BEND6 | 283 .6504848 | 0 .028812433 | 0 . 15068 | 0 . 19122 | 0 .84835 | 0 .87204 |
| BEND7 | 339 . 1 1 18022 | -0 . 148596781 | 0 . 1632 | -0 .9105 | 0 .36253 | 0 .41821 |
| BEST1 | 1206 .789036 | 0 .609003657 | 0 . 19173 | 3 . 17644 | 0 .00149 | 0 .00273 |
| BEST2 | 37 .29840193 | -1 .731480828 | 0 .30788 | -5 .6238 | 1 .87E-08 | 7 . 19E-08 |
| BEST3 | 31 .8552572 | -1 .783343959 | 0 .38811 | -4 .595 | 4 .33E-06 | 1 . 17E-05 |
| BEST4 | 35 .29348483 | -0 .675238319 | 0 . 16837 | -4 .0105 | 6 .06E-05 | 0 .00014 |
| BET1 | 2148 .595978 | -0 . 147462151 | 0 . 1422 | -1 .037 | 0 .29975 | 0 .35337 |
| BET1L | 1300 .706606 | 0 . 149602549 | 0 .0751 | 1 .99198 | 0 .04637 | 0 .06535 |
| BEX1 | 30 .04369182 | -0 .2301712 | 0 .34566 | -0 .6659 | 0 .50548 | 0 .56014 |
| BEX2 | 466 .6840213 | -0 .833462149 | 0 . 17243 | -4 .8337 | 1 .34E-06 | 3 .91E-06 |
| BEX3 | 4196 .359924 | 0 .238053848 | 0 . 15874 | 1 .49965 | 0 . 1337 | 0 . 17137 |
| BEX4 | 1643 .037112 | -0 . 177454416 | 0 . 14206 | -1 .2492 | 0 .2116 | 0 .25918 |
| BEX5 | 89 .93714476 | -1 .704903259 | 0 .27276 | -6 .2506 | 4 .09E-10 | 1 .95E-09 |
| BFAR | 5520 .719092 | 0 . 1 1751235 | 0 . 12864 | 0 .91353 | 0 .36096 | 0 .41673 |
| BFSP1 | 308 . 1667782 | -0 .794225115 | 0 .23199 | -3 .4236 | 0 .00062 | 0 .0012 |
| BFSP2 | 31 .93376589 | 0 . 1 15030158 | 0 .34874 | 0 .32985 | 0 .74152 | 0 .77823 |
| BGLAP | 14 .32990742 | -0 .583827408 | 0 .20579 | -2 .837 | 0 .00455 | 0 .00771 |
| BGN | 1655 .407275 | 1 .554855205 | 0 . 19395 | 8 .01662 | 1 .09E-15 | 1 .00E-14 |
| BHLHA15 | 35 .58263811 | 3 .304059012 | 0 .43984 | 7 .51193 | 5 .83E-14 | 4 .41E-13 |
| BHLHA9 | 0 .852022805 | -0 .550958493 | 0 .84495 | -0 .6521 | 0 .51436 | 0 .56864 |
| BHLHB9 | 546 .4308411 | -0 .701778426 | 0 . 13058 | -5 .3745 | 7 .68E-08 | 2 .71E-07 |
| BHLHE22 | 157 .9046454 | 1 .259526229 | 0 .25096 | 5 .01882 | 5 .20E-07 | 1 .62E-06 |
| BHLHE23 | 0 .79270193 | 0 .044392496 | 1 .3035 | 0 .03406 | 0 .97283 | 0 .97686 |
| BHLHE40 | 3617 . 159793 | 0 .386435135 | 0 . 14463 | 2 .67194 | 0 .00754 | 0 .01228 |
| BHLHE41 | 1474 .930776 | 1 . 198593883 | 0 .21263 | 5 .637 | 1 .73E-08 | 6 .69E-08 |
| BHMG1 | 5 .729882037 | -2 .290316305 | 0 .39323 | -5 .8244 | 5 .73E-09 | 2 .37E-08 |
| BHMT | 35 .72968385 | -1 . 133903746 | 0 .25859 | -4 .385 | 1 . 16E-05 | 2 .95E-05 |
| BHMT2 | 207 .0725085 | 0 .225632781 | 0 . 19745 | 1 . 14275 | 0 .25314 | 0 .30407 |
| BICC1 | 3531 .935621 | 0 .551159685 | 0 . 17074 | 3 .22806 | 0 .00125 | 0 .00232 |
| BICD1 | 2542 .694956 | -0 .532856237 | 0 . 16651 | -3 .2002 | 0 .00137 | 0 .00254 |
| BICD2 | 10396 .75521 | -0 . 155731275 | 0 . 13204 | -1 . 1794 | 0 .23823 | 0 .28822 |
| BICDL1 | 1363 .647019 | -0 .938027805 | 0 . 19881 | -4 .7182 | 2 .38E-06 | 6 .68E-06 |
| BICDL2 | 687 .2567607 | -0 .231313738 | 0 .22836 | -1 .0129 | 0 .311 1 | 0 .36554 |
| BID | 1856 . 168755 | 0 .816481926 | 0 . 10072 | 8 . 10639 | 5 .21E-16 | 5 .06E-15 |
| BIK | 191 .5953444 | 1 .324915991 | 0 . 1972 | 6 .7187 | 1 .83E-1 1 | 1 .04E-10 |
| BIN1 | 1057 .580463 | 0 .792041749 | 0 . 1 1847 | 6 .68582 | 2 .30E-1 1 | 1 .28E-10 |
| BIN2 | 514 . 1703115 | 1 .695521248 | 0 . 1939 | 8 .74432 | 2 .24E-18 | 2 .86E-17 |
| BIN3 | 1022 .481557 | 0 .68870158 | 0 . 16292 | 4 .22718 | 2 .37E-05 | 5 .76E-05 |
| BIRC2 | 7110 .065608 | -0 .235307992 | 0 .09719 | -2 .421 | 0 .01548 | 0 .02377 |
| BIRC3 | 4460 .092164 | 0 .975643924 | 0 .20744 | 4 .70318 | 2 .56E-06 | 7 . 16E-06 |
| BIRC5 | 475 .5235286 | 1 .751636206 | 0 .2161 | 8 . 10549 | 5 .25E-16 | 5 .09E-15 |
| BIRC6 | 31355 .31917 | -0 .363614977 | 0 .06994 | -5 . 1991 | 2 .00E-07 | 6 .62E-07 |

| BIRC7 | 3367 .099745 | 0 .41451121 | 0 .36491 | 1 . 13592 | 0 .25599 | 0 .30718 |
| --- | --- | --- | --- | --- | --- | --- |
| BIRC8 | 3 .214547977 | -5 .06476241 | 0 .98338 | -5 . 1503 | 2 .60E-07 | 8 .47E-07 |
| BIVM | 1442 .679424 | -0 .006719695 | 0 . 13929 | -0 .0482 | 0 .96152 | 0 .96758 |
| BLACE | 3 . 14556643 | -4 .007983094 | 0 .66563 | -6 .0213 | 1 .73E-09 | 7 .61E-09 |
| BLCAP | 4387 .566134 | 0 . 1 10118038 | 0 .08249 | 1 .3349 | 0 . 18191 | 0 .22662 |
| BLID | 45 .52877603 | -1 .762793572 | 0 .29354 | -6 .0052 | 1 .91E-09 | 8 .36E-09 |
| BLK | 74 .92218716 | 1 .771279864 | 0 .35856 | 4 .93998 | 7 .81E-07 | 2 .37E-06 |
| BLM | 906 .4778688 | 1 .530238651 | 0 . 15232 | 10 .0463 | 9 .54E-24 | 2 . 19E-22 |
| BLMH | 6317 .239053 | -1 .386810921 | 0 . 13942 | -9 .9471 | 2 .60E-23 | 5 .65E-22 |
| BLNK | 463 .736168 | 0 .434478075 | 0 . 14474 | 3 .00173 | 0 .00268 | 0 .00472 |
| BLOC1S1 | 1027 .917708 | -0 .372800498 | 0 . 13419 | -2 .7781 | 0 .00547 | 0 .00914 |
| BLOC1S2 | 3169 .663479 | -0 . 163600731 | 0 . 14633 | -1 . 1 18 | 0 .26355 | 0 .31519 |
| BLOC1S3 | 430 .8059033 | 0 .862544696 | 0 .08877 | 9 .71714 | 2 .55E-22 | 5 .00E-21 |
| BLOC1S4 | 717 .4982392 | 0 .695177973 | 0 . 10963 | 6 .34105 | 2 .28E-10 | 1 . 12E-09 |
| BLOC1S5 | 1347 .819034 | -0 .053415072 | 0 .09572 | -0 .5581 | 0 .57681 | 0 .6284 |
| BLOC1S6 | 7974 .004562 | 0 .349882265 | 0 . 12433 | 2 .81425 | 0 .00489 | 0 .00824 |
| BLVRA | 1978 .064757 | 0 . 162211 16 | 0 . 12136 | 1 .3366 | 0 . 18135 | 0 .22608 |
| BLVRB | 3397 .23977 | -0 . 198565044 | 0 . 13377 | -1 .4844 | 0 . 13771 | 0 . 17608 |
| BLZF1 | 3548 .255232 | -0 .545769409 | 0 .07726 | -7 .064 | 1 .62E-12 | 1 .05E-1 1 |
| BMF | 2164 .618862 | 0 .998295885 | 0 . 1389 | 7 . 1874 | 6 .60E-13 | 4 .45E-12 |
| BMI1 | 4126 .385142 | -0 .301911714 | 0 . 13582 | -2 .2229 | 0 .02623 | 0 .03881 |
| BMP1 | 2034 .691063 | 1 . 136091075 | 0 . 12066 | 9 .41594 | 4 .69E-21 | 8 .02E-20 |
| BMP10 | 4 .645912662 | -2 .556222729 | 0 .65713 | -3 .89 | 0 .0001 | 0 .00022 |
| BMP15 | 1 .378477256 | -3 .250082172 | 1 .0296 | -3 . 1567 | 0 .0016 | 0 .00291 |
| BMP2 | 648 .8693996 | -0 . 19604158 | 0 . 19505 | -1 .0051 | 0 .31486 | 0 .36928 |
| BMP2K | 3220 .855598 | 0 .433700399 | 0 .07863 | 5 .5154 | 3 .48E-08 | 1 .29E-07 |
| BMP3 | 74 .87497702 | -0 .494223003 | 0 .38315 | -1 .2899 | 0 . 19708 | 0 .24354 |
| BMP4 | 834 .3643568 | -1 .929687308 | 0 .22294 | -8 .6558 | 4 .89E-18 | 5 .98E-17 |
| BMP5 | 35 .08401827 | -0 .714634417 | 0 .42072 | -1 .6986 | 0 .0894 | 0 . 1 1911 |
| BMP6 | 210 .5728078 | 0 .690850217 | 0 . 18008 | 3 .83631 | 0 .00012 | 0 .00027 |
| BMP7 | 1 158 .76227 | -0 .352509144 | 0 .27544 | -1 .2798 | 0 .20061 | 0 .24733 |
| BMP8A | 30 .88911774 | 1 .276159277 | 0 . 16349 | 7 .80568 | 5 .92E-15 | 5 .01E-14 |
| BMP8B | 891 .3923785 | 2 . 190783378 | 0 .2336 | 9 .37828 | 6 .71E-21 | 1 . 13E-19 |
| BMPER | 80 .40876343 | -0 .641685407 | 0 .23732 | -2 .7039 | 0 .00685 | 0 .01124 |
| BMPR1A | 6849 .905336 | -0 .699434733 | 0 . 10488 | -6 .6691 | 2 .57E-1 1 | 1 .43E-10 |
| BMPR1B | 1215 .050344 | 0 .701514858 | 0 .28876 | 2 .42944 | 0 .01512 | 0 .02327 |
| BMPR2 | 16259 .90235 | 0 .783442851 | 0 . 1 1079 | 7 .0715 | 1 .53E-12 | 9 .94E-12 |
| BMS1 | 3852 .028667 | -0 .20980987 | 0 .08165 | -2 .5695 | 0 .01018 | 0 .01617 |
| BMT2 | 2396 .414778 | -0 .241308509 | 0 .09635 | -2 .5044 | 0 .01227 | 0 .01919 |
| BMX | 179 .4828812 | -0 .019275513 | 0 . 17643 | -0 . 1093 | 0 .913 | 0 .92775 |
| BNC1 | 1526 .221076 | -0 . 153640938 | 0 .25503 | -0 .6025 | 0 .54687 | 0 .59972 |
| BNC2 | 5189 .784527 | -0 .787126024 | 0 . 16035 | -4 .9089 | 9 . 16E-07 | 2 .75E-06 |
| BNIP1 | 515 .6013404 | -0 .203576613 | 0 . 1217 | -1 .6728 | 0 .09438 | 0 . 12502 |
| BNIP2 | 1 1401 .87884 | 0 . 163272409 | 0 .08783 | 1 .85887 | 0 .06305 | 0 .08644 |
| BNIP3 | 2720 . 146827 | -0 .340749482 | 0 . 14762 | -2 .3083 | 0 .02098 | 0 .03158 |
| BNIP3L | 15332 .7236 | 0 .031201993 | 0 . 1 1258 | 0 .27716 | 0 .78166 | 0 .81341 |
| BNIPL | 3216 . 165 | -1 .209368364 | 0 .23679 | -5 . 1073 | 3 .27E-07 | 1 .05E-06 |
| BOC | 3141 .964146 | -1 .301898611 | 0 . 18138 | -7 . 1776 | 7 .09E-13 | 4 .76E-12 |
| BOD1 | 2388 .000064 | -0 .45568682 | 0 . 10427 | -4 .3701 | 1 .24E-05 | 3 . 15E-05 |
| BOD1L1 | 9315 .368821 | 0 . 1 14157065 | 0 . 10482 | 1 .08908 | 0 .27612 | 0 .32871 |
| BOD1L2 | 7 .305934631 | -5 .54237302 | 0 .79224 | -6 .9958 | 2 .64E-12 | 1 .66E-1 1 |
| BOK | 1 142 . 141429 | 0 .829220463 | 0 . 18679 | 4 .43939 | 9 .02E-06 | 2 .33E-05 |
| BOLA1 | 756 .3258803 | -0 .4911 10605 | 0 . 1 1707 | -4 . 1949 | 2 .73E-05 | 6 .59E-05 |
| BOLA2B | 34 .22245558 | 0 .582391303 | 0 .20496 | 2 .84153 | 0 .00449 | 0 .00761 |
| BOLA3 | 784 .6480217 | 0 .461447367 | 0 .20954 | 2 .2022 | 0 .02765 | 0 .04068 |
| BOLL | 31 .24044727 | -0 .826729555 | 0 .28869 | -2 .8637 | 0 .00419 | 0 .00714 |

| BORA BORCS5 BORCS6 BORCS7 BORCS8 | 694 .4921763 863 .0874218 980 .2703858 2346 .990389 360 . 1419585 |
| --- | --- |

BORCS8-ME 3 .34712736

| BPGM  BPHL  BPI  BPIFA1  BPIFA2  BPIFA3  BPIFB1  BPIFB2  BPIFB3  BPIFB4  BPIFB6  BPIFC  BPNT1  BPTF  BPY2B  BRAF  BRAP  BRAT1  BRCA1  BRCA2  BRCC3  BRD1  BRD2  BRD3  BRD4  BRD7  BRD8  BRD9  BRDT  BRE  BRF1  BRF2  BRI3  BRI3BP  BRICD5  BRINP1  BRINP2  BRINP3  BRIP1  BRIX1  BRK1  BRMS1  BRMS1L  BROX  BRPF1  BRPF3  BRS3  BRSK1  BRSK2  BRWD1 | 1 182 .401037 1492 .326316 23 .84993886 2 .558855049 3 .737855907 4 .839459994 9 . 198598236 3 .763541604 4 .408916008 7 . 127321531 4 .976535707 843 .7783191 2534 .447766 12799 .49984 0 .068550461 5998 .253578 2596 .272923 2720 . 125293 1351 .543082 1956 .313826 1 138 .23612 2866 .370801 6841 . 172379 1 189 .491622 2520 . 196998 4295 .430866 2277 .651914 2522 .346725 17 .26626522 1246 .02725 1783 .856324 614 .259009 2327 .096591 4395 . 184377 101 .4704375 27 .61252207 13 .74919252 45 .4293278 1204 .613885 1894 .065461 6024 .618999 3124 . 165976 612 .2471035 6960 .411095 1732 .595385 1464 .914626 10 .9736225 158 .7015746 105 .2363379 17818 . 15381 |
| --- | --- |

0 . 175086629

0 .099374293

0 .332305236

-0 .641015512

0 .29896026

-0 . 16555311

0 . 156582358

-0 .21593187

-1 .024416801

-4 .655758523

-4 .253156947

-4 .257424258

-2 .577593118

-3 .67659914

-3 .962500141

-1 .655035949

-3 . 192245447

-1 .076387289

0 .304147733

-0 .36542553

-0 .083762893

-0 .527308911

-0 .081431312

0 .777105519

0 .533192117

0 .819942333

-0 .031015018

0 .666946101

0 . 196970562

0 .404364652

0 .719653089

0 .016378583

-0 . 149657465

0 .010333699

-4 .076638366

-0 .068508446

0 .42909194

-0 .338686754

0 .425156069

0 .971423786

0 .384156408

-0 .482913134

-1 .337859785

-0 .412454372

0 .702774941

-0 .354264613

-0 .56833571

0 .016962332

-0 .448800049

-0 . 15539423

0 .356336554

0 .328585016

-3 .686022427

2 .094625783

0 .669751213

-0 .591009858

0 .08999 0 .08001 0 . 10427 0 . 13432 0 .09331 0 .36285 0 . 12061 0 . 12129 0 .33133 0 .83329 0 .82489 0 .79622 0 .63215 0 .72762 0 .73581 0 .65202 0 .79481 0 .2735 0 . 1 1963 0 . 10934 3 .0711 1 0 .08135 0 .04854 0 .09034 0 . 1 1873 0 . 12128 0 .06346 0 .08913 0 .07245 0 . 1 1991 0 . 13379 0 .06819 0 .07668 0 .08887 0 .6397 0 .07561 0 .06287 0 . 1 1747 0 . 15056 0 .22916 0 . 12183 0 .2869 0 .46171 0 .57576 0 . 13653 0 . 10519 0 . 1258 0 . 10277 0 .07023 0 .07028 0 .08874 0 . 1 1665 0 .51611 0 .2711 1 0 .21717 0 .09804

1 .94573 1 .24206 3 . 18702 -4 .7722 3 .20396 -0 .4563 1 .29826 -1 .7802 -3 .0918 -5 .5872 -5 . 156 -5 .3471 -4 .0775 -5 .0529 -5 .3852 -2 .5383 -4 .0164 -3 .9356 2 .54244 -3 .3421 -0 .0273 -6 .4817 -1 .6777 8 .6021 4 .49081 6 .76099 -0 .4887 7 .48307 2 .71859 3 .37222 5 .3788 0 .24018 -1 .9517 0 . 1 1627 -6 .3727 -0 .9061 6 .82513 -2 .8832 2 .8239 4 .23902 3 . 15317 -1 .6832 -2 .8976 -0 .7164 5 . 14745 -3 .3678 -4 .5178 0 . 16505 -6 .3905 -2 .211 4 .01571 2 .81688 -7 . 142 7 .726 3 .08401 -6 .028

0 .05169 0 .21421 0 .00144 1 .82E-06 0 .00136 0 .64821 0 . 1942 0 .07504 0 .00199 2 .31E-08 2 .52E-07 8 .94E-08 4 .55E-05 4 .35E-07 7 .24E-08 0 .011 14 5 .91E-05 8 .30E-05 0 .01101 0 .00083 0 .97824 9 .07E-1 1 0 .09341 7 .83E-18 7 . 10E-06 1 .37E-1 1 0 .62502 7 .26E-14 0 .00656 0 .00075 7 .50E-08 0 .81019 0 .05098 0 .90744 1 .86E-10 0 .36491 8 .78E-12 0 .00394 0 .00474 2 .24E-05 0 .00162 0 .09234 0 .00376 0 .47377 2 .64E-07 0 .00076 6 .25E-06 0 .8689 1 .65E-10 0 .02704 5 .93E-05 0 .00485 9 .20E-13 1 . 1 1E-14 0 .00204 1 .66E-09

0 .07213 0 .26205 0 .00264 5 .21E-06 0 .00251 0 .69459 0 .24036 0 . 10151 0 .00357 8 .78E-08 8 .23E-07 3 . 13E-07 0 .00011 1 .37E-06 2 .56E-07 0 .01755 0 .00014 0 .00019 0 .01736 0 .00159 0 .98136 4 .68E-10 0 . 12387 9 .33E-17 1 .86E-05 7 .85E-1 1 0 .67372 5 .42E-13 0 .0108 0 .00144 2 .65E-07 0 .83868 0 .07122 0 .92302 9 .25E-10 0 .4206 5 . 14E-1 1 0 .00675 0 .00801 5 .48E-05 0 .00294 0 . 12264 0 .00647 0 .52928 8 .59E-07 0 .00146 1 .65E-05 0 .88973 8 .28E-10 0 .03989 0 .00014 0 .00818 6 . 10E-12 9 . 10E-14 0 .00366 7 .32E-09

| BRWD3  BSCL2  BSDC1  BSG  BSN  BSND  BSPH1  BSPRY  BST1  BST2  BSX  BTAF1  BTBD1  BTBD10  BTBD11  BTBD16  BTBD17  BTBD18  BTBD19  BTBD2  BTBD3  BTBD6  BTBD7  BTBD8  BTBD9  BTC  BTD  BTF3  BTF3L4  BTG1  BTG2  BTG3  BTG4  BTK  BTLA  BTN1A1  BTN2A1  BTN2A2  BTN3A1  BTN3A2  BTN3A3  BTNL2  BTNL3  BTNL8  BTNL9  BTRC  BUB1  BUB1B | 5545 .319653 121 .8683118 4432 .833408 13297 . 19832 174 .7313776 5 .720533579 3 .402040901 746 .406839 887 . 1 1 1 1928 2167 .93871 1 .467221099 10943 .90894 8746 .055775 3502 .825072 1054 .545385 268 .9904495 4 .254082133 198 .8248388 410 .8182293 2086 .017172 6657 .888389 2444 . 1 10609 4330 .463581 135 .3142913 2967 .09595 1376 .314565 2094 .835905 18552 .21251 4829 .201004 15858 .77771 2996 .042778 2576 .957399 10 .28721556 787 .4161054 273 .5496864 25 .51989417 1818 .481341 3053 . 1 15075 3690 .693719 3376 .574737 1845 .325385 13 .80100515 9 .055975529 17 .20241501 284 .3372531 2296 .783117 1371 .299802 779 .6294123 |
| --- | --- |

BUB1B-PAK62 .643255666

6683 .747353 1 1 12 .428266 1233 .492446 799 .9252563

BUB3 BUD13 BUD31 BVES

BX255923 .2 563 .4315155

BYSL

1082 .524 12450 .31601

BZW1

-0 .493379727

-0 . 165950658

-0 .049442866

0 .826183243

-0 . 17213371

-2 .632223162

-3 .427838802

-0 .907566749

-0 . 1 15950627

1 .202911 105

-3 .629746744

-0 .780129887

0 .563786296

-0 .080643918

-0 . 170950864

-2 .827615667

0 .966523542

-0 .68802514

0 .426868641

0 . 194876932

-0 .599806455

0 .39629866

-0 . 171424336

-0 .700191751

0 .285188335

-2 .657946681

-0 . 170343568

-1 .204555051

-0 . 135422323

-0 .856520213

0 .62743638

-0 .015553055

-2 . 1 17198849

0 .777510197

1 .752624478

-1 .266835073

0 .210251882

-0 . 142054865

0 .750488643

0 .712006646

0 .776094694

-1 .412406016

-1 .971211717

-0 .63746432

1 . 1 13966084

-0 .577792512

1 .839016168

1 .622276494

-1 .35660557

-0 . 161300061

-0 .283178725

0 .072293325

0 .259842606

-0 .890271932

0 .775654266

0 .392630611

0 . 10046 -4 .9112

0 . 1317 -1 .2601

0 .05662 -0 .8733

0 . 1 1797 7 .00307

0 . 1694 -1 .0161

0 .53715 -4 .9003

0 .90723 -3 .7783

0 .21369 -4 .2472

0 . 18018 -0 .6435

0 .21704 5 .54243

1 . 1238 -3 .2299

0 . 1 1 129 -7 .0097

0 . 12308 4 .58054

0 . 10355 -0 .7788

0 .20554 -0 .8317

0 .27105 -10 .432

0 .41633 2 .32153

0 . 16435 -4 . 1864

0 . 1609 2 .65305

0 .08792 2 .21645

0 . 1 128 -5 .3173

0 .08727 4 .54082

0 .06937 -2 .471

0 . 16113 -4 .3455

0 .0804 3 .54722

0 .33733 -7 .8795

0 .0988 -1 .7241

0 . 16018 -7 .5198

0 .07785 -1 .7395

0 . 1 1233 -7 .625

0 . 16715 3 .75368

0 .08177 -0 . 1902

0 .47218 -4 .4838

0 . 1746 4 .4531

0 .26112 6 .71192

0 .22965 -5 .5164

0 .08334 2 .52292

0 . 15148 -0 .9378

0 . 15476 4 .8493

0 . 19705 3 .6133

0 . 15332 5 .06184

0 .26621 -5 .3055

0 .51037 -3 .8623

0 .38164 -1 .6703

0 .28425 3 .91897

0 .07423 -7 .7835

0 . 17603 10 .4471

0 . 1838 8 .82652

0 .40015 -3 .3903

0 .07311 -2 .2063

0 .07236 -3 .9132

0 . 16882 0 .42822

0 . 19224 1 .35163

0 . 13914 -6 .3983

0 . 13003 5 .96522

0 .09646 4 .07047

9 .05E-07 0 .20764 0 .38249 2 .50E-12 0 .30957 9 .57E-07 0 .00016 2 . 16E-05 0 .51989 2 .98E-08 0 .00124 2 .39E-12 4 .64E-06 0 .43608 0 .40557 1 .77E-25 0 .02026 2 .83E-05 0 .00798 0 .02666 1 .05E-07 5 .60E-06 0 .01347 1 .39E-05 0 .00039 3 .29E-15 0 .08468 5 .48E-14 0 .08195 2 .44E-14 0 .00017 0 .84914 7 .33E-06 8 .46E-06 1 .92E-1 1 3 .46E-08 0 .01164 0 .34836 1 .24E-06 0 .0003 4 . 15E-07 1 . 12E-07 0 .00011 0 .09485 8 .89E-05 7 .05E-15 1 .51E-25 1 .08E-18 0 .0007 0 .02736 9 . 1 1E-05 0 .66849 0 . 17649 1 .57E-10 2 .44E-09 4 .69E-05

2 .72E-06 0 .25484 0 .43871 1 .58E-1 1 0 .36392 2 .86E-06 0 .00034 5 .30E-05 0 .57403 1 . 12E-07 0 .00231 1 .51E-1 1 1 .25E-05 0 .49217 0 .46208 4 .92E-24 0 .03058 6 .81E-05 0 .01293 0 .03938 3 .65E-07 1 .49E-05 0 .02092 3 .50E-05 0 .00078 2 .89E-14 0 . 1 1331 4 . 17E-13 0 . 10996 1 .94E-13 0 .00037 0 .87266 1 .92E-05 2 . 19E-05 1 .08E-10 1 .28E-07 0 .01828 0 .40381 3 .64E-06 0 .00062 1 .31E-06 3 .88E-07 0 .00025 0 . 12559 0 .0002 5 .92E-14 4 .21E-24 1 .43E-17 0 .00135 0 .04033 0 .0002 0 .71298 0 .22059 7 .89E-10 1 .06E-08 0 .00011

| BZW2 | 5888 .319997 0 .228906716 | 0 . 16648 1 .37497 | 0 . 16914 | 0 .21233 |
| --- | --- | --- | --- | --- |
| C10orf10 | 1043 .834244 1 .707817725 | 0 . 18188 9 .3897 | 6 .02E-21 | 1 .02E-19 |
| C10orf105 | 26 .48336371 -0 .315336535 | 0 .24046 -1 .3114 | 0 . 18972 | 0 .23545 |
| C10orf107 | 139 .0049456 -2 .939286237 | 0 .34769 -8 .4538 | 2 .82E-17 | 3 . 16E-16 |
| C10orf1 1 | 1458 .010014 -1 .777398991 | 0 . 14997 -1 1 .852 | 2 . 10E-32 | 1 .20E-30 |
| C10orf1 1 1 | 12 .01514861 -0 .774034223 | 0 .31008 -2 .4962 | 0 .01255 | 0 .01961 |
| C10orf1 13 | 52 .91035289 -1 .338610459 | 0 .34234 -3 .9102 | 9 .22E-05 | 0 .0002 |
| C10orf120 | 2 .661137876 -4 .377452398 | 0 .81923 -5 .3434 | 9 . 12E-08 | 3 . 19E-07 |
| C10orf126 | 51 .91655439 1 .462833012 | 0 .76038 1 .92381 | 0 .05438 | 0 .07554 |
| C10orf128 | 663 .0312927 0 .521066735 | 0 . 14194 3 .67101 | 0 .00024 | 0 .0005 |
| C10orf2 | 577 .3617352 0 .326415848 | 0 . 1 1342 2 .87798 | 0 .004 | 0 .00685 |
| C10orf25 | 93 .45417671 0 .558152445 | 0 . 18102 3 .08342 | 0 .00205 | 0 .00367 |
| C10orf35 | 71 .23095436 -0 .797230026 | 0 . 18953 -4 .2063 | 2 .60E-05 | 6 .28E-05 |
| C10orf53 | 7 .32357511 -5 .356024705 | 0 .8317 -6 .4399 | 1 .20E-10 | 6 .09E-10 |
| C10orf54 | 2713 .748218 0 .453828534 | 0 . 12677 3 .57984 | 0 .00034 | 0 .0007 |
| C10orf55 | 48 .45855562 0 .064011512 | 0 . 16615 0 .38527 | 0 .70004 | 0 .74176 |
| C10orf62 | 10 . 19095333 -0 .730558163 | 0 .38675 -1 .889 | 0 .0589 | 0 .08124 |
| C10orf67 | 66 .67817371 -2 .357036896 | 0 .20178 -1 1 .681 | 1 .59E-31 | 8 . 14E-30 |
| C10orf71 | 13 .58165654 -2 .762811833 | 0 .47518 -5 .8142 | 6 .09E-09 | 2 .51E-08 |
| C10orf76 | 1618 .641092 -0 . 164570955 | 0 .06977 -2 .3589 | 0 .01833 | 0 .02786 |
| C10orf82 | 26 .99131291 -1 .075230547 | 0 .2587 -4 . 1562 | 3 .24E-05 | 7 .71E-05 |
| C10orf88 | 718 .5333362 -0 .351082348 | 0 .06613 -5 .309 | 1 . 10E-07 | 3 .81E-07 |
| C10orf90 | 1001 . 165455 -0 .583028458 | 0 .28637 -2 .0359 | 0 .04176 | 0 .05934 |
| C10orf91 | 16 .91417311 -1 .497903426 | 0 .29512 -5 .0756 | 3 .86E-07 | 1 .23E-06 |
| C10orf95 | 16 .33324542 -0 .786170794 | 0 . 18128 -4 .3367 | 1 .45E-05 | 3 .64E-05 |
| C10orf99 | 462 .5525763 3 .008506908 | 0 .35735 8 .41897 | 3 .80E-17 | 4 . 18E-16 |
| C11orf1 | 896 .2044026 -1 .257483067 | 0 . 15568 -8 .0776 | 6 .61E-16 | 6 .28E-15 |
| C11orf16 | 25 .71606275 -0 .658781809 | 0 . 16728 -3 .9381 | 8 .21E-05 | 0 .00018 |
| C11orf21 | 201 .2835859 1 .226351366 | 0 .21439 5 .72007 | 1 .06E-08 | 4 .23E-08 |
| C11orf24 | 1509 .863148 0 .337492874 | 0 . 14997 2 .25044 | 0 .02442 | 0 .03632 |
| C11orf31 | 2673 .916778 -0 . 10937851 | 0 . 15228 -0 .7183 | 0 .47258 | 0 .52813 |
| C11orf40 | 2 .288119095 -4 .65266474 | 0 .91308 -5 .0956 | 3 .48E-07 | 1 . 1 1E-06 |
| C11orf42 | 46 .67723721 -1 .360735376 | 0 . 16895 -8 .0541 | 8 .01E-16 | 7 .50E-15 |
| C11orf44 | 10 .20470049 -2 .803653968 | 0 .51412 -5 .4533 | 4 .94E-08 | 1 .79E-07 |
| C11orf45 | 303 .3652181 -0 .93553073 | 0 . 14205 -6 .5858 | 4 .52E-1 1 | 2 .44E-10 |
| C11orf49 | 746 .4789256 -0 .522394465 | 0 .09989 -5 .2297 | 1 .70E-07 | 5 .67E-07 |
| C11orf52 | 5 .211047158 -1 .975762028 | 0 .32002 -6 . 1738 | 6 .67E-10 | 3 . 10E-09 |
| C11orf53 | 3 .431676745 -3 .348820584 | 0 .71609 -4 .6765 | 2 .92E-06 | 8 . 10E-06 |
| C11orf54 | 2029 .90276 -0 .529165403 | 0 . 10308 -5 . 1337 | 2 .84E-07 | 9 .21E-07 |
| C11orf57 | 4095 . 19554 -0 .511531896 | 0 .0819 -6 .2457 | 4 .22E-10 | 2 .01E-09 |
| C11orf58 | 12953 .76365 -0 .241798052 | 0 .09377 -2 .5787 | 0 .00992 | 0 .01578 |
| C11orf63 | 410 .9803733 -0 .354184806 | 0 . 12725 -2 .7833 | 0 .00538 | 0 .00901 |
| C11orf65 | 258 . 136239 -1 .045787211 | 0 . 12868 -8 . 1272 | 4 .39E-16 | 4 .31E-15 |
| C11orf68 | 747 .3464423 0 .322585991 | 0 .09597 3 .36138 | 0 .00078 | 0 .00149 |
| C11orf70 | 91 .05580317 -0 .652008631 | 0 .20795 -3 . 1354 | 0 .00172 | 0 .00311 |
| C11orf71 | 23 .51141723 -0 .334734943 | 0 . 17283 -1 .9368 | 0 .05277 | 0 .07349 |
| C11orf72 | 7 .675745193 -1 .753011042 | 0 .49242 -3 .56 | 0 .00037 | 0 .00075 |
| C11orf74 | 736 .8609882 -1 .500363931 | 0 . 1487 -10 .09 | 6 . 14E-24 | 1 .44E-22 |
| C11orf80 | 920 .0273777 -0 .759413402 | 0 . 1 1689 -6 .4969 | 8 .20E-1 1 | 4 .25E-10 |
| C11orf84 | 379 .5071314 1 .085671753 | 0 . 12898 8 .41752 | 3 .85E-17 | 4 .23E-16 |
| C11orf86 | 1 .550718354 -2 .089481901 | 0 .84733 -2 .466 | 0 .01366 | 0 .0212 |
| C11orf87 | 554 .8828025 -2 .994088695 | 0 .30287 -9 .8857 | 4 .80E-23 | 1 .02E-21 |
| C11orf88 | 14 .96627351 -2 . 181196493 | 0 .422 -5 . 1687 | 2 .36E-07 | 7 .71E-07 |
| C11orf91 | 16 .99863673 -0 .406117455 | 0 .23432 -1 .7332 | 0 .08307 | 0 . 1 1 136 |
| C11orf94 | 13 .26731462 -0 . 136346943 | 0 .21122 -0 .6455 | 0 .5186 | 0 .57279 |
| C11orf96 | 735 .7835324 1 .824944774 | 0 .21968 8 .30722 | 9 .80E-17 | 1 .03E-15 |

1213 .233516 3368 .878852 2281 . 109881 78 .92070677 32 .30490005 742 .4427519 1 168 .44888 3243 .292441 21 .88574017 24 .72683063 128 .8639023 3330 .641027 183 . 1598905 994 .2426786 756 .0246192 12 . 12428292 398 . 1 160631 9 .2814836 1063 .045752 1326 .370149 10 .30012811 1411 .728661 38 . 18263503 2538 .525251 3034 .527226 839 .6745796 1906 .475122 5540 .419014 2 .994575777 3 .427396705 14 . 14226017 3148 .338056 783 .86833 975 .9773318 60 .06454529 620 . 1278222 593 .5485555 787 .2440006 2 .98932116

C12orf10 C12orf29 C12orf4 C12orf40 C12orf42 C12orf43 C12orf45 C12orf49 C12orf50 C12orf54 C12orf56 C12orf57 C12orf60 C12orf65 C12orf66 C12orf71 C12orf73 C12orf74 C12orf75 C12orf76 C12orf77 C14orf1 C14orf105 C14orf1 19 C14orf132 C14orf142 C14orf159 C14orf166 C14orf177 C14orf178 C14orf180 C14orf2 C14orf28 C14orf37 C14orf39 C14orf79 C14orf80 C14orf93 C15orf32

C15orf38-AP 0 .050454016

C15orf39 C15orf40 C15orf41 C15orf48 C15orf52 C15orf53 C15orf54 C15orf56 C15orf57 C15orf59 C15orf61 C15orf62 C15orf65 C16orf13 C16orf45 C16orf46

760 .5208798 1 133 .550358 1080 .357251 308 .0948658 1276 .822479 35 .95825146 34 .66776466 42 .05219549 661 .3303339 371 .3108507 324 .7547843 380 .3002223 78 .85356121 1530 .498832 923 .2781333 37 .50946034

-0 .425881194

-1 .098894264

-0 .032274152

-2 .012470871

-1 .390075737

-0 .097891089

-0 .654540228

0 .642978831

-2 .885599563

-0 .902016205

-0 .039764794

-1 .308841325

0 .361554126

0 .266226912

-0 .070126591

-0 .636307869

-0 .016210617

-1 .040991636

0 .467998478

-0 . 143604238

-1 .860666474

-0 .27119591

-1 .644806274

-0 .05444245

-1 .254853777

0 .099184108

0 . 195430385

-0 .485547997

-3 .609898241

-0 .92035043

2 .343633324

-0 .381561487

-0 .893274709

-0 .610870537

0 .509277318

0 .67570456

0 .491246964

-0 .915438583

-2 .937983249

-0 . 196914008

1 .068348297

-0 .081653493

-0 .806244175

-1 .42017884

0 .205944142

-0 .632925824

-0 .579337485

-0 .720868385

-0 .205499884

-0 .372617694

-0 . 126020784

-0 .961827439

-0 .646175703

-0 .089998882

-0 . 193796001

0 .221796998

0 . 12332 -3 .4534

0 .09977 -1 1 .015

0 .08776 -0 .3678

0 .20647 -9 .747

0 .37481 -3 .7087

0 .06772 -1 .4455

0 . 1 1388 -5 .7478

0 . 12739 5 .04721

0 .4819 -5 .988

0 .28374 -3 . 179

0 .21494 -0 . 185

0 . 17238 -7 .5926

0 . 17279 2 .09248

0 .06382 4 . 17152

0 .08012 -0 .8753

0 .3068 -2 .074

0 .0863 -0 . 1878

0 .48631 -2 . 1406

0 . 12778 3 .66255

0 . 16517 -0 .8694

0 .52803 -3 .5238

0 . 10922 -2 .483

0 .21124 -7 .7865

0 . 12592 -0 .4324

0 . 1921 -6 .5322

0 . 14742 0 .67281

0 . 10883 1 .79576

0 . 1 1689 -4 . 154

0 .89411 -4 .0374

0 .39703 -2 .3181

0 .61499 3 .81085

0 . 17306 -2 .2048

0 . 10455 -8 .5438

0 . 16102 -3 .7937

0 .44196 1 . 15231

0 . 10388 6 .5048

0 . 12134 4 .04868

0 .09319 -9 .8231

0 .78299 -3 .7523

3 .0711 1 -0 .0641

0 . 13876 7 .69908

0 .08732 -0 .9351

0 . 1 1404 -7 .07

0 .24129 -5 .8859

0 . 14859 1 .38596

0 .2706 -2 .339

0 .26867 -2 . 1563

0 .20729 -3 .4775

0 .09268 -2 .2172

0 . 19873 -1 .875

0 . 1 1 188 -1 . 1264

0 . 16839 -5 .712

0 . 16553 -3 .9036

0 . 13845 -0 .65

0 . 13338 -1 .453

0 . 16948 1 .30871

0 .00055 3 .25E-28 0 .71305 1 .90E-22 0 .00021 0 . 14833 9 .04E-09 4 .48E-07 2 . 12E-09 0 .00148 0 .85322 3 . 13E-14 0 .0364 3 .03E-05 0 .38142 0 .03808 0 .851 0 .03231 0 .00025 0 .38461 0 .00043 0 .01303 6 .89E-15 0 .66548 6 .48E-1 1 0 .50107 0 .07253 3 .27E-05 5 .40E-05 0 .02044 0 .00014 0 .02747 1 .30E-17 0 .00015 0 .24919 7 .78E-1 1 5 . 15E-05 8 .95E-23 0 .00018 0 .94888 1 .37E-14 0 .34973 1 .55E-12 3 .96E-09 0 . 16576 0 .01934 0 .03106 0 .00051 0 .02661 0 .06079 0 .26001 1 . 12E-08 9 .48E-05 0 .51566 0 . 14622 0 . 19063

0 .00109 1 .20E-26 0 .75371 3 .77E-21 0 .00044 0 . 18826 3 .63E-08 1 .41E-06 9 .25E-09 0 .00271 0 .8762 2 .46E-13 0 .05227 7 .24E-05 0 .43769 0 .05446 0 .87426 0 .04687 0 .00052 0 .441 0 .00085 0 .02029 5 .79E-14 0 .71029 3 .41E-10 0 .55602 0 .09832 7 .78E-05 0 .00012 0 .03083 0 .0003 0 .04045 1 .51E-16 0 .00032 0 .30004 4 .05E-10 0 .00012 1 .84E-21 0 .00037 0 .95711 1 . 1 1E-13 0 .4052 1 .00E-1 1 1 .66E-08 0 .20841 0 .02927 0 .04524 0 .001 0 .03931 0 .08364 0 .31139 4 .43E-08 0 .00021 0 .56988 0 . 18585 0 .23643

| C16orf47 | 16 .48348594 | 0 .577668101 | 0 .37119 | 1 .55625 | 0 . 1 1965 | 0 . 15508 |
| --- | --- | --- | --- | --- | --- | --- |
| C16orf52 | 2094 .839552 | -0 .306871814 | 0 . 10857 | -2 .8266 | 0 .00471 | 0 .00795 |
| C16orf54 | 461 .0587718 | 1 .965361109 | 0 .20733 | 9 .47942 | 2 .56E-21 | 4 .49E-20 |
| C16orf58 | 1567 .60543 | 0 .485635481 | 0 .07457 | 6 .51269 | 7 .38E-1 1 | 3 .85E-10 |
| C16orf59 | 182 . 149841 | 1 .962878201 | 0 .21301 | 9 .2151 | 3 . 1 1E-20 | 4 .89E-19 |
| C16orf62 | 3044 .240477 | -0 .235592013 | 0 .0832 | -2 .8317 | 0 .00463 | 0 .00784 |
| C16orf70 | 1631 .004283 | -0 .006606011 | 0 .09494 | -0 .0696 | 0 .94453 | 0 .95358 |
| C16orf71 | 59 .91425225 | -0 . 166130638 | 0 . 12772 | -1 .3007 | 0 . 19335 | 0 .23943 |
| C16orf72 | 4765 .045502 | 0 .392848269 | 0 .05547 | 7 .08179 | 1 .42E-12 | 9 .27E-12 |
| C16orf74 | 273 .86408 | -0 .339246874 | 0 . 17992 | -1 .8855 | 0 .05936 | 0 .08186 |
| C16orf78 | 3 .412845987 | -2 .56647906 | 0 .6071 | -4 .2275 | 2 .36E-05 | 5 .76E-05 |
| C16orf86 | 164 .0901582 | 0 .534719065 | 0 . 15556 | 3 .43748 | 0 .00059 | 0 .00115 |
| C16orf87 | 823 .6864605 | -0 .086015039 | 0 .06877 | -1 .2508 | 0 .21102 | 0 .25854 |
| C16orf89 | 70 .68715923 | 1 .271525363 | 0 .32594 | 3 .90107 | 9 .58E-05 | 0 .00021 |
| C16orf90 | 4 .315531686 | -1 .391475872 | 0 .33407 | -4 . 1653 | 3 . 1 1E-05 | 7 .43E-05 |
| C16orf91 | 851 . 1390626 | 0 .212151359 | 0 . 14334 | 1 .48004 | 0 . 13886 | 0 . 17741 |
| C16orf92 | 1 . 156123471 | -3 .672308954 | 0 .91404 | -4 .0177 | 5 .88E-05 | 0 .00013 |
| C16orf95 | 47 .59853243 | 0 .297871841 | 0 . 19248 | 1 .54758 | 0 . 12172 | 0 . 15742 |
| C16orf96 | 29 . 19970353 | -0 .967011 1 16 | 0 .22016 | -4 .3922 | 1 . 12E-05 | 2 .87E-05 |
| C16orf97 | 9 .988746084 | -2 .597507564 | 0 .50113 | -5 . 1833 | 2 . 18E-07 | 7 . 18E-07 |
| C17orf102 | 8 .072513486 | -3 .854269414 | 0 .63446 | -6 .0749 | 1 .24E-09 | 5 .56E-09 |
| C17orf105 | 15 .67084183 | -1 .569134317 | 0 .36107 | -4 .3458 | 1 .39E-05 | 3 .50E-05 |
| C17orf107 | 83 .74697438 | 0 .210219507 | 0 . 16975 | 1 .23839 | 0 .21557 | 0 .2635 |
| C17orf1 12 | 3 .905171492 | -5 .475568159 | 0 .94226 | -5 .811 1 | 6 .21E-09 | 2 .55E-08 |
| C17orf47 | 8 . 1 1365005 | -0 .780884741 | 0 .4097 | -1 .906 | 0 .05665 | 0 .07842 |
| C17orf49 | 42 .29784273 | -0 .438868823 | 0 . 13216 | -3 .3207 | 0 .0009 | 0 .00171 |
| C17orf51 | 520 .5284043 | 0 .388617118 | 0 . 10788 | 3 .60216 | 0 .00032 | 0 .00064 |
| C17orf53 | 126 .2440998 | 0 .897119466 | 0 . 12556 | 7 . 14501 | 9 .00E-13 | 5 .97E-12 |
| C17orf58 | 689 . 1 123826 | 0 .760986194 | 0 . 13128 | 5 .79655 | 6 .77E-09 | 2 .77E-08 |
| C17orf62 | 2425 .868979 | 1 . 16477731 | 0 .08936 | 13 .034 | 7 .84E-39 | 8 .00E-37 |
| C17orf64 | 6 .093789533 | -0 .988122605 | 0 .33047 | -2 .99 | 0 .00279 | 0 .0049 |
| C17orf67 | 126 .9880804 | 0 .567322299 | 0 . 12204 | 4 .64855 | 3 .34E-06 | 9 . 19E-06 |
| C17orf74 | 1 .654381542 | -2 .336956455 | 0 .88671 | -2 .6355 | 0 .0084 | 0 .01356 |
| C17orf75 | 1 1 19 .815613 | -0 .274118028 | 0 . 1 1301 | -2 .4257 | 0 .01528 | 0 .02349 |
| C17orf77 | 13 .36483687 | -1 .436944352 | 0 .74686 | -1 .924 | 0 .05436 | 0 .07552 |
| C17orf80 | 1535 .238526 | -0 . 19051548 | 0 .08285 | -2 .2995 | 0 .02148 | 0 .03225 |
| C17orf82 | 75 .99280848 | -0 .675135592 | 0 .24947 | -2 .7063 | 0 .0068 | 0 .011 17 |
| C17orf89 | 1 172 .89954 | 0 .691003696 | 0 . 17782 | 3 .88607 | 0 .0001 | 0 .00022 |
| C17orf97 | 61 .45277102 | 0 .725024633 | 0 .23809 | 3 .04513 | 0 .00233 | 0 .00413 |
| C17orf99 | 14 . 18884463 | -1 .210212529 | 0 .29261 | -4 . 136 | 3 .53E-05 | 8 .37E-05 |
| C18orf21 | 1264 .763127 | -0 . 15972344 | 0 . 1 1602 | -1 .3767 | 0 . 16861 | 0 .2117 |
| C18orf25 | 3710 .013763 | -0 .003808297 | 0 .06926 | -0 .055 | 0 .95615 | 0 .96308 |
| C18orf32 | 187 .2298775 | 0 .671421762 | 0 . 19092 | 3 .51671 | 0 .00044 | 0 .00087 |
| C18orf54 | 718 .577574 | -0 . 15305167 | 0 . 13622 | -1 . 1236 | 0 .26118 | 0 .31269 |
| C18orf63 | 16 .08884553 | -3 .759297335 | 0 .70003 | -5 .3702 | 7 .87E-08 | 2 .78E-07 |
| C18orf8 | 1333 .423973 | 0 .641162873 | 0 .07333 | 8 .74315 | 2 .27E-18 | 2 .89E-17 |
| C19orf12 | 2853 .396586 | 1 .215577526 | 0 . 12052 | 10 .0858 | 6 .39E-24 | 1 .49E-22 |
| C19orf18 | 40 .94588037 | -0 .468085603 | 0 .22347 | -2 .0946 | 0 .0362 | 0 .05202 |
| C19orf24 | 657 .4252921 | 0 .697048842 | 0 . 13814 | 5 .04606 | 4 .51E-07 | 1 .42E-06 |
| C19orf25 | 1346 .987209 | 0 .476413532 | 0 .0959 | 4 .96759 | 6 .78E-07 | 2 .07E-06 |
| C19orf33 | 64 .82204789 | -1 .559849014 | 0 .31445 | -4 .9605 | 7 .03E-07 | 2 . 14E-06 |
| C19orf35 | 54 .82407069 | 1 .252350874 | 0 .21729 | 5 .7636 | 8 .23E-09 | 3 .32E-08 |
| C19orf38 | 96 .97320375 | 1 .308079731 | 0 .2165 | 6 .04197 | 1 .52E-09 | 6 .75E-09 |
| C19orf43 | 4841 .447846 | -0 .063171165 | 0 . 14311 | -0 .4414 | 0 .6589 | 0 .7046 |
| C19orf44 | 229 .2279382 | -0 .30390153 | 0 . 10376 | -2 .9288 | 0 .0034 | 0 .00589 |
| C19orf45 | 6 .3066412 | -2 . 19904409 | 0 .40188 | -5 .472 | 4 .45E-08 | 1 .62E-07 |

| C19orf47  C19orf48  C19orf52  C19orf53  C19orf54  C19orf57  C19orf60  C19orf66  C19orf67  C19orf68  C19orf70  C19orf71  C19orf73  C19orf81  C19orf84  C1D C1GALT1 | 398 .317965 2181 . 186915 1015 .495725 2573 .895212 136 .6067281 240 .9128946 2058 .861238 786 .2053147 37 .99346296 75 .39972417 2406 .679724 658 .397273 30 . 12944733 32 .88620675 3 .260358445 1 139 .326616 4015 .622402 |
| --- | --- |

C1GALT1C1 1640 .27132

| C1QA C1QB C1QBP C1QC C1QL1 C1QL2 C1QL3 C1QL4 C1QTNF1 | 7607 .551478 5370 .620614 6738 .985956 2162 .546362 22 .640586 5 .274890914 330 .8703477 75 .33504823 1528 .882279 |
| --- | --- |

C1QTNF1-AS15 .74801601 C1QTNF2 1 16 .6195157 C1QTNF3 493 .8712578 C1QTNF3-AM39 .04221521

| C1 C1 C1 C1 C1 C1 C1 C1 C1 C1 C1 C1 C1 C1 C1 C1 C1 C1 C1 C1 C1 C1 C1 C1 C1 | QTNF4 QTNF6 QTNF7 QTNF8 QTNF9 QTNF9B R  RL  S  orf100  orf101  orf105  orf106  orf109  orf1 1 1  orf1 12  orf1 15  orf1 16  orf122  orf123  orf127  orf131  orf137  orf141  orf143 | 79 . 1899293 542 .4211079 609 .8535865 1 .452339295 10 .51386211 15 .98552806 8060 .894107 1476 .829415 18163 .02234 16 .49528523 406 .589298 36 .88664814 1332 .256588 1507 .301186 17 .86098924 1018 .857384 889 .9617391 2334 .816794 1716 .985529 2572 .456032 30 .38057338 1068 .250286 3 .630437441 14 .42285591 4 .698253901 |
| --- | --- | --- |

0 .3667341

-0 . 160101846

0 .796566717

-0 .246528071

0 .469613861

-0 .026631792

0 .719200684

0 .952071569

-0 .919346757

0 .94049106

-0 . 188536532

1 .944243043

-0 . 126885541

1 .024498203

-2 .064864145

-0 .324253488

0 .418519593

0 . 167562371

2 .326914653

3 .306350503

0 .643120999

2 .557334856

1 .814044517

-1 . 135633248

-2 .013951879

0 .337118786

1 .750919773

0 .451795356

-0 .291151639

-0 .529697359

-1 .741565997

-0 .054163156

1 . 1 10795689

-1 .550286966

-1 .691768478

-0 .949073918

-1 .741926436

1 .631280616

0 .668291851

1 .359723824

-0 .64074254

-0 .92208807

-1 .567921554

-0 .560628192

-0 . 108300788

-1 .549674749

-0 . 126498904

0 .896227127

-1 .051863525

1 .0473725

-0 .515155668

0 .676303733

-0 .047188126

-1 .059156855

-4 .810251922

-3 .794821917

0 . 10622 0 . 12672 0 .08637 0 . 19514 0 . 13786 0 . 14438 0 . 12891 0 . 1 1274 0 . 13609 0 . 16259 0 . 14899 0 .22074 0 . 18876 0 .31061 0 .58099 0 . 1 1 127 0 . 12782 0 . 1 1374 0 .25256 0 .25894 0 . 15499 0 .20808 0 .30055 0 .5933 0 .20492 0 .33039 0 . 13556 0 .2736 0 . 19239 0 . 18575 0 .21302 0 .26035 0 . 12174 0 .26163 0 .65967 0 .2857 0 .2928 0 . 16652 0 . 12109 0 . 18116 0 .21856 0 .23275 0 .20819 0 .24909 0 . 10377 0 .31641 0 . 10784 0 . 19229 0 .24484 0 . 16057 0 . 1033 0 .26007 0 .07219 0 .49364 0 .5998 0 .59982

3 .4526 -1 .2635 9 .22326 -1 .2633 3 .40653 -0 . 1845 5 .57901 8 .44482 -6 .7553 5 .7845 -1 .2654 8 .80797 -0 .6722 3 .2983 -3 .554 -2 .914 3 .27442 1 .47324 9 .21326 12 .7686 4 . 14954 12 .2902 6 .03573 -1 .9141 -9 .8282 1 .02037 12 .9166 1 .65127 -1 .5133 -2 .8516 -8 . 1758 -0 .208 9 . 12447 -5 .9256 -2 .5646 -3 .3219 -5 .9493 9 .79653 5 .51907 7 .50558 -2 .9317 -3 .9618 -7 .5313 -2 .2507 -1 .0437 -4 .8976 -1 . 173 4 .66077 -4 .2961 6 .52299 -4 .9868 2 .60047 -0 .6537 -2 . 1456 -8 .0198 -6 .3266

0 .00056 0 .20642 2 .88E-20 0 .20647 0 .00066 0 .85365 2 .42E-08 3 .05E-17 1 .43E-1 1 7 .27E-09 0 .20573 1 .27E-18 0 .50145 0 .00097 0 .00038 0 .00357 0 .00106 0 . 14069 3 . 16E-20 2 .46E-37 3 .33E-05 1 .02E-34 1 .58E-09 0 .05561 8 .52E-23 0 .30755 3 .63E-38 0 .09868 0 . 1302 0 .00435 2 .94E-16 0 .8352 7 .21E-20 3 . 1 1E-09 0 .01033 0 .00089 2 .69E-09 1 . 17E-22 3 .41E-08 6 . 12E-14 0 .00337 7 .44E-05 5 .02E-14 0 .0244 0 .29662 9 .70E-07 0 .24078 3 . 15E-06 1 .74E-05 6 .89E-1 1 6 . 14E-07 0 .00931 0 .51332 0 .03191 1 .06E-15 2 .51E-10

0 .00109 0 .25363 4 .55E-19 0 .25367 0 .00128 0 .8765 9 . 17E-08 3 .40E-16 8 . 15E-1 1 2 .96E-08 0 .25292 1 .67E-17 0 .55632 0 .00184 0 .00076 0 .00616 0 .00199 0 . 1794 4 .96E-19 2 .27E-35 7 .93E-05 7 .08E-33 7 .00E-09 0 .07706 1 .75E-21 0 .36186 3 .53E-36 0 . 13004 0 . 16735 0 .00739 2 .93E-15 0 .86055 1 .09E-18 1 .32E-08 0 .01638 0 .0017 1 . 16E-08 2 .36E-21 1 .27E-07 4 .63E-13 0 .00584 0 .00017 3 .84E-13 0 .0363 0 .35017 2 .90E-06 0 .29112 8 .69E-06 4 .32E-05 3 .61E-10 1 .89E-06 0 .0149 0 .56774 0 .04634 9 .78E-15 1 .23E-09

| C1orf145 | 74 .39184971 | -1 .351560876 | 0 . 18097 -7 .4684 | 8 . 12E-14 | 6 .02E-13 |
| --- | --- | --- | --- | --- | --- |
| C1orf146 | 31 .53756901 | -1 .003365707 | 0 .20908 -4 .799 | 1 .59E-06 | 4 .60E-06 |
| C1orf147 | 58 .57847685 | -0 .399908738 | 0 . 16177 -2 .472 | 0 .01343 | 0 .02087 |
| C1orf158 | 38 .86519835 | 0 .386350327 | 0 .85967 0 .44942 | 0 .65313 | 0 .69909 |
| C1orf159 | 548 .3749736 | 0 . 173603375 | 0 . 10222 1 .6984 | 0 .08943 | 0 . 1 1915 |
| C1orf162 | 480 .4235344 | 0 .807605862 | 0 . 16358 4 .93721 | 7 .92E-07 | 2 .40E-06 |
| C1orf167 | 7 .483304913 | -1 .74061273 | 0 .37465 -4 .6459 | 3 .39E-06 | 9 .31E-06 |
| C1orf168 | 25 .79425392 | -2 .094803213 | 0 .45915 -4 .5623 | 5 .06E-06 | 1 .36E-05 |
| C1orf174 | 1307 .897042 | 0 .541467992 | 0 .06943 7 .79832 | 6 .27E-15 | 5 .30E-14 |
| C1orf185 | 6 .063507458 | -2 .354998887 | 0 .50455 -4 .6675 | 3 .05E-06 | 8 .43E-06 |
| C1orf189 | 6 .500772011 | -1 .297141237 | 0 .2816 -4 .6062 | 4 . 10E-06 | 1 . 1 1E-05 |
| C1orf194 | 21 .97609339 | -0 .928815083 | 0 .24193 -3 .8391 | 0 .00012 | 0 .00027 |
| C1orf195 | 58 .94959502 | -0 .024298742 | 0 .20542 -0 . 1 183 | 0 .90584 | 0 .92169 |
| C1orf198 | 5512 . 154666 | 0 .500407293 | 0 . 10771 4 .64573 | 3 .39E-06 | 9 .31E-06 |
| C1orf204 | 89 .84393892 | -0 .38498987 | 0 . 19692 -1 .9551 | 0 .05058 | 0 .07073 |
| C1orf21 | 8498 .941724 | -0 .512188371 | 0 .09795 -5 .2289 | 1 .71E-07 | 5 .69E-07 |
| C1orf210 | 173 .9716813 | -0 .296306409 | 0 .30233 -0 .9801 | 0 .32705 | 0 .38196 |
| C1orf216 | 1770 .560954 | 0 .584225383 | 0 . 13661 4 .27654 | 1 .90E-05 | 4 .70E-05 |
| C1orf220 | 23 . 1 109106 | -0 .955050317 | 0 .20283 -4 .7087 | 2 .49E-06 | 6 .98E-06 |
| C1orf226 | 102 .2912106 | -0 .022529676 | 0 .20415 -0 . 1 104 | 0 .91212 | 0 .92711 |
| C1orf228 | 90 .56022256 | 0 .326779566 | 0 . 1701 1 .92107 | 0 .05472 | 0 .07596 |
| C1orf229 | 48 .6000119 | -1 .027204914 | 0 . 18087 -5 .6793 | 1 .35E-08 | 5 .30E-08 |
| C1orf234 | 3 .733590836 | -2 . 182203448 | 0 .48589 -4 .4911 | 7 .08E-06 | 1 .86E-05 |
| C1orf27 | 3331 .03449 | -0 .27151911 | 0 . 1 1047 -2 .4579 | 0 .01398 | 0 .02164 |
| C1orf35 | 744 .455123 | 0 . 172938325 | 0 .09293 1 .86094 | 0 .06275 | 0 .08606 |
| C1orf43 | 10390 .30911 | -0 . 154140926 | 0 . 12718 -1 .212 | 0 .22552 | 0 .27438 |
| C1orf50 | 451 .5892105 | -0 .286517484 | 0 .09866 -2 .9042 | 0 .00368 | 0 .00634 |
| C1orf52 | 1409 .252804 | -0 .237710703 | 0 .08054 -2 .9514 | 0 .00316 | 0 .0055 |
| C1orf53 | 131 . 1636621 | 0 .201490388 | 0 .22813 0 .88322 | 0 .37712 | 0 .43327 |
| C1orf54 | 380 .9937691 | 0 .830223641 | 0 . 16513 5 .02781 | 4 .96E-07 | 1 .55E-06 |
| C1orf56 | 179 . 1587777 | 0 .001316617 | 0 .09408 0 .014 | 0 .98883 | 0 .99043 |
| C1orf61 | 43 . 10417249 | -1 .280610415 | 0 .30759 -4 . 1633 | 3 . 14E-05 | 7 .49E-05 |
| C1orf64 | 5 .276979016 | -2 .593547749 | 0 .78889 -3 .2876 | 0 .00101 | 0 .00191 |
| C1orf68 | 1801 .696595 | -2 .616450292 | 0 .32144 -8 . 1397 | 3 .96E-16 | 3 .90E-15 |
| C1orf74 | 490 . 1861994 | 0 .020021732 | 0 . 1 1 169 0 . 17926 | 0 .85773 | 0 .87998 |
| C1orf87 | 18 .43047383 | -3 .03621726 | 0 .42326 -7 . 1733 | 7 .32E-13 | 4 .91E-12 |
| C1orf94 | 5 .409109866 | -3 .419110599 | 0 .62625 -5 .4596 | 4 .77E-08 | 1 .73E-07 |
| C2 | 722 . 1208046 | 1 .827764004 | 0 .22163 8 .24686 | 1 .63E-16 | 1 .66E-15 |
| C20orf141 | 1 .069608562 | 0 .298568052 | 0 .68419 0 .43638 | 0 .66256 | 0 .70776 |
| C20orf144 | 44 .91132023 | 0 .01991546 | 0 . 1776 0 . 1 1214 | 0 .91072 | 0 .92597 |
| C20orf173 | 0 .562166692 | -2 .343754095 | 0 .8331 -2 .8133 | 0 .0049 | 0 .00826 |
| C20orf187 | 4 .044147115 | -3 .547065172 | 0 .54763 -6 .4772 | 9 .35E-1 1 | 4 .82E-10 |
| C20orf194 | 3945 .887178 | 0 .386136741 | 0 .09746 3 .96213 | 7 .43E-05 | 0 .00017 |
| C20orf196 | 259 .9854136 | -0 .265354283 | 0 . 12027 -2 .2063 | 0 .02737 | 0 .04033 |
| C20orf197 | 56 .35322673 | -0 .46006208 | 0 . 19276 -2 .3868 | 0 .017 | 0 .02595 |
| C20orf202 | 38 .03326364 | -0 .003800937 | 0 . 15514 -0 .0245 | 0 .98045 | 0 .98327 |
| C20orf24 | 593 .257117 | 1 .967119675 | 0 .218 9 .0233 | 1 .83E-19 | 2 .64E-18 |
| C20orf27 | 1436 .453937 | 0 .772705595 | 0 . 13788 5 .60427 | 2 .09E-08 | 7 .99E-08 |
| C20orf78 | 2 . 146003576 | -3 . 150624247 | 0 .64007 -4 .9223 | 8 .55E-07 | 2 .58E-06 |
| C20orf85 | 4 .079247888 | -1 .321523694 | 0 .42377 -3 . 1 185 | 0 .00182 | 0 .00329 |
| C20orf96 | 258 .3721487 | -0 .052726616 | 0 . 14917 -0 .3535 | 0 .72373 | 0 .7632 |
| C21orf140 | 37 .04717326 | -0 .675582032 | 0 . 1818 -3 .716 | 0 .0002 | 0 .00042 |
| C21orf2 | 522 . 1587318 | 0 .718309065 | 0 .09896 7 .25889 | 3 .90E-13 | 2 .70E-12 |
| C21orf33 | 4332 .026576 | -0 .044304615 | 0 . 10757 -0 .4119 | 0 .68044 | 0 .72371 |
| C21orf58 | 233 .6686118 | 0 .400328442 | 0 . 12071 3 .3164 | 0 .00091 | 0 .00173 |
| C21orf59 | 641 .7494439 | 0 . 172497374 | 0 .09756 1 .7681 | 0 .07704 | 0 . 10396 |

| C21orf62 65 .67122046 - 1 .238221536 | 0 .21591 -5 .735 | 9 .75E-09 | 3 .89E-08 |
| --- | --- | --- | --- |
| C21orf62-AS 122 .847656 -0 .971038172 | 0 . 14723 -6 .5956 | 4 .24E-1 1 | 2 .29E-10 |
| C21orf91 4382 .738929 0 .039887458 | 0 . 12608 0 .31636 | 0 .75173 | 0 .78689 |
| C22orf15 35 .07151448 -0 .015529471 | 0 .20318 -0 .0764 | 0 .93908 | 0 .94897 |
| C22orf23 622 .6937387 0 .347225646 | 0 . 12481 2 .782 | 0 .0054 | 0 .00904 |
| C22orf24 18 .95297675 -0 .083553177 | 0 .20776 -0 .4022 | 0 .68756 | 0 .73029 |
| C22orf29 1822 .213547 -0 . 100594274 | 0 . 12049 -0 .8349 | 0 .4038 | 0 .46036 |
| C22orf31 34 .78043173 -0 .424014909 | 0 .29485 -1 .4381 | 0 . 15041 | 0 . 19065 |
| C22orf34 181 .4621467 1 . 169709202 | 0 . 17145 6 .82244 | 8 .95E-12 | 5 .23E-1 1 |
| C22orf39 1622 .526802 -0 .091404921 | 0 .08235 -1 . 1 1 | 0 .26699 | 0 .31873 |
| C22orf42 6 .026938272 - 1 .446419092 | 0 .6561 -2 .2046 | 0 .02748 | 0 .04046 |
| C22orf46 462 .6543477 0 .307821155 | 0 . 1 1052 2 .78523 | 0 .00535 | 0 .00896 |
| C2CD2 2867 .482405 0 .042653132 | 0 . 15603 0 .27336 | 0 .78457 | 0 .81601 |
| C2CD2L 690 . 1816164 0 .30744129 | 0 .09965 3 .08514 | 0 .00203 | 0 .00365 |
| C2CD3 2068 . 193845 -0 .224982129 | 0 .08662 -2 .5973 | 0 .0094 | 0 .01503 |
| C2CD4A 37 . 15479227 -0 . 172299838 | 0 .23875 -0 .7217 | 0 .4705 | 0 .52617 |
| C2CD4B 61 .33013218 0 .741455401 | 0 .24666 3 .00602 | 0 .00265 | 0 .00466 |
| C2CD4C 34 .85410766 1 .48714484 | 0 .27195 5 .46841 | 4 .54E-08 | 1 .65E-07 |
| C2CD4D 1 16 .0758676 0 .345579227 | 0 .21276 1 .6243 | 0 . 10431 | 0 . 13688 |
| C2CD5 4535 .573944 -0 .506223007 | 0 . 10432 -4 .8526 | 1 .22E-06 | 3 .58E-06 |
| C2orf15 72 . 17891152 -0 .587161749 | 0 . 13517 -4 .3438 | 1 .40E-05 | 3 .53E-05 |
| C2orf16 167 .8602273 -0 .225391574 | 0 . 13262 -1 .6995 | 0 .08923 | 0 . 1 1891 |
| C2orf27A 280 .8565681 0 . 127229164 | 0 . 17303 0 .73529 | 0 .46216 | 0 .51795 |
| C2orf27B 0 .941255205 -2 .305343516 | 0 .71102 -3 .2423 | 0 .00119 | 0 .00221 |
| C2orf40 100 . 1389639 - 1 .003500611 | 0 .3674 -2 .7313 | 0 .00631 | 0 .01042 |
| C2orf42 680 .4183276 -0 .469462463 | 0 .06731 -6 .975 | 3 .06E-12 | 1 .91E-1 1 |
| C2orf47 818 .39917 -0 . 1 12343926 | 0 .09387 -1 . 1968 | 0 .23137 | 0 .28082 |
| C2orf48 19 .94636732 -0 . 188521771 | 0 .27177 -0 .6937 | 0 .48788 | 0 .54296 |
| C2orf49 2572 . 121166 -0 . 185891093 | 0 .07503 -2 .4775 | 0 .01323 | 0 .02058 |
| C2orf50 18 .76015273 - 1 .354772952 | 0 .27206 -4 .9796 | 6 .37E-07 | 1 .96E-06 |
| C2orf54 1315 .76064 -0 .950661998 | 0 .26528 -3 .5836 | 0 .00034 | 0 .00069 |
| C2orf57 2 .465516696 - 1 .546721594 | 0 .5687 -2 .7198 | 0 .00653 | 0 .01076 |
| C2orf61 26 .83799805 -0 .563855447 | 0 .20103 -2 .8049 | 0 .00503 | 0 .00847 |
| C2orf66 32 . 19755223 -0 .738171278 | 0 .26187 -2 .8189 | 0 .00482 | 0 .00813 |
| C2orf68 2018 .709057 0 .067555594 | 0 .06146 1 .09916 | 0 .2717 | 0 .32385 |
| C2orf69 2149 .61465 -0 .337866455 | 0 .09402 -3 .5937 | 0 .00033 | 0 .00066 |
| C2orf70 55 .97317261 -0 .051255972 | 0 .33561 -0 . 1527 | 0 .87862 | 0 .89824 |
| C2orf71 52 .26040954 - 1 .774952135 | 0 .25173 -7 .051 | 1 .78E-12 | 1 . 14E-1 1 |
| C2orf72 175 .5875967 3 .559156929 | 0 .32965 10 .7968 | 3 .56E-27 | 1 . 19E-25 |
| C2orf73 28 .31371843 - 1 .588426282 | 0 .24235 -6 .5542 | 5 .59E-1 1 | 2 .98E-10 |
| C2orf74 0 .292684249 -0 .435753674 | 1 .5124 -0 .2881 | 0 .77325 | 0 .80543 |
| C2orf76 667 .7506362 -0 .814027804 | 0 . 13645 -5 .9657 | 2 .44E-09 | 1 .05E-08 |
| C2orf78 8 .263565031 -4 .454707047 | 0 .61686 -7 .2216 | 5 . 14E-13 | 3 .50E-12 |
| C2orf80 6 .821027021 -4 .338639047 | 0 .6075 -7 . 1418 | 9 .21E-13 | 6 . 1 1E-12 |
| C2orf81 164 .673119 -0 . 1 13712803 | 0 . 1 1712 -0 .9709 | 0 .33161 | 0 .38661 |
| C2orf82 202 . 1283656 0 .854371688 | 0 .29948 2 .85282 | 0 .00433 | 0 .00736 |
| C2orf83 5 .024307819 -3 .319641431 | 0 .71241 -4 .6597 | 3 . 17E-06 | 8 .74E-06 |
| C2orf88 3795 .903366 - 1 .476114822 | 0 . 18194 -8 . 1 133 | 4 .93E-16 | 4 .80E-15 |
| C2orf91 1 1 .61836512 -2 .252092675 | 0 .39164 -5 .7504 | 8 .90E-09 | 3 .58E-08 |
| C3 16826 .05776 2 .474860684 | 0 .28069 8 .817 | 1 . 18E-18 | 1 .55E-17 |
| C3AR1 486 .2298348 1 .824331009 | 0 .20479 8 .9085 | 5 . 17E-19 | 7 .09E-18 |
| C3orf14 1274 .481202 -0 .426991774 | 0 . 16078 -2 .6558 | 0 .00791 | 0 .01284 |
| C3orf18 756 .9113487 -0 .68248129 | 0 . 14542 -4 .6933 | 2 .69E-06 | 7 .50E-06 |
| C3orf20 38 .91893399 - 1 .239983365 | 0 .22901 -5 .4146 | 6 . 14E-08 | 2 .20E-07 |
| C3orf22 6 .648717934 - 1 .415016137 | 0 .36703 -3 .8553 | 0 .00012 | 0 .00025 |
| C3orf30 10 .55861952 -0 .955457058 | 0 .43311 -2 .206 | 0 .02738 | 0 .04035 |

| C3orf33 | 261 .4923521 -0 .2727217 | 0 . 12247 -2 .2268 | 0 .02596 | 0 .03845 |
| --- | --- | --- | --- | --- |
| C3orf35 | 215 .3178717 -0 .862606153 | 0 . 19188 -4 .4956 | 6 .94E-06 | 1 .82E-05 |
| C3orf36 | 16 . 13158554 0 .498478908 | 0 .29041 1 .71647 | 0 .08608 | 0 . 1 1502 |
| C3orf38 | 3132 .507124 -0 .043742146 | 0 .09448 -0 .463 | 0 .6434 | 0 .69021 |
| C3orf49 | 88 .66904626 -0 .974160835 | 0 . 15954 -6 . 1059 | 1 .02E-09 | 4 .64E-09 |
| C3orf52 | 2345 . 1 17917 -1 .888840946 | 0 .21133 -8 .9378 | 3 .97E-19 | 5 .48E-18 |
| C3orf56 | 2 .584123713 -3 .556953454 | 0 .78225 -4 .5471 | 5 .44E-06 | 1 .45E-05 |
| C3orf58 | 3375 . 145749 -0 .320690205 | 0 . 14172 -2 .2629 | 0 .02364 | 0 .03525 |
| C3orf62 | 449 .5725158 0 .228617643 | 0 .07682 2 .9761 | 0 .00292 | 0 .00511 |
| C3orf67 | 375 .8424342 0 .24135335 | 0 .22196 1 .08739 | 0 .27686 | 0 .32949 |
| C3orf70 | 2150 .468836 -0 .584258382 | 0 . 16009 -3 .6495 | 0 .00026 | 0 .00054 |
| C3orf79 | 186 .8570676 -0 .25943182 | 0 .6146 -0 .4221 | 0 .67294 | 0 .71693 |
| C3orf80 | 42 .5436664 2 .919331973 | 0 .31653 9 .22297 | 2 .89E-20 | 4 .56E-19 |
| C3orf84 | 13 .07650517 -0 .693920653 | 0 .22858 -3 .0358 | 0 .0024 | 0 .00426 |
| C4A | 89 .69512548 1 .682838147 | 0 .21968 7 .66024 | 1 .86E-14 | 1 .49E-13 |
| C4B | 66 .88344237 2 .706573435 | 0 .29042 9 .31938 | 1 . 17E-20 | 1 .93E-19 |
| C4BPA | 27 .36242638 -2 .278429477 | 0 .30371 -7 .502 | 6 .28E-14 | 4 .74E-13 |
| C4BPB | 20 .05845729 -1 .424517041 | 0 .30563 -4 .6609 | 3 . 15E-06 | 8 .69E-06 |
| C4orf17 | 1 1 .64316905 -3 .478697151 | 0 .56732 -6 . 1318 | 8 .69E-10 | 3 .98E-09 |
| C4orf19 | 507 .5918628 -1 .219697417 | 0 . 19866 -6 . 1397 | 8 .27E-10 | 3 .80E-09 |
| C4orf22 | 22 .3387375 -2 .214222592 | 0 .66256 -3 .3419 | 0 .00083 | 0 .00159 |
| C4orf26 | 1 1 .33695992 -1 .318598392 | 0 .38638 -3 .4127 | 0 .00064 | 0 .00125 |
| C4orf3 | 9048 .747022 -0 .437817841 | 0 .08125 -5 .3885 | 7 . 1 1E-08 | 2 .52E-07 |
| C4orf32 | 1368 .534461 -0 . 156931801 | 0 . 1559 -1 .0066 | 0 .31412 | 0 .3686 |
| C4orf33 | 1 162 .838684 -0 .657955025 | 0 .0982 -6 .7001 | 2 .08E-1 1 | 1 . 17E-10 |
| C4orf36 | 407 . 1913151 -0 .649239973 | 0 .09296 -6 .9838 | 2 .87E-12 | 1 .80E-1 1 |
| C4orf45 | 42 .97001202 -0 .511222582 | 0 .20339 -2 .5135 | 0 .01195 | 0 .01874 |
| C4orf46 | 935 .9653624 0 .557642683 | 0 .09667 5 .7688 | 7 .98E-09 | 3 .23E-08 |
| C4orf47 | 72 .07103588 -0 .802307922 | 0 . 16911 -4 .7443 | 2 .09E-06 | 5 .92E-06 |
| C4orf48 | 651 .8985686 1 .934868916 | 0 .25932 7 .46145 | 8 .56E-14 | 6 .33E-13 |
| C4orf50 | 15 .33632719 -1 . 146826915 | 0 .43355 -2 .6452 | 0 .00816 | 0 .01321 |
| C4orf51 | 9 .361125601 -2 .008337377 | 0 .25598 -7 .8456 | 4 .31E-15 | 3 .73E-14 |
| C5 | 1 121 .376541 -1 .40552106 | 0 . 13883 -10 . 124 | 4 .33E-24 | 1 .03E-22 |
| C5AR1 | 554 .446453 3 . 1 14777475 | 0 .27129 1 1 .4813 | 1 .64E-30 | 7 .39E-29 |
| C5AR2 | 186 .345341 1 .714187418 | 0 . 16302 10 .5151 | 7 .36E-26 | 2 . 14E-24 |
| C5orf15 | 6254 .49726 0 .268931775 | 0 . 1 1751 2 .28868 | 0 .0221 | 0 .03312 |
| C5orf17 | 68 .02174921 0 .618350137 | 0 .48462 1 .27595 | 0 .20197 | 0 .24881 |
| C5orf22 | 1757 .860497 0 .68508404 | 0 . 1 1867 5 .77287 | 7 .79E-09 | 3 . 16E-08 |
| C5orf24 | 6200 .057101 0 .049993736 | 0 . 10297 0 .48552 | 0 .62731 | 0 .67579 |
| C5orf30 | 2374 .280536 -0 .942779086 | 0 . 14293 -6 .5962 | 4 .22E-1 1 | 2 .28E-10 |
| C5orf34 | 446 .8818872 0 .038295631 | 0 . 1029 0 .37216 | 0 .70977 | 0 .75079 |
| C5orf38 | 133 .7491464 -0 .413899667 | 0 .26423 -1 .5664 | 0 . 1 1725 | 0 . 15225 |
| C5orf42 | 6641 .523675 -0 .531015243 | 0 . 10819 -4 .9082 | 9 . 19E-07 | 2 .76E-06 |
| C5orf45 | 958 .6319136 -0 .293968778 | 0 . 1 1263 -2 .61 | 0 .00905 | 0 .01453 |
| C5orf46 | 1679 . 109348 -2 .474928967 | 0 .30307 -8 . 1663 | 3 . 18E-16 | 3 . 16E-15 |
| C5orf47 | 16 .3054938 -1 .45217028 | 0 .40324 -3 .6013 | 0 .00032 | 0 .00065 |
| C5orf49 | 43 . 16064406 -0 .034905936 | 0 .24368 -0 . 1432 | 0 .8861 | 0 .90452 |
| C5orf51 | 4009 .835243 -0 .00508119 | 0 .07342 -0 .0692 | 0 .94482 | 0 .95382 |
| C5orf52 | 1 .594966119 -2 .7173108 | 0 .71934 -3 .7775 | 0 .00016 | 0 .00034 |
| C5orf56 | 459 .7656338 0 .035250567 | 0 . 14911 0 .2364 | 0 .81312 | 0 .84123 |
| C5orf58 | 1 1 1 .8920728 1 . 147965194 | 0 .21322 5 .38384 | 7 .29E-08 | 2 .58E-07 |
| C5orf60 | 5 . 150032393 -2 . 138966626 | 0 .56565 -3 .7814 | 0 .00016 | 0 .00033 |
| C5orf63 | 644 .3097997 -1 .451562748 | 0 . 14462 -10 .037 | 1 .05E-23 | 2 .39E-22 |
| C5orf64 | 24 .55715695 -0 .926689206 | 0 .34921 -2 .6537 | 0 .00796 | 0 .01291 |
| C5orf66 | 136 .5478318 -0 .266240324 | 0 . 17141 -1 .5532 | 0 . 12037 | 0 . 15583 |
| C5orf67 | 25 .45072581 -0 .861906328 | 0 .40142 -2 . 1472 | 0 .03178 | 0 .04619 |

66 .26390078 1567 .603023 58 . 10016682 7831 .084535 7 .366160216 3357 .549231 1778 .672727 215 .3645419 200 .3731529 88 . 18313361 292 .7233419 43 .5714865 592 .6904335 22 .87665079 10 .43611835 201 .5638515 2 .469989166 61 . 17508781 1360 .046447 1 1400 .03521 62 .63174994 20 . 104164 16186 .93288 8636 . 185689 20 .25777989 1001 .681219 94 .79151718 921 .8766929 206 .0877497 5 . 122053265 5 .351766266 615 .5918495 1235 .967601 2709 .302398 136 .3231414 37 . 17769973 8 .201793436 6 .092070276 4 .833850889 2 .060793151 5 .826625385 31 .67837486 5 .595222478 2721 . 155316 7 .434068874 6 .939392507 36 .461765 38 .78365447 26 . 14644014 47 .42246581 3252 .651809 89 .84473901

C6

C6orf1 C6orf10 C6orf106 C6orf1 18 C6orf120 C6orf132 C6orf136 C6orf141 C6orf15 C6orf163 C6orf201 C6orf203 C6orf222 C6orf223 C6orf226 C6orf229 C6orf25 C6orf47 C6orf48 C6orf52 C6orf58 C6orf62 C6orf89 C6orf99

C7

C7orf25 C7orf26 C7orf31 C7orf33 C7orf34 C7orf43 C7orf49 C7orf50 C7orf55 C7orf57 C7orf61 C7orf62 C7orf65 C7orf66 C7orf69 C7orf71 C7orf72 C7orf73

C8A

C8B

C8G

C8orf17 C8orf22 C8orf31 C8orf33 C8orf34

C8orf34-AS1 9 .051865571

C8orf37 C8orf4 C8orf44

497 .0602737 451 .0566383 419 .2118749

-1 .031466203

-0 .389244684

-1 .649913043

0 .462390331

-3 .052527346

0 .084604892

0 .063697591

0 . 121351897

-0 .555766788

0 .007118974

-1 . 123145509

-1 .001834424

-0 .634489709

-2 .493623277

0 . 169304248

-0 .030423397

-1 . 150727686

-0 .548841053

0 .268288509

-1 .063225052

-0 .545364958

-1 .348307012

0 .838205452

0 .292823551

-1 .215457075

1 .338335279

-0 .565516551

0 .605434811

-0 .509068809

-1 .218295207

-0 .222186097

0 .472163256

0 .356214032

0 .721708864

0 . 165153794

0 . 137070351

-0 .459838712

-1 .337516607

-3 .430286412

-2 .263978355

-1 .039385655

-0 .950570009

-5 .457506325

0 .015277838

-5 .270941807

-4 .338188379

0 .505094106

-0 .220325523

-0 .592309646

0 .550098622

0 .543605202

-1 .414818109

-2 .08326942

0 .06271146

1 . 182667639

-0 .372694736

0 .40283 0 . 1499 0 .31495 0 .07846 0 .55562 0 .0948 0 .24742 0 .08884 0 .24224 0 .32671 0 . 1884 0 . 18888 0 .09969 0 .36323 0 .36999 0 . 15141 0 .47337 0 .26189 0 .0767 0 . 18706 0 . 16114 0 .2881 0 .07988 0 .06503 0 .24631 0 .37775 0 . 14806 0 .06852 0 . 13653 0 .60676 0 .47675 0 . 10802 0 . 10216 0 . 1341 0 .21378 0 .24775 0 .27232 0 .54664 0 .68202 0 .77995 0 .50957 0 .21231 0 .77627 0 . 10366 0 .76775 0 .84835 0 . 16646 0 .22513 0 .3978 0 .2266 0 . 1 1685 0 .30421 0 .4866 0 . 10841 0 . 17097 0 . 14836

-2 .5606 -2 .5967 -5 .2386 5 .89316 -5 .4939 0 .89243 0 .25745 1 .36598 -2 .2943 0 .02179 -5 .9614 -5 .304 -6 .3646 -6 .8652 0 .45759 -0 .2009 -2 .4309 -2 .0957 3 .49785 -5 .6838 -3 .3844 -4 .68 10 .4931 4 .50304 -4 .9347 3 .54289 -3 .8196 8 .83613 -3 .7287 -2 .0079 -0 .466 4 .37106 3 .48693 5 .38201 0 .77253 0 .55327 -1 .6886 -2 .4468 -5 .0296 -2 .9027 -2 .0397 -4 .4773 -7 .0305 0 . 14739 -6 .8655 -5 . 1 137 3 .03438 -0 .9787 -1 .489 2 .4276 4 .65202 -4 .6508 -4 .2813 0 .57848 6 .91744 -2 .5121

0 .01045 0 .00941 1 .62E-07 3 .79E-09 3 .93E-08 0 .37216 0 .79683 0 . 17194 0 .02177 0 .98262 2 .50E-09 1 . 13E-07 1 .96E-10 6 .64E-12 0 .64725 0 .84075 0 .01506 0 .03611 0 .00047 1 .32E-08 0 .00071 2 .87E-06 9 .30E-26 6 .70E-06 8 .03E-07 0 .0004 0 .00013 9 .91E-19 0 .00019 0 .04466 0 .64118 1 .24E-05 0 .00049 7 .37E-08 0 .4398 0 .58008 0 .0913 0 .01441 4 .92E-07 0 .0037 0 .04138 7 .56E-06 2 .06E-12 0 .88282 6 .63E-12 3 . 16E-07 0 .00241 0 .32774 0 . 1365 0 .0152 3 .29E-06 3 .31E-06 1 .86E-05 0 .56294 4 .60E-12 0 .012

0 .01655 0 .01505 5 .42E-07 1 .59E-08 1 .45E-07 0 .42811 0 .82697 0 .2154 0 .03267 0 .98513 1 .08E-08 3 .90E-07 9 .73E-10 3 .96E-1 1 0 .69372 0 .86547 0 .02318 0 .05191 0 .00093 5 . 17E-08 0 .00138 7 .97E-06 2 .65E-24 1 .77E-05 2 .43E-06 0 .00079 0 .00029 1 .32E-17 0 .00041 0 .06314 0 .68825 3 . 14E-05 0 .00097 2 .61E-07 0 .49569 0 .63139 0 . 12142 0 .02226 1 .54E-06 0 .00637 0 .05884 1 .97E-05 1 .31E-1 1 0 .90172 3 .95E-1 1 1 .02E-06 0 .00428 0 .38266 0 . 17469 0 .02338 9 .05E-06 9 . 10E-06 4 .61E-05 0 .61521 2 .80E-1 1 0 .01881

C8orf44-SGK8 .065950139

C8orf46 C8orf48 C8orf49 C8orf58 C8orf59 C8orf59P1 C8orf74 C8orf76 C8orf82 C8orf86 C8orf88

C9

C9orf1 14 C9orf1 16 C9orf129 C9orf131 C9orf135 C9orf139 C9orf142 C9orf147 C9orf152 C9orf153 C9orf16 C9orf163 C9orf170 C9orf172 C9orf24 C9orf3 C9orf40 C9orf43 C9orf47 C9orf50 C9orf57 C9orf62 C9orf64 C9orf66 C9orf69 C9orf72 C9orf78 C9orf84 C9orf85 C9orf91 C9orf92

CA1

CA10

CA11

CA12

CA13

CA14

CA2

CA3

CA4

CA5A

CA5B

CA6

836 .9461505 501 .2590674 4 . 17612916 514 .8887121 1400 .523482 1 .460915624 50 . 12104423 357 .2616613 885 . 1066248 6 .354884017 392 .6932271 139 .936805 1294 .634242 65 .01742533 2 . 136626044 263 .0457332 8 .793205349 53 .68755999 967 .9099542 47 .59237147 41 .55740429 23 .58433977 2051 .098239 54 .9307791 81 .59445713 49 .84239987 21 . 15293286 6426 . 137705 286 . 1233586 61 .78839149 38 .97480277 25 .74778883 6 .49854355 1 .965129769 1 1 18 .630456 93 .0075443 2455 .671542 2934 .080375 2756 .548693 578 . 1 192662 1279 .3915 1397 .099447 4 .612787917 188 .905269 16 .93148744 280 .3317516 5365 .31025 935 .5944432 839 .2986027 2223 .504258 103 .8130206 50 .99955189 3 . 188962412 4206 .58502 591 .4045059

-0 .48639817

1 .468569057

-0 .726203889

-1 .933853507

0 . 177010463

-0 .490156013

-2 .880862751

-0 .087017411

0 .511350788

0 .767663489

-2 . 17251905

-0 .941465387

-1 .272058473

-0 .09448964

1 .083445188

-0 .420661511

-0 .23925422

-4 .027536597

0 .963066858

0 .317539174

-1 .070895318

-0 .255948177

-1 .688415212

0 . 168707386

0 .2486432

-1 .222392968

0 .806091314

-0 .830587105

-1 . 195107085

0 .232780529

-1 . 1 12846554

-0 .602683874

-0 .605750222

-2 .403862159

-2 .98621694

0 .267055852

-1 .015525062

0 .910042838

-0 .652459128

0 .214757876

-0 .536921669

-0 .635695516

0 .47526755

-1 .286990652

-1 .601403938

-2 .098064204

0 . 1 10771577

-0 .598253108

-0 .880753345

1 .644428817

-1 .324990132

-1 .446544684

1 .69827256

-1 .955235911

-1 .511 1 16526

0 .941794078

0 .27477 -1 .7702

0 .3483 4 .21643

0 .22762 -3 . 1904

0 .82055 -2 .3568

0 .0966 1 .83248

0 . 12974 -3 .7781

0 .64488 -4 .4673

0 .28905 -0 .3011

0 . 10813 4 .72898

0 . 14182 5 .41292

0 .44673 -4 .8632

0 . 14466 -6 .508

0 .29259 -4 .3475

0 .09205 -1 .0265

0 .22779 4 .75629

0 .51 -0 .8248

0 .20306 -1 . 1782

0 .56655 -7 . 1089

0 .21579 4 .46289

0 . 10677 2 .97396

0 . 1846 -5 .8012

0 .34463 -0 .7427

0 .24457 -6 .9036

0 . 1745 0 .96681

0 . 1423 1 .74734

0 .20712 -5 .9019

0 . 18524 4 .35171

0 .24224 -3 .4288

0 . 14834 -8 .0563

0 . 12845 1 .81223

0 . 19184 -5 .8008

0 .24385 -2 .4715

0 .2372 -2 .5538

0 .39506 -6 .0848

0 .78742 -3 .7924

0 .08921 2 .99364

0 . 1642 -6 . 1846

0 . 10073 9 .03475

0 . 1254 -5 .2028

0 .09924 2 . 16401

0 . 19349 -2 .775

0 .08573 -7 .4155

0 . 1 1566 4 . 10908

0 .40717 -3 . 1608

0 .26613 -6 .0173

0 .46362 -4 .5254

0 . 10548 1 .05018

0 .20021 -2 .9881

0 . 17952 -4 .9062

0 .27298 6 .02405

0 .20862 -6 .3511

0 .28806 -5 .0217

0 .34523 4 .9193

0 .53646 -3 .6447

0 . 1425 -10 .604

0 .43205 2 . 17983

0 .0767 2 .48E-05 0 .00142 0 .01843 0 .06688 0 .00016 7 .92E-06 0 .76338 2 .26E-06 6 .20E-08 1 . 15E-06 7 .61E-1 1 1 .38E-05 0 .30465 1 .97E-06 0 .40947 0 .2387 1 . 17E-12 8 .09E-06 0 .00294 6 .58E-09 0 .45767 5 .07E-12 0 .33364 0 .08058 3 .59E-09 1 .35E-05 0 .00061 7 .87E-16 0 .06995 6 .60E-09 0 .01345 0 .01066 1 . 17E-09 0 .00015 0 .00276 6 .23E-10 1 .64E-19 1 .96E-07 0 .03046 0 .00552 1 .21E-13 3 .97E-05 0 .00157 1 .77E-09 6 .03E-06 0 .29363 0 .00281 9 .29E-07 1 .70E-09 2 . 14E-10 5 . 12E-07 8 .69E-07 0 .00027 2 .85E-26 0 .02927

0 . 10354 6 .03E-05 0 .00261 0 .028 0 .09126 0 .00034 2 .06E-05 0 .79694 6 .36E-06 2 .22E-07 3 .41E-06 3 .97E-10 3 .48E-05 0 .35866 5 .60E-06 0 .46599 0 .28875 7 .67E-12 2 . 10E-05 0 .00514 2 .70E-08 0 .51351 3 .07E-1 1 0 .38862 0 . 10821 1 .51E-08 3 .41E-05 0 .00118 7 .37E-15 0 .09505 2 .70E-08 0 .02089 0 .01685 5 .25E-09 0 .00032 0 .00484 2 .91E-09 2 .39E-18 6 .50E-07 0 .04446 0 .00923 8 .83E-13 9 .33E-05 0 .00287 7 .79E-09 1 .60E-05 0 .34712 0 .00492 2 .79E-06 7 .49E-09 1 .06E-09 1 .60E-06 2 .62E-06 0 .00055 8 .57E-25 0 .04286

| CA7 | 7 .034984473 | -0 .532044756 | 0 .39891 -1 .3337 | 0 . 18229 | 0 .22706 |
| --- | --- | --- | --- | --- | --- |
| CA8 | 530 .5269656 | 1 .347637518 | 0 .38678 3 .48425 | 0 .00049 | 0 .00097 |
| CA9 | 46 .07707147 | -1 .027130082 | 0 .30876 -3 .3267 | 0 .00088 | 0 .00167 |
| CAAP1 | 1975 .653408 | -0 .027122691 | 0 . 1 1067 -0 .2451 | 0 .80639 | 0 .83535 |
| CAB39 | 8950 .365844 | 0 .07784407 | 0 .07262 1 .07198 | 0 .28373 | 0 .33666 |
| CAB39L | 2244 .854827 | -0 .942198305 | 0 . 13891 -6 .7828 | 1 . 18E-1 1 | 6 .80E-1 1 |
| CABIN1 | 4115 .600581 | 0 .267972171 | 0 . 1041 2 .57419 | 0 .01005 | 0 .01598 |
| CABLES1 | 3818 .279938 | -0 .395581655 | 0 . 17732 -2 .2309 | 0 .02569 | 0 .03807 |
| CABLES2 | 286 .8930827 | 0 . 105947252 | 0 . 14496 0 .73085 | 0 .46487 | 0 .52067 |
| CABP1 | 37 .28738922 | -0 .616917947 | 0 .22262 -2 .7712 | 0 .00559 | 0 .00933 |
| CABP2 | 1 .390199855 | -2 .880603588 | 0 .89212 -3 .2289 | 0 .00124 | 0 .00231 |
| CABP4 | 52 . 19426876 | 1 .239428138 | 0 .23225 5 .33662 | 9 .47E-08 | 3 .30E-07 |
| CABP5 | 4 .527497213 | -4 .654254143 | 0 .82684 -5 .629 | 1 .81E-08 | 6 .99E-08 |
| CABP7 | 18 .02379848 | -0 . 1 19733561 | 0 .22832 -0 .5244 | 0 .6 | 0 .65049 |
| CABS1 | 5 .374557843 | -3 .063525299 | 1 . 10107 -2 .7823 | 0 .0054 | 0 .00903 |
| CABYR | 174 .788604 | -0 .643803409 | 0 . 14917 -4 .3158 | 1 .59E-05 | 3 .97E-05 |
| CACFD1 | 645 .4949776 | 0 .545123585 | 0 . 10037 5 .43125 | 5 .60E-08 | 2 .01E-07 |
| CACHD1 | 2550 .937958 | -0 .294616519 | 0 . 1476 -1 .9961 | 0 .04593 | 0 .06477 |
| CACNA1A | 56 .48555099 | -0 .704252089 | 0 .29309 -2 .4029 | 0 .01627 | 0 .02491 |
| CACNA1B | 65 .92230005 | -1 .569281405 | 0 .32037 -4 .8983 | 9 .67E-07 | 2 .89E-06 |
| CACNA1C | 635 .0497423 | 1 .486830874 | 0 . 19577 7 .59485 | 3 .08E-14 | 2 .42E-13 |
| CACNA1D | 563 .8899008 | 1 . 164553347 | 0 .24594 4 .73519 | 2 . 19E-06 | 6 . 18E-06 |
| CACNA1E | 81 .61747019 | -1 .018664334 | 0 .34757 -2 .9308 | 0 .00338 | 0 .00586 |
| CACNA1F | 45 .47752699 | -1 .245120235 | 0 .21549 -5 .7781 | 7 .55E-09 | 3 .07E-08 |
| CACNA1G | 168 .6025365 | 0 .234618617 | 0 .22573 1 .03936 | 0 .29864 | 0 .35232 |
| CACNA1H | 418 .8218973 | 0 .608929316 | 0 .28297 2 . 15192 | 0 .0314 | 0 .04571 |
| CACNA1I | 40 .60928379 | -0 . 171210529 | 0 .30983 -0 .5526 | 0 .58054 | 0 .63175 |
| CACNA1S | 15 .09709416 | -2 .574228124 | 0 .43365 -5 .9362 | 2 .92E-09 | 1 .25E-08 |
| CACNA2D1 | 2302 .83039 | -0 .412582697 | 0 . 1967 -2 .0976 | 0 .03594 | 0 .05168 |
| CACNA2D2 | 102 .994609 | -0 .374329127 | 0 .20764 -1 .8028 | 0 .07142 | 0 .09688 |
| CACNA2D3 | 172 .3336916 | 0 . 159790097 | 0 . 19458 0 .82122 | 0 .41152 | 0 .46808 |
| CACNA2D4 | 285 .9898866 | 0 .318148646 | 0 . 14192 2 .24175 | 0 .02498 | 0 .03708 |
| CACNB1 | 305 .9298118 | 0 . 124952534 | 0 . 1572 0 .79487 | 0 .42669 | 0 .48323 |
| CACNB2 | 450 .0586486 | -0 .726497212 | 0 . 18221 -3 .987 | 6 .69E-05 | 0 .00015 |
| CACNB3 | 650 .0078637 | 0 .283885164 | 0 . 1 1792 2 .40739 | 0 .01607 | 0 .02461 |
| CACNB4 | 1573 . 174432 | -1 .952109647 | 0 .2421 -8 .0632 | 7 .43E-16 | 7 .00E-15 |
| CACNG1 | 5 .292389754 | -0 .383917357 | 0 .35396 -1 .0846 | 0 .27808 | 0 .33079 |
| CACNG2 | 7 .2614311 15 | -4 .39195136 | 0 .69878 -6 .2852 | 3 .27E-10 | 1 .58E-09 |
| CACNG3 | 4 .890152203 | -4 .335277528 | 0 .73199 -5 .9226 | 3 . 17E-09 | 1 .35E-08 |
| CACNG4 | 13 .72314767 | -0 . 122948855 | 0 .38915 -0 .3159 | 0 .75205 | 0 .78709 |
| CACNG5 | 3 .0799078 | -2 .43130481 | 0 .73552 -3 .3055 | 0 .00095 | 0 .0018 |
| CACNG6 | 3 .622520224 | -1 .574333586 | 0 .69822 -2 .2548 | 0 .02415 | 0 .03595 |
| CACNG7 | 4 .53709527 | -3 .559385631 | 0 .75856 -4 .6923 | 2 .70E-06 | 7 .53E-06 |
| CACNG8 | 22 .0046913 | -1 .95263436 | 0 .4863 -4 .0153 | 5 .94E-05 | 0 .00014 |
| CACTIN | 752 .8887014 | 0 .905691497 | 0 .0867 10 .4464 | 1 .52E-25 | 4 .24E-24 |
| CACUL1 | 6386 .556054 | 0 .278176978 | 0 .08313 3 .34633 | 0 .00082 | 0 .00157 |
| CACYBP | 4740 .678461 | 0 .009996686 | 0 . 13518 0 .07395 | 0 .94105 | 0 .95081 |
| CAD | 3961 .409445 | 0 .524773608 | 0 . 1 1 184 4 .69225 | 2 .70E-06 | 7 .53E-06 |
| CADM1 | 7127 .431624 | -1 .351733298 | 0 . 19406 -6 .9657 | 3 .27E-12 | 2 .03E-1 1 |
| CADM2 | 295 .6053132 | -1 .395795928 | 0 .39145 -3 .5657 | 0 .00036 | 0 .00073 |
| CADM3 | 422 .3628959 | 0 .727391298 | 0 .32981 2 .2055 | 0 .02742 | 0 .04039 |
| CADM4 | 1 137 .063046 | 0 .47283788 | 0 . 1949 2 .42611 | 0 .01526 | 0 .02347 |
| CADPS | 1 196 .213048 | 1 .543007953 | 0 .31995 4 .82271 | 1 .42E-06 | 4 . 1 1E-06 |
| CADPS2 | 3820 .696974 | -0 .973363796 | 0 .21517 -4 .5238 | 6 .07E-06 | 1 .61E-05 |
| CAGE1 | 22 .76643454 | -2 .445813986 | 0 .26585 -9 .2001 | 3 .58E-20 | 5 .59E-19 |
| CALB1 | 158 .9180529 | -1 .41108659 | 0 .20695 -6 .8184 | 9 .20E-12 | 5 .37E-1 1 |

| CALB2 153 .2597834 | 1 .357181382 | 0 .308 | 4 .40641 | 1 .05E-05 | 2 .69E-05 |
| --- | --- | --- | --- | --- | --- |
| CALCA 9 . 101700025 | -0 .466724141 | 0 .37125 | -1 .2572 | 0 .20869 | 0 .25602 |
| CALCB 24 .82841948 | -1 .088740364 | 0 .30929 | -3 .5201 | 0 .00043 | 0 .00086 |
| CALCOCO1 7038 .920379 | -0 .816743594 | 0 .09884 | -8 .2634 | 1 .42E-16 | 1 .46E-15 |
| CALCOCO2 3992 .409355 | -0 .269987563 | 0 .09605 | -2 .8108 | 0 .00494 | 0 .00832 |
| CALCR 71 .20954697 | 0 .343897299 | 0 .34958 | 0 .98373 | 0 .32525 | 0 .38012 |
| CALCRL 3580 .752746 | 0 .435599769 | 0 . 14971 | 2 .90967 | 0 .00362 | 0 .00624 |
| CALD1 17137 .53435 | 0 .788630271 | 0 . 1 1836 | 6 .6632 | 2 .68E-1 1 | 1 .49E-10 |
| CALHM1 13 .3459085 | -0 .381152767 | 0 .24791 | -1 .5375 | 0 . 12417 | 0 . 16034 |
| CALHM2 928 .7598598 | 0 .387628228 | 0 . 1 1 197 | 3 .46192 | 0 .00054 | 0 .00105 |
| CALHM3 3 .415451891 | -1 .287967184 | 0 .5822 | -2 .2122 | 0 .02695 | 0 .03977 |
| CALM1 27058 .58105 | -0 . 10554748 | 0 .08724 | -1 .2099 | 0 .22632 | 0 .27524 |
| CALM2 36185 .82685 | -0 .079156977 | 0 . 12091 | -0 .6547 | 0 .51267 | 0 .56713 |
| CALM3 4493 .27234 | 0 .362422591 | 0 . 10472 | 3 .46075 | 0 .00054 | 0 .00106 |
| CALML3 3153 .582637 | 1 .460353157 | 0 .31748 | 4 .59986 | 4 .23E-06 | 1 . 15E-05 |
| CALML4 1 125 .994035 | -0 .34095373 | 0 .08273 | -4 . 1214 | 3 .77E-05 | 8 .87E-05 |
| CALML5 39220 .46324 | -0 .556577216 | 0 .28576 | -1 .9477 | 0 .05145 | 0 .0718 |
| CALML6 17 .63192812 | -0 .062649511 | 0 .25728 | -0 .2435 | 0 .80761 | 0 .83653 |
| CALN1 207 .0964339 | -1 .613663405 | 0 .39943 | -4 .0399 | 5 .35E-05 | 0 .00012 |
| CALR 20296 .27317 | 0 .954367077 | 0 .09936 | 9 .60532 | 7 .59E-22 | 1 .41E-20 |
| CALR3 4 .922835182 | -1 .905931389 | 0 .37452 | -5 .089 | 3 .60E-07 | 1 . 15E-06 |
| CALU 19396 .07819 | 1 .542529997 | 0 . 17685 | 8 .72204 | 2 .73E-18 | 3 .44E-17 |
| CALY 6 .641995432 | 0 . 104097071 | 0 .49024 | 0 .21234 | 0 .83184 | 0 .85787 |
| CAMK1 140 .0777531 | 0 .534083075 | 0 . 13881 | 3 .8477 | 0 .00012 | 0 .00026 |
| CAMK1D 2957 .991872 | -0 .216120017 | 0 . 1808 | -1 . 1954 | 0 .23195 | 0 .2814 |
| CAMK1G 23 .56319722 | -0 .349854715 | 0 .30226 | -1 . 1575 | 0 .24708 | 0 .29767 |
| CAMK2A 67 .7685746 | 0 .246146802 | 0 .28791 | 0 .85493 | 0 .39259 | 0 .44902 |
| CAMK2B 59 .07595841 | 0 .093888227 | 0 .32498 | 0 .28891 | 0 .77265 | 0 .80505 |
| CAMK2D 6578 .852522 | 0 .209183546 | 0 .09311 | 2 .24655 | 0 .02467 | 0 .03666 |
| CAMK2G 1608 .797364 | -0 .049532467 | 0 .09585 | -0 .5168 | 0 .60532 | 0 .65522 |
| CAMK2N1 583 .2724811 | 1 .526436689 | 0 . 15234 | 10 .02 | 1 .25E-23 | 2 .79E-22 |
| CAMK2N2 3 .536182558 | 1 .208070437 | 0 .35082 | 3 .44354 | 0 .00057 | 0 .00112 |
| CAMK4 915 .2550452 | 0 .950011748 | 0 .23002 | 4 . 13011 | 3 .63E-05 | 8 .56E-05 |
| CAMKK1 744 .5032318 | -0 .361712576 | 0 . 1 1967 | -3 .0227 | 0 .00251 | 0 .00443 |
| CAMKK2 2850 .791615 | 0 .418150607 | 0 .0558 | 7 .49359 | 6 .70E-14 | 5 .02E-13 |
| CAMKMT 538 .8637059 | -0 .632885437 | 0 . 1 1458 | -5 .5237 | 3 .32E-08 | 1 .23E-07 |
| CAMKV 15 .37201975 | 0 . 197274653 | 0 .37945 | 0 .51989 | 0 .60314 | 0 .6533 |
| CAMLG 3355 .585976 | -1 . 131273288 | 0 . 12661 | -8 .9354 | 4 .06E-19 | 5 .59E-18 |
| CAMP 14 .96198052 | 1 .280780957 | 0 .35451 | 3 .61284 | 0 .0003 | 0 .00062 |
| CAMSAP1 3661 .45964 | 0 .022753751 | 0 .076 | 0 .29938 | 0 .76465 | 0 .79819 |
| CAMSAP2 8617 .843914 | -0 . 182043571 | 0 .08126 | -2 .2402 | 0 .02508 | 0 .03721 |
| CAMSAP3 492 . 1676174 | -0 .31030054 | 0 .29868 | -1 .0389 | 0 .29885 | 0 .35255 |
| CAMTA1 1930 .306305 | -0 .057287543 | 0 .09132 | -0 .6273 | 0 .53045 | 0 .5842 |
| CAMTA2 563 .5599836 | 0 .461129925 | 0 . 14574 | 3 . 16399 | 0 .00156 | 0 .00284 |
| CAND1 18336 .31548 | -0 . 148277492 | 0 .08995 | -1 .6485 | 0 .09925 | 0 . 13073 |
| CAND2 396 .0661556 | 0 . 162941856 | 0 .20197 | 0 .80675 | 0 .41981 | 0 .47647 |
| CANT1 2009 .250551 | 0 .913298273 | 0 .0766 | 1 1 .9231 | 8 .97E-33 | 5 .27E-31 |
| CANX 63072 .04994 | 0 .381986085 | 0 . 101 | 3 .78217 | 0 .00016 | 0 .00033 |
| CAP1 20511 . 1 159 | 0 .720647068 | 0 . 10814 | 6 .66427 | 2 .66E-1 1 | 1 .48E-10 |
| CAP2 363 . 1329728 | 1 .614538005 | 0 .23767 | 6 .79323 | 1 . 10E-1 1 | 6 .35E-1 1 |
| CAPG 1 1978 .92995 | 1 .408182724 | 0 .21594 | 6 .52117 | 6 .98E-1 1 | 3 .65E-10 |
| CAPN1 6077 .613215 | 0 .264702535 | 0 .09773 | 2 .70858 | 0 .00676 | 0 .011 1 |
| CAPN10 1211 .574054 | 0 .57242661 | 0 .08647 | 6 .61987 | 3 .60E-1 1 | 1 .96E-10 |
| CAPN11 81 .50328255 | -0 .454867712 | 0 . 17508 | -2 .598 | 0 .00938 | 0 .015 |
| CAPN12 77 . 1 1743619 | 0 . 173690567 | 0 .22429 | 0 .77439 | 0 .4387 | 0 .49466 |
| CAPN13 41 .8070318 | 0 . 1 13045922 | 0 .50933 | 0 .22195 | 0 .82435 | 0 .85157 |

| CAPN14 | 120 .4959838 | -0 .472737405 | 0 .21374 | -2 .2117 | 0 .02698 | 0 .03982 |
| --- | --- | --- | --- | --- | --- | --- |
| CAPN15 | 1606 .760399 | 1 .344784326 | 0 . 10235 | 13 . 1386 | 1 .98E-39 | 2 . 18E-37 |
| CAPN2 | 13547 .20237 | 0 .718564428 | 0 .0796 | 9 .02741 | 1 .76E-19 | 2 .55E-18 |
| CAPN3 | 862 .9993877 | 0 .428817267 | 0 .24199 | 1 .77202 | 0 .07639 | 0 . 10318 |
| CAPN5 | 854 .401943 | 0 .460176475 | 0 . 16519 | 2 .78575 | 0 .00534 | 0 .00894 |
| CAPN6 | 138 .44374 | -1 .228964363 | 0 .32827 | -3 .7438 | 0 .00018 | 0 .00038 |
| CAPN7 | 4707 .887309 | -0 .392401385 | 0 .06266 | -6 .2628 | 3 .78E-10 | 1 .81E-09 |
| CAPN8 | 78 .23594671 | 0 .359771896 | 0 .411 15 | 0 .87504 | 0 .38155 | 0 .43778 |
| CAPN9 | 22 .90581357 | -1 .434859063 | 0 .25098 | -5 .7171 | 1 .08E-08 | 4 .30E-08 |
| CAPNS1 | 3225 .752119 | 0 .977119638 | 0 . 10545 | 9 .26657 | 1 .92E-20 | 3 . 10E-19 |
| CAPNS2 | 2447 .423162 | -1 .397108617 | 0 .24646 | -5 .6688 | 1 .44E-08 | 5 .62E-08 |
| CAPRIN1 | 19921 .37318 | 0 .024781048 | 0 .05915 | 0 .41894 | 0 .67526 | 0 .71908 |
| CAPRIN2 | 2010 .036647 | -0 .568585559 | 0 . 10092 | -5 .6339 | 1 .76E-08 | 6 .81E-08 |
| CAPS | 580 .610192 | 0 . 129600605 | 0 . 16399 | 0 .7903 | 0 .42935 | 0 .48586 |
| CAPS2 | 740 .0269657 | -0 .861545351 | 0 . 14747 | -5 .842 | 5 . 16E-09 | 2 . 14E-08 |
| CAPSL | 4 .961490901 | -3 .76321877 | 0 .71687 | -5 .2495 | 1 .53E-07 | 5 . 14E-07 |
| CAPZA1 | 13285 .48994 | 0 . 189986132 | 0 .08348 | 2 .27573 | 0 .02286 | 0 .03417 |
| CAPZA2 | 12975 .9613 | 0 .20576456 | 0 . 13892 | 1 .48123 | 0 . 13855 | 0 . 17706 |
| CAPZA3 | 17 .6301223 | -2 .294605716 | 0 .45239 | -5 .0722 | 3 .93E-07 | 1 .25E-06 |
| CAPZB | 6003 .211233 | 1 .221784944 | 0 .08153 | 14 .9863 | 9 .03E-51 | 2 .93E-48 |
| CARD10 | 604 . 1984514 | -0 .05046228 | 0 . 16235 | -0 .3108 | 0 .75593 | 0 .79034 |
| CARD11 | 344 .0092204 | 2 .58994209 | 0 .22939 | 1 1 .2905 | 1 .46E-29 | 6 . 13E-28 |
| CARD14 | 730 .9166719 | 0 .073223633 | 0 . 16007 | 0 .45745 | 0 .64735 | 0 .69379 |
| CARD16 | 556 .8354689 | -0 .300172262 | 0 .22669 | -1 .3242 | 0 . 18545 | 0 .23063 |
| CARD17 | 54 .76568898 | -0 .976376672 | 0 .30245 | -3 .2282 | 0 .00125 | 0 .00232 |
| CARD18 | 948 .9700623 | -1 .418949534 | 0 .37309 | -3 .8032 | 0 .00014 | 0 .00031 |
| CARD19 | 1483 .670562 | 0 .325782105 | 0 . 13695 | 2 .37877 | 0 .01737 | 0 .02647 |
| CARD6 | 999 .8282651 | -0 . 170522082 | 0 . 10395 | -1 .6404 | 0 . 10092 | 0 . 13281 |
| CARD8 | 3228 .934753 | 0 .362020357 | 0 .09436 | 3 .83678 | 0 .00012 | 0 .00027 |
| CARD9 | 335 .9046981 | 0 .725026036 | 0 . 15217 | 4 .7645 | 1 .89E-06 | 5 .40E-06 |
| CARF | 1845 .085182 | -0 .788967311 | 0 . 1 1 1 14 | -7 .0988 | 1 .26E-12 | 8 .23E-12 |
| CARHSP1 | 1492 .670571 | 0 .745321047 | 0 .09919 | 7 .51429 | 5 .72E-14 | 4 .34E-13 |
| CARM1 | 1485 .094327 | 1 . 139380741 | 0 .07089 | 16 .0734 | 3 .92E-58 | 2 .80E-55 |
| CARMIL2 | 160 .633277 | 1 .584893048 | 0 .21879 | 7 .24377 | 4 .36E-13 | 3 .00E-12 |
| CARMIL3 | 237 .6384174 | -0 .31541277 | 0 . 18771 | -1 .6803 | 0 .0929 | 0 . 12328 |
| CARNMT1 | 2860 . 123134 | -0 .063262346 | 0 . 10696 | -0 .5914 | 0 .55422 | 0 .60666 |
| CARNS1 | 133 .0973949 | 0 .040685676 | 0 . 18605 | 0 .21868 | 0 .8269 | 0 .85369 |
| CARS | 1825 . 10969 | 0 .03471434 | 0 .06698 | 0 .51826 | 0 .60428 | 0 .65417 |
| CARS2 | 3359 .823372 | -0 .036796348 | 0 .09457 | -0 .3891 | 0 .69721 | 0 .73909 |
| CARTPT | 3 .487753287 | -1 .68611 1936 | 0 .54843 | -3 .0744 | 0 .00211 | 0 .00377 |
| CASC1 | 75 .24160236 | -1 .978130134 | 0 .20324 | -9 .7328 | 2 . 18E-22 | 4 .30E-21 |
| CASC10 | 153 .9248784 | -0 .408721712 | 0 . 128 | -3 . 1932 | 0 .00141 | 0 .00259 |
| CASC3 | 3667 .642134 | 0 .00458588 | 0 .07715 | 0 .05944 | 0 .9526 | 0 .96006 |
| CASC4 | 7425 .632955 | 0 .071076139 | 0 . 10176 | 0 .69845 | 0 .4849 | 0 .53999 |
| CASC5 | 1606 .884054 | 1 .647999617 | 0 . 16486 | 9 .99656 | 1 .58E-23 | 3 .50E-22 |
| CASD1 | 5229 .788201 | -0 .755033648 | 0 . 14297 | -5 .2811 | 1 .28E-07 | 4 .39E-07 |
| CASK | 4196 .456794 | 0 .352619217 | 0 .09904 | 3 .5605 | 0 .00037 | 0 .00075 |
| CASKIN1 | 76 .87247058 | 0 .435842619 | 0 . 19701 | 2 .21232 | 0 .02694 | 0 .03977 |
| CASKIN2 | 740 .5631436 | 0 .831462289 | 0 . 12254 | 6 .78507 | 1 . 16E-1 1 | 6 .70E-1 1 |
| CASP1 | 2296 .916104 | -0 . 163039833 | 0 . 16193 | -1 .0069 | 0 .31399 | 0 .36847 |
| CASP10 | 1276 .27535 | 0 .88273147 | 0 . 12469 | 7 .07918 | 1 .45E-12 | 9 .43E-12 |
| CASP12 | 318 .256124 | -4 .332578286 | 0 .30131 | -14 .379 | 7 .00E-47 | 1 .58E-44 |
| CASP14 | 9033 .592258 | -0 .055392449 | 0 .30001 | -0 . 1846 | 0 .85352 | 0 .87645 |
| CASP16P | 47 .91775676 | -0 .351617844 | 0 . 19019 | -1 .8488 | 0 .06449 | 0 .08826 |
| CASP2 | 1981 .976699 | 0 .40351109 | 0 . 10734 | 3 .75909 | 0 .00017 | 0 .00036 |
| CASP3 | 3206 .442598 | -0 .092468881 | 0 . 1 1791 | -0 .7842 | 0 .43291 | 0 .48936 |

| CASP4 8382 .840488 | -0 .979096667 | 0 . 1 1343 | -8 .6316 | 6 .05E-18 | 7 .29E-17 |
| --- | --- | --- | --- | --- | --- |
| CASP5 35 .49636813 | 0 .204442917 | 0 .30254 | 0 .67576 | 0 .4992 | 0 .55407 |
| CASP6 466 .8399361 | 0 . 124100022 | 0 .07954 | 1 .56025 | 0 . 1 187 | 0 . 15397 |
| CASP7 2348 .661795 | -0 . 121172579 | 0 . 10536 | -1 . 15 | 0 .25012 | 0 .30103 |
| CASP8 2346 .037407 | 0 .595143123 | 0 .08765 | 6 .79023 | 1 . 12E-1 1 | 6 .47E-1 1 |
| CASP9 450 .9140879 | 0 .6963052 | 0 . 1 1523 | 6 .04274 | 1 .52E-09 | 6 .72E-09 |
| CASQ1 25 . 14864732 | -0 .274736121 | 0 .26629 | -1 .0317 | 0 .3022 | 0 .356 |
| CASQ2 208 . 1675068 | 1 .654488621 | 0 .26811 | 6 . 17091 | 6 .79E-10 | 3 . 16E-09 |
| CASR 10 . 15403255 | -3 .459905882 | 0 .51234 | -6 .7531 | 1 .45E-1 1 | 8 .28E-1 1 |
| CASS4 203 .0169157 | 0 .87215478 | 0 . 17362 | 5 .02322 | 5 .08E-07 | 1 .58E-06 |
| CAST 16278 .95209 | -0 .266620394 | 0 .08547 | -3 . 1 195 | 0 .00181 | 0 .00327 |
| CASZ1 2021 .087363 | -0 . 109289769 | 0 .25452 | -0 .4294 | 0 .66763 | 0 .71222 |
| CAT 8760 .972965 | -0 .373011719 | 0 . 10467 | -3 .5635 | 0 .00037 | 0 .00074 |
| CATIP 15 .59073054 | -1 .567508867 | 0 .26929 | -5 .8209 | 5 .85E-09 | 2 .41E-08 |
| CATSPER1 51 .09314607 | 0 .200298572 | 0 . 1809 | 1 . 10722 | 0 .2682 | 0 .3201 |
| CATSPER2 366 .8497102 | -0 .791659526 | 0 . 18988 | -4 . 1692 | 3 .06E-05 | 7 .31E-05 |
| CATSPER3 1 1 .31800876 | -0 .909313326 | 0 .21847 | -4 . 1622 | 3 . 15E-05 | 7 .52E-05 |
| CATSPER4 5 .06005424 | -2 .911981287 | 0 .58404 | -4 .986 | 6 . 17E-07 | 1 .90E-06 |
| CATSPERB 140 .0431248 | -0 .22847775 | 0 .21972 | -1 .0399 | 0 .29841 | 0 .35211 |
| CATSPERD 15 .67081455 | -1 .692277639 | 0 .32127 | -5 .2674 | 1 .38E-07 | 4 .70E-07 |
| CATSPERG 163 . 127563 | -0 .355163946 | 0 . 16649 | -2 . 1332 | 0 .03291 | 0 .04765 |
| CAV1 20407 .0422 | -0 .677584759 | 0 .09935 | -6 .8205 | 9 .08E-12 | 5 .30E-1 1 |
| CAV2 8707 . 195707 | -0 . 169144236 | 0 .09779 | -1 .7297 | 0 .08369 | 0 . 1 1211 |
| CAV3 1 1 .09756961 | -0 .81143918 | 0 .28813 | -2 .8162 | 0 .00486 | 0 .00819 |
| CBARP 80 . 1095931 | 1 .001398696 | 0 . 15543 | 6 .44262 | 1 . 17E-10 | 5 .99E-10 |
| CBFA2T2 2521 .878805 | -0 .035657472 | 0 .0966 | -0 .3691 | 0 .71203 | 0 .7528 |
| CBFA2T3 162 .5559504 | 1 .258397729 | 0 .22863 | 5 .50397 | 3 .71E-08 | 1 .37E-07 |
| CBFB 5125 .326639 | 0 .894693733 | 0 . 1 1413 | 7 .83904 | 4 .54E-15 | 3 .91E-14 |
| CBL 4739 .552057 | 0 .929163679 | 0 .08567 | 10 .8453 | 2 . 10E-27 | 7 . 13E-26 |
| CBLB 3838 . 142059 | -0 . 130041674 | 0 . 12921 | -1 .0065 | 0 .31419 | 0 .36864 |
| CBLC 574 .9999525 | -1 .051012968 | 0 .24816 | -4 .2352 | 2 .28E-05 | 5 .57E-05 |
| CBLL1 2479 .316655 | -0 . 1 15228806 | 0 .08659 | -1 .3307 | 0 . 18328 | 0 .22816 |
| CBLN1 47 .67725382 | 2 .067693957 | 0 .44608 | 4 .63522 | 3 .57E-06 | 9 .77E-06 |
| CBLN2 80 .43746524 | 0 .844359667 | 0 .38559 | 2 . 18977 | 0 .02854 | 0 .04187 |
| CBLN3 101 .3404814 | 0 .073264024 | 0 . 17585 | 0 .41663 | 0 .67695 | 0 .7206 |
| CBLN4 9 .896182361 | -1 .833947433 | 0 .52368 | -3 .502 | 0 .00046 | 0 .00092 |
| CBR1 4118 .984155 | -0 . 176438237 | 0 .09203 | -1 .9172 | 0 .05521 | 0 .07658 |
| CBR3 920 .3645257 | 0 .082571619 | 0 . 12753 | 0 .64749 | 0 .51731 | 0 .57154 |
| CBR4 3796 .77591 | -1 .438265392 | 0 . 12182 | -1 1 .807 | 3 .59E-32 | 1 .97E-30 |
| CBS 604 .2666257 | 0 .486296491 | 0 .27554 | 1 .76488 | 0 .07758 | 0 . 10459 |
| CBWD1 1899 .832669 | -1 .053773737 | 0 . 10065 | -10 .47 | 1 . 19E-25 | 3 .36E-24 |
| CBWD2 1600 .688329 | -0 .538841321 | 0 .06578 | -8 . 1916 | 2 .58E-16 | 2 .59E-15 |
| CBWD3 191 .4068199 | -0 .795361963 | 0 . 1385 | -5 .7427 | 9 .32E-09 | 3 .73E-08 |
| CBWD5 319 .6101266 | -0 .549271422 | 0 . 10595 | -5 . 1844 | 2 . 17E-07 | 7 . 14E-07 |
| CBWD7 7 . 1 18704496 | -0 .611220903 | 0 .28417 | -2 . 1509 | 0 .03148 | 0 .04581 |
| CBX1 2613 .566581 | 0 . 10063436 | 0 .07906 | 1 .27297 | 0 .20303 | 0 .24998 |
| CBX2 649 . 1888713 | 0 .38563091 | 0 . 14579 | 2 .64502 | 0 .00817 | 0 .01322 |
| CBX3 18396 .78528 | 0 .212123013 | 0 . 16587 | 1 .27886 | 0 .20094 | 0 .24764 |
| CBX4 1724 .024253 | 0 .817430209 | 0 . 1 1096 | 7 .36682 | 1 .75E-13 | 1 .26E-12 |
| CBX5 15059 . 14499 | 0 .0511 15306 | 0 . 10066 | 0 .50779 | 0 .6116 | 0 .66105 |
| CBX6 5868 .670719 | 0 .619797028 | 0 .09927 | 6 .24374 | 4 .27E-10 | 2 .03E-09 |
| CBX7 1983 .400665 | -0 .077475728 | 0 . 12753 | -0 .6075 | 0 .54351 | 0 .59656 |
| CBX8 613 .6625013 | 0 .612357147 | 0 . 1441 | 4 .24954 | 2 . 14E-05 | 5 .25E-05 |
| CBY1 694 .5162349 | -0 .200483682 | 0 .07916 | -2 .5325 | 0 .01132 | 0 .01783 |
| CBY3 15 .5304685 | -0 .312554925 | 0 . 18646 | -1 .6762 | 0 .09369 | 0 . 12421 |
| CC2D1A 933 .8047765 | 0 .78892454 | 0 . 13598 | 5 .80173 | 6 .56E-09 | 2 .69E-08 |

| CC2D1B CC2D2A CC2D2B CCAR1 CCAR2 CCBE1 CCDC102A CCDC102B CCDC103 CCDC105 CCDC106 CCDC107 CCDC109B CCDC110 CCDC112 CCDC113 CCDC114 CCDC115 CCDC116 CCDC117 CCDC12 CCDC120 CCDC121 CCDC122 CCDC124 CCDC125 CCDC126 CCDC127 CCDC129 CCDC13 CCDC130 CCDC134 CCDC136 CCDC137 CCDC138 CCDC14 CCDC140 CCDC141 CCDC142 CCDC144A | 3150 .076431 3320 .469597 55 .24397548 3872 .522062 2965 .089198 498 .6921459 353 .0257863 748 .4880988 0 . 144030662 3 .731285859 310 .5293095 197 .2549486 1833 .814366 324 .7221785 749 .5462202 411 .3769491 29 .34915542 2343 .281156 14 .56835204 2244 .788651 1066 .84238 450 .041587 488 .317863 701 .4188662 674 .7788005 1078 .821597 1572 .654098 1457 .445403 109 .9507479 95 .08757739 1284 .737203 396 .3609243 389 .0483862 859 .7951906 745 .2374688 8359 .096139 1 1 1 .7424106 781 .5044969 656 .7867557 939 .2431439 |
| --- | --- |

CCDC144NL 30 .4963762

| CCDC146 CCDC148 CCDC149 CCDC15 CCDC150 CCDC151 CCDC152 CCDC153 CCDC154 CCDC155 CCDC157 CCDC158 CCDC159 CCDC160 CCDC166 | 1078 .744607 135 .8548072 1387 .095007 651 .2444481 259 . 1031501 20 .24613127 266 .9505613 72 .68551844 208 .7529702 15 .40110204 171 . 1489883 45 .40801475 366 .7727664 26 .08277775 2 .415864707 |
| --- | --- |

0 .037266457

-1 . 149945997

-0 .63179558

-0 . 148410047

0 .427558251

-0 .078515959

0 .351106589

-0 .452197567

-0 .291210102

-2 .229925945

0 .947733652

0 .42603112

0 .47578109

-1 .797448734

-0 .230774007

-0 .446190352

-1 .342286172

-0 .287700179

-0 .822932974

0 .382209463

-0 .227662555

0 .235190712

-1 . 1 1 141444

-0 .748699942

0 .547982964

-0 .075108494

-0 .53899724

0 .07208799

0 .516594857

-1 .67567506

0 .069565001

0 .074403124

0 .00543263

0 .473565556

-0 .614502734

-0 .85522644

-1 .505089662

0 .263810189

-0 . 160286223

-0 .524083865

-1 .288232208

-1 .040751732

-1 .939012763

-0 .485020068

-0 .560128073

-0 .437800034

-1 .504099974

-0 .22028379

-0 .442569922

-1 .3011 18496

-1 . 169308222

0 .597438764

-1 .351018145

-0 .392736918

0 .395862237

0 .59328007

0 .08324 0 .4477

0 . 10995 -10 .459

0 . 1926 -3 .2803

0 .08141 -1 .8229

0 .09168 4 .66347

0 .25149 -0 .3122

0 . 1 1576 3 .03297

0 .21029 -2 . 1504

2 .65666 -0 . 1096

0 .5107 -4 .3664

0 . 12731 7 .44402

0 . 14249 2 .99001

0 . 12577 3 .78284

0 .20769 -8 .6545

0 . 13035 -1 .7704

0 . 14105 -3 . 1633

0 .2243 -5 .9843

0 .09924 -2 .8992

0 .25454 -3 .233

0 .09029 4 .23303

0 . 10267 -2 .2175

0 .20195 1 . 16461

0 . 1 1229 -9 .8974

0 . 1 191 -6 .2865

0 . 15949 3 .43581

0 .0891 -0 .843

0 . 1 1949 -4 .5109

0 .07 1 .02982

0 .35265 1 .46491

0 . 16057 -10 .436

0 .07403 0 .93974

0 .08571 0 .86812

0 .21418 0 .02536

0 .08426 5 .62045

0 . 10043 -6 . 1 188

0 . 14934 -5 .7266

0 .23271 -6 .4677

0 .21916 1 .20372

0 .09307 -1 .7222

0 .46098 -1 . 1369

0 .66687 -1 .9318

0 . 17248 -6 .034

0 . 18906 -10 .256

0 . 1 1245 -4 .3131

0 . 1 1506 -4 .8681

0 . 15134 -2 .8928

0 .25432 -5 .9141

0 . 18775 -1 . 1733

0 . 14737 -3 .0032

0 . 19079 -6 .8198

0 .47585 -2 .4573

0 .0952 6 .27536

0 .30881 -4 .3749

0 .0922 -4 .2597

0 .47377 0 .83556

0 .63839 0 .92934

0 .65437 1 .34E-25 0 .00104 0 .06832 3 . 1 1E-06 0 .75488 0 .00242 0 .03153 0 .91271 1 .26E-05 9 .77E-14 0 .00279 0 .00016 4 .95E-18 0 .07667 0 .00156 2 . 17E-09 0 .00374 0 .00123 2 .31E-05 0 .02659 0 .24418 4 .27E-23 3 .25E-10 0 .00059 0 .39922 6 .46E-06 0 .3031 0 . 14295 1 .70E-25 0 .34735 0 .38533 0 .97976 1 .90E-08 9 .43E-10 1 .02E-08 9 .95E-1 1 0 .2287 0 .08503 0 .25558 0 .05339 1 .60E-09 1 . 1 1E-24 1 .61E-05 1 . 13E-06 0 .00382 3 .34E-09 0 .24067 0 .00267 9 . 12E-12 0 .014 3 .49E-10 1 .21E-05 2 .05E-05 0 .4034 0 .35271

0 .70006 3 .75E-24 0 .00195 0 .09306 8 .59E-06 0 .78932 0 .0043 0 .04587 0 .92756 3 .20E-05 7 . 17E-13 0 .0049 0 .00033 6 .05E-17 0 . 10351 0 .00285 9 .46E-09 0 .00644 0 .00228 5 .63E-05 0 .03929 0 .29461 9 . 12E-22 1 .57E-09 0 .00115 0 .45583 1 .71E-05 0 .35699 0 . 18204 4 .73E-24 0 .40281 0 .4417 0 .98263 7 .32E-08 4 .30E-09 4 .08E-08 5 . 1 1E-10 0 .27785 0 . 1 1372 0 .30671 0 .07429 7 .07E-09 2 .84E-23 4 .02E-05 3 .33E-06 0 .00656 1 .41E-08 0 .29101 0 .0047 5 .33E-1 1 0 .02167 1 .68E-09 3 .09E-05 5 .04E-05 0 .45994 0 .40826

| CCDC167 | 763 .3916548 0 .610929266 | 0 . 16666 3 .66571 | 0 .00025 | 0 .00051 |
| --- | --- | --- | --- | --- |
| CCDC168 | 33 .07584718 -3 .049228296 | 0 .39804 -7 .6605 | 1 .85E-14 | 1 .49E-13 |
| CCDC169 | 46 .86202905 -0 .665929121 | 0 .29422 -2 .2634 | 0 .02361 | 0 .03521 |
| CCDC17 | 169 .717634 -0 .285055024 | 0 . 14744 -1 .9334 | 0 .05319 | 0 .07404 |
| CCDC170 | 342 .4653705 0 .534234443 | 0 . 17831 2 .99609 | 0 .00273 | 0 .00481 |
| CCDC171 | 1839 .74749 -0 .82377346 | 0 . 19285 -4 .2716 | 1 .94E-05 | 4 .80E-05 |
| CCDC172 | 30 .21308532 -0 . 15840867 | 0 .64466 -0 .2457 | 0 .8059 | 0 .83493 |
| CCDC173 | 97 .62025262 -1 .616226274 | 0 . 15104 -10 .701 | 1 .01E-26 | 3 . 18E-25 |
| CCDC174 | 1481 .871573 -0 .629185982 | 0 .07452 -8 .4431 | 3 .09E-17 | 3 .45E-16 |
| CCDC175 | 16 .87716929 -2 .200274632 | 0 .40033 -5 .4961 | 3 .88E-08 | 1 .43E-07 |
| CCDC177 | 3 .04805827 0 .442368957 | 0 .58468 0 .7566 | 0 .44929 | 0 .50514 |
| CCDC178 | 36 .01461359 -1 .502465774 | 0 .29964 -5 .0143 | 5 .32E-07 | 1 .65E-06 |
| CCDC179 | 25 .59631371 0 .790921267 | 0 .37199 2 . 12617 | 0 .03349 | 0 .04843 |
| CCDC18 | 946 .201237 -0 . 149823362 | 0 . 12512 -1 . 1974 | 0 .23115 | 0 .28062 |
| CCDC180 | 166 .5014363 -0 .94118494 | 0 .20465 -4 .599 | 4 .25E-06 | 1 . 15E-05 |
| CCDC181 | 123 .7412753 -0 .896437524 | 0 . 18079 -4 .9585 | 7 . 10E-07 | 2 . 17E-06 |
| CCDC182 | 2 .673727752 -2 .892506517 | 0 .69399 -4 . 1679 | 3 .07E-05 | 7 .35E-05 |
| CCDC183 | 38 .39453515 0 .229286828 | 0 . 14024 1 .63502 | 0 . 10205 | 0 . 13415 |
| CCDC184 | 27 .46758996 0 .258267348 | 0 .29846 0 .86532 | 0 .38686 | 0 .44314 |
| CCDC185 | 3 .845562488 -1 .563261009 | 0 .58163 -2 .6877 | 0 .00719 | 0 .01177 |
| CCDC186 | 4876 .78269 -0 .660300806 | 0 . 13905 -4 .7488 | 2 .05E-06 | 5 .80E-06 |
| CCDC188 | 49 .04647048 0 .836172621 | 0 . 19337 4 .32423 | 1 .53E-05 | 3 .84E-05 |
| CCDC189 | 28 . 18475734 0 .648280814 | 0 . 16357 3 .96344 | 7 .39E-05 | 0 .00017 |
| CCDC190 | 13 .4772204 -2 .470876629 | 0 .45948 -5 .3775 | 7 .55E-08 | 2 .67E-07 |
| CCDC191 | 632 . 1 148166 -1 .008294875 | 0 . 12943 -7 .7903 | 6 .69E-15 | 5 .63E-14 |
| CCDC22 | 1048 . 160229 -0 .254924834 | 0 .07392 -3 .4489 | 0 .00056 | 0 .0011 |
| CCDC24 | 177 .7576117 0 .379332443 | 0 . 14351 2 .64326 | 0 .00821 | 0 .01328 |
| CCDC25 | 3108 .529141 -0 . 144677364 | 0 .0948 -1 .5262 | 0 . 12697 | 0 . 16353 |
| CCDC27 | 9 .527793854 -1 .741837047 | 0 .3004 -5 .7984 | 6 .69E-09 | 2 .74E-08 |
| CCDC28A | 1 1 16 .272693 -0 .07731226 | 0 .09617 -0 .8039 | 0 .42145 | 0 .47798 |
| CCDC28B | 292 .9262689 0 .710236872 | 0 . 12198 5 .82258 | 5 .79E-09 | 2 .39E-08 |
| CCDC3 | 3734 .808465 -1 .471646835 | 0 .209 -7 .0412 | 1 .91E-12 | 1 .22E-1 1 |
| CCDC30 | 353 .9316658 -0 .959953143 | 0 . 13679 -7 .0177 | 2 .26E-12 | 1 .43E-1 1 |
| CCDC33 | 9 .864827824 -2 .886973641 | 0 .5518 -5 .2319 | 1 .68E-07 | 5 .61E-07 |
| CCDC34 | 500 .8435672 0 .0924009 | 0 . 125 0 .73919 | 0 .45979 | 0 .51556 |
| CCDC36 | 212 .6759907 0 . 148620938 | 0 .2102 0 .70703 | 0 .47955 | 0 .5349 |
| CCDC38 | 73 .33163898 -1 . 1 19419318 | 0 . 18647 -6 .0031 | 1 .94E-09 | 8 .46E-09 |
| CCDC39 | 1 173 . 101766 -1 .322291069 | 0 . 18985 -6 .965 | 3 .28E-12 | 2 .04E-1 1 |
| CCDC40 | 260 .9879137 -0 .512892496 | 0 . 13888 -3 .693 | 0 .00022 | 0 .00046 |
| CCDC42 | 5 .33419083 -0 .62823535 | 0 .44403 -1 .4148 | 0 . 15711 | 0 . 19837 |
| CCDC43 | 1969 .040014 -0 . 125553825 | 0 . 10336 -1 .2147 | 0 .22447 | 0 .27326 |
| CCDC47 | 6845 .350746 0 . 162152956 | 0 .08601 1 .88529 | 0 .05939 | 0 .08189 |
| CCDC50 | 24484 .55249 -1 . 184965097 | 0 . 12742 -9 .2999 | 1 .41E-20 | 2 .30E-19 |
| CCDC51 | 1209 .488165 -0 .516114985 | 0 . 14113 -3 .6571 | 0 .00026 | 0 .00053 |
| CCDC53 | 1954 .941504 -0 .847358277 | 0 .09178 -9 .2322 | 2 .65E-20 | 4 .22E-19 |
| CCDC54 | 9 .035391138 -1 .962964719 | 0 .28319 -6 .9317 | 4 . 16E-12 | 2 .54E-1 1 |
| CCDC57 | 1522 .208829 0 .264962775 | 0 . 10479 2 .52844 | 0 .01146 | 0 .01801 |
| CCDC58 | 829 .4917169 -0 .469508453 | 0 . 10728 -4 .3764 | 1 .21E-05 | 3 .07E-05 |
| CCDC59 | 1715 .726946 -0 .754752166 | 0 . 1015 -7 .4361 | 1 .04E-13 | 7 .60E-13 |
| CCDC6 | 7960 .246282 -0 . 142949222 | 0 . 1 1243 -1 .2715 | 0 .20356 | 0 .25052 |
| CCDC60 | 17 .52326189 -1 .84079809 | 0 .37689 -4 .8842 | 1 .04E-06 | 3 .09E-06 |
| CCDC61 | 413 .5777409 0 . 133377942 | 0 .0941 1 .41741 | 0 . 15636 | 0 . 19748 |
| CCDC62 | 88 .65151314 -0 .67390221 | 0 . 15759 -4 .2764 | 1 .90E-05 | 4 .70E-05 |
| CCDC63 | 9 .629968964 -1 .43109989 | 0 .43151 -3 .3165 | 0 .00091 | 0 .00173 |
| CCDC65 | 137 .4827102 0 . 177110826 | 0 . 1 14 1 .55362 | 0 . 12027 | 0 . 15573 |
| CCDC66 | 2061 .961153 -0 .872478845 | 0 . 10926 -7 .9855 | 1 .40E-15 | 1 .27E-14 |

| CCDC67 | 45 .89493495 | -1 .602190435 | 0 .23839 | -6 .721 | 1 .81E-1 1 | 1 .02E-10 |
| --- | --- | --- | --- | --- | --- | --- |
| CCDC68 | 703 .6801657 | -3 .231031169 | 0 .25607 | -12 .618 | 1 .68E-36 | 1 .42E-34 |
| CCDC69 | 1465 .884143 | 1 .392220575 | 0 . 15995 | 8 .70419 | 3 .20E-18 | 3 .99E-17 |
| CCDC7 | 975 . 1 198786 | -1 .489243602 | 0 . 12778 | -1 1 .655 | 2 . 17E-31 | 1 .09E-29 |
| CCDC70 | 2 .982497718 | -1 .59865113 | 0 .65861 | -2 .4273 | 0 .01521 | 0 .0234 |
| CCDC71 | 982 .6416233 | -0 .080640383 | 0 .06544 | -1 .2323 | 0 .21784 | 0 .26595 |
| CCDC71L | 2114 .897081 | 1 .473969066 | 0 . 16032 | 9 . 19384 | 3 .79E-20 | 5 .90E-19 |
| CCDC73 | 52 .05220655 | -1 .891563076 | 0 .24955 | -7 .5798 | 3 .46E-14 | 2 .70E-13 |
| CCDC74A | 195 .6729808 | -0 .004449976 | 0 . 1575 | -0 .0283 | 0 .97746 | 0 .98076 |
| CCDC74B | 53 .44443222 | -0 . 121651121 | 0 .23378 | -0 .5204 | 0 .60281 | 0 .65303 |
| CCDC77 | 590 .8532966 | -0 . 179362847 | 0 .07603 | -2 .359 | 0 .01832 | 0 .02785 |
| CCDC78 | 213 .6904697 | 0 .843987494 | 0 . 17209 | 4 .90427 | 9 .38E-07 | 2 .81E-06 |
| CCDC8 | 769 .5387108 | -0 .34671065 | 0 . 19499 | -1 .7781 | 0 .07539 | 0 . 10194 |
| CCDC80 | 17621 .26029 | 0 .398930039 | 0 .21255 | 1 .87689 | 0 .06053 | 0 .08334 |
| CCDC81 | 78 . 10542379 | -1 .066983363 | 0 . 15058 | -7 .0859 | 1 .38E-12 | 9 .01E-12 |
| CCDC82 | 3069 .991163 | -0 .793691895 | 0 . 1 1995 | -6 .6169 | 3 .67E-1 1 | 2 .00E-10 |
| CCDC83 | 15 .61485397 | -2 .286169095 | 0 .30346 | -7 .5336 | 4 .94E-14 | 3 .78E-13 |
| CCDC84 | 1248 .221167 | -0 .245119498 | 0 . 10831 | -2 .2631 | 0 .02363 | 0 .03523 |
| CCDC85A | 120 .773149 | -0 .049405501 | 0 .20549 | -0 .2404 | 0 .81 | 0 .83855 |
| CCDC85B | 1647 .251621 | 0 .94639545 | 0 . 17421 | 5 .43255 | 5 .56E-08 | 2 .00E-07 |
| CCDC85C | 1098 .787404 | 0 .467108221 | 0 . 1281 | 3 .64629 | 0 .00027 | 0 .00055 |
| CCDC86 | 423 . 1390189 | 0 .428692413 | 0 . 16052 | 2 .67058 | 0 .00757 | 0 .01233 |
| CCDC87 | 24 .93273808 | -0 .650658221 | 0 . 19454 | -3 .3446 | 0 .00082 | 0 .00158 |
| CCDC88A | 9693 .356374 | -0 .34505113 | 0 . 1 1 1 1 | -3 . 1059 | 0 .0019 | 0 .00342 |
| CCDC88B | 663 .2151312 | 1 .560811761 | 0 . 15715 | 9 .93222 | 3 .01E-23 | 6 .51E-22 |
| CCDC88C | 1 140 .524544 | 0 .981290994 | 0 . 19694 | 4 .9827 | 6 .27E-07 | 1 .93E-06 |
| CCDC89 | 1 19 .6665277 | -0 .958559153 | 0 . 18491 | -5 . 1839 | 2 . 17E-07 | 7 . 16E-07 |
| CCDC9 | 374 .5450488 | 0 .356911438 | 0 . 14337 | 2 .48936 | 0 .0128 | 0 .01997 |
| CCDC90B | 2286 .738095 | -0 .759168754 | 0 .07228 | -10 .503 | 8 .38E-26 | 2 .41E-24 |
| CCDC91 | 3124 .316748 | -0 .509740186 | 0 .08584 | -5 .9385 | 2 .88E-09 | 1 .23E-08 |
| CCDC92 | 500 .7730914 | 0 .504572767 | 0 . 12952 | 3 .89578 | 9 .79E-05 | 0 .00022 |
| CCDC93 | 3860 .998506 | -0 .239098996 | 0 .07261 | -3 .2931 | 0 .00099 | 0 .00187 |
| CCDC94 | 920 .6358597 | 0 .006047405 | 0 . 10973 | 0 .05511 | 0 .95605 | 0 .96303 |
| CCDC96 | 45 .60919853 | 0 .257917317 | 0 . 15444 | 1 .67002 | 0 .09492 | 0 . 12564 |
| CCDC97 | 864 .8728328 | 0 .062874718 | 0 . 10214 | 0 .6156 | 0 .53816 | 0 .5915 |
| CCER1 | 6 .535615381 | -4 .606445033 | 0 .73416 | -6 .2744 | 3 .51E-10 | 1 .69E-09 |
| CCER2 | 62 .36619883 | 0 . 134538243 | 0 .37901 | 0 .35497 | 0 .72261 | 0 .76214 |
| CCHCR1 | 1366 .516889 | 0 . 121126793 | 0 . 10644 | 1 . 13794 | 0 .25515 | 0 .30627 |
| CCIN | 18 .54000259 | -0 . 196623588 | 0 .20047 | -0 .9808 | 0 .3267 | 0 .38165 |
| CCK | 9 .862360238 | -0 .387626053 | 0 .41768 | -0 .9281 | 0 .35338 | 0 .40888 |
| CCKAR | 8 .869586117 | -2 .64933052 | 0 .4302 | -6 . 1584 | 7 .35E-10 | 3 .40E-09 |
| CCKBR | 18 .82873331 | -1 .602820816 | 0 .37223 | -4 .306 | 1 .66E-05 | 4 . 15E-05 |
| CCL1 | 5 .620464036 | -0 .794147828 | 0 .47551 | -1 .6701 | 0 .0949 | 0 . 12562 |
| CCL11 | 1 1 .5287009 | 0 .417327318 | 0 .51625 | 0 .80838 | 0 .41887 | 0 .47554 |
| CCL13 | 792 .6172434 | -0 .042120787 | 0 .27876 | -0 . 1511 | 0 .8799 | 0 .89916 |
| CCL17 | 131 .0853815 | 1 .084816072 | 0 .28102 | 3 .86031 | 0 .00011 | 0 .00025 |
| CCL19 | 1479 .412486 | 2 .775215201 | 0 .33948 | 8 . 17489 | 2 .96E-16 | 2 .95E-15 |
| CCL2 | 1040 .56396 | 0 .918260459 | 0 .24055 | 3 .81727 | 0 .00013 | 0 .00029 |
| CCL20 | 79 .63699145 | 0 .261003148 | 0 .32094 | 0 .81325 | 0 .41607 | 0 .47274 |
| CCL21 | 1051 .729492 | 0 .225899162 | 0 .32442 | 0 .69632 | 0 .48623 | 0 .54128 |
| CCL22 | 311 .6188285 | 0 .961929234 | 0 .2424 | 3 .96834 | 7 .24E-05 | 0 .00016 |
| CCL24 | 63 .90798883 | 1 .969811557 | 0 .25817 | 7 .62988 | 2 .35E-14 | 1 .87E-13 |
| CCL25 | 4 .250181625 | -1 .021898053 | 0 .7541 | -1 .3551 | 0 . 17538 | 0 .21934 |
| CCL26 | 34 .88550938 | -0 . 14210569 | 0 .34815 | -0 .4082 | 0 .68314 | 0 .72627 |
| CCL28 | 1798 .455783 | -0 .061228979 | 0 . 1 1078 | -0 .5527 | 0 .58045 | 0 .63172 |
| CCL7 | 16 . 18728627 | 1 .77278365 | 0 .53623 | 3 .30601 | 0 .00095 | 0 .00179 |

| CCL8 | 306 .9982595 | 2 .226582675 | 0 .29788 | 7 .47467 | 7 .74E-14 | 5 .75E-13 |
| --- | --- | --- | --- | --- | --- | --- |
| CCM2 | 2134 .394809 | 0 .981083877 | 0 .07526 | 13 .0351 | 7 .72E-39 | 7 .92E-37 |
| CCM2L | 104 . 1453127 | 1 .07045407 | 0 . 1642 | 6 .51937 | 7 .06E-1 1 | 3 .69E-10 |
| CCNA1 | 30 . 13808981 | 0 .572341209 | 0 .33209 | 1 .72344 | 0 .08481 | 0 . 1 1345 |
| CCNA2 | 1340 .766332 | 1 . 168758624 | 0 . 17199 | 6 .79541 | 1 .08E-1 1 | 6 .26E-1 1 |
| CCNB1 | 1637 .635461 | 1 .500160982 | 0 . 17512 | 8 .56647 | 1 .07E-17 | 1 .25E-16 |
| CCNB1IP1 | 4005 .553823 | -0 .951597836 | 0 . 13515 | -7 .0412 | 1 .91E-12 | 1 .22E-1 1 |
| CCNB2 | 772 .7070123 | 1 .449803911 | 0 . 16807 | 8 .62626 | 6 .34E-18 | 7 .62E-17 |
| CCNB3 | 67 .67455576 | -1 .313936293 | 0 . 19374 | -6 .7821 | 1 . 18E-1 1 | 6 .83E-1 1 |
| CCNC | 6252 .335254 | -0 .426462331 | 0 . 10983 | -3 .883 | 0 .0001 | 0 .00023 |
| CCND1 | 20273 .77284 | 0 .612711723 | 0 . 17455 | 3 .5103 | 0 .00045 | 0 .00089 |
| CCND2 | 8504 .586853 | -0 .090328621 | 0 . 16805 | -0 .5375 | 0 .59091 | 0 .6418 |
| CCND3 | 3270 .896705 | 0 .055305431 | 0 . 1 197 | 0 .46204 | 0 .64405 | 0 .69083 |
| CCNDBP1 | 6223 .837952 | -0 .314982422 | 0 . 1 1093 | -2 .8394 | 0 .00452 | 0 .00766 |
| CCNE1 | 279 .6468715 | 0 .931221345 | 0 . 17677 | 5 .26804 | 1 .38E-07 | 4 .69E-07 |
| CCNE2 | 381 .0323367 | 0 .421756028 | 0 . 13356 | 3 . 15782 | 0 .00159 | 0 .0029 |
| CCNF | 933 .6311652 | 1 .670141637 | 0 . 1409 | 1 1 .8537 | 2 .06E-32 | 1 . 18E-30 |
| CCNG1 | 16761 .20825 | -0 .827726576 | 0 . 16746 | -4 .9428 | 7 .70E-07 | 2 .34E-06 |
| CCNG2 | 4946 .910763 | -0 .088908986 | 0 . 10912 | -0 .8148 | 0 .41519 | 0 .47187 |
| CCNH | 3820 .597436 | -0 .779848784 | 0 .0784 | -9 .9468 | 2 .61E-23 | 5 .66E-22 |
| CCNI | 17870 .2432 | -0 .003767361 | 0 .07527 | -0 .0501 | 0 .96008 | 0 .96633 |
| CCNI2 | 53 . 12368607 | -0 .710681465 | 0 . 1736 | -4 .0937 | 4 .25E-05 | 9 .92E-05 |
| CCNJ | 798 .779814 | -0 .00637703 | 0 .07359 | -0 .0867 | 0 .93095 | 0 .94264 |
| CCNJL | 162 .0861342 | 0 .861401359 | 0 .20818 | 4 . 13781 | 3 .51E-05 | 8 .31E-05 |
| CCNK | 1455 .734939 | 0 .001362119 | 0 .08658 | 0 .01573 | 0 .98745 | 0 .98941 |
| CCNL1 | 10167 .20511 | -0 .356581427 | 0 . 1 1656 | -3 .0592 | 0 .00222 | 0 .00396 |
| CCNL2 | 7115 .886332 | 0 .053836064 | 0 . 1 1239 | 0 .479 | 0 .63194 | 0 .67991 |
| CCNO | 68 .57167063 | 1 . 164399748 | 0 .21027 | 5 .53752 | 3 .07E-08 | 1 . 15E-07 |
| CCNT1 | 5925 .334169 | -0 . 166468681 | 0 .08138 | -2 .0455 | 0 .04081 | 0 .05809 |
| CCNT2 | 6090 .860768 | -0 .402614459 | 0 .08207 | -4 .9059 | 9 .30E-07 | 2 .79E-06 |
| CCNY | 5993 .527914 | 0 .299113228 | 0 .07123 | 4 . 19952 | 2 .67E-05 | 6 .46E-05 |
| CCNYL1 | 1388 . 122936 | 1 .218288566 | 0 .08639 | 14 . 1028 | 3 .65E-45 | 7 .22E-43 |
| CCP110 | 2536 .387813 | -0 .214179237 | 0 .08393 | -2 .5519 | 0 .01071 | 0 .01693 |
| CCPG1 | 1338 .660418 | 0 .280986121 | 0 . 1 1817 | 2 .37781 | 0 .01742 | 0 .02653 |
| CCR1 | 1315 .763407 | 2 .520007554 | 0 .22282 | 1 1 .3095 | 1 . 18E-29 | 5 .02E-28 |
| CCR10 | 40 .08365924 | 0 .509467626 | 0 . 18219 | 2 .79635 | 0 .00517 | 0 .00867 |
| CCR2 | 1 123 .288999 | 1 .096096667 | 0 .21202 | 5 . 16971 | 2 .34E-07 | 7 .68E-07 |
| CCR3 | 26 .65946887 | -1 .082230818 | 0 .29584 | -3 .6581 | 0 .00025 | 0 .00053 |
| CCR4 | 873 .028106 | 1 .830200692 | 0 .26542 | 6 .89561 | 5 .36E-12 | 3 .24E-1 1 |
| CCR5 | 779 .2457234 | 2 .269553152 | 0 .21552 | 10 .5308 | 6 .23E-26 | 1 .82E-24 |
| CCR6 | 33 .66135608 | -0 . 189539277 | 0 .26078 | -0 .7268 | 0 .46735 | 0 .52314 |
| CCR7 | 433 .355851 | 2 .723053157 | 0 .25073 | 10 .8604 | 1 .78E-27 | 6 . 1 1E-26 |
| CCR8 | 1 15 .3855818 | 1 .000343757 | 0 .23543 | 4 .24908 | 2 . 15E-05 | 5 .26E-05 |
| CCR9 | 60 .22639056 | -0 .822548573 | 0 .23243 | -3 .539 | 0 .0004 | 0 .00081 |
| CCRL2 | 222 .3411937 | 2 .321887201 | 0 .25356 | 9 . 15697 | 5 .34E-20 | 8 .21E-19 |
| CCS | 1 176 .036444 | 0 .075864287 | 0 .09865 | 0 .76903 | 0 .44188 | 0 .49774 |
| CCSAP | 3587 . 1 1 1266 | 0 .07731513 | 0 . 12292 | 0 .62897 | 0 .52937 | 0 .58314 |
| CCSER1 | 1454 .282012 | -2 . 109940113 | 0 .20902 | -10 .094 | 5 .86E-24 | 1 .38E-22 |
| CCSER2 | 8359 .254641 | -0 .554571121 | 0 .09101 | -6 .0938 | 1 . 10E-09 | 4 .98E-09 |
| CCT2 | 1 1660 .40729 | -0 .068112948 | 0 . 13752 | -0 .4953 | 0 .6204 | 0 .66947 |
| CCT3 | 21156 .96536 | -0 .041437865 | 0 . 15442 | -0 .2683 | 0 .78843 | 0 .81961 |
| CCT4 | 1 1597 .45473 | -0 . 194788088 | 0 . 12217 | -1 .5944 | 0 . 1 1085 | 0 . 14463 |
| CCT5 | 10910 . 15924 | 0 .273629364 | 0 . 1256 | 2 . 17861 | 0 .02936 | 0 .04297 |
| CCT6A | 17440 .79374 | 0 .392115803 | 0 . 1373 | 2 .85584 | 0 .00429 | 0 .00731 |
| CCT6B | 285 .0556107 | -0 .777388817 | 0 . 1497 | -5 . 193 | 2 .07E-07 | 6 .84E-07 |
| CCT7 | 1 1263 .04297 | -0 .083544354 | 0 . 1 1775 | -0 .7095 | 0 .47801 | 0 .53337 |

| CCT8 | 1 1525 .76522 | -0 .447745213 | 0 . 12332 | -3 .6308 | 0 .00028 | 0 .00058 |
| --- | --- | --- | --- | --- | --- | --- |
| CCT8L2 | 3 . 1 1740266 | -3 .876713494 | 0 .851 | -4 .5555 | 5 .23E-06 | 1 .40E-05 |
| CCZ1 | 787 .7062419 | -0 .285120894 | 0 .09151 | -3 . 1 157 | 0 .00184 | 0 .00331 |
| CCZ1B | 838 .7401601 | -0 . 179181795 | 0 . 1 1388 | -1 .5735 | 0 . 1 1561 | 0 . 15036 |
| CD101 | 275 .2657965 | 0 .391348217 | 0 . 16188 | 2 .41745 | 0 .01563 | 0 .02399 |
| CD109 | 15146 .0847 | 0 .623243164 | 0 . 1534 | 4 .06279 | 4 .85E-05 | 0 .00011 |
| CD14 | 3140 .086026 | 1 .89490566 | 0 . 17166 | 1 1 .0386 | 2 .49E-28 | 9 .34E-27 |
| CD151 | 7226 .910302 | 0 .90117188 | 0 . 1 1495 | 7 .83982 | 4 .51E-15 | 3 .89E-14 |
| CD160 | 198 . 1220996 | -0 .235819578 | 0 . 14821 | -1 .5912 | 0 . 1 1 157 | 0 . 14552 |
| CD163 | 3440 .971641 | 2 .088503844 | 0 . 16994 | 12 .2899 | 1 .03E-34 | 7 .08E-33 |
| CD163L1 | 1216 .090239 | 0 .877481451 | 0 . 16069 | 5 .46078 | 4 .74E-08 | 1 .72E-07 |
| CD164 | 24973 .22273 | -0 . 126362313 | 0 . 10026 | -1 .2603 | 0 .20755 | 0 .25476 |
| CD164L2 | 106 .3150119 | 0 . 138245279 | 0 .24422 | 0 .56608 | 0 .57134 | 0 .62322 |
| CD180 | 283 .4587984 | 2 .301836919 | 0 .23598 | 9 .75432 | 1 .77E-22 | 3 .52E-21 |
| CD19 | 62 .94293136 | 1 .826961084 | 0 .411 16 | 4 .44348 | 8 .85E-06 | 2 .29E-05 |
| CD1A | 1 135 .23943 | -1 .338641041 | 0 .29149 | -4 .5925 | 4 .38E-06 | 1 . 19E-05 |
| CD1B | 301 .8992045 | 0 .641996948 | 0 .33571 | 1 .91234 | 0 .05583 | 0 .07735 |
| CD1C | 787 . 1590411 | -0 .499059102 | 0 .2851 | -1 .7505 | 0 .08003 | 0 . 10757 |
| CD1D | 95 .03577936 | 1 .628906076 | 0 .21598 | 7 .54203 | 4 .63E-14 | 3 .56E-13 |
| CD1E | 677 .3973027 | 0 .043597182 | 0 .32301 | 0 . 13497 | 0 .89263 | 0 .91029 |
| CD2 | 889 . 1724153 | 2 .550072866 | 0 .26754 | 9 .53151 | 1 .55E-21 | 2 .80E-20 |
| CD200 | 820 . 1973152 | -0 .33066072 | 0 . 16976 | -1 .9479 | 0 .05143 | 0 .07179 |
| CD200R1 | 1562 .369054 | -1 . 147304899 | 0 .24445 | -4 .6933 | 2 .69E-06 | 7 .49E-06 |
| CD200R1L | 9 .035969594 | -1 .897692826 | 0 .4291 | -4 .4225 | 9 .76E-06 | 2 .51E-05 |
| CD207 | 2038 .46488 | -1 .524952767 | 0 .30257 | -5 .04 | 4 .66E-07 | 1 .46E-06 |
| CD209 | 994 .4811251 | 1 .955743192 | 0 . 16236 | 12 .0459 | 2 .04E-33 | 1 .27E-31 |
| CD22 | 280 .0139634 | 2 .910297228 | 0 .29407 | 9 .89677 | 4 .30E-23 | 9 . 16E-22 |
| CD226 | 502 .717193 | 1 .029234544 | 0 . 18433 | 5 .58355 | 2 .36E-08 | 8 .95E-08 |
| CD244 | 159 .7845593 | 1 .661577969 | 0 .20599 | 8 .06615 | 7 .25E-16 | 6 .85E-15 |
| CD247 | 460 .6808274 | 2 .429635087 | 0 .24215 | 10 .0338 | 1 .08E-23 | 2 .45E-22 |
| CD248 | 1299 .070258 | 2 .306112901 | 0 . 18476 | 12 .4813 | 9 .44E-36 | 7 .42E-34 |
| CD27 | 126 .2161395 | 2 . 123978348 | 0 .25339 | 8 .38232 | 5 . 19E-17 | 5 .63E-16 |
| CD274 | 627 .3436217 | 1 .07060113 | 0 . 18689 | 5 .7285 | 1 .01E-08 | 4 .03E-08 |
| CD276 | 4175 .986365 | 0 .913634336 | 0 . 12421 | 7 .35536 | 1 .90E-13 | 1 .36E-12 |
| CD28 | 811 .4031368 | 2 .230417896 | 0 .20094 | 1 1 .0998 | 1 .26E-28 | 4 .84E-27 |
| CD2AP | 6577 .390354 | -0 . 170213863 | 0 .09519 | -1 .7881 | 0 .07376 | 0 .0999 |
| CD2BP2 | 4264 .046294 | 0 .235008688 | 0 .09347 | 2 .51418 | 0 .01193 | 0 .01871 |
| CD300A | 523 .7049002 | 1 .897627034 | 0 . 19341 | 9 .81155 | 1 .00E-22 | 2 .04E-21 |
| CD300C | 103 .4446499 | 1 .212699164 | 0 . 19257 | 6 .29747 | 3 .03E-10 | 1 .47E-09 |
| CD300E | 214 .5974909 | 2 .089422226 | 0 .27246 | 7 .6686 | 1 .74E-14 | 1 .40E-13 |
| CD300LB | 78 .75093442 | 1 .360948252 | 0 .20599 | 6 .6068 | 3 .93E-1 1 | 2 . 13E-10 |
| CD300LD | 3 .411 141171 | -2 .368747135 | 0 .5101 | -4 .6437 | 3 .42E-06 | 9 .39E-06 |
| CD300LF | 151 .6806246 | 2 .969292339 | 0 .29017 | 10 .2328 | 1 .41E-24 | 3 .56E-23 |
| CD300LG | 149 .0729941 | 1 .331948494 | 0 .29167 | 4 .56665 | 4 .96E-06 | 1 .33E-05 |
| CD302 | 1006 .524452 | -0 .580216559 | 0 . 1 1217 | -5 . 1726 | 2 .31E-07 | 7 .57E-07 |
| CD320 | 1500 .93906 | 0 .007499185 | 0 . 19403 | 0 .03865 | 0 .96917 | 0 .9738 |
| CD33 | 293 .2479226 | 0 .398751186 | 0 . 17265 | 2 .30959 | 0 .02091 | 0 .03148 |
| CD34 | 1578 .204359 | 0 .882811859 | 0 .20672 | 4 .27066 | 1 .95E-05 | 4 .82E-05 |
| CD36 | 6842 .059751 | 1 .492982301 | 0 .21833 | 6 .83815 | 8 .02E-12 | 4 .72E-1 1 |
| CD37 | 706 .354947 | 1 .849315887 | 0 . 17342 | 10 .6637 | 1 .50E-26 | 4 .64E-25 |
| CD38 | 446 .6268036 | 2 .980579984 | 0 .33321 | 8 .94503 | 3 .72E-19 | 5 . 15E-18 |
| CD3D | 628 .4385 | 2 .058323518 | 0 .27133 | 7 .58597 | 3 .30E-14 | 2 .58E-13 |
| CD3E | 406 .0506237 | 2 .350508167 | 0 .26903 | 8 .73704 | 2 .39E-18 | 3 .03E-17 |
| CD3EAP | 166 .2712115 | 0 .804135498 | 0 . 12171 | 6 .60688 | 3 .92E-1 1 | 2 . 13E-10 |
| CD3G | 551 .6535102 | 2 .490493004 | 0 .25713 | 9 .68568 | 3 .47E-22 | 6 .70E-21 |
| CD4 | 3241 .243316 | 1 .756761633 | 0 . 1603 | 10 .959 | 6 .02E-28 | 2 . 15E-26 |

| CD40 | 826 .3689925 | 0 .216685989 | 0 . 14013 | 1 .54632 | 0 . 12203 | 0 . 15779 |
| --- | --- | --- | --- | --- | --- | --- |
| CD40LG | 121 .0837779 | 0 .521579377 | 0 .26077 | 2 .00018 | 0 .04548 | 0 .06419 |
| CD44 | 99763 .44742 | -0 .860321834 | 0 .08933 | -9 .6303 | 5 .95E-22 | 1 . 12E-20 |
| CD46 | 17070 . 16594 | -0 .070385297 | 0 . 10258 | -0 .6861 | 0 .49264 | 0 .54772 |
| CD47 | 14671 .97507 | 0 . 175924965 | 0 .09595 | 1 .83349 | 0 .06673 | 0 .09106 |
| CD48 | 1228 .981648 | 2 .043855667 | 0 .23748 | 8 .60646 | 7 .54E-18 | 9 .01E-17 |
| CD5 | 365 .5055896 | 1 . 166706379 | 0 .20663 | 5 .64644 | 1 .64E-08 | 6 .36E-08 |
| CD52 | 820 .7844467 | 1 .680969437 | 0 .24331 | 6 .90864 | 4 .89E-12 | 2 .97E-1 1 |
| CD53 | 3435 .575846 | 2 . 1 10500936 | 0 .20801 | 10 . 1459 | 3 .46E-24 | 8 .32E-23 |
| CD55 | 5927 .793532 | -0 .233445562 | 0 . 1 1733 | -1 .9896 | 0 .04663 | 0 .06569 |
| CD58 | 2079 .46518 | 0 .380429324 | 0 . 1 1917 | 3 . 19233 | 0 .00141 | 0 .0026 |
| CD59 | 61402 .49497 | -0 .650074763 | 0 . 13125 | -4 .9528 | 7 .31E-07 | 2 .23E-06 |
| CD5L | 43 .30771415 | 0 .933115735 | 0 .47936 | 1 .9466 | 0 .05158 | 0 .07199 |
| CD6 | 445 .8631931 | 2 .478450098 | 0 .23512 | 10 .541 | 5 .59E-26 | 1 .64E-24 |
| CD63 | 55727 .71253 | 0 .09297233 | 0 .215 | 0 .43244 | 0 .66542 | 0 .71026 |
| CD68 | 1 .685663161 | -0 .700901151 | 0 .48008 | -1 .46 | 0 . 1443 | 0 . 18357 |
| CD69 | 751 .7310992 | 1 .568783145 | 0 .25535 | 6 . 14359 | 8 .07E-10 | 3 .72E-09 |
| CD7 | 99 .25498364 | 3 .780708734 | 0 .30447 | 12 .4172 | 2 . 1 1E-35 | 1 .59E-33 |
| CD70 | 104 .9845023 | 2 .640280915 | 0 .31425 | 8 .40186 | 4 .39E-17 | 4 .80E-16 |
| CD72 | 199 .4352388 | 1 .973575497 | 0 .25511 | 7 .7362 | 1 .02E-14 | 8 .45E-14 |
| CD74 | 40586 .41074 | 2 .076412563 | 0 .22503 | 9 .2271 | 2 .78E-20 | 4 .41E-19 |
| CD79A | 293 . 1313939 | 4 .853625432 | 0 .46533 | 10 .4305 | 1 .80E-25 | 4 .98E-24 |
| CD79B | 423 .5411797 | 0 .419934528 | 0 .26912 | 1 .56039 | 0 . 1 1867 | 0 . 15394 |
| CD80 | 204 .2682377 | 1 .576927965 | 0 .20034 | 7 .87144 | 3 .51E-15 | 3 .07E-14 |
| CD81 | 12595 .72157 | 0 .946312645 | 0 .09462 | 10 .0012 | 1 .50E-23 | 3 .35E-22 |
| CD82 | 3388 .329204 | 0 .517125663 | 0 . 12266 | 4 .21596 | 2 .49E-05 | 6 .04E-05 |
| CD83 | 1956 .340002 | 0 .777019882 | 0 . 15401 | 5 .04527 | 4 .53E-07 | 1 .42E-06 |
| CD84 | 4075 .577046 | 2 . 1 13776616 | 0 .20942 | 10 .0935 | 5 .90E-24 | 1 .39E-22 |
| CD86 | 1 102 .638216 | 1 .407045795 | 0 . 18696 | 7 .52611 | 5 .23E-14 | 3 .99E-13 |
| CD8A | 983 .004258 | 3 .438974715 | 0 .31332 | 10 .976 | 4 .99E-28 | 1 .81E-26 |
| CD8B | 261 .8424086 | 1 .990943173 | 0 .30413 | 6 .54641 | 5 .89E-1 1 | 3 . 12E-10 |
| CD9 | 10159 .42222 | -0 .33167806 | 0 . 1 1906 | -2 .7858 | 0 .00534 | 0 .00894 |
| CD93 | 6743 .697629 | 2 .241674473 | 0 . 14449 | 15 .5144 | 2 .77E-54 | 1 . 18E-51 |
| CD96 | 816 .4090922 | 1 .828756441 | 0 .24238 | 7 .54498 | 4 .52E-14 | 3 .49E-13 |
| CD99 | 6369 .787261 | 0 .39077206 | 0 . 1 1457 | 3 .41078 | 0 .00065 | 0 .00126 |
| CD99L2 | 3209 .067135 | 0 .264257101 | 0 .09441 | 2 .79899 | 0 .00513 | 0 .00861 |
| CDA | 155 .9109533 | 1 .316373882 | 0 .25635 | 5 . 1351 | 2 .82E-07 | 9 . 15E-07 |
| CDADC1 | 577 .8056822 | -0 .759450384 | 0 .0888 | -8 .5527 | 1 .20E-17 | 1 .41E-16 |
| CDAN1 | 1040 .889639 | 0 .076154767 | 0 .08498 | 0 .8961 | 0 .3702 | 0 .42614 |
| CDC123 | 3483 .854797 | -0 .34091781 | 0 . 10511 | -3 .2436 | 0 .00118 | 0 .0022 |
| CDC14A | 1311 .031462 | -0 .276585302 | 0 . 1 1279 | -2 .4522 | 0 .0142 | 0 .02195 |
| CDC14B | 2545 . 143164 | -0 .518397926 | 0 .09786 | -5 .2974 | 1 . 17E-07 | 4 .04E-07 |
| CDC16 | 7142 .214334 | 0 .320456575 | 0 . 1 1982 | 2 .6745 | 0 .00748 | 0 .0122 |
| CDC20 | 698 .5187355 | 1 .534263882 | 0 . 18949 | 8 .09665 | 5 .65E-16 | 5 .44E-15 |
| CDC20B | 19 .43929714 | -3 . 1 1 1055787 | 0 .48393 | -6 .4287 | 1 .29E-10 | 6 .52E-10 |
| CDC23 | 2859 .983662 | -0 .019616775 | 0 .06728 | -0 .2916 | 0 .77063 | 0 .80333 |
| CDC25A | 324 .9032987 | 1 .850430475 | 0 . 1994 | 9 .2799 | 1 .70E-20 | 2 .74E-19 |
| CDC25B | 1592 .303769 | 1 .452002951 | 0 . 14374 | 10 . 1015 | 5 .44E-24 | 1 .29E-22 |
| CDC25C | 183 .789432 | 1 .301912436 | 0 . 17687 | 7 .36075 | 1 .83E-13 | 1 .31E-12 |
| CDC26 | 270 .020076 | -0 .609250719 | 0 . 1329 | -4 .5844 | 4 .55E-06 | 1 .23E-05 |
| CDC27 | 8148 .933368 | 0 . 179823658 | 0 .0984 | 1 .82757 | 0 .06761 | 0 .0922 |
| CDC34 | 1694 .479871 | 0 .277424303 | 0 . 10179 | 2 .72546 | 0 .00642 | 0 .01059 |
| CDC37 | 4887 .317255 | 0 .403858521 | 0 .09431 | 4 .28218 | 1 .85E-05 | 4 .59E-05 |
| CDC37L1 | 1683 . 197274 | -0 .879051252 | 0 .0875 | -10 .047 | 9 .49E-24 | 2 . 18E-22 |
| CDC40 | 2465 .722069 | -0 .254740865 | 0 .08246 | -3 .0893 | 0 .00201 | 0 .0036 |
| CDC42 | 29700 .02109 | 0 .077868782 | 0 .09471 | 0 .82219 | 0 .41097 | 0 .46751 |

| CDC42BPA 8209 .047389 | 0 . 13207166 | 0 . 10708 | 1 .23336 | 0 .21744 | 0 .26551 |
| --- | --- | --- | --- | --- | --- |
| CDC42BPB 5553 .794552 | 0 .410965611 | 0 . 1 1095 | 3 .70405 | 0 .00021 | 0 .00044 |
| CDC42BPG 1422 .609005 | -0 .333411446 | 0 .2407 | -1 .3852 | 0 . 16599 | 0 .20868 |
| CDC42EP1 828 . 190887 | 0 .399120719 | 0 . 15055 | 2 .65103 | 0 .00802 | 0 .013 |
| CDC42EP2 217 .896396 | 0 .706049516 | 0 . 14887 | 4 .74266 | 2 . 1 1E-06 | 5 .96E-06 |
| CDC42EP3 5591 .596919 | 0 .062711544 | 0 . 16211 | 0 .38685 | 0 .69887 | 0 .74064 |
| CDC42EP4 2420 .687883 | 0 .876915728 | 0 . 14492 | 6 .05091 | 1 .44E-09 | 6 .41E-09 |
| CDC42EP5 397 .2586521 | 0 . 14852226 | 0 . 1579 | 0 .94063 | 0 .34689 | 0 .40242 |
| CDC42SE1 7087 .799941 | 0 .490469698 | 0 .07275 | 6 .7418 | 1 .56E-1 1 | 8 .91E-1 1 |
| CDC42SE2 6737 .592189 | 0 .320197439 | 0 . 1 1 148 | 2 .87222 | 0 .00408 | 0 .00697 |
| CDC45 421 .591966 | 1 .809333425 | 0 . 18703 | 9 .67401 | 3 .89E-22 | 7 .47E-21 |
| CDC5L 5788 .321337 | 0 .024488318 | 0 .09364 | 0 .26152 | 0 .79369 | 0 .82402 |
| CDC6 624 .674623 | 1 .807396001 | 0 . 19051 | 9 .48735 | 2 .37E-21 | 4 . 18E-20 |
| CDC7 1309 .889897 | 0 .746555565 | 0 . 16708 | 4 .46815 | 7 .89E-06 | 2 .05E-05 |
| CDC73 5502 .36605 | -0 . 171699976 | 0 .08332 | -2 .0607 | 0 .03933 | 0 .05614 |
| CDCA2 752 .5294691 | 1 .786492168 | 0 . 19353 | 9 .23122 | 2 .68E-20 | 4 .25E-19 |
| CDCA3 301 .5870426 | 0 .806212414 | 0 . 17511 | 4 .60406 | 4 . 14E-06 | 1 . 13E-05 |
| CDCA4 1848 .613077 | 0 .468265652 | 0 . 1 168 | 4 .00909 | 6 . 10E-05 | 0 .00014 |
| CDCA5 412 .7411697 | 1 .348984943 | 0 . 16902 | 7 .98104 | 1 .45E-15 | 1 .32E-14 |
| CDCA7 579 .4452134 | 0 .055904499 | 0 . 17618 | 0 .31732 | 0 .751 | 0 .78634 |
| CDCA7L 2005 .223878 | -0 .655388943 | 0 . 14379 | -4 .558 | 5 . 16E-06 | 1 .38E-05 |
| CDCA8 308 . 1 198416 | 1 .495911222 | 0 . 18984 | 7 .88001 | 3 .27E-15 | 2 .88E-14 |
| CDCP1 2333 .991031 | 0 . 178886826 | 0 . 19804 | 0 .9033 | 0 .36637 | 0 .42218 |
| CDCP2 7 .252554658 | -0 .634543423 | 0 .3408 | -1 .8619 | 0 .06262 | 0 .0859 |
| CDH1 15184 . 1 1 181 | 0 .978421851 | 0 . 17577 | 5 .56652 | 2 .60E-08 | 9 .82E-08 |
| CDH10 29 .29565522 | -1 . 16129033 | 0 .46087 | -2 .5198 | 0 .01174 | 0 .01844 |
| CDH11 5035 .358249 | 1 .266880595 | 0 . 19704 | 6 .42958 | 1 .28E-10 | 6 .49E-10 |
| CDH12 219 .9705801 | -1 .742669756 | 0 .33556 | -5 . 1933 | 2 .07E-07 | 6 .82E-07 |
| CDH13 2160 .316656 | -0 .58582675 | 0 . 15963 | -3 .6699 | 0 .00024 | 0 .0005 |
| CDH15 85 .64776947 | 2 .523736354 | 0 .45353 | 5 .56461 | 2 .63E-08 | 9 .91E-08 |
| CDH16 8 .849868714 | -1 .484342394 | 0 .44364 | -3 .3459 | 0 .00082 | 0 .00157 |
| CDH17 27 . 15957306 | -1 .405046758 | 0 .26269 | -5 .3486 | 8 .86E-08 | 3 . 10E-07 |
| CDH18 24 .71447402 | -2 .227575253 | 0 .44769 | -4 .9757 | 6 .50E-07 | 2 .00E-06 |
| CDH19 18995 .46011 | -0 .888233507 | 0 .28572 | -3 . 1087 | 0 .00188 | 0 .00339 |
| CDH2 482 .4475307 | 0 .849473544 | 0 .36314 | 2 .33927 | 0 .01932 | 0 .02925 |
| CDH20 91 .58543871 | -1 . 121616171 | 0 .22479 | -4 .9896 | 6 .05E-07 | 1 .87E-06 |
| CDH22 85 .78527302 | -1 .667697197 | 0 .36324 | -4 .5912 | 4 .41E-06 | 1 . 19E-05 |
| CDH23 494 .333536 | 0 .479907506 | 0 . 16991 | 2 .82441 | 0 .00474 | 0 .008 |
| CDH24 213 .6760847 | 1 .230435669 | 0 . 14674 | 8 .38518 | 5 .06E-17 | 5 .50E-16 |
| CDH26 185 .3444134 | 0 .207556683 | 0 . 15727 | 1 .31974 | 0 . 18692 | 0 .2323 |
| CDH3 7403 . 183394 | 1 . 199364735 | 0 . 1935 | 6 . 19836 | 5 .71E-10 | 2 .67E-09 |
| CDH4 1 12 .4478351 | 0 .2607296 | 0 .32012 | 0 .81448 | 0 .41537 | 0 .47204 |
| CDH5 1652 .256955 | 1 .703174199 | 0 . 16679 | 10 .2116 | 1 .76E-24 | 4 .36E-23 |
| CDH6 734 .3446583 | 1 .273016577 | 0 . 19581 | 6 .50138 | 7 .96E-1 1 | 4 . 14E-10 |
| CDH7 50 .35875435 | 0 .511722964 | 0 .54914 | 0 .93186 | 0 .35141 | 0 .4069 |
| CDH8 1 12 .368296 | -1 . 123733319 | 0 .29549 | -3 .8029 | 0 .00014 | 0 .00031 |
| CDH9 15 . 12107959 | -5 .835870776 | 0 .72909 | -8 .0044 | 1 .20E-15 | 1 . 10E-14 |
| CDHR1 3769 .574721 | -1 . 19546944 | 0 .35053 | -3 .4105 | 0 .00065 | 0 .00126 |
| CDHR2 42 .51201831 | -0 .612970726 | 0 . 18565 | -3 .3018 | 0 .00096 | 0 .00182 |
| CDHR3 547 . 1 13365 | 0 .250306305 | 0 . 17539 | 1 .42711 | 0 . 15355 | 0 . 19427 |
| CDHR4 19 .50924573 | -1 . 196909073 | 0 .36534 | -3 .2761 | 0 .00105 | 0 .00198 |
| CDHR5 76 .52994891 | 0 .712189107 | 0 .22465 | 3 . 1702 | 0 .00152 | 0 .00279 |
| CDIP1 951 . 1327567 | 0 .28452308 | 0 .09542 | 2 .98174 | 0 .00287 | 0 .00502 |
| CDIPT 3389 .587196 | 0 . 150249504 | 0 .09169 | 1 .63875 | 0 . 10126 | 0 . 13322 |
| CDK1 1752 .658618 | 1 . 171983109 | 0 . 17115 | 6 .84781 | 7 .50E-12 | 4 .43E-1 1 |
| CDK10 1656 .012399 | 0 .21177705 | 0 . 10032 | 2 . 1 1 105 | 0 .03477 | 0 .05013 |

| CDK11A CDK11B CDK12 CDK13 CDK14 CDK15 CDK16 CDK17 CDK18 CDK19 CDK2 CDK20 CDK2AP1 CDK2AP2 CDK3 CDK4 CDK5 CDK5R1 CDK5R2 CDK5RAP1 CDK5RAP2 CDK5RAP3 CDK6 CDK7 CDK8 CDK9 CDKAL1 CDKL1 CDKL2 CDKL3 CDKL4 CDKL5 CDKN1A CDKN1B CDKN1C CDKN2A | 83 .78676203 517 .8139008 5083 .449747 7056 .559274 2090 .850268 426 .9497876 2053 .607193 5987 .590999 319 .0789931 3476 .614683 6118 .905395 443 .0143558 1832 .599221 460 .4527258 12 .6756584 3155 .421214 841 .5144085 1469 .601465 3 .397234772 1523 .234197 5110 .655623 2611 .715209 19612 .97307 2140 . 133995 1 167 .613151 2536 .316998 3258 .630215 779 .4107331 232 .712093 70 .50995944 59 .34638946 648 .0219884 7146 .900224 5120 . 17724 1505 .888148 541 .439967 |
| --- | --- |

CDKN2A-AS 4 .001763568 CDKN2AIP 2032 .82738 CDKN2AIPNL1395 .357231

| CDKN2B CDKN2C CDKN2D CDKN3 CDNF CDO1 CDON CDPF1 CDR1 CDR2 CDR2L CDRT1 CDRT15 CDRT15L2 CDRT4 CDS1 CDS2 | 2364 .936906 1238 .529612 632 . 1240834 381 .3984053 166 .8179846 326 .0653229 4533 .341612 537 .8897565 3478 .557327 1021 .822167 316 .7732357 37 .50090063 12 .81201765 4 .512724457 59 .51050008 1597 .711684 9434 .861549 |
| --- | --- |

-0 .043446232

0 .31332057

0 .276203959

0 .038293411

-0 .720971172

0 .622357336

0 .93906973

0 .282237241

0 .62120326

-0 .626438753

1 .712426299

-0 .513506943

1 . 16418769

1 .296145274

-0 .034983345

0 .030417695

1 .019922638

0 .972138798

-1 .710859311

0 .073971955

-0 .342543827

-0 .090397555

-0 .312329017

-0 .447927372

0 .003448176

0 .607605948

-0 .230540819

-0 .314502778

-1 .914152389

-0 .504509628

-1 . 169426446

-0 .624238418

1 .092288386

0 .312088145

-0 .39924103

0 .554986373

-1 . 166180785

-0 .51950858

0 .079631066

0 .526366494

0 .554541558

0 .863538027

0 .948863556

-1 .344886552

-1 .210653233

-0 .76765848

-0 . 107362283

-1 .337405703

0 .623968983

1 .484414849

-0 .754532206

-0 .374928094

-3 .072585721

-0 .732942622

-0 .304310816

0 .360957698

0 . 12173 0 . 1 1245 0 .06776 0 .06939 0 . 14503 0 .20217 0 . 10555 0 .09535 0 .22119 0 .08938 0 .21038 0 . 1 1897 0 . 13013 0 . 1226 0 . 18129 0 . 14016 0 . 18155 0 .20357 0 .66498 0 .06836 0 .09431 0 .09045 0 . 17291 0 . 10668 0 .0675 0 .06712 0 .07718 0 . 1 1427 0 .27382 0 . 14508 0 . 17559 0 .08896 0 . 1522 0 .0962 0 . 18828 0 .31301 0 .46969 0 .08329 0 . 13045 0 . 16346 0 . 13616 0 . 12333 0 .2184 0 . 16917 0 .32909 0 . 17339 0 . 12942 0 .33144 0 .09845 0 . 12466 0 .25259 0 .32525 0 .63366 0 .22791 0 .21771 0 .09322

-0 .3569 2 .78621 4 .07651 0 .55182 -4 .9712 3 .07835 8 .89723 2 .95986 2 .80849 -7 .009 8 . 1396 -4 .3164 8 .94635 10 .5722 -0 . 193 0 .21702 5 .61792 4 .77554 -2 .5728 1 .08214 -3 .6319 -0 .9994 -1 .8063 -4 . 1989 0 .05108 9 .05288 -2 .9871 -2 .7523 -6 .9906 -3 .4774 -6 .6602 -7 .0174 7 . 17653 3 .24402 -2 . 1205 1 .77305 -2 .4829 -6 .2372 0 .61041 3 .22023 4 .07267 7 .00168 4 .34457 -7 .95 -3 .6788 -4 .4275 -0 .8296 -4 .0352 6 .33777 1 1 .9073 -2 .9872 -1 . 1527 -4 .8489 -3 .216 -1 .3978 3 .87226

0 .72117 0 .00533 4 .57E-05 0 .58107 6 .65E-07 0 .00208 5 .73E-19 0 .00308 0 .00498 2 .40E-12 3 .97E-16 1 .59E-05 3 .67E-19 4 .01E-26 0 .84699 0 .82819 1 .93E-08 1 .79E-06 0 .01009 0 .27919 0 .00028 0 .31759 0 .07088 2 .68E-05 0 .95926 1 .39E-19 0 .00282 0 .00592 2 .74E-12 0 .00051 2 .74E-1 1 2 .26E-12 7 . 15E-13 0 .00118 0 .03396 0 .07622 0 .01303 4 .46E-10 0 .54159 0 .00128 4 .65E-05 2 .53E-12 1 .40E-05 1 .87E-15 0 .00023 9 .53E-06 0 .40677 5 .46E-05 2 .33E-10 1 .08E-32 0 .00282 0 .24902 1 .24E-06 0 .0013 0 . 16217 0 .00011

0 .76081 0 .00893 0 .00011 0 .63226 2 .04E-06 0 .00373 7 .80E-18 0 .00536 0 .00838 1 .52E-1 1 3 .91E-15 3 .97E-05 5 . 1 1E-18 1 . 19E-24 0 .87101 0 .85489 7 .41E-08 5 . 13E-06 0 .01603 0 .33195 0 .00058 0 .37212 0 .0962 6 .47E-05 0 .9658 2 .04E-18 0 .00494 0 .00984 1 .72E-1 1 0 .001 1 .52E-10 1 .44E-1 1 4 .80E-12 0 .0022 0 .04906 0 . 10297 0 .02029 2 . 1 1E-09 0 .59469 0 .00238 0 .00011 1 .60E-1 1 3 .52E-05 1 .69E-14 0 .00049 2 .46E-05 0 .46332 0 .00013 1 . 15E-09 6 .32E-31 0 .00494 0 .29987 3 .65E-06 0 .00241 0 .20423 0 .00024

| CDSN 186 .5815728 | -0 .234576195 | 0 .28311 | -0 .8286 | 0 .40734 | 0 .46391 |
| --- | --- | --- | --- | --- | --- |
| CDT1 353 . 1 163604 | 0 .904636052 | 0 . 10491 | 8 .62296 | 6 .52E-18 | 7 .84E-17 |
| CDV3 12219 .93441 | 0 . 146496408 | 0 .07143 | 2 .05095 | 0 .04027 | 0 .05738 |
| CDX1 35 .63770075 | 1 .654660051 | 0 .26775 | 6 . 17988 | 6 .41E-10 | 2 .99E-09 |
| CDX2 6 .315191528 | -3 .202212207 | 0 .70044 | -4 .5717 | 4 .84E-06 | 1 .30E-05 |
| CDX4 27 .83721409 | -3 .054285246 | 0 .4096 | -7 .4568 | 8 .87E-14 | 6 .55E-13 |
| CDYL 4470 .585761 | -0 .275962132 | 0 . 10024 | -2 .7529 | 0 .00591 | 0 .00982 |
| CDYL2 982 .3884686 | 1 .449422825 | 0 . 14131 | 10 .2569 | 1 . 10E-24 | 2 .82E-23 |
| CEACAM1 1250 .295116 | -0 .66276411 1 | 0 .23848 | -2 .7792 | 0 .00545 | 0 .00912 |
| CEACAM16 3 .863745528 | -0 .364409836 | 0 .433 | -0 .8416 | 0 .40001 | 0 .45664 |
| CEACAM18 2 .7350748 | -3 .697480693 | 0 .87298 | -4 .2355 | 2 .28E-05 | 5 .57E-05 |
| CEACAM19 160 .4706592 | -0 .341765194 | 0 .20664 | -1 .6539 | 0 .09814 | 0 . 12947 |
| CEACAM21 69 .0192487 | 2 .090703837 | 0 .23681 | 8 .82845 | 1 .06E-18 | 1 .41E-17 |
| CEACAM3 20 .37396065 | 0 .638554011 | 0 .39742 | 1 .60675 | 0 . 10811 | 0 . 14143 |
| CEACAM4 26 .24261919 | 1 .684565065 | 0 .2861 | 5 .88804 | 3 .91E-09 | 1 .64E-08 |
| CEACAM5 82 .68333178 | 2 .086691322 | 0 .32826 | 6 .35686 | 2 .06E-10 | 1 .02E-09 |
| CEACAM6 316 . 1928153 | 3 .245685683 | 0 .34942 | 9 .28889 | 1 .56E-20 | 2 .54E-19 |
| CEACAM7 45 .64801066 | 0 .303328776 | 0 .40939 | 0 .74093 | 0 .45874 | 0 .51456 |
| CEACAM8 10 .88679027 | -1 .528436926 | 0 .38694 | -3 .9501 | 7 .81E-05 | 0 .00017 |
| CEBPA 80 .980236 | 0 .960155968 | 0 .33834 | 2 .83786 | 0 .00454 | 0 .00769 |
| CEBPB 1880 . 168959 | 1 .683097824 | 0 . 15657 | 10 .7498 | 5 .94E-27 | 1 .93E-25 |
| CEBPD 1637 .634268 | 0 .83647312 | 0 . 14429 | 5 .79715 | 6 .75E-09 | 2 .76E-08 |
| CEBPE 8 .340451455 | 0 .406387089 | 0 .34352 | 1 . 183 | 0 .23681 | 0 .28672 |
| CEBPG 3543 .882395 | -0 .373089707 | 0 .0867 | -4 .3034 | 1 .68E-05 | 4 . 19E-05 |
| CEBPZ 4046 .438296 | -0 .31520968 | 0 . 1 1 197 | -2 .8152 | 0 .00487 | 0 .00822 |
| CEBPZOS 1841 .602867 | -0 .213363091 | 0 . 1013 | -2 . 1062 | 0 .03519 | 0 .05068 |
| CECR1 4183 .239151 | 1 .682370094 | 0 .20944 | 8 .03276 | 9 .53E-16 | 8 .84E-15 |
| CECR2 376 .775801 | -0 .73104534 | 0 .28019 | -2 .6091 | 0 .00908 | 0 .01456 |
| CECR5 2098 .455161 | 0 .830464656 | 0 . 10748 | 7 .72643 | 1 . 1 1E-14 | 9 .08E-14 |
| CECR6 72 .75836232 | 0 .857630644 | 0 . 14821 | 5 .78646 | 7 . 19E-09 | 2 .93E-08 |
| CEL 16 .20626714 | 0 . 152799862 | 0 .27428 | 0 .55709 | 0 .57747 | 0 .62902 |
| CELA1 3 .438142393 | -2 .039616476 | 0 .53898 | -3 .7842 | 0 .00015 | 0 .00033 |
| CELA2A 4 .418437163 | -1 .210862941 | 0 .45854 | -2 .6407 | 0 .00827 | 0 .01337 |
| CELA2B 13 .7750878 | -0 .378231343 | 0 .21477 | -1 .7611 | 0 .07822 | 0 . 10535 |
| CELA3A 1 .78639962 | -3 . 10118307 | 0 .88121 | -3 .5192 | 0 .00043 | 0 .00086 |
| CELA3B 2 .504129273 | -3 .407504255 | 0 .78988 | -4 .314 | 1 .60E-05 | 4 .00E-05 |
| CELF1 8782 .214153 | 0 .271454945 | 0 .07645 | 3 .55057 | 0 .00038 | 0 .00077 |
| CELF2 12033 .60154 | 0 .023934135 | 0 . 12242 | 0 . 19551 | 0 .84499 | 0 .86926 |
| CELF3 14 .2098983 | -2 .086983502 | 0 .25571 | -8 . 1614 | 3 .31E-16 | 3 .28E-15 |
| CELF4 63 .05944106 | -1 .251785377 | 0 .20687 | -6 .051 | 1 .44E-09 | 6 .41E-09 |
| CELF5 23 .08996112 | -1 .931913518 | 0 .34603 | -5 .583 | 2 .36E-08 | 8 .97E-08 |
| CELF6 2 .550143004 | -0 .294220143 | 0 .48938 | -0 .6012 | 0 .5477 | 0 .60051 |
| CELSR1 3418 .980292 | 0 . 122064737 | 0 . 15681 | 0 .77845 | 0 .43631 | 0 .49239 |
| CELSR2 6913 .672156 | -0 .083723771 | 0 . 16929 | -0 .4945 | 0 .62092 | 0 .66999 |
| CELSR3 226 .0321684 | 1 .023597724 | 0 . 14488 | 7 .0653 | 1 .60E-12 | 1 .04E-1 1 |
| CEMIP 2249 .548686 | 1 .659282513 | 0 .22561 | 7 .35462 | 1 .91E-13 | 1 .37E-12 |
| CEND1 31 .07041595 | 0 .357462928 | 0 . 18808 | 1 .90059 | 0 .05736 | 0 .07928 |
| CENPA 273 .5958867 | 1 .306333863 | 0 . 18753 | 6 .966 | 3 .26E-12 | 2 .03E-1 1 |
| CENPB 5089 . 1 10156 | 0 .908508182 | 0 .09883 | 9 . 19301 | 3 .82E-20 | 5 .94E-19 |
| CENPBD1 195 .2299836 | 0 .660192626 | 0 . 1 1006 | 5 .99858 | 1 .99E-09 | 8 .69E-09 |
| CENPC 4053 .256715 | -0 .577657893 | 0 . 10831 | -5 .3335 | 9 .63E-08 | 3 .36E-07 |
| CENPE 1273 .049742 | 0 .942748448 | 0 . 17269 | 5 .45922 | 4 .78E-08 | 1 .73E-07 |
| CENPF 5892 .706909 | 2 .059040561 | 0 .2014 | 10 .2239 | 1 .55E-24 | 3 .88E-23 |
| CENPH 788 .5503224 | 0 .321625337 | 0 . 12033 | 2 .67285 | 0 .00752 | 0 .01225 |
| CENPI 589 .8692577 | 0 .476985961 | 0 . 14294 | 3 .33688 | 0 .00085 | 0 .00162 |
| CENPJ 1094 .31041 | -0 .202269801 | 0 . 10707 | -1 .8891 | 0 .05887 | 0 .08122 |

| CENPK | 563 .6633876 | 0 .977749288 | 0 . 1623 | 6 .02426 | 1 .70E-09 | 7 .48E-09 |
| --- | --- | --- | --- | --- | --- | --- |
| CENPL | 848 . 17045 | 0 .862141471 | 0 . 1267 | 6 .80463 | 1 .01E-1 1 | 5 .89E-1 1 |
| CENPM | 305 .5609698 | 0 . 1 14690352 | 0 . 17193 | 0 .66706 | 0 .50473 | 0 .55948 |
| CENPN | 923 .6876954 | 1 . 1 18262177 | 0 . 16995 | 6 .58006 | 4 .70E-1 1 | 2 .53E-10 |
| CENPO | 466 .5390433 | 0 .422795018 | 0 . 10223 | 4 . 13565 | 3 .54E-05 | 8 .38E-05 |
| CENPP | 1313 .804091 | -1 .08676747 | 0 . 12143 | -8 .9499 | 3 .56E-19 | 4 .97E-18 |
| CENPQ | 758 .7040261 | -0 .05434046 | 0 . 13939 | -0 .3899 | 0 .69665 | 0 .73866 |
| CENPT | 1692 . 16289 | -0 .295742931 | 0 . 1 1 16 | -2 .65 | 0 .00805 | 0 .01304 |
| CENPU | 1460 .276912 | 0 . 175803822 | 0 . 17126 | 1 .02652 | 0 .30465 | 0 .35866 |
| CENPV | 272 .4557345 | -0 .386690662 | 0 . 16419 | -2 .3552 | 0 .01851 | 0 .0281 |
| CENPW | 272 . 1894758 | 0 .345390699 | 0 .2055 | 1 .68074 | 0 .09281 | 0 . 12318 |
| CEP104 | 1661 .737184 | 0 .479766882 | 0 .09076 | 5 .28619 | 1 .25E-07 | 4 .28E-07 |
| CEP112 | 814 .9892404 | -0 .858156841 | 0 . 16299 | -5 .2652 | 1 .40E-07 | 4 .75E-07 |
| CEP120 | 3245 .812874 | -0 .203881862 | 0 .09372 | -2 . 1753 | 0 .02961 | 0 .0433 |
| CEP126 | 1067 .792296 | -0 .92245695 | 0 .20271 | -4 .5506 | 5 .35E-06 | 1 .43E-05 |
| CEP128 | 892 .6796494 | -0 .446435983 | 0 . 12262 | -3 .6409 | 0 .00027 | 0 .00056 |
| CEP131 | 499 .5661895 | 0 .711293467 | 0 . 13393 | 5 .31092 | 1 .09E-07 | 3 .77E-07 |
| CEP135 | 1310 .256535 | 0 .364187789 | 0 . 12031 | 3 .02701 | 0 .00247 | 0 .00437 |
| CEP152 | 1084 .940647 | -0 .070517887 | 0 . 10419 | -0 .6768 | 0 .4985 | 0 .5534 |
| CEP162 | 1902 .931188 | -0 .770658551 | 0 . 1 1655 | -6 .6121 | 3 .79E-1 1 | 2 .07E-10 |
| CEP164 | 1972 . 157373 | 0 .047319184 | 0 .09268 | 0 .51054 | 0 .60967 | 0 .6593 |
| CEP170 | 9087 .56202 | -0 .479759435 | 0 . 13183 | -3 .6391 | 0 .00027 | 0 .00056 |
| CEP170B | 2295 .856583 | 0 . 151334262 | 0 . 13782 | 1 .09805 | 0 .27218 | 0 .32436 |
| CEP19 | 257 .7347833 | 0 .479196217 | 0 . 15764 | 3 .0399 | 0 .00237 | 0 .0042 |
| CEP192 | 7402 .315157 | -0 .437565524 | 0 .08983 | -4 .8713 | 1 . 1 1E-06 | 3 .29E-06 |
| CEP250 | 1903 .327097 | 0 .408808258 | 0 . 1 1 1 19 | 3 .67665 | 0 .00024 | 0 .00049 |
| CEP290 | 2603 .82391 | -0 .763137828 | 0 . 12762 | -5 .9796 | 2 .24E-09 | 9 .72E-09 |
| CEP295 | 2789 .550293 | -0 .449389134 | 0 .08503 | -5 .2853 | 1 .25E-07 | 4 .29E-07 |
| CEP295NL | 224 .9019884 | -0 .077100975 | 0 .22389 | -0 .3444 | 0 .73057 | 0 .76884 |
| CEP350 | 16916 .29116 | 0 .044403494 | 0 .07883 | 0 .56327 | 0 .57325 | 0 .62501 |
| CEP41 | 968 .4425404 | 0 .241509365 | 0 . 13194 | 1 .83047 | 0 .06718 | 0 .09165 |
| CEP44 | 2764 .723959 | -0 .544589699 | 0 . 1 1488 | -4 .7404 | 2 . 13E-06 | 6 .03E-06 |
| CEP55 | 788 .6595384 | 1 .811718184 | 0 . 17046 | 10 .6281 | 2 .20E-26 | 6 .75E-25 |
| CEP57 | 5863 .813288 | -0 .891330375 | 0 .09746 | -9 . 1458 | 5 .92E-20 | 9 .05E-19 |
| CEP57L1 | 1263 .29062 | -0 .445854783 | 0 .09981 | -4 .467 | 7 .93E-06 | 2 .06E-05 |
| CEP63 | 1461 .208677 | -0 .406331441 | 0 .07768 | -5 .2305 | 1 .69E-07 | 5 .65E-07 |
| CEP68 | 4314 .582013 | -0 .38293932 | 0 .09159 | -4 . 1809 | 2 .90E-05 | 6 .97E-05 |
| CEP70 | 1315 .90043 | -1 . 16520341 | 0 . 10481 | -1 1 . 1 17 | 1 .04E-28 | 4 .05E-27 |
| CEP72 | 547 .2173543 | 0 .54869898 | 0 . 1502 | 3 .65303 | 0 .00026 | 0 .00054 |
| CEP76 | 881 .3723991 | -0 .275030601 | 0 .09705 | -2 .834 | 0 .0046 | 0 .00778 |
| CEP78 | 2628 .523454 | 0 .009320035 | 0 .09422 | 0 .09891 | 0 .92121 | 0 .93446 |
| CEP83 | 1750 . 152287 | -0 .528708657 | 0 . 1 1258 | -4 .6964 | 2 .65E-06 | 7 .39E-06 |
| CEP85 | 1339 .726061 | 0 .405459387 | 0 . 10304 | 3 .93486 | 8 .32E-05 | 0 .00019 |
| CEP85L | 4157 .207494 | -1 .033037954 | 0 . 10921 | -9 .4596 | 3 .09E-21 | 5 .38E-20 |
| CEP89 | 960 .8688787 | -0 . 135718136 | 0 .07301 | -1 .8589 | 0 .06304 | 0 .08644 |
| CEP95 | 2319 .684855 | -0 .398975081 | 0 . 132 | -3 .0225 | 0 .00251 | 0 .00443 |
| CEP97 | 3222 .502866 | -0 . 120028418 | 0 . 10748 | -1 . 1 168 | 0 .26408 | 0 .31573 |
| CEPT1 | 3780 .585733 | -0 .475866961 | 0 .08451 | -5 .6309 | 1 .79E-08 | 6 .91E-08 |
| CER1 | 6 .857301927 | -1 .600437404 | 0 .44442 | -3 .6011 | 0 .00032 | 0 .00065 |
| CERCAM | 1633 .684603 | 0 .674896639 | 0 . 13114 | 5 . 14622 | 2 .66E-07 | 8 .65E-07 |
| CERK | 4855 .288709 | 0 .764279737 | 0 .095 | 8 .04505 | 8 .62E-16 | 8 .04E-15 |
| CERKL | 404 .9823596 | 0 .207701105 | 0 . 18577 | 1 . 1 1805 | 0 .26355 | 0 .31519 |
| CERS1 | 1 13 .6674017 | -0 . 122988673 | 0 .26446 | -0 .4651 | 0 .64189 | 0 .68881 |
| CERS2 | 1812 .034057 | 0 .503891316 | 0 . 12276 | 4 . 10457 | 4 .05E-05 | 9 .50E-05 |
| CERS3 | 3506 . 164378 | -0 .769825248 | 0 .27202 | -2 .8301 | 0 .00465 | 0 .00787 |
| CERS4 | 3241 .916463 | -0 . 137763752 | 0 . 17427 | -0 .7905 | 0 .42922 | 0 .48577 |

| CERS5 | 2660 .822937 0 .062614603 | 0 .07203 0 .86933 | 0 .38467 | 0 .44104 |
| --- | --- | --- | --- | --- |
| CERS6 | 4281 .620144 -0 .694990886 | 0 . 13357 -5 .2033 | 1 .96E-07 | 6 .49E-07 |
| CES1 | 935 .7314934 1 .207027644 | 0 .32728 3 .68806 | 0 .00023 | 0 .00047 |
| CES2 | 1793 .387523 0 . 156435311 | 0 . 10318 1 .51613 | 0 . 12949 | 0 . 16654 |
| CES3 | 83 .71907992 0 .383269548 | 0 .23181 1 .65338 | 0 .09825 | 0 . 1296 |
| CES4A | 1062 .086143 -1 .716476326 | 0 .2351 -7 .3011 | 2 .85E-13 | 2 .00E-12 |
| CES5A | 27 . 10883909 -1 .61358775 | 0 .38523 -4 . 1886 | 2 .81E-05 | 6 .75E-05 |
| CETN1 | 1 .617368845 -4 . 193605531 | 1 .05078 -3 .991 | 6 .58E-05 | 0 .00015 |
| CETN2 | 2210 .43756 -0 .200723226 | 0 . 1 1906 -1 .6859 | 0 .09181 | 0 . 12202 |
| CETN3 | 1 180 .702245 -1 .085283967 | 0 . 15468 -7 .0165 | 2 .27E-12 | 1 .45E-1 1 |
| CETP | 69 . 17208201 1 .206885795 | 0 . 17299 6 .97661 | 3 .02E-12 | 1 .89E-1 1 |
| CFAP100 | 40 .63444802 -0 .820240707 | 0 .22645 -3 .6221 | 0 .00029 | 0 .0006 |
| CFAP126 | 388 . 1 136682 -0 . 1732402 | 0 . 10632 -1 .6295 | 0 . 10321 | 0 . 13555 |
| CFAP157 | 83 .03702863 0 .346042128 | 0 . 13919 2 .48613 | 0 .01291 | 0 .02013 |
| CFAP161 | 60 .3077386 -0 .509816359 | 0 . 17137 -2 .975 | 0 .00293 | 0 .00512 |
| CFAP20 | 1921 .428283 -0 .318965107 | 0 . 1 1664 -2 .7346 | 0 .00625 | 0 .01033 |
| CFAP206 | 6 .94760173 -1 .716083909 | 0 .3598 -4 .7695 | 1 .85E-06 | 5 .27E-06 |
| CFAP221 | 43 .02641829 -1 .468875224 | 0 .33839 -4 .3408 | 1 .42E-05 | 3 .58E-05 |
| CFAP36 | 1944 .587797 -0 .72054617 | 0 . 10902 -6 .6096 | 3 .85E-1 1 | 2 . 10E-10 |
| CFAP43 | 160 .560399 -1 .708632101 | 0 . 19683 -8 .6808 | 3 .93E-18 | 4 .87E-17 |
| CFAP44 | 2315 .432741 -1 .336698082 | 0 . 16242 -8 .2298 | 1 .88E-16 | 1 .90E-15 |
| CFAP45 | 67 .02338463 -0 .208153486 | 0 .22596 -0 .9212 | 0 .35694 | 0 .41258 |
| CFAP46 | 50 .69821567 -1 .560336331 | 0 .28442 -5 .4861 | 4 . 1 1E-08 | 1 .50E-07 |
| CFAP47 | 17 .50139585 -1 .779346635 | 0 .5193 -3 .4265 | 0 .00061 | 0 .00119 |
| CFAP52 | 20 .76560947 -2 .312169356 | 0 .29905 -7 .7318 | 1 .06E-14 | 8 .72E-14 |
| CFAP53 | 57 .53433108 -0 .7330039 | 0 . 1699 -4 .3144 | 1 .60E-05 | 4 .00E-05 |
| CFAP54 | 183 .4675543 -0 .690378425 | 0 .25773 -2 .6787 | 0 .00739 | 0 .01206 |
| CFAP57 | 55 .07716494 -1 .594196972 | 0 .27615 -5 .7729 | 7 .79E-09 | 3 . 16E-08 |
| CFAP58 | 41 .29168047 -0 .678224355 | 0 .22969 -2 .9528 | 0 .00315 | 0 .00548 |
| CFAP61 | 156 . 1816575 0 .942114536 | 0 .36837 2 .55754 | 0 .01054 | 0 .01669 |
| CFAP65 | 31 .28940384 -2 . 12306544 | 0 .40463 -5 .2469 | 1 .55E-07 | 5 .20E-07 |
| CFAP69 | 910 .4250977 -0 .681770465 | 0 . 16746 -4 .0712 | 4 .68E-05 | 0 .00011 |
| CFAP70 | 663 .3400188 -1 .882331962 | 0 .21174 -8 .8899 | 6 . 12E-19 | 8 .30E-18 |
| CFAP73 | 41 .90810889 -0 .773170542 | 0 . 19859 -3 .8933 | 9 .89E-05 | 0 .00022 |
| CFAP74 | 19 .75296536 -2 .408364535 | 0 .34665 -6 .9475 | 3 .72E-12 | 2 .29E-1 1 |
| CFAP77 | 9 .907099777 -0 .333454884 | 0 .37766 -0 .8829 | 0 .37727 | 0 .43341 |
| CFAP97 | 8782 .389879 -0 . 170972956 | 0 . 10816 -1 .5807 | 0 . 1 1394 | 0 . 14837 |
| CFB | 152 .3287639 0 .212827216 | 0 . 13927 1 .52815 | 0 . 12648 | 0 . 16292 |
| CFC1 | 0 .468507167 -2 .470383355 | 1 .59053 -1 .5532 | 0 . 12038 | 0 . 15584 |
| CFD | 9773 .271438 0 .748366035 | 0 .2936 2 .54894 | 0 .01081 | 0 .01706 |
| CFDP1 | 1357 .699501 -0 .69926376 | 0 . 1 123 -6 .2266 | 4 .77E-10 | 2 .25E-09 |
| CFH | 62368 .60891 -3 . 167310551 | 0 .23256 -13 .619 | 3 .08E-42 | 4 .41E-40 |
| CFHR1 | 25 .78233903 -1 .862566263 | 0 .30497 -6 . 1074 | 1 .01E-09 | 4 .60E-09 |
| CFHR2 | 3 .683976255 -5 .690557011 | 1 . 1 1382 -5 . 109 | 3 .24E-07 | 1 .04E-06 |
| CFHR3 | 34 .92934541 -2 .007362289 | 0 .38125 -5 .2652 | 1 .40E-07 | 4 .75E-07 |
| CFHR4 | 32 .80742104 0 .628901143 | 0 .63035 0 .99769 | 0 .31843 | 0 .37294 |
| CFHR5 | 8 .601902421 -6 .291978868 | 0 .75898 -8 .2901 | 1 . 13E-16 | 1 . 19E-15 |
| CFI | 560 . 1764074 1 . 168635916 | 0 . 18406 6 .34905 | 2 . 17E-10 | 1 .07E-09 |
| CFL1 | 14019 .43717 0 .750149748 | 0 . 14479 5 . 18112 | 2 .21E-07 | 7 .26E-07 |
| CFL2 | 2962 .09351 -0 .640702869 | 0 . 128 -5 .0057 | 5 .57E-07 | 1 .72E-06 |
| CFLAR | 9673 .851052 0 .334882517 | 0 . 10013 3 .34446 | 0 .00082 | 0 .00158 |
| CFP | 325 .710963 0 .816149791 | 0 . 19447 4 . 19683 | 2 .71E-05 | 6 .53E-05 |
| CFTR | 361 .9047964 0 .846570048 | 0 .29525 2 .86726 | 0 .00414 | 0 .00707 |
| CGA | 5 .797151804 -0 .858274855 | 0 .6997 -1 .2266 | 0 .21996 | 0 .26816 |
| CGB2 | 0 .651600604 -1 .601289803 | 1 .23021 -1 .3016 | 0 . 19304 | 0 .23916 |
| CGB3 | 0 .43947128 -1 .561381627 | 1 .40569 -1 . 1 108 | 0 .26667 | 0 .31841 |

| CGB5 | 0 .801779354 | -2 .440386706 | 0 .94421 | -2 .5846 | 0 .00975 | 0 .01554 |
| --- | --- | --- | --- | --- | --- | --- |
| CGB7 | 13 .42746683 | -0 .569633674 | 0 .26977 | -2 . 1 1 15 | 0 .03473 | 0 .05008 |
| CGB8 | 0 .435207749 | -1 .510453735 | 1 .22622 | -1 .2318 | 0 .21803 | 0 .2661 |
| CGGBP1 | 8881 .489706 | -0 .023558067 | 0 .08854 | -0 .2661 | 0 .79019 | 0 .8211 |
| CGN | 822 .6405321 | -0 .723137396 | 0 . 16438 | -4 .3992 | 1 .09E-05 | 2 .78E-05 |
| CGNL1 | 2024 .717625 | -0 .201819536 | 0 .25994 | -0 .7764 | 0 .43751 | 0 .49348 |
| CGREF1 | 472 .9446884 | 0 .351577234 | 0 .2283 | 1 .53995 | 0 . 12357 | 0 . 15962 |
| CGRRF1 | 1272 .403463 | -0 .901774799 | 0 . 13145 | -6 .86 | 6 .89E-12 | 4 . 10E-1 1 |
| CH25H | 205 .0502619 | 0 .633836818 | 0 .21021 | 3 .01524 | 0 .00257 | 0 .00453 |
| CHAC1 | 125 .2093338 | 2 .866772766 | 0 .33128 | 8 .65351 | 4 .99E-18 | 6 . 10E-17 |
| CHAC2 | 342 .2720855 | 0 . 142310869 | 0 . 1 1045 | 1 .28841 | 0 . 1976 | 0 .2441 |
| CHAD | 55 .23945547 | -0 .32087165 | 0 .21091 | -1 .5214 | 0 . 12816 | 0 . 16494 |
| CHADL | 560 .0312951 | -0 .302811795 | 0 .27361 | -1 . 1067 | 0 .26841 | 0 .32033 |
| CHAF1A | 727 .5625476 | 0 .587926586 | 0 . 1 1831 | 4 .96941 | 6 .72E-07 | 2 .06E-06 |
| CHAF1B | 467 .4075273 | 0 .365978515 | 0 . 1244 | 2 .94204 | 0 .00326 | 0 .00567 |
| CHAMP1 | 952 .5086043 | 0 .736100172 | 0 . 13958 | 5 .27382 | 1 .34E-07 | 4 .55E-07 |
| CHAT | 5 .227126715 | -3 .040626559 | 0 .72064 | -4 .2193 | 2 .45E-05 | 5 .95E-05 |
| CHCHD1 | 1370 .280583 | -0 .359684754 | 0 . 12861 | -2 .7966 | 0 .00516 | 0 .00867 |
| CHCHD10 | 265 .6089421 | 1 . 17267532 | 0 . 18431 | 6 .36242 | 1 .99E-10 | 9 .86E-10 |
| CHCHD2 | 17724 .73448 | 0 .010721522 | 0 . 15938 | 0 .06727 | 0 .94637 | 0 .95518 |
| CHCHD3 | 4409 .823973 | 0 .298165579 | 0 . 15007 | 1 .98686 | 0 .04694 | 0 .06608 |
| CHCHD4 | 633 .5529718 | -0 .085819506 | 0 . 1206 | -0 .7116 | 0 .4767 | 0 .53219 |
| CHCHD5 | 410 . 1095112 | -0 .099946878 | 0 . 1674 | -0 .597 | 0 .55048 | 0 .60314 |
| CHCHD6 | 5091 .628503 | -1 .467581271 | 0 .25589 | -5 .7351 | 9 .74E-09 | 3 .89E-08 |
| CHCHD7 | 2006 . 165858 | -0 .418016983 | 0 . 10339 | -4 .0432 | 5 .27E-05 | 0 .00012 |
| CHD1 | 9595 .950248 | 0 . 199621286 | 0 . 12259 | 1 .62835 | 0 . 10345 | 0 . 13583 |
| CHD1L | 4769 .328992 | 0 . 186092606 | 0 . 12953 | 1 .43666 | 0 . 15081 | 0 . 19109 |
| CHD2 | 12236 .63342 | -0 .263802974 | 0 .08953 | -2 .9465 | 0 .00321 | 0 .00559 |
| CHD3 | 5891 .775106 | 0 .270303946 | 0 . 12427 | 2 . 17511 | 0 .02962 | 0 .04332 |
| CHD4 | 16731 .90768 | 0 .426642477 | 0 .09003 | 4 .73896 | 2 . 15E-06 | 6 .07E-06 |
| CHD5 | 30 .82848736 | -0 .885165497 | 0 .33484 | -2 .6436 | 0 .0082 | 0 .01327 |
| CHD6 | 9183 .717003 | -0 .214095517 | 0 .08288 | -2 .5833 | 0 .00978 | 0 .01559 |
| CHD7 | 4158 .700271 | 0 .372822711 | 0 . 13031 | 2 .86103 | 0 .00422 | 0 .0072 |
| CHD8 | 8524 . 126052 | -0 .077257838 | 0 .07754 | -0 .9964 | 0 .31908 | 0 .37368 |
| CHD9 | 19155 . 10182 | -0 .640519855 | 0 .09277 | -6 .9045 | 5 .04E-12 | 3 .05E-1 1 |
| CHDH | 315 .939928 | 1 . 172158301 | 0 .23041 | 5 .08735 | 3 .63E-07 | 1 . 16E-06 |
| CHEK1 | 1026 .910228 | 0 .532023506 | 0 . 12216 | 4 .35508 | 1 .33E-05 | 3 .36E-05 |
| CHEK2 | 628 .0638504 | 0 .497750136 | 0 . 1 1 124 | 4 .47468 | 7 .65E-06 | 2 .00E-05 |
| CHERP | 444 .7723404 | 0 .801999272 | 0 . 13942 | 5 .7526 | 8 .79E-09 | 3 .53E-08 |
| CHFR | 2632 .078419 | 0 .69462628 | 0 .06605 | 10 .5167 | 7 .23E-26 | 2 . 10E-24 |
| CHGA | 36 .09451197 | 0 .043512502 | 0 .24179 | 0 . 17996 | 0 .85719 | 0 .87951 |
| CHGB | 20 .54774085 | -0 .985666288 | 0 .49371 | -1 .9964 | 0 .04589 | 0 .06472 |
| CHI3L1 | 3323 .858748 | 0 .609412668 | 0 .38537 | 1 .58135 | 0 . 1 138 | 0 . 14821 |
| CHI3L2 | 1254 .273704 | 1 . 1 16429159 | 0 .26844 | 4 . 1589 | 3 .20E-05 | 7 .62E-05 |
| CHIA | 1 1 .85822297 | -4 .442419807 | 0 .69578 | -6 .3848 | 1 .72E-10 | 8 .58E-10 |
| CHIC1 | 2571 .411035 | -0 .805066103 | 0 . 1 1 1 12 | -7 .2448 | 4 .33E-13 | 2 .98E-12 |
| CHIC2 | 2013 .300845 | -0 .246501629 | 0 .09712 | -2 .538 | 0 .011 15 | 0 .01756 |
| CHID1 | 2022 .665376 | 0 . 145984276 | 0 .08651 | 1 .68753 | 0 .0915 | 0 . 12167 |
| CHIT1 | 6791 .804564 | 6 .77305818 | 0 .53071 | 12 .7621 | 2 .67E-37 | 2 .44E-35 |
| CHKA | 1246 .915672 | 0 .548162528 | 0 . 13286 | 4 . 12599 | 3 .69E-05 | 8 .71E-05 |
| CHKB | 36 .94285739 | 0 .098566272 | 0 . 1 1738 | 0 .83971 | 0 .40107 | 0 .45769 |
| CHL1 | 44824 .64586 | -2 .661178176 | 0 .26059 | -10 .212 | 1 .75E-24 | 4 .34E-23 |
| CHM | 3808 .429179 | -0 .230542817 | 0 .09692 | -2 .3787 | 0 .01738 | 0 .02647 |
| CHML | 7001 .814154 | -1 .029817974 | 0 . 15742 | -6 .5417 | 6 .08E-1 1 | 3 .22E-10 |
| CHMP1A | 3925 .430814 | 0 .388655122 | 0 .07334 | 5 .29927 | 1 . 16E-07 | 4 .00E-07 |
| CHMP1B | 4560 .253966 | 0 .329570904 | 0 .08975 | 3 .67229 | 0 .00024 | 0 .0005 |

| CHMP2A CHMP2B CHMP3 CHMP4A CHMP4B CHMP4C CHMP5 CHMP6 CHMP7 CHN1 CHN2 CHODL CHORDC1 CHP1 CHP2 CHPF CHPF2 CHPT1 CHRAC1 CHRD CHRDL1 CHRDL2 | 2778 .300168 4500 . 12077 4914 .574856 73 .48337328 3062 .353721 574 .5050623 5048 .049344 797 .5083485 2956 .416996 789 .2030231 1608 .401747 156 .7852678 3657 .434526 19025 .83905 2358 .277804 2545 .882372 2895 .867541 3773 . 129909 898 .9408908 500 .9009713 2701 .288916 30 .48380568 |
| --- | --- |

CHRFAM7A 24 . 13509376

| CHRM1 CHRM2 CHRM3 CHRM4 CHRM5 CHRNA1 CHRNA10 CHRNA2 CHRNA3 CHRNA4 CHRNA5 CHRNA6 CHRNA7 CHRNA9 CHRNB1 CHRNB2 CHRNB3 CHRNB4 CHRND CHRNE CHRNG CHST1 CHST10 CHST11 CHST12 CHST13 CHST14 CHST15 CHST2 CHST3 CHST4 CHST5 CHST6 | 197 .6113868 43 .90228531 285 .7288909 20 .89723243 80 .27220066 131 .5649211 48 .41742207 22 .9567899 47 .30442189 10 .59748382 67 .64906638 60 .31543972 39 .23114341 222 .4323566 585 .6149302 252 .6300621 7 .422758418 16 .93422774 25 .31968115 107 .5396603 132 .461049 68 .59986677 887 .4699241 4726 .512158 632 .0709639 49 .09130063 586 .2641856 1402 .206326 940 .758097 2772 .923386 10 .3902842 6 .462743814 235 .2013618 |
| --- | --- |

-0 .040860593

-0 .344879702

-0 .417955877

0 .020797106

0 . 150901634

-0 .783004583

-0 .553000011

0 . 193244025

0 .700365143

1 .41311388

-0 .321538001

-1 .358120577

-0 .468714476

-0 .680604045

-2 .334869417

1 .388195012

1 .404341048

-0 .56374858

0 .280675582

0 .757933185

-0 .297309979

0 .671300634

0 .510727622

-1 . 194117508

-2 .622034046

1 .425735615

-1 .810998208

-0 .911553022

3 .237918635

-0 .688587332

-0 .946905538

0 .803670582

-2 . 162150655

1 .209887452

2 .027050014

0 .733735039

0 .52420494

0 .079853477

0 . 160763426

-2 .83023148

-1 .658026388

-0 . 130052959

-0 .323082204

-0 .532346542

2 .35262968

1 . 164991127

1 .714761745

0 .813277479

2 .656912809

0 .61316802

0 .560086186

2 .657978816

0 .356412396

-0 .956054264

-1 . 164290314

2 . 133446329

0 . 14848 0 .09607 0 .08611 0 . 14821 0 . 1041 0 .25769 0 . 12773 0 .08018 0 .09337 0 . 18948 0 . 18418 0 . 18614 0 . 12134 0 . 13719 0 .34746 0 . 16833 0 . 1 1444 0 . 10042 0 . 12283 0 . 1951 0 .30823 0 .37026 0 .28007 0 .30812 0 .54312 0 .31 0 .31408 0 . 14929 0 .4086 0 . 14277 0 .27708 0 .28428 0 .53267 0 .25002 0 .4472 0 .2754 0 .31684 0 .09571 0 .22525 0 .59808 0 .4126 0 .30772 0 .20808 0 . 19052 0 .25326 0 . 13259 0 . 1832 0 . 10355 0 .24837 0 . 12131 0 . 1316 0 . 17048 0 . 10839 0 .4008 0 .44885 0 .25704

-0 .2752 -3 .59 -4 .8538 0 . 14033 1 .44953 -3 .0385 -4 .3295 2 .41001 7 .50089 7 .45791 -1 .7458 -7 .2964 -3 .8628 -4 .9609 -6 .7199 8 .24707 12 .2719 -5 .6138 2 .28505 3 .88489 -0 .9646 1 .81306 1 .82358 -3 .8755 -4 .8277 4 .59908 -5 .7661 -6 . 1061 7 .92439 -4 .8232 -3 .4174 2 .82708 -4 .0591 4 .83925 4 .53276 2 .66425 1 .6545 0 .83434 0 .71372 -4 .7322 -4 .0185 -0 .4226 -1 .5527 -2 .7942 9 .28947 8 .78613 9 .36024 7 .85388 10 .6974 5 .05445 4 .25596 15 .5913 3 .28838 -2 .3853 -2 .594 8 .30015

0 .78316 0 .00033 1 .21E-06 0 .8884 0 . 14719 0 .00238 1 .49E-05 0 .01595 6 .34E-14 8 .79E-14 0 .08084 2 .96E-13 0 .00011 7 .02E-07 1 .82E-1 1 1 .62E-16 1 .28E-34 1 .98E-08 0 .02231 0 .0001 0 .33475 0 .06982 0 .06822 0 .00011 1 .38E-06 4 .24E-06 8 . 1 1E-09 1 .02E-09 2 .29E-15 1 .41E-06 0 .00063 0 .0047 4 .93E-05 1 .30E-06 5 .82E-06 0 .00772 0 .09803 0 .40409 0 .4754 2 .22E-06 5 .86E-05 0 .67256 0 . 12049 0 .0052 1 .55E-20 1 .55E-18 7 .96E-21 4 .03E-15 1 .05E-26 4 .32E-07 2 .08E-05 8 .35E-55 0 .00101 0 .01706 0 .00949 1 .04E-16

0 .81467 0 .00067 3 .56E-06 0 .90631 0 . 18696 0 .00422 3 .75E-05 0 .02445 4 .78E-13 6 .49E-13 0 . 10856 2 .07E-12 0 .00024 2 . 14E-06 1 .03E-10 1 .66E-15 8 .82E-33 7 .58E-08 0 .03341 0 .00022 0 .3898 0 .09491 0 .09295 0 .00023 4 .02E-06 1 . 15E-05 3 .28E-08 4 .64E-09 2 .06E-14 4 . 1 1E-06 0 .00123 0 .00794 0 .00011 3 .81E-06 1 .55E-05 0 .01254 0 . 12933 0 .46067 0 .53092 6 .26E-06 0 .00013 0 .71665 0 . 15598 0 .00873 2 .53E-19 2 .01E-17 1 .33E-19 3 .50E-14 3 .29E-25 1 .36E-06 5 . 12E-05 3 .91E-52 0 .0019 0 .02605 0 .01516 1 .09E-15

| CHST7 CHST8 CHST9 CHSY1 CHSY3 CHTF18 CHTF8 CHTOP CHUK CHURC1 CIAO1 CIAPIN1 CIART CIB1 CIB2 CIB3 CIB4 CIC CIDEA CIDEC CIITA CILP CILP2 CINP CIPC CIR1 CIRBP CISD1 CISD2 CISH CIT CITED1 CITED2 CITED4 CIZ1 CKAP2 CKAP2L CKAP4 CKAP5 CKB CKLF | 291 .7133041 19 .47609347 538 .6167289 4020 . 124543 1245 .279281 518 .7101893 1387 .816566 2960 .863283 3091 .987888 4300 .245756 5373 .035678 4020 .459504 262 .6590825 1938 . 167357 294 .6329999 2 .578268617 5 .031964591 2367 .549504 959 .3847259 106 .7907377 3032 . 174054 1474 .431551 154 .5572144 1926 .660456 2950 .439725 1414 .308044 15416 . 1352 2786 . 193505 2443 .773631 485 .8645446 1599 .576305 299 .881029 1915 .070182 210 .9301896 1559 .769707 2679 .557454 475 .8080768 4277 .000085 8470 .232246 1394 .518611 382 . 1386234 |
| --- | --- |

CKLF-CMTM 7 .721928404

| CKM CKMT1A CKMT1B CKMT2 CKS1B CKS1BP3 CKS2 CLASP1 CLASP2 CLASRP CLC CLCA1 CLCA2 CLCA4 | 23 .58412848 1 13 .9023079 195 .73057 680 .5909059 1225 .71511 1 7 .807158042 1211 .278755 7754 .262151 13093 .88905 531 .0249027 5 .620699857 10 .56763871 1 1204 .86098 666 .3670966 |
| --- | --- |

1 .207658273

0 .726037469

-0 .975977559

0 .903522229

0 .449935629

0 .808921699

0 . 108811742

-0 .082138623

0 .031644372

-0 .223572588

0 .595121554

0 .209892342

-0 .494137329

0 .866766671

0 .221084045

-2 .214789871

-1 .909637125

0 .466780683

1 . 102363103

2 .236481649

1 .819723595

0 .886692672

1 .665668118

0 .07575037

-0 .023596419

-0 .088656437

-1 . 162828861

-0 .650001335

-0 .048297296

0 .884223453

1 .233529493

3 .304401211

0 .084529102

1 .870210737

0 .035803965

0 .772954036

1 .599585923

1 . 173998308

0 .23332172

-0 .622035117

0 .542117328

0 .952501076

0 .432763364

-0 .771041604

-0 .671873924

-1 .060957204

0 .377063936

-0 .456936092

1 .567220742

-0 . 176684901

-0 .798353812

0 .322405203

-2 .445598972

-4 . 1711 13135

0 .228611773

-1 .602091658

0 . 14448 0 .43247 0 .366 0 .095 0 . 17943 0 . 1 1287 0 .0824 0 .06573 0 .08923 0 .09003 0 .08836 0 . 12027 0 .216 0 . 1 138 0 . 14602 0 .63609 0 .47264 0 . 14053 0 .46263 0 .43828 0 . 18449 0 .33317 0 .2818 0 . 1 1262 0 .07787 0 .08067 0 . 13006 0 . 14705 0 . 14506 0 . 16515 0 . 16315 0 .45416 0 . 12654 0 .2082 0 . 13176 0 . 12342 0 . 17839 0 .08661 0 .08658 0 . 18614 0 . 14223 0 .25906 0 .23139 0 .28151 0 .26278 0 .20433 0 . 15141 0 .27492 0 .22267 0 .08159 0 .09121 0 . 1 1483 0 .64372 0 .50466 0 .26166 0 .32766

8 .35868 1 .67881 -2 .6666 9 .51096 2 .50765 7 . 16705 1 .32056 -1 .2496 0 .35464 -2 .4832 6 .73533 1 .74522 -2 .2876 7 .6166 1 .51412 -3 .4819 -4 .0404 3 .32148 2 .38279 5 . 10283 9 .86363 2 .66137 5 .9109 0 .67262 -0 .303 -1 .099 -8 .9409 -4 .4204 -0 .3329 5 .35421 7 .56088 7 .27585 0 .668 8 .98275 0 .27173 6 .26302 8 .96666 13 .5552 2 .69474 -3 .3417 3 .81149 3 .67677 1 .87031 -2 .739 -2 .5568 -5 . 1924 2 .49027 -1 .662 7 .03841 -2 . 1655 -8 .7534 2 .80767 -3 .7992 -8 .2651 0 .87369 -4 .8895

6 .34E-17 0 .09319 0 .00766 1 .89E-21 0 .01215 7 .66E-13 0 . 18665 0 .21143 0 .72286 0 .01302 1 .64E-1 1 0 .08095 0 .02216 2 .60E-14 0 . 13 0 .0005 5 .34E-05 0 .0009 0 .01718 3 .35E-07 5 .98E-23 0 .00778 3 .40E-09 0 .50119 0 .76189 0 .27176 3 .86E-19 9 .85E-06 0 .73917 8 .59E-08 4 .00E-14 3 .44E-13 0 .50413 2 .64E-19 0 .78583 3 .78E-10 3 .06E-19 7 .38E-42 0 .00704 0 .00083 0 .00014 0 .00024 0 .06144 0 .00616 0 .01056 2 .08E-07 0 .01276 0 .0965 1 .94E-12 0 .03035 2 .07E-18 0 .00499 0 .00015 1 .40E-16 0 .38229 1 .01E-06

6 .84E-16 0 . 12363 0 .01246 3 .36E-20 0 .01903 5 . 13E-12 0 .23203 0 .25901 0 .76232 0 .02028 9 .30E-1 1 0 . 10869 0 .0332 2 .06E-13 0 . 16711 0 .00098 0 .00012 0 .0017 0 .02621 1 .07E-06 1 .25E-21 0 .01264 1 .44E-08 0 .55612 0 .79569 0 .32389 5 .34E-18 2 .53E-05 0 .7762 3 .02E-07 3 . 1 1E-13 2 .39E-12 0 .55891 3 .75E-18 0 .81714 1 .81E-09 4 .29E-18 1 .02E-39 0 .01154 0 .00159 0 .0003 0 .00049 0 .08442 0 .0102 0 .01672 6 .85E-07 0 .01992 0 . 12754 1 .24E-1 1 0 .0443 2 .65E-17 0 .0084 0 .00031 1 .44E-15 0 .4385 3 .01E-06

| CLCC1 | 1611 .539637 | -0 .232517448 | 0 .08751 | -2 .6572 | 0 .00788 | 0 .01279 |
| --- | --- | --- | --- | --- | --- | --- |
| CLCF1 | 180 .0643455 | -0 .275001791 | 0 . 16357 | -1 .6813 | 0 .0927 | 0 . 12305 |
| CLCN1 | 14 .79679535 | -1 .35641132 | 0 .37719 | -3 .5961 | 0 .00032 | 0 .00066 |
| CLCN2 | 266 .7509469 | 0 .542608091 | 0 . 13194 | 4 . 1 1255 | 3 .91E-05 | 9 . 19E-05 |
| CLCN3 | 15204 .20155 | -0 .419215781 | 0 . 10304 | -4 .0686 | 4 .73E-05 | 0 .00011 |
| CLCN4 | 602 . 164513 | 0 .810284074 | 0 .21324 | 3 .79994 | 0 .00014 | 0 .00031 |
| CLCN5 | 3790 .318922 | -0 . 143243637 | 0 . 10449 | -1 .3709 | 0 . 1704 | 0 .21372 |
| CLCN6 | 1248 .333629 | -0 .081659952 | 0 . 1 1591 | -0 .7045 | 0 .4811 1 | 0 .5363 |
| CLCN7 | 5453 .89764 | 0 .87455895 | 0 . 14348 | 6 .09531 | 1 .09E-09 | 4 .94E-09 |
| CLCNKA | 16 .95149467 | 0 .528316428 | 0 .42321 | 1 .24836 | 0 .2119 | 0 .25954 |
| CLCNKB | 12 .81340938 | -0 .678061168 | 0 .32576 | -2 .0815 | 0 .03739 | 0 .05358 |
| CLDN1 | 13236 .41809 | -1 .364419309 | 0 .26465 | -5 . 1555 | 2 .53E-07 | 8 .25E-07 |
| CLDN10 | 172 .8472456 | 3 .200856415 | 0 .4074 | 7 .8568 | 3 .94E-15 | 3 .43E-14 |
| CLDN11 | 600 .8826568 | -0 .344984341 | 0 .25181 | -1 .37 | 0 . 17069 | 0 .21402 |
| CLDN12 | 3077 .523298 | 0 .366679787 | 0 . 15928 | 2 .3021 | 0 .02133 | 0 .03206 |
| CLDN14 | 59 .44668491 | 2 .267536438 | 0 .43668 | 5 . 19261 | 2 .07E-07 | 6 .85E-07 |
| CLDN15 | 294 .7449981 | 0 .69364241 | 0 . 16362 | 4 .23925 | 2 .24E-05 | 5 .48E-05 |
| CLDN16 | 98 .67622391 | -1 .764197586 | 0 .27964 | -6 .3088 | 2 .81E-10 | 1 .37E-09 |
| CLDN17 | 21 .2808698 | 2 .424139885 | 0 .6047 | 4 .00883 | 6 . 10E-05 | 0 .00014 |
| CLDN18 | 173 . 1599643 | -1 .397018533 | 0 . 19946 | -7 .004 | 2 .49E-12 | 1 .57E-1 1 |
| CLDN19 | 18 .36407265 | 0 .601583222 | 0 .43502 | 1 .38289 | 0 . 1667 | 0 .20949 |
| CLDN2 | 47 .08415403 | -1 .415808334 | 0 .30893 | -4 .583 | 4 .58E-06 | 1 .24E-05 |
| CLDN20 | 170 .6316381 | -0 .733306865 | 0 . 16625 | -4 .4109 | 1 .03E-05 | 2 .64E-05 |
| CLDN23 | 67 . 17178404 | 0 .364234925 | 0 . 19125 | 1 .90452 | 0 .05684 | 0 .07866 |
| CLDN24 | 16 .75328093 | -1 .435946846 | 0 .21679 | -6 .6238 | 3 .50E-1 1 | 1 .92E-10 |
| CLDN25 | 1 .57771855 | -3 .447769921 | 0 .71022 | -4 .8545 | 1 .21E-06 | 3 .55E-06 |
| CLDN3 | 47 . 17167383 | 1 .884586657 | 0 .42552 | 4 .42894 | 9 .47E-06 | 2 .44E-05 |
| CLDN34 | 8 .464443303 | 0 .216689989 | 0 .3635 | 0 .59612 | 0 .5511 | 0 .60365 |
| CLDN4 | 2949 .384593 | -0 .910743463 | 0 .25953 | -3 .5092 | 0 .00045 | 0 .00089 |
| CLDN5 | 864 .0398695 | 1 .86612735 | 0 .20685 | 9 .02158 | 1 .85E-19 | 2 .68E-18 |
| CLDN6 | 3 .095350395 | -1 . 175740367 | 0 .56915 | -2 .0658 | 0 .03885 | 0 .05551 |
| CLDN7 | 177 .3154601 | 0 .869448337 | 0 . 18017 | 4 .82558 | 1 .40E-06 | 4 .06E-06 |
| CLDN8 | 479 .889182 | -0 .443118069 | 0 .42874 | -1 .0335 | 0 .30136 | 0 .35506 |
| CLDN9 | 3 . 161490545 | -1 .542639232 | 0 .55022 | -2 .8037 | 0 .00505 | 0 .00849 |
| CLDND1 | 4952 .226875 | -1 . 13916106 | 0 .08705 | -13 .087 | 3 .93E-39 | 4 . 14E-37 |
| CLDND2 | 28 .65558112 | -0 .08703149 | 0 . 18184 | -0 .4786 | 0 .6322 | 0 .68014 |
| CLEC10A | 1718 .299082 | 0 .465557672 | 0 .24883 | 1 .87101 | 0 .06134 | 0 .08431 |
| CLEC11A | 1768 .247185 | 1 . 105199871 | 0 .22935 | 4 .81877 | 1 .44E-06 | 4 . 19E-06 |
| CLEC12A | 318 .2274915 | 1 .27955289 | 0 .26694 | 4 .79342 | 1 .64E-06 | 4 .72E-06 |
| CLEC12B | 269 . 1346307 | -0 . 163426093 | 0 .33418 | -0 .489 | 0 .62481 | 0 .67355 |
| CLEC14A | 897 .2671784 | 1 .03049469 | 0 . 14744 | 6 .98908 | 2 .77E-12 | 1 .74E-1 1 |
| CLEC16A | 2144 .996285 | 0 .272360845 | 0 .09485 | 2 .87162 | 0 .00408 | 0 .00698 |
| CLEC17A | 35 .29949138 | 1 .465467935 | 0 .42433 | 3 .45362 | 0 .00055 | 0 .00108 |
| CLEC18A | 25 .481932 | -0 .53943544 | 0 .27055 | -1 .9938 | 0 .04617 | 0 .06509 |
| CLEC18B | 19 .41399616 | -1 .201908779 | 0 .31701 | -3 .7913 | 0 .00015 | 0 .00032 |
| CLEC18C | 5 .819388979 | -0 .812695455 | 0 .33295 | -2 .4409 | 0 .01465 | 0 .02261 |
| CLEC19A | 3 .7114145 | -4 . 148484382 | 0 .68595 | -6 .0478 | 1 .47E-09 | 6 .53E-09 |
| CLEC1A | 259 .5699398 | 0 . 163302236 | 0 . 19335 | 0 .84458 | 0 .39835 | 0 .45499 |
| CLEC1B | 74 .9427267 | 0 .479640024 | 0 .26123 | 1 .83611 | 0 .06634 | 0 .0906 |
| CLEC2A | 1062 .655613 | -2 .488429161 | 0 .32484 | -7 .6605 | 1 .85E-14 | 1 .49E-13 |
| CLEC2B | 3280 . 174488 | -1 . 178866719 | 0 . 15734 | -7 .4924 | 6 .76E-14 | 5 .06E-13 |
| CLEC2D | 1833 .643071 | 0 . 107264763 | 0 . 15302 | 0 .70099 | 0 .48331 | 0 .53838 |
| CLEC2L | 9 .414422196 | 0 .354055571 | 0 .4325 | 0 .81863 | 0 .413 | 0 .46954 |
| CLEC3A | 9 .490859553 | -3 .78015309 | 0 .81186 | -4 .6562 | 3 .22E-06 | 8 .88E-06 |
| CLEC3B | 742 .9114233 | -0 .366348214 | 0 .28774 | -1 .2732 | 0 .20295 | 0 .24992 |
| CLEC4A | 332 .6607187 | 0 .432155619 | 0 . 16993 | 2 .54315 | 0 .01099 | 0 .01733 |

| CLEC4C | 95 .33928721 | 2 . 136847662 | 0 .30218 | 7 .07133 | 1 .53E-12 | 9 .95E-12 |
| --- | --- | --- | --- | --- | --- | --- |
| CLEC4D | 46 .3899877 | 1 .275622465 | 0 .35223 | 3 .62152 | 0 .00029 | 0 .0006 |
| CLEC4E | 106 .3656833 | 1 .828368288 | 0 .3344 | 5 .46758 | 4 .56E-08 | 1 .66E-07 |
| CLEC4F | 53 .43750886 | -0 .332664764 | 0 .29465 | -1 . 129 | 0 .25889 | 0 .31022 |
| CLEC4G | 82 .4373166 | 1 .938729267 | 0 .29453 | 6 .58248 | 4 .63E-1 1 | 2 .49E-10 |
| CLEC4M | 9 .022886315 | -2 .784480107 | 0 .45689 | -6 .0944 | 1 . 10E-09 | 4 .96E-09 |
| CLEC5A | 419 .2816341 | 0 .607116545 | 0 . 1722 | 3 .52573 | 0 .00042 | 0 .00084 |
| CLEC6A | 49 .72863481 | 1 .58328733 | 0 .46287 | 3 .42056 | 0 .00062 | 0 .00122 |
| CLEC7A | 2043 .460194 | 1 .777672344 | 0 . 16684 | 10 .6547 | 1 .66E-26 | 5 .08E-25 |
| CLEC9A | 103 .9950975 | 0 .386790285 | 0 .23121 | 1 .67292 | 0 .09434 | 0 . 12499 |
| CLECL1 | 77 .90719158 | 0 .487875513 | 0 .23239 | 2 .09937 | 0 .03578 | 0 .05148 |
| CLGN | 32 .50741988 | -0 .849462269 | 0 .32953 | -2 .5778 | 0 .00994 | 0 .01583 |
| CLHC1 | 445 .5968864 | -0 .638913711 | 0 . 17443 | -3 .6629 | 0 .00025 | 0 .00052 |
| CLIC1 | 18432 .39672 | 0 . 198798785 | 0 . 12256 | 1 .62209 | 0 . 10478 | 0 . 13748 |
| CLIC2 | 1772 .245317 | 0 .218492023 | 0 . 18375 | 1 . 18908 | 0 .23441 | 0 .28406 |
| CLIC3 | 772 .0693377 | -0 .271616575 | 0 .22735 | -1 . 1947 | 0 .2322 | 0 .28164 |
| CLIC4 | 25992 .09743 | 0 .485712503 | 0 . 12684 | 3 .82941 | 0 .00013 | 0 .00028 |
| CLIC5 | 150 .9283897 | 0 .441971199 | 0 .21882 | 2 .01983 | 0 .0434 | 0 .06149 |
| CLIC6 | 373 .2640241 | 1 .632210617 | 0 . 1968 | 8 .2937 | 1 . 10E-16 | 1 . 15E-15 |
| CLINT1 | 8797 .814275 | -0 .069019664 | 0 .08369 | -0 .8247 | 0 .40954 | 0 .46605 |
| CLIP1 | 13866 .56147 | 0 . 126560265 | 0 .09887 | 1 .28004 | 0 .20053 | 0 .24727 |
| CLIP2 | 1733 .246297 | 0 .885431752 | 0 . 13156 | 6 .73013 | 1 .70E-1 1 | 9 .62E-1 1 |
| CLIP3 | 177 .796488 | 0 .666063627 | 0 . 16516 | 4 .03278 | 5 .51E-05 | 0 .00013 |
| CLIP4 | 6626 .805416 | -0 .658063916 | 0 . 10327 | -6 .3724 | 1 .86E-10 | 9 .27E-10 |
| CLK1 | 9860 .753377 | -0 .839895421 | 0 . 12677 | -6 .6254 | 3 .46E-1 1 | 1 .90E-10 |
| CLK2 | 1541 .01198 | -0 .045573376 | 0 .07925 | -0 .5751 | 0 .56523 | 0 .61737 |
| CLK3 | 1771 .801563 | -0 .262650501 | 0 .07905 | -3 .3224 | 0 .00089 | 0 .0017 |
| CLK4 | 3308 .956189 | -0 .976597185 | 0 . 12797 | -7 .6316 | 2 .32E-14 | 1 .85E-13 |
| CLLU1 | 25 .89388783 | -2 .227216256 | 0 .3696 | -6 .0261 | 1 .68E-09 | 7 .40E-09 |
| CLLU1OS | 7 .974034901 | -0 .697243342 | 0 .40944 | -1 .7029 | 0 .08859 | 0 . 1 1813 |
| CLMN | 2026 .780334 | 0 . 169433024 | 0 . 15414 | 1 .09919 | 0 .27169 | 0 .32385 |
| CLMP | 1540 .546078 | 0 .942432951 | 0 .27713 | 3 .40065 | 0 .00067 | 0 .0013 |
| CLN3 | 214 .0893979 | 0 .422412375 | 0 .083 | 5 .08909 | 3 .60E-07 | 1 . 15E-06 |
| CLN5 | 3391 .056466 | -0 .21003813 | 0 . 10222 | -2 .0548 | 0 .0399 | 0 .05689 |
| CLN6 | 2170 .041194 | 1 .073007948 | 0 . 13033 | 8 .23312 | 1 .82E-16 | 1 .86E-15 |
| CLN8 | 3083 .325625 | 0 .21238026 | 0 . 1 1907 | 1 .78372 | 0 .07447 | 0 . 10077 |
| CLNK | 157 . 1997385 | 0 .24712717 | 0 .24524 | 1 .00768 | 0 .31361 | 0 .36812 |
| CLNS1A | 8727 .500681 | -0 .295596234 | 0 . 17812 | -1 .6596 | 0 .097 | 0 . 12809 |
| CLOCK | 7727 .740028 | -0 .012247137 | 0 . 10816 | -0 . 1 132 | 0 .90985 | 0 .92519 |
| CLP1 | 1271 .479053 | 0 .081311623 | 0 . 12107 | 0 .67162 | 0 .50183 | 0 .55671 |
| CLPB | 1653 . 141215 | 0 .731644952 | 0 . 1 1 169 | 6 .55061 | 5 .73E-1 1 | 3 .04E-10 |
| CLPP | 1226 .841673 | 0 .2866083 | 0 . 1303 | 2 . 19957 | 0 .02784 | 0 .04093 |
| CLPS | 1 .256154705 | -3 .236428983 | 0 .81024 | -3 .9944 | 6 .49E-05 | 0 .00015 |
| CLPSL1 | 3 .076556599 | -2 .395878464 | 0 .94773 | -2 .528 | 0 .01147 | 0 .01803 |
| CLPSL2 | 6 . 1 10503253 | 1 .743403187 | 0 .84643 | 2 .05971 | 0 .03943 | 0 .05626 |
| CLPTM1 | 2184 .697685 | 0 .804883345 | 0 . 1314 | 6 . 12527 | 9 .05E-10 | 4 . 14E-09 |
| CLPTM1L | 4962 .303217 | 0 .976009094 | 0 .09966 | 9 .79369 | 1 .20E-22 | 2 .42E-21 |
| CLPX | 5528 .5515 | 0 .076433577 | 0 .07245 | 1 .05498 | 0 .29143 | 0 .34478 |
| CLRN1 | 8 .221554019 | -3 .941350346 | 0 .60717 | -6 .4913 | 8 .51E-1 1 | 4 .40E-10 |
| CLRN2 | 1 .638833403 | -3 .297479772 | 1 . 15557 | -2 .8536 | 0 .00432 | 0 .00735 |
| CLRN3 | 3 .791299384 | -2 .512473131 | 0 .63523 | -3 .9552 | 7 .65E-05 | 0 .00017 |
| CLSPN | 467 .9644846 | 2 .000155968 | 0 .22421 | 8 .92104 | 4 .62E-19 | 6 .35E-18 |
| CLSTN1 | 9337 .622742 | 0 .388663836 | 0 . 12453 | 3 . 12105 | 0 .0018 | 0 .00326 |
| CLSTN2 | 712 .2273786 | 1 . 105061413 | 0 .28725 | 3 .84698 | 0 .00012 | 0 .00026 |
| CLSTN3 | 1738 .985585 | 1 .791076381 | 0 .25531 | 7 .01538 | 2 .29E-12 | 1 .46E-1 1 |
| CLTA | 8506 .507495 | -0 .04554134 | 0 . 12625 | -0 .3607 | 0 .7183 | 0 .75834 |

| CLTB  CLTC  CLTCL1  CLU  CLUAP1  CLUH  CLUL1  CLVS1  CLVS2  CLYBL  CMA1  CMAS | 6574 .014324 52147 .07667 728 .6204517 3518 .472581 956 .0961529 3152 . 177542 63 .24438158 1 12 .2801638 171 .5300669 603 .9333883 400 . 1696715 2063 .857054 |
| --- | --- |

CMB9-22P1359 .84455539 CMB9-55A1822 .84870144

| CMBL CMC1 CMC2 CMIP CMKLR1 CMPK1 CMPK2 CMSS1 CMTM1 CMTM2 CMTM3 CMTM4 CMTM5 CMTM6 CMTM7 CMTM8 CMTR1 CMTR2 CMYA5 CNBD1 CNBD2 CNBP CNDP1 CNDP2 CNEP1R1 CNFN CNGA1 CNGA2 CNGA3 CNGA4 CNGB1 CNGB3 CNIH1 CNIH2 CNIH3 CNIH4 CNKSR1 CNKSR2 CNKSR3 CNN1 CNN2 CNN3 | 565 .8735474 1 165 .632896 1240 .234695 1564 .710036 2136 .201865 15537 .30298 1053 .221593 1539 . 174089 64 .97401298 14 . 10290017 2055 .692028 3586 .095184 334 .2519123 12080 .22855 506 .5794159 1 10 .5464814 4532 .608718 4187 .529839 1586 .504006 20 .36206433 30 .91548027 21882 .09433 108 .5485171 4036 . 12957 1942 .008453 957 .0205049 190 .9075983 3 .977569123 16 . 13742499 9 .046215322 34 .80875731 59 .55214014 4806 .502147 42 .27035872 1516 .093351 4220 .638812 162 .6737294 499 .8529527 2687 .373663 606 .3646021 3282 .974962 1 1440 .30577 |
| --- | --- |

-0 .417152806

0 .634872461

-0 .085025529

0 .650402874

-0 .225606989

0 .783060614

-0 .826049007

-1 . 126636919

-1 .329175665

-0 .414484779

-0 .734271955

0 .039371821

0 .074784937

-0 .719888123

-0 .345084582

-0 .494253957

0 .081960492

0 .850326997

1 .237197258

-0 .508356129

1 .417847041

-0 .372264056

0 .494188634

0 . 154515403

0 .840144825

0 .004944397

-1 .38929697

-0 . 199546047

0 .311 169568

-0 .444054209

0 .5373669

-0 .049844751

-1 .330779907

-2 .228829969

-1 .691674841

-0 .410382378

1 . 15370628

0 .996534341

0 .247790826

0 .500431372

-1 . 10992472

-4 .490342956

-2 .061720066

-0 .794273325

0 .095739885

-1 .427029357

-0 .674904765

0 .074904172

1 .014074722

-0 .04262808

-0 .474763283

-1 .445650368

-0 .297250673

3 .229218863

1 .51399075

0 .260837468

0 . 14299 0 . 10835 0 . 13923 0 .29004 0 .08372 0 . 12144 0 .31126 0 .24626 0 .47107 0 . 13072 0 .30258 0 . 1 1013 0 .22031 0 . 1523 0 .20837 0 .08159 0 . 13524 0 . 14894 0 . 15643 0 .09866 0 .223 0 . 10256 0 . 1239 0 .33113 0 . 10282 0 . 13465 0 .3982 0 . 10237 0 . 12092 0 .20358 0 .09234 0 . 1 1 193 0 .25403 0 .41805 0 .23763 0 .07834 0 .4216 0 . 1 1019 0 . 1 1518 0 .30186 0 .24113 0 .83448 0 .39291 0 .343 0 .38871 0 .21201 0 . 13076 0 . 13444 0 .27556 0 . 1 1275 0 .23309 0 .21248 0 . 14596 0 .34265 0 . 13462 0 . 12253

-2 .9174 5 .85924 -0 .6107 2 .24247 -2 .6948 6 .4479 -2 .6539 -4 .5749 -2 .8216 -3 . 1708 -2 .4267 0 .35749 0 .33945 -4 .7266 -1 .6561 -6 .0579 0 .60606 5 .70919 7 .90911 -5 . 1525 6 .35793 -3 .6298 3 .98863 0 .46663 8 . 17132 0 .03672 -3 .4889 -1 .9492 2 .57336 -2 . 1813 5 .81956 -0 .4453 -5 .2387 -5 .3315 -7 . 1 19 -5 .2382 2 .73651 9 .04345 2 . 15127 1 .65785 -4 .6031 -5 .381 -5 .2472 -2 .3156 0 .2463 -6 .7311 -5 . 1614 0 .55716 3 .68005 -0 .3781 -2 .0368 -6 .8038 -2 .0365 9 .42427 1 1 .2462 2 . 12881

0 .00353 4 .65E-09 0 .54142 0 .02493 0 .00704 1 . 13E-10 0 .00796 4 .76E-06 0 .00478 0 .00152 0 .01524 0 .72072 0 .73427 2 .28E-06 0 .09771 1 .38E-09 0 .54448 1 . 14E-08 2 .59E-15 2 .57E-07 2 .04E-10 0 .00028 6 .65E-05 0 .64076 3 .05E-16 0 .97071 0 .00048 0 .05127 0 .01007 0 .02916 5 .90E-09 0 .65608 1 .62E-07 9 .74E-08 1 .09E-12 1 .62E-07 0 .00621 1 .52E-19 0 .03145 0 .09735 4 . 16E-06 7 .41E-08 1 .54E-07 0 .02058 0 .80545 1 .68E-1 1 2 .45E-07 0 .57742 0 .00023 0 .70537 0 .04167 1 .02E-1 1 0 .0417 4 .33E-21 2 .42E-29 0 .03327

0 .0061 1 .94E-08 0 .59464 0 .03701 0 .01154 5 .79E-10 0 .0129 1 .28E-05 0 .00807 0 .00278 0 .02344 0 .7604 0 .77202 6 .42E-06 0 . 12894 6 . 15E-09 0 .59752 4 .50E-08 2 .31E-14 8 .38E-07 1 .01E-09 0 .00058 0 .00015 0 .68788 3 .04E-15 0 .97477 0 .00096 0 .0716 0 .01601 0 .04271 2 .43E-08 0 .70181 5 .42E-07 3 .39E-07 7 . 16E-12 5 .43E-07 0 .01027 2 .21E-18 0 .04578 0 . 1285 1 . 13E-05 2 .62E-07 5 . 19E-07 0 .03102 0 .83455 9 .57E-1 1 8 .01E-07 0 .62902 0 .00049 0 .74692 0 .05922 5 .92E-1 1 0 .05926 7 .44E-20 9 .96E-28 0 .04815

| CNNM1 | 413 .3260506 | -0 .206505585 | 0 . 1923 -1 .0739 | 0 .28289 | 0 .33582 |
| --- | --- | --- | --- | --- | --- |
| CNNM2 | 1383 .294094 | -0 .383937676 | 0 . 1 1734 -3 .272 | 0 .00107 | 0 .00201 |
| CNNM3 | 1049 .358058 | 0 .001347404 | 0 .09515 0 .01416 | 0 .9887 | 0 .99035 |
| CNNM4 | 978 .7027259 | 0 .033213582 | 0 .07475 0 .4443 | 0 .65683 | 0 .70253 |
| CNOT1 | 21007 .00096 | 0 .036740293 | 0 .0702 0 .52339 | 0 .6007 | 0 .6511 1 |
| CNOT10 | 2038 .919884 | -0 .233659714 | 0 .05063 -4 .6155 | 3 .92E-06 | 1 .07E-05 |
| CNOT11 | 1881 . 137408 | 0 .627536545 | 0 .05238 1 1 .98 | 4 .53E-33 | 2 .70E-31 |
| CNOT2 | 7842 .043668 | -0 .630021169 | 0 .05893 -10 .69 | 1 . 13E-26 | 3 .53E-25 |
| CNOT3 | 522 .623692 | 0 .441259099 | 0 . 14763 2 .98903 | 0 .0028 | 0 .00491 |
| CNOT4 | 2273 .088656 | -0 .489513497 | 0 .08949 -5 .4702 | 4 .50E-08 | 1 .64E-07 |
| CNOT6 | 4225 .254108 | 0 . 180856333 | 0 .07298 2 .47823 | 0 .0132 | 0 .02054 |
| CNOT6L | 8235 .472339 | -0 .026831191 | 0 . 10337 -0 .2596 | 0 .79519 | 0 .82544 |
| CNOT7 | 6016 .89669 | 0 . 135970998 | 0 .07652 1 .77695 | 0 .07558 | 0 . 10216 |
| CNOT8 | 4110 .996097 | -0 .033209132 | 0 .06258 -0 .5307 | 0 .59565 | 0 .64625 |
| CNOT9 | 4429 .539205 | 0 .331127874 | 0 .05667 5 .84353 | 5 . 1 1E-09 | 2 . 12E-08 |
| CNP | 6709 .297119 | 0 .765724742 | 0 . 1 1472 6 .67447 | 2 .48E-1 1 | 1 .38E-10 |
| CNPPD1 | 3071 .920597 | 0 .284676289 | 0 .09671 2 .94354 | 0 .00324 | 0 .00564 |
| CNPY1 | 1 1 . 16986538 | -1 .342731079 | 0 .5911 1 -2 .2715 | 0 .02311 | 0 .03451 |
| CNPY2 | 488 .8842998 | -0 .044072415 | 0 . 13158 -0 .335 | 0 .73766 | 0 .77482 |
| CNPY3 | 1944 .502689 | 0 .493622256 | 0 . 1 156 4 .27021 | 1 .95E-05 | 4 .82E-05 |
| CNPY4 | 1033 .248446 | 0 .258250885 | 0 . 10134 2 .54837 | 0 .01082 | 0 .01709 |
| CNR1 | 724 .0331327 | -0 .973283269 | 0 .27781 -3 .5035 | 0 .00046 | 0 .00091 |
| CNR2 | 89 .58995684 | 1 .671625416 | 0 .39592 4 .22215 | 2 .42E-05 | 5 .88E-05 |
| CNRIP1 | 2242 .957951 | -1 .799214595 | 0 . 18038 -9 .9743 | 1 .97E-23 | 4 .36E-22 |
| CNST | 6306 .23915 | -0 .595887046 | 0 .08551 -6 .969 | 3 . 19E-12 | 1 .98E-1 1 |
| CNTD1 | 72 .2047113 | -0 .834194192 | 0 . 16989 -4 .9103 | 9 .09E-07 | 2 .74E-06 |
| CNTD2 | 9 .392243796 | -0 .658709902 | 0 .29685 -2 .219 | 0 .02649 | 0 .03916 |
| CNTF | 214 .6826583 | 0 .01948437 | 0 .23107 0 .08432 | 0 .9328 | 0 .94417 |
| CNTFR | 84 .4709227 | 0 .930360064 | 0 .38951 2 .38855 | 0 .01692 | 0 .02584 |
| CNTLN | 2243 .766048 | -1 .213220033 | 0 . 1 1507 -10 .543 | 5 .47E-26 | 1 .61E-24 |
| CNTN1 | 13923 .05069 | -2 .927877779 | 0 .20041 -14 .609 | 2 .44E-48 | 6 .28E-46 |
| CNTN2 | 157 .4777611 | -1 .217623475 | 0 .24047 -5 .0635 | 4 . 12E-07 | 1 .30E-06 |
| CNTN3 | 1007 .659843 | -1 .432806306 | 0 .25443 -5 .6315 | 1 .79E-08 | 6 .89E-08 |
| CNTN4 | 1 155 .399862 | -2 .757929835 | 0 .27482 -10 .036 | 1 .06E-23 | 2 .42E-22 |
| CNTN5 | 52 .58521634 | -3 .933265243 | 0 .47083 -8 .3539 | 6 .60E-17 | 7 . 10E-16 |
| CNTN6 | 101 .703385 | -1 .342905983 | 0 .41329 -3 .2493 | 0 .00116 | 0 .00216 |
| CNTNAP1 | 545 .3895761 | 0 .979311327 | 0 . 13895 7 .04815 | 1 .81E-12 | 1 . 17E-1 1 |
| CNTNAP2 | 120 .0299548 | 0 .407966004 | 0 .35114 1 . 16183 | 0 .2453 | 0 .29575 |
| CNTNAP3 | 1341 .868108 | -1 .864687872 | 0 .2904 -6 .4212 | 1 .35E-10 | 6 .83E-10 |
| CNTNAP3B | 974 . 1627546 | -2 .018903291 | 0 .2556 -7 .8985 | 2 .82E-15 | 2 .50E-14 |
| CNTNAP4 | 41 .06850171 | -1 .375999727 | 0 .34737 -3 .9612 | 7 .46E-05 | 0 .00017 |
| CNTNAP5 | 22 .30322185 | -2 .370224661 | 0 .58274 -4 .0674 | 4 .75E-05 | 0 .00011 |
| CNTRL | 3487 .446085 | -0 .394293649 | 0 . 1 1547 -3 .4147 | 0 .00064 | 0 .00124 |
| CNTROB | 1321 .008756 | 0 . 177443232 | 0 .09655 1 .83781 | 0 .06609 | 0 .09028 |
| COA1 | 3732 .345673 | -0 .049584442 | 0 .07022 -0 .7061 | 0 .48014 | 0 .53537 |
| COA3 | 2252 .801213 | -0 .482735472 | 0 . 14567 -3 .3138 | 0 .00092 | 0 .00175 |
| COA4 | 2343 .240728 | -0 .210719225 | 0 . 16595 -1 .2698 | 0 .20417 | 0 .25117 |
| COA5 | 2833 .871642 | -0 .484497936 | 0 .097 -4 .9946 | 5 .90E-07 | 1 .82E-06 |
| COA6 | 1002 .85275 | 0 . 193148784 | 0 . 15701 1 .23019 | 0 .21863 | 0 .26674 |
| COA7 | 989 .7204168 | 0 .445754733 | 0 . 1 1684 3 .81506 | 0 .00014 | 0 .00029 |
| COASY | 1365 . 160665 | 0 .358185303 | 0 .08517 4 .20547 | 2 .61E-05 | 6 .30E-05 |
| COBL | 1866 .38256 | -1 .089422056 | 0 .21636 -5 .0351 | 4 .78E-07 | 1 .50E-06 |
| COBLL1 | 4961 .758672 | -0 .965917556 | 0 . 1674 -5 .7701 | 7 .92E-09 | 3 .21E-08 |
| COCH | 320 .9359449 | -0 .080855468 | 0 .30627 -0 .264 | 0 .79178 | 0 .82237 |
| COG1 | 1340 .479482 | -0 . 142397033 | 0 .07855 -1 .8128 | 0 .06987 | 0 .09497 |
| COG2 | 3178 .255377 | -0 .088931956 | 0 .07103 -1 .2521 | 0 .21053 | 0 .25799 |

| COG3 | 4677 .459306 | -0 . 127403287 | 0 .08825 | -1 .4437 | 0 . 14883 | 0 . 18884 |
| --- | --- | --- | --- | --- | --- | --- |
| COG4 | 4781 .888048 | 0 .019257554 | 0 .09276 | 0 .20761 | 0 .83553 | 0 .86076 |
| COG5 | 5225 .703445 | -0 .228769844 | 0 .07482 | -3 .0577 | 0 .00223 | 0 .00398 |
| COG6 | 3522 .948056 | -0 .230532425 | 0 . 10035 | -2 .2973 | 0 .0216 | 0 .03242 |
| COG7 | 1007 . 186926 | 0 .076893083 | 0 .06604 | 1 . 16439 | 0 .24427 | 0 .2947 |
| COG8 | 1 159 .231049 | 0 .476534587 | 0 .08866 | 5 .37503 | 7 .66E-08 | 2 .71E-07 |
| COIL | 1463 . 143182 | -0 .276889448 | 0 .0716 | -3 .8673 | 0 .00011 | 0 .00024 |
| COL10A1 | 905 .7818446 | -0 . 188314112 | 0 .22994 | -0 .819 | 0 .4128 | 0 .46934 |
| COL11A1 | 3048 .239995 | 1 .888956312 | 0 .41379 | 4 .565 | 5 .00E-06 | 1 .34E-05 |
| COL11A2 | 1780 .473091 | -1 .357233622 | 0 .42834 | -3 . 1686 | 0 .00153 | 0 .0028 |
| COL12A1 | 20921 .23396 | 1 .537965144 | 0 .20423 | 7 .53066 | 5 .05E-14 | 3 .86E-13 |
| COL13A1 | 424 .8122169 | 1 .099076675 | 0 . 19163 | 5 .73536 | 9 .73E-09 | 3 .88E-08 |
| COL14A1 | 1 1221 .44281 | -0 .370409622 | 0 .23179 | -1 .598 | 0 . 1 1004 | 0 . 14375 |
| COL15A1 | 1 1901 .94924 | 1 .912034931 | 0 . 16255 | 1 1 .7624 | 6 .09E-32 | 3 .26E-30 |
| COL16A1 | 8137 .958405 | 0 . 106582839 | 0 . 13474 | 0 .79105 | 0 .42891 | 0 .48548 |
| COL17A1 | 36892 .84538 | -0 .788535697 | 0 .28897 | -2 .7288 | 0 .00636 | 0 .0105 |
| COL18A1 | 8733 .439296 | 2 .226587235 | 0 . 14103 | 15 .7881 | 3 .76E-56 | 2 . 12E-53 |
| COL19A1 | 948 .9455538 | 0 .785577034 | 0 .45906 | 1 .71128 | 0 .08703 | 0 . 1 1619 |
| COL1A1 | 68881 .40922 | 3 .542760573 | 0 .22874 | 15 .4884 | 4 . 15E-54 | 1 .69E-51 |
| COL1A2 | 122656 . 1952 | 1 .696069166 | 0 . 18337 | 9 .24922 | 2 .26E-20 | 3 .63E-19 |
| COL20A1 | 35 .50747249 | -0 .809989129 | 0 .47633 | -1 .7005 | 0 .08904 | 0 . 1 1869 |
| COL21A1 | 1572 .851284 | -1 .744061943 | 0 .26002 | -6 .7075 | 1 .98E-1 1 | 1 . 12E-10 |
| COL22A1 | 1054 .682649 | 0 . 171772207 | 0 .4126 | 0 .41631 | 0 .67718 | 0 .72081 |
| COL23A1 | 265 .7863102 | 0 .524506824 | 0 . 17851 | 2 .93828 | 0 .0033 | 0 .00573 |
| COL24A1 | 2275 .330497 | -2 .658564665 | 0 .28379 | -9 .368 | 7 .39E-21 | 1 .24E-19 |
| COL25A1 | 673 .7253262 | -1 .68327978 | 0 .26743 | -6 .2942 | 3 .09E-10 | 1 .49E-09 |
| COL27A1 | 2963 .860854 | -1 .911873174 | 0 .20721 | -9 .2266 | 2 .79E-20 | 4 .42E-19 |
| COL28A1 | 627 .2460212 | -1 .617183737 | 0 .28639 | -5 .6468 | 1 .63E-08 | 6 .35E-08 |
| COL2A1 | 75 .20798034 | 0 .226995085 | 0 .32529 | 0 .69782 | 0 .48529 | 0 .54039 |
| COL3A1 | 211352 .0837 | 1 .254368654 | 0 .20449 | 6 . 13406 | 8 .57E-10 | 3 .93E-09 |
| COL4A1 | 20489 .63724 | 2 .799966869 | 0 . 18912 | 14 .8053 | 1 .35E-49 | 3 .94E-47 |
| COL4A2 | 15874 . 19421 | 2 .517655794 | 0 . 17911 | 14 .0562 | 7 .06E-45 | 1 .35E-42 |
| COL4A3 | 425 .2622657 | -0 .271379367 | 0 . 18147 | -1 .4955 | 0 . 1348 | 0 . 17266 |
| COL4A3BP | 6821 .268674 | -0 .214056684 | 0 .08645 | -2 .4762 | 0 .01328 | 0 .02065 |
| COL4A4 | 1247 .450349 | 1 . 19032607 | 0 .23105 | 5 . 1518 | 2 .58E-07 | 8 .41E-07 |
| COL4A5 | 2981 .781507 | -0 .82434175 | 0 .2635 | -3 . 1284 | 0 .00176 | 0 .00318 |
| COL4A6 | 803 . 1510471 | -0 .898874262 | 0 .24743 | -3 .6328 | 0 .00028 | 0 .00058 |
| COL5A1 | 9977 .760631 | 2 .40392296 | 0 . 1673 | 14 .3686 | 8 . 15E-47 | 1 .82E-44 |
| COL5A2 | 23648 .66398 | 1 .551930551 | 0 . 18442 | 8 .41503 | 3 .93E-17 | 4 .32E-16 |
| COL5A3 | 3131 .838708 | 1 .538460358 | 0 . 14203 | 10 .8321 | 2 .43E-27 | 8 . 19E-26 |
| COL6A1 | 22757 .72343 | 2 .060285735 | 0 . 15927 | 12 .9355 | 2 .84E-38 | 2 .79E-36 |
| COL6A2 | 30462 . 14768 | 1 .691631712 | 0 . 14846 | 1 1 .3948 | 4 .44E-30 | 1 .92E-28 |
| COL6A3 | 71199 .95251 | 1 .695130174 | 0 . 16801 | 10 .0893 | 6 . 16E-24 | 1 .44E-22 |
| COL6A5 | 2307 .812056 | 0 .382584847 | 0 .34286 | 1 . 1 1586 | 0 .26448 | 0 .31609 |
| COL6A6 | 2420 .425799 | 1 .093101862 | 0 .29195 | 3 .74409 | 0 .00018 | 0 .00038 |
| COL7A1 | 7636 .813898 | 0 .0311 1647 | 0 . 18001 | 0 . 17286 | 0 .86276 | 0 .88438 |
| COL8A1 | 951 .0435914 | 1 .004582325 | 0 .27662 | 3 .63164 | 0 .00028 | 0 .00058 |
| COL8A2 | 1946 .981515 | 0 .671097166 | 0 . 15377 | 4 .36419 | 1 .28E-05 | 3 .23E-05 |
| COL9A1 | 323 .220688 | -0 .350682607 | 0 .55704 | -0 .6295 | 0 .52899 | 0 .58285 |
| COL9A2 | 355 .6787654 | 0 .560762569 | 0 . 12252 | 4 .57676 | 4 .72E-06 | 1 .27E-05 |
| COL9A3 | 1007 .845471 | 0 .374323597 | 0 .30936 | 1 .21 | 0 .22628 | 0 .27522 |
| COLCA1 | 92 .57406329 | -1 .388471092 | 0 .217 | -6 .3986 | 1 .57E-10 | 7 .87E-10 |
| COLCA2 | 29 .70088788 | -0 .803824167 | 0 .25205 | -3 . 1891 | 0 .00143 | 0 .00263 |
| COLEC10 | 8 .969901707 | -2 .26180459 | 0 .43489 | -5 .2009 | 1 .98E-07 | 6 .56E-07 |
| COLEC11 | 183 .7689229 | -1 . 197900092 | 0 .3211 1 | -3 .7305 | 0 .00019 | 0 .0004 |
| COLEC12 | 2650 .979663 | 0 .666838408 | 0 . 17201 | 3 .8767 | 0 .00011 | 0 .00023 |

| COLGALT1 | 4919 .634455 | 1 . 159309621 | 0 .08914 | 13 .0048 | 1 . 15E-38 | 1 . 15E-36 |
| --- | --- | --- | --- | --- | --- | --- |
| COLGALT2 | 1395 . 1 18297 | -0 .793540832 | 0 .2273 | -3 .4911 | 0 .00048 | 0 .00095 |
| COLQ | 201 .0299341 | 0 .020499764 | 0 . 17473 | 0 . 1 1732 | 0 .90661 | 0 .92238 |
| COMMD1 | 1304 .46238 | -0 .386243052 | 0 .09561 | -4 .0397 | 5 .35E-05 | 0 .00012 |
| COMMD10 | 2294 .325528 | -0 .364230341 | 0 . 13676 | -2 .6632 | 0 .00774 | 0 .01258 |
| COMMD2 | 3698 .817284 | -0 . 1 19374443 | 0 .08531 | -1 .3993 | 0 . 16173 | 0 .20369 |
| COMMD3 | 1309 .338196 | -0 .572033285 | 0 . 1201 | -4 .7631 | 1 .91E-06 | 5 .43E-06 |
| COMMD4 | 2301 .415497 | -0 .002797159 | 0 . 13254 | -0 .0211 | 0 .98316 | 0 .98563 |
| COMMD5 | 1340 .576708 | 0 .682460687 | 0 . 12689 | 5 .3783 | 7 .52E-08 | 2 .66E-07 |
| COMMD6 | 6088 . 108742 | -1 .421218806 | 0 . 159 | -8 .9384 | 3 .95E-19 | 5 .46E-18 |
| COMMD7 | 1575 .253023 | 0 .201447525 | 0 .0942 | 2 . 13853 | 0 .03247 | 0 .0471 |
| COMMD8 | 2060 .054307 | -0 .49199017 | 0 . 14623 | -3 .3644 | 0 .00077 | 0 .00147 |
| COMMD9 | 2245 . 147401 | -0 .303910638 | 0 .095 | -3 . 199 | 0 .00138 | 0 .00254 |
| COMP | 2742 .963358 | 1 .805978363 | 0 .36152 | 4 .99557 | 5 .87E-07 | 1 .81E-06 |
| COMT | 5002 .998789 | -0 .271822991 | 0 . 1 199 | -2 .2671 | 0 .02338 | 0 .0349 |
| COMTD1 | 645 .2628277 | 1 . 151789803 | 0 . 16454 | 7 .00025 | 2 .56E-12 | 1 .61E-1 1 |
| COPA | 13295 .66846 | 0 .689755366 | 0 . 10085 | 6 .83917 | 7 .97E-12 | 4 .69E-1 1 |
| COPB1 | 13734 .84885 | 0 . 151346096 | 0 . 12294 | 1 .23108 | 0 .21829 | 0 .26638 |
| COPB2 | 9065 .919839 | 0 . 175409525 | 0 .08553 | 2 .05078 | 0 .04029 | 0 .0574 |
| COPE | 3106 .513063 | 0 .342069682 | 0 . 10602 | 3 .22656 | 0 .00125 | 0 .00233 |
| COPG1 | 8515 .394708 | 0 .426995561 | 0 . 10336 | 4 . 13101 | 3 .61E-05 | 8 .53E-05 |
| COPG2 | 1503 . 138023 | -0 .07741892 | 0 . 1245 | -0 .6218 | 0 .53404 | 0 .58765 |
| COPRS | 2303 .216454 | 0 . 106164389 | 0 . 15938 | 0 .6661 | 0 .50535 | 0 .56003 |
| COPS2 | 10638 . 17886 | -0 .72325248 | 0 . 10587 | -6 .8312 | 8 .42E-12 | 4 .94E-1 1 |
| COPS3 | 3997 .633106 | -0 .058966676 | 0 . 10557 | -0 .5586 | 0 .57646 | 0 .62806 |
| COPS4 | 3613 .799452 | -0 .403745177 | 0 . 10505 | -3 .8432 | 0 .00012 | 0 .00026 |
| COPS5 | 4184 .386576 | -0 . 192033292 | 0 .0881 | -2 . 1798 | 0 .02928 | 0 .04286 |
| COPS6 | 2338 .216086 | 0 .089981495 | 0 . 10869 | 0 .82787 | 0 .40775 | 0 .46431 |
| COPS7A | 3250 . 124175 | 0 . 152204203 | 0 .09017 | 1 .68789 | 0 .09143 | 0 . 12158 |
| COPS7B | 888 .6393316 | -0 . 174479153 | 0 .08761 | -1 .9915 | 0 .04643 | 0 .06542 |
| COPS8 | 5373 .439339 | 0 .234031949 | 0 . 1 1769 | 1 .98855 | 0 .04675 | 0 .06584 |
| COPS9 | 2248 .793003 | -0 .350663043 | 0 . 16564 | -2 . 1 17 | 0 .03426 | 0 .04945 |
| COPZ1 | 7144 .88823 | 0 . 16719927 | 0 . 10216 | 1 .6367 | 0 . 10169 | 0 . 13372 |
| COPZ2 | 794 .6796292 | 0 .016803488 | 0 . 1 1303 | 0 . 14867 | 0 .88181 | 0 .90083 |
| COQ10A | 462 .4476541 | -0 .290992029 | 0 . 1 1893 | -2 .4468 | 0 .01441 | 0 .02226 |
| COQ10B | 3047 .245306 | 0 .518090546 | 0 . 10179 | 5 .09 | 3 .58E-07 | 1 . 14E-06 |
| COQ2 | 871 .6497526 | 0 .013608064 | 0 . 10089 | 0 . 13488 | 0 .89271 | 0 .91031 |
| COQ3 | 454 .0075827 | -0 .437680894 | 0 . 1046 | -4 . 1843 | 2 .86E-05 | 6 .87E-05 |
| COQ4 | 2206 .328919 | 0 .325833174 | 0 .08262 | 3 .94384 | 8 .02E-05 | 0 .00018 |
| COQ5 | 1496 .662063 | -0 .504981459 | 0 .09531 | -5 .2982 | 1 . 17E-07 | 4 .02E-07 |
| COQ6 | 404 .8400598 | -0 .257324596 | 0 .06513 | -3 .9507 | 7 .79E-05 | 0 .00017 |
| COQ7 | 1358 .35329 | -0 . 137177181 | 0 .07952 | -1 .7251 | 0 .08451 | 0 . 1 1312 |
| COQ9 | 1861 .32411 | 0 . 108425359 | 0 .08969 | 1 .20891 | 0 .2267 | 0 .27562 |
| CORIN | 909 .319664 | 0 .926832936 | 0 .30814 | 3 .00782 | 0 .00263 | 0 .00463 |
| CORO1A | 1663 .50234 | 1 .844507651 | 0 . 18687 | 9 .8704 | 5 .59E-23 | 1 . 18E-21 |
| CORO1B | 4038 .962416 | 0 .688537547 | 0 . 10016 | 6 .87427 | 6 .23E-12 | 3 .73E-1 1 |
| CORO1C | 1 1412 .06647 | 0 .402835801 | 0 .09318 | 4 .32336 | 1 .54E-05 | 3 .85E-05 |
| CORO2A | 1026 .385996 | -0 .236592263 | 0 . 15304 | -1 .546 | 0 . 12212 | 0 . 15789 |
| CORO2B | 2117 .257339 | -1 .417013671 | 0 .26197 | -5 .409 | 6 .34E-08 | 2 .27E-07 |
| CORO6 | 155 .0258619 | 0 .074632797 | 0 . 18536 | 0 .40263 | 0 .68722 | 0 .73008 |
| CORO7 | 380 .7581806 | 1 .027637975 | 0 . 10063 | 10 .2125 | 1 .74E-24 | 4 .33E-23 |
| CORT | 10 .78644809 | 0 .238716933 | 0 .24351 | 0 .98032 | 0 .32693 | 0 .38185 |
| COTL1 | 3823 .686202 | 2 . 151312102 | 0 . 13586 | 15 .835 | 1 .79E-56 | 1 .07E-53 |
| COX10 | 884 .8679618 | 0 . 138854736 | 0 .0704 | 1 .97243 | 0 .04856 | 0 .06813 |
| COX11 | 3551 .957805 | -0 .401822336 | 0 .08278 | -4 .8538 | 1 .21E-06 | 3 .56E-06 |
| COX14 | 1510 .920764 | 0 .031397656 | 0 . 14707 | 0 .21349 | 0 .83095 | 0 .85713 |

| COX15 | 2988 .2405 -0 .347975261 | 0 .05783 -6 .0173 | 1 .77E-09 | 7 .79E-09 |
| --- | --- | --- | --- | --- |
| COX16 | 1467 .534125 -0 .297760786 | 0 . 12053 -2 .4705 | 0 .01349 | 0 .02094 |
| COX17 | 1075 .004035 -0 .399962984 | 0 . 176 -2 .2725 | 0 .02306 | 0 .03444 |
| COX18 | 2184 .620455 0 .354149335 | 0 .09212 3 .84426 | 0 .00012 | 0 .00026 |
| COX19 | 988 .4090511 -0 . 164630328 | 0 .06394 -2 .5749 | 0 .01003 | 0 .01595 |
| COX20 | 365 . 1660933 -0 .960890489 | 0 . 12712 -7 .5592 | 4 .06E-14 | 3 . 14E-13 |
| COX4I1 | 14918 .4378 -0 .609934985 | 0 . 14731 -4 . 1405 | 3 .47E-05 | 8 .22E-05 |
| COX4I2 | 79 . 10215204 1 . 121697616 | 0 . 19739 5 .6827 | 1 .33E-08 | 5 .20E-08 |
| COX5A | 2638 .097943 1 .477681142 | 0 . 14665 10 .076 | 7 .05E-24 | 1 .64E-22 |
| COX5B | 1 1628 . 10692 -0 .257514156 | 0 . 16368 -1 .5733 | 0 . 1 1566 | 0 . 15042 |
| COX6A1 | 3583 .827236 0 .496869418 | 0 . 16484 3 .01433 | 0 .00258 | 0 .00455 |
| COX6A1P2 | 100 .6441916 1 .618288842 | 0 .29076 5 .56567 | 2 .61E-08 | 9 .86E-08 |
| COX6A2 | 6 .615716969 -1 .210139993 | 0 .29743 -4 .0687 | 4 .73E-05 | 0 .00011 |
| COX6B1 | 8791 .281743 -0 .047551765 | 0 . 16186 -0 .2938 | 0 .76893 | 0 .80194 |
| COX6B2 | 20 .52389794 -0 .919830417 | 0 .22462 -4 .0951 | 4 .22E-05 | 9 .87E-05 |
| COX6C | 10271 .89725 -0 . 135273471 | 0 . 15583 -0 .8681 | 0 .38533 | 0 .4417 |
| COX7A1 | 668 .8577189 0 .341739013 | 0 .26691 1 .28037 | 0 .20041 | 0 .24716 |
| COX7A2 | 6652 .746088 -0 .207986439 | 0 . 18393 -1 . 1308 | 0 .25814 | 0 .30944 |
| COX7A2L | 8564 .861876 -0 .92218461 | 0 . 14174 -6 .5062 | 7 .71E-1 1 | 4 .02E-10 |
| COX7B | 7088 .835446 -0 .248798151 | 0 . 14227 -1 .7488 | 0 .08033 | 0 . 10793 |
| COX7B2 | 4 .497458866 -0 .8153296 | 1 .23336 -0 .6611 | 0 .50857 | 0 .56302 |
| COX7C | 13836 .96034 -0 .727659628 | 0 . 16893 -4 .3074 | 1 .65E-05 | 4 . 12E-05 |
| COX8A | 4832 .69978 -0 .065569948 | 0 . 16834 -0 .3895 | 0 .6969 | 0 .73884 |
| COX8C | 3 .325672961 -0 .952472633 | 0 .46418 -2 .0519 | 0 .04018 | 0 .05726 |
| CP | 1849 .304346 1 .052947262 | 0 .23779 4 .42815 | 9 .50E-06 | 2 .45E-05 |
| CPA1 | 6 .518107215 -1 .257799021 | 0 .39454 -3 . 188 | 0 .00143 | 0 .00263 |
| CPA2 | 39 .57005405 1 . 123562201 | 0 .39215 2 .86515 | 0 .00417 | 0 .00711 |
| CPA3 | 2850 .507953 -0 .552438652 | 0 .28122 -1 .9644 | 0 .04948 | 0 .06933 |
| CPA4 | 3396 .605421 -0 .48447412 | 0 .27325 -1 .773 | 0 .07623 | 0 . 10298 |
| CPA5 | 21 .80426251 -1 .085337541 | 0 .27219 -3 .9874 | 6 .68E-05 | 0 .00015 |
| CPA6 | 16 .48550814 -0 .552412206 | 0 .38565 -1 .4324 | 0 . 15203 | 0 . 1925 |
| CPAMD8 | 123 .8153851 0 . 18417902 | 0 . 1933 0 .95283 | 0 .34067 | 0 .39609 |
| CPB1 | 56 .05482876 -0 .45625427 | 0 .38998 -1 . 1699 | 0 .24203 | 0 .29242 |
| CPB2 | 187 .3009986 -0 .353689255 | 0 .25318 -1 .397 | 0 . 16242 | 0 .20451 |
| CPD | 10187 .83809 0 .352524935 | 0 . 10232 3 .44549 | 0 .00057 | 0 .00112 |
| CPE | 4081 .080112 0 .442711838 | 0 .20074 2 .20536 | 0 .02743 | 0 .0404 |
| CPEB1 | 444 .2989688 -1 .210781516 | 0 .20174 -6 .0018 | 1 .95E-09 | 8 .53E-09 |
| CPEB2 | 12465 .51709 -0 . 101260639 | 0 . 14256 -0 .7103 | 0 .47753 | 0 .53299 |
| CPEB3 | 1015 .302006 -0 . 191030615 | 0 . 15147 -1 .2612 | 0 .20724 | 0 .25446 |
| CPEB4 | 9052 .848784 0 . 171672501 | 0 .0979 1 .75353 | 0 .07951 | 0 . 10695 |
| CPED1 | 2674 .537721 -1 .229347044 | 0 . 19996 -6 . 1479 | 7 .85E-10 | 3 .62E-09 |
| CPLX1 | 29 .20009109 0 .95599249 | 0 .25659 3 .72579 | 0 .00019 | 0 .00041 |
| CPLX2 | 18 .2479673 -1 .901873341 | 0 .35725 -5 .3237 | 1 .02E-07 | 3 .53E-07 |
| CPLX4 | 7 .655153387 -4 .597615782 | 0 .69537 -6 .6117 | 3 .80E-1 1 | 2 .07E-10 |
| CPM | 4522 .023788 -0 .004884333 | 0 . 15904 -0 .0307 | 0 .9755 | 0 .97912 |
| CPN1 | 372 .7854794 0 .650229783 | 0 .43309 1 .50137 | 0 . 13326 | 0 . 17083 |
| CPN2 | 24 .72563881 1 .434520252 | 0 .56131 2 .55566 | 0 .0106 | 0 .01677 |
| CPNE1 | 2571 .96845 0 .429637701 | 0 . 1 1074 3 .87962 | 0 .0001 | 0 .00023 |
| CPNE2 | 1294 .731499 0 .317995862 | 0 . 103 3 .08731 | 0 .00202 | 0 .00363 |
| CPNE3 | 14737 .30751 -0 .236747 | 0 . 1067 -2 .2188 | 0 .0265 | 0 .03917 |
| CPNE4 | 53 .28842213 -2 .097772097 | 0 .26039 -8 .0562 | 7 .87E-16 | 7 .37E-15 |
| CPNE5 | 337 .8761338 -0 .726723888 | 0 .28449 -2 .5545 | 0 .01063 | 0 .01682 |
| CPNE6 | 28 .00292541 -0 .235675063 | 0 .31265 -0 .7538 | 0 .45097 | 0 .50688 |
| CPNE7 | 220 .2823574 2 .463060695 | 0 .29738 8 .28263 | 1 .20E-16 | 1 .26E-15 |
| CPNE8 | 2056 .959633 -0 .704588647 | 0 . 1 1615 -6 .066 | 1 .31E-09 | 5 .87E-09 |
| CPNE9 | 20 .60574079 -0 .946086327 | 0 .25324 -3 .7359 | 0 .00019 | 0 .00039 |

| CPO | 22 .95287332 | -1 .538865813 | 0 .29252 | -5 .2608 | 1 .43E-07 | 4 .86E-07 |
| --- | --- | --- | --- | --- | --- | --- |
| CPOX | 2338 .03165 | 0 .096705506 | 0 . 12838 | 0 .75328 | 0 .45128 | 0 .50714 |
| CPPED1 | 3114 .708044 | 0 .006470938 | 0 . 10917 | 0 .05927 | 0 .95274 | 0 .96014 |
| CPQ | 7508 .750112 | -0 .41438697 | 0 . 12991 | -3 . 1899 | 0 .00142 | 0 .00262 |
| CPS1 | 1609 .807448 | -0 .734463342 | 0 .21686 | -3 .3868 | 0 .00071 | 0 .00137 |
| CPSF1 | 2407 .874556 | 0 .539880437 | 0 . 1 166 | 4 .63005 | 3 .66E-06 | 9 .99E-06 |
| CPSF2 | 6340 .424912 | 0 .301087708 | 0 .08949 | 3 .36446 | 0 .00077 | 0 .00147 |
| CPSF3 | 3050 . 1 12721 | 0 . 199830718 | 0 .09146 | 2 . 18478 | 0 .0289 | 0 .04237 |
| CPSF3L | 3138 .416868 | 0 . 175254669 | 0 .06757 | 2 .5938 | 0 .00949 | 0 .01516 |
| CPSF4 | 1490 .390241 | 0 .639981955 | 0 . 10701 | 5 .9804 | 2 .23E-09 | 9 .68E-09 |
| CPSF4L | 16 .28346367 | -0 .903187071 | 0 .24502 | -3 .6862 | 0 .00023 | 0 .00047 |
| CPSF6 | 10030 .0909 | -0 .460967077 | 0 . 10281 | -4 .4838 | 7 .33E-06 | 1 .92E-05 |
| CPSF7 | 2564 .071616 | 0 .025212321 | 0 .07699 | 0 .32746 | 0 .74332 | 0 .7797 |
| CPT1A | 3204 .236827 | 1 . 165066186 | 0 . 14057 | 8 .28815 | 1 . 15E-16 | 1 .21E-15 |
| CPT1B | 33 .86117723 | 0 . 149658791 | 0 . 18126 | 0 .82566 | 0 .409 | 0 .4656 |
| CPT1C | 241 .9116986 | 0 .478311384 | 0 . 13398 | 3 .57009 | 0 .00036 | 0 .00072 |
| CPT2 | 881 .0101134 | 0 .63756095 | 0 .09834 | 6 .48355 | 8 .96E-1 1 | 4 .63E-10 |
| CPTP | 904 .8505665 | 0 .636039877 | 0 . 13365 | 4 .7589 | 1 .95E-06 | 5 .54E-06 |
| CPVL | 5641 .824113 | 0 .692262035 | 0 . 17898 | 3 .86788 | 0 .00011 | 0 .00024 |
| CPXCR1 | 4 .223680326 | -4 .295876112 | 0 .93368 | -4 .601 | 4 .20E-06 | 1 . 14E-05 |
| CPXM1 | 1384 .667037 | 3 .569440772 | 0 .28635 | 12 .4653 | 1 . 15E-35 | 8 .89E-34 |
| CPXM2 | 1466 .675486 | -0 .48601377 | 0 .21281 | -2 .2838 | 0 .02238 | 0 .03352 |
| CPZ | 28 .90652209 | 0 .883558817 | 0 .2461 | 3 .59018 | 0 .00033 | 0 .00067 |
| CR1 | 390 .3514552 | 2 .277276899 | 0 .25513 | 8 .92581 | 4 .42E-19 | 6 . 10E-18 |
| CR1L | 18 .07631176 | -1 .235132595 | 0 .3862 | -3 . 1982 | 0 .00138 | 0 .00255 |
| CR2 | 40 .28863737 | 0 . 108666646 | 0 .55465 | 0 . 19592 | 0 .84467 | 0 .869 |
| CRABP1 | 59 .52978068 | 1 . 122060361 | 0 .28367 | 3 .95552 | 7 .64E-05 | 0 .00017 |
| CRABP2 | 2164 .533356 | -0 .019148961 | 0 .24024 | -0 .0797 | 0 .93647 | 0 .94683 |
| CRACR2A | 554 .0688202 | 1 .725115463 | 0 . 17557 | 9 .826 | 8 .70E-23 | 1 .79E-21 |
| CRACR2B | 194 . 1084822 | 1 .442295227 | 0 .22997 | 6 .27164 | 3 .57E-10 | 1 .72E-09 |
| CRADD | 922 .9311624 | -0 .553520216 | 0 . 10495 | -5 .2742 | 1 .33E-07 | 4 .55E-07 |
| CRAMP1 | 899 .9861842 | 0 . 152613452 | 0 .09171 | 1 .66405 | 0 .0961 | 0 . 12708 |
| CRAT | 3560 .37456 | 1 .933203196 | 0 .39029 | 4 .95324 | 7 .30E-07 | 2 .22E-06 |
| CRB1 | 100 . 136859 | -0 .922176889 | 0 .34376 | -2 .6826 | 0 .00731 | 0 .01193 |
| CRB2 | 182 .9595202 | 0 .414581615 | 0 .25917 | 1 .59963 | 0 . 10968 | 0 . 14332 |
| CRB3 | 472 .7425453 | -0 .892480186 | 0 .25164 | -3 .5467 | 0 .00039 | 0 .00078 |
| CRBN | 4629 .791348 | -0 .901229758 | 0 .08453 | -10 .661 | 1 .55E-26 | 4 .76E-25 |
| CRCP | 2664 .651328 | 0 .358303089 | 0 .07936 | 4 .51466 | 6 .34E-06 | 1 .68E-05 |
| CRCT1 | 1487 .447765 | -0 .430526951 | 0 .30343 | -1 .4189 | 0 . 15593 | 0 . 19707 |
| CREB1 | 6639 .684298 | -0 .224890072 | 0 .06595 | -3 .4098 | 0 .00065 | 0 .00126 |
| CREB3 | 1899 .54071 | -0 .371299427 | 0 . 10642 | -3 .4891 | 0 .00048 | 0 .00096 |
| CREB3L1 | 157 .3238849 | 2 .271897313 | 0 .22974 | 9 .88882 | 4 .65E-23 | 9 .89E-22 |
| CREB3L2 | 5898 . 124658 | 0 .820991527 | 0 .09972 | 8 .23256 | 1 .83E-16 | 1 .86E-15 |
| CREB3L3 | 5 .365990895 | -3 . 182731213 | 0 .63377 | -5 .0219 | 5 . 12E-07 | 1 .59E-06 |
| CREB3L4 | 851 .9614413 | -0 .258562374 | 0 . 13635 | -1 .8963 | 0 .05793 | 0 .08001 |
| CREB5 | 2071 .862277 | -0 .024284838 | 0 .20549 | -0 . 1 182 | 0 .90592 | 0 .92173 |
| CREBBP | 4974 .927491 | 0 .214937425 | 0 . 1 1845 | 1 .81456 | 0 .06959 | 0 .09461 |
| CREBL2 | 9431 . 187652 | -0 .306313934 | 0 .09184 | -3 .3354 | 0 .00085 | 0 .00162 |
| CREBRF | 8055 .085578 | -0 .590674951 | 0 . 1039 | -5 .6851 | 1 .31E-08 | 5 . 14E-08 |
| CREBZF | 6808 .055813 | -0 .586826316 | 0 . 1 1043 | -5 .3139 | 1 .07E-07 | 3 .71E-07 |
| CREG1 | 12349 .71401 | 0 .019814316 | 0 . 18105 | 0 . 10944 | 0 .91285 | 0 .92765 |
| CREG2 | 48 .25955313 | -0 .228824723 | 0 .37856 | -0 .6045 | 0 .54553 | 0 .59841 |
| CRELD1 | 981 .2043688 | 0 .077520646 | 0 . 1 1742 | 0 .66019 | 0 .50913 | 0 .56357 |
| CRELD2 | 1630 .87957 | 1 .019696678 | 0 . 1 1446 | 8 .90901 | 5 . 15E-19 | 7 .06E-18 |
| CREM | 1589 .500556 | 0 .436923831 | 0 . 15579 | 2 .80458 | 0 .00504 | 0 .00847 |
| CRH | 3 . 136488666 | -3 . 121369255 | 0 .86031 | -3 .6282 | 0 .00029 | 0 .00059 |

| CRHBP | 87 .26845817 -0 .062380275 | 0 .2589 -0 .2409 | 0 .8096 | 0 .83818 |
| --- | --- | --- | --- | --- |
| CRHR1 | 19 .64304296 -0 .22318735 | 0 .34946 -0 .6387 | 0 .52304 | 0 .57723 |
| CRHR2 | 28 .53151071 -0 .037186282 | 0 .2196 -0 . 1693 | 0 .86553 | 0 .88679 |
| CRIM1 | 6760 .599501 0 .032863434 | 0 . 153 0 .2148 | 0 .82992 | 0 .8563 |
| CRIP1 | 178 .7167406 -0 . 1 12936929 | 0 .21337 -0 .5293 | 0 .59659 | 0 .64712 |
| CRIP2 | 3098 .237874 -0 .75410808 | 0 . 14711 -5 . 1263 | 2 .96E-07 | 9 .55E-07 |
| CRIP3 | 24 .63658579 -0 .889541215 | 0 .25241 -3 .5241 | 0 .00042 | 0 .00085 |
| CRIPAK | 495 .7069761 0 .426860225 | 0 . 16214 2 .63267 | 0 .00847 | 0 .01366 |
| CRIPT | 1578 .7391 -0 . 150135154 | 0 . 10281 -1 .4603 | 0 . 14421 | 0 . 18347 |
| CRISP1 | 8 .003929164 -5 .003996594 | 0 .79941 -6 .2596 | 3 .86E-10 | 1 .85E-09 |
| CRISP2 | 15 .49688409 -1 . 1 10134616 | 0 .44416 -2 .4994 | 0 .01244 | 0 .01944 |
| CRISP3 | 130 .3858761 1 .743738502 | 0 .39879 4 .37255 | 1 .23E-05 | 3 . 12E-05 |
| CRISPLD1 | 2921 .228478 -0 .777659921 | 0 .20485 -3 .7963 | 0 .00015 | 0 .00032 |
| CRISPLD2 | 3199 . 14796 1 .099624772 | 0 . 17632 6 .23642 | 4 .48E-10 | 2 . 12E-09 |
| CRK | 6985 .619781 0 .587375467 | 0 .06603 8 .8951 | 5 .84E-19 | 7 .93E-18 |
| CRKL | 7416 .766825 0 . 199990312 | 0 .08114 2 .46462 | 0 .01372 | 0 .02127 |
| CRLF1 | 289 .8545065 0 .369715358 | 0 .2423 1 .52587 | 0 . 12704 | 0 . 1636 |
| CRLF2 | 24 .73478515 0 .09411 1251 | 0 .23838 0 .39479 | 0 .693 | 0 .73528 |
| CRLF3 | 2315 .355586 0 .047511506 | 0 .07852 0 .60512 | 0 .5451 | 0 .598 |
| CRLS1 | 3314 .939694 0 .291866609 | 0 . 12648 2 .30753 | 0 .02103 | 0 .03164 |
| CRMP1 | 632 .7007796 -0 . 163001974 | 0 . 16635 -0 .9798 | 0 .32716 | 0 .38207 |
| CRNKL1 | 4459 .721353 -0 .367626678 | 0 .09404 -3 .9092 | 9 .26E-05 | 0 .00021 |
| CRNN | 64 .80256982 -0 .815048342 | 0 .41722 -1 .9535 | 0 .05076 | 0 .07095 |
| CROCC | 1445 .736864 0 .098213657 | 0 . 131 0 .74973 | 0 .45341 | 0 .50927 |
| CROCC2 | 18 .82536969 -0 .222857287 | 0 .26778 -0 .8322 | 0 .40528 | 0 .4618 |
| CROT | 2293 .250356 -0 .265132919 | 0 . 1 1094 -2 .39 | 0 .01685 | 0 .02575 |
| CRP | 3 .734755016 -3 .379745764 | 0 .6935 -4 .8735 | 1 . 10E-06 | 3 .25E-06 |
| CRTAC1 | 7077 .339403 -2 .525685301 | 0 .33707 -7 .4931 | 6 .72E-14 | 5 .04E-13 |
| CRTAM | 199 .8161512 2 .228513597 | 0 .29957 7 .43903 | 1 .01E-13 | 7 .43E-13 |
| CRTAP | 15908 .48953 -0 .046434592 | 0 . 1 1289 -0 .4113 | 0 .68083 | 0 .72401 |
| CRTC1 | 514 . 1098464 0 .371282 | 0 . 13783 2 .69385 | 0 .00706 | 0 .01157 |
| CRTC2 | 478 .4136857 0 .383210649 | 0 .09677 3 .9601 | 7 .49E-05 | 0 .00017 |
| CRTC3 | 2977 .379399 0 .834827458 | 0 .07059 1 1 .8268 | 2 .84E-32 | 1 .58E-30 |
| CRX | 12 .88458973 -4 .921027078 | 0 .68033 -7 .2332 | 4 .72E-13 | 3 .23E-12 |
| CRY1 | 1515 .875344 0 . 141470234 | 0 .07475 1 .89267 | 0 .0584 | 0 .08063 |
| CRY2 | 2998 . 179089 -0 .715139969 | 0 . 1 168 -6 . 1227 | 9 .20E-10 | 4 .20E-09 |
| CRYAA | 2 .455290829 -1 . 120794394 | 0 .50824 -2 .2053 | 0 .02744 | 0 .0404 |
| CRYAB | 1388 .512986 -0 .720796303 | 0 .20692 -3 .4835 | 0 .00049 | 0 .00098 |
| CRYBA1 | 40 .91955451 -1 .086413134 | 0 . 14443 -7 .5223 | 5 .38E-14 | 4 . 10E-13 |
| CRYBA2 | 4 .813232783 -1 . 1 1 1205725 | 0 .53523 -2 .0761 | 0 .03788 | 0 .05421 |
| CRYBA4 | 3 .770903528 -1 . 149959479 | 0 .52837 -2 . 1764 | 0 .02952 | 0 .0432 |
| CRYBB1 | 24 . 19614597 0 .677542902 | 0 .23771 2 .85029 | 0 .00437 | 0 .00742 |
| CRYBB2 | 4 .448197304 -1 .554337606 | 0 .4079 -3 .8106 | 0 .00014 | 0 .0003 |
| CRYBB3 | 12 .89047562 -1 .001910082 | 0 .2455 -4 .0811 | 4 .48E-05 | 0 .0001 |
| CRYBG3 | 2938 .61402 -0 .612727183 | 0 . 1 1321 -5 .4121 | 6 .23E-08 | 2 .23E-07 |
| CRYGA | 1 .058115775 -3 .672459816 | 0 .93419 -3 .9312 | 8 .45E-05 | 0 .00019 |
| CRYGB | 1 .454139616 -4 .319036961 | 0 .98445 -4 .3873 | 1 . 15E-05 | 2 .93E-05 |
| CRYGC | 1 .834627773 -3 .969592144 | 0 .99014 -4 .0091 | 6 .09E-05 | 0 .00014 |
| CRYGD | 1 .429603172 -1 .237029939 | 1 . 19584 -1 .0344 | 0 .30093 | 0 .35463 |
| CRYGN | 7 . 105855351 -1 .280537123 | 0 .3826 -3 .3469 | 0 .00082 | 0 .00156 |
| CRYGS | 240 .7201415 -0 .700480783 | 0 . 13916 -5 .0335 | 4 .82E-07 | 1 .51E-06 |
| CRYL1 | 4403 .085278 -1 .03993732 | 0 . 18176 -5 .7214 | 1 .06E-08 | 4 .20E-08 |
| CRYM | 1629 .807732 -3 .031331492 | 0 .27945 -10 .848 | 2 .05E-27 | 6 .98E-26 |
| CRYZ | 2474 .212361 -0 .42330599 | 0 . 12838 -3 .2974 | 0 .00098 | 0 .00184 |
| CRYZL1 | 1357 .07358 -0 .853642252 | 0 .08449 -10 . 103 | 5 .36E-24 | 1 .27E-22 |
| CS | 5390 .469579 0 . 180979838 | 0 . 10995 1 .64595 | 0 .09977 | 0 . 13135 |

| CSAD  CSAG1  CSDC2  CSDE1  CSE1L  CSF1  CSF1R  CSF2  CSF2RA  CSF2RB  CSF3  CSF3R | 877 .0083193 237 .4481548 41 . 17999673 41633 .55752 8440 .573596 981 .3698825 3035 .614893 17 .85833108 1 155 .627205 2215 .3617 21 .4350581 396 .0837695 |
| --- | --- |

CSGALNACT3365 .809404 CSGALNACT2651 .513952

| CSH1 CSH2 CSHL1 CSK CSMD1 CSMD2 CSMD3 CSN1S1 CSN2 CSN3 CSNK1A1 CSNK1A1L CSNK1D CSNK1E CSNK1G1 CSNK1G2 CSNK1G3 CSNK2A1 CSNK2A2 CSNK2A3 CSNK2B CSPG4 CSPG5 CSPP1 CSRNP1 CSRNP2 CSRNP3 CSRP1 CSRP2 CSRP3 CST1 CST11 CST2 CST3 CST4 CST5 CST6 CST7 CST8 CST9 CST9L CSTA | 0 .95416428 1 .396995361 1 .85698489 2022 .58657 313 .7241906 191 .333455 86 .47489715 10 .85853025 4 .318634665 3 .440322465 14691 .73935 1 1 .27379291 2250 .294728 3873 .756148 2453 .380765 2736 .438259 4092 .017513 8605 .085378 7610 .604142 31 .4537688 778 .013132 22028 .40997 206 .4059956 1998 .371662 1551 .657716 2136 .89471 1082 . 152378 17238 .66904 1663 .362153 10 .76628283 13 .64258786 2 .247761502 98 .96259959 17090 .02264 0 .765960471 4 . 181506936 10943 .7807 437 .7393859 2 .039046654 2 .803955674 1 . 199998207 17001 .93456 |
| --- | --- |

-0 .05100352

6 . 128928296

0 .406115212

-0 .391350549

0 . 1899811

1 .760578199

1 .054720246

0 .790363654

1 .653091003

1 .819911273

0 .926615094

2 .259179488

0 .439807433

-0 . 194336944

-3 .52861935

-3 .257832024

-4 .099574533

1 .706411344

0 .605257218

-0 .364963339

-3 . 1 10804006

-1 .225220214

-4 .822848539

-4 .067113733

-0 . 136652341

-1 .583922066

0 .699023329

0 .317996602

0 .345165525

1 . 178552289

-0 .609619177

0 . 146728464

-0 .277849813

0 .69635529

-0 .034403623

-0 .455357548

-0 .352985719

-0 .518376786

1 .050742056

-0 .026285928

-0 .333684473

-0 . 13738411

1 .549066659

-1 .867046997

0 .970176333

-1 .879077875

2 .445651577

0 .717766638

-2 .354558414

0 .273802195

-1 .096703814

2 .476960098

-3 .653807855

-3 .565791865

-3 .783231559

-0 . 194987363

0 . 1 1896 0 .69885 0 .26186 0 .07515 0 .09612 0 . 14445 0 . 14556 0 .27564 0 . 19944 0 . 15635 0 .48906 0 .24492 0 . 14189 0 .08698 1 .09632 0 .86516 0 .99943 0 . 10628 0 .35789 0 .24658 0 .35068 0 .8812 0 .90341 1 .02839 0 .06803 0 .30582 0 .09529 0 .09003 0 .06251 0 .07742 0 .08907 0 .06193 0 .08961 0 .24608 0 . 1 1562 0 .26815 0 .26229 0 .09692 0 . 16838 0 .06512 0 . 16984 0 . 1 1496 0 .20124 0 .30679 0 .69296 0 .80303 0 .4969 0 . 12978 0 .9546 0 .80047 0 .38516 0 .28378 0 .93056 0 .73272 0 .90092 0 .27128

-0 .4288 8 .77003 1 .55088 -5 .2077 1 .9765 12 . 188 7 .24599 2 .86737 8 .28876 1 1 .6397 1 .89467 9 .22429 3 .09955 -2 .2343 -3 .2186 -3 .7656 -4 . 1019 16 .0556 1 .69119 -1 .4801 -8 .8707 -1 .3904 -5 .3385 -3 .9548 -2 .0087 -5 . 1793 7 .33585 3 .53223 5 .5217 15 .2236 -6 .844 2 .36918 -3 . 1006 2 .82983 -0 .2975 -1 .6981 -1 .3458 -5 .3483 6 .24047 -0 .4036 -1 .9647 -1 . 1951 7 .69751 -6 .0857 1 .40004 -2 .34 4 .92184 5 .53056 -2 .4665 0 .34205 -2 .8474 8 .7283 -3 .9265 -4 .8665 -4 . 1993 -0 .7188

0 .6681 1 .79E-18 0 . 12093 1 .91E-07 0 .0481 3 .60E-34 4 .29E-13 0 .00414 1 . 14E-16 2 .59E-31 0 .05814 2 .85E-20 0 .00194 0 .02547 0 .00129 0 .00017 4 . 10E-05 5 .22E-58 0 .0908 0 . 13885 7 .27E-19 0 . 16441 9 .37E-08 7 .66E-05 0 .04457 2 .23E-07 2 .20E-13 0 .00041 3 .36E-08 2 .47E-52 7 .70E-12 0 .01783 0 .00193 0 .00466 0 .76605 0 .08948 0 . 17837 8 .88E-08 4 .36E-10 0 .68648 0 .04945 0 .23206 1 .39E-14 1 . 16E-09 0 . 1615 0 .01928 8 .57E-07 3 . 19E-08 0 .01364 0 .73231 0 .00441 2 .59E-18 8 .62E-05 1 . 14E-06 2 .68E-05 0 .47228

0 .71268 2 .31E-17 0 . 15649 6 .35E-07 0 .06756 2 .40E-32 2 .95E-12 0 .00707 1 .20E-15 1 .29E-29 0 .08027 4 .51E-19 0 .00349 0 .03776 0 .00239 0 .00035 9 .60E-05 3 .58E-55 0 . 12084 0 . 1774 9 .79E-18 0 .20681 3 .27E-07 0 .00017 0 .06303 7 .32E-07 1 .56E-12 0 .00082 1 .25E-07 9 . 10E-50 4 .54E-1 1 0 .02713 0 .00348 0 .00787 0 .79947 0 . 1 1922 0 .22272 3 . 1 1E-07 2 .07E-09 0 .72945 0 .06929 0 .28149 1 . 13E-13 5 .22E-09 0 .20343 0 .0292 2 .59E-06 1 . 19E-07 0 .02117 0 .77035 0 .00748 3 .26E-17 0 .00019 3 .36E-06 6 .46E-05 0 .5279

| CSTB 3050 .477014 1 .331839579 | 0 . 19761 6 .73958 | 1 .59E-1 1 | 9 .04E-1 1 |
| --- | --- | --- | --- |
| CSTF1 2688 .753825 0 . 1 15052657 | 0 .08367 1 .37514 | 0 . 16909 | 0 .21228 |
| CSTF2 966 .2096204 -0 .000534672 | 0 .07277 -0 .0073 | 0 .99414 | 0 .995 |
| CSTF2T 3177 .818009 -0 .032455063 | 0 .08055 -0 .4029 | 0 .68701 | 0 .72993 |
| CSTF3 1507 .784156 -0 .230064073 | 0 .07678 -2 .9963 | 0 .00273 | 0 .0048 |
| CSTL1 4 .764536535 -2 .552473359 | 0 .56652 -4 .5055 | 6 .62E-06 | 1 .75E-05 |
| CT45A5 0 .094279041 -1 .064407988 | 3 .0711 1 -0 .3466 | 0 .7289 | 0 .76751 |
| CT47B1 1 .353203713 -2 .094421796 | 0 .94225 -2 .2228 | 0 .02623 | 0 .03881 |
| CT55 6 .315780845 -0 .520613748 | 0 .50485 -1 .0312 | 0 .30244 | 0 .35626 |
| CT62 9 .655429654 -1 .593516279 | 0 .4204 -3 .7905 | 0 .00015 | 0 .00032 |
| CT83 3 . 148213665 -1 .698820187 | 0 .67676 -2 .5102 | 0 .01207 | 0 .0189 |
| CTAG1B 1 .568836818 3 .434056067 | 2 .3294 1 .47422 | 0 . 14042 | 0 . 1791 |
| CTAG2 193 .813886 5 .055661422 | 1 .04238 4 .85012 | 1 .23E-06 | 3 .63E-06 |
| CTAGE1 13 .91244597 -4 .579268815 | 0 .6552 -6 .9891 | 2 .77E-12 | 1 .74E-1 1 |
| CTAGE5 170 .5214897 -0 .008926613 | 0 .09767 -0 .0914 | 0 .92718 | 0 .93927 |
| CTAGE6 24 .53187408 -0 .824339069 | 0 .28311 -2 .9118 | 0 .00359 | 0 .0062 |
| CTAGE8 37 .41863633 -1 . 126485539 | 0 .30956 -3 .639 | 0 .00027 | 0 .00056 |
| CTAGE9 32 .67416643 -1 .028609849 | 0 .28742 -3 .5787 | 0 .00035 | 0 .0007 |
| CTB-129P6 . 113 .30541445 -0 .579669619 | 0 . 19055 -3 .0421 | 0 .00235 | 0 .00417 |
| CTB-133G6 . 174 . 12681403 2 . 150791464 | 0 .27712 7 .76118 | 8 .41E-15 | 7 .00E-14 |
| CTB-134H23 3 .08687648 -1 .500418965 | 0 .4682 -3 .2046 | 0 .00135 | 0 .0025 |
| CTB-167G5 .63 .978391902 -1 .539892762 | 0 .57536 -2 .6764 | 0 .00744 | 0 .01214 |
| CTB-25B13 .545 .31040852 -0 .568181208 | 0 . 1774 -3 .2028 | 0 .00136 | 0 .00251 |
| CTB-50L17 . 1986 .3344627 0 .362124413 | 0 . 1 145 3 . 1626 | 0 .00156 | 0 .00286 |
| CTB-50L17 . 1 12 .89954992 1 .410657641 | 0 .61315 2 .30067 | 0 .02141 | 0 .03217 |
| CTB-78H18 . 10 .839219782 -2 .88412536 | 1 . 15941 -2 .4876 | 0 .01286 | 0 .02006 |
| CTBP1 5980 .8359 0 .400169668 | 0 .07071 5 .65933 | 1 .52E-08 | 5 .92E-08 |
| CTBP2 3588 .717603 0 .239655045 | 0 .09958 2 .40669 | 0 .0161 | 0 .02466 |
| CTBS 2974 .53738 -0 .205145396 | 0 . 10633 -1 .9293 | 0 .0537 | 0 .07469 |
| CTC-236F12 78 .75070935 -1 .252568613 | 0 . 17367 -7 .2124 | 5 .50E-13 | 3 .73E-12 |
| CTC-241N9 . 159 .2493554 -0 .41877089 | 0 . 1 1596 -3 .6114 | 0 .0003 | 0 .00062 |
| CTC-242N15 4 .389805298 -3 .036856454 | 0 .59082 -5 . 1401 | 2 .75E-07 | 8 .92E-07 |
| CTC-343N3 . 1437 .7848653 -0 .318227565 | 0 . 13962 -2 .2792 | 0 .02265 | 0 .03387 |
| CTC-429P9 .42 .50709313 -0 .880391342 | 0 .34788 -2 .5307 | 0 .01138 | 0 .01791 |
| CTC-435M100 .327177372 0 .402689076 | 0 .79519 0 .50641 | 0 .61257 | 0 .66187 |
| CTC-454I21 .36 .28531026 -1 .224863129 | 0 .28879 -4 .2414 | 2 .22E-05 | 5 .43E-05 |
| CTC-479C5 . 1133 .655418 0 .473192529 | 0 . 1368 3 .45889 | 0 .00054 | 0 .00106 |
| CTC-487M236 .987823174 -0 .667826133 | 0 .24676 -2 .7063 | 0 .0068 | 0 .011 17 |
| CTC-529I10 .**2**2 .27779943 -0 .725666711 | 0 .21525 -3 .3713 | 0 .00075 | 0 .00144 |
| CTC-534A2 .2307 .3705779 -0 .359536902 | 0 . 1 1397 -3 . 1548 | 0 .00161 | 0 .00293 |
| CTC-554D6 . 10 .92064995 -0 .793818826 | 0 .62789 -1 .2643 | 0 .20613 | 0 .25336 |
| CTC-575C13 7 .639767025 -0 .869345096 | 0 .29884 -2 .909 | 0 .00363 | 0 .00625 |
| CTC1 828 .0216442 -0 .204828827 | 0 . 13028 -1 .5722 | 0 . 1 159 | 0 . 15072 |
| CTCF 2790 .407688 0 .231927306 | 0 .0646 3 .59013 | 0 .00033 | 0 .00067 |
| CTCFL 30 .29594096 -1 .586214777 | 0 .73388 -2 . 1614 | 0 .03066 | 0 .04471 |
| CTD-2006C10 .046550843 -0 .743812114 | 3 .0711 1 -0 .2422 | 0 .80863 | 0 .83744 |
| CTD-2006C1 14 .91181767 -1 .008336668 | 0 . 1911 1 -5 .2763 | 1 .32E-07 | 4 .50E-07 |
| CTD-2014B14 .056802005 -1 .071175193 | 0 .41629 -2 .5732 | 0 .01008 | 0 .01602 |
| CTD-2021H9 2 .71147884 -1 .914522539 | 0 .59883 -3 . 1971 | 0 .00139 | 0 .00256 |
| CTD-2026K1 106 .6419313 -0 .484950093 | 0 .23834 -2 .0347 | 0 .04188 | 0 .0595 |
| CTD-2054N2 174 .3567322 0 .003802562 | 0 .21281 0 .01787 | 0 .98574 | 0 .98796 |
| CTD-2105E1 0 . 127592317 -0 .511224058 | 3 .0711 1 -0 . 1665 | 0 .86779 | 0 .88892 |
| CTD-2116N1 19 .94426212 -0 .634875202 | 0 .2334 -2 .7202 | 0 .00652 | 0 .01075 |
| CTD-2117L1**2** .874529384 -3 .489826307 | 0 .66355 -5 .2593 | 1 .45E-07 | 4 .89E-07 |
| CTD-2144E2 1 .011832303 -0 .256764996 | 0 .67887 -0 .3782 | 0 .70527 | 0 .74685 |
| CTD-2162K1 13 .41364963 -0 .864005694 | 0 .34862 -2 .4784 | 0 .0132 | 0 .02054 |

CTD-2192J16110 . 1215652 CTD-2192J160 .744066085 CTD-2203A3 5 . 140306865 CTD-2207O20 .286178844 CTD-2260A1 72 .09076712 CTD-2267D1 183 .0118486 CTD-2330K9 13 .57727919 CTD-2349B8 2 .605750753 CTD-2368P2 276 .9494551 CTD-2369P2 0 . 16695462 CTD-2370N5 19 .92355517 CTD-2371O326 .70058328 CTD-2527I21191 .0379818 CTD-2528L192 .700242597 CTD-2535L242 .006126541 CTD-2547L2435 .30269716 CTD-2550O81 .881625872 CTD-2600O922 .48640864 CTD-2616J111 .007453224 CTD-3088G3343 .6412486 CTD-3105H18 .37203157 CTD-3105H18 .910781127 CTD-3193O123 .27885954 CTD-3214H1 1 .415270869 CTD-3222D13 .653204009

| CTDNEP1 CTDP1 CTDSP1 CTDSP2 CTDSPL CTDSPL2 CTF1 CTGF CTH CTHRC1 CTIF CTLA4 CTNNA1 CTNNA2 CTNNA3 CTNNAL1 CTNNB1 CTNNBIP1 CTNNBL1 CTNND1 CTNND2 CTNS CTPS1 CTPS2 CTR9 CTRB1 CTRB2 CTRC CTRL CTSA CTSB | 1290 .961012 1930 .360429 4012 . 100091 8617 .680398 7838 .524767 6528 .062632 25 .89500002 4952 . 13021 506 .6508472 4110 .747539 1 157 .353129 260 . 1263066 28865 . 18827 471 .805874 101 .0958925 3587 .462073 54697 .70106 5974 . 180775 2139 .46051 22214 .65296 87 .67832926 1310 .78299 1958 .340167 1934 .398753 3574 . 178825 3 .810814734 3 .308558859 10 . 17127032 2 .206674335 4342 .533657 73682 .06983 |
| --- | --- |

0 .495930003

1 .397271769

-3 .872371884

-0 .940177344

-0 .466186191

1 .201316077

-0 .6672375

-1 .84249964

-0 .415177771

-0 .071195911

-0 .772514446

-1 .435953094

-1 .278135014

-1 .35658784

-0 .227559417

0 .800488539

0 .29672946

-0 .952258183

-0 .294015707

-0 .03519494

-0 .748783435

-0 .638212526

-0 .968352357

-0 .851314492

-0 . 165569286

0 .307168916

0 .667372

-0 .068489959

-0 .071603051

-0 .655716001

-0 .2067986

-0 .245450376

1 .760620989

-0 .83570183

2 .661169788

0 .791439611

2 .625279891

-0 .011267594

2 .04149355

-1 .267742962

-0 . 1951187

0 .657887834

-1 .004377618

0 . 194940059

-0 . 128676255

0 . 140791356

0 .632974255

1 .407182339

-0 .360348836

0 .055119995

0 .036915929

-0 .358140908

-1 .492408177

0 .646083151

1 .257884736

2 . 199342242

0 .09334 0 .67844 0 .70772 1 .50678 0 . 12571 0 . 14457 0 .24503 0 .4438 0 . 15316 1 .84286 0 .26396 0 . 17202 0 .37921 0 .45766 0 .42236 0 .20385 0 .41385 0 .25367 0 .51261 0 . 16725 0 .28414 0 .21884 0 . 16446 0 .51541 0 .365 0 .08609 0 .08063 0 .07345 0 .08302 0 . 10082 0 .07853 0 . 17781 0 .24278 0 . 15622 0 .22682 0 . 12848 0 .25274 0 .09419 0 .39079 0 .31066 0 . 14147 0 . 1 1812 0 . 15838 0 .07037 0 .09572 0 .42746 0 .08836 0 . 12936 0 .07356 0 .06935 0 .38627 0 .61335 0 .36576 0 .42916 0 . 13148 0 .21794

5 .3134 2 .05954 -5 .4716 -0 .624 -3 .7084 8 .30946 -2 .723 -4 . 1517 -2 .7108 -0 .0386 -2 .9266 -8 .3477 -3 .3705 -2 .9642 -0 .5388 3 .92685 0 .717 -3 .7539 -0 .5736 -0 .2104 -2 .6352 -2 .9163 -5 .888 -1 .6517 -0 .4536 3 .56803 8 .27649 -0 .9324 -0 .8625 -6 .5037 -2 .6333 -1 .3804 7 .2518 -5 .3494 1 1 .7326 6 . 16021 10 .3872 -0 . 1 196 5 .22403 -4 .0808 -1 .3792 5 .56974 -6 .3415 2 .77018 -1 .3443 0 .32937 7 . 16359 10 .8784 -4 .8989 0 .79485 0 .09557 -0 .5839 -4 .0803 1 .50547 9 .56738 10 .0915

1 .08E-07 0 .03944 4 .46E-08 0 .53265 0 .00021 9 .61E-17 0 .00647 3 .30E-05 0 .00671 0 .96918 0 .00343 6 .96E-17 0 .00075 0 .00304 0 .59004 8 .61E-05 0 .47338 0 .00017 0 .56626 0 .83333 0 .00841 0 .00354 3 .91E-09 0 .09859 0 .65011 0 .00036 1 .27E-16 0 .3511 1 0 .38841 7 .84E-1 1 0 .00846 0 . 16745 4 . 1 1E-13 8 .82E-08 8 .68E-32 7 .26E-10 2 .84E-25 0 .90478 1 .75E-07 4 .49E-05 0 . 16784 2 .55E-08 2 .27E-10 0 .0056 0 . 17885 0 .74188 7 .86E-13 1 .46E-27 9 .64E-07 0 .4267 0 .92386 0 .55928 4 .50E-05 0 . 1322 1 . 10E-21 6 .02E-24

3 .72E-07 0 .05628 1 .62E-07 0 .58629 0 .00044 1 .01E-15 0 .01067 7 .86E-05 0 .01103 0 .9738 0 .00593 7 .46E-16 0 .00144 0 .0053 0 .64096 0 .00019 0 .52891 0 .00037 0 .61825 0 .85913 0 .01357 0 .00612 1 .64E-08 0 . 12996 0 .69628 0 .00073 1 .32E-15 0 .40658 0 .4447 4 .08E-10 0 .01364 0 .21038 2 .83E-12 3 .09E-07 4 .56E-30 3 .36E-09 7 .66E-24 0 .92091 5 .84E-07 0 .0001 0 .21082 9 .65E-08 1 . 12E-09 0 .00935 0 .22327 0 .77857 5 .25E-12 5 .07E-26 2 .88E-06 0 .48323 0 .93653 0 .6116 0 .0001 0 . 16962 2 .01E-20 1 .41E-22

| CTSC CTSD CTSE CTSF CTSG CTSH CTSK CTSL CTSO CTSS CTSV CTSW CTSZ CTTN CTTNBP2 | 16143 .5856 10138 .26402 15 . 15759764 6190 .007881 1089 .746341 9175 .260416 33676 .56408 1 1 186 .42384 4416 .215737 12011 .23002 2471 .895727 332 .6049109 1 1372 .60095 1 1500 .04121 1288 .376894 |
| --- | --- |

CTTNBP2NL 3392 .722317

| CTU1 CTU2 CTXN1 CTXN2 CTXN3 CUBN CUEDC1 CUEDC2 CUL1 CUL2 CUL3 CUL4A CUL4B CUL5 CUL7 CUL9 CUTA CUTC CUX1 CUX2 CUZD1 CWC15 CWC22 CWC27 CWF19L1 CWF19L2 CWH43 CX3CL1 CX3CR1 CXADR CXCL1 CXCL10 CXCL11 CXCL12 CXCL13 CXCL14 CXCL16 CXCL17 CXCL2 CXCL3 | 325 .5510599 620 .0937847 30 .7374113 7 .909473181 7 .73697495 1906 .27671 1289 .649753 1665 .042572 6735 .861599 2693 . 145712 7273 .45793 8328 .293675 6991 .401479 7685 .272936 3152 .797 3147 .801303 5074 .680757 834 .6955777 5095 .709482 148 .2514823 93 .65379851 4372 . 120883 2000 .898197 940 .3655116 1930 .058386 2449 .298604 2063 .291324 842 .9494969 432 .5743878 7084 .451558 491 .6498809 4020 .353453 686 .6725899 10292 .52666 512 .7642324 19724 .07971 945 .9080741 20 .93583326 204 .2874155 90 .37580452 |
| --- | --- |

0 .856321091

2 .313583152

-0 .558343746

-0 .411 176942

-0 .09795685

0 .469901384

0 .982714581

1 .54564242

-0 .353052503

2 .229939663

-0 .819316411

2 .023118403

2 .008816339

0 .378705737

-1 . 108735647

-0 .254239662

1 .446311471

0 .481841774

2 .881038134

-2 .030969678

-2 .394998065

-0 .655597279

0 .476081931

-0 . 136955862

0 .225800711

-0 .206086171

-0 .070840498

0 .076949826

-0 .295976703

-0 .482496981

0 .228740733

-0 .093908214

0 . 13004638

-0 .357154085

0 .578302388

1 .993305826

-1 . 153695924

-0 .620437901

-0 .547465756

-0 .397911253

-0 . 166885209

-0 .868442855

-0 . 19389841

0 .884498699

0 .547034972

-0 .288057804

3 .867203035

4 .520350768

4 .012707001

1 .30536804

5 .026733853

0 .38862676

1 .853971172

-0 .211534794

2 .789594746

2 .615057514

0 . 13416 0 . 19065 0 .37326 0 . 13062 0 .28008 0 . 1476 0 . 19033 0 .2034 0 . 12057 0 .20337 0 .29532 0 .24962 0 . 17904 0 . 10232 0 . 17764 0 .08903 0 . 17634 0 . 1 1907 0 .29032 0 .51518 0 .61388 0 .20674 0 .09413 0 . 12182 0 .07893 0 .06585 0 .07852 0 .09869 0 .07873 0 .09744 0 . 12172 0 .09957 0 . 17314 0 .0924 0 . 1213 0 .44437 0 . 15786 0 . 10315 0 .09505 0 .09848 0 .07257 0 . 10331 0 .2538 0 . 18335 0 .21029 0 .24718 0 .37658 0 .40792 0 .38526 0 .238 0 .4554 0 .26062 0 . 14362 0 .26737 0 .29933 0 .29565

6 .38296 12 . 1352 -1 .4959 -3 . 1478 -0 .3497 3 . 18368 5 . 16334 7 .59892 -2 .9281 10 .9648 -2 .7743 8 . 10486 1 1 .2201 3 .70104 -6 .2415 -2 .8557 8 .20187 4 .04671 9 .92371 -3 .9423 -3 .9014 -3 . 1712 5 .05746 -1 . 1242 2 .86093 -3 . 1297 -0 .9022 0 .77968 -3 .7593 -4 .9519 1 .8792 -0 .9431 0 .7511 1 -3 .8653 4 .76736 4 .48566 -7 .3083 -6 .0149 -5 .7596 -4 .0406 -2 .2995 -8 .4065 -0 .764 4 .82397 2 .60133 -1 . 1654 10 .2692 1 1 .0814 10 .4157 5 .48467 1 1 .038 1 .49114 12 .909 -0 .7912 9 .31941 8 .84516

1 .74E-10 6 .87E-34 0 . 13469 0 .00164 0 .72653 0 .00145 2 .43E-07 2 .99E-14 0 .00341 5 .64E-28 0 .00553 5 .28E-16 3 .25E-29 0 .00021 4 .34E-10 0 .00429 2 .37E-16 5 . 19E-05 3 .28E-23 8 .07E-05 9 .56E-05 0 .00152 4 .25E-07 0 .26092 0 .00422 0 .00175 0 .36697 0 .43558 0 .00017 7 .35E-07 0 .06022 0 .34562 0 .45259 0 .00011 1 .87E-06 7 .27E-06 2 .70E-13 1 .80E-09 8 .43E-09 5 .33E-05 0 .02148 4 .23E-17 0 .44488 1 .41E-06 0 .00929 0 .24387 9 .70E-25 1 .55E-28 2 . 10E-25 4 . 14E-08 2 .50E-28 0 . 13593 4 .00E-38 0 .42884 1 . 17E-20 9 . 14E-19

8 .69E-10 4 .48E-32 0 . 17255 0 .00299 0 .76556 0 .00267 7 .93E-07 2 .35E-13 0 .00591 2 .02E-26 0 .00924 5 . 1 1E-15 1 .33E-27 0 .00045 2 .06E-09 0 .00731 2 .38E-15 0 .00012 7 .08E-22 0 .00018 0 .00021 0 .00278 1 .34E-06 0 .31239 0 .0072 0 .00317 0 .4228 0 .49175 0 .00036 2 .24E-06 0 .08295 0 .40122 0 .50848 0 .00024 5 .33E-06 1 .90E-05 1 .90E-12 7 .90E-09 3 .40E-08 0 .00012 0 .03225 4 .63E-16 0 .50077 4 .09E-06 0 .01487 0 .29433 2 .50E-23 5 .89E-27 5 .79E-24 1 .52E-07 9 .39E-27 0 . 17403 3 .88E-36 0 .48542 1 .93E-19 1 .22E-17

| CXCL5 | 359 .2880305 | 4 .982485885 | 0 .68009 | 7 .32619 | 2 .37E-13 | 1 .67E-12 |
| --- | --- | --- | --- | --- | --- | --- |
| CXCL6 | 33 .48130839 | 1 .265935448 | 0 .5863 | 2 . 15921 | 0 .03083 | 0 .04494 |
| CXCL8 | 3083 .424327 | 5 .624128887 | 0 .50938 | 1 1 .0412 | 2 .42E-28 | 9 . 1 1E-27 |
| CXCL9 | 5985 .510954 | 5 .867241058 | 0 .41952 | 13 .9856 | 1 .91E-44 | 3 .49E-42 |
| CXCR1 | 37 .65528204 | 1 .373905119 | 0 .36307 | 3 .78418 | 0 .00015 | 0 .00033 |
| CXCR2 | 129 .6526313 | 0 .352472239 | 0 .22722 | 1 .55126 | 0 . 12084 | 0 . 15638 |
| CXCR3 | 126 .3381235 | 2 .570854117 | 0 .27194 | 9 .45385 | 3 .27E-21 | 5 .66E-20 |
| CXCR4 | 1 1 19 .572136 | 3 .063490666 | 0 .21422 | 14 .3006 | 2 . 17E-46 | 4 .63E-44 |
| CXCR5 | 8 .693587396 | 0 .589626179 | 0 .40394 | 1 .45969 | 0 . 14438 | 0 . 18366 |
| CXCR6 | 838 .0200344 | 0 .555074577 | 0 . 15338 | 3 .61901 | 0 .0003 | 0 .00061 |
| CXXC1 | 1748 .663888 | 0 . 1 12210575 | 0 .07853 | 1 .42881 | 0 . 15306 | 0 . 19373 |
| CXXC4 | 1 12 .5833555 | -0 .509914765 | 0 .30577 | -1 .6677 | 0 .09538 | 0 . 12619 |
| CXXC5 | 2859 .374722 | 1 .339868365 | 0 . 1 1672 | 1 1 .4792 | 1 .68E-30 | 7 .56E-29 |
| CXorf21 | 236 .9303739 | 1 .36863834 | 0 . 18886 | 7 .24672 | 4 .27E-13 | 2 .94E-12 |
| CXorf23 | 1922 .920966 | -0 .558126932 | 0 . 10481 | -5 .3251 | 1 .01E-07 | 3 .50E-07 |
| CXorf36 | 792 .6987448 | 0 .821710691 | 0 . 15562 | 5 .28036 | 1 .29E-07 | 4 .40E-07 |
| CXorf38 | 1 153 .086608 | 0 .713893137 | 0 .09122 | 7 .82569 | 5 .05E-15 | 4 .32E-14 |
| CXorf40A | 728 .779048 | -0 . 15709456 | 0 .09501 | -1 .6534 | 0 .09825 | 0 . 1296 |
| CXorf40B | 888 .6282358 | -0 . 182761696 | 0 .07968 | -2 .2937 | 0 .02181 | 0 .03271 |
| CXorf56 | 2638 .037162 | 0 .057645669 | 0 .08818 | 0 .65376 | 0 .51327 | 0 .56772 |
| CXorf57 | 665 .6194782 | -1 .402056009 | 0 . 17609 | -7 .9623 | 1 .69E-15 | 1 .53E-14 |
| CXorf58 | 17 .56325146 | -1 .210412876 | 0 .24956 | -4 .8502 | 1 .23E-06 | 3 .62E-06 |
| CXorf65 | 76 .9103517 | 0 . 1 10831477 | 0 .20392 | 0 .54352 | 0 .58677 | 0 .63788 |
| CXorf66 | 3 .550325264 | -5 .524974551 | 1 . 10178 | -5 .0146 | 5 .32E-07 | 1 .65E-06 |
| CXorf67 | 6 .816875455 | -2 .686044291 | 0 .41431 | -6 .4832 | 8 .98E-1 1 | 4 .64E-10 |
| CYB561 | 2490 .460425 | 0 .281415234 | 0 .09424 | 2 .98613 | 0 .00283 | 0 .00495 |
| CYB561A3 | 4380 .070558 | 0 .214691646 | 0 . 14215 | 1 .51033 | 0 . 13096 | 0 . 16823 |
| CYB561D1 | 1369 .619985 | -0 . 100026193 | 0 . 12942 | -0 .7729 | 0 .43959 | 0 .49548 |
| CYB561D2 | 828 .4236211 | 0 .648328093 | 0 . 12885 | 5 .03157 | 4 .86E-07 | 1 .52E-06 |
| CYB5A | 3183 .062643 | 0 . 150815047 | 0 .23077 | 0 .65352 | 0 .51342 | 0 .56779 |
| CYB5B | 4668 .007467 | 0 . 166581757 | 0 .09802 | 1 .69955 | 0 .08922 | 0 . 1 189 |
| CYB5D1 | 880 .7710781 | -0 .223789153 | 0 .08672 | -2 .5807 | 0 .00986 | 0 .0157 |
| CYB5D2 | 1 190 .240079 | 0 .068951309 | 0 .099 | 0 .69648 | 0 .48613 | 0 .54123 |
| CYB5R1 | 7067 .850586 | -0 .496783464 | 0 .09471 | -5 .2454 | 1 .56E-07 | 5 .24E-07 |
| CYB5R2 | 1520 .996411 | -1 . 179649136 | 0 . 15006 | -7 .8614 | 3 .80E-15 | 3 .32E-14 |
| CYB5R3 | 3229 .818712 | 0 .700948627 | 0 . 12314 | 5 .69212 | 1 .25E-08 | 4 .94E-08 |
| CYB5R4 | 2266 .92693 | -0 . 135459026 | 0 .07909 | -1 .7127 | 0 .08676 | 0 . 1 1585 |
| CYB5RL | 386 .3741821 | 0 .072614492 | 0 . 1034 | 0 .70229 | 0 .4825 | 0 .53766 |
| CYBA | 6605 .771247 | 1 .67763732 | 0 . 18445 | 9 .09517 | 9 .44E-20 | 1 .41E-18 |
| CYBB | 7718 .513199 | 2 . 192670796 | 0 .21064 | 10 .4096 | 2 .24E-25 | 6 . 1 1E-24 |
| CYBRD1 | 19976 .57004 | -0 .70444308 | 0 . 17001 | -4 . 1435 | 3 .42E-05 | 8 . 12E-05 |
| CYC1 | 6681 .581907 | 0 .604939621 | 0 . 13933 | 4 .34175 | 1 .41E-05 | 3 .56E-05 |
| CYCS | 8467 .780359 | 0 .424245701 | 0 . 14917 | 2 .84403 | 0 .00445 | 0 .00756 |
| CYFIP2 | 1672 .479454 | 1 .00270108 | 0 . 18133 | 5 .52981 | 3 .21E-08 | 1 . 19E-07 |
| CYGB | 1202 .253002 | -0 .882721 | 0 .22632 | -3 .9003 | 9 .61E-05 | 0 .00021 |
| CYHR1 | 1827 .049559 | 0 .491380119 | 0 .08053 | 6 . 10167 | 1 .05E-09 | 4 .76E-09 |
| CYLC1 | 1 1 .06176268 | -6 .836933109 | 0 .93128 | -7 .3414 | 2 . 1 1E-13 | 1 .50E-12 |
| CYLC2 | 14 .40258021 | -6 .48235923 | 0 .73288 | -8 .845 | 9 . 15E-19 | 1 .22E-17 |
| CYLD | 7701 .979931 | 0 .039439094 | 0 . 10313 | 0 .3824 | 0 .70216 | 0 .74381 |
| CYP11A1 | 29 .56707179 | 0 . 108100711 | 0 .30001 | 0 .36032 | 0 .71861 | 0 .75859 |
| CYP11B1 | 7 .844820948 | -1 .992674735 | 0 .46934 | -4 .2457 | 2 . 18E-05 | 5 .34E-05 |
| CYP11B2 | 3 .614605606 | -3 .821303045 | 0 .72498 | -5 .2709 | 1 .36E-07 | 4 .62E-07 |
| CYP17A1 | 31 .77497433 | -1 .081744269 | 0 .24525 | -4 .4107 | 1 .03E-05 | 2 .64E-05 |
| CYP19A1 | 342 .0323898 | 0 .828861343 | 0 .29798 | 2 .78164 | 0 .00541 | 0 .00905 |
| CYP1A1 | 125 .2386034 | -0 .042360761 | 0 .37993 | -0 . 1 1 15 | 0 .91122 | 0 .92641 |
| CYP1A2 | 7 .530919705 | -3 .700416899 | 0 .5392 | -6 .8627 | 6 .75E-12 | 4 .02E-1 1 |

| CYP1B1 | 2404 .549396 | 0 .982747454 | 0 .23866 | 4 . 1 1782 | 3 .82E-05 | 9 .00E-05 |
| --- | --- | --- | --- | --- | --- | --- |
| CYP20A1 | 1863 .499986 | 0 . 135614414 | 0 .06269 | 2 . 1632 | 0 .03053 | 0 .04454 |
| CYP21A2 | 16 .68148635 | -1 .302050709 | 0 .27421 | -4 .7484 | 2 .05E-06 | 5 .81E-06 |
| CYP24A1 | 88 .74337197 | -0 .577264301 | 0 .35249 | -1 .6377 | 0 . 10149 | 0 . 13347 |
| CYP26A1 | 15 . 16881186 | 0 .333999006 | 0 .43767 | 0 .76314 | 0 .44538 | 0 .50122 |
| CYP26B1 | 1492 .793919 | 0 .554120851 | 0 . 18864 | 2 .93752 | 0 .00331 | 0 .00574 |
| CYP26C1 | 7 .83197742 | -0 .25732554 | 0 .30454 | -0 .845 | 0 .39814 | 0 .4548 |
| CYP27A1 | 5883 .286856 | 0 .908214667 | 0 . 18263 | 4 .97295 | 6 .59E-07 | 2 .02E-06 |
| CYP27B1 | 83 .54579444 | 0 .731566469 | 0 . 19744 | 3 .70535 | 0 .00021 | 0 .00044 |
| CYP27C1 | 285 .3282051 | 0 . 167931647 | 0 .22607 | 0 .74282 | 0 .45759 | 0 .51345 |
| CYP2A13 | 3 .394222532 | -3 .674317653 | 0 .73642 | -4 .9894 | 6 .06E-07 | 1 .87E-06 |
| CYP2A6 | 22 .36779125 | 0 .026016019 | 0 .31138 | 0 .08355 | 0 .93341 | 0 .94464 |
| CYP2A7 | 13 .47342475 | -1 .016188747 | 0 .31046 | -3 .2732 | 0 .00106 | 0 .002 |
| CYP2B6 | 14 .35318284 | -1 .823985995 | 0 .68479 | -2 .6636 | 0 .00773 | 0 .01256 |
| CYP2C18 | 194 .7019012 | -1 .295930182 | 0 .27374 | -4 .7342 | 2 .20E-06 | 6 .20E-06 |
| CYP2C19 | 62 .03815567 | -2 .468231173 | 0 .31291 | -7 .888 | 3 .07E-15 | 2 .71E-14 |
| CYP2C8 | 19 . 14846635 | -1 .867897543 | 0 .32617 | -5 .7268 | 1 .02E-08 | 4 .07E-08 |
| CYP2C9 | 37 .96663997 | -2 . 167730498 | 0 .31784 | -6 .8203 | 9 .09E-12 | 5 .31E-1 1 |
| CYP2D6 | 72 .03469935 | 0 . 132799435 | 0 .21676 | 0 .61266 | 0 .5401 | 0 .5934 |
| CYP2E1 | 283 .3609906 | -1 .441525865 | 0 .22728 | -6 .3424 | 2 .26E-10 | 1 . 1 1E-09 |
| CYP2F1 | 7 .231313373 | -2 .746422094 | 0 .54628 | -5 .0275 | 4 .97E-07 | 1 .55E-06 |
| CYP2J2 | 388 . 132358 | -1 .458409542 | 0 .20375 | -7 . 1577 | 8 .20E-13 | 5 .47E-12 |
| CYP2R1 | 1790 .938911 | -0 .467666747 | 0 . 10649 | -4 .3917 | 1 . 12E-05 | 2 .87E-05 |
| CYP2S1 | 128 .3968102 | 0 .699033332 | 0 . 16595 | 4 .21231 | 2 .53E-05 | 6 . 13E-05 |
| CYP2U1 | 3683 .331872 | -0 .267489561 | 0 . 13614 | -1 .9648 | 0 .04944 | 0 .06928 |
| CYP2W1 | 74 .08934354 | -1 .906821688 | 0 .3128 | -6 .0959 | 1 .09E-09 | 4 .92E-09 |
| CYP39A1 | 1750 .447331 | -2 .788589031 | 0 .20205 | -13 .802 | 2 .49E-43 | 4 . 15E-41 |
| CYP3A4 | 107 .2141763 | -0 .928376031 | 0 .3416 | -2 .7178 | 0 .00657 | 0 .01082 |
| CYP3A43 | 20 .80119794 | -1 .652159992 | 0 .31541 | -5 .2382 | 1 .62E-07 | 5 .43E-07 |
| CYP3A5 | 1589 .351415 | -2 . 149198419 | 0 . 19262 | -1 1 . 158 | 6 .55E-29 | 2 .58E-27 |
| CYP3A7 | 65 .23518916 | -0 .78910812 | 0 . 17333 | -4 .5527 | 5 .30E-06 | 1 .42E-05 |
| CYP46A1 | 102 .6298637 | -0 .882799622 | 0 .27886 | -3 . 1658 | 0 .00155 | 0 .00283 |
| CYP4A11 | 83 .83516037 | -1 . 172771955 | 0 .43636 | -2 .6876 | 0 .0072 | 0 .01177 |
| CYP4A22 | 24 .58806338 | -1 .076365646 | 0 .30639 | -3 .5131 | 0 .00044 | 0 .00088 |
| CYP4B1 | 858 .3613709 | -1 .761377907 | 0 .35872 | -4 .9101 | 9 . 10E-07 | 2 .74E-06 |
| CYP4F11 | 58 .94121228 | 0 .412285382 | 0 .44099 | 0 .9349 | 0 .34984 | 0 .40528 |
| CYP4F12 | 1 172 .01439 | -2 .467962239 | 0 .3074 | -8 .0284 | 9 .87E-16 | 9 . 15E-15 |
| CYP4F2 | 283 .6494043 | 1 . 19768024 | 0 .57469 | 2 .08404 | 0 .03716 | 0 .05328 |
| CYP4F22 | 1 1 10 .932696 | 0 .690165069 | 0 .31269 | 2 .20718 | 0 .0273 | 0 .04025 |
| CYP4F3 | 391 .8482244 | -0 .344052338 | 0 .28937 | -1 . 189 | 0 .23445 | 0 .28409 |
| CYP4V2 | 5193 .794067 | -0 .508714219 | 0 . 15456 | -3 .2914 | 0 .001 | 0 .00188 |
| CYP4X1 | 260 .0344006 | 0 .263862307 | 0 .27154 | 0 .97173 | 0 .33118 | 0 .38616 |
| CYP4Z1 | 42 .5281011 1 | -0 .594089772 | 0 .37694 | -1 .5761 | 0 . 1 1501 | 0 . 14966 |
| CYP51A1 | 848 . 141157 | 0 .500856188 | 0 . 14023 | 3 .57156 | 0 .00035 | 0 .00072 |
| CYP7A1 | 105 .517914 | -2 .381362602 | 0 .30324 | -7 .853 | 4 .06E-15 | 3 .53E-14 |
| CYP7B1 | 2780 .506408 | 0 .719878575 | 0 . 1436 | 5 .01294 | 5 .36E-07 | 1 .66E-06 |
| CYP8B1 | 23 .85410099 | -0 . 156331557 | 0 .32391 | -0 .4826 | 0 .62936 | 0 .67765 |
| CYR61 | 1377 .824275 | 2 .598632112 | 0 .26761 | 9 .71046 | 2 .72E-22 | 5 .31E-21 |
| CYS1 | 251 .9932914 | 0 .571668864 | 0 .25091 | 2 .2784 | 0 .0227 | 0 .03394 |
| CYSLTR1 | 613 .6694859 | 0 .430280712 | 0 . 17503 | 2 .45832 | 0 .01396 | 0 .02162 |
| CYSLTR2 | 538 .4130815 | 1 .906080327 | 0 .37274 | 5 . 1 1371 | 3 . 16E-07 | 1 .02E-06 |
| CYSRT1 | 769 .5211283 | -0 .798783846 | 0 .23231 | -3 .4385 | 0 .00059 | 0 .00114 |
| CYSTM1 | 1728 . 147695 | -0 .043324374 | 0 . 14844 | -0 .2919 | 0 .7704 | 0 .80318 |
| CYTH1 | 5414 .710038 | 0 .520913246 | 0 .09066 | 5 .74555 | 9 . 16E-09 | 3 .67E-08 |
| CYTH2 | 1566 .779026 | 0 .017314412 | 0 .09074 | 0 . 19081 | 0 .84867 | 0 .87223 |
| CYTH3 | 12349 .83616 | 1 .230276101 | 0 . 16414 | 7 .4954 | 6 .61E-14 | 4 .96E-13 |

| CYTH4 | 2021 .829636 | 1 .905645282 | 0 . 15563 | 12 .2443 | 1 .80E-34 | 1 .23E-32 |
| --- | --- | --- | --- | --- | --- | --- |
| CYTIP | 1307 .763268 | 1 .270839694 | 0 . 19451 | 6 .53352 | 6 .42E-1 1 | 3 .39E-10 |
| CYTL1 | 811 .6321214 | 2 .079133322 | 0 .35345 | 5 .88236 | 4 .04E-09 | 1 .69E-08 |
| CYYR1 | 739 .0971329 | 0 .576871903 | 0 . 16594 | 3 .47637 | 0 .00051 | 0 .001 |
| D2HGDH | 754 .4702945 | 0 .391489691 | 0 .09229 | 4 .24192 | 2 .22E-05 | 5 .42E-05 |
| DAAM1 | 7124 .290929 | -0 .708907876 | 0 . 13841 | -5 . 1219 | 3 .03E-07 | 9 .77E-07 |
| DAAM2 | 3640 .404279 | 1 .04432623 | 0 . 1975 | 5 .28781 | 1 .24E-07 | 4 .24E-07 |
| DAB1 | 78 .79848539 | -1 .313716152 | 0 .31844 | -4 . 1255 | 3 .70E-05 | 8 .72E-05 |
| DAB2 | 7087 .37082 | 0 .936122163 | 0 . 12525 | 7 .47395 | 7 .78E-14 | 5 .78E-13 |
| DAB2IP | 3104 .371994 | -0 .03917623 | 0 . 1 1398 | -0 .3437 | 0 .73107 | 0 .76924 |
| DACH2 | 38 .30239037 | -2 .844068026 | 0 .39495 | -7 .2011 | 5 .97E-13 | 4 .04E-12 |
| DACT1 | 482 .9948184 | 1 . 15606933 | 0 . 19449 | 5 .94408 | 2 .78E-09 | 1 . 19E-08 |
| DACT2 | 214 . 1587013 | -1 .709010982 | 0 .27677 | -6 . 1749 | 6 .62E-10 | 3 .08E-09 |
| DACT3 | 219 .8056567 | 1 . 1 12462724 | 0 . 17436 | 6 .38015 | 1 .77E-10 | 8 .84E-10 |
| DAD1 | 7242 .365763 | -0 .266595578 | 0 . 14793 | -1 .8022 | 0 .07152 | 0 .09701 |
| DAG1 | 6958 .82475 | 0 .849841611 | 0 . 12305 | 6 .90638 | 4 .97E-12 | 3 .01E-1 1 |
| DAGLA | 250 .3699809 | 1 .551434636 | 0 . 16966 | 9 . 1445 | 5 .99E-20 | 9 . 15E-19 |
| DAGLB | 1237 . 151997 | 0 . 147148045 | 0 .07287 | 2 .01929 | 0 .04346 | 0 .06156 |
| DALRD3 | 1339 .478746 | 0 . 126256617 | 0 .09702 | 1 .3013 | 0 . 19316 | 0 .23924 |
| DAND5 | 5 .652276246 | -1 .602770574 | 0 .49815 | -3 .2174 | 0 .00129 | 0 .0024 |
| DAO | 9 .424410165 | -3 .559186223 | 0 .5118 | -6 .9543 | 3 .54E-12 | 2 . 19E-1 1 |
| DAOA | 5 .214748532 | -5 .492464272 | 0 .89263 | -6 . 1531 | 7 .60E-10 | 3 .51E-09 |
| DAP | 2704 .489331 | 0 .856562575 | 0 . 1221 | 7 .01527 | 2 .30E-12 | 1 .46E-1 1 |
| DAP3 | 5489 .603146 | -0 . 178788692 | 0 . 1 1 167 | -1 .6011 | 0 . 10935 | 0 . 14294 |
| DAPK1 | 4517 .754306 | 0 .05415942 | 0 . 12671 | 0 .42743 | 0 .66906 | 0 .71347 |
| DAPK2 | 661 .7895612 | 0 .266031191 | 0 .23834 | 1 . 1 1617 | 0 .26435 | 0 .31595 |
| DAPK3 | 2303 .515136 | 1 . 174357431 | 0 . 1002 | 1 1 .7196 | 1 .01E-31 | 5 .26E-30 |
| DAPL1 | 1822 .415053 | -0 .913156034 | 0 .27731 | -3 .293 | 0 .00099 | 0 .00187 |
| DAPP1 | 2200 .482982 | -0 . 170397623 | 0 . 16648 | -1 .0236 | 0 .30605 | 0 .3602 |
| DARS | 6221 .594259 | -0 .466154557 | 0 .08717 | -5 .3474 | 8 .92E-08 | 3 . 12E-07 |
| DARS2 | 2236 .37673 | 0 .530037201 | 0 . 12205 | 4 .34281 | 1 .41E-05 | 3 .54E-05 |
| DAW1 | 31 . 18809227 | -2 .879657202 | 0 .31304 | -9 . 199 | 3 .61E-20 | 5 .64E-19 |
| DAXX | 2253 .016919 | 0 .238948774 | 0 .08738 | 2 .73465 | 0 .00624 | 0 .01033 |
| DAZ1 | 0 .374380828 | 1 .397838206 | 1 .83721 | 0 .76085 | 0 .44675 | 0 .50258 |
| DAZ2 | 1 .877688353 | 3 .689042805 | 1 .27201 | 2 .90016 | 0 .00373 | 0 .00642 |
| DAZ3 | 3 .025466625 | 4 .378986186 | 1 . 13266 | 3 .86612 | 0 .00011 | 0 .00024 |
| DAZ4 | 1 .364189788 | 3 .236027068 | 1 .25866 | 2 .57101 | 0 .01014 | 0 .01611 |
| DAZAP1 | 3173 .591586 | 0 .774871278 | 0 .07149 | 10 .839 | 2 .25E-27 | 7 .62E-26 |
| DAZAP2 | 8866 .464102 | -0 . 1 18286268 | 0 .07549 | -1 .5669 | 0 . 1 1714 | 0 . 15212 |
| DAZL | 45 .71102775 | 0 .616589108 | 0 .36666 | 1 .68162 | 0 .09264 | 0 . 12299 |
| DBF4 | 1328 .210358 | 0 .585283497 | 0 . 13696 | 4 .27344 | 1 .92E-05 | 4 .76E-05 |
| DBF4B | 266 .5597953 | 0 .38213764 | 0 . 13224 | 2 .88965 | 0 .00386 | 0 .00662 |
| DBH | 21 .50891626 | 0 .463560919 | 0 .31872 | 1 .45443 | 0 . 14583 | 0 . 1854 |
| DBI | 5733 .813875 | -0 .365503397 | 0 . 16282 | -2 .2448 | 0 .02478 | 0 .03681 |
| DBN1 | 2002 .946213 | 1 .485650028 | 0 . 12568 | 1 1 .8207 | 3 .05E-32 | 1 .69E-30 |
| DBNDD1 | 463 .8079314 | 0 .908886052 | 0 . 19522 | 4 .65564 | 3 .23E-06 | 8 .90E-06 |
| DBNDD2 | 134 .8617825 | 0 . 141397258 | 0 . 1 1666 | 1 .21201 | 0 .22551 | 0 .27438 |
| DBNL | 8559 .086667 | 0 .388405103 | 0 .07214 | 5 .38414 | 7 .28E-08 | 2 .58E-07 |
| DBP | 739 .3588596 | -0 .494066859 | 0 . 1 1256 | -4 .3892 | 1 . 14E-05 | 2 .90E-05 |
| DBR1 | 1664 . 129729 | 0 . 1 16357309 | 0 .0772 | 1 .50718 | 0 . 13176 | 0 . 16917 |
| DBT | 2993 .007973 | -0 .016244092 | 0 . 1 1324 | -0 . 1435 | 0 .88593 | 0 .90441 |
| DBX1 | 3 .78026274 | -1 .711 171687 | 0 .47146 | -3 .6295 | 0 .00028 | 0 .00058 |
| DBX2 | 36 .57177732 | -2 .35309388 | 0 .36367 | -6 .4704 | 9 .78E-1 1 | 5 .03E-10 |
| DCAF1 | 1985 .407535 | -0 .030058921 | 0 .07261 | -0 .414 | 0 .6789 | 0 .72234 |
| DCAF10 | 4951 .614649 | 0 .002956535 | 0 .07769 | 0 .03805 | 0 .96964 | 0 .97406 |
| DCAF11 | 4175 .700599 | 0 .025513972 | 0 .06293 | 0 .40541 | 0 .68517 | 0 .72814 |

| DCAF12 | 3250 .649097 | 0 . 186325914 | 0 .06787 | 2 .74535 | 0 .00604 | 0 .01003 |
| --- | --- | --- | --- | --- | --- | --- |
| DCAF12L1 | 62 .24033146 | -0 .613929711 | 0 .67543 | -0 .9089 | 0 .36338 | 0 .41904 |
| DCAF12L2 | 16 .65469283 | 0 .306834364 | 0 .3088 | 0 .99362 | 0 .32041 | 0 .37512 |
| DCAF13 | 4529 .98349 | 0 .270270662 | 0 . 14103 | 1 .91637 | 0 .05532 | 0 .0767 |
| DCAF15 | 687 .3501591 | 0 .430302184 | 0 . 1 1778 | 3 .65339 | 0 .00026 | 0 .00054 |
| DCAF16 | 5966 .32398 | 0 . 178869314 | 0 .08269 | 2 . 16309 | 0 .03053 | 0 .04455 |
| DCAF17 | 2316 .029741 | -0 .230919483 | 0 .07755 | -2 .9775 | 0 .00291 | 0 .00508 |
| DCAF4 | 1016 .961146 | -0 .368863533 | 0 . 1 1 167 | -3 .3032 | 0 .00096 | 0 .00181 |
| DCAF4L1 | 84 .45747466 | 0 .090382585 | 0 . 18757 | 0 .48185 | 0 .62991 | 0 .67813 |
| DCAF4L2 | 14 .85673246 | -1 .92012054 | 0 .47569 | -4 .0365 | 5 .43E-05 | 0 .00012 |
| DCAF5 | 3054 .337421 | -0 . 153370071 | 0 .07473 | -2 .0524 | 0 .04013 | 0 .05721 |
| DCAF6 | 5263 .83798 | -0 .214494448 | 0 .07486 | -2 .8652 | 0 .00417 | 0 .00711 |
| DCAF7 | 3962 . 125905 | 0 .823680796 | 0 . 1022 | 8 .05955 | 7 .66E-16 | 7 . 19E-15 |
| DCAF8 | 4631 .437902 | -0 .094961848 | 0 .08272 | -1 . 148 | 0 .25095 | 0 .3019 |
| DCAF8L1 | 6 .740349314 | -3 .387115778 | 0 .77977 | -4 .3437 | 1 .40E-05 | 3 .53E-05 |
| DCAF8L2 | 10 .46143365 | -2 .688726908 | 1 .04695 | -2 .5681 | 0 .01022 | 0 .01622 |
| DCAKD | 1 180 .019269 | -0 . 162131375 | 0 .07542 | -2 . 1497 | 0 .03158 | 0 .04594 |
| DCBLD1 | 1063 . 150047 | 0 .27038181 | 0 .087 | 3 . 10773 | 0 .00189 | 0 .0034 |
| DCBLD2 | 7833 .543286 | 0 .050722128 | 0 . 19322 | 0 .26251 | 0 .79293 | 0 .82336 |
| DCC | 73 .95951313 | -2 .415161579 | 0 .34182 | -7 .0656 | 1 .60E-12 | 1 .04E-1 1 |
| DCD | 7562 .955453 | 9 .762821926 | 0 .62979 | 15 .5017 | 3 .38E-54 | 1 .41E-51 |
| DCDC1 | 102 .0160106 | -2 .7513293 | 0 .20999 | -13 . 102 | 3 .20E-39 | 3 .43E-37 |
| DCDC2 | 92 .86251207 | 0 .211242391 | 0 .29652 | 0 .71239 | 0 .47622 | 0 .53168 |
| DCDC2B | 91 .30670353 | -0 .288608295 | 0 . 17497 | -1 .6495 | 0 .09905 | 0 . 13049 |
| DCDC2C | 1 1 .6798425 | -2 .817628235 | 0 .45305 | -6 .2192 | 5 .00E-10 | 2 .36E-09 |
| DCHS1 | 2800 .617474 | -0 .384829279 | 0 . 15203 | -2 .5313 | 0 .01136 | 0 .01789 |
| DCHS2 | 354 .2868038 | -1 . 181854609 | 0 .24859 | -4 .7543 | 1 .99E-06 | 5 .65E-06 |
| DCK | 2510 .035974 | 0 . 124159767 | 0 . 1 1737 | 1 .05784 | 0 .29013 | 0 .3434 |
| DCLK1 | 6484 .912156 | 0 .596032339 | 0 . 16101 | 3 .70183 | 0 .00021 | 0 .00045 |
| DCLK2 | 691 .3890933 | -0 .494247717 | 0 . 13102 | -3 .7723 | 0 .00016 | 0 .00034 |
| DCLK3 | 48 . 10299784 | -0 .328641639 | 0 .25146 | -1 .3069 | 0 . 19124 | 0 .23713 |
| DCLRE1A | 1304 .407495 | -0 .326864153 | 0 .08519 | -3 .8371 | 0 .00012 | 0 .00027 |
| DCLRE1B | 703 .9864002 | 0 .463718388 | 0 .0912 | 5 .08462 | 3 .68E-07 | 1 . 18E-06 |
| DCLRE1C | 1872 .030805 | 0 .284909335 | 0 .07996 | 3 .56312 | 0 .00037 | 0 .00074 |
| DCN | 72012 .32942 | 0 .20869155 | 0 .23352 | 0 .89366 | 0 .3715 | 0 .42743 |
| DCP1B | 496 .9393976 | -0 .552121438 | 0 .08978 | -6 . 1497 | 7 .76E-10 | 3 .58E-09 |
| DCP2 | 7818 .927422 | -0 . 126747981 | 0 . 1 1365 | -1 . 1 153 | 0 .26473 | 0 .31635 |
| DCPS | 636 .448709 | 0 . 194036165 | 0 . 14432 | 1 .34445 | 0 . 1788 | 0 .22322 |
| DCST1 | 50 .38047731 | -1 .03228848 | 0 .20847 | -4 .9518 | 7 .35E-07 | 2 .24E-06 |
| DCST2 | 70 .04053996 | -0 .928369054 | 0 .22233 | -4 . 1757 | 2 .97E-05 | 7 . 1 1E-05 |
| DCSTAMP | 106 .4718912 | 3 . 134730357 | 0 .50263 | 6 .23665 | 4 .47E-10 | 2 . 12E-09 |
| DCT | 127560 .4263 | -0 .730898817 | 0 .33123 | -2 .2066 | 0 .02734 | 0 .0403 |
| DCTD | 4391 .004764 | -0 .341285669 | 0 .09071 | -3 .7625 | 0 .00017 | 0 .00036 |
| DCTN1 | 6710 .312557 | 0 . 170338958 | 0 .08886 | 1 .91692 | 0 .05525 | 0 .07663 |
| DCTN2 | 7158 .885743 | -0 .062679475 | 0 .07119 | -0 .8805 | 0 .37861 | 0 .43477 |
| DCTN3 | 2895 .514617 | -0 .789527026 | 0 . 12655 | -6 .2391 | 4 .40E-10 | 2 .09E-09 |
| DCTN4 | 7565 .412388 | -0 .29554133 | 0 .06003 | -4 .9228 | 8 .53E-07 | 2 .57E-06 |
| DCTN5 | 6588 .486617 | 0 . 187578829 | 0 .08209 | 2 .28502 | 0 .02231 | 0 .03341 |
| DCTN6 | 1488 .725919 | -0 .491699602 | 0 . 1 1636 | -4 .2256 | 2 .38E-05 | 5 .80E-05 |
| DCTPP1 | 872 .8549443 | 0 .311376982 | 0 . 13031 | 2 .38943 | 0 .01687 | 0 .02578 |
| DCUN1D1 | 5802 .34337 | -0 .382868147 | 0 .08723 | -4 .3889 | 1 . 14E-05 | 2 .91E-05 |
| DCUN1D2 | 932 .4916792 | 0 . 190159736 | 0 .08245 | 2 .30633 | 0 .02109 | 0 .03172 |
| DCUN1D3 | 1886 .405718 | 0 .027701377 | 0 .08232 | 0 .33651 | 0 .73648 | 0 .77377 |
| DCUN1D4 | 4129 . 100934 | -0 .297825763 | 0 . 10465 | -2 .8458 | 0 .00443 | 0 .00752 |
| DCUN1D5 | 1911 .851586 | 0 .321489123 | 0 .0929 | 3 .46059 | 0 .00054 | 0 .00106 |
| DCX | 470 .0481841 | -3 .979327189 | 0 .40141 | -9 .9133 | 3 .64E-23 | 7 .82E-22 |

| DCXR | 1670 .477309 | 0 . 193834566 | 0 . 16668 | 1 . 16291 | 0 .24486 | 0 .29533 |
| --- | --- | --- | --- | --- | --- | --- |
| DDA1 | 920 .5765783 | 0 .559166906 | 0 . 1 1 187 | 4 .99833 | 5 .78E-07 | 1 .79E-06 |
| DDAH1 | 1 147 .883177 | 0 .439128797 | 0 . 18783 | 2 .33793 | 0 .01939 | 0 .02934 |
| DDAH2 | 2156 .479267 | 0 .342987107 | 0 . 12014 | 2 .855 | 0 .0043 | 0 .00732 |
| DDB1 | 23899 .8447 | -0 . 156551517 | 0 .09568 | -1 .6363 | 0 . 10178 | 0 . 13384 |
| DDB2 | 2627 .468691 | -0 .643012744 | 0 .09815 | -6 .5515 | 5 .70E-1 1 | 3 .03E-10 |
| DDC | 42 .22767162 | 0 .839223757 | 0 .64085 | 1 .30954 | 0 . 19035 | 0 .23611 |
| DDHD1 | 3422 .940786 | 0 .010762688 | 0 . 10831 | 0 .09937 | 0 .92085 | 0 .93419 |
| DDHD2 | 6237 .527771 | -0 .936796518 | 0 .09895 | -9 .4676 | 2 .86E-21 | 5 .01E-20 |
| DDI1 | 82 . 17967428 | -1 .960364491 | 0 .31353 | -6 .2526 | 4 .04E-10 | 1 .93E-09 |
| DDI2 | 3819 .629778 | 0 .324056206 | 0 .09911 | 3 .26953 | 0 .00108 | 0 .00202 |
| DDIAS | 449 .2802534 | 0 .953481102 | 0 . 14841 | 6 .42443 | 1 .32E-10 | 6 .70E-10 |
| DDIT3 | 939 .7908747 | -0 . 123201241 | 0 . 14458 | -0 .8521 | 0 .39415 | 0 .45073 |
| DDIT4 | 1844 .457036 | 0 .332402434 | 0 . 13641 | 2 .43671 | 0 .01482 | 0 .02285 |
| DDIT4L | 209 .5357985 | 0 .420978796 | 0 .28818 | 1 .46081 | 0 . 14407 | 0 . 18333 |
| DDN | 20 .81434371 | 0 .941261882 | 0 .27964 | 3 .36594 | 0 .00076 | 0 .00147 |
| DDO | 127 .4066217 | 1 .401610224 | 0 .29347 | 4 .77602 | 1 .79E-06 | 5 . 1 1E-06 |
| DDOST | 10520 .05084 | 0 .593673694 | 0 . 12194 | 4 .8687 | 1 . 12E-06 | 3 .33E-06 |
| DDR1 | 5070 . 1 15248 | 0 .227273655 | 0 . 12426 | 1 .82896 | 0 .06741 | 0 .09194 |
| DDR2 | 13991 .90167 | -0 .423284226 | 0 . 1 1541 | -3 .6677 | 0 .00024 | 0 .00051 |
| DDRGK1 | 1561 .007787 | 0 .352050038 | 0 . 14678 | 2 .39841 | 0 .01647 | 0 .02519 |
| DDT | 623 .5910764 | 0 .271456012 | 0 . 19152 | 1 .41734 | 0 . 15638 | 0 . 19749 |
| DDTL | 83 .68488432 | -0 . 135310993 | 0 . 12888 | -1 .0499 | 0 .29378 | 0 .34728 |
| DDX1 | 7000 .078389 | -0 . 122774631 | 0 .06636 | -1 .85 | 0 .06431 | 0 .08804 |
| DDX10 | 1793 .465049 | -0 .328183619 | 0 .07144 | -4 .5935 | 4 .36E-06 | 1 . 18E-05 |
| DDX11 | 1332 .836831 | -0 . 181544098 | 0 . 17138 | -1 .0593 | 0 .28946 | 0 .34267 |
| DDX17 | 49844 .82772 | -0 .228271374 | 0 .0859 | -2 .6573 | 0 .00788 | 0 .01279 |
| DDX18 | 6128 .286418 | 0 .029876209 | 0 .07535 | 0 .39648 | 0 .69175 | 0 .73416 |
| DDX19A | 2446 .638094 | 0 .447782132 | 0 .08504 | 5 .26541 | 1 .40E-07 | 4 .75E-07 |
| DDX19B | 996 .5917409 | -0 .011581654 | 0 .07 | -0 . 1655 | 0 .86858 | 0 .88944 |
| DDX20 | 2523 .347885 | -0 . 144992877 | 0 .07641 | -1 .8976 | 0 .05775 | 0 .07978 |
| DDX21 | 8721 . 124 | 0 .294440779 | 0 .09577 | 3 .07439 | 0 .00211 | 0 .00377 |
| DDX23 | 5012 . 19333 | 0 . 152033106 | 0 .07284 | 2 .08709 | 0 .03688 | 0 .05292 |
| DDX24 | 8377 .450711 | 0 .050014198 | 0 .08101 | 0 .61738 | 0 .53699 | 0 .59049 |
| DDX25 | 162 .7733233 | -0 .00871574 | 0 .28832 | -0 .0302 | 0 .97588 | 0 .97941 |
| DDX27 | 2381 .649006 | 0 .245603287 | 0 . 10682 | 2 .29914 | 0 .0215 | 0 .03228 |
| DDX28 | 1 105 .7 | 0 .377480815 | 0 . 1 1287 | 3 .3445 | 0 .00082 | 0 .00158 |
| DDX31 | 1595 .746088 | -0 .097518335 | 0 .07422 | -1 .3138 | 0 . 1889 | 0 .23456 |
| DDX39A | 2793 .279474 | 0 .785435736 | 0 . 104 | 7 .55256 | 4 .27E-14 | 3 .30E-13 |
| DDX39B | 2336 .324779 | 0 .263070839 | 0 . 12678 | 2 .07498 | 0 .03799 | 0 .05435 |
| DDX3X | 26033 .62246 | -0 .325000312 | 0 .07561 | -4 .2983 | 1 .72E-05 | 4 .28E-05 |
| DDX3Y | 2410 . 178821 | 1 1 . 10063598 | 0 .73929 | 15 .0153 | 5 .83E-51 | 1 .96E-48 |
| DDX4 | 17 . 16900555 | -3 .503385373 | 0 .51551 | -6 .796 | 1 .08E-1 1 | 6 .24E-1 1 |
| DDX41 | 2305 . 144062 | 0 . 10560317 | 0 .08947 | 1 . 18037 | 0 .23785 | 0 .28781 |
| DDX42 | 6173 .843524 | -0 .271130886 | 0 .09546 | -2 .8403 | 0 .00451 | 0 .00764 |
| DDX43 | 151 .5800072 | -0 .868757708 | 0 .37962 | -2 .2885 | 0 .02211 | 0 .03313 |
| DDX46 | 4135 .472838 | -0 . 14438536 | 0 .06738 | -2 . 1427 | 0 .03214 | 0 .04665 |
| DDX47 | 217 .0644412 | -0 .805149656 | 0 . 13098 | -6 . 1471 | 7 .89E-10 | 3 .64E-09 |
| DDX49 | 2657 .674906 | 0 .004380646 | 0 .09683 | 0 .04524 | 0 .96391 | 0 .96937 |
| DDX5 | 45065 .0214 | 0 . 100703964 | 0 . 10781 | 0 .93409 | 0 .35026 | 0 .40569 |
| DDX50 | 3618 . 153184 | -0 .851719387 | 0 .0895 | -9 .5164 | 1 .79E-21 | 3 .20E-20 |
| DDX51 | 944 .0624727 | 0 .507738325 | 0 .07662 | 6 .62705 | 3 .42E-1 1 | 1 .88E-10 |
| DDX53 | 7 . 151498618 | -2 .291859047 | 0 .58065 | -3 .947 | 7 .91E-05 | 0 .00018 |
| DDX54 | 1836 .799717 | 0 .484350627 | 0 .09528 | 5 .08339 | 3 .71E-07 | 1 . 18E-06 |
| DDX55 | 1630 .035851 | -0 .070205708 | 0 .07839 | -0 .8956 | 0 .37044 | 0 .42636 |
| DDX56 | 3564 .3679 | 0 . 135423618 | 0 .0934 | 1 .44986 | 0 . 1471 | 0 . 18688 |

| DDX58 | 3609 .701636 0 .008497061 | 0 . 13449 0 .06318 | 0 .94962 | 0 .95768 |
| --- | --- | --- | --- | --- |
| DDX59 | 2170 .824894 -0 .281302712 | 0 .07224 -3 .8943 | 9 .85E-05 | 0 .00022 |
| DDX6 | 21736 .00349 -0 .091515528 | 0 .07122 -1 .2849 | 0 . 19883 | 0 .24543 |
| DDX60 | 7308 .075702 0 . 181877719 | 0 . 16521 1 . 10091 | 0 .27093 | 0 .32304 |
| DDX60L | 3865 .595578 0 .758505931 | 0 . 14375 5 .27651 | 1 .32E-07 | 4 .49E-07 |
| DEAF1 | 1 190 .20064 0 .585680371 | 0 . 1 1718 4 .99813 | 5 .79E-07 | 1 .79E-06 |
| 1-Dec | 52 .38911494 -0 .944944733 | 0 .34157 -2 .7665 | 0 .00567 | 0 .00945 |
| DECR1 | 3788 .492524 -0 .097311821 | 0 . 10262 -0 .9483 | 0 .34298 | 0 .39849 |
| DECR2 | 323 .2284032 0 .97519818 | 0 . 12047 8 .09522 | 5 .72E-16 | 5 .50E-15 |
| DEDD | 972 .6953065 0 .026498108 | 0 .07291 0 .36345 | 0 .71627 | 0 .75645 |
| DEDD2 | 1098 .00825 0 .720087083 | 0 . 10096 7 . 13257 | 9 .85E-13 | 6 .52E-12 |
| DEF6 | 1403 .39541 0 .33019948 | 0 . 14603 2 .26119 | 0 .02375 | 0 .03539 |
| DEF8 | 3807 .806092 0 . 155699388 | 0 .08886 1 .75209 | 0 .07976 | 0 . 10724 |
| DEFA1 | 0 .071079971 -0 . 190628184 | 3 .0711 1 -0 .0621 | 0 .95051 | 0 .95839 |
| DEFA3 | 2 .494851661 -0 .542221191 | 0 .90623 -0 .5983 | 0 .54962 | 0 .60231 |
| DEFA4 | 1 .3918157 -3 .079309263 | 0 .89568 -3 .4379 | 0 .00059 | 0 .00115 |
| DEFA5 | 2 . 129180274 -2 .667494172 | 0 .87965 -3 .0324 | 0 .00243 | 0 .0043 |
| DEFA6 | 2 .742027438 -2 .567064817 | 0 .78623 -3 .265 | 0 .00109 | 0 .00205 |
| DEFB1 | 742 .2042603 -0 .845147743 | 0 .32257 -2 .62 | 0 .00879 | 0 .01414 |
| DEFB103A | 0 .586902113 0 .896894587 | 0 .9662 0 .92827 | 0 .35327 | 0 .40883 |
| DEFB103B | 0 .41605651 0 .798827163 | 1 . 19406 0 .669 | 0 .5035 | 0 .55827 |
| DEFB104A | 0 .256326307 -1 .871059918 | 1 .48631 -1 .2589 | 0 .20808 | 0 .25536 |
| DEFB104B | 0 . 167907201 -1 . 16499822 | 2 .02782 -0 .5745 | 0 .56562 | 0 .61776 |
| DEFB105A | 0 .240741736 -1 .506217996 | 1 .7883 -0 .8423 | 0 .39964 | 0 .45625 |
| DEFB106A | 0 .275666051 -1 .949064189 | 2 . 16341 -0 .9009 | 0 .36763 | 0 .42351 |
| DEFB106B | 0 .392571606 -2 .763999461 | 1 .57011 -1 .7604 | 0 .07834 | 0 . 10548 |
| DEFB107A | 0 .358945333 -2 .494441775 | 1 .34379 -1 .8563 | 0 .06341 | 0 .0869 |
| DEFB107B | 0 .296206231 -2 . 124209541 | 1 .611 16 -1 .3184 | 0 . 18736 | 0 .23278 |
| DEFB108B | 13 .74655099 -1 .285980412 | 0 .32261 -3 .9862 | 6 .71E-05 | 0 .00015 |
| DEFB110 | 1 .794089798 -4 .431871876 | 1 . 18085 -3 .7531 | 0 .00017 | 0 .00037 |
| DEFB112 | 0 .849337678 -3 .723020023 | 1 . 14196 -3 .2602 | 0 .0011 1 | 0 .00209 |
| DEFB113 | 1 .874180412 -4 .696167576 | 1 .00236 -4 .6851 | 2 .80E-06 | 7 .79E-06 |
| DEFB114 | 0 .933875813 -4 .018117525 | 1 .28717 -3 . 1217 | 0 .0018 | 0 .00325 |
| DEFB115 | 1 .800998793 -4 .572709578 | 1 .04776 -4 .3643 | 1 .28E-05 | 3 .23E-05 |
| DEFB116 | 1 .213519663 -4 . 189282099 | 1 .08427 -3 .8637 | 0 .00011 | 0 .00024 |
| DEFB118 | 3 . 17539443 -4 .743724289 | 0 .77908 -6 .0889 | 1 . 14E-09 | 5 . 13E-09 |
| DEFB119 | 2 .044786593 -4 .448144666 | 0 .97486 -4 .5628 | 5 .05E-06 | 1 .35E-05 |
| DEFB121 | 2 .235828749 -4 .963555437 | 1 .02262 -4 .8538 | 1 .21E-06 | 3 .56E-06 |
| DEFB123 | 1 .655843534 -4 .572894258 | 0 .97072 -4 .7108 | 2 .47E-06 | 6 .91E-06 |
| DEFB124 | 4 .527474526 0 .709657609 | 0 .49913 1 .42178 | 0 . 15509 | 0 . 19608 |
| DEFB125 | 5 . 1 1237721 -5 .876476188 | 0 .91303 -6 .4363 | 1 .22E-10 | 6 .23E-10 |
| DEFB126 | 4 .367459392 -1 . 121514177 | 0 .83914 -1 .3365 | 0 . 18139 | 0 .22611 |
| DEFB127 | 1 .963330878 -4 .893928282 | 1 .28782 -3 .8002 | 0 .00014 | 0 .00031 |
| DEFB128 | 1 .772259229 -4 .665998945 | 1 .02808 -4 .5386 | 5 .66E-06 | 1 .51E-05 |
| DEFB129 | 2 .862501134 -4 .574062147 | 1 . 14317 -4 .0012 | 6 .30E-05 | 0 .00014 |
| DEFB130 | 0 . 143023967 -1 . 164992363 | 2 .64701 -0 .4401 | 0 .65985 | 0 .70529 |
| DEFB131 | 1 .054852314 -3 .058909206 | 1 .01787 -3 .0052 | 0 .00265 | 0 .00467 |
| DEFB132 | 7 .053034476 -2 .232152008 | 0 .86946 -2 .5673 | 0 .01025 | 0 .01626 |
| DEFB133 | 0 .882323098 -3 .786266227 | 1 .22774 -3 .0839 | 0 .00204 | 0 .00366 |
| DEFB134 | 19 .3677151 0 .005071491 | 0 .39791 0 .01275 | 0 .98983 | 0 .99133 |
| DEFB135 | 6 . 187883104 0 .232373966 | 0 .49241 0 .47191 | 0 .63699 | 0 .68441 |
| DEFB136 | 2 . 109177023 -0 .822989547 | 0 .72193 -1 . 14 | 0 .25429 | 0 .30534 |
| DEFB4A | 654 .9608192 4 .997905807 | 0 .70765 7 .06272 | 1 .63E-12 | 1 .06E-1 1 |
| DEFB4B | 3 .29761373 1 . 12679157 | 0 .76008 1 .48247 | 0 . 13822 | 0 . 17669 |
| DEGS1 | 15504 .74852 -0 .497367899 | 0 . 13828 -3 .5969 | 0 .00032 | 0 .00066 |
| DEGS2 | 1 191 .50749 -0 .712850841 | 0 .2821 -2 .527 | 0 .01151 | 0 .01808 |

| DEK | 10728 .85434 | 0 .20620881 | 0 . 10852 | 1 .9002 | 0 .05741 | 0 .07934 |
| --- | --- | --- | --- | --- | --- | --- |
| DENND1A | 2131 .314847 | -1 .224333538 | 0 . 13999 | -8 .7458 | 2 .21E-18 | 2 .82E-17 |
| DENND1B | 4129 .633711 | -0 .087074539 | 0 . 12636 | -0 .6891 | 0 .49076 | 0 .54582 |
| DENND1C | 468 .7634939 | 0 . 194216852 | 0 . 16708 | 1 . 16239 | 0 .24508 | 0 .29555 |
| DENND2A | 418 .5219877 | -0 .356628022 | 0 .22318 | -1 .5979 | 0 . 1 1006 | 0 . 14376 |
| DENND2C | 3886 .716095 | -1 . 191589834 | 0 . 18754 | -6 .3537 | 2 . 10E-10 | 1 .04E-09 |
| DENND2D | 2268 .04534 | 0 .512685804 | 0 . 15022 | 3 .41281 | 0 .00064 | 0 .00125 |
| DENND3 | 1451 .716676 | 0 .990391555 | 0 . 1 1574 | 8 .55701 | 1 . 16E-17 | 1 .36E-16 |
| DENND4A | 5055 .822369 | -0 .280480058 | 0 .07163 | -3 .9155 | 9 .02E-05 | 0 .0002 |
| DENND4B | 1744 .27212 | 0 .874812697 | 0 . 10325 | 8 .47251 | 2 .40E-17 | 2 .72E-16 |
| DENND4C | 12753 .23253 | -0 .697674819 | 0 . 10779 | -6 .4727 | 9 .62E-1 1 | 4 .95E-10 |
| DENND5A | 5842 .653085 | 0 .640983373 | 0 .07571 | 8 .46583 | 2 .54E-17 | 2 .86E-16 |
| DENND5B | 3266 .926379 | -0 .697632003 | 0 . 12602 | -5 .5358 | 3 . 10E-08 | 1 . 16E-07 |
| DENND6A | 4569 .004505 | -0 .066435664 | 0 .08686 | -0 .7648 | 0 .44438 | 0 .50029 |
| DENND6B | 224 .9303588 | 0 .485659382 | 0 . 1 1524 | 4 .21443 | 2 .50E-05 | 6 .08E-05 |
| DENR | 2800 .374607 | 0 .261295074 | 0 .06598 | 3 .96046 | 7 .48E-05 | 0 .00017 |
| DEPDC1 | 931 .8282794 | 1 .411369512 | 0 .22795 | 6 . 19148 | 5 .96E-10 | 2 .79E-09 |
| DEPDC1B | 384 . 1671808 | 1 . 129548562 | 0 . 16717 | 6 .75696 | 1 .41E-1 1 | 8 .06E-1 1 |
| DEPDC4 | 88 . 1 1776677 | -1 .01193776 | 0 . 1 1 129 | -9 .0931 | 9 .62E-20 | 1 .44E-18 |
| DEPDC5 | 1084 .39619 | -0 .014711336 | 0 .08846 | -0 . 1663 | 0 .86792 | 0 .88896 |
| DEPDC7 | 1020 .542626 | -2 . 136394394 | 0 . 16327 | -13 .085 | 4 .03E-39 | 4 .22E-37 |
| DEPTOR | 1537 .002179 | -0 .290064787 | 0 . 15643 | -1 .8543 | 0 .0637 | 0 .08725 |
| DERA | 5034 .220449 | -0 . 161714235 | 0 . 14633 | -1 . 1051 | 0 .2691 | 0 .32112 |
| DERL1 | 4087 .395344 | 0 .505381063 | 0 .07132 | 7 .08593 | 1 .38E-12 | 9 .01E-12 |
| DERL2 | 2864 .470776 | 0 .087413918 | 0 .05907 | 1 .47985 | 0 . 13891 | 0 . 17746 |
| DERL3 | 313 .9107854 | 2 .297093917 | 0 .25822 | 8 .89576 | 5 .80E-19 | 7 .89E-18 |
| DES | 1255 .505907 | 3 .656963565 | 0 .48933 | 7 .47341 | 7 .81E-14 | 5 .80E-13 |
| DESI1 | 2748 . 179571 | 0 .413927827 | 0 .06814 | 6 .07441 | 1 .24E-09 | 5 .57E-09 |
| DESI2 | 6764 .899719 | 0 . 183994944 | 0 . 10674 | 1 .72384 | 0 .08474 | 0 . 1 1338 |
| DET1 | 642 .7260742 | -0 .613724738 | 0 . 10055 | -6 . 1035 | 1 .04E-09 | 4 .71E-09 |
| DEXI | 31 .36914842 | 0 .658986373 | 0 . 19513 | 3 .37711 | 0 .00073 | 0 .00141 |
| DFFA | 3249 . 187204 | 0 .40610344 | 0 .08243 | 4 .92663 | 8 .37E-07 | 2 .53E-06 |
| DFFB | 490 .6822387 | 0 .759606195 | 0 .09899 | 7 .6732 | 1 .68E-14 | 1 .35E-13 |
| DFNA5 | 1018 . 1 15728 | 2 .030723775 | 0 .21788 | 9 .32025 | 1 . 16E-20 | 1 .92E-19 |
| DFNB59 | 157 .3853307 | -0 .717267705 | 0 . 13672 | -5 .2461 | 1 .55E-07 | 5 .22E-07 |
| DGAT1 | 927 .3675896 | 0 .878370479 | 0 .09404 | 9 .34048 | 9 .59E-21 | 1 .60E-19 |
| DGAT2 | 8496 .032002 | 0 .308503594 | 0 .35724 | 0 .86358 | 0 .38782 | 0 .44415 |
| DGAT2L6 | 1419 .979408 | 1 .503792542 | 0 .69619 | 2 . 16004 | 0 .03077 | 0 .04485 |
| DGCR14 | 892 .9119065 | -0 . 179683058 | 0 .09143 | -1 .9652 | 0 .0494 | 0 .06922 |
| DGCR2 | 6384 .915726 | 0 .218461576 | 0 .08598 | 2 .5407 | 0 .01106 | 0 .01744 |
| DGCR6 | 869 . 1395723 | 0 . 180660861 | 0 . 15434 | 1 . 17055 | 0 .24178 | 0 .29216 |
| DGCR6L | 2560 .290118 | -0 .220625316 | 0 . 1 1666 | -1 .8911 | 0 .05861 | 0 .08087 |
| DGCR8 | 1700 . 172521 | 0 . 104115914 | 0 .09491 | 1 .09703 | 0 .27263 | 0 .32481 |
| DGKA | 2344 .762456 | -0 .683260399 | 0 . 16055 | -4 .2558 | 2 .08E-05 | 5 . 12E-05 |
| DGKB | 79 .69160127 | -2 .043137227 | 0 .32611 | -6 .2651 | 3 .73E-10 | 1 .79E-09 |
| DGKD | 1575 . 152899 | 1 .034083494 | 0 .0996 | 10 .3822 | 2 .99E-25 | 8 .05E-24 |
| DGKE | 1038 . 199673 | 0 .594657664 | 0 . 14442 | 4 . 1 1759 | 3 .83E-05 | 9 .00E-05 |
| DGKG | 451 .2675647 | -0 . 199947605 | 0 . 14816 | -1 .3495 | 0 . 17717 | 0 .22133 |
| DGKH | 4672 .473989 | 0 .49710436 | 0 . 12163 | 4 .08714 | 4 .37E-05 | 0 .0001 |
| DGKI | 2073 .558507 | -2 .009182299 | 0 .31814 | -6 .3154 | 2 .69E-10 | 1 .31E-09 |
| DGKQ | 1412 .272204 | 1 .06622568 | 0 . 10066 | 10 .5922 | 3 .24E-26 | 9 .68E-25 |
| DGKZ | 2404 .284988 | 1 . 199121539 | 0 .08807 | 13 .6149 | 3 .27E-42 | 4 .64E-40 |
| DGUOK | 1440 .957271 | -0 .036760455 | 0 . 1 1506 | -0 .3195 | 0 .74936 | 0 .78496 |
| DHCR24 | 18407 .21847 | -0 .499551062 | 0 .25962 | -1 .9242 | 0 .05433 | 0 .07549 |
| DHCR7 | 2539 .667694 | 0 .431417812 | 0 .28698 | 1 .50332 | 0 . 13276 | 0 . 17025 |
| DHDDS | 2165 .011742 | 0 .280800685 | 0 .0695 | 4 .04055 | 5 .33E-05 | 0 .00012 |

| DHDH | 35 .39997666 | 1 .340183485 | 0 .29173 | 4 .59393 | 4 .35E-06 | 1 . 18E-05 |
| --- | --- | --- | --- | --- | --- | --- |
| DHFR | 1 1 13 .514219 | 0 .958881137 | 0 . 12288 | 7 .80311 | 6 .04E-15 | 5 . 1 1E-14 |
| DHFRL1 | 1237 .530038 | -0 .641277424 | 0 .09282 | -6 .9089 | 4 .89E-12 | 2 .97E-1 1 |
| DHH | 30 .34465324 | 1 .209813177 | 0 . 19838 | 6 .09855 | 1 .07E-09 | 4 .85E-09 |
| DHODH | 575 .6687605 | 0 .087011 178 | 0 .09547 | 0 .91142 | 0 .36207 | 0 .41783 |
| DHPS | 1666 .44532 | -0 .499201913 | 0 .09239 | -5 .4034 | 6 .54E-08 | 2 .33E-07 |
| DHRS1 | 1385 .094157 | -0 .335897381 | 0 . 10461 | -3 .211 | 0 .00132 | 0 .00245 |
| DHRS12 | 684 .9397161 | -0 .732900759 | 0 . 1 1796 | -6 .2129 | 5 .20E-10 | 2 .45E-09 |
| DHRS13 | 185 .5126849 | 0 .489377354 | 0 . 1 1892 | 4 . 1 1505 | 3 .87E-05 | 9 . 10E-05 |
| DHRS2 | 1 16 . 142383 | 1 .589163046 | 0 .40945 | 3 .88123 | 0 .0001 | 0 .00023 |
| DHRS3 | 1791 .022269 | 0 .315564234 | 0 . 15746 | 2 .00406 | 0 .04506 | 0 .06367 |
| DHRS4 | 601 .2557536 | -0 .283701757 | 0 . 138 | -2 .0558 | 0 .0398 | 0 .05676 |
| DHRS4L2 | 760 .0122046 | -0 .337468883 | 0 . 12992 | -2 .5975 | 0 .00939 | 0 .01502 |
| DHRS7 | 4614 . 156194 | -0 .080134204 | 0 . 1 1562 | -0 .6931 | 0 .48827 | 0 .54336 |
| DHRS7B | 1859 .978315 | -0 .098542106 | 0 . 10278 | -0 .9587 | 0 .33769 | 0 .39289 |
| DHRS7C | 2 .912919988 | -3 .743990329 | 0 .80502 | -4 .6508 | 3 .31E-06 | 9 . 10E-06 |
| DHRS9 | 485 .5309268 | 0 .796588826 | 0 .2492 | 3 . 19656 | 0 .00139 | 0 .00256 |
| DHRSX | 868 .2783134 | 0 . 122063062 | 0 . 10245 | 1 . 19143 | 0 .23348 | 0 .28299 |
| DHTKD1 | 2240 .943683 | 0 .016178556 | 0 .08306 | 0 . 19479 | 0 .84556 | 0 .86972 |
| DHX15 | 16584 .62124 | -0 .290888507 | 0 . 10708 | -2 .7165 | 0 .0066 | 0 .01086 |
| DHX16 | 2302 .573549 | 0 .399858134 | 0 .08564 | 4 .66898 | 3 .03E-06 | 8 .37E-06 |
| DHX29 | 4006 .086997 | -0 . 155923058 | 0 .07293 | -2 . 1381 | 0 .03251 | 0 .04714 |
| DHX30 | 5280 .880644 | 0 .248232657 | 0 .07293 | 3 .4038 | 0 .00066 | 0 .00129 |
| DHX32 | 3507 .477336 | -0 .361381772 | 0 .08553 | -4 .2251 | 2 .39E-05 | 5 .81E-05 |
| DHX33 | 3086 .378408 | 1 . 185523718 | 0 . 1 1818 | 10 .0318 | 1 . 1 1E-23 | 2 .50E-22 |
| DHX34 | 862 .0761978 | 0 .437043957 | 0 .08628 | 5 .0655 | 4 .07E-07 | 1 .29E-06 |
| DHX35 | 1509 .840635 | -0 .047568334 | 0 .06912 | -0 .6882 | 0 .49134 | 0 .54636 |
| DHX36 | 8135 .540993 | -0 .049862983 | 0 .09102 | -0 .5478 | 0 .58383 | 0 .63497 |
| DHX37 | 1792 .365427 | 0 .723286172 | 0 .0884 | 8 . 18164 | 2 .80E-16 | 2 .80E-15 |
| DHX38 | 2989 .355126 | 0 . 148577856 | 0 .09456 | 1 .57119 | 0 . 1 1614 | 0 . 151 |
| DHX40 | 6387 .600181 | -0 .276850167 | 0 . 10322 | -2 .6822 | 0 .00731 | 0 .01195 |
| DHX57 | 2277 .889233 | -0 . 161547466 | 0 .06308 | -2 .561 | 0 .01044 | 0 .01653 |
| DHX58 | 1015 .727126 | 0 .377406768 | 0 . 12857 | 2 .93548 | 0 .00333 | 0 .00578 |
| DHX8 | 4848 .999911 | 0 . 157943925 | 0 .05742 | 2 .75067 | 0 .00595 | 0 .00988 |
| DHX9 | 16517 .83119 | 0 .019798027 | 0 .08272 | 0 .23934 | 0 .81084 | 0 .83928 |
| DIABLO | 519 .944167 | 0 .096392936 | 0 .08473 | 1 . 13762 | 0 .25528 | 0 .30641 |
| DIAPH1 | 6448 .237078 | 0 .689807658 | 0 .09671 | 7 . 1325 | 9 .86E-13 | 6 .52E-12 |
| DIAPH2 | 4526 .806501 | -0 .392494244 | 0 . 1 1088 | -3 .5398 | 0 .0004 | 0 .0008 |
| DIAPH3 | 615 .2205753 | 1 .078252172 | 0 . 16531 | 6 .5228 | 6 .90E-1 1 | 3 .62E-10 |
| DICER1 | 15380 .60752 | -0 . 133089578 | 0 .09092 | -1 .4638 | 0 . 14325 | 0 . 18238 |
| DIDO1 | 6287 .483139 | 0 . 1 1874196 | 0 .07574 | 1 .56785 | 0 . 1 1692 | 0 . 15185 |
| DIEXF | 2902 .934886 | -0 . 131403387 | 0 .07604 | -1 .7281 | 0 .08397 | 0 . 1 1243 |
| DIMT1 | 3525 .901494 | 0 .094581958 | 0 .09847 | 0 .96049 | 0 .33681 | 0 .39193 |
| DIO1 | 57 .64077782 | -0 .449451071 | 0 . 17284 | -2 .6003 | 0 .00931 | 0 .01491 |
| DIO2 | 9555 . 178394 | 0 .852126694 | 0 .20371 | 4 . 18295 | 2 .88E-05 | 6 .91E-05 |
| DIO3 | 62 .05810521 | 1 .304489022 | 0 .3297 | 3 .95661 | 7 .60E-05 | 0 .00017 |
| DIP2A | 3586 .982956 | 0 .050945316 | 0 .0982 | 0 .5188 | 0 .6039 | 0 .6539 |
| DIP2B | 13233 .47678 | -0 . 131472143 | 0 .06932 | -1 .8967 | 0 .05787 | 0 .07993 |
| DIP2C | 5217 .50118 | -0 .599737167 | 0 . 1 1959 | -5 .015 | 5 .30E-07 | 1 .65E-06 |
| DIRAS1 | 479 .007451 | 0 .899798211 | 0 .23636 | 3 .80697 | 0 .00014 | 0 .0003 |
| DIRAS2 | 71 .911 18551 | -0 .846618021 | 0 .29135 | -2 .9058 | 0 .00366 | 0 .00631 |
| DIRAS3 | 247 .21595 | -0 .719433783 | 0 .20116 | -3 .5765 | 0 .00035 | 0 .00071 |
| DIRC1 | 8 .789709268 | -0 .881804246 | 0 .43852 | -2 .0108 | 0 .04434 | 0 .06272 |
| DIRC2 | 2667 . 1 14279 | 0 .071852222 | 0 .08737 | 0 .82241 | 0 .41085 | 0 .4674 |
| DIRC3 | 150 .018725 | -0 .245042425 | 0 .20489 | -1 . 1959 | 0 .23172 | 0 .28117 |
| DIS3 | 6716 .55113 | -0 .05500257 | 0 .09172 | -0 .5997 | 0 .54872 | 0 .60152 |

2515 .551388 1822 .52072 2652 .54569 2375 .575011 81 .91914971 22 .01732521 3992 . 170735 2688 .914816

DIS3L DIS3L2 DISC1 DISP1 DISP2 DISP3 DIXDC1 DKC1

DKFZp779M010 . 16293688

| DKK1 DKK2 DKK3 DKK4 DKKL1 DLAT DLC1 DLD DLEC1 DLEU1 DLEU2L DLEU7 DLG1 DLG2 DLG3 DLG4 DLG5 DLGAP1 DLGAP2 DLGAP3 DLGAP4 DLGAP5 DLK1 DLK2 DLL1 DLL3 DLL4 DLST DLX1 DLX2 DLX3 DLX4 DLX5 DLX6 DMAP1 DMBT1 DMBX1 DMC1 DMD DMGDH DMKN DMP1 DMPK DMRT1 DMRT2 DMRT3 DMRTA1 | 329 .2808203 1520 . 167097 4121 .450207 15 .70239869 29 .70622283 3228 .416157 1 1498 .91936 3656 .309491 141 .637264 654 .5947952 86 .54930841 199 .7442161 5811 .996492 1237 .325002 1695 .679277 390 .0815046 2150 .347054 314 .3794176 78 .09831516 22 .37776169 1478 . 190235 854 .2811201 5 .813002621 175 .7338982 717 .5877374 252 .5150925 329 . 1304121 5033 .933639 75 . 1519198 17 .81769754 282 . 1695076 21 .56461183 1 1 1 .9138104 35 .57823112 1308 .384788 43 .29440138 16 .82205634 84 .28182071 1 1214 . 19222 200 . 1793916 40581 .99062 9 .898564407 454 .3506683 6 .627798478 64 .64747242 15 .3530726 542 .0693708 |
| --- | --- |

-0 .257294072

-0 . 133843495

0 .286980842

-2 .220738339

0 .448492367

-0 .582221574

-0 .88969705

0 . 107103476

0 . 182994797

2 .296185394

-0 .576348658

0 . 102777816

-1 .917337988

-0 .602232619

0 .025193425

-1 .515860031

0 .051803865

-0 .246859774

-0 .443407362

-0 .710824578

0 .722309863

-0 .529249975

-2 .019227501

-0 .460347915

0 .594016632

-0 .393263166

1 .488279418

-1 .958472835

0 .268176286

0 .659703847

1 .594235081

-0 .98308935

-0 .967076013

0 .019001665

2 .06878805

0 .866750517

0 . 100575512

2 . 187119543

1 .494053994

-0 .21203385

-0 .068339927

0 . 141024073

-0 .429265416

-0 .372849576

-0 .378455825

0 .482778779

-0 .890445236

-1 .7143277

-0 .699046106

-1 .436209812

-1 .845743609

0 .90410462

-4 .208081778

-0 . 164796634

0 .409627649

-0 .757940302

0 .07573 0 .08095 0 . 13013 0 . 14837 0 .21772 0 .39376 0 . 12022 0 .08931 0 .28891 0 .41865 0 .27494 0 . 16316 0 .2557 0 .27493 0 .08146 0 . 1748 0 . 10553 0 . 18873 0 . 1 1 12 0 . 16867 0 .205 0 .09403 0 .24713 0 . 12523 0 . 1 1027 0 . 16971 0 .26891 0 .26412 0 .20068 0 . 15439 0 . 17868 0 .66775 0 . 18218 0 . 15273 0 .3963 0 . 1482 0 .06222 0 .39488 0 .42293 0 .30781 0 .29629 0 .28836 0 .30078 0 .08979 0 .41969 0 .63366 0 . 18855 0 . 19865 0 . 18631 0 .24123 0 .67615 0 . 13149 0 .68208 0 .28484 0 .35218 0 .3621

-3 .3976 -1 .6534 2 .20539 -14 .968 2 .06 -1 .4786 -7 .4008 1 . 19922 0 .63341 5 .4847 -2 .0963 0 .62991 -7 .4983 -2 . 1905 0 .30926 -8 .6722 0 .49089 -1 .308 -3 .9875 -4 .2144 3 .52349 -5 .6283 -8 . 1708 -3 .6762 5 .38671 -2 .3173 5 .53441 -7 .415 1 .33636 4 .27294 8 .92219 -1 .4722 -5 .3082 0 . 12441 5 .22022 5 .84859 1 .61643 5 .53863 3 .53264 -0 .6889 -0 .2306 0 .48906 -1 .4272 -4 . 1526 -0 .9018 0 .76189 -4 .7227 -8 .6299 -3 .7521 -5 .9536 -2 .7298 6 .87572 -6 . 1695 -0 .5786 1 . 16313 -2 .0932

0 .00068 0 .09826 0 .02743 1 . 19E-50 0 .0394 0 . 13924 1 .35E-13 0 .23044 0 .52647 4 . 14E-08 0 .03606 0 .52875 6 .46E-14 0 .02849 0 .75712 4 .24E-18 0 .6235 0 . 19087 6 .68E-05 2 .50E-05 0 .00043 1 .82E-08 3 .06E-16 0 .00024 7 . 18E-08 0 .02049 3 . 12E-08 1 .22E-13 0 . 18143 1 .93E-05 4 .57E-19 0 . 14095 1 . 1 1E-07 0 .90099 1 .79E-07 4 .96E-09 0 . 106 3 .05E-08 0 .00041 0 .49092 0 .81759 0 .6248 0 . 15353 3 .29E-05 0 .36718 0 .44613 2 .33E-06 6 . 14E-18 0 .00018 2 .62E-09 0 .00634 6 . 17E-12 6 .85E-10 0 .56288 0 .24478 0 .03634

0 .00132 0 . 1296 0 .0404 3 .81E-48 0 .05622 0 . 17783 9 .84E-13 0 .27983 0 .58049 1 .52E-07 0 .05183 0 .58263 4 .87E-13 0 .0418 0 .79136 5 .23E-17 0 .6724 0 .23671 0 .00015 6 .08E-05 0 .00085 7 .01E-08 3 .05E-15 0 .00049 2 .55E-07 0 .03089 1 . 17E-07 8 .86E-13 0 .22615 4 .77E-05 6 .30E-18 0 . 17971 3 .82E-07 0 .91749 5 .95E-07 2 .06E-08 0 . 1389 1 . 14E-07 0 .00082 0 .54596 0 .8454 0 .67355 0 . 19426 7 .83E-05 0 .42302 0 .50197 6 .54E-06 7 .39E-17 0 .00037 1 . 13E-08 0 .01047 3 .69E-1 1 3 . 18E-09 0 .61519 0 .29524 0 .05219

| DMRTA2 DMRTB1 DMRTC1B DMRTC2 DMTF1 DMTN DMWD DMXL1 DMXL2 DNA2 DNAAF1 DNAAF2 DNAAF3 DNAAF5 DNAH1 DNAH10 | 4 .591814817 3 .502978847 0 .336242272 6 . 140662821 6542 .791275 258 . 1015603 806 .6235568 17922 .03435 10909 .02359 540 .5123885 30 .85377571 1041 .987664 25 .93741582 1024 .705391 1437 .90891 1 12 .3670259 |
| --- | --- |

DNAH10OS 65 .50929854

| DNAH11 DNAH12 DNAH14 DNAH17 DNAH2 DNAH3 DNAH5 DNAH6 DNAH7 DNAH8 DNAH9 DNAI1 DNAI2 DNAJA1 DNAJA2 DNAJA3 DNAJA4 DNAJB1 DNAJB11 DNAJB12 DNAJB13 DNAJB14 DNAJB2 DNAJB4 DNAJB5 DNAJB6 DNAJB7 DNAJB8 DNAJB9 DNAJC1 DNAJC10 DNAJC11 DNAJC12 DNAJC13 DNAJC14 DNAJC15 DNAJC16 DNAJC17 DNAJC18 | 381 .8127081 77 . 10896496 2279 .549196 558 .2806946 387 .27614 201 .4341871 133 . 1666701 486 .6644716 494 .9918673 152 .6966245 153 .2133607 26 . 15315284 9 .079645746 8150 .384444 7530 .739359 2996 . 173176 3262 .757353 5434 .804107 3101 . 107529 2449 .285032 10 .05892856 10271 .85072 3763 .504601 1741 .052372 489 .7770264 8345 .58876 54 .795769 6 .2134397 3165 .442903 2541 .02731 12531 .75108 1632 .836016 65 . 17698112 15563 .83669 1032 .03758 2790 .293041 2148 .853237 812 .6278335 923 .6146135 |
| --- | --- |

-0 .983835771

-2 . 141331336

-1 .591262072

-3 .534472075

-0 .621338435

0 .264028679

0 .728940397

-0 .409972603

0 .05006528

0 .291357914

-1 .34278226

-0 .023856074

0 .399904225

0 .644174553

-0 .218209309

-1 . 100532069

-0 . 169123118

-1 .969570712

-1 .80562136

-0 .759604994

0 .359105144

0 .431940609

-1 .350619311

-1 .024043728

-2 .532695597

-0 .673830538

-1 .936744262

-0 .598105763

-1 .437902179

-1 .461551784

0 .368116131

0 .008942013

0 .413773615

0 .273159396

0 . 136854819

0 .537744873

-0 . 154378166

-0 .757544031

-0 .224426722

-0 .391828742

-0 .468685168

0 .907102549

0 . 137562578

-0 .648893171

-2 .715199253

0 .384043895

0 . 168874702

-0 .061096425

0 .253220311

1 . 161514288

-0 .040485549

0 .654976496

0 .254745283

0 . 159993244

-0 .203888489

-0 .545064389

0 .61556 0 .88098 1 .62272 0 .76813 0 . 12083 0 . 19152 0 .09516 0 . 1 125 0 . 15617 0 . 10412 0 .28661 0 .09215 0 .23829 0 . 10923 0 . 15482 0 .20956 0 . 18773 0 .27452 0 .32043 0 . 18327 0 .27971 0 .38622 0 .21528 0 .34611 0 .24913 0 .24373 0 .37235 0 .2688 0 .27513 0 .36423 0 . 14718 0 . 1024 0 .08244 0 . 1598 0 . 14361 0 .09612 0 .06475 0 .34712 0 . 12514 0 .07496 0 . 12299 0 . 10118 0 .07954 0 . 1932 0 .57118 0 . 15353 0 . 1 1707 0 .09553 0 . 1 1732 0 .3531 0 . 10973 0 .09845 0 . 13944 0 .06822 0 .06328 0 . 10775

-1 .5983 -2 .4306 -0 .9806 -4 .6014 -5 . 1421 1 .37863 7 .65983 -3 .6443 0 .32058 2 .79831 -4 .685 -0 .2589 1 .6782 5 .89719 -1 .4095 -5 .2517 -0 .9009 -7 . 1745 -5 .6349 -4 . 1448 1 .28383 1 . 1 1838 -6 .2739 -2 .9587 -10 . 166 -2 .7646 -5 .2014 -2 .2251 -5 .2262 -4 .0128 2 .50113 0 .08732 5 .01921 1 .70934 0 .95296 5 .59456 -2 .3843 -2 . 1824 -1 .7934 -5 .2269 -3 .8109 8 .96563 1 .72952 -3 .3587 -4 .7537 2 .50145 1 .44257 -0 .6396 2 . 15836 3 .28944 -0 .3689 6 .6526 1 .82691 2 .34542 -3 .2218 -5 .0585

0 . 10998 0 .01507 0 .32678 4 .20E-06 2 .72E-07 0 . 16801 1 .86E-14 0 .00027 0 .74853 0 .00514 2 .80E-06 0 .79573 0 .09331 3 .70E-09 0 . 1587 1 .51E-07 0 .36766 7 .26E-13 1 .75E-08 3 .40E-05 0 . 1992 0 .26341 3 .52E-10 0 .00309 2 .81E-24 0 .0057 1 .98E-07 0 .02608 1 .73E-07 6 .00E-05 0 .01238 0 .93042 5 . 19E-07 0 .08739 0 .34061 2 .21E-08 0 .01711 0 .02908 0 .07291 1 .72E-07 0 .00014 3 .09E-19 0 .08372 0 .00078 2 .00E-06 0 .01237 0 . 14914 0 .52245 0 .0309 0 .001 0 .71217 2 .88E-1 1 0 .06771 0 .01901 0 .00127 4 .23E-07

0 . 14369 0 .0232 0 .38173 1 . 14E-05 8 .83E-07 0 .21102 1 .49E-13 0 .00055 0 .78426 0 .00862 7 .79E-06 0 .82592 0 . 12375 1 .55E-08 0 .2002 5 .08E-07 0 .42351 4 .86E-12 6 .77E-08 8 .08E-05 0 .24585 0 .3151 1 .69E-09 0 .00538 6 .84E-23 0 .0095 6 .54E-07 0 .03861 5 .77E-07 0 .00014 0 .01936 0 .9423 1 .61E-06 0 . 1 1664 0 .39606 8 .44E-08 0 .02611 0 .0426 0 .09881 5 .75E-07 0 .0003 4 .33E-18 0 . 1 1213 0 .0015 5 .67E-06 0 .01934 0 . 18916 0 .57664 0 .04503 0 .00189 0 .75286 1 .59E-10 0 .09232 0 .0288 0 .00237 1 .33E-06

| DNAJC19 | 2567 .095397 | -0 .979780882 | 0 .09754 | -10 .045 | 9 .64E-24 | 2 .21E-22 |
| --- | --- | --- | --- | --- | --- | --- |
| DNAJC2 | 1812 .503572 | -0 .006228138 | 0 . 1 1301 | -0 .0551 | 0 .95605 | 0 .96303 |
| DNAJC21 | 3434 .870275 | -0 . 128090064 | 0 .0836 | -1 .5322 | 0 . 12548 | 0 . 16183 |
| DNAJC22 | 395 . 1540764 | 1 .201020012 | 0 .23757 | 5 .0555 | 4 .29E-07 | 1 .35E-06 |
| DNAJC24 | 1887 .680369 | -0 .411949049 | 0 .07324 | -5 .625 | 1 .86E-08 | 7 . 14E-08 |
| DNAJC25 | 655 .3219558 | 0 . 122947346 | 0 . 1 1345 | 1 .08374 | 0 .27848 | 0 .33123 |
| DNAJC27 | 883 .3174296 | -0 .660158703 | 0 .07487 | -8 .8174 | 1 . 17E-18 | 1 .54E-17 |
| DNAJC28 | 125 .3871555 | -0 . 197511 136 | 0 . 10164 | -1 .9433 | 0 .05198 | 0 .07249 |
| DNAJC3 | 1 1418 .93692 | 0 .580195209 | 0 .09423 | 6 . 15695 | 7 .42E-10 | 3 .43E-09 |
| DNAJC30 | 266 . 1 166106 | 0 .41162872 | 0 . 1081 | 3 .8079 | 0 .00014 | 0 .0003 |
| DNAJC4 | 532 .0294118 | -0 .251426802 | 0 . 10886 | -2 .3095 | 0 .02091 | 0 .03149 |
| DNAJC5 | 6873 .337576 | 0 .865236075 | 0 .08592 | 10 .0706 | 7 .45E-24 | 1 .73E-22 |
| DNAJC5B | 47 .84533836 | 0 .951590116 | 0 .49326 | 1 .92918 | 0 .05371 | 0 .0747 |
| DNAJC5G | 5 .36174007 | -3 .740796285 | 0 .70192 | -5 .3293 | 9 .86E-08 | 3 .43E-07 |
| DNAJC6 | 510 .0704853 | 1 .01367101 | 0 .20736 | 4 .88848 | 1 .02E-06 | 3 .03E-06 |
| DNAJC7 | 3536 .072375 | -0 .312621966 | 0 .06467 | -4 .8343 | 1 .34E-06 | 3 .90E-06 |
| DNAJC8 | 3421 .22724 | -0 .08162579 | 0 . 10497 | -0 .7776 | 0 .43678 | 0 .49283 |
| DNAJC9 | 649 .7225185 | -0 .368588149 | 0 .06448 | -5 .7165 | 1 .09E-08 | 4 .32E-08 |
| DNAL1 | 1500 .269191 | 0 .037575767 | 0 . 10235 | 0 .36715 | 0 .71351 | 0 .75404 |
| DNAL4 | 1062 .329231 | -0 .350801254 | 0 . 10879 | -3 .2245 | 0 .00126 | 0 .00234 |
| DNALI1 | 202 .5534686 | -1 .602443744 | 0 . 19805 | -8 .0912 | 5 .91E-16 | 5 .67E-15 |
| DNASE1 | 781 .6584097 | -0 .098556083 | 0 . 12918 | -0 .7629 | 0 .44551 | 0 .50134 |
| DNASE1L1 | 661 .9340944 | 0 . 179842595 | 0 . 1 1689 | 1 .53859 | 0 . 1239 | 0 . 16002 |
| DNASE1L2 | 712 . 1980156 | 0 .327109756 | 0 .33442 | 0 .97813 | 0 .32801 | 0 .38295 |
| DNASE1L3 | 395 .3368981 | 0 .444623567 | 0 .20752 | 2 . 14259 | 0 .03215 | 0 .04666 |
| DNASE2 | 2479 .351185 | 0 .820874983 | 0 . 1 1011 | 7 .45496 | 8 .99E-14 | 6 .63E-13 |
| DNASE2B | 81 .30010897 | 1 .985684505 | 0 .4153 | 4 .78129 | 1 .74E-06 | 4 .99E-06 |
| DNER | 444 . 1503521 | 3 .018351002 | 0 .3629 | 8 .3172 | 9 .01E-17 | 9 .53E-16 |
| DNHD1 | 1609 .540646 | -0 .35247618 | 0 . 14782 | -2 .3845 | 0 .0171 | 0 .0261 |
| DNLZ | 44 .81797907 | -0 .025540278 | 0 . 17194 | -0 . 1485 | 0 .88191 | 0 .90088 |
| DNM1 | 1537 .313605 | -0 .545706752 | 0 . 18869 | -2 .8921 | 0 .00383 | 0 .00657 |
| DNM1L | 7853 .74793 | 0 .057636193 | 0 .07709 | 0 .74767 | 0 .45466 | 0 .51046 |
| DNM2 | 6794 .676651 | 0 .643234712 | 0 .07019 | 9 . 16413 | 4 .99E-20 | 7 .71E-19 |
| DNM3 | 812 . 1769005 | -0 .576531796 | 0 . 13422 | -4 .2954 | 1 .74E-05 | 4 .34E-05 |
| DNMBP | 1660 .875712 | -0 .59943677 | 0 . 1379 | -4 .347 | 1 .38E-05 | 3 .48E-05 |
| DNMT1 | 2870 .936721 | 0 .767808184 | 0 . 1 1 186 | 6 .86428 | 6 .68E-12 | 3 .98E-1 1 |
| DNMT3A | 2219 .30987 | 0 .404589741 | 0 . 1 1297 | 3 .58125 | 0 .00034 | 0 .00069 |
| DNMT3B | 422 . 1818955 | 0 . 19009462 | 0 . 12306 | 1 .54472 | 0 . 12241 | 0 . 15824 |
| DNMT3L | 4 .821758016 | -1 .531627512 | 0 .40918 | -3 .7431 | 0 .00018 | 0 .00038 |
| DNPEP | 2637 .510437 | 0 .425956045 | 0 .0705 | 6 .04153 | 1 .53E-09 | 6 .77E-09 |
| DNPH1 | 1970 .437236 | 0 .640673448 | 0 . 18195 | 3 .52113 | 0 .00043 | 0 .00086 |
| DNTT | 7 .485976596 | -2 .85969991 | 0 .64895 | -4 .4067 | 1 .05E-05 | 2 .69E-05 |
| DNTTIP1 | 765 .2254522 | 0 .510463723 | 0 . 1 1739 | 4 .34838 | 1 .37E-05 | 3 .46E-05 |
| DNTTIP2 | 3067 .508436 | -0 . 192198533 | 0 . 10159 | -1 .8919 | 0 .0585 | 0 .08074 |
| DOC2A | 27 .20147714 | -0 .922828259 | 0 . 19205 | -4 .8052 | 1 .55E-06 | 4 .46E-06 |
| DOC2B | 57 . 13969208 | 1 .268110668 | 0 .26859 | 4 .72129 | 2 .34E-06 | 6 .59E-06 |
| DOCK1 | 10399 .20788 | -0 .668821339 | 0 .0934 | -7 . 1611 | 8 .00E-13 | 5 .34E-12 |
| DOCK10 | 8091 .958008 | -0 .418691944 | 0 . 14317 | -2 .9245 | 0 .00345 | 0 .00597 |
| DOCK11 | 4046 .605854 | 0 .566129505 | 0 . 1 1555 | 4 .89962 | 9 .60E-07 | 2 .87E-06 |
| DOCK2 | 4324 .281356 | 1 .677868214 | 0 . 16596 | 10 . 1 103 | 4 .98E-24 | 1 . 18E-22 |
| DOCK3 | 339 .4129449 | -0 .741770099 | 0 . 18212 | -4 .0729 | 4 .64E-05 | 0 .00011 |
| DOCK4 | 3910 .315561 | 0 .225561199 | 0 . 10716 | 2 . 10495 | 0 .0353 | 0 .05082 |
| DOCK5 | 5808 .909139 | 0 . 14378198 | 0 . 1576 | 0 .91234 | 0 .36159 | 0 .41732 |
| DOCK6 | 1412 .311412 | 0 .491913277 | 0 . 15778 | 3 . 1 1774 | 0 .00182 | 0 .00329 |
| DOCK7 | 12000 .90354 | -0 .4854259 | 0 . 1 1883 | -4 .0851 | 4 .41E-05 | 0 .0001 |
| DOCK8 | 5504 .834254 | -0 . 142118215 | 0 . 1511 1 | -0 .9405 | 0 .34697 | 0 .40248 |

| DOCK9 | 8776 .281052 | -0 .071165966 | 0 . 14113 | -0 .5043 | 0 .61407 | 0 .66331 |
| --- | --- | --- | --- | --- | --- | --- |
| DOHH | 529 .0820452 | 0 .639139118 | 0 .09189 | 6 .95523 | 3 .52E-12 | 2 . 17E-1 1 |
| DOK1 | 467 .5290498 | 1 .358543303 | 0 . 12589 | 10 .7915 | 3 .77E-27 | 1 .25E-25 |
| DOK2 | 782 .2931818 | 1 .770541906 | 0 . 17761 | 9 .96894 | 2 .08E-23 | 4 .59E-22 |
| DOK3 | 367 .2052091 | 1 .83321171 | 0 . 15892 | 1 1 .5351 | 8 .77E-31 | 4 .04E-29 |
| DOK4 | 1559 .924239 | -0 .252278404 | 0 . 1 1922 | -2 . 1 161 | 0 .03434 | 0 .04954 |
| DOK5 | 198 .8941531 | -0 .925812807 | 0 .28306 | -3 .2707 | 0 .00107 | 0 .00201 |
| DOK6 | 469 .8940126 | 0 . 1 15141924 | 0 . 18834 | 0 .61134 | 0 .54097 | 0 .59422 |
| DOK7 | 66 .6543315 | -0 . 146814552 | 0 .22176 | -0 .662 | 0 .50794 | 0 .56238 |
| DOLK | 1049 .77411 | 0 .278569531 | 0 .09802 | 2 .84185 | 0 .00449 | 0 .00761 |
| DOLPP1 | 673 .6596424 | -0 . 1 18559392 | 0 .09225 | -1 .2852 | 0 . 19872 | 0 .24532 |
| DONSON | 829 .7261713 | 0 .508575671 | 0 . 10797 | 4 .71017 | 2 .48E-06 | 6 .93E-06 |
| DOPEY1 | 6577 .219252 | -1 .009109193 | 0 . 12566 | -8 .0304 | 9 .71E-16 | 9 .00E-15 |
| DOPEY2 | 2700 .392716 | 0 .960269941 | 0 . 12409 | 7 .73837 | 1 .01E-14 | 8 .31E-14 |
| DOT1L | 1078 .393932 | 0 .785977423 | 0 . 13563 | 5 .7949 | 6 .84E-09 | 2 .79E-08 |
| DPAGT1 | 774 .3974015 | 0 .502227239 | 0 .09777 | 5 . 13698 | 2 .79E-07 | 9 .06E-07 |
| DPCD | 701 .6618796 | -0 .273301194 | 0 . 12761 | -2 . 1417 | 0 .03222 | 0 .04676 |
| DPCR1 | 19 .67014183 | -3 .871314652 | 0 .48855 | -7 .924 | 2 .30E-15 | 2 .06E-14 |
| DPEP1 | 86 .65648854 | 3 .241458274 | 0 .47006 | 6 .89582 | 5 .36E-12 | 3 .24E-1 1 |
| DPEP2 | 220 .9427217 | 1 .028014307 | 0 .20252 | 5 .07612 | 3 .85E-07 | 1 .22E-06 |
| DPEP3 | 10 .55180977 | 0 .499921219 | 0 .42463 | 1 . 17732 | 0 .23907 | 0 .28916 |
| DPF1 | 34 .47716652 | 0 .539399218 | 0 .28822 | 1 .8715 | 0 .06128 | 0 .08422 |
| DPF2 | 1573 .918919 | 0 .03952291 | 0 .0817 | 0 .48376 | 0 .62855 | 0 .67694 |
| DPF3 | 124 .3587348 | -0 .684692394 | 0 .20597 | -3 .3242 | 0 .00089 | 0 .00169 |
| DPH1 | 796 .8050213 | 0 .017913401 | 0 .09626 | 0 . 18609 | 0 .85238 | 0 .87542 |
| DPH2 | 1419 .364573 | 0 .690324185 | 0 . 1319 | 5 .23362 | 1 .66E-07 | 5 .56E-07 |
| DPH3 | 2032 .473504 | 0 .011842231 | 0 . 1 1994 | 0 .09874 | 0 .92135 | 0 .93455 |
| DPH5 | 856 .3519286 | -1 . 190762066 | 0 . 10444 | -1 1 .401 | 4 . 13E-30 | 1 .80E-28 |
| DPH6 | 2118 .201105 | -0 .913965857 | 0 .09426 | -9 .6965 | 3 . 12E-22 | 6 .07E-21 |
| DPH7 | 1090 .288712 | 0 . 138747125 | 0 .07704 | 1 .80094 | 0 .07171 | 0 .09725 |
| DPM1 | 2727 .048481 | -0 .079169218 | 0 . 13347 | -0 .5932 | 0 .55308 | 0 .60551 |
| DPM2 | 1021 .272307 | -0 .272013172 | 0 . 12529 | -2 . 171 | 0 .02993 | 0 .04374 |
| DPM3 | 1 105 .543452 | 0 .228964324 | 0 . 16888 | 1 .35576 | 0 . 17518 | 0 .2191 |
| DPP10 | 140 .4067254 | 0 .217436932 | 0 .54542 | 0 .39866 | 0 .69014 | 0 .73257 |
| DPP3 | 1696 .5972 | 1 . 133973881 | 0 . 12097 | 9 .37372 | 7 .00E-21 | 1 . 18E-19 |
| DPP4 | 1724 .5827 | 0 .71836352 | 0 .21138 | 3 .39844 | 0 .00068 | 0 .00131 |
| DPP6 | 319 .7620855 | -0 .442480664 | 0 .38033 | -1 . 1634 | 0 .24466 | 0 .29512 |
| DPP7 | 4302 .946628 | 0 .588400016 | 0 . 13405 | 4 .38942 | 1 . 14E-05 | 2 .90E-05 |
| DPP8 | 6448 .448526 | 0 . 16215209 | 0 .0595 | 2 .72512 | 0 .00643 | 0 .0106 |
| DPP9 | 1483 .717525 | 1 .259772482 | 0 .09384 | 13 .4246 | 4 .34E-41 | 5 .51E-39 |
| DPPA2 | 5 .664507265 | -3 .408063988 | 0 .95532 | -3 .5674 | 0 .00036 | 0 .00073 |
| DPPA3 | 4 .618871996 | -3 .813789068 | 0 .64402 | -5 .9218 | 3 . 18E-09 | 1 .35E-08 |
| DPPA4 | 24 .58137227 | -0 .922966412 | 0 .42949 | -2 . 149 | 0 .03163 | 0 .046 |
| DPPA5 | 0 .97640542 | -2 .575729447 | 0 .84285 | -3 .056 | 0 .00224 | 0 .004 |
| DPRX | 2 .918942418 | -3 .404640937 | 0 .81747 | -4 . 1648 | 3 . 12E-05 | 7 .45E-05 |
| DPT | 4218 .827925 | -0 .682106195 | 0 .2472 | -2 .7593 | 0 .00579 | 0 .00965 |
| DPY19L1 | 3866 . 19959 | 0 .471149281 | 0 . 13212 | 3 .56609 | 0 .00036 | 0 .00073 |
| DPY19L2 | 2233 .08246 | -2 .077063449 | 0 .24783 | -8 .381 | 5 .25E-17 | 5 .69E-16 |
| DPY19L3 | 4019 .540534 | -0 .612785754 | 0 . 1 1749 | -5 .2156 | 1 .83E-07 | 6 .09E-07 |
| DPY19L4 | 5336 . 16356 | -0 .006779557 | 0 . 14227 | -0 .0477 | 0 .96199 | 0 .968 |
| DPY30 | 1617 .46308 | -0 .489317586 | 0 . 1 1878 | -4 . 1 196 | 3 .80E-05 | 8 .94E-05 |
| DPYD | 5632 .709307 | -0 .717217609 | 0 . 17041 | -4 .2087 | 2 .57E-05 | 6 .22E-05 |
| DPYS | 50 .6420511 1 | 0 .6093812 | 0 .38165 | 1 .59671 | 0 . 1 1033 | 0 . 14404 |
| DPYSL2 | 19923 .87035 | -0 .471187692 | 0 . 1 1661 | -4 .0408 | 5 .33E-05 | 0 .00012 |
| DPYSL3 | 7634 .060678 | -0 .987895953 | 0 . 14296 | -6 .9102 | 4 .84E-12 | 2 .94E-1 1 |
| DPYSL4 | 201 .6374457 | 0 .88693357 | 0 .23384 | 3 .79294 | 0 .00015 | 0 .00032 |

| DPYSL5 | 13 .21321491 | -2 .319053789 | 0 .55625 | -4 . 1691 | 3 .06E-05 | 7 .32E-05 |
| --- | --- | --- | --- | --- | --- | --- |
| DQX1 | 92 .60771455 | -1 .745459168 | 0 . 19457 | -8 .9708 | 2 .94E-19 | 4 . 15E-18 |
| DR1 | 9242 .698901 | -0 . 1 15415202 | 0 .07436 | -1 .5522 | 0 . 12062 | 0 . 15613 |
| DRAM1 | 1639 .80039 | 1 .470576051 | 0 . 16484 | 8 .92114 | 4 .62E-19 | 6 .35E-18 |
| DRAM2 | 3921 .573302 | -0 .482266269 | 0 . 10019 | -4 .8137 | 1 .48E-06 | 4 .29E-06 |
| DRAP1 | 2603 .743959 | 0 .759734552 | 0 . 15284 | 4 .97082 | 6 .67E-07 | 2 .04E-06 |
| DRAXIN | 96 .22409896 | 1 .464283415 | 0 .354 | 4 . 13634 | 3 .53E-05 | 8 .36E-05 |
| DRC1 | 18 . 17059811 | -1 .658818106 | 0 .35059 | -4 .7316 | 2 .23E-06 | 6 .28E-06 |
| DRC3 | 323 .6175499 | -0 .546741364 | 0 . 13135 | -4 . 1626 | 3 . 15E-05 | 7 .51E-05 |
| DRC7 | 1 1 .21415462 | -2 .62995561 | 0 .43332 | -6 .0693 | 1 .28E-09 | 5 .75E-09 |
| DRD1 | 23 .56205454 | -0 .240741972 | 0 .34239 | -0 .7031 | 0 .48198 | 0 .53723 |
| DRD2 | 15 .51960621 | -1 .444327975 | 0 .30929 | -4 .6698 | 3 .02E-06 | 8 .34E-06 |
| DRD3 | 6 .435544507 | -3 .238531447 | 0 .65633 | -4 .9343 | 8 .04E-07 | 2 .43E-06 |
| DRD4 | 12 .55768742 | 0 .590418855 | 0 .29861 | 1 .9772 | 0 .04802 | 0 .06745 |
| DRD5 | 10 .6184808 | -0 .974436423 | 0 .3186 | -3 .0585 | 0 .00222 | 0 .00397 |
| DRG1 | 2308 .487697 | -0 .305137886 | 0 . 1 1601 | -2 .6302 | 0 .00853 | 0 .01375 |
| DRG2 | 1903 . 152558 | -0 .069050994 | 0 .06306 | -1 .095 | 0 .27352 | 0 .32579 |
| DRGX | 26 .5126966 | -1 .415551632 | 0 .39905 | -3 .5473 | 0 .00039 | 0 .00078 |
| DRICH1 | 25 .390026 | -0 .971641774 | 0 .21062 | -4 .6131 | 3 .97E-06 | 1 .08E-05 |
| DROSHA | 3082 . 147721 | -0 .269094897 | 0 .07584 | -3 .5484 | 0 .00039 | 0 .00078 |
| DRP2 | 80 .4850883 | -1 . 123080657 | 0 .22585 | -4 .9726 | 6 .60E-07 | 2 .02E-06 |
| DSC1 | 32023 .69535 | -1 .038253126 | 0 .29711 | -3 .4945 | 0 .00047 | 0 .00094 |
| DSC2 | 10761 .08521 | 2 .428578684 | 0 .23708 | 10 .2438 | 1 .26E-24 | 3 . 19E-23 |
| DSC3 | 55101 .45464 | -0 .614540886 | 0 .2919 | -2 . 1053 | 0 .03526 | 0 .05078 |
| DSCAM | 28 .93038157 | -2 .399857126 | 0 .41334 | -5 .8061 | 6 .40E-09 | 2 .62E-08 |
| DSCAML1 | 39 .47883952 | -0 .541147107 | 0 .326 | -1 .66 | 0 .09692 | 0 . 12802 |
| DSCC1 | 556 .6298645 | 0 .434532502 | 0 . 15056 | 2 .88611 | 0 .0039 | 0 .00669 |
| DSCR3 | 5188 .452275 | 0 .247727317 | 0 .08231 | 3 .00967 | 0 .00262 | 0 .00461 |
| DSCR4 | 12 .76925261 | -0 .350318973 | 0 .90458 | -0 .3873 | 0 .69855 | 0 .74035 |
| DSCR8 | 65 .61317271 | 2 .656373838 | 0 .94728 | 2 .8042 | 0 .00504 | 0 .00848 |
| DSE | 5559 .437827 | 0 .041245271 | 0 . 12417 | 0 .33217 | 0 .73976 | 0 .77673 |
| DSEL | 1051 .423524 | -0 . 1 19709383 | 0 . 17526 | -0 .683 | 0 .49458 | 0 .54959 |
| DSG1 | 87811 . 13116 | -1 .053645035 | 0 .28392 | -3 .711 | 0 .00021 | 0 .00043 |
| DSG2 | 2260 . 180752 | 0 .74163373 | 0 .26735 | 2 .77403 | 0 .00554 | 0 .00925 |
| DSG3 | 1 1775 .94383 | 0 .94565635 | 0 .27006 | 3 .50171 | 0 .00046 | 0 .00092 |
| DSG4 | 1 13 .5480124 | -0 .010268521 | 0 .46457 | -0 .0221 | 0 .98237 | 0 .98498 |
| DSN1 | 1450 .810826 | 0 .698332834 | 0 . 12133 | 5 .75584 | 8 .62E-09 | 3 .47E-08 |
| DSP | 168448 .9579 | -0 .32880048 | 0 .29679 | -1 . 1078 | 0 .26793 | 0 .31981 |
| DSPP | 14 .50132882 | -2 .985334246 | 0 .56942 | -5 .2428 | 1 .58E-07 | 5 .31E-07 |
| DST | 88148 .75495 | -0 .362935752 | 0 . 12825 | -2 .83 | 0 .00466 | 0 .00787 |
| DSTN | 18198 .62011 | -0 .093676344 | 0 .09373 | -0 .9994 | 0 .31759 | 0 .37212 |
| DSTYK | 13070 .99246 | 0 .00665132 | 0 . 15099 | 0 .04405 | 0 .96486 | 0 .97012 |
| DTD1 | 2761 .980592 | -0 .254703698 | 0 . 1 1758 | -2 . 1662 | 0 .03029 | 0 .04423 |
| DTD2 | 1968 .342021 | 0 . 195226134 | 0 . 10316 | 1 .89248 | 0 .05843 | 0 .08065 |
| DTHD1 | 198 .7899745 | 1 .617604556 | 0 .29561 | 5 .47211 | 4 .45E-08 | 1 .62E-07 |
| DTL | 1 169 .401276 | 1 .613328836 | 0 . 18301 | 8 .81554 | 1 . 19E-18 | 1 .56E-17 |
| DTNA | 811 .9859095 | 0 .216331819 | 0 .20248 | 1 .06844 | 0 .28532 | 0 .33838 |
| DTNB | 644 .4388158 | 0 . 159538285 | 0 .09552 | 1 .67028 | 0 .09486 | 0 . 1256 |
| DTNBP1 | 1058 .287425 | 0 .553841699 | 0 .09087 | 6 .09499 | 1 .09E-09 | 4 .95E-09 |
| DTWD1 | 2299 .480097 | -0 .565680049 | 0 .09502 | -5 .9532 | 2 .63E-09 | 1 . 13E-08 |
| DTWD2 | 1307 .272189 | 0 .627046201 | 0 . 10395 | 6 .0323 | 1 .62E-09 | 7 . 14E-09 |
| DTX1 | 189 .2093426 | 1 .006924278 | 0 .23596 | 4 .26738 | 1 .98E-05 | 4 .88E-05 |
| DTX2 | 749 . 1413882 | 0 .346706397 | 0 . 1 1813 | 2 .93499 | 0 .00334 | 0 .00579 |
| DTX3 | 790 .9838593 | 0 . 138555031 | 0 . 10679 | 1 .29744 | 0 . 19448 | 0 .24069 |
| DTX3L | 8168 .983105 | 1 .644161061 | 0 . 15129 | 10 .8675 | 1 .65E-27 | 5 .67E-26 |
| DTX4 | 1918 .362893 | 1 .000663555 | 0 . 17377 | 5 .75857 | 8 .48E-09 | 3 .42E-08 |

| DTYMK | 1294 .757899 | 0 .924922303 | 0 . 13958 | 6 .62648 | 3 .44E-1 1 | 1 .89E-10 |
| --- | --- | --- | --- | --- | --- | --- |
| DUOX1 | 3716 .345243 | -0 .220666806 | 0 .22348 | -0 .9874 | 0 .32344 | 0 .37837 |
| DUOX2 | 173 .9879346 | -0 .696005469 | 0 .25042 | -2 .7794 | 0 .00545 | 0 .00911 |
| DUOXA1 | 1 122 .015979 | -0 . 136854078 | 0 .23396 | -0 .5849 | 0 .55859 | 0 .61105 |
| DUOXA2 | 34 .60866003 | -0 .461915406 | 0 .3094 | -1 .4929 | 0 . 13545 | 0 . 17345 |
| DUPD1 | 2 .401161016 | -0 .679064086 | 0 .49712 | -1 .366 | 0 . 17194 | 0 .2154 |
| DUS1L | 2957 .487313 | 0 .855558031 | 0 .06991 | 12 .2376 | 1 .96E-34 | 1 .33E-32 |
| DUS2 | 716 . 1474932 | 0 .018275193 | 0 .09003 | 0 .20299 | 0 .83914 | 0 .86405 |
| DUS3L | 1220 .441639 | 0 . 164580579 | 0 .0991 | 1 .6607 | 0 .09677 | 0 . 12784 |
| DUS4L | 729 .7532401 | -0 .424063868 | 0 . 10843 | -3 .911 1 | 9 . 19E-05 | 0 .0002 |
| DUSP1 | 7958 .643487 | 0 .505752638 | 0 .23655 | 2 . 13806 | 0 .03251 | 0 .04714 |
| DUSP10 | 1651 .725909 | 1 .513736802 | 0 . 17837 | 8 .48652 | 2 . 13E-17 | 2 .42E-16 |
| DUSP11 | 1622 .539687 | -0 .376650207 | 0 .05925 | -6 .3575 | 2 .05E-10 | 1 .02E-09 |
| DUSP12 | 1411 .212818 | -0 . 195623737 | 0 . 10974 | -1 .7826 | 0 .07465 | 0 . 10101 |
| DUSP13 | 23 .99676725 | -1 . 186589961 | 0 .33824 | -3 .5081 | 0 .00045 | 0 .0009 |
| DUSP15 | 170 .9969698 | 1 .083866102 | 0 .24584 | 4 .40885 | 1 .04E-05 | 2 .67E-05 |
| DUSP16 | 3181 .812788 | 0 .370167115 | 0 . 1 1938 | 3 . 10079 | 0 .00193 | 0 .00347 |
| DUSP18 | 342 .4390316 | 0 .277141881 | 0 .07452 | 3 .71926 | 0 .0002 | 0 .00042 |
| DUSP19 | 715 .9836422 | -0 . 105382149 | 0 . 14239 | -0 .7401 | 0 .45924 | 0 .515 |
| DUSP2 | 677 . 1046406 | 0 .742085609 | 0 . 18762 | 3 .95521 | 7 .65E-05 | 0 .00017 |
| DUSP21 | 1 .417372176 | -4 .045125063 | 0 .88561 | -4 .5676 | 4 .93E-06 | 1 .33E-05 |
| DUSP22 | 3937 . 129609 | -0 .202326194 | 0 .08899 | -2 .2735 | 0 .023 | 0 .03436 |
| DUSP23 | 1456 .222818 | 0 .582631997 | 0 . 15116 | 3 .85434 | 0 .00012 | 0 .00025 |
| DUSP26 | 5 .551202292 | -1 .382224225 | 0 .48973 | -2 .8224 | 0 .00477 | 0 .00805 |
| DUSP27 | 1 1 .85160273 | -1 .379930431 | 0 .4204 | -3 .2824 | 0 .00103 | 0 .00194 |
| DUSP28 | 327 .4359903 | -0 .023401898 | 0 .06593 | -0 .355 | 0 .72261 | 0 .76214 |
| DUSP3 | 7146 .20455 | 0 .671768543 | 0 .09774 | 6 .87315 | 6 .28E-12 | 3 .76E-1 1 |
| DUSP4 | 1 1851 .36026 | 1 .095784717 | 0 . 19949 | 5 .49296 | 3 .95E-08 | 1 .45E-07 |
| DUSP5 | 588 .6229222 | 0 .861302891 | 0 . 16631 | 5 . 17898 | 2 .23E-07 | 7 .34E-07 |
| DUSP6 | 1 1333 .5371 | -0 . 168963133 | 0 . 16069 | -1 .0515 | 0 .29304 | 0 .34653 |
| DUSP7 | 2071 .433136 | -0 .003408143 | 0 . 12653 | -0 .0269 | 0 .97851 | 0 .98148 |
| DUSP8 | 103 .4110218 | 1 .686120143 | 0 .21343 | 7 .89996 | 2 .79E-15 | 2 .47E-14 |
| DUSP9 | 142 .4818502 | 0 .941778753 | 0 .31796 | 2 .96192 | 0 .00306 | 0 .00533 |
| DUT | 4274 .802528 | 0 .050957275 | 0 . 1 156 | 0 .44079 | 0 .65936 | 0 .70485 |
| DUXA | 4 .088159797 | -4 .358137241 | 0 .8268 | -5 .2711 | 1 .36E-07 | 4 .62E-07 |
| DVL1 | 3107 .593381 | 0 .540329648 | 0 .09739 | 5 .54796 | 2 .89E-08 | 1 .08E-07 |
| DVL2 | 1964 .850972 | 0 .346527881 | 0 .08019 | 4 .32108 | 1 .55E-05 | 3 .89E-05 |
| DVL3 | 2136 . 159142 | 0 .440431515 | 0 . 10336 | 4 .26115 | 2 .03E-05 | 5 .01E-05 |
| DXO | 325 .2781543 | 0 .062926286 | 0 . 10226 | 0 .61538 | 0 .5383 | 0 .59159 |
| DYDC1 | 3 .354488389 | -3 .453675819 | 0 .77509 | -4 .4558 | 8 .36E-06 | 2 . 17E-05 |
| DYDC2 | 35 .47387459 | 1 . 177310179 | 0 .47156 | 2 .49664 | 0 .01254 | 0 .01959 |
| DYM | 3986 .455501 | -0 .499902463 | 0 .05196 | -9 .6212 | 6 .51E-22 | 1 .22E-20 |
| DYNAP | 13 .51537637 | -1 .08367125 | 0 .5337 | -2 .0305 | 0 .04231 | 0 .06006 |
| DYNC1H1 | 49130 .93133 | 0 . 1 1771592 | 0 .08689 | 1 .35483 | 0 . 17547 | 0 .21945 |
| DYNC1I1 | 1013 .527245 | -1 .549027133 | 0 .2289 | -6 .7671 | 1 .31E-1 1 | 7 .53E-1 1 |
| DYNC1I2 | 8226 .982037 | -0 .322616471 | 0 .08161 | -3 .9529 | 7 .72E-05 | 0 .00017 |
| DYNC1LI1 | 4608 .203747 | -0 . 135091226 | 0 .06427 | -2 . 1021 | 0 .03555 | 0 .05116 |
| DYNC1LI2 | 8250 .588476 | -0 .005769695 | 0 .08108 | -0 .0712 | 0 .94327 | 0 .95256 |
| DYNC2H1 | 7979 .412356 | -0 .973511393 | 0 . 1 1381 | -8 .5535 | 1 . 19E-17 | 1 .40E-16 |
| DYNC2LI1 | 1586 . 170246 | -0 .593439277 | 0 . 1 136 | -5 .2241 | 1 .75E-07 | 5 .83E-07 |
| DYNLL1 | 1 1901 . 15451 | -0 .453010779 | 0 . 12371 | -3 .6619 | 0 .00025 | 0 .00052 |
| DYNLL2 | 4792 .470642 | -0 .005455448 | 0 . 10087 | -0 .0541 | 0 .95687 | 0 .96365 |
| DYNLRB1 | 1263 .744572 | -0 .397498167 | 0 . 17016 | -2 .3361 | 0 .01949 | 0 .02948 |
| DYNLRB2 | 51 . 13147331 | -1 .884915966 | 0 .21214 | -8 .8852 | 6 .38E-19 | 8 .63E-18 |
| DYNLT1 | 1567 .537776 | -0 .018481721 | 0 . 13162 | -0 . 1404 | 0 .88833 | 0 .90628 |
| DYNLT3 | 3950 .870605 | -0 .583146414 | 0 . 14937 | -3 .9041 | 9 .46E-05 | 0 .00021 |

| DYRK1A | 10982 .32729 | 0 .065753381 | 0 .05917 | 1 . 1 1 127 | 0 .26645 | 0 .31818 |
| --- | --- | --- | --- | --- | --- | --- |
| DYRK1B | 505 .4772524 | 0 .558830783 | 0 . 1 158 | 4 .82582 | 1 .39E-06 | 4 .06E-06 |
| DYRK2 | 6216 .392459 | 0 .936980962 | 0 .09073 | 10 .3274 | 5 .30E-25 | 1 .41E-23 |
| DYRK3 | 714 .5707088 | 1 .349994127 | 0 . 17016 | 7 .93384 | 2 . 12E-15 | 1 .91E-14 |
| DYRK4 | 896 .9858027 | -0 .727838941 | 0 . 12347 | -5 .8948 | 3 .75E-09 | 1 .58E-08 |
| DYSF | 1029 .45949 | 1 .980036761 | 0 . 1548 | 12 .7908 | 1 .85E-37 | 1 .73E-35 |
| DYTN | 7 .218520426 | -5 . 183804353 | 0 .79805 | -6 .4956 | 8 .27E-1 1 | 4 .29E-10 |
| DYX1C1 | 36 .67507368 | -1 . 1 13038392 | 0 . 15188 | -7 .3283 | 2 .33E-13 | 1 .65E-12 |
| DZANK1 | 226 .9230114 | -0 . 126418691 | 0 . 1 1446 | -1 . 1045 | 0 .26939 | 0 .3214 |
| DZIP1 | 898 .4504257 | -0 .000234623 | 0 .2071 | -0 .0011 | 0 .9991 | 0 .9992 |
| DZIP1L | 932 .2738962 | -0 .601653777 | 0 . 14448 | -4 . 1642 | 3 . 13E-05 | 7 .47E-05 |
| DZIP3 | 2529 .677808 | -0 .700635077 | 0 . 1259 | -5 .5651 | 2 .62E-08 | 9 .89E-08 |
| E2F1 | 657 .8087986 | 2 .078572152 | 0 . 18853 | 1 1 .0253 | 2 .89E-28 | 1 .08E-26 |
| E2F2 | 541 .7574985 | 0 .511424435 | 0 . 14507 | 3 .52539 | 0 .00042 | 0 .00084 |
| E2F3 | 1665 .779888 | 1 .449392405 | 0 . 12254 | 1 1 .8282 | 2 .79E-32 | 1 .56E-30 |
| E2F4 | 2753 . 149122 | 0 .50906922 | 0 .09796 | 5 . 1968 | 2 .03E-07 | 6 .70E-07 |
| E2F5 | 542 .6844823 | -0 .721686685 | 0 . 13346 | -5 .4076 | 6 .39E-08 | 2 .28E-07 |
| E2F6 | 1468 .010315 | 0 .359137098 | 0 .0954 | 3 .76454 | 0 .00017 | 0 .00035 |
| E2F7 | 474 .9035273 | 1 .608625081 | 0 . 18259 | 8 .80993 | 1 .25E-18 | 1 .64E-17 |
| E2F8 | 218 . 1264024 | -0 .293845922 | 0 . 18413 | -1 .5959 | 0 . 1 1052 | 0 . 14426 |
| E4F1 | 1732 .017797 | 0 .556844342 | 0 .05952 | 9 .35487 | 8 .37E-21 | 1 .40E-19 |
| EAF1 | 1513 .244717 | 0 .527271845 | 0 .07535 | 6 .99774 | 2 .60E-12 | 1 .64E-1 1 |
| EAF2 | 184 .849343 | 0 .513871741 | 0 . 19867 | 2 .58657 | 0 .00969 | 0 .01546 |
| EAPP | 2893 .347616 | -0 .704392504 | 0 . 1 1 197 | -6 .291 | 3 . 15E-10 | 1 .52E-09 |
| EARS2 | 2590 .675119 | 0 .264750596 | 0 .08943 | 2 .96051 | 0 .00307 | 0 .00535 |
| EBAG9 | 1603 .354587 | -0 .425032653 | 0 .09027 | -4 .7083 | 2 .50E-06 | 6 .99E-06 |
| EBF1 | 2017 .985177 | 0 .585439138 | 0 .22541 | 2 .5972 | 0 .0094 | 0 .01503 |
| EBF2 | 202 .266418 | 0 .343221913 | 0 .288 | 1 . 19174 | 0 .23336 | 0 .28288 |
| EBF3 | 532 .4779583 | 0 .280835329 | 0 .21898 | 1 .28249 | 0 . 19967 | 0 .24629 |
| EBF4 | 155 .3187032 | 0 .334218298 | 0 . 17406 | 1 .92013 | 0 .05484 | 0 .07612 |
| EBI3 | 52 .61414517 | 2 .208703143 | 0 .27162 | 8 . 13159 | 4 .24E-16 | 4 . 16E-15 |
| EBLN1 | 2 .380530286 | -4 .081787671 | 0 .96166 | -4 .2445 | 2 . 19E-05 | 5 .36E-05 |
| EBLN2 | 405 .416397 | -0 .988035769 | 0 . 15891 | -6 .2176 | 5 .05E-10 | 2 .38E-09 |
| EBNA1BP2 | 2818 .859524 | 0 .381873802 | 0 . 12397 | 3 .08044 | 0 .00207 | 0 .0037 |
| EBP | 1947 .942428 | 0 .490632212 | 0 . 16006 | 3 .06535 | 0 .00217 | 0 .00388 |
| EBPL | 1347 .039189 | -0 .49267275 | 0 . 12546 | -3 .927 | 8 .60E-05 | 0 .00019 |
| ECD | 2495 .49644 | -0 .241441352 | 0 .05889 | -4 . 1 | 4 . 13E-05 | 9 .67E-05 |
| ECE1 | 3357 .925045 | 1 .004659729 | 0 . 12969 | 7 .74654 | 9 .44E-15 | 7 .81E-14 |
| ECE2 | 278 .0825605 | 0 .466082339 | 0 . 16151 | 2 .8857 | 0 .00391 | 0 .0067 |
| ECEL1 | 56 .93427291 | 3 .010235123 | 0 .37693 | 7 .98622 | 1 .39E-15 | 1 .27E-14 |
| ECH1 | 1279 .679733 | 0 .521505502 | 0 . 15641 | 3 .33425 | 0 .00086 | 0 .00163 |
| ECHDC1 | 4437 .0512 | -0 .09772948 | 0 . 10978 | -0 .8902 | 0 .37336 | 0 .42926 |
| ECHDC2 | 1950 .634523 | -0 .575489254 | 0 . 17915 | -3 .2124 | 0 .00132 | 0 .00244 |
| ECHDC3 | 911 .7223575 | -0 .056195561 | 0 . 16263 | -0 .3455 | 0 .72968 | 0 .76821 |
| ECHS1 | 3959 . 174152 | 0 . 139313848 | 0 . 12155 | 1 . 14615 | 0 .25173 | 0 .30262 |
| ECI1 | 1014 .931497 | 0 . 194710523 | 0 . 1 1502 | 1 .69289 | 0 .09048 | 0 . 12043 |
| ECI2 | 1572 .869599 | -0 .003964501 | 0 . 10408 | -0 .0381 | 0 .96962 | 0 .97406 |
| ECM1 | 2486 .824742 | 1 .365381747 | 0 .21031 | 6 .49227 | 8 .46E-1 1 | 4 .38E-10 |
| ECM2 | 2339 .791145 | -0 .694244656 | 0 . 19509 | -3 .5585 | 0 .00037 | 0 .00075 |
| ECSCR | 366 .089115 | 0 .674740533 | 0 . 17183 | 3 .9268 | 8 .61E-05 | 0 .00019 |
| ECSIT | 1271 . 104572 | -0 . 186056224 | 0 . 10976 | -1 .6951 | 0 .09005 | 0 . 1 1988 |
| ECT2 | 2064 .330718 | 0 .816641264 | 0 . 13535 | 6 .03355 | 1 .60E-09 | 7 .09E-09 |
| ECT2L | 233 .521636 | -1 .399853674 | 0 . 18533 | -7 .5532 | 4 .25E-14 | 3 .28E-13 |
| EDA | 477 .8633788 | -1 . 176249971 | 0 .20485 | -5 .7419 | 9 .36E-09 | 3 .74E-08 |
| EDA2R | 1063 .878704 | -0 .584142335 | 0 . 19903 | -2 .9349 | 0 .00334 | 0 .00579 |
| EDAR | 78 .05514801 | 0 .70847683 | 0 .32515 | 2 . 17889 | 0 .02934 | 0 .04295 |

| EDARADD 237 .0580558 -0 .544246462 | 0 . 17465 -3 . 1 162 | 0 .00183 | 0 .00331 |
| --- | --- | --- | --- |
| EDC3 1 120 .573151 -0 . 186702998 | 0 .09889 -1 .888 | 0 .05902 | 0 .0814 |
| EDC4 1870 .546394 0 .831420596 | 0 .09304 8 .93574 | 4 .04E-19 | 5 .58E-18 |
| EDDM3A 4 .054664619 -2 . 121709147 | 0 .51535 -4 . 1 17 | 3 .84E-05 | 9 .03E-05 |
| EDDM3B 4 .640506414 -1 .707430739 | 0 .52197 -3 .2712 | 0 .00107 | 0 .00201 |
| EDEM1 7214 .574785 0 .601558024 | 0 .091 6 .6108 | 3 .82E-1 1 | 2 .08E-10 |
| EDEM2 1872 .078457 0 .71249494 | 0 . 12359 5 .76493 | 8 . 17E-09 | 3 .30E-08 |
| EDEM3 6543 .756752 0 .461904989 | 0 .09833 4 .69756 | 2 .63E-06 | 7 .35E-06 |
| EDF1 10175 .94292 -0 .429894159 | 0 . 13809 -3 . 1 132 | 0 .00185 | 0 .00334 |
| EDIL3 2420 .971464 0 .006094304 | 0 .22595 0 .02697 | 0 .97848 | 0 .98148 |
| EDN1 688 . 1826032 -0 .424332057 | 0 .21409 -1 .9821 | 0 .04747 | 0 .06675 |
[truncated: 1,187,562 more chars]
