# Supplementary material for: Identification of a Seven-Differentially Expressed Gene-Based Recurrence-Free Survival Model for Melanoma Patients
Source: Dis Markers. 2022 Jul 14;2022:3915112. doi: 10.1155/2022/3915112 (PMC9303152; doi:10.1155/2022/3915112)
Supplement: Supplementary Materials — Figure S1: functional enrichment result of overlapped DEGs between GSE98394 and GSE46517. Figure S2: the K-M curves show genes that significantly correlated with PFS. Grouping of samples is based on median gene expression. [file 3915112.f1.zip › Supplementary table 2 (1).docx]

**Supplementary table 2. The differentially expressed genes between melanoma and nevus tissues in GSE46517 cohort.**

**Gene_ID** **log** **(Foldchange） AveExpression** **t** ***P*** **-Value** **Adjust.*P*** **-Value**

HLF -1 .216195619 7.050147082 -8 .4065 1 .46E-10 1 .42E-06

| PRAME | 3 .630892273 | 8 .307626459 | 8 .26974 | 2 .27E-10 1 .42E-06 |
| --- | --- | --- | --- | --- |
| PHYHIP | -1 .769629478 | 5 .804513669 | -7 .8456 | 8 .88E-10 3 .71E-06 |
| CTSA | 0 .958815623 | 10 .00399312 | 7 .56781 | 2 . 19E-09 5 .99E-06 |
| ID4 | -1 .018311075 | 9 .317216144 | -7 .5417 | 2 .39E-09 5 .99E-06 |
| TUBB2A | 1 .269557788 | 10 .56576418 | 7 .3797 | 4 .05E-09 8 .48E-06 |
| CTSZ | 1 .352671936 | 9 .027447988 | 7 .2081 | 7 . 12E-09 1 .28E-05 |
| RPS15A | -1 .508448827 | 10 . 15098147 | -7 . 1226 | 9 .44E-09 1 .36E-05 |
| TMEM47 | -2 . 196522538 | 7 .096241101 | -7 . 1 121 | 9 .77E-09 1 .36E-05 |
| S100A7 | 4 . 157931862 | 10 .90641346 | 7 .06091 | 1 . 16E-08 1 .45E-05 |
| HN1 | 1 .300592814 | 7 .254503291 | 7 .01604 | 1 .34E-08 1 .51E-05 |
| TGFBR3 | -1 . 17208581 | 8 . 138537521 | -6 .9843 | 1 .49E-08 1 .51E-05 |
| NPY1R | -1 .9909411 18 | 4 .412745548 | -6 .9513 | 1 .66E-08 1 .51E-05 |
| LRRC59 | 0 .943206427 | 9 .007478766 | 6 .94602 | 1 .69E-08 1 .51E-05 |
| FBXW7 | -1 .455003042 | 7 .346992187 | -6 .9102 | 1 .90E-08 1 .59E-05 |
| ALDH3A2 | -1 . 107942193 | 9 .855463642 | -6 .7711 | 3 .01E-08 2 .36E-05 |
| TNFRSF21 | 1 .293498827 | 8 .962555156 | 6 .74668 | 3 .27E-08 2 .41E-05 |
| CRBN | -1 .046994346 | 8 . 199084493 | -6 .7196 | 3 .57E-08 2 .49E-05 |
| NFIB | -1 .412909643 | 9 .994734235 | -6 .7039 | 3 .76E-08 2 .49E-05 |
| BARD1 | -1 . 181835142 | 5 .820679024 | -6 .6038 | 5 .25E-08 3 .29E-05 |
| FBXO21 | -0 .73778977 | 8 .458140671 | -6 .5765 | 5 .74E-08 3 .37E-05 |
| CEACAM6 | -1 .04386429 | 7 .729541867 | -6 .5661 | 5 .95E-08 3 .37E-05 |
| RNASE4 | -1 .221269792 | 7 .635734415 | -6 .5546 | 6 . 18E-08 3 .37E-05 |
| SPAG5 | 1 .030636323 | 5 .864149632 | 6 .5277 | 6 .75E-08 3 .42E-05 |
| PAQR4 | 0 .960696649 | 7 .521722313 | 6 .52538 | 6 .81E-08 3 .42E-05 |
| DENND4C | -0 .892763613 | 6 .878323265 | -6 .5099 | 7 . 17E-08 3 .46E-05 |
| SPRR1A | 3 .501917863 | 1 1 .27659982 | 6 .45865 | 8 .49E-08 3 .95E-05 |
| DNAJB4 | -0 .796482692 | 6 . 188130861 | -6 .4424 | 8 .97E-08 4 .02E-05 |
| SPRR1B | 3 .805693765 | 10 .30748325 | 6 .39251 | 1 .06E-07 4 .58E-05 |
| RGS1 | 2 .708202775 | 7 .814872811 | 6 .37346 | 1 . 13E-07 4 .71E-05 |
| PGS1 | 0 .770552457 | 7 .235109054 | 6 .35418 | 1 .20E-07 4 .86E-05 |
| GNAQ | -0 .736278096 | 9 .37134357 | -6 .3073 | 1 .40E-07 5 .51E-05 |
| RNF38 | -0 .768514624 | 7 .56567436 | -6 .2896 | 1 .49E-07 5 .66E-05 |
| TPX2 | 0 .998640857 | 7 .562711271 | 6 .21089 | 1 .93E-07 7 . 14E-05 |
| LETMD1 | -1 .214342457 | 7 .713369104 | -6 . 1874 | 2 .09E-07 7 .45E-05 |
| RPL9 | -1 .010459132 | 1 1 .33835116 | -6 . 1808 | 2 . 14E-07 7 .45E-05 |
| FABP5 | 2 . 141472737 | 10 .91285676 | 6 . 16343 | 2 .26E-07 7 .61E-05 |
| ZNF292 | -1 . 177052828 | 7 .41868598 | -6 . 1581 | 2 .31E-07 7 .61E-05 |
| CBX7 | -1 .055753354 | 7 .355439977 | -6 .0955 | 2 .84E-07 9 .00E-05 |
| CEP68 | -0 .682333541 | 8 .990843674 | -6 .0923 | 2 .87E-07 9 .00E-05 |
| KRT6A | 1 .927122574 | 1 1 .49525689 | 6 .08042 | 2 .98E-07 9 . 13E-05 |
| EXPH5 | -1 . 182724401 | 8 .724874109 | -6 .054 | 3 .26E-07 9 .58E-05 |
| IVL | 2 .755571358 | 8 .864063556 | 6 .05004 | 3 .30E-07 9 .58E-05 |
| ZNF395 | -0 .939439635 | 8 .290246491 | -6 .0448 | 3 .36E-07 9 .58E-05 |
| SERPINB3 | 3 . 120852718 | 9 .259296118 | 6 .00537 | 3 .83E-07 0 .000106771 |
| IL11RA | -1 .45143543 | 7 .401578199 | -5 .9902 | 4 .03E-07 0 .000109836 |
| FTL | 0 .672444794 | 13 .58441658 | 5 .97212 | 4 .28E-07 0 .000114163 |
| AKR1B10 | 2 .028538069 | 7 . 1 10352121 | 5 .93543 | 4 .83E-07 0 .000126264 |
| FGFR2 | -1 .075934021 | 8 .835699481 | -5 .9113 | 5 .23E-07 0 .000133741 |
| SERPINB4 | 3 .484403426 | 7 .093874451 | 5 .90579 | 5 .33E-07 0 .000133741 |
| S100A9 | 3 .288648018 | 9 .332284588 | 5 .89354 | 5 .55E-07 0 .000135332 |
| PMEL | 1 .039255494 | 12 .00919653 | 5 .89041 | 5 .61E-07 0 .000135332 |
| GLA | 0 .512120272 | 8 .341290954 | 5 .87059 | 5 .99E-07 0 .000138126 |

| DFNA5 | 1 .061098391 | 7 .343277743 | 5 .86998 | 6 .00E-07 0 .000138126 |
| --- | --- | --- | --- | --- |
| BMP2 | -0 .928261205 | 6 .52007583 | -5 .8631 | 6 . 14E-07 0 .000138126 |
| LAMB4 | -0 .862808842 | 6 .410318669 | -5 .8619 | 6 . 16E-07 0 .000138126 |
| PFDN5 | -1 .067307173 | 10 .56863872 | -5 .8422 | 6 .58E-07 0 .000144869 |
| RPL27 | -1 .02186293 | 10 .82963714 | -5 .8184 | 7 . 12E-07 0 .000154075 |
| FABP7 | -2 .082316339 | 8 .706366127 | -5 .8116 | 7 .28E-07 0 .000154903 |
| UPP1 | 1 .640380614 | 6 .890081245 | 5 .79482 | 7 .70E-07 0 .000161045 |
| ASS1 | -1 .547674916 | 8 . 180096482 | -5 .7866 | 7 .91E-07 0 .000162806 |
| CLDN8 | -1 . 17286879 | 4 .911 129519 | -5 .7699 | 8 .36E-07 0 .000169255 |
| ITPR2 | -0 .7222115 | 7 .952755154 | -5 .6912 | 1 .09E-06 0 .000214553 |
| PI3 | 3 .361760646 | 9 .958390743 | 5 .68876 | 1 .09E-06 0 .000214553 |
| MKL2 | -0 .781145534 | 7 .223466193 | -5 .6758 | 1 . 14E-06 0 .00022053 |
| SERHL2 | -1 .225489519 | 6 .077592146 | -5 .6656 | 1 . 18E-06 0 .000224615 |
| PGAP1 | -1 .019468047 | 6 .851509613 | -5 .6431 | 1 .27E-06 0 .000238409 |
| DDX39A | 0 .725008085 | 8 .750814905 | 5 .6378 | 1 .30E-06 0 .000239028 |
| ADD3 | -1 . 130650681 | 10 .91122936 | -5 .6327 | 1 .32E-06 0 .000239601 |
| HSD11B2 | -1 .545835039 | 6 .200538158 | -5 .6087 | 1 .43E-06 0 .000255644 |
| SAV1 | -0 .947156555 | 6 .660678276 | -5 .5915 | 1 .51E-06 0 .000266813 |
| RBM19 | 0 .639630075 | 7 .41018504 | 5 .58248 | 1 .56E-06 0 .000271069 |
| AUTS2 | -1 .001814888 | 7 .960505214 | -5 .5744 | 1 .60E-06 0 .000274259 |
| CERS6 | -0 .665355101 | 8 .482823042 | -5 .5706 | 1 .62E-06 0 .000274259 |
| PHACTR1 | 1 .66788811 1 | 7 .404230846 | 5 .55851 | 1 .68E-06 0 .000281674 |
| GALK1 | -2 .21616296 | 3 .873816432 | -5 .5355 | 1 .82E-06 0 .000299934 |
| EFS | -0 .990670066 | 7 .993996546 | -5 .5302 | 1 .85E-06 0 .000301236 |
| ARL3 | -0 .592752863 | 7 .98347941 | -5 .5169 | 1 .93E-06 0 .000307114 |
| FHL1 | -1 . 1 10087048 | 9 .806496619 | -5 .5124 | 1 .96E-06 0 .000307114 |
| MST4 | -1 .748384452 | 5 .508226037 | -5 .5088 | 1 .98E-06 0 .000307114 |
| SMIM8 | -0 .692098007 | 6 .276106915 | -5 .5058 | 2 .00E-06 0 .000307114 |
| ITM2A | -1 .371920093 | 8 .447601334 | -5 .5053 | 2 .01E-06 0 .000307114 |
| CFH | -1 .961738224 | 7 .65317701 | -5 .5009 | 2 .04E-06 0 .000307903 |
| KRT19 | -2 .374543651 | 5 . 187104584 | -5 .4861 | 2 . 14E-06 0 .000316584 |
| SDC4 | -0 .957935256 | 9 .003145654 | -5 .4852 | 2 . 14E-06 0 .000316584 |
| TACC3 | 1 . 170914014 | 6 .075993528 | 5 .48143 | 2 . 17E-06 0 .00031683 |
| LRP4 | -0 .890320678 | 6 .45724424 | -5 .4479 | 2 .43E-06 0 .000349763 |
| INSR | -0 .862321199 | 7 .620120856 | -5 .435 | 2 .53E-06 0 .000359545 |
| SRM | 0 .7671358 | 9 .540580901 | 5 .42669 | 2 .60E-06 0 .000359545 |
| SCGB1D2 | -2 .87379085 | 5 .569474288 | -5 .4266 | 2 .60E-06 0 .000359545 |
| UBE2S | 1 .067212777 | 8 .027035848 | 5 .42593 | 2 .61E-06 0 .000359545 |
| CENPM | 1 .499972285 | 5 .025767918 | 5 .40995 | 2 .75E-06 0 .000374857 |
| ZIC1 | 2 . 144873121 | 5 .575791422 | 5 .39411 | 2 .90E-06 0 .000390696 |
| PIAS1 | -0 .529808453 | 8 .962072699 | -5 .3693 | 3 . 14E-06 0 .000419403 |
| AFF1 | -0 .552726935 | 8 .937541728 | -5 .3562 | 3 .28E-06 0 .000433231 |
| MCOLN1 | 0 .912368628 | 6 .94912712 | 5 .34345 | 3 .42E-06 0 .000447139 |
| CFHR2 | -0 .879816085 | 6 .468841722 | -5 .3359 | 3 .51E-06 0 .000451528 |
| COX7A2L | -0 .783673638 | 8 .882336642 | -5 .3342 | 3 .53E-06 0 .000451528 |
| S100A8 | 2 .829651461 | 1 1 .04385986 | 5 .32794 | 3 .60E-06 0 .000456277 |
| MAOA | -1 .216157897 | 8 .068061096 | -5 .3123 | 3 .79E-06 0 .000475483 |
| JAG2 | -0 .882609725 | 9 .095335251 | -5 .2962 | 4 .00E-06 0 .000496346 |
| PMM2 | 0 .883444545 | 6 .498761268 | 5 .29169 | 4 .06E-06 0 .000498875 |
| MAN2B1 | 0 .722439259 | 9 .498669533 | 5 .28634 | 4 . 13E-06 0 .00050279 |
| SCD | 0 .819743752 | 10 .22234812 | 5 .27266 | 4 .32E-06 0 .000520816 |
| CHP2 | -1 .552540045 | 6 .828649566 | -5 .2668 | 4 .40E-06 0 .000525895 |
| EPCAM | -1 .296558593 | 5 .263204024 | -5 .2579 | 4 .53E-06 0 .000536298 |
| PUS1 | 0 .761583963 | 6 .567416214 | 5 .25011 | 4 .65E-06 0 .000545101 |
| PDZD2 | -1 .404925536 | 5 .59848408 | -5 .2395 | 4 .81E-06 0 .000559094 |
| KCNJ2 | -0 .839122093 | 6 .266133363 | -5 .2321 | 4 .93E-06 0 .000563974 |

| KLF3-AS1 | -0 .847139817 | 4 .852747555 | -5 .2313 | 4 .94E-06 0 .000563974 |
| --- | --- | --- | --- | --- |
| LMBRD1 | -0 .571262029 | 8 .702879667 | -5 .2116 | 5 .27E-06 0 .000592466 |
| PPP3CB | -0 .619108393 | 8 .058979449 | -5 .2108 | 5 .29E-06 0 .000592466 |
| CDC45 | 0 .782842488 | 5 .654122863 | 5 .20569 | 5 .38E-06 0 .000597073 |
| ALDH6A1 | -0 .748934855 | 8 .909638019 | -5 . 1968 | 5 .54E-06 0 .000609351 |
| CCNI | -0 .692299764 | 1 1 .69873007 | -5 . 1866 | 5 .72E-06 0 .000624589 |
| GARS | 0 .617403285 | 9 .628384812 | 5 . 18241 | 5 .80E-06 0 .000627707 |
| GPR37 | -1 .422307464 | 4 .31043669 | -5 . 1774 | 5 .90E-06 0 .000632634 |
| IFI16 | 0 .885126051 | 1 1 .34135717 | 5 . 1748 | 5 .95E-06 0 .000632634 |
| CRY2 | -0 .638358131 | 6 .991233561 | -5 . 171 | 6 .02E-06 0 .000635104 |
| PPP3CA | -0 .713554981 | 9 .330831059 | -5 . 163 | 6 . 18E-06 0 .0006466 |
| DLG5 | -0 .656978791 | 7 .973221421 | -5 . 1588 | 6 .27E-06 0 .000650112 |
| ATOX1 | 1 .007538689 | 8 .929394322 | 5 . 14923 | 6 .47E-06 0 .000660148 |
| C9orf3 | -0 .873698649 | 7 .498233357 | -5 . 1491 | 6 .47E-06 0 .000660148 |
| KLHDC2 | -0 .707831464 | 8 .310047322 | -5 . 1 167 | 7 . 19E-06 0 .000723419 |
| CAMLG | -0 .796670792 | 8 .519144047 | -5 . 1 162 | 7 .21E-06 0 .000723419 |
| SUN1 | -0 .661031189 | 8 .635774523 | -5 . 1 121 | 7 .30E-06 0 .000727299 |
| WIPI1 | 1 .218575336 | 8 .320927083 | 5 . 10917 | 7 .37E-06 0 .000728479 |
| PSENEN | 0 .833401599 | 8 . 180134107 | 5 . 10372 | 7 .51E-06 0 .000734633 |
| C1QB | 2 .368196293 | 8 .512137668 | 5 . 10181 | 7 .55E-06 0 .000734633 |
| AVEN | 0 .743983923 | 6 .899468407 | 5 .09727 | 7 .67E-06 0 .000739856 |
| RPN2 | 0 .620462482 | 12 .25585379 | 5 .08126 | 8 .08E-06 0 .000767472 |
| ZNF273 | -0 .600757478 | 6 .502697165 | -5 .0808 | 8 .09E-06 0 .000767472 |
| MELK | 0 .802953349 | 6 .866995257 | 5 .07905 | 8 . 13E-06 0 .000767472 |
| UBE2C | 0 .681165068 | 8 .755483817 | 5 .07235 | 8 .31E-06 0 .000772162 |
| LONP1 | 0 .526611046 | 8 .73170776 | 5 .07053 | 8 .36E-06 0 .000772162 |
| KPNA2 | 1 .01751265 | 9 .425052845 | 5 .06935 | 8 .40E-06 0 .000772162 |
| BCL11A | -0 .971769737 | 8 .760993009 | -5 .0681 | 8 .43E-06 0 .000772162 |
| 2-Mar | 0 .825967433 | 7 . 120413133 | 5 .06315 | 8 .57E-06 0 .000779051 |
| TSPAN8 | -1 .087481342 | 5 .925010851 | -5 .0548 | 8 .80E-06 0 .000794797 |
| ZSCAN18 | -1 .358362284 | 7 .295516421 | -5 .0507 | 8 .92E-06 0 .00079761 |
| PPP2R5E | -0 .875080401 | 7 .56528542 | -5 .0479 | 9 .00E-06 0 .00079761 |
| SSBP2 | -0 .944216991 | 7 .974150236 | -5 .0458 | 9 .07E-06 0 .00079761 |
| GIPC2 | -1 .20399359 | 4 .440863457 | -5 .045 | 9 .09E-06 0 .00079761 |
| ADH1B | -1 . 155396546 | 6 .982978121 | -5 .0428 | 9 . 16E-06 0 .000797852 |
| DUSP14 | 0 .779916412 | 8 .888019002 | 5 .00962 | 1 .02E-05 0 .000882694 |
| ZBTB20 | -0 .655282991 | 9 . 1 19877928 | -4 .9997 | 1 .05E-05 0 .0009055 |
| PTN | -1 .051019848 | 10 .06990617 | -4 .9922 | 1 .08E-05 0 .000921563 |
| ASPM | 1 .363043128 | 5 .212348631 | 4 .98748 | 1 . 10E-05 0 .000929368 |
| PCBP2 | -0 .587076808 | 1 1 .08290083 | -4 .9824 | 1 . 1 1E-05 0 .000936934 |
| WDR52 | -0 .754857715 | 5 .373276119 | -4 .9809 | 1 . 12E-05 0 .000936934 |
| PLOD3 | 0 .968513132 | 10 .39078613 | 4 .97541 | 1 . 14E-05 0 .000947365 |
| BUB1B | 0 .976267476 | 6 .041835534 | 4 .96612 | 1 . 17E-05 0 .000953005 |
| THY1 | 1 .036260745 | 9 .941739433 | 4 .96583 | 1 . 18E-05 0 .000953005 |
| NR3C1 | -0 .705694565 | 10 .48098476 | -4 .9657 | 1 . 18E-05 0 .000953005 |
| NDRG2 | -0 .891251397 | 9 .322110536 | -4 .9655 | 1 . 18E-05 0 .000953005 |
| MED13 | -0 .59934277 | 8 .476367899 | -4 .959 | 1 .20E-05 0 .00096219 |
| PWP2 | 0 .529835202 | 6 .749461012 | 4 .95863 | 1 .20E-05 0 .00096219 |
| DICER1 | -0 .455928078 | 9 .658309708 | -4 .9562 | 1 .21E-05 0 .000963613 |
| ZBTB16 | -1 .403790003 | 7 .024845113 | -4 .9537 | 1 .22E-05 0 .000965553 |
| KCTD5 | 0 .580547119 | 7 .812331895 | 4 .94613 | 1 .25E-05 0 .000982051 |
| TYK2 | 0 .541810603 | 8 .455913932 | 4 .94459 | 1 .26E-05 0 .000982051 |
| PLAGL1 | -0 .809992246 | 8 . 189738622 | -4 .9389 | 1 .28E-05 0 .000994277 |
| MMP11 | 1 . 143040865 | 7 .721333164 | 4 .92861 | 1 .33E-05 0 .00102132 |
| PER3 | -0 .871106477 | 6 . 154169322 | -4 .9268 | 1 .33E-05 0 .00102132 |
| GSTP1 | 0 .698005892 | 10 .90688993 | 4 .92224 | 1 .35E-05 0 .001025981 |

| RAB11 FIP2 | -0 .868213661 | 7 .715435015 | -4 .9202 | 1 .36E-05 0 .001025981 |
| --- | --- | --- | --- | --- |
| ARPC1B | 0 .630894097 | 10 .47788539 | 4 .91983 | 1 .37E-05 0 .001025981 |
| ZFYVE21 | -0 .691430834 | 6 .729783872 | -4 .9127 | 1 .40E-05 0 .001043776 |
| LPP | -0 .492490197 | 9 .365780927 | -4 .9078 | 1 .42E-05 0 .001051808 |
| FGFR3 | -1 .65734339 | 8 .752743751 | -4 .9067 | 1 .42E-05 0 .001051808 |
| CSK | 0 .663541513 | 8 .689055174 | 4 .9028 | 1 .44E-05 0 .001058852 |
| DAAM1 | -0 .887535782 | 7 .360688799 | -4 .8967 | 1 .47E-05 0 .001073607 |
| ACTB | 0 .540896944 | 14 . 17948221 | 4 .89294 | 1 .49E-05 0 .001075337 |
| CTPS1 | 0 .820637253 | 6 .519834904 | 4 .89266 | 1 .49E-05 0 .001075337 |
| TIMP2 | 1 .230502335 | 9 .0666127 | 4 .88098 | 1 .55E-05 0 .001109899 |
| LMNB1 | 0 .702871257 | 7 .486317717 | 4 .87936 | 1 .56E-05 0 .001109899 |
| SPRR2B | 2 .7759626 | 8 .441802775 | 4 .86664 | 1 .62E-05 0 .00114534 |
| PIP | -1 .843219214 | 8 . 160370568 | -4 .8662 | 1 .62E-05 0 .00114534 |
| PPP1CA | 0 .705827477 | 8 .997329521 | 4 .86226 | 1 .65E-05 0 .001153389 |
| KRT16 | 2 .554579229 | 9 .835973745 | 4 .85967 | 1 .66E-05 0 .001156642 |
| CTSD | 0 .858637473 | 9 .038843325 | 4 .85493 | 1 .68E-05 0 .001168019 |
| SASH1 | -0 .583000134 | 10 .04038377 | -4 .8531 | 1 .69E-05 0 .00116861 |
| SOWAHC | -0 .811989491 | 7 .347519882 | -4 .8492 | 1 .72E-05 0 .001171132 |
| CXADR | -1 .726995957 | 8 .210787571 | -4 .8487 | 1 .72E-05 0 .001171132 |
| TNC | 1 .556566229 | 8 .787061052 | 4 .84357 | 1 .75E-05 0 .001171132 |
| PLCH2 | -1 .047310829 | 5 .717817494 | -4 .8434 | 1 .75E-05 0 .001171132 |
| SLC20A1 | 0 .940018821 | 8 .200351684 | 4 .84323 | 1 .75E-05 0 .001171132 |
| RPL34 | -0 .840924884 | 1 1 . 17615423 | -4 .8423 | 1 .76E-05 0 .001171132 |
| RABGAP1 | -0 .664276002 | 9 .058908665 | -4 .8407 | 1 .76E-05 0 .001171132 |
| CHL1 | -2 .31991249 | 7 . 144247165 | -4 .8351 | 1 .80E-05 0 .001186261 |
| DBF4 | 0 .944020144 | 5 .612338755 | 4 .82876 | 1 .83E-05 0 .001204561 |
| PKN2 | -0 .646253361 | 8 .896083757 | -4 .8214 | 1 .88E-05 0 .001226918 |
| SCAMP1 | -0 .574792495 | 9 .625896416 | -4 .8176 | 1 .90E-05 0 .00123599 |
| PPP1R3C | -1 .223236888 | 7 . 1 19407442 | -4 .8159 | 1 .91E-05 0 .001236399 |
| PLLP | -0 .982259848 | 6 .788275507 | -4 .8132 | 1 .93E-05 0 .001238477 |
| BBIP1 | -0 .788367716 | 7 .573456137 | -4 .8122 | 1 .93E-05 0 .001238477 |
| DCUN1D1 | -0 .597430974 | 7 .019466401 | -4 .81 | 1 .95E-05 0 .001240976 |
| ADRM1 | 0 .734807058 | 9 .29876949 | 4 .80604 | 1 .97E-05 0 .001250381 |
| CTSB | 0 .910691414 | 13 .0813418 | 4 .80054 | 2 .01E-05 0 .001259683 |
| CDC25B | 0 .696348811 | 8 .462222545 | 4 .79933 | 2 .02E-05 0 .001259683 |
| TNS1 | -0 .85267537 | 10 .4996759 | -4 .7991 | 2 .02E-05 0 .001259683 |
| FAIM3 | 1 .412903846 | 8 .325676944 | 4 .78937 | 2 .08E-05 0 .001292654 |
| OSBPL8 | -0 .9579212 | 9 .077219935 | -4 .788 | 2 .09E-05 0 .001292654 |
| MFHAS1 | 0 .861212042 | 7 .590591529 | 4 .76831 | 2 .23E-05 0 .001370564 |
| NTRK2 | -0 .8510215 | 9 .027454643 | -4 .755 | 2 .33E-05 0 .001418529 |
| AURKA | 0 .81493252 | 8 .298670829 | 4 .7546 | 2 .33E-05 0 .001418529 |
| C12orf29 | -0 .618693084 | 6 .343602935 | -4 .7474 | 2 .38E-05 0 .001444945 |
| SLC52A2 | 0 .883554375 | 8 .899167932 | 4 .73346 | 2 .49E-05 0 .001503782 |
| RPS7 | -0 .945552177 | 1 1 .63040029 | -4 .729 | 2 .53E-05 0 .001511909 |
| SH3BGRL3 | 0 .734644285 | 10 .7911383 | 4 .7288 | 2 .53E-05 0 .001511909 |
| DLGAP5 | 0 .788134102 | 5 .270908382 | 4 .72127 | 2 .59E-05 0 .00154164 |
| ACOT7 | 0 .83995647 | 9 .29985892 | 4 .71902 | 2 .61E-05 0 .001545499 |
| SLC38A2 | -0 .797388664 | 10 .35649295 | -4 .7162 | 2 .64E-05 0 .001545737 |
| DMD | -0 .626718756 | 7 .695052608 | -4 .716 | 2 .64E-05 0 .001545737 |
| PBX1 | -0 .696125973 | 8 .447688671 | -4 .7031 | 2 .75E-05 0 .001600213 |
| NUSAP1 | 0 .916498426 | 8 .619720572 | 4 .70236 | 2 .75E-05 0 .001600213 |
| WARS | 1 .471688788 | 10 .21233238 | 4 .70039 | 2 .77E-05 0 .00160297 |
| DSG2 | -1 . 120434672 | 5 .723759302 | -4 .69 | 2 .87E-05 0 .001649482 |
| CLCN7 | 0 .603082486 | 10 .2437578 | 4 .68657 | 2 .90E-05 0 .001660329 |
| SSPN | -0 .66909771 | 8 .03753325 | -4 .6829 | 2 .93E-05 0 .001672271 |
| SCARB1 | 0 .859615719 | 9 .3192542 | 4 .68082 | 2 .95E-05 0 .00167593 |

| EMR2 | 0 .752410113 | 5 .849635221 | 4 .67149 | 3 .04E-05 0 .001719048 |
| --- | --- | --- | --- | --- |
| CRYM | -0 .784166963 | 7 .440603828 | -4 .6693 | 3 .06E-05 0 .001723301 |
| CFL1 | 0 .476365912 | 12 .48467956 | 4 .66722 | 3 .08E-05 0 .001727161 |
| SLC7A5 | 1 . 144698972 | 9 .269464308 | 4 .66143 | 3 . 14E-05 0 .001751721 |
| PIK3CB | -0 .474640479 | 7 .837307661 | -4 .6591 | 3 . 16E-05 0 .001757157 |
| CLDN7 | -0 .825520523 | 5 .394744688 | -4 .6532 | 3 .23E-05 0 .001782739 |
| PTTG1 | 0 .823354346 | 9 .032597702 | 4 .64887 | 3 .27E-05 0 .001797161 |
| DENND4B | 0 .767589381 | 7 .385805682 | 4 .64793 | 3 .28E-05 0 .001797161 |
| GHR | -0 .971318916 | 5 .079396814 | -4 .6436 | 3 .33E-05 0 .001814387 |
| LIPT1 | -0 .530087561 | 6 .231066929 | -4 .6419 | 3 .34E-05 0 .001816313 |
| NFAT5 | -0 .726386601 | 8 .000207864 | -4 .6355 | 3 .41E-05 0 .001846166 |
| LIMCH1 | -0 .602311583 | 7 .332793562 | -4 .6321 | 3 .45E-05 0 .001857808 |
| NUAK1 | -0 .829898438 | 7 .240721873 | -4 .6245 | 3 .53E-05 0 .00188836 |
| CLDN10 | -1 . 158483309 | 6 . 17383101 | -4 .6244 | 3 .54E-05 0 .00188836 |
| PJA2 | -0 .535108905 | 8 .453068025 | -4 .6213 | 3 .57E-05 0 .001889687 |
| GRN | 0 .639382985 | 12 .21733368 | 4 .62083 | 3 .58E-05 0 .001889687 |
| G6PC3 | 0 .659822417 | 9 .418400452 | 4 .62018 | 3 .58E-05 0 .001889687 |
| ECI1 | -0 .676051386 | 7 .69001507 | -4 .6131 | 3 .67E-05 0 .001918198 |
| COBL | -1 . 156624917 | 6 .224282095 | -4 .611 | 3 .69E-05 0 .001918198 |
| RPS5 | -0 .586387921 | 1 1 .42087245 | -4 .6104 | 3 .70E-05 0 .001918198 |
| ECHDC2 | -0 .724445973 | 7 .566452913 | -4 .6103 | 3 .70E-05 0 .001918198 |
| MICB | 1 .243130684 | 6 .036258719 | 4 .60768 | 3 .73E-05 0 .001921919 |
| BBX | -0 .701809796 | 8 .666244457 | -4 .6071 | 3 .74E-05 0 .001921919 |
| LMAN2L | -0 .507065322 | 6 .67719845 | -4 .6044 | 3 .77E-05 0 .001930831 |
| KCNK5 | -0 .564805173 | 6 .46528386 | -4 .5971 | 3 .86E-05 0 .001966317 |
| HINT1 | -0 .820348912 | 1 1 .22776927 | -4 .5961 | 3 .87E-05 0 .001966317 |
| ACTR1B | -0 .539019587 | 8 .94254119 | -4 .5919 | 3 .92E-05 0 .001984302 |
| RPL15 | -0 .588241038 | 12 .55967609 | -4 .5897 | 3 .95E-05 0 .001984302 |
| RNASEH2A | 0 .521193834 | 7 .652546366 | 4 .58948 | 3 .95E-05 0 .001984302 |
| NR1D2 | -1 .009711039 | 6 .662155185 | -4 .582 | 4 .05E-05 0 .002015154 |
| CCNB1 | 0 .921218547 | 6 .525523047 | 4 .58046 | 4 .07E-05 0 .002015154 |
| AP2S1 | 0 .54466157 | 1 1 .75202743 | 4 .5802 | 4 .07E-05 0 .002015154 |
| CDC37L1 | -0 .515559013 | 5 .566720614 | -4 .5797 | 4 .08E-05 0 .002015154 |
| IL6 | 0 .942587049 | 7 .25147657 | 4 .57825 | 4 . 10E-05 0 .002016384 |
| MMP1 | 2 .731681683 | 5 . 150270237 | 4 .57501 | 4 . 14E-05 0 .002029312 |
| EMC8 | 0 .498764488 | 7 .764144749 | 4 .56848 | 4 .23E-05 0 .002063261 |
| RPS17 | -0 .840880099 | 12 .95914954 | -4 .5674 | 4 .24E-05 0 .002063261 |
| ATP6AP1 | 0 .440998401 | 9 .961093717 | 4 .56577 | 4 .26E-05 0 .002065762 |
| PAMR1 | -1 .023420668 | 6 .466968789 | -4 .5645 | 4 .28E-05 0 .002066063 |
| DRAP1 | 0 .838691802 | 8 .265741984 | 4 .55904 | 4 .36E-05 0 .002094411 |
| TCIRG1 | 0 .862909241 | 7 .0418943 | 4 .55013 | 4 .48E-05 0 .002143859 |
| C2 | 1 .556743259 | 8 . 14699348 | 4 .54931 | 4 .49E-05 0 .002143859 |
| SMARCA2 | -0 .72672001 | 9 .650197916 | -4 .5471 | 4 .52E-05 0 .002150493 |
| EPB41L4B | -0 .650683977 | 7 .309791375 | -4 .5449 | 4 .56E-05 0 .002157742 |
| ZNF148 | -0 .384353127 | 8 .890122209 | -4 .5376 | 4 .66E-05 0 .002200076 |
| FAM134B | -0 .60404541 | 7 .8787268 | -4 .5284 | 4 .80E-05 0 .002252612 |
| CHRDL1 | -1 .260473538 | 6 .328564746 | -4 .5278 | 4 .81E-05 0 .002252612 |
| WIF1 | -1 .370577603 | 4 .304542476 | -4 .5225 | 4 .89E-05 0 .002282767 |
| EIF4EBP1 | 0 .832096386 | 7 .748772894 | 4 .51861 | 4 .95E-05 0 .002302484 |
| AHNAK2 | -1 .264867569 | 9 . 182004271 | -4 .5131 | 5 .04E-05 0 .002334182 |
| GABRP | -0 .729301292 | 5 .710358505 | -4 .5088 | 5 . 1 1E-05 0 .002358163 |
| PPP1CB | -0 .739435043 | 8 .298180793 | -4 .5068 | 5 . 14E-05 0 .002358578 |
| RPL35A | -0 .730124905 | 10 .66811461 | -4 .5064 | 5 . 15E-05 0 .002358578 |
| PIK3R1 | -0 .665510327 | 9 .832583086 | -4 .5044 | 5 . 18E-05 0 .002358578 |
| TMEM57 | 0 .797459876 | 6 .255515017 | 4 .50411 | 5 . 19E-05 0 .002358578 |
| CEP55 | 1 .225851509 | 5 .558882787 | 4 .50082 | 5 .24E-05 0 .002374796 |

| COTL1 | 1 .081865619 | 8 .776156735 | 4 .49387 | 5 .36E-05 0 .002418998 |
| --- | --- | --- | --- | --- |
| NISCH | -0 .464918564 | 8 .773850125 | -4 .4924 | 5 .38E-05 0 .002421545 |
| GDI2 | -0 .767272311 | 10 .46283736 | -4 .4851 | 5 .51E-05 0 .002469143 |
| REPIN1 | 0 .426992106 | 8 .637566867 | 4 .48348 | 5 .54E-05 0 .002473372 |
| RXRA | -0 .637954784 | 9 .353142778 | -4 .4816 | 5 .57E-05 0 .002479558 |
| PAWR | -0 .586183302 | 7 .998136529 | -4 .4761 | 5 .67E-05 0 .002514115 |
| BRD3 | -0 .500250276 | 9 .427809185 | -4 .4721 | 5 .74E-05 0 .002533373 |
| LGALS3BP | 0 .782165505 | 10 .78677062 | 4 .47146 | 5 .75E-05 0 .002533373 |
| TFPT | 0 .703163437 | 7 .235298248 | 4 .46188 | 5 .93E-05 0 .002596477 |
| MTUS1 | -0 .77547343 | 9 .335958465 | -4 .4615 | 5 .94E-05 0 .002596477 |
| MMP3 | 1 .624222846 | 5 .916948733 | 4 .4591 | 5 .98E-05 0 .002607026 |
| CCL18 | 1 .977585345 | 9 .80899756 | 4 .45004 | 6 . 16E-05 0 .002673641 |
| PSMC4 | 0 .786859132 | 7 .531936008 | 4 .44692 | 6 .22E-05 0 .002690818 |
| RPL31 | -0 .67293399 | 12 .03813398 | -4 .4407 | 6 .34E-05 0 .002734714 |
| CKAP4 | 0 .569745458 | 10 .85934851 | 4 .43684 | 6 .42E-05 0 .002758996 |
| VPS26A | -0 .751158557 | 8 .561428725 | -4 .4344 | 6 .47E-05 0 .002771006 |
| FRZB | -1 .395687409 | 8 . 104657626 | -4 .4208 | 6 .75E-05 0 .002882285 |
| ARMC9 | 0 .94173801 | 6 .791834105 | 4 .41435 | 6 .89E-05 0 .002927515 |
| TNFRSF12A | 0 .875685797 | 7 .069865937 | 4 .41377 | 6 .91E-05 0 .002927515 |
| HIBCH | -0 .964005814 | 7 .945422074 | -4 .4089 | 7 .01E-05 0 .002962563 |
| DLC1 | -0 .778481411 | 9 .438362812 | -4 .4065 | 7 .07E-05 0 .002968972 |
| AEN | 0 .781842457 | 6 .975358555 | 4 .40612 | 7 .07E-05 0 .002968972 |
| DIXDC1 | -1 . 169726023 | 5 .839201568 | -4 .4012 | 7 . 19E-05 0 .003002393 |
| H2AFX | 0 .532241955 | 9 .431475593 | 4 .39984 | 7 .22E-05 0 .003002393 |
| CCK | 0 .993404194 | 4 .020107349 | 4 .3994 | 7 .23E-05 0 .003002393 |
| HMOX1 | 0 .847844213 | 8 .088255168 | 4 .39746 | 7 .27E-05 0 .003010889 |
| RAPGEF5 | -0 .695765507 | 7 .758585298 | -4 .3925 | 7 .38E-05 0 .00304791 |
| EPHX2 | -1 .322808947 | 5 .886006931 | -4 .39 | 7 .44E-05 0 .00306197 |
| SCAMP2 | 0 .464464313 | 8 .792972367 | 4 .38536 | 7 .55E-05 0 .00309497 |
| RPS10L | -0 .871488331 | 7 .329141472 | -4 .3846 | 7 .57E-05 0 .00309497 |
| SORBS2 | -1 .036986602 | 7 .337380349 | -4 .3783 | 7 .72E-05 0 .003146362 |
| LRRC1 | -0 .640167986 | 7 .686081929 | -4 .3761 | 7 .78E-05 0 .003158454 |
| TRPV2 | 0 .941415054 | 8 .057808427 | 4 .37481 | 7 .81E-05 0 .00316065 |
| PURA | -0 .506942022 | 9 .517809536 | -4 .3676 | 7 .99E-05 0 .00321925 |
| CCL27 | -1 .706359149 | 7 .616407047 | -4 .3669 | 8 .00E-05 0 .00321925 |
| TPI1 | 0 .422922092 | 1 1 .84548999 | 4 .3658 | 8 .03E-05 0 .003220415 |
| SLC25A44 | 0 .656593405 | 8 .721820154 | 4 .36347 | 8 .09E-05 0 .003225426 |
| LUZP1 | 1 .064991077 | 9 .371386584 | 4 .36328 | 8 . 10E-05 0 .003225426 |
| PKM | 0 .715054193 | 1 1 .04926943 | 4 .36103 | 8 . 15E-05 0 .003238063 |
| ZNF652 | -1 .210530863 | 4 .658010381 | -4 .3596 | 8 . 19E-05 0 .003242258 |
| FANCI | 0 .671982388 | 7 .499672144 | 4 .35791 | 8 .23E-05 0 .003249396 |
| DOCK1 | -0 .472564328 | 7 .604591513 | -4 .3561 | 8 .28E-05 0 .003258024 |
| FAM189A2 | -0 .77806614 | 7 .905146016 | -4 .3522 | 8 .38E-05 0 .003287289 |
| CLPP | 0 .540750578 | 8 .024894322 | 4 .3484 | 8 .48E-05 0 .00330523 |
| SHOC2 | -0 .72862482 | 7 .758677155 | -4 .3482 | 8 .49E-05 0 .00330523 |
| CGRRF1 | -0 .611 1 15413 | 5 .827419457 | -4 .3475 | 8 .51E-05 0 .00330523 |
| TCF7L2 | -0 .676843351 | 9 .884639248 | -4 .3415 | 8 .67E-05 0 .003358562 |
| ABCA5 | -1 .21861513 | 6 .889394954 | -4 .3364 | 8 .81E-05 0 .003401341 |
| POTEKP | 0 .697851042 | 7 .540551207 | 4 .33531 | 8 .84E-05 0 .00340304 |
| NAB1 | -0 .500778487 | 8 .595537104 | -4 .3322 | 8 .93E-05 0 .003426273 |
| DERL1 | 0 .719546006 | 8 .27993595 | 4 .32907 | 9 .02E-05 0 .003449229 |
| CYP3A5 | -0 .697835738 | 6 .941870473 | -4 .3272 | 9 .07E-05 0 .003458468 |
| FZD7 | -0 .918326471 | 7 .983466713 | -4 .3256 | 9 . 1 1E-05 0 .003465429 |
| NDUFS4 | -0 .67715399 | 8 .669007055 | -4 .3177 | 9 .34E-05 0 .003542044 |
| SLC5A3 | -0 .823315885 | 8 .77914416 | -4 .3139 | 9 .45E-05 0 .003573531 |
| PLAU | 0 .832603779 | 8 . 158709004 | 4 .3117 | 9 .52E-05 0 .003587668 |

| FOXO3 | -0 .631906543 | 8 .756411743 | -4 .3083 | 9 .62E-05 0 .003614604 |
| --- | --- | --- | --- | --- |
| POLR2L | 0 .580851053 | 10 .32657162 | 4 .3074 | 9 .65E-05 0 .003614604 |
| TFAP2B | -1 .806783712 | 7 .291432262 | -4 .3029 | 9 .79E-05 0 .003654956 |
| TXNRD1 | 0 .713556694 | 7 .895868994 | 4 .30185 | 9 .82E-05 0 .003656209 |
| SHC1 | 0 .791745353 | 10 .2043483 | 4 .30058 | 9 .86E-05 0 .003659932 |
| AQP5 | -0 .884829081 | 6 .535116287 | -4 .2968 | 9 .98E-05 0 .003673865 |
| TNFSF9 | 0 .598059102 | 6 .296395036 | 4 .29639 | 9 .99E-05 0 .003673865 |
| SLC35A1 | -1 .001181707 | 7 .384714805 | -4 .2956 | 0 .0001 0 .003673865 |
| RPL39 | -0 .539432601 | 1 1 .68833843 | -4 .295 | 0 .0001 0 .003673865 |
| IMPDH2 | -0 .651654974 | 10 .40280014 | -4 .293 | 0 .0001 0 .003673865 |
| GDF15 | 2 .423134776 | 8 .056691835 | 4 .29295 | 0 .0001 0 .003673865 |
| FBL | -0 .587688381 | 10 . 17601104 | -4 .2928 | 0 .0001 0 .003673865 |
| TTC39A | 0 .760689497 | 6 .905866889 | 4 .29067 | 0 .0001 0 .003675133 |
| NIPA2 | 0 .462548277 | 9 .330669699 | 4 .29013 | 0 .0001 0 .003675133 |
| DPYSL3 | -0 .810185319 | 8 .384847393 | -4 .2899 | 0 .0001 0 .003675133 |
| GZMB | 2 .093879192 | 6 .550010859 | 4 .28638 | 0 .0001 0 .00370578 |
| NET1 | -1 .002215752 | 8 .237171982 | -4 .2838 | 0 .0001 0 .003721573 |
| BIN3 | 0 .689280844 | 7 .831906373 | 4 .28303 | 0 .0001 0 .003721573 |
| PLSCR4 | -1 .012874419 | 7 . 166577242 | -4 .2823 | 0 .0001 0 .003721573 |
| ANG | -0 .701055021 | 6 .597728862 | -4 .2785 | 0 .00011 0 .003747246 |
| TK1 | 0 .687632736 | 7 .274439122 | 4 .27827 | 0 .00011 0 .003747246 |
| PHLDA2 | 1 . 109833908 | 8 .81044213 | 4 .27458 | 0 .00011 0 .003774375 |
| ATP6V0C | 0 .46429011 1 | 1 1 .60619601 | 4 .27417 | 0 .00011 0 .003774375 |
| GBAP1 | 0 .810921398 | 8 .31049275 | 4 .26684 | 0 .00011 0 .003845317 |
| FABP4 | -1 .670847102 | 6 .337270434 | -4 .2664 | 0 .00011 0 .003845317 |
| USP33 | -0 .563897499 | 8 .330943392 | -4 .2623 | 0 .00011 0 .00388413 |
| C12orf5 | 0 .745510925 | 6 .972855609 | 4 .25994 | 0 .00011 0 .003888075 |
| FOXC1 | -0 .856137555 | 7 .454198403 | -4 .2599 | 0 .00011 0 .003888075 |
| TRIP13 | 0 .447194639 | 6 .875443468 | 4 .25916 | 0 .00011 0 .003888075 |
| ECM1 | 1 . 185246957 | 8 .921741863 | 4 .25844 | 0 .00011 0 .003888075 |
| TMEM168 | -0 .753342462 | 7 .377038979 | -4 .257 | 0 .00011 0 .003895373 |
| HIST1H2BH | 1 .369246706 | 4 .736072032 | 4 .25374 | 0 .00011 0 .00391557 |
| S100A11 | 0 .75949109 | 1 1 .57174494 | 4 .25354 | 0 .00011 0 .00391557 |
| LPAR1 | -0 .537563981 | 8 .836422946 | -4 .2515 | 0 .00011 0 .00392937 |
| GNPDA1 | 0 .50373288 | 7 .867951182 | 4 .24886 | 0 .00012 0 .003951672 |
| TRPC1 | -0 .572347208 | 6 .661813055 | -4 .2371 | 0 .00012 0 .004088729 |
| AKAP1 | -0 .53388738 | 9 .45946817 | -4 .2344 | 0 .00012 0 .004104651 |
| CCT4 | -0 .767118516 | 9 .544493514 | -4 .2341 | 0 .00012 0 .004104651 |
| PDGFD | -1 .07348758 | 7 .451886527 | -4 .2329 | 0 .00012 0 .004109024 |
| ZFP36L1 | -0 .57556671 | 9 .870599908 | -4 .2289 | 0 .00012 0 .004148856 |
| RPS24 | -0 .692651898 | 1 1 .46237747 | -4 .2217 | 0 .00013 0 .004231238 |
| CHST11 | 0 .878756122 | 6 .592856783 | 4 .22063 | 0 .00013 0 .00423471 |
| CHD9 | -0 .742670821 | 8 .241296194 | -4 .2129 | 0 .00013 0 .004326756 |
| TCN2 | 0 .812183103 | 6 .749287453 | 4 .21186 | 0 .00013 0 .004328806 |
| HIST1H2BD | 0 .76939219 | 8 .618541067 | 4 .20737 | 0 .00013 0 .004373646 |
| IMPDH1 | 0 .680292932 | 8 .581276307 | 4 .20685 | 0 .00013 0 .004373646 |
| RSBN1 | -0 .796449129 | 6 .037307607 | -4 .2053 | 0 .00013 0 .004383581 |
| ZNF12 | -0 .553218994 | 7 .355379981 | -4 .2028 | 0 .00013 0 .004405695 |
| HADH | -0 .677653941 | 8 .897766081 | -4 .2011 | 0 .00013 0 .00440718 |
| P2RX5 | 0 .704549289 | 5 .497958849 | 4 .20102 | 0 .00013 0 .00440718 |
| PBX3 | -0 .70859558 | 6 .790125254 | -4 . 1925 | 0 .00014 0 .004513389 |
| LGMN | 1 . 124247586 | 8 .282242745 | 4 . 1881 | 0 .00014 0 .004563834 |
| TMED1 | 0 .797230434 | 7 .943764997 | 4 . 18092 | 0 .00014 0 .004645176 |
| TMEM43 | -0 .38806867 | 9 .012618349 | -4 . 1805 | 0 .00014 0 .004645176 |
| IRAK1 | 0 .65569494 | 9 .654670215 | 4 . 17991 | 0 .00014 0 .004645176 |
| PTTG3P | 1 .009826769 | 6 .504058153 | 4 . 17152 | 0 .00015 0 .00474857 |

| CDHR1 | -0 .923509614 | 7 .549514486 | -4 . 1712 | 0 .00015 | 0 .00474857 |
| --- | --- | --- | --- | --- | --- |
| SUCLG2 | -0 .643604966 | 9 .436845712 | -4 . 1675 | 0 .00015 | 0 .004790462 |
| GATM | -1 . 130253741 | 8 .355755852 | -4 . 1661 | 0 .00015 | 0 .004798817 |
| FERMT2 | -0 .521125459 | 8 .845621428 | -4 . 163 | 0 .00015 | 0 .004833666 |
| CSNK2B | 0 .439423395 | 10 .36223829 | 4 . 16152 | 0 .00015 | 0 .004843129 |
| TM7SF3 | -1 .218757517 | 7 .020033543 | -4 . 1599 | 0 .00015 | 0 .00485571 |
| GTPBP2 | 0 .597348358 | 7 .388341029 | 4 . 15839 | 0 .00015 | 0 .004865617 |
| PUM2 | -0 .420667833 | 9 .570081449 | -4 . 1549 | 0 .00016 | 0 .004906401 |
| PDGFRL | -1 .678368609 | 5 .971209207 | -4 . 1533 | 0 .00016 | 0 .004918665 |
| MAD1L1 | 0 .978298706 | 7 .848913262 | 4 . 15202 | 0 .00016 | 0 .00491954 |
| CYBA | 0 .85667761 | 8 .911440847 | 4 . 15159 | 0 .00016 | 0 .00491954 |
| NFU1 | -0 .791709989 | 6 .721807216 | -4 . 1506 | 0 .00016 | 0 .00492178 |
| SPP1 | 2 .659571169 | 6 .541994634 | 4 . 14891 | 0 .00016 | 0 .004932778 |
| FH | 0 .644945041 | 10 .48567645 | 4 . 14771 | 0 .00016 | 0 .004932778 |
| TOP1 | 0 .622303114 | 9 . 144058392 | 4 . 14716 | 0 .00016 | 0 .004932778 |
| TOM1 | 0 .605942498 | 8 .214270637 | 4 . 14671 | 0 .00016 | 0 .004932778 |
| GPD1L | -0 .691872528 | 7 .474184374 | -4 . 1455 | 0 .00016 | 0 .00493861 |
| MYO1E | 0 .609225161 | 6 .4276448 | 4 . 14442 | 0 .00016 | 0 .004943424 |
| TUBBP5 | 0 .482855901 | 5 .420651899 | 4 . 14341 | 0 .00016 | 0 .004946846 |
| HNRNPA3 | -0 .549572597 | 9 .693632001 | -4 . 1426 | 0 .00016 | 0 .004947471 |
| HBEGF | 0 .915684428 | 7 .89760591 | 4 . 14038 | 0 .00016 | 0 .004968996 |
| SNRPA1 | 0 .499350018 | 8 .781808335 | 4 . 13842 | 0 .00016 | 0 .004987091 |
| FKBP11 | 1 .237767789 | 8 .955036306 | 4 . 13583 | 0 .00016 | 0 .005015087 |
| DDX49 | 0 .413799726 | 8 .960262461 | 4 . 12235 | 0 .00017 | 0 .005215835 |
| ECHDC3 | -0 .608556115 | 6 .836225927 | -4 . 1 178 | 0 .00017 | 0 .00526811 |
| EFNA5 | -0 .473619484 | 7 .26862138 | -4 . 1 174 | 0 .00017 | 0 .00526811 |
| DDA1 | 0 .551254388 | 6 .804980977 | 4 . 1 1678 | 0 .00017 | 0 .00526811 |
| LAMTOR2 | 0 .712428519 | 8 .066205399 | 4 . 1 1452 | 0 .00018 | 0 .005280225 |
| UFSP2 | -0 .671135636 | 7 .330403968 | -4 . 1 142 | 0 .00018 | 0 .005280225 |
| RYK | -0 .524957355 | 8 .66060845 | -4 . 1 137 | 0 .00018 | 0 .005280225 |
| MMP7 | -1 .2522454 | 6 .808412984 | -4 . 1 1 15 | 0 .00018 | 0 .005303159 |
| NCOA1 | -0 .400026958 | 9 .798037413 | -4 . 1087 | 0 .00018 | 0 .005331521 |
| SOBP | -1 .014006843 | 6 .049660457 | -4 . 1083 | 0 .00018 | 0 .005331521 |
| NFKBIB | 0 .561728384 | 7 .599803818 | 4 . 10556 | 0 .00018 | 0 .005363598 |
| ATP6V1B1 | -1 .064612746 | 4 .605342325 | -4 . 1 | 0 .00018 | 0 .005443864 |
| FEN1 | 0 .609197143 | 8 .299185228 | 4 .09307 | 0 .00019 | 0 .005545388 |
| GGH | 0 .951755405 | 7 .489604758 | 4 .09246 | 0 .00019 | 0 .005545388 |
| PLTP | 0 .588430063 | 9 .573744474 | 4 .08921 | 0 .00019 | 0 .005560814 |
| VPS51 | -0 .733892722 | 9 .485895792 | -4 .0888 | 0 .00019 | 0 .005560814 |
| INHBB | -1 .313706393 | 7 . 184075744 | -4 .0888 | 0 .00019 | 0 .005560814 |
| OXR1 | -1 .045611332 | 7 .068947985 | -4 .0885 | 0 .00019 | 0 .005560814 |
| CRISP3 | -1 .350658688 | 3 .906653269 | -4 .0838 | 0 .00019 | 0 .005630145 |
| THYN1 | -0 .574784182 | 7 .08105666 | -4 .0795 | 0 .0002 | 0 .005691933 |
| SETMAR | -0 .842411692 | 5 .723373657 | -4 .0745 | 0 .0002 | 0 .005766508 |
| ASMTL | -0 .526587791 | 9 .321190905 | -4 .0709 | 0 .0002 | 0 .005816753 |
| FOXO1 | -0 .608366712 | 8 .848136012 | -4 .0697 | 0 .0002 | 0 .005825489 |
| EIF4E2 | 0 .451893112 | 9 .446138989 | 4 .0675 | 0 .0002 | 0 .00585107 |
| ALDOA | 0 .532080695 | 12 .94720687 | 4 .06625 | 0 .0002 | 0 .005860172 |
| GATAD2A | 0 .421649662 | 8 .495488996 | 4 .05808 | 0 .00021 | 0 .005995593 |
| PHKB | -0 .477184242 | 7 .630311045 | -4 .057 | 0 .00021 | 0 .006002043 |
| CHFR | 0 .434594261 | 8 .069953731 | 4 .05013 | 0 .00021 | 0 .006109475 |
| RPS10 | -0 .50832125 | 13 .95321356 | -4 .0497 | 0 .00021 | 0 .006109475 |
| PSMB10 | 0 .764581089 | 7 .662170123 | 4 .04837 | 0 .00022 | 0 .006121277 |
| BAI2 | 0 .803458162 | 5 .093187236 | 4 .04489 | 0 .00022 | 0 .006152233 |
| COX7C | -0 .639122957 | 1 1 . 1 1365518 | -4 .0444 | 0 .00022 | 0 .006152233 |
| HMGB3P1 | 0 .562776527 | 7 . 139782305 | 4 .04392 | 0 .00022 | 0 .006152233 |

| POU2F3 | -0 .923714282 | 6 . 143066715 | -4 .0438 | 0 .00022 | 0 .006152233 |
| --- | --- | --- | --- | --- | --- |
| PON3 | -0 .83400982 | 6 .860237819 | -4 .0424 | 0 .00022 | 0 .006164947 |
| GPRASP1 | -0 .45343493 | 5 .631366945 | -4 .0385 | 0 .00022 | 0 .006226033 |
| PTPLB | -0 .581636101 | 8 . 135701616 | -4 .037 | 0 .00022 | 0 .006239004 |
| ISOC1 | -0 .74136711 1 | 7 . 185344344 | -4 .0337 | 0 .00023 | 0 .006289383 |
| GSTA4 | -0 .559961596 | 8 .915056963 | -4 .0327 | 0 .00023 | 0 .006294653 |
| PRC1 | 0 .880737709 | 7 . 128903399 | 4 .02875 | 0 .00023 | 0 .006345054 |
| RPL18 | -0 .353249646 | 1 1 .8254845 | -4 .0287 | 0 .00023 | 0 .006345054 |
| PI4K2A | 0 .375325996 | 8 .486194194 | 4 .02668 | 0 .00023 | 0 .006369684 |
| CGGBP1 | 0 .3713446 | 8 .566265084 | 4 .0248 | 0 .00023 | 0 .006385572 |
| VCL | -0 .574717905 | 9 .42238088 | -4 .0244 | 0 .00023 | 0 .006385572 |
| BCL6 | -0 .528670556 | 8 .953480512 | -4 .0219 | 0 .00023 | 0 .006413021 |
| IGBP1 | -0 .741851884 | 8 .491066296 | -4 .0216 | 0 .00023 | 0 .006413021 |
| DDX56 | 0 .381458715 | 8 .351512257 | 4 .018 | 0 .00024 | 0 .006470072 |
| GTPBP8 | -0 .399711586 | 7 .97428606 | -4 .0115 | 0 .00024 | 0 .006586311 |
| HJURP | 1 .071592583 | 5 .965885261 | 4 .01051 | 0 .00024 | 0 .006586353 |
| YPEL5 | -0 .635184011 | 8 .059351603 | -4 .0101 | 0 .00024 | 0 .006586353 |
| NR3C2 | -0 .856076257 | 5 .496507181 | -4 .0082 | 0 .00024 | 0 .006609982 |
| EHD1 | 0 .522871949 | 9 .940705308 | 4 .00515 | 0 .00025 | 0 .006657057 |
| PDIA3 | 0 .616570238 | 10 .06713557 | 4 .00131 | 0 .00025 | 0 .006721233 |
| CENPN | 0 .471384474 | 6 .024712235 | 3 .99758 | 0 .00025 | 0 .006776504 |
| CCNB1IP1 | -0 .585245315 | 8 . 183182002 | -3 .9966 | 0 .00025 | 0 .006776504 |
| C17orf62 | 0 .575150857 | 8 .232700916 | 3 .99559 | 0 .00025 | 0 .006776504 |
| DTL | 0 .862578931 | 6 .031636312 | 3 .99533 | 0 .00025 | 0 .006776504 |
| CCNG2 | -0 .60674891 | 8 .565865734 | -3 .9951 | 0 .00025 | 0 .006776504 |
| DYSF | 0 .662770285 | 6 .325670476 | 3 .99254 | 0 .00026 | 0 .00681614 |
| STX3 | 0 .521373247 | 7 .65554844 | 3 .98679 | 0 .00026 | 0 .006922143 |
| KLF5 | -1 .058144062 | 10 .30304627 | -3 .9851 | 0 .00026 | 0 .006943355 |
| PARD3 | -0 .706559909 | 9 .027352453 | -3 .9844 | 0 .00026 | 0 .006944034 |
| AOC3 | -0 .980877426 | 6 .480528462 | -3 .9748 | 0 .00027 | 0 .007135243 |
| CYP17A1 | 0 .847214272 | 7 .345613042 | 3 .97375 | 0 .00027 | 0 .007142682 |
| IFT81 | 1 .045436732 | 6 .08596259 | 3 .97113 | 0 .00027 | 0 .007185079 |
| S100A1 | 1 .93609751 | 10 . 17160151 | 3 .96819 | 0 .00028 | 0 .007225213 |
| CSTB | 0 .658768271 | 1 1 .35207902 | 3 .96793 | 0 .00028 | 0 .007225213 |
| ADIRF | -1 .296965817 | 7 .72205736 | -3 .9664 | 0 .00028 | 0 .007244419 |
| COL4A2 | 0 .836668407 | 10 .02986887 | 3 .96488 | 0 .00028 | 0 .007262267 |
| COL4A5 | -1 .270525818 | 6 .061001667 | -3 .964 | 0 .00028 | 0 .00726693 |
| EN1 | -0 .941276626 | 6 .872447534 | -3 .9629 | 0 .00028 | 0 .007275806 |
| JAG1 | -0 .597743869 | 9 .826978892 | -3 .96 | 0 .00028 | 0 .007325022 |
| CENPF | 0 .813422871 | 7 .994826218 | 3 .95768 | 0 .00028 | 0 .007362344 |
| SLC25A38 | -0 .59070796 | 7 .652821351 | -3 .9535 | 0 .00029 | 0 .007441452 |
| INPP5A | -0 .567963313 | 7 .968435016 | -3 .9506 | 0 .00029 | 0 .007492298 |
| ADAMTS5 | -1 . 158458031 | 6 .44956836 | -3 .9486 | 0 .00029 | 0 .007521421 |
| ACAT2 | -0 .518901636 | 8 .287239506 | -3 .9466 | 0 .00029 | 0 .007552171 |
| EIF3E | -0 .715234363 | 10 .48475487 | -3 .9439 | 0 .0003 | 0 .007598261 |
| RPL35 | -0 .469622185 | 1 1 . 13337544 | -3 .9375 | 0 .0003 | 0 .007722478 |
| PCYOX1 | -0 .705249167 | 6 .690157505 | -3 .9366 | 0 .0003 | 0 .007722478 |
| PTER | -0 .709836688 | 6 .379416074 | -3 .9357 | 0 .0003 | 0 .007722478 |
| KRT15 | -1 .744519992 | 9 .674697522 | -3 .9354 | 0 .0003 | 0 .007722478 |
| CSDE1 | -0 .482789474 | 10 .77378701 | -3 .9353 | 0 .0003 | 0 .007722478 |
| C18orf8 | 0 .430560192 | 6 .829749402 | 3 .93417 | 0 .00031 | 0 .007732918 |
| ST13 | -0 .660301427 | 10 .60019152 | -3 .9316 | 0 .00031 | 0 .007770214 |
| HOOK2 | -0 .733152923 | 6 .782600498 | -3 .9313 | 0 .00031 | 0 .007770214 |
| WDR46 | 0 .767372812 | 5 .720516692 | 3 .9289 | 0 .00031 | 0 .007810574 |
| LRIG1 | -1 .353652345 | 6 .529597725 | -3 .9253 | 0 .00031 | 0 .007880303 |
| PAIP2B | -0 .835268878 | 5 .927450501 | -3 .9222 | 0 .00032 | 0 .007939338 |

| AEBP1 | 0 .88211 1015 | 9 .657628211 | 3 .92136 | 0 .00032 | 0 .007943821 |
| --- | --- | --- | --- | --- | --- |
| TMEM204 | -0 .733442125 | 8 .969365321 | -3 .9202 | 0 .00032 | 0 .007955993 |
| SLC16A7 | -0 .688223916 | 7 .330476113 | -3 .9194 | 0 .00032 | 0 .007960577 |
| PLEKHA1 | -0 .653008835 | 7 .555894432 | -3 .9176 | 0 .00032 | 0 .007980266 |
| CHIT1 | 1 .976670302 | 6 .783380047 | 3 .91724 | 0 .00032 | 0 .007980266 |
| STAG2 | -0 .635870104 | 9 .75531893 | -3 .9128 | 0 .00033 | 0 .008073523 |
| IGF1R | -1 .032351513 | 9 .754533552 | -3 .9118 | 0 .00033 | 0 .008080246 |
| SLC35A3 | -0 .616421929 | 6 .684570604 | -3 .905 | 0 .00033 | 0 .008233999 |
| CDSN | 2 .57135838 | 8 .744328525 | 3 .8984 | 0 .00034 | 0 .008383055 |
| MMP28 | -1 . 106546538 | 5 . 100914533 | -3 .8973 | 0 .00034 | 0 .00839423 |
| TBCD | 0 .492952831 | 6 .930889096 | 3 .89596 | 0 .00034 | 0 .008412326 |
| EFHD2 | 0 .680190812 | 8 .425794894 | 3 .88991 | 0 .00035 | 0 .008551068 |
| TMED2 | -0 .568341498 | 10 . 10982707 | -3 .8878 | 0 .00035 | 0 .008577865 |
| SLC45A2 | 0 .707866714 | 8 .802682969 | 3 .88759 | 0 .00035 | 0 .008577865 |
| CHPF2 | 0 .388022089 | 8 .618987587 | 3 .88347 | 0 .00036 | 0 .008668879 |
| BAIAP2 | -0 .449777631 | 8 .419133474 | -3 .8813 | 0 .00036 | 0 .008709 |
| DDX50 | -0 .826229196 | 7 .859824418 | -3 .8801 | 0 .00036 | 0 .00872265 |
| DPP3 | 0 .457617695 | 8 .449984077 | 3 .87811 | 0 .00036 | 0 .008748506 |
| ULBP2 | 0 .990175199 | 4 .30203932 | 3 .87789 | 0 .00036 | 0 .008748506 |
| NAALADL1 | -1 .046343283 | 4 .200984121 | -3 .8661 | 0 .00038 | 0 .009048268 |
| ZFAND1 | -0 .634849804 | 6 .667872078 | -3 .863 | 0 .00038 | 0 .009115243 |
| CD34 | -0 .632755643 | 7 .97301669 | -3 .8619 | 0 .00038 | 0 .009115779 |
| SBNO2 | 0 .505057102 | 8 .332316911 | 3 .86174 | 0 .00038 | 0 .009115779 |
| PLXDC2 | -0 .85541966 | 7 .09692018 | -3 .8564 | 0 .00039 | 0 .009246927 |
| NOC4L | 0 .608408816 | 6 .775681504 | 3 .85314 | 0 .00039 | 0 .009306322 |
| RPS21 | -0 .753553577 | 1 1 .25635314 | -3 .853 | 0 .00039 | 0 .009306322 |
| C10orf76 | -0 .503157905 | 7 .468290683 | -3 .8497 | 0 .00039 | 0 .009366016 |
| TMEM147 | 0 .449427435 | 10 .04047066 | 3 .8487 | 0 .0004 | 0 .009366016 |
| NEK2 | 0 .625811501 | 6 .084956269 | 3 .84846 | 0 .0004 | 0 .009366016 |
| CRYZL1 | -0 .450451439 | 6 .561782224 | -3 .8484 | 0 .0004 | 0 .009366016 |
| NDUFA4L2 | 1 .215345377 | 7 .721420552 | 3 .84746 | 0 .0004 | 0 .009374113 |
| ATP13A2 | 0 .923527907 | 6 .636665064 | 3 .84566 | 0 .0004 | 0 .009407433 |
| NEFH | 1 . 1 13169727 | 6 .600391491 | 3 .84277 | 0 .0004 | 0 .009471831 |
| RPS9 | -0 .427141686 | 12 .48494115 | -3 .8404 | 0 .00041 | 0 .00950676 |
| RPS29 | -0 .741767558 | 1 1 .3441509 | -3 .8403 | 0 .00041 | 0 .00950676 |
| IGFBP6 | -0 .794317413 | 7 .310986807 | -3 .8364 | 0 .00041 | 0 .009600167 |
| HK2 | 1 .081977544 | 7 .277446696 | 3 .83307 | 0 .00042 | 0 .009680286 |
| CTNNBL1 | 0 .365497078 | 7 .264792288 | 3 .83184 | 0 .00042 | 0 .009698199 |
| SLC41A3 | -0 .763797166 | 7 .373936733 | -3 .8303 | 0 .00042 | 0 .009724357 |
| ELAC2 | 0 .598139559 | 7 .047522076 | 3 .82606 | 0 .00042 | 0 .009820937 |
| SPSB1 | 0 .563885737 | 7 .285437603 | 3 .82582 | 0 .00042 | 0 .009820937 |
| CDKN1C | -1 .009516597 | 10 .72852907 | -3 .8245 | 0 .00043 | 0 .009840901 |
| LRRC17 | -0 .611499586 | 5 .444516708 | -3 .8204 | 0 .00043 | 0 .009902946 |
| TCEB1 | 0 .591954846 | 9 .298964978 | 3 .8203 | 0 .00043 | 0 .009902946 |
| HPCAL1 | 0 .590741161 | 9 .215266057 | 3 .82006 | 0 .00043 | 0 .009902946 |
| GREM2 | -1 .087713964 | 4 .969131351 | -3 .82 | 0 .00043 | 0 .009902946 |
| BID | 0 .463736734 | 7 .888541338 | 3 .81821 | 0 .00043 | 0 .009938083 |
| PLN | -0 .824226132 | 6 .337219064 | -3 .8144 | 0 .00044 | 0 .010018901 |
| FOXN3 | -0 .477983678 | 10 . 14366038 | -3 .8143 | 0 .00044 | 0 .010018901 |
| CSNK1G2 | 0 .468917708 | 9 .874376147 | 3 .81235 | 0 .00044 | 0 .01004756 |
| TRPM1 | -1 .213186625 | 6 .643854345 | -3 .8121 | 0 .00044 | 0 .01004756 |
| SIRT1 | -0 .625138048 | 6 .482294832 | -3 .8103 | 0 .00044 | 0 .010085608 |
| ZNF14 | -0 .370007236 | 5 .985212015 | -3 .8091 | 0 .00045 | 0 .010090682 |
| GPR124 | -0 .548964775 | 9 . 140433082 | -3 .8089 | 0 .00045 | 0 .010090682 |
| ACAT1 | -0 .710272474 | 8 .077417176 | -3 .8049 | 0 .00045 | 0 .010193237 |
| PYROXD1 | -0 .689509762 | 8 . 100748068 | -3 .8031 | 0 .00045 | 0 .010232681 |

| TNFRSF25 | -0 .587424148 | 8 .567446491 | -3 .8015 | 0 .00046 | 0 .010261994 |
| --- | --- | --- | --- | --- | --- |
| 10-Sep | -0 .682813407 | 8 .69028025 | -3 .7994 | 0 .00046 | 0 .010307007 |
| RPL7 | -0 .632628843 | 12 .9040604 | -3 .7989 | 0 .00046 | 0 .010307007 |
| SYBU | -0 .744539276 | 6 .469771308 | -3 .7966 | 0 .00046 | 0 .010358411 |
| GRB2 | 0 .464698541 | 8 .445486732 | 3 .79538 | 0 .00046 | 0 .010373152 |
| TIMP3 | -0 .852088916 | 10 .53154451 | -3 .7949 | 0 .00047 | 0 .010373152 |
| RPS14 | -0 .508277465 | 1 1 .92821011 | -3 .7938 | 0 .00047 | 0 .01039044 |
| GABARAPL2 | -0 .533005382 | 9 .200661717 | -3 .7923 | 0 .00047 | 0 .010412512 |
| EGFR | -0 .788070711 | 9 .324343987 | -3 .7914 | 0 .00047 | 0 .010412512 |
| HPSE | 1 .001369983 | 5 .900565289 | 3 .79132 | 0 .00047 | 0 .010412512 |
| PALM | -0 .596626421 | 7 .754113363 | -3 .7876 | 0 .00048 | 0 .010511 |
| NRG2 | -0 .75250232 | 7 .005578851 | -3 .7862 | 0 .00048 | 0 .01053612 |
| ACTR10 | -0 .463919258 | 8 .356198087 | -3 .7851 | 0 .00048 | 0 .010553228 |
| SCGB2A2 | -1 .955490479 | 6 .798636682 | -3 .7822 | 0 .00048 | 0 .01062628 |
| METAP2 | -0 .570537039 | 8 .068135638 | -3 .7783 | 0 .00049 | 0 .010730936 |
| VARS | 0 .461320373 | 8 .391499817 | 3 .77728 | 0 .00049 | 0 .010746131 |
| CREB1 | -0 .360633256 | 9 .036769268 | -3 .7766 | 0 .00049 | 0 .010750919 |
| RSL24D1 | -0 .850971953 | 7 .75303209 | -3 .7716 | 0 .0005 | 0 .010891393 |
| ITIH5 | -1 . 1 1812905 | 6 .861877597 | -3 .7708 | 0 .0005 | 0 .010901182 |
| MYBBP1A | 0 .665895852 | 7 . 147298358 | 3 .76952 | 0 .0005 | 0 .010922572 |
| ADD3-AS1 | -0 .739699518 | 4 .566863439 | -3 .7685 | 0 .0005 | 0 .010936844 |
| AURKB | 0 .982386512 | 6 . 1 19361602 | 3 .76229 | 0 .00051 | 0 .011 1 154 |
| CNOT7 | -0 .479795689 | 8 .977130884 | -3 .7619 | 0 .00051 | 0 .011 1 154 |
| HMGA1 | 0 .448054042 | 9 .819354933 | 3 .759 | 0 .00052 | 0 .011 175021 |
| TMEM158 | 1 .462745903 | 6 .397674666 | 3 .75814 | 0 .00052 | 0 .011 175021 |
| TBC1D4 | -0 .854965042 | 8 .67726357 | -3 .7581 | 0 .00052 | 0 .011 175021 |
| SYF2 | -0 .552502749 | 8 .334819924 | -3 .7578 | 0 .00052 | 0 .011 175021 |
| SLC39A7 | 0 .830331412 | 7 .88357164 | 3 .75405 | 0 .00053 | 0 .0112438 |
| SERPINF1 | 0 .774909679 | 1 1 .04431213 | 3 .75342 | 0 .00053 | 0 .0112438 |
| RABIF | 0 .489948991 | 8 .239091507 | 3 .75336 | 0 .00053 | 0 .0112438 |
| ZKSCAN1 | -0 .656604367 | 8 .613500284 | -3 .7531 | 0 .00053 | 0 .0112438 |
| GRIA1 | -1 .079284222 | 5 .032612116 | -3 .7523 | 0 .00053 | 0 .0112438 |
| CACNB4 | -0 .858128816 | 5 .334729373 | -3 .7519 | 0 .00053 | 0 .0112438 |
| PGRMC2 | -0 .655539435 | 8 .464441429 | -3 .7518 | 0 .00053 | 0 .0112438 |
| SOD3 | -0 .784113267 | 7 .957974714 | -3 .7507 | 0 .00053 | 0 .011262279 |
| PDSS1 | 0 .469868867 | 6 .412399097 | 3 .74929 | 0 .00053 | 0 .011277994 |
| ECM2 | -0 .889467934 | 6 .415803902 | -3 .7486 | 0 .00053 | 0 .011277994 |
| TSC22D3 | -0 .6138009 | 9 .519982465 | -3 .748 | 0 .00054 | 0 .011277994 |
| LSAMP | 0 .994847923 | 5 .984020842 | 3 .74796 | 0 .00054 | 0 .011277994 |
| PRR13 | 0 .459577846 | 9 .095830586 | 3 .74636 | 0 .00054 | 0 .011302655 |
| NUP93 | 0 .45894919 | 7 .598412346 | 3 .7461 | 0 .00054 | 0 .011302655 |
| MAP1B | -0 .716708058 | 8 .051272227 | -3 .7424 | 0 .00054 | 0 .011391629 |
| RFC1 | -0 .368056076 | 8 .48039656 | -3 .7424 | 0 .00054 | 0 .011391629 |
| RPL3 | -0 .433619577 | 12 .36099188 | -3 .7391 | 0 .00055 | 0 .011483868 |
| RRBP1 | 0 .469331091 | 10 . 18440251 | 3 .73597 | 0 .00056 | 0 .011569706 |
| MLLT11 | 0 .458784542 | 8 .639061319 | 3 .73548 | 0 .00056 | 0 .011569706 |
| TPM4 | 0 .754594413 | 9 .360638987 | 3 .73323 | 0 .00056 | 0 .01162109 |
| ITGB4 | -0 .686241018 | 9 .026762655 | -3 .7324 | 0 .00056 | 0 .01162109 |
| AQP1 | -0 .893407593 | 10 .34204463 | -3 .7323 | 0 .00056 | 0 .01162109 |
| CAT | -0 .504949203 | 9 .50136161 | -3 .7299 | 0 .00057 | 0 .011687393 |
| KHDRBS1 | -0 .388547525 | 10 .45721484 | -3 .7292 | 0 .00057 | 0 .011692858 |
| CADM1 | -1 .073893665 | 9 .865170435 | -3 .7259 | 0 .00057 | 0 .01178582 |
| ATP5C1 | -0 .612390585 | 1 1 .8052804 | -3 .7216 | 0 .00058 | 0 .011906736 |
| APOL2 | 0 .689425665 | 7 .432244682 | 3 .72141 | 0 .00058 | 0 .011906736 |
| CDK2 | 0 .848790176 | 8 .791133579 | 3 .72044 | 0 .00058 | 0 .011921579 |
| COLGALT1 | 0 .329974282 | 8 .844016093 | 3 .71934 | 0 .00058 | 0 .011941283 |

| NDN | -0 .789982431 | 8 .307065197 | -3 .7135 | 0 .00059 | 0 .012114245 |
| --- | --- | --- | --- | --- | --- |
| GAS1 | -0 .663808867 | 8 .499596803 | -3 .7134 | 0 .00059 | 0 .012114245 |
| NSUN5 | 0 .426530797 | 9 .890906762 | 3 .70927 | 0 .0006 | 0 .012244555 |
| NME6 | -0 .487591542 | 6 .70904422 | -3 .7057 | 0 .00061 | 0 .012355559 |
| NPLOC4 | 0 .509487599 | 8 .566070582 | 3 .70489 | 0 .00061 | 0 .012364808 |
| CDC25A | 0 .623219635 | 5 .895766113 | 3 .70384 | 0 .00061 | 0 .012383343 |
| MEOX2 | -0 .990370363 | 5 . 149361426 | -3 .7029 | 0 .00061 | 0 .012396411 |
| AKR1C3 | -0 .946164447 | 5 .82855952 | -3 .7012 | 0 .00062 | 0 .012441651 |
| SYCP2 | 0 .765772469 | 3 .897815799 | 3 .69958 | 0 .00062 | 0 .012480407 |
| MYH11 | -2 .021942336 | 9 .073387783 | -3 .6987 | 0 .00062 | 0 .012491311 |
| TSPAN6 | -0 .629253605 | 8 . 124896815 | -3 .6967 | 0 .00062 | 0 .012535769 |
| SNAP29 | 0 .595684355 | 7 .594110914 | 3 .69647 | 0 .00062 | 0 .012535769 |
| BTG1 | -0 .60695828 | 1 1 .52582794 | -3 .6951 | 0 .00063 | 0 .012565113 |
| COMMD4 | 0 .329573847 | 9 .525135808 | 3 .69341 | 0 .00063 | 0 .012609877 |
| POMP | 0 .498175185 | 10 .02145472 | 3 .69042 | 0 .00064 | 0 .012674779 |
| SCYL2 | 0 .474874984 | 6 . 172600974 | 3 .69013 | 0 .00064 | 0 .012674779 |
| IGF2-AS | -0 .603088754 | 5 .6071743 | -3 .6901 | 0 .00064 | 0 .012674779 |
| TIMP1 | 0 .770842898 | 1 1 . 15024711 | 3 .6895 | 0 .00064 | 0 .012676046 |
| SEC14L2 | 0 .48030505 | 6 .203708749 | 3 .68514 | 0 .00065 | 0 .012820692 |
| NAPA | 0 .428182412 | 9 .486181944 | 3 .68201 | 0 .00065 | 0 .012919578 |
| VCPIP1 | 0 .722973475 | 5 . 197894107 | 3 .68129 | 0 .00065 | 0 .012926956 |
| ENO1 | 0 .491126472 | 1 1 .68468106 | 3 .6804 | 0 .00065 | 0 .012940324 |
| CXCL2 | 1 .457221968 | 4 .955447718 | 3 .67894 | 0 .00066 | 0 .012976047 |
| E2F3 | 0 .692239275 | 8 . 175757158 | 3 .67526 | 0 .00066 | 0 .013097723 |
| NEBL | -0 .444080691 | 9 .005138478 | -3 .6732 | 0 .00067 | 0 .013142588 |
| SRD5A1 | -0 .929278892 | 8 .725744023 | -3 .673 | 0 .00067 | 0 .013142588 |
| WASL | 0 .562555394 | 8 .077130847 | 3 .67101 | 0 .00067 | 0 .01320154 |
| IL12RB2 | 0 .474003496 | 6 .989948762 | 3 .66992 | 0 .00068 | 0 .013205254 |
| CEBPB | 0 .726084298 | 10 .53969451 | 3 .66972 | 0 .00068 | 0 .013205254 |
| BMP4 | -1 .640194515 | 4 .89378115 | -3 .6693 | 0 .00068 | 0 .013205254 |
| TTBK2 | -0 .716085036 | 5 .206008947 | -3 .6687 | 0 .00068 | 0 .013209627 |
| MYLK | -1 . 148639975 | 8 .220264493 | -3 .668 | 0 .00068 | 0 .013214729 |
| RPL12 | -0 .543080157 | 13 . 18513505 | -3 .6664 | 0 .00068 | 0 .013256655 |
| EPM2AIP1 | -0 .600519001 | 7 .649725083 | -3 .6634 | 0 .00069 | 0 .013347932 |
| PSMB2 | 0 .386966783 | 10 .05824134 | 3 .66308 | 0 .00069 | 0 .013347932 |
| SYNM | -1 . 161198184 | 7 .427752829 | -3 .6599 | 0 .0007 | 0 .013452188 |
| GALNT6 | 0 .888444439 | 7 .091420492 | 3 .65628 | 0 .0007 | 0 .013576931 |
| TMEM132A | 0 .451403983 | 6 .75801361 | 3 .6548 | 0 .00071 | 0 .013615779 |
| MAT2B | -0 .521123344 | 8 .551214016 | -3 .6513 | 0 .00071 | 0 .013734533 |
| FBLN1 | -0 .856309641 | 10 . 12182586 | -3 .6503 | 0 .00072 | 0 .013754066 |
| CTNNBIP1 | -0 .515020808 | 9 . 169551041 | -3 .6475 | 0 .00072 | 0 .013822294 |
| MXRA5 | -0 .75460324 | 8 .744143109 | -3 .6474 | 0 .00072 | 0 .013822294 |
| MMP9 | 1 .231862519 | 9 .01328912 | 3 .64711 | 0 .00072 | 0 .013822294 |
| RAB14 | -0 .462683432 | 9 .900827848 | -3 .6458 | 0 .00073 | 0 .013855869 |
| ASF1A | -0 .584082923 | 6 .862026371 | -3 .6441 | 0 .00073 | 0 .013901966 |
| THEMIS2 | 0 .705300803 | 7 .756148699 | 3 .64181 | 0 .00073 | 0 .013976146 |
| COL4A3BP | -0 .611942086 | 7 .792312546 | -3 .6391 | 0 .00074 | 0 .01406573 |
| KIAA0485 | -0 .486682208 | 6 .531482961 | -3 .6354 | 0 .00075 | 0 .014198588 |
| ALG3 | 0 .49456957 | 7 .809548455 | 3 .63205 | 0 .00076 | 0 .014305837 |
| CMPK1 | -0 .679743359 | 8 .486093461 | -3 .6319 | 0 .00076 | 0 .014305837 |
| TXNIP | -0 .616658156 | 12 .38893874 | -3 .6303 | 0 .00076 | 0 .01435101 |
| MRPS17 | 0 .523285424 | 7 .476793037 | 3 .62814 | 0 .00076 | 0 .014416895 |
| XYLT1 | -1 .052940763 | 6 .798672416 | -3 .6277 | 0 .00077 | 0 .014416895 |
| TSPYL1 | -0 .622687027 | 8 .498246378 | -3 .6233 | 0 .00078 | 0 .014557214 |
| CNN1 | -1 .388071047 | 6 .5725011 15 | -3 .6225 | 0 .00078 | 0 .014557214 |
| LTBP4 | -0 .647589423 | 8 .462742494 | -3 .6225 | 0 .00078 | 0 .014557214 |

| SLC25A13 | 0 .793794983 | 7 .472907464 | 3 .62237 | 0 .00078 | 0 .014557214 |
| --- | --- | --- | --- | --- | --- |
| ESD | -0 .494606209 | 10 .86032983 | -3 .6177 | 0 .00079 | 0 .014737284 |
| EIF3L | -0 .496635672 | 1 1 . 10704569 | -3 .6155 | 0 .00079 | 0 .014803898 |
| RHOBTB2 | 0 .544811738 | 6 .379012902 | 3 .61514 | 0 .00079 | 0 .014803898 |
| NADSYN1 | 0 .792035861 | 6 .077669625 | 3 .61459 | 0 .0008 | 0 .014805953 |
| DST | -0 .618298834 | 10 .32467154 | -3 .6133 | 0 .0008 | 0 .014839101 |
| EIF1AX | -0 .474921916 | 10 . 19599875 | -3 .6067 | 0 .00081 | 0 .015110009 |
| KTN1 | -0 .471637695 | 10 . 13734283 | -3 .6058 | 0 .00082 | 0 .015125736 |
| PITPNM1 | 0 .736961608 | 7 .018066665 | 3 .60367 | 0 .00082 | 0 .015198343 |
| H2AFZ | 0 .624133385 | 1 1 . 15618438 | 3 .59953 | 0 .00083 | 0 .015361932 |
| MYO1D | 0 .741742711 | 8 .76882919 | 3 .59901 | 0 .00083 | 0 .015362775 |
| TP53AIP1 | -0 .556489245 | 6 .390784729 | -3 .5979 | 0 .00084 | 0 .015388742 |
| ACSL1 | -1 .243894645 | 9 .253244008 | -3 .5965 | 0 .00084 | 0 .015429391 |
| SLC16A10 | -0 .609794715 | 6 .397775593 | -3 .5931 | 0 .00085 | 0 .015561153 |
| WNT4 | -0 .848396376 | 6 .415804134 | -3 .5914 | 0 .00085 | 0 .015617095 |
| PPARD | 0 .554427537 | 9 .068512198 | 3 .59008 | 0 .00085 | 0 .015655166 |
| PRSS3 | 0 .74939411 1 | 8 .429017592 | 3 .58279 | 0 .00087 | 0 .015970007 |
| AP1B1 | 0 .524487596 | 7 .64104584 | 3 .58159 | 0 .00088 | 0 .016003312 |
| SEC61A1 | 0 .390506739 | 8 .642648755 | 3 .58082 | 0 .00088 | 0 .016015765 |
| TRAF1 | 0 .581100141 | 6 .892759055 | 3 .57926 | 0 .00088 | 0 .016065772 |
| SERPINB13 | 0 .685594026 | 8 .474635386 | 3 .57729 | 0 .00089 | 0 .016116229 |
| GPHN | -0 .496246674 | 6 .292416987 | -3 .577 | 0 .00089 | 0 .016116229 |
| CSF3R | 0 .709715452 | 5 .871231475 | 3 .57671 | 0 .00089 | 0 .016116229 |
| SLC2A3 | 0 .89145719 | 8 .078737065 | 3 .5758 | 0 .00089 | 0 .016136112 |
| SLAMF7 | 1 .424149218 | 5 .777384281 | 3 .57527 | 0 .00089 | 0 .016137833 |
| TIA1 | -0 .463929153 | 9 .003445369 | -3 .5739 | 0 .0009 | 0 .016170295 |
| ARMCX1 | -0 .753873186 | 7 .444327665 | -3 .5734 | 0 .0009 | 0 .016170295 |
| SYT17 | -1 .03248318 | 5 .522724647 | -3 .5731 | 0 .0009 | 0 .016170295 |
| POPDC3 | 1 .21020966 | 4 .962122822 | 3 .57178 | 0 .0009 | 0 .016210087 |
| ZCCHC2 | -0 .46941394 | 6 .257242792 | -3 .5711 | 0 .0009 | 0 .016219232 |
| CD14 | 1 .061568661 | 8 .044863782 | 3 .5684 | 0 .00091 | 0 .016324231 |
| CH25H | 0 .903936082 | 6 .593769631 | 3 .5675 | 0 .00091 | 0 .016344066 |
| LDB2 | -0 .785965218 | 6 . 193451823 | -3 .5657 | 0 .00092 | 0 .016406595 |
| ADAMDEC1 | 1 .597832861 | 6 .861780131 | 3 .56422 | 0 .00092 | 0 .016454494 |
| FAM13A | -0 .389501093 | 8 .817064781 | -3 .5631 | 0 .00092 | 0 .016485684 |
| FZD1 | -0 .594016406 | 7 .576296002 | -3 .5601 | 0 .00093 | 0 .016586564 |
| FNBP1L | -0 .740533246 | 5 .882357848 | -3 .5597 | 0 .00093 | 0 .016586564 |
| TRIM38 | 0 .396857103 | 9 . 132207899 | 3 .55954 | 0 .00093 | 0 .016586564 |
| ZNF175 | -0 .64290902 | 5 .826808923 | -3 .559 | 0 .00094 | 0 .016589213 |
| CUL5 | -0 .546536763 | 8 .274942067 | -3 .5565 | 0 .00094 | 0 .016686957 |
| TMEM8B | -0 .529911 192 | 6 .820414372 | -3 .5557 | 0 .00095 | 0 .016703443 |
| ELN | -0 .818295034 | 8 .032499283 | -3 .5535 | 0 .00095 | 0 .016786941 |
| MTMR11 | -0 .448891765 | 6 .345508298 | -3 .553 | 0 .00095 | 0 .016788872 |
| CABP5 | 2 .079677902 | 4 .641940518 | 3 .54999 | 0 .00096 | 0 .016912267 |
| FOSL1 | 0 .761166762 | 7 . 159445728 | 3 .54687 | 0 .00097 | 0 .017043129 |
| TRPS1 | -0 .585886175 | 6 .549192649 | -3 .5457 | 0 .00097 | 0 .017066207 |
| GINS1 | 0 .523144376 | 6 .492761362 | 3 .54545 | 0 .00097 | 0 .017066207 |
| ZMYND11 | -0 .646980984 | 9 .378454674 | -3 .5448 | 0 .00098 | 0 .017076952 |
| NPIPA1 | -0 .705317789 | 9 .687658695 | -3 .5442 | 0 .00098 | 0 .01707862 |
| SHCBP1 | 0 .644106721 | 5 .81762604 | 3 .53927 | 0 .00099 | 0 .017271006 |
| MAP7D1 | 0 .548836655 | 8 .368639148 | 3 .5387 | 0 .00099 | 0 .017271006 |
| PSIP1 | -0 .502565445 | 8 .442187797 | -3 .5384 | 0 .00099 | 0 .017271006 |
| FOXA1 | -0 .491630674 | 5 .34962746 | -3 .5382 | 0 .00099 | 0 .017271006 |
| C1QA | 1 . 165720371 | 9 .620975871 | 3 .53802 | 0 .001 | 0 .017271006 |
| GLUD1 | -0 .515807893 | 9 . 173100228 | -3 .5372 | 0 .001 | 0 .017287782 |
| PBK | 0 .968043543 | 5 .341585836 | 3 .53657 | 0 .001 | 0 .017295985 |

| RRAS  C21orf33  NDNF RP4-581F12 . 1  CTDP1  RRM2  ZFPM2  UBAC1  HLA-A  SESN1  RFX5  SLURP1  ANKRD36  DHX8  RTP4  TPD52L1  TLE4  EMCN  PNRC1  SLC47A1  GUCY1A3  DNM1  CLEC2B  APOE  RPS27  RPL10A  LPXN  SH3YL1  N4BP2L2  MIF  FRMD4B  RAB3GAP1  MYD88  POLR2E  ALDH9A1  NCALD  CRCT1  TBC1D31  BLOC1S1  SEC61G  MCM4  KIF11  AHCYL2  TOX3  CTDSPL  PNKP  NR1H3  KLK7  TAP1  MICALL1  KDM4C | -0 .530762165 -0 .454990251 -0 .586425953 0 .675399673 0 .382974031 0 .700764278 -0 .807101527 -0 .448755561 0 .446112873 -0 .469993494 0 .454497674 1 .8640553 -1 .081405352 0 .426407392 0 .706458297 -0 .733144351 -0 .590001707 -0 .673008081 -0 .789815385 -0 .500516215 -0 .521624954 -0 .757315508 -0 .632541562 0 .784321477 -0 .557438645 -0 .525501819 0 .809576652 -0 .782337146 -0 .569922551 0 .530459729 -0 .619560686 -0 .320857657 0 .474331877 0 .436321128 -0 .492620075 -0 .834461287 2 . 145842012 0 .532950926 -0 .428660409 0 .46405711 0 .56259634 0 .714704567 -0 .880570726 -0 .857014812 -0 .543905656 0 .517113578 -0 .500439639 2 .029239418 0 .93445765 0 .545997667 -0 .431744913 |
| --- | --- |

DKFZP586I1420 -0 .656089841

0 .808704953 -0 .520482404 0 .414033352 -1 .026809992

NPL SETD5 SOS1 GATA3

7 .746163158 8 .866214345 5 .859956717 6 .30521696 6 .791028148 7 .971988608 4 .577068123 7 .61573018 13 .42635564 7 .329997077 8 .25851679 7 .320658778 4 .813098322 6 .282429083 6 .870768195 8 .822811748 6 .655923228 5 .816479316 9 .416157518 6 .082649144 7 .510886058 7 .528449584 7 .648809804 12 .88696732 1 1 .41134937 1 1 .06945563 7 .926570428 7 .352800346 8 .885025377 10 .71401179 7 .47851058 9 .542165307 9 .097649904 10 . 1 1962502 9 .69433765 6 .840580724 7 .652524546 5 .27090052 8 .308215638 9 .827332441 8 .648077102 5 .623680102 8 .813039849 4 .844590289 9 .608917784 7 .009940635 7 .81736548 7 .719133777 9 .077398572 9 . 135798432 6 .953260493 7 .267736607 6 . 189220056 8 .437351524 8 .019005388 9 .552719352

-3 .5329 -3 .5328 -3 .5281 3 .52736 3 .527 3 .52392 -3 .5238 -3 .5237 3 .52142 -3 .5189 3 .51674 3 .51342 -3 .5133 3 .5131 3 .5127 -3 .5125 -3 .5117 -3 .5099 -3 .5092 -3 .5076 -3 .5064 -3 .5061 -3 .5053 3 .50507 -3 .504 -3 .503 3 .50285 -3 .5025 -3 .502 3 .50125 -3 .5009 -3 .5005 3 .49707 3 .4967 -3 .4958 -3 .4943 3 .49391 3 .49363 -3 .4918 3 .48832 3 .48786 3 .48785 -3 .4865 -3 .4855 -3 .4849 3 .48248 -3 .4818 3 .47971 3 .47705 3 .47511 -3 .475 -3 .475 3 .47476 -3 .472 3 .4716 -3 .4657

0 .00101 0 .00101 0 .00102 0 .00103 0 .00103 0 .00104 0 .00104 0 .00104 0 .00104 0 .00105 0 .00106 0 .00107 0 .00107 0 .00107 0 .00107 0 .00107 0 .00107 0 .00108 0 .00108 0 .00109 0 .00109 0 .00109 0 .00109 0 .0011 0 .0011 0 .0011 0 .0011 0 .0011 0 .0011 1 0 .0011 1 0 .0011 1 0 .0011 1 0 .00112 0 .00112 0 .00113 0 .00113 0 .00113 0 .00113 0 .00114 0 .00115 0 .00115 0 .00115 0 .00116 0 .00116 0 .00116 0 .00117 0 .00117 0 .00118 0 .00119 0 .00119 0 .00119 0 .0012 0 .0012 0 .00121 0 .00121 0 .00123

0 .017441205

0 .017441205

0 .017653056

0 .017663365

0 .017663365

0 .01776053

0 .01776053

0 .01776053

0 .0178544

0 .017960903

0 .018050142

0 .018149637

0 .018149637

0 .018149637

0 .018149637

0 .018149637

0 .018170535

0 .018241038

0 .01825017

0 .018309286

0 .018344124

0 .018344124

0 .018348022

0 .018348022

0 .018379482

0 .018388211

0 .018388211

0 .018388211

0 .018391509

0 .018394748

0 .018394748

0 .018394748

0 .018551548

0 .018551548

0 .018574816

0 .018619152

0 .018619152

0 .018619152

0 .018694752

0 .018835255

0 .018835255

0 .018835255

0 .018884864

0 .018915511

0 .018922033

0 .019030882

0 .019045191

0 .019134313

0 .019257226

0 .019285679

0 .019285679

0 .019285679

0 .019285679

0 .019412992

0 .019412992

0 .019721326

| OLFML2A | -0 .539299865 | 8 .297455791 | -3 .4616 | 0 .00124 | 0 .019922554 |
| --- | --- | --- | --- | --- | --- |
| ERBB2 | -0 .578077912 | 8 .61585827 | -3 .4613 | 0 .00124 | 0 .019922554 |
| RBM7 | -0 .474676315 | 6 .801519462 | -3 .4602 | 0 .00125 | 0 .019958256 |
| TLE1 | -0 .514621673 | 9 .33278147 | -3 .4575 | 0 .00126 | 0 .020088658 |
| 6-Mar | -0 .48376944 | 9 .816408706 | -3 .4567 | 0 .00126 | 0 .020112292 |
| KRT6B | 1 .315022158 | 12 .56549795 | 3 .456 | 0 .00126 | 0 .020113074 |
| IDH3A | 0 .360709786 | 8 .076987991 | 3 .4558 | 0 .00126 | 0 .020113074 |
| FXYD1 | -0 .942231781 | 7 .469468326 | -3 .4549 | 0 .00127 | 0 .020139528 |
| LCE2B | 1 .981417568 | 7 .747244193 | 3 .45391 | 0 .00127 | 0 .020171348 |
| PROSC | -0 .39351492 | 9 .351330825 | -3 .4533 | 0 .00127 | 0 .020183859 |
| COX7A1 | -1 . 164457704 | 6 .991195769 | -3 .4503 | 0 .00128 | 0 .020331872 |
| DSC2 | 1 .003843724 | 8 .038320237 | 3 .44872 | 0 .00129 | 0 .020367807 |
| HBB | -0 .625388888 | 8 .830948487 | -3 .4483 | 0 .00129 | 0 .020367807 |
| ARL1 | -0 .346234434 | 9 .078780064 | -3 .4481 | 0 .00129 | 0 .020367807 |
| LRRK1 | 0 .451748123 | 5 .683666407 | 3 .44706 | 0 .0013 | 0 .020367807 |
| GNL2 | 0 .699748466 | 6 .710187585 | 3 .44692 | 0 .0013 | 0 .020367807 |
| YTHDC1 | -0 .414063649 | 8 .800256683 | -3 .4469 | 0 .0013 | 0 .020367807 |
| PHB2 | -0 .419810554 | 10 .8366567 | -3 .4466 | 0 .0013 | 0 .020367807 |
| B4GALT7 | 0 .33370184 | 8 .000207264 | 3 .44603 | 0 .0013 | 0 .020376968 |
| SPOCK2 | 0 .836011453 | 8 .938245657 | 3 .44388 | 0 .00131 | 0 .020477906 |
| UGDH | -0 .528244859 | 7 . 135939385 | -3 .437 | 0 .00133 | 0 .02085882 |
| FGFBP1 | 0 .685661353 | 8 . 147125934 | 3 .43576 | 0 .00134 | 0 .02091032 |
| RAB40A | -0 .903956821 | 5 .590607896 | -3 .4329 | 0 .00135 | 0 .021055906 |
| KIF23 | 1 .039847424 | 4 .791305116 | 3 .43248 | 0 .00135 | 0 .021055906 |
| NEK9 | -0 .39156691 | 8 . 144989894 | -3 .4308 | 0 .00136 | 0 .021091058 |
| CFD | -1 .68890712 | 8 .999465593 | -3 .4307 | 0 .00136 | 0 .021091058 |
| TLE3 | 0 .317263563 | 8 .081032107 | 3 .43061 | 0 .00136 | 0 .021091058 |
| RUFY3 | -0 .48035932 | 8 .878206864 | -3 .4292 | 0 .00136 | 0 .021148759 |
| SLC3A2 | 0 .51938824 | 9 . 148906636 | 3 .42783 | 0 .00137 | 0 .021198221 |
| MGEA5 | -0 .428478002 | 8 .599946616 | -3 .4276 | 0 .00137 | 0 .021198221 |
| TMEM135 | -0 .700285178 | 6 .04076772 | -3 .4271 | 0 .00137 | 0 .0211991 |
| WBSCR22 | 0 .43208441 | 8 .447660449 | 3 .42633 | 0 .00137 | 0 .021220957 |
| C2orf68 | -0 .597605255 | 6 .592701054 | -3 .4215 | 0 .00139 | 0 .021488308 |
| TNFRSF1B | 1 .068751195 | 7 .602957993 | 3 .41839 | 0 .00141 | 0 .021657036 |
| SUV420H1 | -0 .670171247 | 7 .796065301 | -3 .4176 | 0 .00141 | 0 .021667467 |
| NAA60 | 0 .31152753 | 9 .527950115 | 3 .41737 | 0 .00141 | 0 .021667467 |
| SCML1 | -0 .797813916 | 5 .26985465 | -3 .4138 | 0 .00143 | 0 .02186143 |
| ATP2A2 | 0 .443337469 | 10 .63615807 | 3 .41069 | 0 .00144 | 0 .022020621 |
| PLEKHM2 | 0 .49893078 | 7 .796389057 | 3 .41046 | 0 .00144 | 0 .022020621 |
| CPNE3 | -0 .548427672 | 9 .415869905 | -3 .4093 | 0 .00144 | 0 .02204777 |
| RGS20 | 1 .297234523 | 6 .762675194 | 3 .40898 | 0 .00145 | 0 .02204777 |
| PSEN1 | -0 .338768689 | 8 .602958022 | -3 .4086 | 0 .00145 | 0 .02204777 |
| SNRK | -0 .465431633 | 8 . 187427594 | -3 .4083 | 0 .00145 | 0 .02204777 |
| SPINK1 | 0 .590971906 | 5 .986203368 | 3 .40691 | 0 .00145 | 0 .022065484 |
| HOXB5 | -0 .361652591 | 8 . 124313079 | -3 .4067 | 0 .00145 | 0 .022065484 |
| C6orf120 | -0 .373514556 | 8 .075202406 | -3 .4066 | 0 .00145 | 0 .022065484 |
| SNX5 | -0 .375961607 | 7 .551804681 | -3 .4063 | 0 .00146 | 0 .022065484 |
| JMJD1C | -0 .531825177 | 6 .556162368 | -3 .4059 | 0 .00146 | 0 .022065484 |
| ATP5O | -0 .575252497 | 9 .57510916 | -3 .4055 | 0 .00146 | 0 .022065484 |
| JUN | 0 .67451273 | 9 .738679596 | 3 .4049 | 0 .00146 | 0 .022077933 |
| NUP62 | 0 .365548152 | 8 .958850571 | 3 .40448 | 0 .00146 | 0 .022077968 |
| NAP1L1 | -0 .585771816 | 12 . 15494754 | -3 .4005 | 0 .00148 | 0 .022302795 |
| APOBEC3G | 1 .083202133 | 6 .910790452 | 3 .39814 | 0 .00149 | 0 .022429077 |
| HMGN3 | -0 .565960602 | 9 .063768053 | -3 .3973 | 0 .00149 | 0 .022455359 |
| SPOP | -0 .324118005 | 8 .997934268 | -3 .3959 | 0 .0015 | 0 .022520078 |
| DEF8 | 0 .543551993 | 6 .520013039 | 3 .393 | 0 .00151 | 0 .022679937 |

| SEMA3C | -0 .681575704 | 8 .961869641 | -3 .3886 | 0 .00153 | 0 .022918555 |
| --- | --- | --- | --- | --- | --- |
| OSR2 | -0 .913612546 | 7 .277418088 | -3 .3883 | 0 .00153 | 0 .022918555 |
| NCAPG | 0 .790924756 | 6 .035364444 | 3 .38809 | 0 .00153 | 0 .022918555 |
| HLA-F | 0 .571764847 | 12 .72543658 | 3 .38628 | 0 .00154 | 0 .02300963 |
| ADAMTS13 | 1 .09153083 | 5 . 168335035 | 3 .38495 | 0 .00155 | 0 .023069933 |
| PPP1R11 | 0 .391303713 | 9 .898512076 | 3 .38304 | 0 .00156 | 0 .023168868 |
| CDC6 | 0 .44638745 | 6 .273123318 | 3 .382 | 0 .00156 | 0 .023210192 |
| SOX13 | 0 .819438396 | 8 .889633265 | 3 .37981 | 0 .00157 | 0 .023328529 |
| TMEM214 | 0 .360696442 | 8 .989571393 | 3 .37913 | 0 .00157 | 0 .023346382 |
| CCNJL | 0 .457558283 | 5 .837342279 | 3 .37782 | 0 .00158 | 0 .023385234 |
| KIF20A | 1 .299072562 | 6 . 138208354 | 3 .37767 | 0 .00158 | 0 .023385234 |
| COMMD9 | 0 .43758202 | 8 .018749585 | 3 .3773 | 0 .00158 | 0 .023385234 |
| MDK | 0 .769244559 | 6 .348493576 | 3 .37549 | 0 .00159 | 0 .023479189 |
| 2-Mar | -0 .495097528 | 6 .946476017 | -3 .3731 | 0 .0016 | 0 .023609594 |
| LCP2 | 0 .961077023 | 8 .217842346 | 3 .37272 | 0 .0016 | 0 .023609594 |
| NDC80 | 1 .465678783 | 4 . 191095193 | 3 .37134 | 0 .00161 | 0 .02367411 |
| TCN1 | 1 .081989265 | 6 .905815558 | 3 .37095 | 0 .00161 | 0 .02367411 |
| LYVE1 | -0 .775780327 | 5 .474451645 | -3 .3681 | 0 .00162 | 0 .023837443 |
| KIAA0100 | 0 .454901902 | 9 .587680599 | 3 .36681 | 0 .00163 | 0 .023899218 |
| PIK3CD | 0 .512443388 | 8 .540608752 | 3 .36628 | 0 .00163 | 0 .02390709 |
| SNRPC | 0 .378492825 | 8 . 1 12559778 | 3 .36209 | 0 .00165 | 0 .024166076 |
| USP47 | -0 .481972358 | 7 .970068125 | -3 .3613 | 0 .00166 | 0 .02419042 |
| IL36RN | 1 . 14359582 | 7 .044965301 | 3 .35908 | 0 .00167 | 0 .02431774 |
| GAPDH | 0 .429666639 | 14 .24140075 | 3 .35806 | 0 .00167 | 0 .024345987 |
| DNAJB14 | -0 .623388541 | 6 .680581197 | -3 .3579 | 0 .00167 | 0 .024345987 |
| ACSF2 | -0 .694025664 | 5 .926587906 | -3 .3558 | 0 .00168 | 0 .024460369 |
| TP53I3 | 0 .3428953 | 6 .883582687 | 3 .35481 | 0 .00169 | 0 .024501247 |
| ORMDL2 | 0 .436934985 | 8 .366528911 | 3 .35402 | 0 .00169 | 0 .024528135 |
| GPI | 0 .503297294 | 9 .802113592 | 3 .35265 | 0 .0017 | 0 .024595817 |
| SERPINB7 | 1 .69599354 | 5 .88243762 | 3 .34932 | 0 .00171 | 0 .024798475 |
| PRR7 | 0 .765700143 | 6 .069010664 | 3 .34895 | 0 .00172 | 0 .024798475 |
| TBC1D12 | -0 .419847132 | 6 . 137093434 | -3 .3482 | 0 .00172 | 0 .024825735 |
| NRIP3 | 0 .470470157 | 7 .035540228 | 3 .34647 | 0 .00173 | 0 .024916834 |
| GAR1 | 0 .41851626 | 7 .899645784 | 3 .34546 | 0 .00173 | 0 .024959531 |
| PMS1 | -0 .436485947 | 6 .889202274 | -3 .3393 | 0 .00176 | 0 .025374032 |
| RPS27L | -0 .830598853 | 8 .58741683 | -3 .3383 | 0 .00177 | 0 .025414297 |
| CCR1 | 0 .582248772 | 7 .616013535 | 3 .33498 | 0 .00178 | 0 .02562033 |
| HSPB7 | -0 .613834169 | 6 .854847959 | -3 .3338 | 0 .00179 | 0 .02562033 |
| SLC7A8 | 0 .414098457 | 10 .04233988 | 3 .33352 | 0 .00179 | 0 .02562033 |
| FTSJ2 | 0 .419213019 | 8 .405466314 | 3 .33338 | 0 .00179 | 0 .02562033 |
| SSR4 | 0 .603989481 | 10 . 13603717 | 3 .33327 | 0 .00179 | 0 .02562033 |
| CHP1 | -0 .480582508 | 10 .42412948 | -3 .3328 | 0 .0018 | 0 .02562033 |
| CLDND1 | -0 .765323571 | 7 .209663781 | -3 .3326 | 0 .0018 | 0 .02562033 |
| TMEM8A | 0 .597268897 | 7 .916797085 | 3 .33125 | 0 .0018 | 0 .025692456 |
| MMP13 | 0 .675617147 | 5 .003994163 | 3 .32821 | 0 .00182 | 0 .02587531 |
| TCF7L1 | -0 .892379853 | 7 .695246399 | -3 .3275 | 0 .00182 | 0 .02587531 |
| ATRX | -0 .433220423 | 8 .70319453 | -3 .3273 | 0 .00182 | 0 .02587531 |
| ARHGAP11A | 0 .710257306 | 3 .968410331 | 3 .32693 | 0 .00183 | 0 .02587531 |
| PID1 | -1 . 135823181 | 6 .380197094 | -3 .3268 | 0 .00183 | 0 .02587531 |
| LMAN2 | 0 .424391501 | 7 .720225889 | 3 .32598 | 0 .00183 | 0 .025903246 |
| SSTR1 | 0 .580326077 | 5 .885313698 | 3 .3251 | 0 .00184 | 0 .025938908 |
| IFRD2 | 0 .346364348 | 8 . 1 13773706 | 3 .32231 | 0 .00185 | 0 .026115672 |
| PLAUR | 0 .562206797 | 8 .782713594 | 3 .32093 | 0 .00186 | 0 .026173489 |
| C5AR1 | 1 .408550523 | 5 .51584471 | 3 .32073 | 0 .00186 | 0 .026173489 |
| CTSL | 0 .634519033 | 8 .983218307 | 3 .3202 | 0 .00186 | 0 .026183889 |
| MCMBP | -0 .422400671 | 6 .630747834 | -3 .3182 | 0 .00187 | 0 .026302603 |

-0 .453866845 -0 .578036725 -0 .463327651 -0 .50822027 0 .524387413 -0 .676258283 0 .436286279 -0 .465436066 -0 .598275405 0 .472632394 0 .382963585 0 .302448363 0 .400383927 0 .684374117 -0 .460819359 0 .50232617 1 .031343396 0 .595110704 0 .413296263 0 .306169553 -0 .505752186 0 .964861423 -0 .578310431 -0 .379795632 -0 .430961692 0 .312967743 0 .728199515 -0 .47176096 -0 .871553333 0 .459731279 0 .459479865 -0 .713124109 0 .680469392 -1 .08126436 -0 .439026351 0 .530779199 -1 .290832979 -0 .442863425 -0 .464922232 0 .285404609 -0 .348985273 0 .639172084 1 . 137040979 -0 .440203431 0 .394510966 0 .427629216 0 .467114399 -0 .582194677 -0 .446088264 0 .829072761 -0 .879420383 0 .604595282 0 .748400602 0 .90858431 0 .665258145

NSL1 FLJ42627 THSD7A PTPRK CDK5 NRCAM NOP2 PLXNB1 NMRK1 BCKDK PSAP TNIP2 H2AFY HMHB1 SSBP3 SKIV2L CYTL1 CD55 CDC37 MED8 ZNHIT3 TGM1 MEGF6 CHCHD2 BTAF1 AIMP2 LDHAL6B PTEN LGR4 COPG1

CARHSP1

C6orf48

FCGR1B RORA TBC1D5 ZDHHC24 ABCA8 RPS6 FAM114A1 HMOX2 RBPJ RASSF2 HSPB8 MAP3K4 ATP6V0A1 MBOAT7 HLX

BPTF

FIS1

CALCB

AACS

CALU

FKBP10

FCGR2A

ACP5

CYP3A7-CYP3AP-0 .556941148

7 .860614345 7 .445416507 6 .552588823 7 .714613881 7 .32648531 7 .697604492 8 . 153902924 7 .348457678 6 .83011903 7 .851123555 12 .0117011 1 8 .98297253 10 .81665505 5 .864611057 8 . 1 1 1079656 7 .604369318 6 .719612253 8 .018508506 7 .803362515 8 .654205406 7 . 151759178 6 . 189070907 7 .258062806 10 .50736392 6 .41518297 10 .2386251 4 .634573098 8 .456220082 6 .532177086 8 .52660467 8 .261149126 9 .84857466 6 . 176678059 8 .423586625 9 .560965215 7 .781025784 6 .880123894 13 .73579231 8 .478318285 9 .550380161 9 .907292961 7 .415716161 8 .066067743 7 .948942249 9 .391932258 7 .833595917 6 .423545204 8 .813631712 8 .812977198 3 .934472098 8 .266180629 10 .56112047 7 .303766551 6 .914180397 9 .063513317 4 .640297583

-3 .3168 -3 .3167 -3 .3142 -3 .3127 3 .31202 -3 .3108 3 .30816 -3 .3077 -3 .3073 3 .30673 3 .30545 3 .3053 3 .30515 3 .30471 -3 .3021 3 .2997 3 .29945 3 .29922 3 .29426 3 .29421 -3 .2936 3 .29358 -3 .2924 -3 .2911 -3 .2906 3 .29054 3 .28999 -3 .2899 -3 .2873 3 .28667 3 .28581 -3 .2854 3 .28499 -3 .2842 -3 .2824 3 .28079 -3 .2793 -3 .2773 -3 .2765 3 .27626 -3 .2761 3 .27504 3 .27498 -3 .2746 3 .27403 3 .2738 3 .27043 -3 .2702 -3 .2678 3 .26745 -3 .2669 3 .26617 3 .26378 3 .26259 3 .26258 -3 .2622

0 .00188 0 .00188 0 .00189 0 .0019 0 .0019 0 .00191 0 .00193 0 .00193 0 .00193 0 .00193 0 .00194 0 .00194 0 .00194 0 .00194 0 .00196 0 .00197 0 .00197 0 .00198 0 .002 0 .002 0 .00201 0 .00201 0 .00201 0 .00202 0 .00202 0 .00202 0 .00203 0 .00203 0 .00204 0 .00205 0 .00205 0 .00205 0 .00206 0 .00206 0 .00207 0 .00208 0 .00209 0 .0021 0 .00211 0 .00211 0 .00211 0 .00211 0 .00211 0 .00212 0 .00212 0 .00212 0 .00214 0 .00214 0 .00216 0 .00216 0 .00216 0 .00217 0 .00218 0 .00219 0 .00219 0 .00219

0 .026357956

0 .026357956

0 .026516428

0 .026594618

0 .026618129

0 .026679364

0 .026850555

0 .026856939

0 .026858651

0 .026870026

0 .026900615

0 .026900615

0 .026900615

0 .026904778

0 .027073839

0 .027205651

0 .027205651

0 .027205651

0 .02752159

0 .02752159

0 .02752159

0 .02752159

0 .027581247

0 .027631272

0 .027631272

0 .027631272

0 .027631272

0 .027631272

0 .027797088

0 .027819682

0 .027857271

0 .027861321

0 .027861321

0 .027896682

0 .028003517

0 .02810216

0 .028190322

0 .028316749

0 .02832648

0 .02832648

0 .02832648

0 .028349874

0 .028349874

0 .028349874

0 .028355484

0 .028355484

0 .028582858

0 .028582858

0 .028744629

0 .028744629

0 .028760236

0 .028787391

0 .028950322

0 .028983951

0 .028983951

0 .028983951

| IFITM2 | 0 .429011709 | 12 . 15934077 | 3 .26061 | 0 .0022 | 0 .029075039 |
| --- | --- | --- | --- | --- | --- |
| POLDIP3 | -0 .35421013 | 8 .950510804 | -3 .2601 | 0 .00221 | 0 .029075039 |
| ZNF573 | -0 .498543286 | 6 .627789462 | -3 .2597 | 0 .00221 | 0 .029075039 |
| TCEAL1 | -0 .604600401 | 7 . 179124529 | -3 .2596 | 0 .00221 | 0 .029075039 |
| RAPGEF6 | -0 .430464312 | 6 .359213658 | -3 .2593 | 0 .00221 | 0 .029075039 |
| SUCLG1 | -0 .459743008 | 9 .288011715 | -3 .2585 | 0 .00221 | 0 .029102373 |
| CHRM4 | -0 .575660237 | 6 .267425735 | -3 .2568 | 0 .00223 | 0 .029129714 |
| HIST1H4A | 0 .503993573 | 4 .901586001 | 3 .25679 | 0 .00223 | 0 .029129714 |
| STAG1 | -0 .471444127 | 8 .098370362 | -3 .2566 | 0 .00223 | 0 .029129714 |
| RABGGTA | 0 .460521676 | 6 .832705228 | 3 .25659 | 0 .00223 | 0 .029129714 |
| UNC119B | -0 .450917761 | 7 .774492071 | -3 .2563 | 0 .00223 | 0 .029129714 |
| TPM2 | -0 .913488779 | 7 .295243274 | -3 .256 | 0 .00223 | 0 .029129714 |
| RPS6KA5 | -0 .567523791 | 8 .055929308 | -3 .2542 | 0 .00224 | 0 .029243487 |
| ARF3 | 0 .341682178 | 10 .44767725 | 3 .25064 | 0 .00226 | 0 .029508989 |
| CMAHP | -0 .539090877 | 6 . 137492414 | -3 .2455 | 0 .0023 | 0 .029899199 |
| ORAI2 | 0 .351968948 | 7 . 106109489 | 3 .24521 | 0 .0023 | 0 .029899199 |
| SLC4A2 | 0 .456461348 | 8 .721359345 | 3 .24428 | 0 .00231 | 0 .02991405 |
| HOMER3 | 0 .38591309 | 8 .388573323 | 3 .2433 | 0 .00231 | 0 .02991405 |
| FZD2 | 0 .46871501 | 5 .64079331 | 3 .24307 | 0 .00231 | 0 .02991405 |
| PEA15 | 0 .448648058 | 10 .72371146 | 3 .24294 | 0 .00231 | 0 .02991405 |
| CAND2 | 0 .531447196 | 7 .008438231 | 3 .24285 | 0 .00231 | 0 .02991405 |
| GNG5 | 0 .347769719 | 10 .63272119 | 3 .24282 | 0 .00231 | 0 .02991405 |
| ALOX12B | 1 .276002022 | 6 .722720837 | 3 .24141 | 0 .00232 | 0 .029988798 |
| MAPKAPK2 | 0 .379956404 | 9 .810702591 | 3 .2412 | 0 .00233 | 0 .029988798 |
| SOX9 | -1 .0927885 | 9 .50479778 | -3 .2406 | 0 .00233 | 0 .029996419 |
| TRAK1 | -0 .421451012 | 9 .279426926 | -3 .2404 | 0 .00233 | 0 .029996419 |
| CEP57 | -0 .456921614 | 8 .485625242 | -3 .239 | 0 .00234 | 0 .030060679 |
| CEMIP | 0 .71407154 | 5 .379029113 | 3 .23859 | 0 .00234 | 0 .030060679 |
| FANCG | -0 .449714606 | 7 .528714458 | -3 .2385 | 0 .00234 | 0 .030060679 |
| MID2 | -0 .68101292 | 8 .545848678 | -3 .2367 | 0 .00236 | 0 .030158417 |
| RYBP | -0 .379253502 | 9 .681598203 | -3 .2366 | 0 .00236 | 0 .030158417 |
| MAGED1 | 0 .456985575 | 9 .536543362 | 3 .23584 | 0 .00236 | 0 .030193806 |
| DDB2 | -0 .461184636 | 7 .620113827 | -3 .2347 | 0 .00237 | 0 .030252065 |
| RHOT1 | -0 .5254245 | 8 .816909327 | -3 .2344 | 0 .00237 | 0 .030252065 |
| RPS6KA4 | 0 .440373579 | 8 .764874116 | 3 .23326 | 0 .00238 | 0 .030309534 |
| IGF2BP3 | 0 .647626344 | 6 .433781224 | 3 .23276 | 0 .00238 | 0 .030309534 |
| VEZF1 | -0 .599264615 | 9 . 1 15044175 | -3 .2325 | 0 .00238 | 0 .030309534 |
| DCAF10 | -0 .3627642 | 6 .498600622 | -3 .2323 | 0 .00238 | 0 .030309534 |
| EPHB4 | -0 .314505438 | 8 .575901354 | -3 .231 | 0 .00239 | 0 .030390188 |
| CDK19 | -0 .632416628 | 8 .441269229 | -3 .2304 | 0 .0024 | 0 .03041348 |
| CITED1 | 1 .403616002 | 5 .509882116 | 3 .22984 | 0 .0024 | 0 .030426227 |
| RRP1 | 0 .400858926 | 7 .788686182 | 3 .22737 | 0 .00242 | 0 .030606296 |
| NUDT1 | 0 .648316004 | 5 .828929416 | 3 .22667 | 0 .00242 | 0 .030635259 |
| LOC728392 | -0 .598923118 | 6 .709884029 | -3 .2262 | 0 .00243 | 0 .030646288 |
| ACSBG1 | -1 .430355579 | 7 .571399096 | -3 .2237 | 0 .00244 | 0 .030831091 |
| MSL2 | -0 .369910696 | 7 .229631266 | -3 .2229 | 0 .00245 | 0 .030866391 |
| GSN | -0 .435277807 | 10 .88003617 | -3 .2221 | 0 .00245 | 0 .030901952 |
| RPL24 | -0 .428341169 | 12 .29728323 | -3 .221 | 0 .00246 | 0 .030955457 |
| HDLBP | 0 .361658406 | 10 .72812591 | 3 .22025 | 0 .00247 | 0 .030955457 |
| KCNK10 | 1 .572888668 | 4 .967659468 | 3 .22003 | 0 .00247 | 0 .030955457 |
| PXN | 0 .361656123 | 8 .73637298 | 3 .21979 | 0 .00247 | 0 .030955457 |
| FPR1 | 0 .519757315 | 6 .85536074 | 3 .21972 | 0 .00247 | 0 .030955457 |
| HIST1H2BK | 0 .760739766 | 9 .233433183 | 3 .21815 | 0 .00248 | 0 .031035346 |
| GYS1 | 0 .328663896 | 8 .348016453 | 3 .21808 | 0 .00248 | 0 .031035346 |
| SERPINE1 | 0 .659393675 | 7 .247685142 | 3 .21731 | 0 .00249 | 0 .031070301 |
| ARHGAP5 | -0 .573159686 | 6 .738287131 | -3 .217 | 0 .00249 | 0 .031070301 |

| KAT6A | -0 .327037923 | 9 .049572217 | -3 .2132 | 0 .00252 | 0 .031370059 |
| --- | --- | --- | --- | --- | --- |
| MORC4 | -0 .313196513 | 5 .840918557 | -3 .212 | 0 .00252 | 0 .031442296 |
| TRIM62 | 0 .503339196 | 7 .838926833 | 3 .2113 | 0 .00253 | 0 .031471498 |
| PTHLH | 0 .616405953 | 5 .529238529 | 3 .2109 | 0 .00253 | 0 .031475171 |
| TAF10 | 0 .439399639 | 9 .806216399 | 3 .21008 | 0 .00254 | 0 .031515678 |
| SATB1 | -0 .735455648 | 8 .031813601 | -3 .2088 | 0 .00255 | 0 .031598543 |
| LOXL2 | 0 .568215464 | 7 .667429346 | 3 .20812 | 0 .00255 | 0 .03162656 |
| CCSER2 | -0 .347475739 | 8 .559343609 | -3 .2054 | 0 .00257 | 0 .031824239 |
| CCL5 | 1 .600059888 | 9 . 144784086 | 3 .20517 | 0 .00257 | 0 .031824239 |
| TOMM70A | -0 .370948488 | 9 .215278705 | -3 .2041 | 0 .00258 | 0 .031887331 |
| CKS2 | 0 .883751249 | 5 .914540379 | 3 .20324 | 0 .00259 | 0 .031933398 |
| ARID5B | -0 .579226255 | 8 .81983735 | -3 . 1984 | 0 .00262 | 0 .032336326 |
| FAM173A | 0 .61380354 | 6 .37570511 1 | 3 . 19731 | 0 .00263 | 0 .032400871 |
| IRS1 | -0 .674885375 | 6 .26577814 | -3 . 1965 | 0 .00263 | 0 .032423407 |
| SCAF8 | -0 .398966038 | 8 .096136234 | -3 . 196 | 0 .00264 | 0 .032423407 |
| CEBPG | 0 .51208433 | 6 .764317071 | 3 . 1957 | 0 .00264 | 0 .032423407 |
| TPGS2 | 0 .3300621 | 9 .060481389 | 3 . 19565 | 0 .00264 | 0 .032423407 |
| CXCR4 | 1 .063451508 | 9 .007256378 | 3 . 19415 | 0 .00265 | 0 .032527459 |
| GALNT2 | 0 .50236003 | 8 .545831383 | 3 . 19259 | 0 .00266 | 0 .032636987 |
| KDM5A | -0 .402909974 | 8 .896814986 | -3 . 1919 | 0 .00267 | 0 .032670417 |
| BMPR1B | -0 .664476215 | 5 .304128389 | -3 . 188 | 0 .0027 | 0 .032996155 |
| MLEC | 0 .558447573 | 10 .30118213 | 3 . 18752 | 0 .0027 | 0 .033003938 |
| PEBP1 | -0 .326817104 | 12 .20149629 | -3 . 1847 | 0 .00272 | 0 .033211652 |
| LRP6 | -0 .764948282 | 8 .47014539 | -3 . 1846 | 0 .00272 | 0 .033211652 |
| SOX2 | 0 .451637209 | 7 .004450917 | 3 . 18307 | 0 .00273 | 0 .0332881 |
| TMEM80 | -0 .432944297 | 8 . 166824896 | -3 . 1828 | 0 .00274 | 0 .0332881 |
| FTH1 | 0 .395684453 | 12 .25208441 | 3 . 18242 | 0 .00274 | 0 .0332881 |
| ZNF329 | -0 .524403957 | 5 .267843379 | -3 . 1823 | 0 .00274 | 0 .0332881 |
| ZMYND8 | 0 .413889617 | 9 .222504176 | 3 . 17999 | 0 .00276 | 0 .033473029 |
| EPN1 | 0 .405223756 | 6 .097273074 | 3 . 17843 | 0 .00277 | 0 .03356116 |
| FAM64A | 0 .450311 1 13 | 6 .363022363 | 3 . 17835 | 0 .00277 | 0 .03356116 |
| QPRT | 0 .925472165 | 7 . 133579724 | 3 . 17635 | 0 .00279 | 0 .033715203 |
| RNF128 | -0 .971941014 | 5 .384033512 | -3 . 1756 | 0 .00279 | 0 .033750633 |
| SLK | -0 .527218663 | 7 .80901155 | -3 . 1731 | 0 .00281 | 0 .033956611 |
| MCOLN3 | -0 .863759481 | 7 .609452985 | -3 . 1726 | 0 .00282 | 0 .033971283 |
| CHPT1 | -0 .51283334 | 8 .824747962 | -3 . 1717 | 0 .00282 | 0 .0340217 |
| HSPA12A | -0 .768841873 | 8 . 190694657 | -3 . 1705 | 0 .00283 | 0 .034075689 |
| RNF123 | 0 .386013044 | 7 .512774069 | 3 . 17044 | 0 .00283 | 0 .034075689 |
| FMR1 | -0 .615901253 | 7 .982802428 | -3 . 1688 | 0 .00284 | 0 .034193749 |
| RDH11 | -0 .543481983 | 9 .755527788 | -3 . 1682 | 0 .00285 | 0 .034226478 |
| NF2 | 0 .29707871 | 8 .835216768 | 3 . 16731 | 0 .00286 | 0 .034274249 |
| EZH1 | -0 .421296107 | 8 .497033483 | -3 . 1651 | 0 .00287 | 0 .034452085 |
| AR | -0 .463815361 | 7 .267620286 | -3 . 1644 | 0 .00288 | 0 .034478848 |
| ARPC3 | 0 .423499082 | 9 .260776454 | 3 . 16413 | 0 .00288 | 0 .034478848 |
| TEAD1 | 0 .424403805 | 7 .0217077 | 3 . 16329 | 0 .00289 | 0 .03450112 |
| MLLT10 | -0 .526955688 | 8 .719009523 | -3 . 163 | 0 .00289 | 0 .03450112 |
| NME1 | 0 .509108473 | 9 .041392799 | 3 . 16286 | 0 .00289 | 0 .03450112 |
| PIK3C2B | -1 . 131103646 | 7 .249586351 | -3 . 1624 | 0 .0029 | 0 .034502627 |
| MRPS11 | 0 .405895669 | 7 .324052581 | 3 . 16216 | 0 .0029 | 0 .034502627 |
| MBNL1 | -0 .56589597 | 10 . 17434005 | -3 . 1612 | 0 .00291 | 0 .034541254 |
| UBE2H | 0 .362471669 | 8 .387128347 | 3 . 16107 | 0 .00291 | 0 .034541254 |
| GAS6 | -0 .63651314 | 10 . 16149751 | -3 . 1601 | 0 .00291 | 0 .034604695 |
| PRKCI | -0 .369008864 | 8 .539499318 | -3 . 1579 | 0 .00293 | 0 .034766613 |
| NCS1 | 0 .484791484 | 6 .702846415 | 3 . 15755 | 0 .00294 | 0 .034766613 |
| GTF3C2 | 0 .248135752 | 9 .964980259 | 3 . 15736 | 0 .00294 | 0 .034766613 |
| RPL22 | -0 .285675201 | 13 .79535619 | -3 . 1569 | 0 .00294 | 0 .034780945 |

| TACC2 -0 .703166169 | 7 .803886779 | -3 . 1565 | 0 .00294 | 0 .034786637 |
| --- | --- | --- | --- | --- |
| SEMA3G -0 .690154332 | 6 .675869976 | -3 . 1546 | 0 .00296 | 0 .034910414 |
| RREB1 -0 .321515992 | 8 .870592438 | -3 . 1542 | 0 .00296 | 0 .034910414 |
| MALL 0 .881993191 | 7 .913589756 | 3 . 15416 | 0 .00296 | 0 .034910414 |
| SLC4A7 -0 .45360116 | 8 .324994662 | -3 . 1504 | 0 .00299 | 0 .035247276 |
| FOXD2 0 .717282494 | 6 .039174494 | 3 . 14985 | 0 .003 | 0 .035263364 |
| EXOSC4 0 .380892996 | 9 .809101185 | 3 . 14781 | 0 .00302 | 0 .03542925 |
| EHF -0 .820616823 | 5 .213809328 | -3 . 1463 | 0 .00303 | 0 .0355445 |
| THUMPD1 -0 .363786036 | 8 .454952054 | -3 . 1458 | 0 .00303 | 0 .035555343 |
| MAPKAPK3 0 .407312517 | 8 .807133411 | 3 . 14529 | 0 .00304 | 0 .035577074 |
| GPR19 1 .01581568 | 5 . 175880697 | 3 . 14296 | 0 .00306 | 0 .035773143 |
| ZNF7 -0 .603412726 | 6 .97653785 | -3 . 1422 | 0 .00306 | 0 .035816977 |
| DENND1B -0 .510518838 | 6 .085863153 | -3 . 1412 | 0 .00307 | 0 .035869889 |
| ACAD8 -0 .581528598 | 6 .803567214 | -3 . 141 | 0 .00307 | 0 .035869889 |
| SLC12A2 -0 .43008738 | 7 .977122701 | -3 . 1404 | 0 .00308 | 0 .035892624 |
| GALT -0 .578009808 | 7 .300241458 | -3 . 1378 | 0 .0031 | 0 .036107934 |
| KATNB1 0 .37896917 | 8 .540202355 | 3 . 13732 | 0 .0031 | 0 .036107934 |
| FBXO3 -0 .594749864 | 6 .511 182248 | -3 . 1372 | 0 .0031 | 0 .036107934 |
| XPO6 0 .337107976 | 9 .294448962 | 3 . 13607 | 0 .00311 | 0 .036165414 |
| WNT16 -0 .703117117 | 4 .972596533 | -3 . 1358 | 0 .00312 | 0 .036165414 |
| FAM192A -0 .377425935 | 7 . 121232402 | -3 . 1356 | 0 .00312 | 0 .036165414 |
| SGTA 0 .557710618 | 6 .946090321 | 3 . 13465 | 0 .00313 | 0 .036230739 |
| OPTN -0 .46701136 | 9 . 185941076 | -3 . 134 | 0 .00313 | 0 .036238703 |
| EEF1A2 -0 .595834625 | 7 .811592788 | -3 . 1339 | 0 .00313 | 0 .036238703 |
| ARNTL2 0 .481202012 | 6 . 180743071 | 3 . 1328 | 0 .00314 | 0 .036315732 |
| SETD1A 0 .610445 | 5 . 102527357 | 3 . 12973 | 0 .00317 | 0 .036590944 |
| CTSS 0 .92887724 | 8 .595622586 | 3 . 12881 | 0 .00318 | 0 .036649624 |
| ZFYVE26 -0 .477022523 | 8 .363029866 | -3 . 1276 | 0 .00319 | 0 .036730857 |
| TBL1XR1 -0 .719118381 | 7 .017144629 | -3 . 1273 | 0 .00319 | 0 .036730857 |
| CTSH 0 .739895582 | 9 .828258102 | 3 . 12116 | 0 .00325 | 0 .037281977 |
| ACKR4 -0 .852613322 | 4 .458299971 | -3 . 1211 | 0 .00325 | 0 .037281977 |
| PUM1 -0 .329562283 | 10 .27588756 | -3 . 1209 | 0 .00325 | 0 .037281977 |
| FBXL5 -0 .674118149 | 8 .715393587 | -3 . 12 | 0 .00326 | 0 .037342148 |
| H2BFS 0 .819283152 | 7 .553523741 | 3 . 1 1907 | 0 .00326 | 0 .037404835 |
| IFITM3 0 .385451251 | 12 .30086162 | 3 . 1 1859 | 0 .00327 | 0 .037420121 |
| DAZAP1 0 .423210451 | 8 .84016187 | 3 . 1 1794 | 0 .00327 | 0 .037444651 |
| ANKH -0 .692110484 | 3 .996989009 | -3 . 1 177 | 0 .00328 | 0 .037444651 |
| DDAH1 -0 .771922032 | 6 .749350066 | -3 . 1 168 | 0 .00328 | 0 .037498421 |
| SUPT16H -0 .420254042 | 8 .700732963 | -3 . 1 165 | 0 .00329 | 0 .037501064 |
| CAMK2N1 -0 .547060406 | 7 .398404283 | -3 . 1 156 | 0 .0033 | 0 .037541823 |
| NUP88 0 .3323494 | 7 .675125607 | 3 . 1 1532 | 0 .0033 | 0 .037541823 |
| DKFZP434L187 1 .066307185 | 3 .61045339 | 3 . 1 1501 | 0 .0033 | 0 .037541823 |
| MRPS33 -0 .588830158 | 7 .873042407 | -3 . 1 148 | 0 .0033 | 0 .037541823 |
| CD163 0 .967308856 | 8 .416829083 | 3 . 1 1382 | 0 .00331 | 0 .037606061 |
| FAM46A -0 .345286043 | 9 .051326435 | -3 . 1 124 | 0 .00332 | 0 .037720903 |
| RNF4 0 .311826487 | 8 . 173997811 | 3 . 1 1 171 | 0 .00333 | 0 .037756142 |
| COX7B -0 .757603943 | 8 .399662872 | -3 . 1 105 | 0 .00334 | 0 .03785232 |
| CNOT8 -0 .40970799 | 8 .402700067 | -3 . 1098 | 0 .00335 | 0 .037882982 |
| TMEM159 -0 .40813882 | 8 . 167986024 | -3 . 1092 | 0 .00335 | 0 .037910518 |
| SS18 0 .337126607 | 8 . 198103498 | 3 . 10851 | 0 .00336 | 0 .037937761 |
| SLC25A12 -0 .330138445 | 7 .759146136 | -3 . 1081 | 0 .00336 | 0 .037937761 |
| RP11-209A2 . 1 -0 .424207875 | 10 .45761437 | -3 . 1079 | 0 .00337 | 0 .037937761 |
| PQLC2 0 .463791031 | 6 .84019146 | 3 . 10767 | 0 .00337 | 0 .037937761 |
| BCAM -0 .626923628 | 8 .298847367 | -3 . 1057 | 0 .00339 | 0 .038104015 |
| DIO2 0 .595755517 | 9 .686271391 | 3 . 10509 | 0 .00339 | 0 .038124907 |
| PRSS2 1 .039751184 | 7 .506548474 | 3 . 1049 | 0 .00339 | 0 .038124907 |

| FAT2 -0 .749287917 | 6 .335206055 | -3 . 1044 | 0 .0034 | 0 .038147223 |
| --- | --- | --- | --- | --- |
| BST2 1 .231217636 | 8 .591347935 | 3 . 10384 | 0 .0034 | 0 .038156582 |
| C12orf43 -0 .380449602 | 6 .564670654 | -3 . 1036 | 0 .00341 | 0 .038156582 |
| ARL4A -0 .544082773 | 8 . 147852843 | -3 . 103 | 0 .00341 | 0 .038190474 |
| PSMD1 0 .364954556 | 10 .24599209 | 3 . 10233 | 0 .00342 | 0 .038198626 |
| CNNM3 -0 .349089287 | 7 .896446676 | -3 . 1021 | 0 .00342 | 0 .038198626 |
| ARHGAP10 -0 .468096933 | 6 .87330514 | -3 . 1019 | 0 .00342 | 0 .038198626 |
| TCF4 -0 .615568778 | 1 1 .24595239 | -3 . 1016 | 0 .00343 | 0 .038202645 |
| PNPLA6 0 .419299823 | 7 .962819781 | 3 . 10122 | 0 .00343 | 0 .038203683 |
| GSTA3 -0 .717939704 | 5 .416751465 | -3 .0997 | 0 .00344 | 0 .038330171 |
| RPL8 -0 .276920359 | 12 . 1 1319894 | -3 .0969 | 0 .00347 | 0 .03858921 |
| SRSF6 -0 .434666836 | 9 .062544484 | -3 .0966 | 0 .00347 | 0 .03858921 |
| LYPLA2 0 .308736916 | 10 .65107491 | 3 .09612 | 0 .00348 | 0 .038604523 |
| UBAP2L 0 .410006792 | 9 .656392215 | 3 .09556 | 0 .00348 | 0 .038629655 |
| PFN1 0 .451296866 | 1 1 .20451585 | 3 .09519 | 0 .00349 | 0 .038634818 |
| PTPRF -0 .834857138 | 9 .740414087 | -3 .0934 | 0 .0035 | 0 .038790954 |
| SLC25A32 0 .722008915 | 6 .919926291 | 3 .09142 | 0 .00352 | 0 .038934561 |
| HSPB2 -1 .430155639 | 5 .461196589 | -3 .0914 | 0 .00352 | 0 .038934561 |
| ADRB2 -0 .451467656 | 6 .814858206 | -3 .0899 | 0 .00354 | 0 .039049976 |
| CRYBB1 0 .730745672 | 3 .393963656 | 3 .08968 | 0 .00354 | 0 .039049976 |
| REL -0 .43922143 | 7 .379285047 | -3 .0865 | 0 .00357 | 0 .03932398 |
| LOC399491 -0 .600477735 | 9 .51688773 | -3 .0865 | 0 .00357 | 0 .03932398 |
| FKBP2 0 .526540774 | 8 .482472829 | 3 .08601 | 0 .00357 | 0 .039339877 |
| FAM171A1 -0 .519147396 | 7 .369226628 | -3 .0837 | 0 .0036 | 0 .039555753 |
| CDC34 0 .398198284 | 7 .309968369 | 3 .08301 | 0 .0036 | 0 .039594663 |
| FEZ1 -0 .515089394 | 8 .443011715 | -3 .0818 | 0 .00362 | 0 .03968883 |
| TUBB3 0 .53625013 | 1 1 .59160887 | 3 .0807 | 0 .00363 | 0 .039774398 |
| EIF3D -0 .402865471 | 10 .29670043 | -3 .0804 | 0 .00363 | 0 .039774398 |
| RPL36 -0 .378877283 | 1 1 .00244591 | -3 .0798 | 0 .00364 | 0 .039803509 |
| LCN2 1 .403144395 | 7 .084287586 | 3 .07887 | 0 .00364 | 0 .039870679 |
| P2RY6 0 .347209946 | 7 .447007517 | 3 .0778 | 0 .00366 | 0 .039952927 |
| PNP 0 .464817884 | 7 .018917655 | 3 .07535 | 0 .00368 | 0 .040173707 |
| IGFBP2 0 .951677549 | 7 .035321836 | 3 .075 | 0 .00368 | 0 .040173707 |
| HSD17B8 -0 .346309254 | 8 .023034997 | -3 .0748 | 0 .00369 | 0 .040173707 |
| LARP6 -0 .3551211 19 | 6 .685375719 | -3 .0736 | 0 .0037 | 0 .040268418 |
| RBFOX2 -0 .290421174 | 9 .885232469 | -3 .0701 | 0 .00373 | 0 .040572057 |
| DDX52 0 .315601641 | 7 .86892736 | 3 .06996 | 0 .00373 | 0 .040572057 |
| CD70 -0 .592867533 | 7 .586506346 | -3 .0699 | 0 .00373 | 0 .040572057 |
| C9orf1 14 0 .312540742 | 6 .89892827 | 3 .06941 | 0 .00374 | 0 .040596455 |
| SPEF1 -0 .51176808 | 5 .94301666 | -3 .0689 | 0 .00375 | 0 .040616002 |
| TOMM40 0 .473704971 | 7 .646081934 | 3 .06844 | 0 .00375 | 0 .040633243 |
| WASF2 -0 .362923949 | 8 .716628908 | -3 .0649 | 0 .00379 | 0 .040989944 |
| RPL11 -0 .416320853 | 1 1 .40416582 | -3 .0644 | 0 .00379 | 0 .041013864 |
| CROCC -0 .512718103 | 7 .929900265 | -3 .064 | 0 .0038 | 0 .041019879 |
| NOTCH2 -0 .388135507 | 10 .89846462 | -3 .058 | 0 .00386 | 0 .041644364 |
| CTNS 0 .334553937 | 8 .474508968 | 3 .05785 | 0 .00386 | 0 .041644364 |
| ATP6V0E2 0 .767903581 | 8 .454683103 | 3 .05743 | 0 .00386 | 0 .041656089 |
| EID1 -0 .564838659 | 10 .70445162 | -3 .0571 | 0 .00387 | 0 .041658146 |
| NCK2 0 .517329751 | 9 .288834283 | 3 .05671 | 0 .00387 | 0 .041665806 |
| ST3GAL1 0 .690545293 | 6 .095340456 | 3 .05563 | 0 .00388 | 0 .041753745 |
| CXCL9 1 .983617278 | 9 . 194689057 | 3 .05513 | 0 .00389 | 0 .04177435 |
| YKT6 0 .508513487 | 8 .369663754 | 3 .05433 | 0 .0039 | 0 .041829883 |
| COX5B -0 .474977096 | 1 1 .80742654 | -3 .0533 | 0 .00391 | 0 .041907402 |
| IRF7 0 .941780872 | 8 .640889123 | 3 .05074 | 0 .00394 | 0 .042167653 |
| LOC103344931 -0 .642399611 | 4 .492498228 | -3 .0504 | 0 .00394 | 0 .042171903 |
| NPR2 -0 .504114149 | 7 .924897881 | -3 .0497 | 0 .00395 | 0 .042211708 |

| VIPR1 | -0 .557154129 | 7 . 101030778 | -3 .0476 | 0 .00397 | 0 .04241839 |
| --- | --- | --- | --- | --- | --- |
| CYP4F8 | -1 .572600569 | 3 .913237536 | -3 .0465 | 0 .00398 | 0 .042510547 |
| PMF1 | 0 .348624588 | 8 .095140459 | 3 .04408 | 0 .00401 | 0 .042756971 |
| ANK3 | -0 .567008322 | 8 .40500188 | -3 .0438 | 0 .00401 | 0 .042757508 |
| TTC4 | -0 .394592607 | 6 .237377656 | -3 .0426 | 0 .00402 | 0 .042821083 |
| CDK1 | 0 .440169858 | 8 .031788116 | 3 .0422 | 0 .00403 | 0 .042821083 |
| MAST4 | -0 .573812265 | 9 .496296614 | -3 .0418 | 0 .00403 | 0 .042821083 |
| SHFM1 | 0 .467301761 | 9 .661743958 | 3 .0417 | 0 .00403 | 0 .042821083 |
| MATN4 | -0 .67539653 | 5 .227699169 | -3 .0415 | 0 .00404 | 0 .042821083 |
| ICMT | 0 .370910986 | 8 .949167707 | 3 .04108 | 0 .00404 | 0 .042821083 |
| FOXJ2 | -0 .311361758 | 7 .938307116 | -3 .0407 | 0 .00404 | 0 .042821083 |
| ASTE1 | -0 .386504166 | 6 . 187663229 | -3 .0407 | 0 .00404 | 0 .042821083 |
| FSHR | 0 .651818871 | 3 .25414571 | 3 .04015 | 0 .00405 | 0 .042825235 |
| HDDC2 | -0 .454627713 | 8 .649803902 | -3 .0401 | 0 .00405 | 0 .042825235 |
| SRPX | 0 .641323109 | 8 .955649262 | 3 .03969 | 0 .00406 | 0 .042832116 |
| LGALS9 | 0 .755196032 | 7 .291995916 | 3 .03827 | 0 .00407 | 0 .042961616 |
| SYPL1 | -0 .486112784 | 9 .637398021 | -3 .0364 | 0 .00409 | 0 .04314169 |
| MAGEA6 | 2 .099601511 | 6 .32497782 | 3 .03609 | 0 .0041 | 0 .043144622 |
| PCMT1 | -0 .454737185 | 9 .878545618 | -3 .0355 | 0 .0041 | 0 .043145601 |
| MGAT1 | 0 .370355962 | 8 .244608218 | 3 .03473 | 0 .00411 | 0 .043145601 |
| CA12 | -0 .875113912 | 10 .92988407 | -3 .0346 | 0 .00411 | 0 .043145601 |
| ABCF2 | 0 .257348686 | 8 .859514653 | 3 .03434 | 0 .00411 | 0 .043145601 |
| CYP39A1 | -0 .885603699 | 4 .228305744 | -3 .0343 | 0 .00412 | 0 .043145601 |
| GS1-124K5 .9 | -0 .735054328 | 4 .472859095 | -3 .0342 | 0 .00412 | 0 .043145601 |
| RTN3 | -0 .439271993 | 8 .831686679 | -3 .0337 | 0 .00412 | 0 .043171723 |
| TLE2 | -0 .358024189 | 8 .049196292 | -3 .0333 | 0 .00413 | 0 .043180174 |
| ABCA7 | 0 .412796299 | 6 . 198611066 | 3 .03141 | 0 .00415 | 0 .043314059 |
| FTH1P5 | 0 .33619511 1 | 12 .21075882 | 3 .03135 | 0 .00415 | 0 .043314059 |
| RPS27A | -0 .333642983 | 1 1 .74050789 | -3 .0313 | 0 .00415 | 0 .043314059 |
| RP4-781L3 . 1 | 0 .61928439 | 7 .502701508 | 3 .03095 | 0 .00415 | 0 .043314223 |
| OR2F2 | 0 .66622477 | 4 .774440869 | 3 .03006 | 0 .00416 | 0 .043383002 |
| C6orf211 | -0 .686668673 | 6 .085371224 | -3 .029 | 0 .00418 | 0 .043476827 |
| NDST1 | 0 .363254937 | 7 .964597692 | 3 .02856 | 0 .00418 | 0 .043477129 |
| SPARCL1 | -1 .039957748 | 10 .38629195 | -3 .0283 | 0 .00418 | 0 .043477129 |
| NHLRC2 | -0 .619912701 | 6 . 182101989 | -3 .0274 | 0 .00419 | 0 .043548247 |
| MPC1 | -0 .509244944 | 8 .464101141 | -3 .0262 | 0 .00421 | 0 .043645129 |
| NYNRIN | -0 .719966003 | 6 .903907268 | -3 .026 | 0 .00421 | 0 .043645129 |
| FEZ2 | -0 .347979209 | 9 .520505687 | -3 .0248 | 0 .00422 | 0 .043751603 |
| ZNF32 | -0 .497511606 | 6 .956282325 | -3 .0221 | 0 .00425 | 0 .04403398 |
| CARS | 0 .392165793 | 9 .79482435 | 3 .02058 | 0 .00427 | 0 .044181564 |
| LAG3 | 0 .720101899 | 6 .293509798 | 3 .01796 | 0 .0043 | 0 .044424294 |
| LYPD1 | 0 .730162943 | 5 .254907491 | 3 .01794 | 0 .0043 | 0 .044424294 |
| SAMD9 | 0 .52442396 | 5 .98994123 | 3 .01706 | 0 .00431 | 0 .044447387 |
| EIF4B | -0 .42721494 | 1 1 .5145362 | -3 .017 | 0 .00431 | 0 .044447387 |
| CLPB | 0 .451693108 | 6 .324320523 | 3 .01657 | 0 .00432 | 0 .044447387 |
| RUNDC3B | -0 .758519253 | 4 .490528674 | -3 .0163 | 0 .00432 | 0 .044447387 |
| GLRX5 | -0 .396777607 | 8 .32806219 | -3 .0162 | 0 .00432 | 0 .044447387 |
| GALNT10 | 0 .350382993 | 7 .63892466 | 3 .01589 | 0 .00433 | 0 .044452191 |
| TTC13 | 0 .593518977 | 5 .652717087 | 3 .01523 | 0 .00433 | 0 .044495146 |
| KLK6 | 0 .866690931 | 7 .642051691 | 3 .01426 | 0 .00434 | 0 .044575607 |
| RHBDF2 | 0 .554176152 | 7 .038495619 | 3 .01366 | 0 .00435 | 0 .044612322 |
| RPS3 | -0 .419878382 | 1 1 .65902746 | -3 .013 | 0 .00436 | 0 .04466025 |
| VAV3 | -0 .535175335 | 8 .229843829 | -3 .0117 | 0 .00437 | 0 .044771503 |
| CASS4 | 0 .795330893 | 3 .96140347 | 3 .01098 | 0 .00438 | 0 .044826646 |
| NOTCH1 | -0 .513686483 | 8 .252446011 | -3 .0103 | 0 .00439 | 0 .044867112 |
| FTSJ1 | 0 .260508456 | 8 .620855179 | 3 .00952 | 0 .0044 | 0 .044930303 |

| CAP1 | 0 .402533063 | 1 1 .63962782 | 3 .00611 | 0 .00444 | 0 .045270221 |
| --- | --- | --- | --- | --- | --- |
| SNRPF | 0 .456560393 | 8 .809539386 | 3 .00604 | 0 .00444 | 0 .045270221 |
| HDGF | 0 .29187932 | 9 .965685754 | 3 .00583 | 0 .00444 | 0 .045270221 |
| FAHD2A | -0 .45409781 | 8 .367491927 | -3 .0041 | 0 .00447 | 0 .045440307 |
| BMP1 | 0 .427775172 | 9 . 145719044 | 3 .00336 | 0 .00447 | 0 .045499029 |
| LIN7C | -0 .350022374 | 7 .876907875 | -3 .0027 | 0 .00448 | 0 .045539174 |
| C19orf73 | 0 .511620191 | 5 .019659823 | 3 .00177 | 0 .00449 | 0 .045620761 |
| LINC00302 | 0 .814659401 | 5 .867268652 | 3 .00067 | 0 .00451 | 0 .045719446 |
| PIM2 | 0 .972079844 | 6 .590689706 | 2 .99816 | 0 .00454 | 0 .045990075 |
| NLRX1 | 0 .544802035 | 8 .214060505 | 2 .99789 | 0 .00454 | 0 .045990075 |
| TRPM4 | -0 .429046762 | 6 .928048784 | -2 .9975 | 0 .00455 | 0 .045994991 |
| GLTSCR1L | -0 .479719785 | 7 .301677923 | -2 .996 | 0 .00456 | 0 .046144704 |
| FCGR3B | 0 .521344402 | 6 .622913646 | 2 .99558 | 0 .00457 | 0 .046165479 |
| MPHOSPH8 | -0 .804474929 | 6 .491031172 | -2 .9949 | 0 .00458 | 0 .046209407 |
| ZNF160 | -0 .611282945 | 9 .212820078 | -2 .9943 | 0 .00459 | 0 .04625643 |
| FAH | 0 .587532409 | 7 .589394663 | 2 .99086 | 0 .00463 | 0 .04664392 |
| TIMM9 | -0 .477823656 | 7 .905865963 | -2 .9903 | 0 .00463 | 0 .046666276 |
| RPS28 | 0 .255167457 | 12 .85031005 | 2 .99008 | 0 .00464 | 0 .046666276 |
| TBC1D8B | -0 .364978721 | 5 .926731122 | -2 .9877 | 0 .00467 | 0 .046930506 |
| DPYSL2 | -0 .712355898 | 9 .31739605 | -2 .9868 | 0 .00468 | 0 .046987138 |
| GAD1 | -0 .599484999 | 6 .336979976 | -2 .9865 | 0 .00468 | 0 .046987138 |
| TNFAIP1 | 0 .310823653 | 8 .788889188 | 2 .98635 | 0 .00468 | 0 .046987138 |
| LEPROT | -0 .478028936 | 9 .915014964 | -2 .9855 | 0 .0047 | 0 .047059928 |
| LMNB2 | 0 .422606088 | 6 .472865266 | 2 .98105 | 0 .00475 | 0 .047586399 |
| CRIP1 | -0 .919497001 | 9 .079053012 | -2 .9806 | 0 .00476 | 0 .047602843 |
| CST7 | 0 .677294154 | 7 .906962938 | 2 .98003 | 0 .00476 | 0 .047640325 |
| GSTM5 | -0 .840856398 | 7 .448405036 | -2 .9783 | 0 .00479 | 0 .047821603 |
| PARP16 | -0 .364357731 | 6 .994279153 | -2 .9766 | 0 .00481 | 0 .048001848 |
| PCDH7 | -0 .787552687 | 8 .942024205 | -2 .9763 | 0 .00481 | 0 .048004986 |
| MARCKS | 0 .416940667 | 1 1 .07968594 | 2 .97548 | 0 .00482 | 0 .048074593 |
| SASH3 | 0 .692255495 | 7 .653401182 | 2 .97456 | 0 .00484 | 0 .048155226 |
| NGDN | -0 .300064088 | 7 .660848595 | -2 .9725 | 0 .00486 | 0 .048377924 |
| TPD52L2 | 0 .584338356 | 8 .337026149 | 2 .97215 | 0 .00487 | 0 .048391293 |
| CHST10 | 0 .36691202 | 7 .404393945 | 2 .97122 | 0 .00488 | 0 .048447091 |
| WAC | -0 .314357347 | 9 . 123922359 | -2 .971 | 0 .00488 | 0 .048447091 |
| DECR2 | 0 .322110049 | 7 .940121399 | 2 .97083 | 0 .00488 | 0 .048447091 |
| DCTN3 | -0 .296213695 | 9 . 182039684 | -2 .9685 | 0 .00491 | 0 .048714285 |
| MVD | -0 .48085911 | 6 .865126042 | -2 .9682 | 0 .00492 | 0 .048716991 |
| KRT7 | -0 .932034157 | 5 .67588011 1 | -2 .9678 | 0 .00492 | 0 .048724187 |
| DLG1 | -0 .456314585 | 8 .592531038 | -2 .9658 | 0 .00495 | 0 .048901424 |
| ZNF813 | -0 .574958529 | 4 .766375508 | -2 .9657 | 0 .00495 | 0 .048901424 |
| ADAP2 | 0 .569580505 | 6 .85392979 | 2 .96554 | 0 .00495 | 0 .048901424 |
| SIRPA | 0 .389543175 | 9 .215985238 | 2 .9653 | 0 .00496 | 0 .048901424 |
| HLA-C | 0 .445870832 | 14 .27104178 | 2 .96291 | 0 .00499 | 0 .049169983 |
| COL6A3 | 0 .654483681 | 10 .44765986 | 2 .96241 | 0 .005 | 0 .049169983 |
| PRKACA | 0 .333288362 | 9 .766028714 | 2 .96238 | 0 .005 | 0 .049169983 |
| DDHD2 | -0 .551817848 | 7 .310126712 | -2 .9621 | 0 .005 | 0 .049171068 |
| LMCD1 | -0 .540191219 | 8 . 131662939 | -2 .9614 | 0 .00501 | 0 .049216283 |
| SNTB2 | -0 .325498761 | 8 .420460587 | -2 .9609 | 0 .00502 | 0 .049239343 |
| IER3 | 0 .846109778 | 9 .01022179 | 2 .96068 | 0 .00502 | 0 .049239343 |
| HLA-B | 0 .551585899 | 13 .89106717 | 2 .95948 | 0 .00504 | 0 .049359422 |
| SREK1IP1 | -0 .485053996 | 6 .563190004 | -2 .9586 | 0 .00505 | 0 .04943655 |
| PHF3 | -0 .424743337 | 8 .832360422 | -2 .9566 | 0 .00507 | 0 .04966304 |
| RP4-595K12 . 1 | -0 .375248384 | 10 .97165006 | -2 .954 | 0 .00511 | 0 .049977123 |
| COX15 | -0 .462228959 | 7 . 179141266 | -2 .9497 | 0 .00517 | 0 .050511891 |
| MOB4 | -0 .438546841 | 7 .468374219 | -2 .9492 | 0 .00518 | 0 .050545571 |

| TMED9 | 0 .384471548 | 10 .74421022 | 2 .94817 | 0 .00519 | 0 .050639722 |
| --- | --- | --- | --- | --- | --- |
| RPS25 | -0 .442837562 | 10 .87761916 | -2 .9469 | 0 .00521 | 0 .050767593 |
| GNG11 | -0 .815531217 | 6 .880607407 | -2 .9461 | 0 .00522 | 0 .050838786 |
| NSA2 | -0 .55025358 | 9 .508502945 | -2 .9438 | 0 .00525 | 0 .0511 1 121 |
| ATP5S | -0 .294025732 | 7 .828977069 | -2 .9432 | 0 .00526 | 0 .051164801 |
| COL4A1 | 0 .559348509 | 9 .407455345 | 2 .94214 | 0 .00527 | 0 .051263331 |
| DUSP4 | 0 .852825216 | 9 .536827398 | 2 .94089 | 0 .00529 | 0 .051395511 |
| ARID4A | -0 .404989483 | 6 . 172852058 | -2 .9403 | 0 .0053 | 0 .051432055 |
| HSPA4 | 0 .433436435 | 9 .543955625 | 2 .93954 | 0 .00531 | 0 .051501745 |
| KLK5 | 1 .450942699 | 8 . 1 19275427 | 2 .93752 | 0 .00534 | 0 .051719057 |
| PALMD | -0 .774349874 | 6 .954436709 | -2 .9374 | 0 .00534 | 0 .051719057 |
| COX7A2 | -0 .482134508 | 8 .985792094 | -2 .9369 | 0 .00535 | 0 .051750781 |
| PCYT1B | 0 .514209236 | 6 .893298251 | 2 .9361 | 0 .00536 | 0 .051816914 |
| FTCD | 0 .596848953 | 5 .784568026 | 2 .93567 | 0 .00537 | 0 .051820822 |
| MMRN2 | -0 .55230826 | 6 .54598807 | -2 .9355 | 0 .00537 | 0 .051820822 |
| SGK1 | 0 .673289461 | 9 .591504926 | 2 .93456 | 0 .00538 | 0 .051910175 |
| LUZP2 | 0 .726186175 | 4 . 10591082 | 2 .93268 | 0 .00541 | 0 .052130875 |
| CPSF4 | 0 .302545655 | 8 .026372663 | 2 .93215 | 0 .00542 | 0 .052164096 |
| POLL | -0 .432157162 | 6 .509124001 | -2 .9316 | 0 .00543 | 0 .052205095 |
| DNAJC1 | 0 .480009103 | 8 . 138973492 | 2 .9308 | 0 .00544 | 0 .052272242 |
| TMEM254 | -0 .585542993 | 5 .954556305 | -2 .9291 | 0 .00546 | 0 .052470557 |
| PIK3CA | -0 .543313196 | 5 .766205319 | -2 .9282 | 0 .00547 | 0 .05256228 |
| G0S2 | -0 .897202342 | 7 .025117251 | -2 .9265 | 0 .0055 | 0 .052735632 |
| RPL30 | -0 .492077989 | 1 1 .8874849 | -2 .9263 | 0 .0055 | 0 .052735632 |
| SLC35A2 | 0 .258198059 | 8 .71326555 | 2 .9248 | 0 .00552 | 0 .0528812 |
| LMOD1 | -0 .700223721 | 6 .521260513 | -2 .9242 | 0 .00553 | 0 .0528812 |
| PIGH | -0 .480038571 | 6 .685388221 | -2 .9242 | 0 .00553 | 0 .0528812 |
| TEAD4 | 0 .415743283 | 8 . 122354707 | 2 .92416 | 0 .00553 | 0 .0528812 |
| PEX5 | -0 .286315192 | 7 .963533871 | -2 .9214 | 0 .00557 | 0 .05322911 |
| USB1 | 0 .327609628 | 7 .477931067 | 2 .91957 | 0 .0056 | 0 .053450166 |
| PIGN | -0 .319948179 | 6 .583061576 | -2 .9189 | 0 .00561 | 0 .053510995 |
| ANKRD46 | -0 .517990642 | 6 .586041293 | -2 .9182 | 0 .00562 | 0 .053566281 |
| TRIM9 | -1 . 183874921 | 5 .547654361 | -2 .9166 | 0 .00565 | 0 .05368694 |
| EIF4EBP2 | -0 .483496081 | 8 .225682966 | -2 .9165 | 0 .00565 | 0 .05368694 |
| SPARC | 0 .434972305 | 12 .27506759 | 2 .91649 | 0 .00565 | 0 .05368694 |
| HAL | 0 .83325947 | 7 .436019605 | 2 .91589 | 0 .00566 | 0 .053731716 |
| DEPDC1 | 0 .421319989 | 5 .569435933 | 2 .91525 | 0 .00567 | 0 .053781744 |
| DARS | -0 .352160466 | 9 .761552365 | -2 .9145 | 0 .00568 | 0 .053849402 |
| RRAGA | -0 .4924732 | 9 .356341857 | -2 .9127 | 0 .0057 | 0 .054064965 |
| FASTKD5 | 0 .314234529 | 6 .813729508 | 2 .91189 | 0 .00572 | 0 .054142555 |
| BDH2 | -0 .492290546 | 7 .220365847 | -2 .9102 | 0 .00574 | 0 .054286672 |
| BMPR1A | -0 .372177035 | 7 .901451768 | -2 .9101 | 0 .00574 | 0 .054286672 |
| ABHD6 | -0 .373412253 | 8 .41669966 | -2 .9098 | 0 .00575 | 0 .054286672 |
| SORBS1 | -0 .492825335 | 9 .557120464 | -2 .9098 | 0 .00575 | 0 .054286672 |
| ISG20 | 1 .252507626 | 7 .393335518 | 2 .90939 | 0 .00576 | 0 .054297553 |
| RCAN3 | 0 .640105618 | 4 .224493231 | 2 .90874 | 0 .00577 | 0 .05435105 |
| PYCRL | 0 .477299669 | 5 .9427868 | 2 .9075 | 0 .00578 | 0 .054490139 |
| FMO3 | 0 .534584372 | 5 .66977219 | 2 .90698 | 0 .00579 | 0 .054523439 |
| PRSS3P2 | 0 .349954829 | 8 .390652995 | 2 .90634 | 0 .0058 | 0 .054575922 |
| HSD17B10 | 0 .329333739 | 9 .067114751 | 2 .9052 | 0 .00582 | 0 .054695043 |
| STAT5B | -0 .263041867 | 8 .340438056 | -2 .9046 | 0 .00583 | 0 .054695043 |
| ERLIN2 | -0 .421738508 | 7 .91252065 | -2 .9044 | 0 .00583 | 0 .054695043 |
| OR7C1 | 0 .794745094 | 4 .67102798 | 2 .90439 | 0 .00583 | 0 .054695043 |
| CDA | 0 .672140709 | 6 .521052663 | 2 .90398 | 0 .00584 | 0 .054713256 |
| CDKN2C | 0 .551221647 | 6 .735139258 | 2 .90257 | 0 .00586 | 0 .054878196 |
| PCP4 | -0 .88336726 | 5 .431949589 | -2 .9019 | 0 .00587 | 0 .054901886 |

| RRAGD | -0 .704121683 | 8 .767499875 | -2 .9018 | 0 .00587 | 0 .054901886 |
| --- | --- | --- | --- | --- | --- |
| CUEDC2 | -0 .358240664 | 8 .726190551 | -2 .9012 | 0 .00588 | 0 .054914341 |
| ABCD1 | 0 .394066646 | 8 . 16567586 | 2 .90105 | 0 .00588 | 0 .054914341 |
| STK17A | 0 .525082211 | 7 .916291696 | 2 .90092 | 0 .00589 | 0 .054914341 |
| COL21A1 | -0 .917779415 | 5 .403074484 | -2 .8986 | 0 .00592 | 0 .055213979 |
| DPH2 | 0 .385487669 | 7 .615880558 | 2 .89781 | 0 .00593 | 0 .055285927 |
| FBXO17 | -1 .223605847 | 4 .453174047 | -2 .8957 | 0 .00597 | 0 .055559441 |
| CLPTM1 | 0 .398997296 | 9 .878906109 | 2 .89391 | 0 .006 | 0 .055729075 |
| LSM4 | 0 .34610607 | 9 .034813692 | 2 .89379 | 0 .006 | 0 .055729075 |
| PLOD1 | 0 .662746041 | 8 . 103161088 | 2 .89346 | 0 .006 | 0 .055729075 |
| SIAH1 | -0 .426684929 | 8 .27148917 | -2 .8934 | 0 .006 | 0 .055729075 |
| DOPEY1 | -0 .334601388 | 7 . 181334083 | -2 .893 | 0 .00601 | 0 .055754199 |
| FKBPL | 0 .300310801 | 6 .902552249 | 2 .89247 | 0 .00602 | 0 .055784464 |
| TSPAN13 | -0 .719227002 | 7 . 198721208 | -2 .8906 | 0 .00605 | 0 .056021081 |
| CAV1 | -0 .474534748 | 1 1 .64692064 | -2 .8902 | 0 .00606 | 0 .056024748 |
| ARHGEF2 | 0 .318919735 | 9 .356638706 | 2 .88991 | 0 .00606 | 0 .056024748 |
| RAPGEFL1 | -1 .37065769 | 6 .297075835 | -2 .8896 | 0 .00607 | 0 .056024748 |
| APOLD1 | 0 .871580511 | 6 . 185186992 | 2 .88945 | 0 .00607 | 0 .056024748 |
| ARHGEF12 | -0 .295060344 | 9 .525617735 | -2 .8879 | 0 .00609 | 0 .056221583 |
| DCHS1 | -0 .484522933 | 7 .774755303 | -2 .8869 | 0 .00611 | 0 .056318473 |
| COPA | 0 .304360337 | 9 .684409861 | 2 .88634 | 0 .00612 | 0 .056363935 |
| THAP1 | -0 .463713732 | 5 .41351684 | -2 .886 | 0 .00612 | 0 .056367794 |
| ATF5 | 0 .35598536 | 8 .094898324 | 2 .88451 | 0 .00615 | 0 .056552239 |
| ARHGAP29 | -0 .488881369 | 6 .655587014 | -2 .8842 | 0 .00615 | 0 .056552239 |
| CD2 | 1 .005376358 | 7 .859143495 | 2 .88372 | 0 .00616 | 0 .056589257 |
| TLK1 | -0 .355432067 | 8 .268402753 | -2 .8834 | 0 .00617 | 0 .056597296 |
| RGS19 | 0 .37907288 | 7 .82288382 | 2 .88128 | 0 .0062 | 0 .056814914 |
| FOSL2 | 0 .422448237 | 8 .263530673 | 2 .88114 | 0 .0062 | 0 .056814914 |
| TYW1 | 0 .283504542 | 7 .254829304 | 2 .8811 1 | 0 .0062 | 0 .056814914 |
| PDGFC | -0 .547125997 | 6 .779950402 | -2 .8785 | 0 .00625 | 0 .057131871 |
| INSIG1 | -0 .959735059 | 8 .914995378 | -2 .8785 | 0 .00625 | 0 .057131871 |
| VASH1 | 0 .429163179 | 5 .8013211 12 | 2 .87772 | 0 .00626 | 0 .057200778 |
| SEC24D | 0 .470729541 | 6 .482643192 | 2 .877 | 0 .00627 | 0 .057262425 |
| AHDC1 | -0 .743330988 | 6 .555057179 | -2 .8768 | 0 .00627 | 0 .057262425 |
| FUCA1 | 0 .691090713 | 8 .701075438 | 2 .87615 | 0 .00629 | 0 .057314579 |
| TULP3 | -0 .374285449 | 8 .735950624 | -2 .8745 | 0 .00631 | 0 .057515466 |
| NCOA4 | -0 .415412199 | 9 .782699197 | -2 .8741 | 0 .00632 | 0 .057538996 |
| SEPHS2 | 0 .57043384 | 8 .748937869 | 2 .8731 | 0 .00634 | 0 .057644221 |
| RP11-473I1 .9 | 0 .474523401 | 5 .891624911 | 2 .87287 | 0 .00634 | 0 .057644221 |
| EFEMP1 | -0 .74279711 | 8 .298822234 | -2 .8713 | 0 .00637 | 0 .05781215 |
| SMARCD2 | -0 .268035978 | 8 . 198392698 | -2 .8712 | 0 .00637 | 0 .05781215 |
| PDE6G | -0 .524883416 | 5 . 19864959 | -2 .8708 | 0 .00637 | 0 .057833609 |
| C1orf1 15 | -0 .648480894 | 6 .864257249 | -2 .87 | 0 .00639 | 0 .057922588 |
| MTMR14 | 0 .272708218 | 8 .033299536 | 2 .86918 | 0 .0064 | 0 .057998901 |
| ZNHIT1 | 0 .482347234 | 8 . 1 18996511 | 2 .86574 | 0 .00646 | 0 .058485885 |
| IGF2BP2 | -0 .84511687 | 7 . 148980409 | -2 .865 | 0 .00647 | 0 .058549529 |
| HCG4 | 0 .744749458 | 4 .239141652 | 2 .86447 | 0 .00648 | 0 .05859613 |
| ZBTB24 | 0 .373216738 | 6 .967668419 | 2 .86379 | 0 .00649 | 0 .05865957 |
| VPS37B | 0 .507946978 | 7 .569307623 | 2 .86314 | 0 .0065 | 0 .058718013 |
| C19orf66 | 0 .321428546 | 9 .57792925 | 2 .8623 | 0 .00652 | 0 .058806276 |
| RP3-334F4 . 1 | -0 .85498228 | 6 .277505287 | -2 .8604 | 0 .00655 | 0 .059024261 |
| EFNA1 | -0 .578276512 | 7 .934470851 | -2 .8603 | 0 .00655 | 0 .059024261 |
| MPC2 | -0 .463260844 | 7 .886430701 | -2 .8592 | 0 .00657 | 0 .059091447 |
| BAG5 | -0 .314169415 | 8 .771472411 | -2 .8591 | 0 .00657 | 0 .059091447 |
| MRPL4 | 0 .328181328 | 8 .036486393 | 2 .8591 | 0 .00657 | 0 .059091447 |
| NES | 0 .587951634 | 9 .429541671 | 2 .85856 | 0 .00658 | 0 .059091982 |

0 .429903043 -0 .55459457 0 .287105459 -0 .601173994 -0 .466952554 -0 .830738593 -0 .279395405 0 .687303037 1 .047119285 0 .369946387 0 .553933083 -0 .334251207 0 .355282271 0 .33000919 0 .38819953 -0 .454022246 0 .577452016 0 .768726078 -0 .813213145 0 .602753963 0 .528470596 0 .499791137 -0 .49919745 -0 .393045816

PPIB

SGCG

CXorf40B

TIMM8B IRX5 NOTCH2NL ASXL2 NSUN5P1 CAPG MFSD5 DGAT1 KDM3B

COPE

C19orf24

BMPR2

ALKBH1

GTF2A1

TYMS

SCEL

UCN

PPM1H

ALAS1

SELT

NUCKS1

RP3-336K20 B -0 .659483888

__

TSPAN7

-0 .810415336 -0 .342025484 -0 .613290188 -0 .503455267 0 .346021509 -0 .34829672 0 .35286746 0 .471543798 -0 .256690876 0 .302645512 -0 .317201174 0 .57699105 -0 .486347794 0 .637715452 0 .473300725 -0 .344262434 -0 .3659583 -0 .574271582 0 .567350034 -0 .382611443 -0 .616043036 0 .484345069 -0 .472723034 0 .371613713 0 .442437778 -0 .556463281 0 .451068868 0 .403876024 0 .528752376 0 .51476413 0 .262522817

CIZ1

PLIN1

LINC01278

AP3D1

SH3PXD2A

PPP3R1

ASCC2

DCAF8

ABCC1

AHCYL1

FOLR2

NKTR

SAMSN1

GSPT1

HBP1

DDAH2

FAM35A

SLC39A4

CD46

PXMP2

MRTO4

ZC2HC1A

PLEKHA2

BUD31

SUZ12

PLAC1

CST4

HLA-G

HLA-J

OXLD1

1 1 .23966775 4 .250812319 7 .898868959 8 .364615787 7 .310626683 8 .307889116 8 .65379021 8 .688132406 8 .707620645 7 .773918013 7 .244304368 8 .975794411 9 . 192386497 7 .369252553 8 .04059742 7 .429316969 6 .00008482 8 .808741626 6 .262883757 4 .294714943 6 .06178138 7 .913265937 7 .392683349 10 .25630148 3 .854803497 8 .32105308 9 .920164083 5 .798320644 6 .654717559 10 .37121559 7 .917516051 6 .326384364 6 .924418283 9 .327428213 8 . 18406517 10 .55163884 7 .815921086 9 .487926101 5 . 133396491 8 .696073933 8 .632782654 9 .238839323 7 .637626036 7 .652039047 8 .920768341 6 .536054658 7 . 149330897 7 .776088035 7 .802770333 7 .952630363 7 .47978686 6 .651613544 6 .270472449 13 .5966487 1 1 . 10091936 7 .904274065

2 .85855 -2 .8582 2 .85675 -2 .8555 -2 .8541 -2 .8534 -2 .8534 2 .85324 2 .85225 2 .85158 2 .85053 -2 .8483 2 .84745 2 .84652 2 .84383 -2 .8424 2 .84157 2 .83919 -2 .8386 2 .8384 2 .83838 2 .83823 -2 .8368 -2 .8355 -2 .8337 -2 .8335 -2 .8323 -2 .8317 -2 .8317 2 .83123 -2 .8288 2 .82799 2 .82678 -2 .8248 2 .82445 -2 .8243 2 .82255 -2 .8198 2 .81711 2 .81698 -2 .8169 -2 .8163 -2 .8163 2 .81572 -2 .8157 -2 .815 2 .81391 -2 .8134 2 .81282 2 .81268 -2 .8124 2 .81193 2 .81121 2 .81069 2 .81065 2 .80891

0 .00658 0 .00659 0 .00661 0 .00664 0 .00666 0 .00667 0 .00667 0 .00668 0 .00669 0 .00671 0 .00672 0 .00676 0 .00678 0 .0068 0 .00684 0 .00687 0 .00688 0 .00693 0 .00694 0 .00694 0 .00694 0 .00694 0 .00697 0 .00699 0 .00703 0 .00703 0 .00705 0 .00706 0 .00707 0 .00707 0 .00712 0 .00713 0 .00716 0 .00719 0 .0072 0 .0072 0 .00724 0 .00729 0 .00734 0 .00734 0 .00734 0 .00736 0 .00736 0 .00737 0 .00737 0 .00738 0 .0074 0 .00741 0 .00742 0 .00742 0 .00743 0 .00744 0 .00745 0 .00746 0 .00746 0 .0075

0 .059091982

0 .059101811

0 .059287743

0 .059441639

0 .059619672

0 .0596253

0 .0596253

0 .0596253

0 .059738086

0 .05980132

0 .059924114

0 .060225807

0 .060324922

0 .06042906

0 .060814722

0 .060997602

0 .061090169

0 .061410573

0 .061410573

0 .061410573

0 .061410573

0 .061410573

0 .061605384

0 .061766692

0 .06199991

0 .06199991

0 .062156682

0 .062170998

0 .062170998

0 .062198099

0 .062545925

0 .062639306

0 .062795391

0 .063071008

0 .063071008

0 .063071008

0 .063316292

0 .063730465

0 .064081812

0 .064081812

0 .064081812

0 .064098121

0 .064098121

0 .064105133

0 .064105133

0 .064170644

0 .064314325

0 .064347583

0 .064386986

0 .064386986

0 .06438908

0 .06442465

0 .06450056

0 .064506882

0 .064506882

0 .064739504

-0 .678296789 -0 .563008687 -0 .619283232 -0 .431859999 -0 .552409772 0 .386682919 -0 .329908464 -1 .280444715 -0 .843539313 -0 .316523305 0 .487565188 -0 .4511 10059 0 .461848214 -0 .824936433 0 .729018062 0 .379810803 0 .368856335 -0 .771392339 -0 .376684404 0 .377915667 -0 .437893282 -0 .476599264 -0 .488574347 0 .897485607

RIN2 ART4 GLCE EMG1 COPS2 TXNL4A GPBP1L1 PCK1 LPHN3 SPTLC1 MAD2L1 HOXA5 MCM5 PECR SHARPIN P4HB MAPK11 ATP1B1 ABCB7 SLC36A1 HEBP1 WEE1 OAT ACPP

LOC101929148 -0 .560986828

0 .37105363 0 .260627982 -0 .728277919 0 .633885731 0 .877047427 -0 .533365309 0 .553779947 0 .38150084 0 .29398059 -0 .387646116 0 .749400214 0 .360088938 0 .418246479 -0 .455516524 0 .558280151 -0 .32557991 0 .370128113 -0 .482360361 0 .347672173 -0 .467752643 0 .354925627 0 .51178526 0 .347054859 0 .556193596 0 .262033438 -0 .403807202 -0 .485775783 0 .346338346 -0 .466596806 -0 .742807348 0 .882918094

LEPRE1 PTBP1 KCTD12 SLC7A11 PRR16 RERGL ZWINT EIF6 SUGP1 PTPN14 EPB41 AK1 COPB2 HES1 NELL2 OCRL ARPC5L RSL1D1 RAB5C CROT STX2 FOXD1 TALDO1 MARCKSL1 CAPNS1 COX11 SERINC1 CKAP2 SP3 RAPSN PLAT

7 .667258418 4 .665264829 5 .830281498 7 .012642326 8 .726242075 9 .555054486 8 . 175403209 2 .345044669 6 .395433639 8 .950396722 6 .769705438 6 .713578475 8 .57725175 5 .300003791 6 .413724318 1 1 .44508461 7 .882093681 9 .933493095 7 .09713749 6 .721382653 9 .086937453 7 .778692461 9 .3836811 13 5 .48009043 4 .585786634 7 .738539021 10 .02296114 9 .541696858 7 .404253138 3 .848270164 4 .211441308 7 .248077702 9 .586003277 6 .480097423 5 .943541971 5 .261946892 8 . 168817867 8 .757503087 10 . 17477713 7 .439376286 7 .546251442 9 .012606506 9 .022990123 9 .666850223 7 .900382631 6 .514698968 5 .893222449 10 . 14999773 10 .45021433 10 .41764718 8 .033222165 9 .358444736 6 .750884717 8 . 138260078 2 .690618554 8 .875294343

-2 .8087 -2 .8078 -2 .8072 -2 .8071 -2 .8071 2 .80607 -2 .8055 -2 .8043 -2 .8029 -2 .8028 2 .80208 -2 .8015 2 .80124 -2 .8012 2 .79829 2 .79807 2 .79742 -2 .7952 -2 .7946 2 .79451 -2 .7931 -2 .7916 -2 .7912 2 .79088 -2 .7909 2 .7888 2 .78716 -2 .7871 2 .78566 2 .78487 -2 .7844 2 .78296 2 .78247 2 .78221 -2 .7812 2 .78065 2 .78006 2 .78001 -2 .7788 2 .77873 -2 .7775 2 .77671 -2 .7761 2 .77355 -2 .7734 2 .77323 2 .77314 2 .77305 2 .77271 2 .7725 -2 .7714 -2 .7706 2 .77027 -2 .7692 -2 .7686 2 .7673

0 .0075 0 .00752 0 .00753 0 .00753 0 .00753 0 .00755 0 .00757 0 .00759 0 .00762 0 .00762 0 .00763 0 .00765 0 .00765 0 .00765 0 .00771 0 .00771 0 .00773 0 .00777 0 .00778 0 .00778 0 .00781 0 .00784 0 .00785 0 .00786 0 .00786 0 .0079 0 .00794 0 .00794 0 .00797 0 .00798 0 .00799 0 .00802 0 .00803 0 .00804 0 .00806 0 .00807 0 .00808 0 .00808 0 .00811 0 .00811 0 .00814 0 .00815 0 .00817 0 .00822 0 .00822 0 .00823 0 .00823 0 .00823 0 .00824 0 .00824 0 .00827 0 .00828 0 .00829 0 .00831 0 .00833 0 .00835

0 .064739504

0 .064840674

0 .064840674

0 .064840674

0 .064840674

0 .064969362

0 .065021087

0 .065174178

0 .065339825

0 .065339825

0 .065423953

0 .065439806

0 .065439806

0 .065439806

0 .065886269

0 .065886269

0 .065951928

0 .066291717

0 .066317449

0 .066317449

0 .066523805

0 .066724454

0 .066724454

0 .066724454

0 .066724454

0 .067037169

0 .06724164

0 .06724164

0 .067449145

0 .067542502

0 .067575655

0 .067787407

0 .067827251

0 .067828516

0 .0679595

0 .06801147

0 .068033076

0 .068033076

0 .06816734

0 .06816734

0 .068340888

0 .068434881

0 .068501665

0 .068811 108

0 .068811 108

0 .068811 108

0 .068811 108

0 .068811 108

0 .068817321

0 .068817321

0 .068961894

0 .069070845

0 .069077264

0 .069220086

0 .069278267

0 .069472114

| CAPN15 | 0 .498090238 | 7 .049227497 | 2 .76656 | 0 .00837 | 0 .069560377 |
| --- | --- | --- | --- | --- | --- |
| FAM149A | -0 .798999391 | 7 .272585502 | -2 .7663 | 0 .00838 | 0 .069565279 |
| LANCL1 | -0 .318450018 | 8 .560489044 | -2 .7659 | 0 .00838 | 0 .069586097 |
| RASSF4 | 0 .650761367 | 7 . 102782647 | 2 .76538 | 0 .0084 | 0 .069634061 |
| BMP2K | 0 .44117312 | 7 .617050965 | 2 .76376 | 0 .00843 | 0 .069881648 |
| VASP | 0 .421387872 | 7 .949153361 | 2 .76267 | 0 .00846 | 0 .070026859 |
| CMTM6 | -0 .41355 | 9 .068031013 | -2 .7624 | 0 .00846 | 0 .070026859 |
| BAK1 | 0 .485194584 | 7 . 196224322 | 2 .76114 | 0 .00849 | 0 .07021755 |
| MAP1LC3B | 0 .378850029 | 9 .884627601 | 2 .75997 | 0 .00851 | 0 .070360248 |
| NBR2 | -0 .672257354 | 4 .40265925 | -2 .7598 | 0 .00852 | 0 .070360248 |
| TNPO2 | -0 .337738451 | 9 .582684876 | -2 .7594 | 0 .00853 | 0 .070365825 |
| RUNX1T1 | -0 .375153046 | 8 .391151369 | -2 .7593 | 0 .00853 | 0 .070365825 |
| RNPEPL1 | 0 .434422723 | 7 .019889295 | 2 .75718 | 0 .00858 | 0 .07070585 |
| LMF1 | 0 .345068633 | 8 .201343048 | 2 .75595 | 0 .0086 | 0 .070884458 |
| ATG9A | 0 .339353419 | 7 .75011 1744 | 2 .75553 | 0 .00861 | 0 .070905209 |
| PEX3 | -0 .326729389 | 8 .430216872 | -2 .7553 | 0 .00862 | 0 .070905209 |
| CTDSP1 | -0 .328208319 | 9 .231318548 | -2 .7547 | 0 .00863 | 0 .070975028 |
| TADA3 | 0 .319741698 | 8 .380592797 | 2 .75345 | 0 .00866 | 0 .071127937 |
| LILRB2 | 0 .4217382 | 6 .930741814 | 2 .75335 | 0 .00866 | 0 .071127937 |
| ABCB1 | -0 .386289195 | 5 .642577582 | -2 .7529 | 0 .00867 | 0 .071162305 |
| LRRC37BP1 | 1 .053166047 | 2 .901352967 | 2 .75236 | 0 .00868 | 0 .071217611 |
| MAPRE1 | 0 .345626112 | 9 .908652063 | 2 .75107 | 0 .00871 | 0 .07137357 |
| TRPV6 | -0 .614120049 | 4 .054516025 | -2 .751 | 0 .00871 | 0 .07137357 |
| VAV1 | 0 .390356588 | 6 .786549802 | 2 .74919 | 0 .00875 | 0 .071661006 |
| ABCA11P | -0 .374326528 | 5 .513169157 | -2 .7489 | 0 .00876 | 0 .071672753 |
| GSTM3 | -0 .474059041 | 8 .515213755 | -2 .7486 | 0 .00877 | 0 .071678684 |
| PRRC1 | 0 .417711081 | 8 .294574236 | 2 .74806 | 0 .00878 | 0 .071724708 |
| TMCC2 | 0 .621304647 | 6 .884622193 | 2 .74784 | 0 .00879 | 0 .071724708 |
| S100A10 | 0 .291086086 | 1 1 .7422301 | 2 .74709 | 0 .0088 | 0 .071817092 |
| SCNN1B | -0 .631931666 | 6 .201447194 | -2 .7459 | 0 .00883 | 0 .071987035 |
| SIK1 | -0 .520901307 | 7 .51604682 | -2 .7447 | 0 .00886 | 0 .072123042 |
| GMPR2 | -0 .302499171 | 7 . 180045749 | -2 .7447 | 0 .00886 | 0 .072123042 |
| MYL12A | 0 .356042155 | 8 .930117761 | 2 .74434 | 0 .00887 | 0 .072139927 |
| MAPKAP1 | 0 .319353646 | 7 .591202191 | 2 .74343 | 0 .00889 | 0 .072262711 |
| FN1 | 0 .828738445 | 12 .34877072 | 2 .74222 | 0 .00891 | 0 .072442174 |
| NR2E1 | -0 .530472348 | 5 .46272463 | -2 .7416 | 0 .00893 | 0 .072512537 |
| NACA | -0 .29804208 | 13 . 14523261 | -2 .7404 | 0 .00896 | 0 .07268864 |
| ZNF91 | -0 .552130949 | 8 .213393419 | -2 .7397 | 0 .00897 | 0 .072776475 |
| DNMT1 | 0 .576626465 | 8 .316186877 | 2 .73816 | 0 .00901 | 0 .072974569 |
| HEXB | 0 .452084878 | 9 .553784735 | 2 .73786 | 0 .00901 | 0 .072974569 |
| GCHFR | -0 .351652495 | 6 .345436065 | -2 .7378 | 0 .00902 | 0 .072974569 |
| MCM10 | 0 .398387375 | 4 .82651982 | 2 .73762 | 0 .00902 | 0 .072974569 |
| ALOX15B | -1 .540735363 | 7 .840412067 | -2 .7365 | 0 .00905 | 0 .073142337 |
| CDK2AP1 | 0 .376723176 | 10 .38561376 | 2 .73547 | 0 .00907 | 0 .073284864 |
| TMEM59 | -0 .511599382 | 9 .306228958 | -2 .7349 | 0 .00908 | 0 .073297677 |
| MKI67 | 0 .37412453 | 8 .786439817 | 2 .7349 | 0 .00908 | 0 .073297677 |
| SEC63 | -0 .400529977 | 9 .531930242 | -2 .7337 | 0 .00911 | 0 .073478228 |
| UBC | 0 .258865984 | 13 .63488758 | 2 .7332 | 0 .00912 | 0 .073523842 |
| TYROBP | 0 .964690291 | 8 .912207769 | 2 .73227 | 0 .00915 | 0 .073619506 |
| MKRN2 | -0 .467499579 | 7 .361039304 | -2 .7321 | 0 .00915 | 0 .073619506 |
| COX6B1 | 0 .321667815 | 10 .79787664 | 2 .73195 | 0 .00915 | 0 .073619506 |
| CELA3A | 0 .453775732 | 6 .817562499 | 2 .73125 | 0 .00917 | 0 .073682486 |
| GMPPA | 0 .465849646 | 6 .946538714 | 2 .7311 1 | 0 .00917 | 0 .073682486 |
| LRP8 | 0 .36009249 | 7 .979424757 | 2 .73014 | 0 .0092 | 0 .073792037 |
| C11orf95 | -0 .382716893 | 6 .734963576 | -2 .73 | 0 .0092 | 0 .073792037 |
| MRPL33 | -0 .432559895 | 8 .46618791 | -2 .7296 | 0 .00921 | 0 .07382256 |

| TOP2A | 0 .78297085 | 7 .64787124 | 2 .72898 | 0 .00922 | 0 .07382256 |
| --- | --- | --- | --- | --- | --- |
| PRR15L | -0 .707536089 | 5 .857658707 | -2 .7289 | 0 .00922 | 0 .07382256 |
| SWAP70 | -0 .69000367 | 8 .908628071 | -2 .7289 | 0 .00922 | 0 .07382256 |
| NEO1 | -0 .410013808 | 7 .630128144 | -2 .728 | 0 .00925 | 0 .07394292 |
| CDKN3 | 0 .404679781 | 6 .48802172 | 2 .72698 | 0 .00927 | 0 .074089394 |
| KIF3C | 0 .386368589 | 8 . 181245161 | 2 .72644 | 0 .00928 | 0 .07411595 |
| FADS2 | -1 .792328807 | 8 .576480596 | -2 .7263 | 0 .00929 | 0 .07411595 |
| ZFP36L2 | -0 .489040046 | 10 .9317082 | -2 .7252 | 0 .00931 | 0 .074289718 |
| PARPBP | 0 .362680723 | 5 .39196022 | 2 .72456 | 0 .00933 | 0 .074361834 |
| EPHB3 | -0 .333252191 | 8 .446463688 | -2 .7241 | 0 .00934 | 0 .074400901 |
| OGFOD3 | 0 .285070611 | 8 .648181032 | 2 .72378 | 0 .00935 | 0 .074417115 |
| EIF3G | -0 .365744877 | 9 .867762042 | -2 .7227 | 0 .00937 | 0 .074568878 |
| ARSA | 0 .374874665 | 7 .026748905 | 2 .72168 | 0 .0094 | 0 .074696888 |
| PIN1 | -0 .278397656 | 8 .344875212 | -2 .7216 | 0 .0094 | 0 .074696888 |
| HCN2 | 0 .737924772 | 6 . 176314256 | 2 .72126 | 0 .00941 | 0 .074710139 |
| CDKN1B | -0 .440358773 | 7 .618027116 | -2 .7209 | 0 .00941 | 0 .074712065 |
| IER2 | 0 .517400407 | 9 .768465378 | 2 .72076 | 0 .00942 | 0 .074712065 |
| FADS3 | 0 .427998839 | 8 . 1 14007731 | 2 .71798 | 0 .00949 | 0 .075178739 |
| WSB1 | -0 .499745404 | 9 .395886234 | -2 .7178 | 0 .00949 | 0 .075178739 |
| PLCB2 | 0 .298966559 | 6 .917210431 | 2 .71724 | 0 .0095 | 0 .075245508 |
| BAX | 0 .523819762 | 7 .469784184 | 2 .71673 | 0 .00952 | 0 .075296746 |
| ENTPD4 | 0 .332427458 | 7 .830788507 | 2 .71545 | 0 .00955 | 0 .075451225 |
| ARID5A | 0 .487308343 | 7 . 106998835 | 2 .7154 | 0 .00955 | 0 .075451225 |
| OR1G1 | 0 .769059826 | 4 .491419395 | 2 .7152 | 0 .00955 | 0 .075451225 |
| PLXNA3 | -0 .321070307 | 6 .094711249 | -2 .7143 | 0 .00958 | 0 .075584704 |
| EVX1 | 0 .92256973 | 5 .260073839 | 2 .71395 | 0 .00959 | 0 .075596337 |
| METTL7A | -0 .554315933 | 8 .873044043 | -2 .7129 | 0 .00961 | 0 .075711085 |
| KDELR3 | 0 .41204119 | 9 .038568371 | 2 .71285 | 0 .00961 | 0 .075711085 |
| IL1RL2 | -0 .478062854 | 4 .770065438 | -2 .7125 | 0 .00962 | 0 .075711085 |
| FAM13B | -0 .577948743 | 7 . 1 13507443 | -2 .7124 | 0 .00962 | 0 .075711085 |
| CCNA2 | 0 .356143126 | 6 .68625334 | 2 .70946 | 0 .0097 | 0 .076232909 |
| MRPS27 | -0 .410929766 | 8 . 1 19413868 | -2 .709 | 0 .00971 | 0 .076252132 |
| ZHX2 | -0 .493205803 | 6 .777467379 | -2 .7087 | 0 .00971 | 0 .076252132 |
| CYBB | 0 .327106514 | 7 .814683526 | 2 .70839 | 0 .00972 | 0 .076252132 |
| ZGPAT | 0 .490328378 | 7 .381008459 | 2 .70838 | 0 .00972 | 0 .076252132 |
| ZNF586 | -0 .320030586 | 6 .9594211 12 | -2 .7061 | 0 .00978 | 0 .076641068 |
| COA3 | 0 .299613916 | 9 .407520813 | 2 .70508 | 0 .00981 | 0 .076767677 |
| IFI27 | 1 .031488893 | 1 1 .02068865 | 2 .70501 | 0 .00981 | 0 .076767677 |
| MFGE8 | 0 .596965769 | 8 .793801686 | 2 .70455 | 0 .00982 | 0 .076810005 |
| SEPW1 | -0 .430552579 | 9 .569332737 | -2 .7034 | 0 .00985 | 0 .076882709 |
| JUND | 0 .300083214 | 1 1 .52369515 | 2 .70307 | 0 .00986 | 0 .076882709 |
| TNFRSF4 | 0 .356784439 | 7 .083184262 | 2 .70275 | 0 .00986 | 0 .076882709 |
| UQCRB | -0 .457650823 | 1 1 .38788543 | -2 .7027 | 0 .00986 | 0 .076882709 |
| TSTA3 | 0 .371177648 | 9 .731024242 | 2 .70265 | 0 .00987 | 0 .076882709 |
| DKK2 | -0 .765484362 | 4 .712165654 | -2 .7026 | 0 .00987 | 0 .076882709 |
| IGFBP5 | -0 .705580838 | 10 .24075884 | -2 .7024 | 0 .00987 | 0 .076882709 |
| TES | -0 .332153992 | 9 .761876745 | -2 .7022 | 0 .00988 | 0 .076882709 |
| IGSF6 | 0 .963315214 | 5 .974021782 | 2 .70143 | 0 .0099 | 0 .076993992 |
| LCN1 | 0 .393969655 | 5 .501418461 | 2 .6999 | 0 .00994 | 0 .077173656 |
| GALNS | 0 .318564291 | 7 .369903979 | 2 .6999 | 0 .00994 | 0 .077173656 |
| CHCHD3 | 0 .429793561 | 8 .272670632 | 2 .69971 | 0 .00994 | 0 .077173656 |
| LOC100287590 | 0 .643941601 | 4 .26776993 | 2 .69955 | 0 .00994 | 0 .077173656 |
| TENC1 | -0 .362081458 | 7 .658373075 | -2 .698 | 0 .00998 | 0 .077424756 |
| NRSN2 | 0 .441108684 | 7 . 123769938 | 2 .69765 | 0 .00999 | 0 .077440209 |
| ZBED8 | -0 .45494551 | 4 .85840474 | -2 .6974 | 0 .01 | 0 .077440209 |
| PSD3 | -0 .567118106 | 7 .468933666 | -2 .697 | 0 .01001 | 0 .077440209 |

| PELI2 -0 .418397547 | 6 .246752791 | -2 .697 | 0 .01001 | 0 .077440209 |
| --- | --- | --- | --- | --- |
| TBX3 -0 .939124623 | 5 .034402557 | -2 .6967 | 0 .01002 | 0 .077440209 |
| CNTN1 -0 .738708798 | 6 .005854896 | -2 .6965 | 0 .01002 | 0 .077449254 |
| TFAP2A 0 .502651904 | 10 .65750382 | 2 .69556 | 0 .01005 | 0 .077580151 |
| PTP4A3 0 .525653904 | 8 .464151282 | 2 .69451 | 0 .01007 | 0 .077740301 |
| MAFF 0 .526840213 | 8 .087800463 | 2 .69357 | 0 .0101 | 0 .077878338 |
| ANKRD49 -0 .377048297 | 7 .075071931 | -2 .6933 | 0 .0101 | 0 .077880599 |
| PIGB 0 .294865749 | 7 .811597587 | 2 .69216 | 0 .01013 | 0 .078038037 |
| KRCC1 -0 .500108161 | 7 . 16279554 | -2 .692 | 0 .01014 | 0 .078038037 |
| ROBO1 -0 .542352592 | 8 . 103288295 | -2 .6916 | 0 .01015 | 0 .078072628 |
| TYMP 0 .737638203 | 8 .884435895 | 2 .69115 | 0 .01016 | 0 .078120398 |
| BIRC5 0 .373917044 | 8 . 16585993 | 2 .69076 | 0 .01017 | 0 .078151857 |
| RFC2 0 .304286421 | 7 .711236302 | 2 .68737 | 0 .01026 | 0 .078781388 |
| RNASE1 0 .801243905 | 9 .62766075 | 2 .68686 | 0 .01027 | 0 .078835531 |
| JADE1 -0 .401723993 | 7 .53174378 | -2 .6864 | 0 .01029 | 0 .078885235 |
| SERPINB1 0 .457358455 | 8 .202248291 | 2 .68411 | 0 .01034 | 0 .079293294 |
| PANX1 1 .004342653 | 6 .497374211 | 2 .68189 | 0 .0104 | 0 .079665706 |
| NFKBIE 0 .533865301 | 7 . 107624184 | 2 .68179 | 0 .01041 | 0 .079665706 |
| RP11-348B17 . 1 -0 .468991984 | 5 .340147553 | -2 .681 | 0 .01043 | 0 .079740124 |
| U2AF2 0 .25726315 | 8 .991942403 | 2 .68094 | 0 .01043 | 0 .079740124 |
| SNX3 -0 .358240318 | 1 1 .37784733 | -2 .6805 | 0 .01044 | 0 .079765178 |
| PNISR -0 .471876519 | 8 . 140849284 | -2 .6803 | 0 .01045 | 0 .079765178 |
| COMMD3 -0 .406686912 | 7 .794326944 | -2 .6801 | 0 .01045 | 0 .079765178 |
| POLD2 0 .299374067 | 8 .511247586 | 2 .67976 | 0 .01046 | 0 .079785156 |
| PSMB3 0 .367608707 | 9 .722463637 | 2 .67933 | 0 .01047 | 0 .079824526 |
| DYNLRB1 0 .283682269 | 1 1 .26672478 | 2 .67891 | 0 .01048 | 0 .079842423 |
| PTGDS 0 .820634123 | 12 .6064078 | 2 .67859 | 0 .01049 | 0 .079842423 |
| UBB 0 .249330349 | 12 .30951893 | 2 .6783 | 0 .0105 | 0 .079842423 |
| RUFY2 -0 .677799466 | 5 . 104679252 | -2 .6783 | 0 .0105 | 0 .079842423 |
| NRBF2 0 .534355329 | 6 .079602487 | 2 .67797 | 0 .01051 | 0 .079858369 |
| ALOX12 0 .481830727 | 6 .59042541 | 2 .67654 | 0 .01055 | 0 .08010007 |
| ANK2 -0 .462462825 | 8 .402295457 | -2 .6753 | 0 .01058 | 0 .080312018 |
| PDZK1 -0 .817521192 | 5 . 145848776 | -2 .675 | 0 .01059 | 0 .080323595 |
| SEMA6A 0 .572035408 | 7 .728603552 | 2 .67346 | 0 .01063 | 0 .080583401 |
| SLC38A6 0 .531560969 | 4 .846757636 | 2 .67225 | 0 .01066 | 0 .080719236 |
| ZNF264 -0 .279861569 | 6 .977883662 | -2 .6722 | 0 .01066 | 0 .080719236 |
| KPNA4 -0 .271857338 | 7 .518088691 | -2 .6721 | 0 .01067 | 0 .080719236 |
| ZNF706 0 .357181544 | 9 .637651574 | 2 .67175 | 0 .01067 | 0 .080739957 |
| TFB1M -0 .301351167 | 7 .30495754 | -2 .6712 | 0 .01069 | 0 .080782469 |
| MRAS 0 .89810782 | 5 .093818482 | 2 .6709 | 0 .0107 | 0 .080782469 |
| KLHL9 -0 .387729257 | 8 .539524988 | -2 .6708 | 0 .0107 | 0 .080782469 |
| CREBL2 -0 .415762901 | 9 .585712586 | -2 .6704 | 0 .01071 | 0 .080832713 |
| SMPD1 0 .269151895 | 8 .747899954 | 2 .67009 | 0 .01072 | 0 .080837001 |
| CIRBP -0 .433478698 | 1 1 .44979119 | -2 .6691 | 0 .01075 | 0 .080992174 |
| SCRIB 0 .402286091 | 7 .343167853 | 2 .66839 | 0 .01077 | 0 .081090448 |
| MSRB2 -0 .511 196635 | 9 .516818151 | -2 .668 | 0 .01078 | 0 .081130322 |
| ERH -0 .368787141 | 9 .693922029 | -2 .667 | 0 .0108 | 0 .081247791 |
| GPR183 0 .748537964 | 5 .499972841 | 2 .66671 | 0 .01081 | 0 .081247791 |
| NPR3 -0 .521705505 | 7 .49374168 | -2 .6664 | 0 .01082 | 0 .081247791 |
| CXCR3 0 .750528218 | 7 .3865415 | 2 .66636 | 0 .01082 | 0 .081247791 |
| ARF6 0 .340239743 | 9 .48722746 | 2 .66621 | 0 .01083 | 0 .081247791 |
| BAG1 -0 .280650281 | 9 .236498544 | -2 .6626 | 0 .01092 | 0 .081934612 |
| TOMM34 0 .281030173 | 7 .996781639 | 2 .66221 | 0 .01094 | 0 .081934612 |
| ELK2AP 0 .506081262 | 5 .601008996 | 2 .66218 | 0 .01094 | 0 .081934612 |
| AUP1 0 .381527798 | 8 .638867583 | 2 .65995 | 0 .011 | 0 .082349543 |
| CAB39L -0 .874876458 | 3 .517150558 | -2 .6597 | 0 .01101 | 0 .082355953 |

| PTGES3 | -0 .422179044 | 9 .808226826 | -2 .6594 | 0 .01101 | 0 .082364881 |
| --- | --- | --- | --- | --- | --- |
| UNC93B1 | 0 .421325867 | 6 .857300581 | 2 .65795 | 0 .01106 | 0 .08260599 |
| CTBS | -0 .508282316 | 7 .264282615 | -2 .6578 | 0 .01106 | 0 .08260599 |
| LTA | 1 .446026871 | 4 .437380063 | 2 .6573 | 0 .01107 | 0 .082618768 |
| TBK1 | -0 .414468801 | 7 .993311857 | -2 .6573 | 0 .01107 | 0 .082618768 |
| EIF3A | -0 .402535525 | 10 .28146956 | -2 .6565 | 0 .011 1 | 0 .082710413 |
| DES | -0 .699763615 | 7 .013541495 | -2 .656 | 0 .011 1 1 | 0 .082710413 |
| SNRPB | 0 .308208789 | 10 .84700229 | 2 .65599 | 0 .011 1 1 | 0 .082710413 |
| CAV2 | -0 .465407164 | 9 .472777041 | -2 .6559 | 0 .011 1 1 | 0 .082710413 |
| SRSF8 | -0 .361201195 | 9 .398043462 | -2 .6554 | 0 .011 13 | 0 .082752489 |
| MED23 | -0 .402961688 | 6 .396308967 | -2 .6547 | 0 .011 15 | 0 .082842587 |
| DPAGT1 | 0 .45397555 | 7 .728506266 | 2 .65455 | 0 .011 15 | 0 .082842587 |
| TMEM50A | 0 .329329068 | 8 .037243882 | 2 .65359 | 0 .011 18 | 0 .082995234 |
| SLC9A2 | 0 .596433107 | 4 .236940039 | 2 .65311 | 0 .011 19 | 0 .083046063 |
| CEP131 | 0 .46959911 1 | 5 .852195117 | 2 .65269 | 0 .0112 | 0 .083086151 |
| ATP5L | -0 .3990646 | 1 1 .6560355 | -2 .652 | 0 .01122 | 0 .083107875 |
| CTTN | 0 .309412686 | 10 .02022776 | 2 .65195 | 0 .01122 | 0 .083107875 |
| CLDN14 | 0 .492801081 | 4 .910758033 | 2 .65171 | 0 .01123 | 0 .083107875 |
| GABARAPL1 | -0 .342675724 | 7 .527115769 | -2 .6516 | 0 .01123 | 0 .083107875 |
| SCO2 | 0 .424920449 | 8 . 1 1 1070902 | 2 .65122 | 0 .01125 | 0 .083107875 |
| ELTD1 | -0 .721803205 | 5 .440914415 | -2 .6512 | 0 .01125 | 0 .083107875 |
| WWP1 | -0 .481690054 | 8 .450863089 | -2 .6505 | 0 .01127 | 0 .083202634 |
| RBM15 | -0 .37567402 | 6 .919256943 | -2 .6491 | 0 .0113 | 0 .083419424 |
| TAF1D | -0 .416551043 | 6 . 185513899 | -2 .6488 | 0 .01131 | 0 .083419424 |
| CDC20 | 0 .918767446 | 6 .077772283 | 2 .64878 | 0 .01131 | 0 .083419424 |
| SLC27A6 | -0 .560558389 | 4 .681805997 | -2 .6476 | 0 .01135 | 0 .083517615 |
| ZBTB18 | -0 .403148591 | 6 .597554568 | -2 .6476 | 0 .01135 | 0 .083517615 |
| NCF4 | 0 .4526241 | 7 .563212248 | 2 .64735 | 0 .01136 | 0 .083517615 |
| RAB11 FIP1 | 0 .484781392 | 7 . 140023864 | 2 .64723 | 0 .01136 | 0 .083517615 |
| VPS33A | 0 .586451262 | 7 .213710986 | 2 .64703 | 0 .01137 | 0 .083517615 |
| RPL32 | -0 .297608185 | 12 .42359721 | -2 .6469 | 0 .01137 | 0 .083517615 |
| RNF43 | -0 .453590788 | 6 .617243868 | -2 .6463 | 0 .01139 | 0 .083605403 |
| MRPS22 | -0 .38535013 | 7 .798431132 | -2 .6457 | 0 .0114 | 0 .083675469 |
| PFDN4 | -0 .386366434 | 7 .506553174 | -2 .6438 | 0 .01146 | 0 .084033515 |
| PTPN22 | 0 .583325594 | 6 .031091373 | 2 .64062 | 0 .01155 | 0 .084568487 |
| STXBP1 | 0 .751337002 | 9 . 17218949 | 2 .64058 | 0 .01155 | 0 .084568487 |
| TMEM176B | 0 .608353396 | 8 .392241316 | 2 .64058 | 0 .01155 | 0 .084568487 |
| OSBP | -0 .275057109 | 8 .477532131 | -2 .6394 | 0 .01159 | 0 .084748285 |
| TAF7L | 0 .731741308 | 4 .861608989 | 2 .63915 | 0 .01159 | 0 .084748285 |
| FOXRED2 | 0 .445196802 | 6 .286455411 | 2 .63905 | 0 .0116 | 0 .084748285 |
| TNFRSF11B | -0 .472693773 | 6 .48243289 | -2 .6368 | 0 .01166 | 0 .085037915 |
| HECTD4 | -0 .3591591 | 6 .470319093 | -2 .6366 | 0 .01167 | 0 .085037915 |
| OARD1 | -0 .35118006 | 6 .472128242 | -2 .6365 | 0 .01167 | 0 .085037915 |
| NCOA3 | -0 .291104269 | 9 .343916944 | -2 .6364 | 0 .01167 | 0 .085037915 |
| MOB3B | -0 .30010656 | 7 .228650093 | -2 .6363 | 0 .01168 | 0 .085037915 |
| PRKAG1 | -0 .300517969 | 8 .253593946 | -2 .6359 | 0 .01169 | 0 .085037915 |
| SEC13 | 0 .375146473 | 9 . 173274354 | 2 .63576 | 0 .01169 | 0 .085037915 |
| TWF2 | 0 .292598527 | 7 .344262978 | 2 .63574 | 0 .01169 | 0 .085037915 |
| PLEK | 0 .581657067 | 8 .385521133 | 2 .6355 | 0 .0117 | 0 .085037915 |
| RGS10 | 0 .4811 18391 | 8 .610894242 | 2 .63538 | 0 .0117 | 0 .085037915 |
| ISG20L2 | 0 .251504413 | 8 .692830191 | 2 .63516 | 0 .01171 | 0 .085037915 |
| KRT31 | -0 .893463441 | 6 .952651805 | -2 .6347 | 0 .01172 | 0 .085080913 |
| RPS6KB2 | 0 .449164255 | 7 .410217467 | 2 .63415 | 0 .01174 | 0 .085148658 |
| OMD | -0 .496085691 | 6 .071537428 | -2 .6338 | 0 .01175 | 0 .085148658 |
| CASKIN2 | -0 .470368078 | 6 .937972013 | -2 .6337 | 0 .01175 | 0 .085148658 |
| MRS2 | 0 .375105115 | 6 .782217153 | 2 .63328 | 0 .01177 | 0 .085196244 |

| SP140L | 0 .638949446 | 5 .909754389 | 2 .63249 | 0 .01179 | 0 .085316184 |
| --- | --- | --- | --- | --- | --- |
| CXCL13 | 1 .423880322 | 5 .701872704 | 2 .63224 | 0 .0118 | 0 .0853212 |
| CHEK1 | 0 .28243448 | 6 .717369362 | 2 .63156 | 0 .01182 | 0 .085408008 |
| CDH22 | -0 .545079628 | 4 .950361022 | -2 .6314 | 0 .01182 | 0 .085408008 |
| RALGAPA1 | -0 .558387664 | 6 .891455788 | -2 .6304 | 0 .01185 | 0 .08557471 |
| CXorf40A | 0 .277615701 | 7 .885443642 | 2 .62982 | 0 .01187 | 0 .085646477 |
| BTN3A2 | 0 .649248734 | 8 .383716907 | 2 .62939 | 0 .01188 | 0 .085688383 |
| ATN1 | -0 .364294038 | 8 .70418792 | -2 .6279 | 0 .01193 | 0 .085959302 |
| BCAT2 | -0 .595842355 | 7 .621484037 | -2 .6268 | 0 .01196 | 0 .086131446 |
| SAE1 | 0 .371526641 | 8 .755916921 | 2 .62666 | 0 .01196 | 0 .086131446 |
| UQCRQ | -0 .515816191 | 9 .21037223 | -2 .6253 | 0 .01201 | 0 .086376816 |
| SLC22A5 | -0 .436178417 | 6 . 190433271 | -2 .6247 | 0 .01202 | 0 .08646927 |
| MCAT | 0 .406707059 | 7 .744807053 | 2 .62353 | 0 .01206 | 0 .086664455 |
| UCHL5 | 0 .25648214 | 7 .695219709 | 2 .62252 | 0 .01209 | 0 .086765588 |
| AARS | 0 .302103322 | 9 .400619265 | 2 .62228 | 0 .0121 | 0 .086765588 |
| ATG2A | 0 .516822874 | 8 .091330295 | 2 .62216 | 0 .0121 | 0 .086765588 |
| CLN6 | 0 .409224323 | 6 .505831875 | 2 .62215 | 0 .0121 | 0 .086765588 |
| CNN2 | 0 .397380059 | 8 .693746918 | 2 .6211 1 | 0 .01213 | 0 .086943507 |
| ATP6V1G1 | -0 .449405038 | 9 .554192705 | -2 .62 | 0 .01217 | 0 .087096455 |
| ADIPOQ | -0 .757689078 | 6 .913107465 | -2 .62 | 0 .01217 | 0 .087096455 |
| GHITM | -0 .349946795 | 10 . 19921804 | -2 .6195 | 0 .01218 | 0 .087130063 |
| MAPK1 | 0 .308928672 | 8 .830053685 | 2 .61911 | 0 .01219 | 0 .087130063 |
| CD97 | 0 .364136191 | 8 .20983421 | 2 .61891 | 0 .0122 | 0 .087130063 |
| IRF6 | -0 .689327321 | 8 .561363546 | -2 .6189 | 0 .0122 | 0 .087130063 |
| B3GNT1 | 0 .578068456 | 8 .005588538 | 2 .61867 | 0 .01221 | 0 .087130063 |
| PAM | -0 .458501823 | 10 .09878538 | -2 .6184 | 0 .01222 | 0 .087150029 |
| ZAK | 0 .433866835 | 5 .578224803 | 2 .61764 | 0 .01224 | 0 .087256518 |
| EFNA4 | -0 .625326699 | 5 .853536034 | -2 .6169 | 0 .01226 | 0 .087364528 |
| KLK13 | 0 .720818146 | 7 .612249713 | 2 .61663 | 0 .01227 | 0 .087379824 |
| GPC3 | -0 .493127928 | 5 .816972833 | -2 .6149 | 0 .01232 | 0 .087674394 |
| CPE | -0 .800149593 | 8 . 1 18638908 | -2 .6148 | 0 .01233 | 0 .087674394 |
| ROBO3 | 0 .72552385 | 4 .070180055 | 2 .61271 | 0 .01239 | 0 .088044647 |
| OR7E156P | 0 .74578591 | 7 .221417608 | 2 .61271 | 0 .01239 | 0 .088044647 |
| APOBEC3B | 0 .555888813 | 6 .557467695 | 2 .61246 | 0 .0124 | 0 .088048785 |
| PTX3 | 0 .599234921 | 4 .696846139 | 2 .61201 | 0 .01241 | 0 .088099128 |
| LAIR1 | 0 .333800214 | 8 .273329795 | 2 .61129 | 0 .01244 | 0 .088205816 |
| FOXM1 | 0 .49752398 | 6 .966689753 | 2 .61108 | 0 .01244 | 0 .088205816 |
| CUL2 | -0 .351000617 | 6 .528655914 | -2 .6106 | 0 .01246 | 0 .088217706 |
| DCXR | -0 .488452782 | 8 .480523664 | -2 .6106 | 0 .01246 | 0 .088217706 |
| APPBP2 | -0 .235386527 | 8 .761508261 | -2 .6099 | 0 .01248 | 0 .088279343 |
| IGHM | 0 .996053691 | 8 .944165922 | 2 .60985 | 0 .01248 | 0 .088279343 |
| GMIP | 0 .556276775 | 7 . 162634662 | 2 .60926 | 0 .0125 | 0 .088326892 |
| PFKP | 0 .402795853 | 8 .496621649 | 2 .60903 | 0 .01251 | 0 .088326892 |
| PRDM2 | -0 .243734088 | 8 .429621049 | -2 .609 | 0 .01251 | 0 .088326892 |
| ENDOD1 | -0 .387603434 | 9 . 183477385 | -2 .6072 | 0 .01256 | 0 .088671264 |
| NUCB1 | 0 .288284984 | 9 .920133382 | 2 .60666 | 0 .01258 | 0 .088738052 |
| SSFA2 | -0 .496168264 | 8 .555983074 | -2 .605 | 0 .01263 | 0 .089065927 |
| GTF3C3 | -0 .377568938 | 6 .837161019 | -2 .6046 | 0 .01265 | 0 .089091701 |
| DGCR2 | -0 .353769482 | 9 .268292994 | -2 .6039 | 0 .01267 | 0 .089211 199 |
| KCNV1 | 0 .898117276 | 5 .431333471 | 2 .60342 | 0 .01268 | 0 .089213275 |
| C6orf47 | 0 .279262893 | 8 .390394242 | 2 .60341 | 0 .01268 | 0 .089213275 |
| SPAG16 | -0 .729582919 | 5 .217840615 | -2 .6025 | 0 .01271 | 0 .089360161 |
| CST3 | 0 .383489417 | 12 .26452793 | 2 .60144 | 0 .01275 | 0 .089554153 |
| TSKU | -0 .407911993 | 8 .214457922 | -2 .601 | 0 .01276 | 0 .089597167 |
| CLDN1 | -0 .617498494 | 6 .623435685 | -2 .6006 | 0 .01277 | 0 .089597167 |
| BATF | 0 .745978997 | 6 .513093335 | 2 .60057 | 0 .01277 | 0 .089597167 |

| IFI6 | 0 .94580848 | 9 .35957234 | 2 .60021 | 0 .01279 | 0 .089628715 |
| --- | --- | --- | --- | --- | --- |
| ARPP19 | -0 .295708965 | 9 .845540854 | -2 .599 | 0 .01282 | 0 .089795387 |
| PRKAA1 | 0 .408823051 | 5 .33861155 | 2 .59898 | 0 .01283 | 0 .089795387 |
| BLM | 0 .332258363 | 6 .410723321 | 2 .5988 | 0 .01283 | 0 .089795387 |
| APEX1 | -0 .287320829 | 9 .625915047 | -2 .5984 | 0 .01284 | 0 .089811394 |
| NQO1 | 0 .5672411 1 1 | 9 .221132774 | 2 .59828 | 0 .01285 | 0 .089811394 |
| COCH | -0 .816815437 | 5 .474482995 | -2 .5978 | 0 .01286 | 0 .089862348 |
| CALML5 | 1 .480996795 | 8 .833118114 | 2 .59499 | 0 .01295 | 0 .090453999 |
| EIF2B2 | -0 .344995946 | 8 .227912037 | -2 .5944 | 0 .01297 | 0 .090498008 |
| IL18 | -0 .612365916 | 6 . 107903597 | -2 .5943 | 0 .01297 | 0 .090498008 |
| SH2B3 | 0 .449779177 | 7 .721301945 | 2 .59321 | 0 .01301 | 0 .090704832 |
| AFTPH | -0 .256889377 | 8 .857899898 | -2 .5925 | 0 .01303 | 0 .090809317 |
| AHSA2 | -0 .63564795 | 5 .079746585 | -2 .5923 | 0 .01304 | 0 .090819963 |
| CACNA2D2 | -0 .330160834 | 6 .230521403 | -2 .5919 | 0 .01306 | 0 .090862295 |
| PGRMC1 | -0 .561911 174 | 9 .875727923 | -2 .5915 | 0 .01307 | 0 .090898641 |
| STAT5A | -0 .342392552 | 7 .724057327 | -2 .5912 | 0 .01308 | 0 .09090009 |
| RASGRP2 | 0 .29338738 | 7 .791473458 | 2 .58993 | 0 .01312 | 0 .091147564 |
| FBXW4 | -0 .388348157 | 7 .639296748 | -2 .5887 | 0 .01316 | 0 .091336255 |
| SAGE1 | 0 .694096838 | 4 .689378731 | 2 .58846 | 0 .01317 | 0 .091336255 |
| CD2BP2 | 0 .27043667 | 8 .223523092 | 2 .58844 | 0 .01317 | 0 .091336255 |
| BANP | 0 .268356052 | 7 .657705085 | 2 .58753 | 0 .0132 | 0 .091493555 |
| ARMC8 | -0 .307545713 | 7 .781719455 | -2 .5869 | 0 .01322 | 0 .091579653 |
| MKRN1 | -0 .279902804 | 9 .09627239 | -2 .5853 | 0 .01327 | 0 .091895001 |
| GMFB | -0 .445013273 | 8 .373132597 | -2 .5851 | 0 .01328 | 0 .091895001 |
| USP36 | 0 .510515433 | 5 .458702725 | 2 .58409 | 0 .01331 | 0 .091998785 |
| IL20RA | -0 .499555716 | 5 .099493197 | -2 .584 | 0 .01331 | 0 .091998785 |
| PLAC8 | 0 .672202116 | 6 . 16030335 | 2 .58398 | 0 .01332 | 0 .091998785 |
| CLK4 | -0 .558625346 | 6 .559947585 | -2 .5838 | 0 .01332 | 0 .091998785 |
| COL17A1 | -1 . 195142448 | 7 .420414383 | -2 .5835 | 0 .01333 | 0 .092022622 |
| RNFT1 | 0 .689463295 | 3 .204305712 | 2 .58195 | 0 .01338 | 0 .092318454 |
| RPL29 | -0 .237736298 | 12 .81680177 | -2 .5813 | 0 .0134 | 0 .092414774 |
| FERMT1 | -0 .807674441 | 7 .855757005 | -2 .5807 | 0 .01342 | 0 .092500954 |
| CDS1 | -0 .434899661 | 6 .302783611 | -2 .5797 | 0 .01346 | 0 .09267494 |
| ZNF747 | -0 .436048413 | 7 .945807587 | -2 .5795 | 0 .01347 | 0 .09268418 |
| MAL | -0 .675211667 | 9 .212537918 | -2 .5792 | 0 .01348 | 0 .09268662 |
| DLEU2 | -0 .413977294 | 6 .202811415 | -2 .579 | 0 .01348 | 0 .09268662 |
| TNNT1 | -1 .072265341 | 5 .093975324 | -2 .5783 | 0 .01351 | 0 .092802854 |
| ATF2 | -0 .229932987 | 8 .221814258 | -2 .5781 | 0 .01351 | 0 .092802854 |
| GID8 | 0 .276984185 | 8 .050739962 | 2 .57714 | 0 .01354 | 0 .092961022 |
| IGFBP3 | -0 .484779455 | 9 .762378635 | -2 .577 | 0 .01355 | 0 .092961022 |
| TBXAS1 | 0 .711739337 | 6 . 164227842 | 2 .57587 | 0 .01359 | 0 .093163642 |
| UBXN7 | -0 .37337885 | 7 .613979649 | -2 .5756 | 0 .0136 | 0 .093171727 |
| CHAC1 | 0 .776249741 | 5 .53870779 | 2 .57529 | 0 .01361 | 0 .093196176 |
| SMARCC2 | -0 .356585598 | 8 .753051549 | -2 .5743 | 0 .01364 | 0 .093373506 |
| NOL10 | 0 .474557737 | 4 .393622923 | 2 .5739 | 0 .01365 | 0 .093416642 |
| FHOD1 | 0 .300667425 | 7 . 193728655 | 2 .5733 | 0 .01367 | 0 .093505812 |
| ZMAT3 | -0 .622361475 | 8 . 131477506 | -2 .5716 | 0 .01373 | 0 .093856752 |
| COIL | -0 .316970957 | 7 .93373809 | -2 .5712 | 0 .01375 | 0 .093889453 |
| SLC25A15 | 0 .360246585 | 6 .732277983 | 2 .57009 | 0 .01378 | 0 .094103211 |
| SMARCD1 | 0 .265876263 | 8 .713154015 | 2 .56967 | 0 .0138 | 0 .094149411 |
| NRF1 | -0 .272601213 | 7 .984677074 | -2 .5694 | 0 .01381 | 0 .094163551 |
| CCDC101 | -0 .268945139 | 7 .976192127 | -2 .5692 | 0 .01382 | 0 .094163551 |
| CFDP1 | -0 .373104507 | 7 .967849014 | -2 .5689 | 0 .01382 | 0 .094175041 |
| PSEN2 | 0 .553113597 | 8 .731290203 | 2 .56849 | 0 .01384 | 0 .094182736 |
| TIPARP | -0 .395607034 | 8 .029446703 | -2 .5681 | 0 .01385 | 0 .094182736 |
| MEIS1 | -0 .33107452 | 6 .538574614 | -2 .568 | 0 .01386 | 0 .094182736 |

| CCR7 | 1 .347873292 | 6 .554152645 | 2 .568 | 0 .01386 | 0 .094182736 |
| --- | --- | --- | --- | --- | --- |
| KANK1 | -0 .575519711 | 9 . 165319106 | -2 .5663 | 0 .01392 | 0 .094538694 |
| C19orf10 | 0 .318535531 | 9 .282633972 | 2 .56509 | 0 .01396 | 0 .094764998 |
| CD74 | 0 .590842687 | 12 .47213912 | 2 .56399 | 0 .01399 | 0 .094972711 |
| ACTG2 | -0 .963185259 | 7 .328392555 | -2 .5635 | 0 .01401 | 0 .095028928 |
| IPO7 | -0 .398738057 | 9 .999542522 | -2 .5629 | 0 .01403 | 0 .095129562 |
| ELP5 | 0 .460996269 | 6 .889368685 | 2 .56266 | 0 .01404 | 0 .09513251 |
| SERTAD2 | -0 .314980186 | 9 .019820312 | -2 .5612 | 0 .01409 | 0 .095420201 |
| CHMP2A | 0 .376219153 | 8 .999823478 | 2 .56015 | 0 .01413 | 0 .095624297 |
| YTHDF3 | -0 .469278206 | 7 .782882883 | -2 .5594 | 0 .01415 | 0 .09570478 |
| RHOG | 0 .488576845 | 9 .581937283 | 2 .55937 | 0 .01416 | 0 .09570478 |
| RAB35 | 0 .261977358 | 8 .61503368 | 2 .55671 | 0 .01425 | 0 .096288477 |
| C16orf58 | 0 .284454969 | 9 . 19058117 | 2 .55603 | 0 .01427 | 0 .096398403 |
| FUS | 0 .323118109 | 9 .836176594 | 2 .55576 | 0 .01428 | 0 .096405255 |
| HYOU1 | 0 .434638786 | 9 .018284869 | 2 .55551 | 0 .01429 | 0 .096405255 |
| POU3F1 | -0 .83878249 | 8 . 148460305 | -2 .5552 | 0 .0143 | 0 .096405255 |
| LYRM1 | -0 .330748013 | 6 .586732962 | -2 .5551 | 0 .01431 | 0 .096405255 |
| CRYBG3 | -0 .41781868 | 7 .546150249 | -2 .5548 | 0 .01432 | 0 .096429856 |
| TMEM30B | -0 .444393528 | 5 .909807337 | -2 .5535 | 0 .01436 | 0 .096655635 |
| HP1BP3 | 0 .768151362 | 4 .320773922 | 2 .55334 | 0 .01437 | 0 .096655635 |
| KIF4A | 0 .686941032 | 6 .019388764 | 2 .55322 | 0 .01437 | 0 .096655635 |
| HSP90B1 | 0 .359131664 | 8 .564047402 | 2 .55258 | 0 .0144 | 0 .096688554 |
| RTFDC1 | 0 .35023556 | 9 . 130056053 | 2 .55245 | 0 .0144 | 0 .096688554 |
| DNASE1L1 | 0 .214147516 | 7 .439593253 | 2 .55244 | 0 .0144 | 0 .096688554 |
| CERS4 | -0 .53020414 | 6 .339583827 | -2 .5518 | 0 .01442 | 0 .096781647 |
| PARL | -0 .334424865 | 8 .031545948 | -2 .5516 | 0 .01443 | 0 .096796996 |
| TICAM1 | 0 .393237919 | 6 .464456768 | 2 .54965 | 0 .0145 | 0 .097197996 |
| KRT18 | -0 .63499723 | 7 .025087327 | -2 .5493 | 0 .01452 | 0 .097197996 |
| SF3A2 | -0 .396758725 | 9 .605652363 | -2 .5492 | 0 .01452 | 0 .097197996 |
| HCN4 | -0 .463477425 | 4 .82158743 | -2 .5474 | 0 .01458 | 0 .09758463 |
| PPIF | 0 .606254291 | 8 .35613395 | 2 .54673 | 0 .01461 | 0 .097698717 |
| COL6A1 | 0 .30981616 | 1 1 .93016641 | 2 .54601 | 0 .01463 | 0 .097820001 |
| MRFAP1L1 | -0 .349749341 | 7 .950403814 | -2 .5451 | 0 .01466 | 0 .09797817 |
| SNX10 | 0 .969437284 | 8 .475347399 | 2 .54472 | 0 .01468 | 0 .098028842 |
| COL14A1 | -0 .420461412 | 7 .581845865 | -2 .5438 | 0 .01471 | 0 .098207867 |
| HSD11B1 | -0 .748240477 | 6 .585437933 | -2 .5424 | 0 .01476 | 0 .098456254 |
| MSI1 | -0 .855686039 | 3 .38297811 1 | -2 .5423 | 0 .01477 | 0 .098456254 |
| SLAMF8 | 0 .678140288 | 8 . 125365774 | 2 .54202 | 0 .01478 | 0 .098476467 |
| TM4SF1 | -0 .545961209 | 9 .828681072 | -2 .5411 | 0 .01481 | 0 .098626833 |
| NEDD4 | -0 .31356167 | 5 .864941387 | -2 .5409 | 0 .01482 | 0 .098626833 |
| DTYMK | 0 .488953221 | 6 .646414903 | 2 .54076 | 0 .01482 | 0 .098626833 |
| SYNE2 | -0 .525108767 | 7 .916430742 | -2 .5379 | 0 .01493 | 0 .099262831 |
| CHN2 | -0 .474405054 | 8 . 142324766 | -2 .5375 | 0 .01495 | 0 .099288933 |
| TCEB3 | 0 .257821074 | 10 .06474963 | 2 .53741 | 0 .01495 | 0 .099288933 |
| NTAN1 | -0 .44037498 | 8 .854445417 | -2 .537 | 0 .01496 | 0 .099342898 |
| LIPG | 0 .452989966 | 5 .679408249 | 2 .53629 | 0 .01499 | 0 .09945806 |
| CTSV | 0 .723160335 | 7 .615004735 | 2 .53582 | 0 .01501 | 0 .099520105 |
| CD164 | -0 .391133463 | 9 .992284635 | -2 .5354 | 0 .01502 | 0 .099566643 |
| SPATA2 | 0 .337515951 | 7 .596157314 | 2 .53502 | 0 .01504 | 0 .099613783 |
| ERMAP | -0 .533890653 | 5 .774358982 | -2 .5339 | 0 .01508 | 0 .099728122 |
| DLGAP4 | 0 .228049673 | 8 .949236506 | 2 .53373 | 0 .01508 | 0 .099728122 |
| RCN3 | 0 .332461808 | 8 .053046785 | 2 .53371 | 0 .01508 | 0 .099728122 |
| SEC11A | -0 .427473523 | 10 .48037115 | -2 .5337 | 0 .01508 | 0 .099728122 |
| GTF2H3 | -0 .729432077 | 7 .621712119 | -2 .533 | 0 .01511 | 0 .09985911 |
| EIF5A | 0 .481755201 | 10 .64197115 | 2 .53185 | 0 .01515 | 0 . 10002998 |
| GPATCH2 | 0 .351932282 | 6 .536925776 | 2 .53183 | 0 .01515 | 0 . 10002998 |

| PCF11 | -0 .398514497 | 5 .6985511 13 | -2 .5312 | 0 .01518 | 0 . 100125724 |
| --- | --- | --- | --- | --- | --- |
| OR7E37P | 0 .460811 133 | 7 .635011668 | 2 .53102 | 0 .01518 | 0 . 100125724 |
| TMSB10 | 0 .403955648 | 1 1 .76419827 | 2 .52924 | 0 .01525 | 0 . 100513927 |
| C4orf19 | -0 .308587907 | 6 .989663957 | -2 .5287 | 0 .01527 | 0 . 100521134 |
| WDR48 | -0 .244043136 | 9 . 167851056 | -2 .5286 | 0 .01527 | 0 . 100521134 |
| HOXC6 | -0 .537367064 | 8 .539573362 | -2 .5286 | 0 .01528 | 0 . 100521134 |
| ITPR3 | 0 .334302992 | 9 . 126751432 | 2 .52689 | 0 .01534 | 0 . 100884857 |
| CENPU | 0 .572431708 | 6 .813700556 | 2 .52627 | 0 .01536 | 0 . 100987123 |
| GRB7 | -0 .538646314 | 6 .247910236 | -2 .5254 | 0 .0154 | 0 . 101145974 |
| TMEM50B | -0 .354615543 | 6 .391226288 | -2 .5249 | 0 .01542 | 0 . 101178867 |
| NOX4 | 0 .558701619 | 5 .579925049 | 2 .52485 | 0 .01542 | 0 . 101178867 |
| PHIP | -0 .325035037 | 8 .485568611 | -2 .5246 | 0 .01543 | 0 . 101178867 |
| ITGAL | 1 .028547562 | 5 .842462669 | 2 .52426 | 0 .01544 | 0 . 10122198 |
| DTX4 | -0 .287300706 | 8 .753638154 | -2 .5236 | 0 .01547 | 0 . 101340127 |
| ETNK1 | 0 .446329583 | 6 .976315677 | 2 .5223 | 0 .01551 | 0 . 101606143 |
| FAM57A | -0 .489937106 | 8 .37422212 | -2 .5198 | 0 .01561 | 0 . 102171733 |
| MUC1 | -0 .696391721 | 7 .884335485 | -2 .5194 | 0 .01563 | 0 . 10223686 |
| SSTR3 | -0 .407143027 | 2 .393545487 | -2 .5191 | 0 .01564 | 0 . 102239407 |
| STRN3 | -0 .312370343 | 6 .654318661 | -2 .5185 | 0 .01566 | 0 . 102295817 |
| CCND2 | -0 .647308221 | 9 .049458024 | -2 .5182 | 0 .01567 | 0 . 102295817 |
| ARHGAP1 | 0 .286430738 | 10 .54553275 | 2 .51812 | 0 .01567 | 0 . 102295817 |
| EMP3 | 0 .442091437 | 10 .9081433 | 2 .51807 | 0 .01568 | 0 . 102295817 |
| TUBB7P | 0 .845951953 | 3 .415896756 | 2 .51736 | 0 .0157 | 0 . 102344138 |
| LCMT1 | 0 .267808098 | 8 .235377936 | 2 .51736 | 0 .0157 | 0 . 102344138 |
| HIST3H3 | -0 .841131262 | 3 .260944137 | -2 .5172 | 0 .01571 | 0 . 102344138 |
| HLA-DRB4 | 1 .05431279 | 7 .473304724 | 2 .51678 | 0 .01573 | 0 . 102386127 |
| ANKS1B | 0 .583285034 | 3 .837257393 | 2 .51666 | 0 .01573 | 0 . 102386127 |
| C11orf24 | 0 .535114239 | 9 .783246846 | 2 .5161 | 0 .01575 | 0 . 102428792 |
| CHAF1A | 0 .323607492 | 8 .302733292 | 2 .51606 | 0 .01575 | 0 . 102428792 |
| ERMP1 | -0 .29768251 | 7 . 162608236 | -2 .5154 | 0 .01578 | 0 . 102510267 |
| UBA3 | -0 .415976701 | 7 .301996324 | -2 .5152 | 0 .01579 | 0 . 102510267 |
| YIPF6 | -0 .262849336 | 8 .762463225 | -2 .5151 | 0 .01579 | 0 . 102510267 |
| SH3GL1 | 0 .342913922 | 8 .628353282 | 2 .51485 | 0 .0158 | 0 . 102521531 |
| CMTR1 | 0 .303159769 | 8 .036985067 | 2 .51463 | 0 .01581 | 0 . 102524136 |
| NPTX1 | 0 .675018726 | 4 .766435891 | 2 .51377 | 0 .01584 | 0 . 102570569 |
| SCARA3 | -0 .472184648 | 6 .909069659 | -2 .5137 | 0 .01585 | 0 . 102570569 |
| RALY | 0 .298681961 | 8 .363188308 | 2 .51367 | 0 .01585 | 0 . 102570569 |
| ICAM3 | 0 .659416905 | 7 .344973847 | 2 .51361 | 0 .01585 | 0 . 102570569 |
| NRP2 | 0 .272982419 | 8 .286327794 | 2 .5121 | 0 .01591 | 0 . 102897431 |
| VPS13A | -0 .288020438 | 7 .752816283 | -2 .5113 | 0 .01594 | 0 . 103050699 |
| LRPPRC | -0 .395932779 | 9 .302479955 | -2 .5105 | 0 .01597 | 0 . 103107475 |
| TAF9B | -0 .33322252 | 7 . 101323052 | -2 .5105 | 0 .01597 | 0 . 103107475 |
| LOC647070 | 0 .64210693 | 4 .72452262 | 2 .51044 | 0 .01597 | 0 . 103107475 |
| COL5A2 | 0 .559165611 | 9 . 196187127 | 2 .50984 | 0 .016 | 0 . 103206505 |
| DNAJC22 | 0 .435625243 | 5 .851725919 | 2 .50879 | 0 .01604 | 0 . 1034056 |
| PEX7 | -0 .291057902 | 7 .281188642 | -2 .5086 | 0 .01604 | 0 . 1034056 |
| GTF3A | 0 .477143852 | 10 .49481719 | 2 .50796 | 0 .01607 | 0 . 103523049 |
| SOCS5 | -0 .387747885 | 7 .767990906 | -2 .5074 | 0 .01609 | 0 . 103616267 |
| FBXO2 | -0 .391592694 | 6 .427193041 | -2 .507 | 0 .01611 | 0 . 103667551 |
| GGCT | 0 .333951421 | 9 .088558977 | 2 .50656 | 0 .01613 | 0 . 103719208 |
| KIF2C | 0 .224680375 | 7 .856596901 | 2 .50437 | 0 .01621 | 0 . 104225005 |
| ATF1 | -0 .727029752 | 5 .600560367 | -2 .5037 | 0 .01624 | 0 . 104347161 |
| TAF6 | 0 .358494958 | 8 .237178747 | 2 .50303 | 0 .01627 | 0 . 104460528 |
| CD248 | -0 .406394283 | 8 .352470685 | -2 .501 | 0 .01635 | 0 . 104922757 |
| PLAG1 | -0 .717643669 | 5 .962396177 | -2 .4989 | 0 .01643 | 0 . 10542311 |
| BCKDHA | 0 .406300908 | 8 .212766772 | 2 .49767 | 0 .01648 | 0 . 105678879 |

| SPINT1 | -0 .648507555 | 7 .482599435 | -2 .4972 | 0 .0165 | 0 . 105742173 |
| --- | --- | --- | --- | --- | --- |
| NAGLU | 0 .303725417 | 7 .73614393 | 2 .49618 | 0 .01654 | 0 . 105957595 |
| TF | -0 .756470275 | 7 .524058193 | -2 .4948 | 0 .0166 | 0 . 106269813 |
| GMPR | 0 .60848678 | 9 .697779453 | 2 .49336 | 0 .01666 | 0 . 106582306 |
| ACOT8 | 0 .211527938 | 7 . 166884134 | 2 .49175 | 0 .01672 | 0 . 106786528 |
| ALOX5 | 0 .292732308 | 7 .551431793 | 2 .49163 | 0 .01673 | 0 . 106786528 |
| ATP12A | 0 .560151729 | 6 .61625787 | 2 .49131 | 0 .01674 | 0 . 106786528 |
| CXorf57 | -0 .504788471 | 4 .567258357 | -2 .4912 | 0 .01674 | 0 . 106786528 |
| CHMP2B | -0 .389352806 | 8 .869462778 | -2 .4912 | 0 .01674 | 0 . 106786528 |
| FKBP15 | 0 .212641075 | 8 .926143458 | 2 .49117 | 0 .01675 | 0 . 106786528 |
| HEATR6 | 0 .255069659 | 7 .854858824 | 2 .49109 | 0 .01675 | 0 . 106786528 |
| PPP1R13L | -0 .589050045 | 7 .47823887 | -2 .4908 | 0 .01676 | 0 . 106786528 |
| GLUL | -0 .397347109 | 10 .59461654 | -2 .4907 | 0 .01677 | 0 . 106786528 |
| STARD7 | -0 .339066352 | 9 .590283657 | -2 .4903 | 0 .01678 | 0 . 106819683 |
| MFSD12 | 0 .768483069 | 9 .691841357 | 2 .49017 | 0 .01679 | 0 . 106819683 |
| LDOC1 | -0 .858977851 | 7 .5313371 | -2 .4897 | 0 .01681 | 0 . 106825285 |
| CSF2RA | 0 .459680926 | 7 .265655596 | 2 .48952 | 0 .01681 | 0 . 106825285 |
| METTL18 | -0 .371628367 | 6 . 132414767 | -2 .4893 | 0 .01682 | 0 . 106825285 |
| IQSEC1 | -0 .309928901 | 8 .887534577 | -2 .4893 | 0 .01682 | 0 . 106825285 |
| NDST3 | 0 .540533966 | 5 .390853878 | 2 .48805 | 0 .01687 | 0 . 10709178 |
| NONO | -0 .320533362 | 1 1 .30326321 | -2 .4878 | 0 .01688 | 0 . 10709178 |
| EWSR1 | 0 .280844328 | 9 .494011406 | 2 .48767 | 0 .01689 | 0 . 10709178 |
| INTS7 | 0 .327032861 | 6 .882800798 | 2 .48708 | 0 .01691 | 0 . 107193439 |
| COX5A | 0 .345184844 | 9 .5558552 | 2 .48682 | 0 .01693 | 0 . 10720794 |
| PPARGC1A | -0 .472941936 | 5 .417714831 | -2 .4865 | 0 .01694 | 0 . 107245926 |
| IL13 | 0 .400594034 | 6 . 134662126 | 2 .4861 | 0 .01696 | 0 . 107251306 |
| CCDC144A | -0 .623273634 | 3 . 147426069 | -2 .4859 | 0 .01696 | 0 . 107251306 |
| SLC27A2 | -0 .549010678 | 6 .848433444 | -2 .4855 | 0 .01698 | 0 . 107251306 |
| SMAD4 | -0 .297400188 | 7 .072937951 | -2 .4853 | 0 .01699 | 0 . 107251306 |
| TMEM41B | -0 .268418426 | 8 .091888488 | -2 .4853 | 0 .01699 | 0 . 107251306 |
| C1GALT1C1 | -0 .46332668 | 7 .033935255 | -2 .4852 | 0 .01699 | 0 . 107251306 |
| SLC38A1 | -0 .79036632 | 7 .674058355 | -2 .485 | 0 .017 | 0 . 107251306 |
| ERN1 | 0 .399629045 | 7 .535661702 | 2 .48443 | 0 .01702 | 0 . 107338952 |
| DOHH | 0 .410477894 | 7 .315701049 | 2 .48426 | 0 .01703 | 0 . 107338952 |
| KIAA1324 | -0 .387908619 | 5 .718834225 | -2 .4836 | 0 .01706 | 0 . 107423555 |
| RAD50 | -0 .474610758 | 6 .956659664 | -2 .4835 | 0 .01706 | 0 . 107423555 |
| ADH5 | -0 .455420259 | 8 .857364204 | -2 .4813 | 0 .01715 | 0 . 10795218 |
| STAT1 | 0 .822169285 | 10 .05946166 | 2 .48085 | 0 .01717 | 0 . 108018386 |
| TMEM185B | 0 .324341205 | 6 .960518698 | 2 .48009 | 0 .01721 | 0 . 10813316 |
| SLIT3 | -0 .348721203 | 8 .558745406 | -2 .48 | 0 .01721 | 0 . 10813316 |
| ARG1 | 1 .010075858 | 7 .522718618 | 2 .4789 | 0 .01726 | 0 . 10837069 |
| HAO2 | -0 .653388867 | 6 .238834834 | -2 .478 | 0 .01729 | 0 . 108552769 |
| RP3-497J21 . 1 | -0 .802743383 | 4 . 175654972 | -2 .4775 | 0 .01732 | 0 . 1086394 |
| LINC00597 | -0 .781683022 | 3 .68091207 | -2 .4765 | 0 .01736 | 0 . 108848343 |
| AIDA | -0 .427296276 | 7 .488146856 | -2 .4761 | 0 .01737 | 0 . 10888599 |
| GNAS | 0 .302460534 | 14 .74635103 | 2 .47585 | 0 .01738 | 0 . 10888599 |
| KAT5 | -0 .228278412 | 9 .738251681 | -2 .4757 | 0 .01739 | 0 . 10888599 |
| TMEM187 | -0 .42626378 | 6 .068415191 | -2 .4754 | 0 .0174 | 0 . 108915473 |
| TPPP3 | -1 . 133670768 | 7 .328952741 | -2 .475 | 0 .01742 | 0 . 10892534 |
| C5orf45 | -0 .46062509 | 5 .883524512 | -2 .475 | 0 .01742 | 0 . 10892534 |
| NDUFB2-AS1 | 0 .524955169 | 4 .999596298 | 2 .47306 | 0 .0175 | 0 . 109365728 |
| BIN2 | 1 .253927382 | 4 .791793811 | 2 .47289 | 0 .01751 | 0 . 109365728 |
| HEY1 | 0 .669985491 | 8 .843831379 | 2 .47255 | 0 .01752 | 0 . 109402402 |
| GRK6 | 0 .267252243 | 9 .521566548 | 2 .47231 | 0 .01754 | 0 . 109413582 |
| CTC-338M12 .4 | 0 .398405831 | 6 .440006862 | 2 .47207 | 0 .01755 | 0 . 109413886 |
| MON1B | 0 .25770281 | 6 .46458154 | 2 .4719 | 0 .01755 | 0 . 109413886 |

| TFCP2L1 | -0 .373296474 | 6 .819297228 | -2 .4711 | 0 .01759 | 0 . 109539278 |
| --- | --- | --- | --- | --- | --- |
| PIM1 | 0 .526584628 | 7 .629529596 | 2 .47085 | 0 .0176 | 0 . 109539278 |
| DNAJC15 | 0 .606472077 | 7 .890872787 | 2 .47081 | 0 .0176 | 0 . 109539278 |
| KIF13A | -0 .559702242 | 5 . 158193826 | -2 .4696 | 0 .01765 | 0 . 109817105 |
| BAG3 | -0 .383928648 | 8 .66606251 | -2 .4687 | 0 .01769 | 0 . 109971945 |
| EPRS | 0 .263093872 | 9 .735479084 | 2 .46858 | 0 .01769 | 0 . 109971945 |
| ATXN7L3B | -0 .277476628 | 9 .605144209 | -2 .4666 | 0 .01778 | 0 . 1 10432132 |
| TTLL4 | 0 .34613224 | 7 .772972399 | 2 .46625 | 0 .0178 | 0 . 1 10432132 |
| NACAP1 | -0 .247091259 | 10 .74046702 | -2 .4661 | 0 .0178 | 0 . 1 10432132 |
| ING3 | -0 .341493773 | 6 .612037012 | -2 .4659 | 0 .01781 | 0 . 1 10432132 |
| INADL | -0 .498810727 | 4 .814814254 | -2 .4659 | 0 .01781 | 0 . 1 10432132 |
| PITPNC1 | 0 .28765621 | 7 .808380601 | 2 .4647 | 0 .01786 | 0 . 1 10685537 |
| PODXL2 | 0 .535693495 | 5 .059892245 | 2 .46425 | 0 .01788 | 0 . 1 10752795 |
| CLTC-IT1 | -0 .60051465 | 4 .569094148 | -2 .464 | 0 .01789 | 0 . 1 10761326 |
| INTS9 | 0 .27193236 | 7 .591427687 | 2 .4633 | 0 .01792 | 0 . 1 10901092 |
| RPP25 | 0 .477222929 | 6 .927688335 | 2 .46268 | 0 .01795 | 0 . 1 1 1013038 |
| TNIP1 | 0 .258277275 | 8 .927842335 | 2 .46203 | 0 .01798 | 0 . 1 1 1 132569 |
| ATP8A1 | -0 .643461855 | 5 .769114061 | -2 .4612 | 0 .01802 | 0 . 1 1 1315539 |
| TNFRSF8 | -0 .314740009 | 6 .614641238 | -2 .46 | 0 .01807 | 0 . 1 1 1571839 |
| LRRC15 | 0 .427442326 | 8 .035587637 | 2 .45976 | 0 .01808 | 0 . 1 1 1581562 |
| ZNF654 | -0 .414629052 | 6 .085823308 | -2 .4587 | 0 .01812 | 0 . 1 1 1811604 |
| RAI2 | -0 .421515343 | 6 .469709927 | -2 .4578 | 0 .01816 | 0 . 1 12005689 |
| ZBTB11 | -0 .411 142824 | 6 .366421811 | -2 .4559 | 0 .01825 | 0 . 1 12444227 |
| LHX2 | -0 .609106891 | 7 .088855552 | -2 .4558 | 0 .01825 | 0 . 1 12444227 |
| ADPRM | -0 .511577467 | 4 .823372496 | -2 .4554 | 0 .01827 | 0 . 1 12485201 |
| ASPN | -0 .650157951 | 6 .530444235 | -2 .4547 | 0 .0183 | 0 . 1 12623619 |
| HIST1H2BE | 0 .567792009 | 5 .976426105 | 2 .45213 | 0 .01842 | 0 . 1 13278101 |
| PADI2 | -0 .84469975 | 6 .834338992 | -2 .4517 | 0 .01844 | 0 . 1 13349713 |
| PNPLA4 | -0 .41573657 | 6 .877658746 | -2 .4503 | 0 .0185 | 0 . 1 13670556 |
| TPM1 | -0 .391654055 | 10 .09769426 | -2 .4501 | 0 .01851 | 0 . 1 13670556 |
| URGCP | 0 .237434709 | 7 .620196536 | 2 .44962 | 0 .01853 | 0 . 1 13670556 |
| MICAL1 | 0 .641148982 | 6 .9896346 | 2 .44961 | 0 .01853 | 0 . 1 13670556 |
| COL5A1 | 0 .40338701 | 9 .883787296 | 2 .44949 | 0 .01853 | 0 . 1 13670556 |
| ACOX2 | -0 .35639972 | 6 .977090507 | -2 .4488 | 0 .01856 | 0 . 1 13797825 |
| ARHGAP22 | 0 .479569565 | 5 .807958157 | 2 .44862 | 0 .01857 | 0 . 1 13797939 |
| ANO1 | -0 .683217943 | 5 .750772522 | -2 .4483 | 0 .01859 | 0 . 1 13818972 |
| ZCWPW1 | 0 .982299428 | 4 .981733159 | 2 .44793 | 0 .0186 | 0 . 1 13877603 |
| ZMIZ1 | -0 .388997567 | 8 .600960239 | -2 .4452 | 0 .01873 | 0 . 1 14582891 |
| WDR1 | 0 .269693692 | 10 .85051667 | 2 .44456 | 0 .01876 | 0 . 1 14697972 |
| MGMT | -0 .518084992 | 7 .503321754 | -2 .4432 | 0 .01882 | 0 . 1 15002565 |
| HIST1H1T | 0 .436045084 | 6 .820351572 | 2 .44306 | 0 .01882 | 0 . 1 15002565 |
| FAM8A1 | -0 .328747663 | 8 . 129356437 | -2 .4424 | 0 .01885 | 0 . 1 15107233 |
| SLC31A1 | 0 .674734048 | 6 .618871025 | 2 .44229 | 0 .01886 | 0 . 1 15107233 |
| BRCA1 | 0 .316080283 | 6 .552833749 | 2 .44177 | 0 .01888 | 0 . 1 15193772 |
| SEC14L1 | 0 .33537992 | 9 .030149104 | 2 .44145 | 0 .0189 | 0 . 1 15228816 |
| HNRNPH3 | -0 .417855442 | 9 .618858361 | -2 .4406 | 0 .01894 | 0 . 1 15396108 |
| YIF1A | 0 .49771986 | 8 .843552331 | 2 .44024 | 0 .01895 | 0 . 1 15452457 |
| CYTH3 | 0 .736853684 | 4 .900936721 | 2 .43936 | 0 .01899 | 0 . 1 15643697 |
| CDRT1 | 0 .70106703 | 6 .421501388 | 2 .43905 | 0 .01901 | 0 . 1 15672504 |
| SPRR2C | 0 .90622642 | 4 .993731581 | 2 .43789 | 0 .01906 | 0 . 1 15834896 |
| ABLIM1 | -0 .637134824 | 9 .777040696 | -2 .4379 | 0 .01906 | 0 . 1 15834896 |
| GNLY | 1 .058412063 | 7 .041399974 | 2 .43776 | 0 .01907 | 0 . 1 15834896 |
| PMEPA1 | 0 .505904415 | 6 .691539591 | 2 .4375 | 0 .01908 | 0 . 1 15834896 |
| MAGT1 | -0 .314575676 | 8 . 1 1 1341963 | -2 .4375 | 0 .01908 | 0 . 1 15834896 |
| GLS2 | -0 .453544679 | 4 .989967115 | -2 .4371 | 0 .0191 | 0 . 1 15881692 |
| CTNNAL1 | -0 .567168919 | 7 . 1 10854419 | -2 .4354 | 0 .01918 | 0 . 1 16311286 |

| OR2W1 | 0 .420625566 | 5 .314704843 | 2 .43479 | 0 .01921 | 0 . 1 16419321 |
| --- | --- | --- | --- | --- | --- |
| CYP4F12 | -0 .426864292 | 7 .252212486 | -2 .4338 | 0 .01925 | 0 . 1 16633828 |
| CCBL2 | -0 .385527514 | 7 .83819186 | -2 .4328 | 0 .0193 | 0 . 1 168151 |
| P2RX1 | -0 .36987032 | 7 . 15149736 | -2 .4328 | 0 .0193 | 0 . 1 168151 |
| JAM3 | -0 .430045718 | 8 .38294283 | -2 .4315 | 0 .01936 | 0 . 1 17099797 |
| DNAL4 | -0 .459201327 | 6 .094786721 | -2 .4314 | 0 .01936 | 0 . 1 17099797 |
| THOC1 | -0 .466194634 | 7 .489472584 | -2 .4308 | 0 .01939 | 0 . 1 17145048 |
| GPSM3 | 0 .479581677 | 8 .882873823 | 2 .4307 | 0 .0194 | 0 . 1 17145048 |
| SUGCT | 0 .507852566 | 4 .615453118 | 2 .43046 | 0 .01941 | 0 . 1 17145048 |
| POLR2J | 0 .275543431 | 9 . 128138187 | 2 .43038 | 0 .01941 | 0 . 1 17145048 |
| ZNF557 | 0 .361269261 | 4 .883001414 | 2 .43022 | 0 .01942 | 0 . 1 17145048 |
| NOC3L | -0 .461220399 | 6 .47788993 | -2 .4295 | 0 .01945 | 0 . 1 17282738 |
| ELL3 | -0 .273993109 | 7 .88736309 | -2 .4291 | 0 .01947 | 0 . 1 17346234 |
| PHGDH | -0 .454130577 | 9 . 1 13043487 | -2 .4277 | 0 .01954 | 0 . 1 17632315 |
| PSMA2 | -0 .3775514 | 9 . 131761569 | -2 .4276 | 0 .01954 | 0 . 1 17632315 |
| CCL1 | -0 .615882306 | 2 .871770828 | -2 .4274 | 0 .01955 | 0 . 1 17632315 |
| HLA-DMA | 0 .725110869 | 8 .973414811 | 2 .4273 | 0 .01956 | 0 . 1 17632315 |
| KIF14 | 0 .462272655 | 5 .448591049 | 2 .42619 | 0 .01961 | 0 . 1 17889526 |
| LAMA5 | -0 .421144281 | 8 .585935727 | -2 .4249 | 0 .01967 | 0 . 1 18183768 |
| S100A2 | 0 .949873552 | 1 1 .04983882 | 2 .42476 | 0 .01968 | 0 . 1 18183768 |
| MSC | 0 .667028963 | 6 .953932569 | 2 .42319 | 0 .01975 | 0 . 1 18574859 |
| DMXL2 | 0 .414690109 | 7 .36942714 | 2 .4227 | 0 .01977 | 0 . 1 18657132 |
| KIRREL | 0 .731193756 | 5 .458622615 | 2 .42195 | 0 .01981 | 0 . 1 1870647 |
| FAM50B | -0 .273696248 | 6 .836074947 | -2 .4219 | 0 .01981 | 0 . 1 1870647 |
| PCTP | -0 .311944501 | 6 .891944028 | -2 .4219 | 0 .01981 | 0 . 1 1870647 |
| CYR61 | 0 .667415537 | 8 . 151074724 | 2 .42167 | 0 .01982 | 0 . 1 18724907 |
| CAPZB | 0 .284556146 | 1 1 .48581022 | 2 .42102 | 0 .01985 | 0 . 1 18856375 |
| ATP1A2 | -0 .394301814 | 6 .077569154 | -2 .4208 | 0 .01986 | 0 . 1 18867184 |
| UBL5 | 0 .467211023 | 7 .920702118 | 2 .4204 | 0 .01988 | 0 . 1 18887047 |
| COL9A1 | -1 .240987679 | 4 .221043813 | -2 .4201 | 0 .0199 | 0 . 1 18887047 |
| ZBED4 | 0 .331076015 | 6 .646938149 | 2 .42012 | 0 .0199 | 0 . 1 18887047 |
| EPHB6 | -0 .858010325 | 8 . 129091829 | -2 .4184 | 0 .01998 | 0 . 1 19327786 |
| EIF2S3 | -0 .321241377 | 6 .984407417 | -2 .4157 | 0 .02011 | 0 . 120051151 |
| CASP3 | 0 .490882887 | 6 .292782751 | 2 .41534 | 0 .02013 | 0 . 120089707 |
| ZNF451 | -0 .373497632 | 7 .646884578 | -2 .4151 | 0 .02014 | 0 . 120102985 |
| PSMD2 | 0 .35953629 | 9 . 136117823 | 2 .4149 | 0 .02015 | 0 . 120102985 |
| DOK1 | 0 .299497325 | 7 .420012731 | 2 .4132 | 0 .02023 | 0 . 120535047 |
| DDX42 | -0 .29261284 | 8 .60484979 | -2 .4123 | 0 .02028 | 0 . 120752103 |
| SETD6 | -0 .607648173 | 5 .898655229 | -2 .4119 | 0 .02029 | 0 . 120791178 |
| HIST1H1D | -0 .490570157 | 5 .657504442 | -2 .4112 | 0 .02033 | 0 . 120941183 |
| COL6A2 | 0 .441156745 | 1 1 . 15385556 | 2 .41065 | 0 .02035 | 0 . 12104602 |
| CD79A | 1 . 139856888 | 6 .699062393 | 2 .40911 | 0 .02043 | 0 . 121436692 |
| LSM12 | 0 .315124517 | 9 .076209839 | 2 .40802 | 0 .02048 | 0 . 121698112 |
| CNN3 | -0 .679334317 | 7 .092629217 | -2 .4076 | 0 .02051 | 0 . 121777326 |
| NAV2 | 0 .544202211 | 7 .650495605 | 2 .40721 | 0 .02052 | 0 . 121819733 |
| AP5S1 | 0 .29471478 | 7 .282517493 | 2 .40696 | 0 .02054 | 0 . 121836429 |
| ZNF606 | -0 .393911306 | 6 .041583522 | -2 .4066 | 0 .02055 | 0 . 121857804 |
| POR | 0 .354061339 | 6 .778970888 | 2 .40639 | 0 .02056 | 0 . 121857804 |
| TAGLN | -0 .670470306 | 8 .762729933 | -2 .4062 | 0 .02057 | 0 . 121857804 |
| UBR7 | -0 .256542264 | 7 .304106961 | -2 .406 | 0 .02058 | 0 . 121857804 |
| CPED1 | -0 .313176325 | 6 .687226306 | -2 .4058 | 0 .02059 | 0 . 121857804 |
| MAGEA8 | 0 .347741731 | 5 .079605027 | 2 .4057 | 0 .0206 | 0 . 121857804 |
| MAPK12 | 0 .510162171 | 5 .810853678 | 2 .4022 | 0 .02077 | 0 . 122828035 |
| HIST1H2AK | -0 .53572198 | 4 .073604575 | -2 .4016 | 0 .0208 | 0 . 122958378 |
| ECT2 | 0 .500714634 | 6 . 139408645 | 2 .40121 | 0 .02082 | 0 . 123002791 |
| PRKCD | 0 .350263141 | 7 .536464972 | 2 .40055 | 0 .02085 | 0 . 12313841 |

| CD7 0 .679464425 | 7 .20270504 | 2 .39998 | 0 .02088 | 0 . 123186241 |
| --- | --- | --- | --- | --- |
| RBM6 -0 .316877063 | 6 .804315092 | -2 .3999 | 0 .02088 | 0 . 123186241 |
| GUSB 0 .43375068 | 9 .074017427 | 2 .3998 | 0 .02089 | 0 . 123186241 |
| ZBED5 -0 .453817899 | 7 .814937992 | -2 .3991 | 0 .02093 | 0 . 123277926 |
| DBF4B 0 .459911337 | 6 . 145134132 | 2 .39901 | 0 .02093 | 0 . 123277926 |
| PHACTR2 -0 .266489826 | 8 .494637176 | -2 .3989 | 0 .02094 | 0 . 123277926 |
| TSSC4 0 .316966958 | 6 . 144394009 | 2 .39871 | 0 .02095 | 0 . 123277926 |
| FRMD1 -0 .513036591 | 7 .805172893 | -2 .3976 | 0 .021 | 0 . 123546208 |
| MYLIP -0 .418313672 | 8 .040930408 | -2 .3972 | 0 .02102 | 0 . 123622073 |
| PDIA4 0 .355555298 | 8 .981213775 | 2 .39605 | 0 .02108 | 0 . 123834959 |
| KCTD15 -0 . 188688731 | 7 .615453096 | -2 .396 | 0 .02108 | 0 . 123834959 |
| CD3G 0 .691920417 | 5 .937535352 | 2 .39489 | 0 .02114 | 0 . 124073331 |
| FRMD4A -0 .420523531 | 6 .490411498 | -2 .3948 | 0 .02114 | 0 . 124073331 |
| ARHGEF10 -0 .315624014 | 7 .379631253 | -2 .3946 | 0 .02115 | 0 . 12407343 |
| ROCK2 -0 .214603532 | 7 .782573873 | -2 .3945 | 0 .02116 | 0 . 12407343 |
| SENP5 0 .348581561 | 9 .565900218 | 2 .39392 | 0 .02119 | 0 . 124161757 |
| C8orf33 0 .400534836 | 7 .414491862 | 2 .39377 | 0 .02119 | 0 . 124161757 |
| NDUFB1 -0 .431755733 | 8 .831667739 | -2 .393 | 0 .02124 | 0 . 124313554 |
| TRAF3IP3 0 .425762085 | 6 .834066551 | 2 .39287 | 0 .02124 | 0 . 124313554 |
| MS4A4A 0 .545538468 | 6 .52567818 | 2 .39258 | 0 .02126 | 0 . 124341378 |
| PMPCB -0 .264694453 | 8 . 194010758 | -2 .3922 | 0 .02127 | 0 . 124381055 |
| TIMM17A 0 .363764835 | 8 .70578005 | 2 .39135 | 0 .02132 | 0 . 124590917 |
| IFNAR2 0 .344251488 | 7 .456573649 | 2 .39091 | 0 .02134 | 0 . 124626347 |
| RBM38 0 .469113057 | 6 .668234499 | 2 .39075 | 0 .02135 | 0 . 124626347 |
| PPP2R2B -0 .309800441 | 7 .564675145 | -2 .3903 | 0 .02137 | 0 . 124626347 |
| BAZ2B -0 .563044559 | 6 .477525053 | -2 .3902 | 0 .02138 | 0 . 124626347 |
| ZNF675 -0 .463743884 | 5 .344832522 | -2 .3901 | 0 .02138 | 0 . 124626347 |
| CBLL1 0 .333671519 | 5 .247385572 | 2 .39006 | 0 .02138 | 0 . 124626347 |
| GML -0 .624643128 | 6 .480643364 | -2 .3898 | 0 .0214 | 0 . 12463704 |
| NOP14 0 .35507417 | 7 .426193207 | 2 .38955 | 0 .02141 | 0 . 124664372 |
| FXYD3 -0 .529146923 | 10 .40829427 | -2 .3888 | 0 .02145 | 0 . 124839529 |
| STEAP1B 0 .96444145 | 5 .381385508 | 2 .388 | 0 .02149 | 0 . 125008547 |
| SUOX -0 .308180703 | 7 . 1 19168737 | -2 .3875 | 0 .02151 | 0 . 125089846 |
| ARPIN -0 .330792472 | 6 .380527367 | -2 .3873 | 0 .02152 | 0 . 125089846 |
| PRSS53 0 .52849205 | 6 .017914309 | 2 .38642 | 0 .02157 | 0 . 125306581 |
| ELF1 0 .290845885 | 9 .547992916 | 2 .38602 | 0 .02159 | 0 . 12537106 |
| DSC3 -0 .734712187 | 9 .938607127 | -2 .3856 | 0 .02161 | 0 . 125431102 |
| CAPN7 -0 .391597396 | 7 .41076525 | -2 .3854 | 0 .02162 | 0 . 125431102 |
| KIAA0141 -0 .294947752 | 8 .596874825 | -2 .3851 | 0 .02164 | 0 . 125471337 |
| KLHL36 0 .384449789 | 5 .490086797 | 2 .38465 | 0 .02166 | 0 . 125548488 |
| GPRC5D 0 .438602803 | 6 .88207667 | 2 .38422 | 0 .02168 | 0 . 125620048 |
| SLC16A6 1 .03022199 | 6 .00629602 | 2 .38391 | 0 .0217 | 0 . 125644088 |
| HECA -0 .296868389 | 8 .757733341 | -2 .3836 | 0 .02171 | 0 . 125644088 |
| PNPLA2 -0 .372041449 | 9 .613718882 | -2 .3836 | 0 .02172 | 0 . 125644088 |
| HPR 1 . 178374155 | 3 .5405318 | 2 .38162 | 0 .02182 | 0 . 126166581 |
| GIPC1 0 .348868283 | 8 .014653578 | 2 .38084 | 0 .02186 | 0 . 126289651 |
| SERTAD3 -0 .331301474 | 6 .53620187 | -2 .3808 | 0 .02186 | 0 . 126289651 |
| MT2A 0 .488732061 | 1 1 .99597136 | 2 .37904 | 0 .02195 | 0 . 126771336 |
| PIPOX 0 .396048025 | 5 .941656082 | 2 .37884 | 0 .02196 | 0 . 126771336 |
| PITPNB 0 .244353087 | 9 .098091057 | 2 .37814 | 0 .022 | 0 . 126912225 |
| B4GALT5 0 .372464016 | 9 .60658413 | 2 .37799 | 0 .02201 | 0 . 126912225 |
| MYCBP2 -0 .412787778 | 9 .738284761 | -2 .3774 | 0 .02204 | 0 . 127018221 |
| LOC100272216 -0 .688163959 | 6 .271391673 | -2 .3768 | 0 .02207 | 0 . 12708891 |
| MYO9A -0 .257868434 | 7 .247077859 | -2 .3767 | 0 .02207 | 0 . 12708891 |
| OXA1L -0 .298539808 | 9 . 192665961 | -2 .3766 | 0 .02208 | 0 . 12708891 |
| TPMT 0 .485472347 | 8 .591039557 | 2 .37595 | 0 .02212 | 0 . 127237664 |

| GPS1 | 0 .268559229 | 7 .405721898 | 2 .37575 | 0 .02213 | 0 . 127239816 |
| --- | --- | --- | --- | --- | --- |
| ASTN1 | -0 .676880842 | 4 .23112284 | -2 .3752 | 0 .02215 | 0 . 127339106 |
| PITX1 | 0 .468181854 | 4 .997569608 | 2 .37433 | 0 .0222 | 0 . 127490375 |
| XPC | -0 .363785346 | 8 .055013515 | -2 .3742 | 0 .02221 | 0 . 127490375 |
| STIL | 0 .379483464 | 5 .353338227 | 2 .37398 | 0 .02222 | 0 . 127490375 |
| CPD | -0 .278319822 | 8 .385430855 | -2 .3738 | 0 .02223 | 0 . 127490375 |
| CCL2 | 0 .848760989 | 7 .522350169 | 2 .37369 | 0 .02224 | 0 . 127490375 |
| EIF2D | -0 .279507532 | 8 .57631688 | -2 .3735 | 0 .02225 | 0 . 127490375 |
| TNFSF18 | 0 .718788224 | 6 .02432752 | 2 .37339 | 0 .02225 | 0 . 127490375 |
| PLEKHF1 | 0 .459279917 | 6 .933446602 | 2 .37314 | 0 .02226 | 0 . 127508021 |
| GOLGB1 | -0 .336283845 | 8 .364496182 | -2 .3721 | 0 .02232 | 0 . 127774674 |
| MMP14 | 0 .291520891 | 10 .90409592 | 2 .37065 | 0 .0224 | 0 . 128148078 |
| PDE4B | 0 .453032664 | 7 .603272033 | 2 .3696 | 0 .02245 | 0 . 128368988 |
| SLC43A3 | 0 .325317981 | 9 .393926624 | 2 .36955 | 0 .02246 | 0 . 128368988 |
| PLA2G4C | -0 .512095113 | 6 .62750815 | -2 .3689 | 0 .02249 | 0 . 128465531 |
| RBM47 | -0 .779886511 | 7 .035844219 | -2 .3688 | 0 .02249 | 0 . 128465531 |
| PLBD1 | 0 .739342709 | 8 . 100424012 | 2 .36858 | 0 .02251 | 0 . 128475949 |
| FCGBP | -1 . 106768466 | 7 .392330676 | -2 .3684 | 0 .02252 | 0 . 128475949 |
| ZMYM5 | 0 .245573214 | 6 .719352394 | 2 .36822 | 0 .02253 | 0 . 128482787 |
| KERA | 0 .799124106 | 3 .601787992 | 2 .36779 | 0 .02255 | 0 . 128556217 |
| PPM1D | -0 .34570535 | 6 .012244564 | -2 .3663 | 0 .02263 | 0 . 128946721 |
| GPT | -0 .896270502 | 4 .590198273 | -2 .366 | 0 .02264 | 0 . 128982196 |
| LSM14A | -0 .255277859 | 10 .01344533 | -2 .3654 | 0 .02268 | 0 . 129104318 |
| TRMT5 | -0 .278783685 | 9 .945550627 | -2 .365 | 0 .0227 | 0 . 129104318 |
| MTA1 | -0 .39881767 | 8 .558578927 | -2 .3649 | 0 .02271 | 0 . 129104318 |
| FASTKD3 | 0 .297723004 | 6 .484795539 | 2 .36472 | 0 .02271 | 0 . 129104318 |
| IFT74 | -0 .449310828 | 5 .437850483 | -2 .3647 | 0 .02272 | 0 . 129104318 |
| DIP2C | -0 .453169101 | 9 . 163270282 | -2 .3643 | 0 .02274 | 0 . 12915899 |
| PTTG1IP | 0 .35088133 | 10 .78539779 | 2 .36314 | 0 .0228 | 0 . 129452859 |
| SDF2L1 | 0 .357653679 | 7 .970586355 | 2 .36249 | 0 .02283 | 0 . 129594086 |
| ACSL4 | -0 .507693547 | 6 . 188161217 | -2 .3621 | 0 .02286 | 0 . 129662514 |
| LOC101930405 | 1 .958046171 | 4 .966642186 | 2 .36149 | 0 .02289 | 0 . 129784385 |
| SZT2 | -0 .256171947 | 7 .334039633 | -2 .3608 | 0 .02292 | 0 . 129924133 |
| SQLE | 0 .499557327 | 9 .305859075 | 2 .36057 | 0 .02294 | 0 . 129950257 |
| PLD3 | 0 .350691577 | 9 .638787379 | 2 .35895 | 0 .02303 | 0 . 130392099 |
| OGFOD1 | 0 .269872622 | 7 .532975343 | 2 .35859 | 0 .02305 | 0 . 130445351 |
| CD81 | 0 .266697886 | 1 1 .80753962 | 2 .35819 | 0 .02307 | 0 . 130509532 |
| DDR2 | -0 .536225445 | 7 .534466912 | -2 .3578 | 0 .02309 | 0 . 130566336 |
| PRDM1 | 0 .431808461 | 6 .891628276 | 2 .35724 | 0 .02312 | 0 . 130688185 |
| CRNKL1 | -0 .304859662 | 6 .392276906 | -2 .3558 | 0 .0232 | 0 . 131043472 |
| ITGA6 | -0 .521062542 | 9 .886996607 | -2 .3557 | 0 .02321 | 0 . 131043472 |
| CRELD2 | 0 .351660579 | 8 .678125525 | 2 .35515 | 0 .02324 | 0 . 13115822 |
| SSBP1 | 0 .282695587 | 8 .958616704 | 2 .35476 | 0 .02326 | 0 . 131169232 |
| LRP12 | 0 .590647372 | 6 .236065567 | 2 .35474 | 0 .02326 | 0 . 131169232 |
| SGCE | -0 .922471727 | 6 .814199155 | -2 .3543 | 0 .02328 | 0 . 131242395 |
| IL33 | -0 .636524514 | 5 .873618958 | -2 .3536 | 0 .02332 | 0 . 131359417 |
| SMARCB1 | 0 .244911425 | 8 .552537913 | 2 .35338 | 0 .02333 | 0 . 131359417 |
| CASD1 | -0 .393072831 | 5 .860853294 | -2 .3532 | 0 .02334 | 0 . 131359417 |
| TPST1 | 0 .303657752 | 6 .509065454 | 2 .35318 | 0 .02334 | 0 . 131359417 |
| C6orf25 | 0 .321108531 | 5 .947361628 | 2 .35226 | 0 .0234 | 0 . 131573415 |
| MYL9 | -0 .71214967 | 8 .565501101 | -2 .3521 | 0 .0234 | 0 . 131573415 |
| IRAK3 | -0 .474212013 | 5 .539426316 | -2 .3517 | 0 .02343 | 0 . 131596383 |
| TAF7 | -0 .438696662 | 8 .080485573 | -2 .3511 | 0 .02346 | 0 . 131596383 |
| FBXO11 | -0 .474976154 | 7 .81132544 | -2 .3511 | 0 .02346 | 0 . 131596383 |
| BTN2A3P | 0 .63285158 | 4 .402215374 | 2 .35106 | 0 .02346 | 0 . 131596383 |
| MPZL1 | -0 .262658322 | 9 .727862948 | -2 .3509 | 0 .02347 | 0 . 131596383 |

| RCL1 | -0 .266382463 | 7 . 186458823 | -2 .3509 | 0 .02347 | 0 . 131596383 |
| --- | --- | --- | --- | --- | --- |
| PTMS | 0 .475320426 | 8 .082347703 | 2 .34859 | 0 .0236 | 0 . 132261203 |
| SCAMP3 | 0 .424130968 | 8 .961008404 | 2 .34828 | 0 .02362 | 0 . 132270221 |
| NCKAP1 | -0 .346445828 | 9 .201317668 | -2 .3482 | 0 .02362 | 0 . 132270221 |
| B3GALNT1 | -0 .300251108 | 5 .621924605 | -2 .3475 | 0 .02366 | 0 . 132379446 |
| TDP2 | -0 .412385673 | 6 .893600969 | -2 .3475 | 0 .02366 | 0 . 132379446 |
| PCDH17 | 0 .366696409 | 6 . 128565856 | 2 .34694 | 0 .02369 | 0 . 132426875 |
| CDC7 | 0 .421745094 | 6 .248012465 | 2 .34693 | 0 .02369 | 0 . 132426875 |
| ZNF710 | 0 .31666504 | 8 .482129434 | 2 .34623 | 0 .02373 | 0 . 132585564 |
| C5 | -0 .497701131 | 5 .925539368 | -2 .3443 | 0 .02384 | 0 . 133060838 |
| DSPP | 0 .402217718 | 4 .61971742 | 2 .34406 | 0 .02385 | 0 . 133060838 |
| KRT8P12 | 0 .410219387 | 5 .761440641 | 2 .34394 | 0 .02386 | 0 . 133060838 |
| RAB15 | -0 .220919336 | 8 .080223716 | -2 .3439 | 0 .02386 | 0 . 133060838 |
| C14orf2 | -0 .482375603 | 9 .220746023 | -2 .3438 | 0 .02387 | 0 . 133060838 |
| PKD1P1 | -0 .479312618 | 5 .220821734 | -2 .343 | 0 .02392 | 0 . 133257649 |
| LRPAP1 | 0 .451982273 | 9 .663968819 | 2 .34216 | 0 .02396 | 0 . 133452766 |
| CHEK2 | -0 .289927645 | 6 .573929326 | -2 .3415 | 0 .024 | 0 . 13358821 |
| MAGEC1 | 0 .453304494 | 5 .506352405 | 2 .34051 | 0 .02405 | 0 . 133801377 |
| KRT75 | 0 .583999234 | 8 .088317346 | 2 .34033 | 0 .02407 | 0 . 133801377 |
| TMOD1 | -0 .430152101 | 7 .809337341 | -2 .3403 | 0 .02407 | 0 . 133801377 |
| TM9SF1 | 0 .318354977 | 6 .92714245 | 2 .34003 | 0 .02408 | 0 . 133801377 |
| HOXA3 | 0 .68394995 | 4 .046664744 | 2 .33978 | 0 .0241 | 0 . 133801377 |
| MTF2 | -0 .402281437 | 8 .983554517 | -2 .3397 | 0 .0241 | 0 . 133801377 |
| NCBP2 | 0 .268696295 | 8 .651979149 | 2 .33876 | 0 .02415 | 0 . 134051749 |
| DHX34 | -0 .422363708 | 7 . 137636941 | -2 .338 | 0 .0242 | 0 . 134240136 |
| TOMM20 | -0 .497455559 | 10 .38052202 | -2 .3362 | 0 .0243 | 0 . 134757064 |
| APTX | -0 .294710382 | 6 .829161381 | -2 .3358 | 0 .02433 | 0 . 134795907 |
| CNBP | -0 .31289116 | 10 .07731217 | -2 .3357 | 0 .02433 | 0 . 134795907 |
| ZDHHC7 | -0 .321589748 | 8 .948347119 | -2 .334 | 0 .02443 | 0 . 135263901 |
| ABCA12 | 0 .597830305 | 5 .931797689 | 2 .33371 | 0 .02444 | 0 . 135300565 |
| TSSC1 | 0 .268468686 | 7 .428519036 | 2 .33259 | 0 .02451 | 0 . 135564975 |
| ANXA2P2 | 0 .378390633 | 1 1 .62406623 | 2 .33251 | 0 .02451 | 0 . 135564975 |
| ARHGEF11 | 0 .341834271 | 8 .652270936 | 2 .33202 | 0 .02454 | 0 . 135655302 |
| OGT | -0 .330537708 | 9 .451201872 | -2 .3314 | 0 .02458 | 0 . 135655302 |
| CBR4 | -0 .507536821 | 6 .761689805 | -2 .3313 | 0 .02458 | 0 . 135655302 |
| HTR7 | -1 .095943545 | 4 .544558321 | -2 .3312 | 0 .02459 | 0 . 135655302 |
| MED13L | -0 .370844561 | 9 . 162227753 | -2 .3311 | 0 .02459 | 0 . 135655302 |
| HLA-F-AS1 | -0 .634528863 | 7 .232477336 | -2 .3311 | 0 .02459 | 0 . 135655302 |
| FUT7 | 0 .531302096 | 5 .240427339 | 2 .33052 | 0 .02463 | 0 . 135782784 |
| SPCS1 | 0 .297170084 | 10 .07465986 | 2 .32973 | 0 .02467 | 0 . 135974179 |
| MPDZ | -0 .500646055 | 7 .409597032 | -2 .3293 | 0 .0247 | 0 . 1360004 |
| KCTD13 | 0 .330575812 | 7 .558744315 | 2 .32928 | 0 .0247 | 0 . 1360004 |
| CAV3 | 0 .294527095 | 7 .225350586 | 2 .32875 | 0 .02473 | 0 . 136054496 |
| GLB1L | 0 .407101498 | 7 .351414342 | 2 .32874 | 0 .02473 | 0 . 136054496 |
| ABL2 | 0 .289926071 | 6 .215227222 | 2 .32797 | 0 .02478 | 0 . 136214344 |
| RNF170 | -0 .461060329 | 6 .060050315 | -2 .3279 | 0 .02478 | 0 . 136214344 |
| TNKS2 | -0 .251590973 | 8 . 193357885 | -2 .3271 | 0 .02483 | 0 . 136334041 |
| EIF2AK3 | 0 .483362431 | 7 .401542035 | 2 .32712 | 0 .02483 | 0 . 136334041 |
| ENTPD6 | 0 .357693798 | 7 .402540507 | 2 .32615 | 0 .02488 | 0 . 136583002 |
| ICAM1 | 0 .302430349 | 9 .322951595 | 2 .32597 | 0 .02489 | 0 . 136583002 |
| IFI44 | 0 .465530189 | 7 .945118065 | 2 .32494 | 0 .02495 | 0 . 136818159 |
| AXL | -0 .420456157 | 8 .411750751 | -2 .3249 | 0 .02496 | 0 . 136818159 |
| MAF | -0 .390290552 | 9 .527612757 | -2 .3244 | 0 .02498 | 0 . 13689003 |
| LEMD3 | -0 .399259322 | 6 .640725117 | -2 .3243 | 0 .02499 | 0 . 13689003 |
| NUDT15 | 0 .453960467 | 6 .706067857 | 2 .32292 | 0 .02507 | 0 . 137218962 |
| EDEM2 | 0 .265955942 | 7 .389168297 | 2 .32272 | 0 .02508 | 0 . 137218962 |

| TRAF3IP1 | 0 .371069709 | 5 .994883567 | 2 .3227 | 0 .02509 | 0 . 137218962 |
| --- | --- | --- | --- | --- | --- |
| LPPR2 | 0 .289122722 | 7 .961507142 | 2 .3225 | 0 .0251 | 0 . 137222534 |
| TRIM14 | 0 .307662034 | 9 .279458127 | 2 .32155 | 0 .02515 | 0 . 137438026 |
| FAS | -0 .350663944 | 8 .299606671 | -2 .3215 | 0 .02516 | 0 . 137438026 |
| HSPA5 | 0 .482766568 | 9 .648133968 | 2 .32113 | 0 .02518 | 0 . 13745136 |
| HLA-E | 0 .348132508 | 13 .26583526 | 2 .32105 | 0 .02518 | 0 . 13745136 |
| MTCH1 | -0 .262089928 | 10 .38922484 | -2 .3204 | 0 .02522 | 0 . 137558586 |
| RTN2 | 0 .224468032 | 9 . 108013899 | 2 .32035 | 0 .02522 | 0 . 137558586 |
| INPP5K | 0 .365589734 | 8 .88101654 | 2 .31998 | 0 .02525 | 0 . 137618733 |
| NADK | 0 . 196674092 | 9 .727314975 | 2 .31851 | 0 .02533 | 0 . 137827123 |
| PCDH9 | 0 .576534894 | 6 .007396869 | 2 .31841 | 0 .02534 | 0 . 137827123 |
| DPP8 | -0 .350180083 | 8 .380451596 | -2 .3183 | 0 .02534 | 0 . 137827123 |
| MAGEA12 | 1 . 13004495 | 5 .743382084 | 2 .31829 | 0 .02535 | 0 . 137827123 |
| DHX58 | 0 .427610703 | 5 .285957969 | 2 .3182 | 0 .02535 | 0 . 137827123 |
| CECR1 | 0 .870468707 | 8 .870125441 | 2 .31817 | 0 .02535 | 0 . 137827123 |
| BCAN | -0 .591126861 | 9 .807851546 | -2 .318 | 0 .02536 | 0 . 137827123 |
| CKMT2 | -0 .618042296 | 6 .435979728 | -2 .3173 | 0 .02541 | 0 . 13801274 |
| MDN1 | -0 .333911 109 | 6 .856904136 | -2 .3171 | 0 .02542 | 0 . 13801274 |
| NKX2-2 | 0 .796791047 | 4 .86239907 | 2 .31624 | 0 .02547 | 0 . 13822651 |
| CLOCK | -0 .26443568 | 8 . 1 12226095 | -2 .3161 | 0 .02548 | 0 . 13822651 |
| CISD1 | -0 .42968002 | 9 .280453115 | -2 .3157 | 0 .0255 | 0 . 138286091 |
| NDUFS7 | 0 .324791528 | 8 .487792245 | 2 .31541 | 0 .02552 | 0 . 138295569 |
| SRP9 | -0 .441395715 | 9 .565504884 | -2 .3153 | 0 .02553 | 0 . 138295569 |
| BBS10 | -0 .533637272 | 5 .520511778 | -2 .3137 | 0 .02562 | 0 . 138718582 |
| UBA52 | 0 . 183525289 | 1 1 .98348513 | 2 .31364 | 0 .02563 | 0 . 138718582 |
| SMA4 | -0 .629199104 | 5 .832112784 | -2 .3133 | 0 .02564 | 0 . 138763551 |
| EIF1B | -0 .423972381 | 9 . 1 19057977 | -2 .3125 | 0 .02569 | 0 . 138959992 |
| SAMD4A | -0 .311527035 | 8 .33163956 | -2 .3114 | 0 .02576 | 0 . 139276352 |
| OBSCN | 0 .405432252 | 5 .052145466 | 2 .31101 | 0 .02578 | 0 . 139336851 |
| OPA3 | 0 .502428769 | 4 .435050415 | 2 .30954 | 0 .02587 | 0 . 139759298 |
| PUS7 | 0 .45349023 | 6 .803819993 | 2 .30934 | 0 .02589 | 0 . 139762975 |
| PLA2G15 | 0 .418332308 | 6 .933677505 | 2 .30902 | 0 .0259 | 0 . 139807997 |
| HTR2C | 0 .425902912 | 5 .45520733 | 2 .30851 | 0 .02594 | 0 . 139916095 |
| SCG2 | 0 .926406184 | 5 .202871819 | 2 .3082 | 0 .02595 | 0 . 139920303 |
| 3-Mar | 0 .41279354 | 6 . 144334205 | 2 .30813 | 0 .02596 | 0 . 139920303 |
| TPP1 | 0 .383933733 | 1 1 .30100428 | 2 .30767 | 0 .02599 | 0 . 140010828 |
| PDZK1IP1 | 0 .839715501 | 7 .349968586 | 2 .30681 | 0 .02604 | 0 . 140148433 |
| BRD1 | -0 .310585809 | 8 . 125762336 | -2 .3067 | 0 .02604 | 0 . 140148433 |
| DIRAS3 | -0 .430753429 | 5 .012979465 | -2 .3067 | 0 .02605 | 0 . 140148433 |
| TAPBPL | 0 .410177978 | 7 .424590361 | 2 .3064 | 0 .02606 | 0 . 140156687 |
| ANP32B | -0 .346495005 | 1 1 . 1 1542777 | -2 .3063 | 0 .02607 | 0 . 140156687 |
| CWF19L1 | 0 .395800319 | 6 . 173333661 | 2 .30603 | 0 .02609 | 0 . 140187861 |
| ARL4C | 0 .521770162 | 9 .645664408 | 2 .30527 | 0 .02613 | 0 . 140377628 |
| NDEL1 | 0 .322047079 | 8 . 1 13093137 | 2 .30422 | 0 .0262 | 0 . 140664273 |
| RIOK3 | -0 .320825671 | 9 .790257732 | -2 .3039 | 0 .02622 | 0 . 140697205 |
| SPAG4 | 0 .573008214 | 4 .797955244 | 2 .30316 | 0 .02626 | 0 . 140892822 |
| KLK8 | 0 .63320251 | 8 .214993672 | 2 .3027 | 0 .02629 | 0 . 140982137 |
| RACGAP1 | 0 .437365298 | 7 .356830254 | 2 .30225 | 0 .02632 | 0 . 141072475 |
| IDE | 0 .399104825 | 8 .987379308 | 2 .30053 | 0 .02643 | 0 . 14158055 |
| CYBRD1 | -0 .620181273 | 5 .421375081 | -2 .3001 | 0 .02645 | 0 . 141645867 |
| CRAT | -0 .76282454 | 8 .432546871 | -2 .2997 | 0 .02648 | 0 . 141738596 |
| FYB | 0 .557348521 | 7 .718481996 | 2 .29949 | 0 .02649 | 0 . 141743567 |
| PRKCG | 0 .720176488 | 2 .408580248 | 2 .29865 | 0 .02654 | 0 . 14196145 |
| BCL2A1 | 0 .572990708 | 6 .821622082 | 2 .29835 | 0 .02656 | 0 . 141999428 |
| ADAM2 | 0 .78141254 | 2 .968698385 | 2 .29758 | 0 .02661 | 0 . 142195684 |
| ZNF302 | -0 .39948133 | 6 .516108707 | -2 .297 | 0 .02664 | 0 . 142296651 |

| RPN1 | 0 .322194716 | 9 .439157266 | 2 .29691 | 0 .02665 | 0 . 142296651 |
| --- | --- | --- | --- | --- | --- |
| CSNK1G1 | 0 .35115731 | 6 .373048853 | 2 .29569 | 0 .02673 | 0 . 142608031 |
| UBE2L3 | 0 .209594819 | 10 .48710199 | 2 .29543 | 0 .02674 | 0 . 142608031 |
| B9D2 | -0 .563773289 | 4 .200475884 | -2 .2954 | 0 .02674 | 0 . 142608031 |
| HLA-DRB6 | 0 .794646102 | 7 .251970599 | 2 .29495 | 0 .02677 | 0 . 142672054 |
| LRRC61 | 0 .412262177 | 6 .52905584 | 2 .29487 | 0 .02678 | 0 . 142672054 |
| FAM89B | -0 .226452308 | 10 .96339078 | -2 .2936 | 0 .02686 | 0 . 143049193 |
| CYB561D2 | 0 .30571938 | 7 .594229154 | 2 .29322 | 0 .02688 | 0 . 143101272 |
| ABCD4 | -0 .347796993 | 7 .415916416 | -2 .2928 | 0 .02691 | 0 . 143157677 |
| SDC2 | -0 .579245854 | 9 .47810547 | -2 .2927 | 0 .02691 | 0 . 143157677 |
| ELAVL1 | 0 .234578915 | 9 .230362464 | 2 .29195 | 0 .02696 | 0 . 143341761 |
| FGF5 | 0 .345828823 | 7 .064378925 | 2 .29178 | 0 .02697 | 0 . 143341761 |
| TRIM26 | 0 .284037263 | 7 .821484608 | 2 .29125 | 0 .027 | 0 . 143457028 |
| FAM160B2 | 0 .272544528 | 7 .739140441 | 2 .28996 | 0 .02709 | 0 . 143827706 |
| AHNAK | -0 .353190129 | 1 1 .23483187 | -2 .2893 | 0 .02713 | 0 . 143990005 |
| KLRG1 | 0 .303953514 | 8 .231576429 | 2 .28737 | 0 .02725 | 0 . 14453989 |
| CBX6 | -0 .32281307 | 10 .06476687 | -2 .2873 | 0 .02725 | 0 . 14453989 |
| TWISTNB | -0 .460052707 | 4 .063743149 | -2 .2866 | 0 .0273 | 0 . 144723097 |
| TFE3 | 0 .237629719 | 9 . 152444418 | 2 .28591 | 0 .02734 | 0 . 144865673 |
| AKAP12 | -0 .835842441 | 6 .955165988 | -2 .2858 | 0 .02735 | 0 . 144865673 |
| ACTR1A | 0 .241697636 | 8 .993359266 | 2 .28556 | 0 .02736 | 0 . 144865673 |
| PAPD7 | -0 .285064831 | 7 .491679724 | -2 .2854 | 0 .02737 | 0 . 144865673 |
| DEGS1 | -0 .475260613 | 9 .866962556 | -2 .2847 | 0 .02742 | 0 . 14500807 |
| WDR59 | -0 .275193871 | 7 .79373243 | -2 .2846 | 0 .02742 | 0 . 14500807 |
| WNT5A | 0 .436963455 | 7 . 19751791 | 2 .28399 | 0 .02747 | 0 . 145156746 |
| TROVE2 | -0 .497779185 | 9 .85225471 | -2 .2838 | 0 .02747 | 0 . 145156746 |
| CLSTN3 | -0 .676712236 | 6 .636528579 | -2 .2832 | 0 .02752 | 0 . 145247056 |
| CELSR2 | -0 .405162436 | 9 . 15985324 | -2 .2832 | 0 .02752 | 0 . 145247056 |
| POLR1E | -0 .369438154 | 6 .982967594 | -2 .283 | 0 .02753 | 0 . 145247056 |
| LDHC | 0 .379365106 | 5 .53784364 | 2 .28221 | 0 .02758 | 0 . 145351102 |
| STAR | -0 .733370102 | 4 .670751559 | -2 .2822 | 0 .02758 | 0 . 145351102 |
| FAM179B | -0 .402494311 | 5 .877110379 | -2 .2822 | 0 .02758 | 0 . 145351102 |
| C11orf58 | -0 .441072539 | 9 .750085876 | -2 .282 | 0 .02759 | 0 . 145357726 |
| FDXR | 0 .431913957 | 5 .947301299 | 2 .28165 | 0 .02761 | 0 . 145409342 |
| ISLR | -0 .560284189 | 7 .739526464 | -2 .2815 | 0 .02763 | 0 . 145409413 |
| ITGB2 | 0 .818788338 | 9 . 174725026 | 2 .2802 | 0 .02771 | 0 . 145735751 |
| RAB31 | 0 .463787727 | 9 .759792874 | 2 .27952 | 0 .02775 | 0 . 145735751 |
| UQCRC2 | -0 .418595181 | 9 .59959392 | -2 .2795 | 0 .02775 | 0 . 145735751 |
| NCF1 | 0 .552211834 | 5 .383240507 | 2 .27942 | 0 .02776 | 0 . 145735751 |
| GALNT11 | 0 .334240644 | 7 .730390257 | 2 .27914 | 0 .02778 | 0 . 145735751 |
| CCDC90B | -0 .285957211 | 7 .069997887 | -2 .2791 | 0 .02778 | 0 . 145735751 |
| CCDC92 | 0 .233935419 | 9 .513814902 | 2 .27907 | 0 .02778 | 0 . 145735751 |
| PODNL1 | -0 .978888261 | 4 .967691676 | -2 .2789 | 0 .02779 | 0 . 145735751 |
| TMEM176A | 0 .577171449 | 8 .585202376 | 2 .27878 | 0 .0278 | 0 . 145735751 |
| IDH1 | -0 .537080436 | 8 .506097603 | -2 .2786 | 0 .02781 | 0 . 145735751 |
| NOTCH3 | -0 .446418201 | 9 .790169989 | -2 .2781 | 0 .02784 | 0 . 145735751 |
| PDE2A | -0 .557448061 | 7 .054279623 | -2 .2781 | 0 .02785 | 0 . 145735751 |
| MCM9 | -0 .307060585 | 7 .390825641 | -2 .278 | 0 .02785 | 0 . 145735751 |
| RAB20 | 0 .400717071 | 6 .791769033 | 2 .27799 | 0 .02785 | 0 . 145735751 |
| ZNF592 | 0 .22788854 | 8 . 141878774 | 2 .27661 | 0 .02794 | 0 . 146143267 |
| HUS1 | 0 .278407185 | 7 .881280472 | 2 .27597 | 0 .02798 | 0 . 1462516 |
| ATP5F1 | -0 .321864968 | 9 .608358812 | -2 .2758 | 0 .02799 | 0 . 1462516 |
| DEAF1 | 0 .274285779 | 7 .804964087 | 2 .27576 | 0 .028 | 0 . 1462516 |
| TREM2 | 1 .022254328 | 3 .841239422 | 2 .27538 | 0 .02802 | 0 . 146320588 |
| AP3S1 | -0 .28423457 | 9 .306708263 | -2 .2746 | 0 .02807 | 0 . 146521567 |
| HNMT | -0 .603939855 | 7 .782172989 | -2 .2744 | 0 .02808 | 0 . 146521567 |

| ELF2 | -0 .259198225 | 7 .793345507 | -2 .2733 | 0 .02816 | 0 . 146849781 |
| --- | --- | --- | --- | --- | --- |
| LAPTM5 | 0 .798383356 | 10 .27634603 | 2 .27277 | 0 .02819 | 0 . 146964871 |
| NT5DC3 | 0 .371646073 | 6 .392349592 | 2 .27239 | 0 .02822 | 0 . 146979073 |
| HPSE2 | -0 .827640064 | 4 .723592812 | -2 .272 | 0 .02824 | 0 . 146979073 |
| GSE1 | -0 .344976803 | 8 . 188029195 | -2 .272 | 0 .02824 | 0 . 146979073 |
| DHFR | 0 .297313554 | 9 .441148895 | 2 .27177 | 0 .02826 | 0 . 146979073 |
| ABHD17B | -0 .456922804 | 4 .948208171 | -2 .2718 | 0 .02826 | 0 . 146979073 |
| GNPAT | -0 .42862024 | 8 .509330737 | -2 .2715 | 0 .02827 | 0 . 146979073 |
| TARBP2 | 0 .243771123 | 7 .763448345 | 2 .27148 | 0 .02828 | 0 . 146979073 |
| ANKZF1 | -0 .314227282 | 6 .719213139 | -2 .2698 | 0 .02839 | 0 . 147487899 |
| BAG6 | 0 .270724667 | 1 1 .65747345 | 2 .26936 | 0 .02842 | 0 . 147580478 |
| CD1C | -0 .439452909 | 8 .366625772 | -2 .2692 | 0 .02843 | 0 . 14759008 |
| MOGS | 0 .391866361 | 6 .938950517 | 2 .26882 | 0 .02845 | 0 . 147645195 |
| ATP5E | 0 .331715301 | 1 1 . 13559099 | 2 .26817 | 0 .02849 | 0 . 147805236 |
| ADNP2 | -0 .497419421 | 8 .73661968 | -2 .2676 | 0 .02853 | 0 . 14792607 |
| YTHDF1 | 0 .248626994 | 8 .690558845 | 2 .26714 | 0 .02856 | 0 . 148035148 |
| VDAC1 | 0 .333500108 | 10 .02936172 | 2 .26657 | 0 .0286 | 0 . 148172797 |
| DYNLL1 | 0 .392374273 | 10 .80773397 | 2 .26466 | 0 .02873 | 0 . 148727146 |
| KIAA0754 | -0 .717035489 | 3 . 127065287 | -2 .2646 | 0 .02873 | 0 . 148727146 |
| MYO9B | 0 .216206739 | 9 .0085734 | 2 .26442 | 0 .02874 | 0 . 148727146 |
| RGS17 | 0 .431147346 | 6 .689456054 | 2 .26421 | 0 .02876 | 0 . 14873747 |
| CYCS | 0 .420664381 | 9 .92851494 | 2 .26394 | 0 .02877 | 0 . 148770578 |
| HS3ST3A1 | 0 .747599328 | 4 .09540309 | 2 .26331 | 0 .02882 | 0 . 148927725 |
| PPIG | -0 .250277864 | 8 .715864722 | -2 .2629 | 0 .02884 | 0 . 149004334 |
| ROPN1B | -0 .719001505 | 6 .504192017 | -2 .2624 | 0 .02888 | 0 . 149121812 |
| PSMC6 | -0 .549761963 | 7 .346179719 | -2 .2617 | 0 .02892 | 0 . 149291516 |
| RNASE6 | 0 .833877307 | 7 .222772948 | 2 .2613 | 0 .02895 | 0 . 149373953 |
| STX1A | 0 .370567957 | 6 .432478916 | 2 .26098 | 0 .02897 | 0 . 149424286 |
| TOB1 | -0 .444523889 | 7 .938824829 | -2 .2597 | 0 .02906 | 0 . 149791999 |
| ADAM8 | 0 .54957226 | 7 . 108672415 | 2 .25941 | 0 .02908 | 0 . 149845743 |
| FAM189A1 | 0 .432036918 | 4 .85468673 | 2 .25849 | 0 .02914 | 0 . 150102882 |
| DCN | -0 .673735725 | 12 . 16071941 | -2 .258 | 0 .02917 | 0 . 150216241 |
| ABCD3 | -0 .459453906 | 7 .522614911 | -2 .2576 | 0 .0292 | 0 . 150302249 |
| RPF1 | -0 .351098522 | 7 .99840146 | -2 .2572 | 0 .02922 | 0 . 150349555 |
| EDEM3 | -0 .416637856 | 8 .059052549 | -2 .2559 | 0 .02932 | 0 . 150712453 |
| HNRNPAB | 0 .266062739 | 9 .916810898 | 2 .2558 | 0 .02932 | 0 . 150712453 |
| GAST | 0 .390874642 | 6 .59877049 | 2 .25568 | 0 .02933 | 0 . 150712453 |
| C8orf4 | 0 .583342845 | 4 .401786534 | 2 .25543 | 0 .02935 | 0 . 150736235 |
| ENO2 | 0 .673670165 | 6 .829192441 | 2 .25471 | 0 .0294 | 0 . 150926591 |
| ASB13 | -0 .304082266 | 8 .093705371 | -2 .2545 | 0 .02941 | 0 . 150926732 |
| LAMTOR3 | -0 .442544804 | 6 .730194007 | -2 .2541 | 0 .02944 | 0 . 151020833 |
| HCLS1 | 0 .777415613 | 8 .633926286 | 2 .25379 | 0 .02946 | 0 . 151061181 |
| CDK2AP2 | 0 .385323443 | 7 .756702719 | 2 .25289 | 0 .02952 | 0 . 151313463 |
| NEB | -0 .370620101 | 6 . 160989208 | -2 .2527 | 0 .02953 | 0 . 151322329 |
| TOR4A | 0 .371787787 | 7 .236697493 | 2 .25237 | 0 .02956 | 0 . 151374316 |
| C2orf27A | -0 .412074377 | 5 .694963884 | -2 .2519 | 0 .02959 | 0 . 151485208 |
| CHN1 | 0 .441514367 | 7 .2621086 | 2 .2511 1 | 0 .02964 | 0 . 151651525 |
| CUL4B | -0 .282886544 | 8 .723313831 | -2 .251 | 0 .02965 | 0 . 151651525 |
| SPRR3 | 0 .351882182 | 6 .424652379 | 2 .25072 | 0 .02967 | 0 . 151651525 |
| RNF19B | 0 .304812468 | 8 .988167156 | 2 .25069 | 0 .02967 | 0 . 151651525 |
| FLOT1 | 0 .281194892 | 1 1 .25420679 | 2 .25002 | 0 .02972 | 0 . 151825336 |
| IL27RA | 0 .37435016 | 8 . 123824244 | 2 .24896 | 0 .02979 | 0 . 152133818 |
| SLC14A1 | -0 .574527636 | 3 .808780917 | -2 .2479 | 0 .02986 | 0 . 152443972 |
| GPC1 | -0 .546981644 | 9 .00197814 | -2 .2463 | 0 .02997 | 0 . 152947197 |
| DHRS3 | -0 .407846346 | 7 .475181201 | -2 .2448 | 0 .03008 | 0 . 153422041 |
| KMT2A | -0 .266305153 | 9 .484494707 | -2 .2445 | 0 .0301 | 0 . 153473754 |

| NUBP1 | 0 .280731997 | 7 .640601791 | 2 .24403 | 0 .03013 | 0 . 153562513 |
| --- | --- | --- | --- | --- | --- |
| IFIT5 | -0 .327522594 | 7 .691039522 | -2 .2436 | 0 .03016 | 0 . 153631015 |
| HEXA | 0 .26652826 | 9 .490653155 | 2 .24349 | 0 .03017 | 0 . 153631015 |
| TMEM127 | 0 .284817458 | 7 .049568108 | 2 .24281 | 0 .03021 | 0 . 153746507 |
| LAP3 | 0 .609654867 | 8 .722793024 | 2 .24281 | 0 .03022 | 0 . 153746507 |
| ANKRD27 | -0 .33112524 | 7 .595733746 | -2 .2426 | 0 .03023 | 0 . 153746591 |
| ZNF668 | 0 .317315201 | 5 .516797636 | 2 .24168 | 0 .03029 | 0 . 153986296 |
| SPATA6L | -0 .467622809 | 4 .267000358 | -2 .2415 | 0 .03031 | 0 . 153986296 |
| NOC2L | 0 .297876429 | 7 .346933318 | 2 .24136 | 0 .03032 | 0 . 153986296 |
| PRCC | 0 .421827089 | 6 .987408657 | 2 .24124 | 0 .03032 | 0 . 153986296 |
| POLD1 | 0 .242527312 | 7 .298131927 | 2 .24092 | 0 .03035 | 0 . 153986296 |
| ACAP1 | 0 .462733544 | 6 .608018555 | 2 .2409 | 0 .03035 | 0 . 153986296 |
| OSBPL9 | -0 .64902859 | 8 .69135417 | -2 .2405 | 0 .03037 | 0 . 154055138 |
| PHKA2 | -0 .286469743 | 7 .964404113 | -2 .2387 | 0 .0305 | 0 . 154654063 |
| EFCAB14 | -0 . 176203294 | 10 .40789087 | -2 .2379 | 0 .03056 | 0 . 154789371 |
| KIAA1279 | -0 .337748338 | 7 .444255767 | -2 .2379 | 0 .03056 | 0 . 154789371 |
| MZT2B | -0 .484344796 | 6 .417124824 | -2 .2378 | 0 .03057 | 0 . 154789371 |
| PSMA7 | 0 .394040703 | 10 .05921862 | 2 .23722 | 0 .03061 | 0 . 154923791 |
| BAI3 | -0 .426205306 | 5 .324500459 | -2 .2369 | 0 .03063 | 0 . 154968057 |
| SORD | -0 .455366458 | 8 .831826338 | -2 .2362 | 0 .03068 | 0 . 155063796 |
| ACACB | -0 .343434376 | 8 .014082591 | -2 .2362 | 0 .03068 | 0 . 155063796 |
| C22orf24 | 0 .28716211 1 | 7 .750621838 | 2 .23612 | 0 .03068 | 0 . 155063796 |
| ARHGDIG | 0 .624919831 | 5 . 184497326 | 2 .23557 | 0 .03072 | 0 . 155200122 |
| P2RX7 | 0 .391684977 | 7 .552198443 | 2 .23428 | 0 .03081 | 0 . 155595596 |
| CYP4F11 | 0 .597729758 | 3 .384849804 | 2 .23382 | 0 .03085 | 0 . 155698435 |
| CDY1 | 0 .547053458 | 3 .90059096 | 2 .23315 | 0 .03089 | 0 . 155875141 |
| TAB1 | -0 .281046239 | 7 . 1 13900478 | -2 .2328 | 0 .03092 | 0 . 155925298 |
| RANBP17 | 0 .596087084 | 5 .949386313 | 2 .23263 | 0 .03093 | 0 . 15593631 |
| ADM | 0 .585353797 | 7 .897015397 | 2 .23232 | 0 .03095 | 0 . 155988059 |
| ZNF204P | -0 .543060259 | 4 .404987852 | -2 .2311 | 0 .03104 | 0 . 15635416 |
| LZTFL1 | -0 .446199475 | 6 .250039596 | -2 .2305 | 0 .03108 | 0 . 156497664 |
| AMD1 | -0 .420742776 | 8 .930131962 | -2 .2292 | 0 .03118 | 0 . 156931288 |
| SSX3 | 0 .399424066 | 7 .338080484 | 2 .22892 | 0 .0312 | 0 . 156958115 |
| RBM26 | -0 .392080649 | 7 .828180394 | -2 .2286 | 0 .03122 | 0 . 157004384 |
| DCTN4 | -0 .311 135603 | 6 . 161066382 | -2 .2281 | 0 .03126 | 0 . 157055518 |
| AKR1C1 | -0 .574168188 | 9 .39045893 | -2 .228 | 0 .03126 | 0 . 157055518 |
| ATF6B | 0 .255458535 | 7 .312121344 | 2 .22795 | 0 .03127 | 0 . 157055518 |
| RHOD | 0 .28872582 | 9 .800502473 | 2 .22745 | 0 .0313 | 0 . 157172797 |
| PER2 | -0 .315069693 | 7 .969378267 | -2 .2271 | 0 .03133 | 0 . 157230235 |
| GSDMD | 0 .327955207 | 7 .0656706 | 2 .22617 | 0 .03139 | 0 . 157484783 |
| MYOC | -0 .717939892 | 5 .331321829 | -2 .2261 | 0 .0314 | 0 . 157484783 |
| NDUFAF3 | 0 .308443762 | 8 .790979518 | 2 .2256 | 0 .03144 | 0 . 157591942 |
| ATP13A1 | 0 .306281099 | 8 .316653905 | 2 .22525 | 0 .03146 | 0 . 157604747 |
| CORO7 | 0 .312108644 | 6 .676484193 | 2 .22507 | 0 .03147 | 0 . 157604747 |
| EIF2AK1 | 0 .2876882 | 9 .875327752 | 2 .22476 | 0 .0315 | 0 . 157604747 |
| RCE1 | 0 .338706449 | 7 .257333563 | 2 .22467 | 0 .0315 | 0 . 157604747 |
| ATG14 | -0 .29915372 | 7 .337683995 | -2 .2245 | 0 .03151 | 0 . 157604747 |
| TRIM33 | -0 .335979369 | 8 .829294092 | -2 .2245 | 0 .03151 | 0 . 157604747 |
| GSTM1 | -0 .467560451 | 9 .504257399 | -2 .223 | 0 .03162 | 0 . 158039851 |
| DKKL1 | 0 .639546888 | 4 .881303927 | 2 .22297 | 0 .03163 | 0 . 158039851 |
| FAIM | 0 .474356266 | 6 . 167371159 | 2 .22245 | 0 .03166 | 0 . 158115005 |
| ODC1 | 0 .441130817 | 8 . 152893317 | 2 .22241 | 0 .03167 | 0 . 158115005 |
| MAGOHB | 0 .679610296 | 5 .727544686 | 2 .22128 | 0 .03175 | 0 . 158463276 |
| EGLN1 | 0 .371153066 | 6 .772705088 | 2 .22097 | 0 .03177 | 0 . 158479956 |
| INPPL1 | 0 .39365909 | 8 .092057804 | 2 .22089 | 0 .03178 | 0 . 158479956 |
| GREB1 | 0 .493636975 | 7 .515866921 | 2 .22032 | 0 .03182 | 0 . 158622366 |

| TUBB6 | 0 .393366214 | 9 .836182107 | 2 .2196 | 0 .03187 | 0 . 158820208 |
| --- | --- | --- | --- | --- | --- |
| NDUFA10 | -0 .248641088 | 9 . 143221524 | -2 .2193 | 0 .0319 | 0 . 158882928 |
| ADCYAP1 | 0 .378423828 | 7 .371871455 | 2 .21859 | 0 .03194 | 0 . 159065154 |
| SLC48A1 | -0 .228149852 | 8 .394041273 | -2 .2181 | 0 .03198 | 0 . 15917335 |
| UPK3B | 0 .740479926 | 5 .583329474 | 2 .217 | 0 .03206 | 0 . 159504507 |
| PTDSS2 | 0 .505248979 | 5 .824300613 | 2 .21686 | 0 .03207 | 0 . 159504507 |
| MORF4L1 | -0 .372506359 | 10 .60417263 | -2 .2166 | 0 .03209 | 0 . 159533844 |
| MAD2L1BP | 0 .426329891 | 6 .943097496 | 2 .21618 | 0 .03212 | 0 . 159628526 |
| WDR62 | 0 .369499594 | 5 .517457622 | 2 .21531 | 0 .03219 | 0 . 159763345 |
| OSGIN1 | 0 .324936605 | 5 .940290826 | 2 .21528 | 0 .03219 | 0 . 159763345 |
| HNRNPA2B1 | 0 .350801457 | 10 . 1683381 | 2 .21516 | 0 .0322 | 0 . 159763345 |
| MICAL2 | 0 .370441237 | 9 .502682328 | 2 .21512 | 0 .0322 | 0 . 159763345 |
| PPP1R37 | 0 .342710298 | 6 .088673389 | 2 .2136 | 0 .03231 | 0 . 160256756 |
| CNGA3 | 0 .479445438 | 4 .283296153 | 2 .21319 | 0 .03234 | 0 . 160341913 |
| DCBLD2 | 0 .841968278 | 2 .757161628 | 2 .21196 | 0 .03243 | 0 . 160731386 |
| CXCL1 | 0 .417481003 | 6 .554682748 | 2 .21155 | 0 .03246 | 0 . 160752173 |
| PCOLCE2 | -0 .576927587 | 5 .556710047 | -2 .2115 | 0 .03247 | 0 . 160752173 |
| PKNOX2 | -0 .372540364 | 9 .529838706 | -2 .2114 | 0 .03248 | 0 . 160752173 |
| ARFGAP1 | 0 .294255853 | 7 .487065668 | 2 .2112 | 0 .03249 | 0 . 160756519 |
| PPDPF | -0 .464583248 | 8 .351094472 | -2 .2103 | 0 .03256 | 0 . 160933022 |
| MAGEH1 | -0 .348057646 | 7 . 128168488 | -2 .2103 | 0 .03256 | 0 . 160933022 |
| LOC257152 | 0 .474389353 | 5 .285997635 | 2 .2102 | 0 .03256 | 0 . 160933022 |
| MCM3 | 0 .319991045 | 8 . 155968169 | 2 .20963 | 0 .03261 | 0 . 161081374 |
| AGTR1 | -0 .247010107 | 7 .202809379 | -2 .2094 | 0 .03262 | 0 . 161100565 |
| MUT | -0 .409682099 | 7 .474264803 | -2 .2083 | 0 .0327 | 0 . 161408817 |
| PIK3C3 | -0 .216177529 | 7 .649331907 | -2 .2082 | 0 .03271 | 0 . 161408817 |
| FKBP1B | -0 .560367802 | 6 .066702255 | -2 .2065 | 0 .03284 | 0 . 161981482 |
| LPIN2 | 0 .245871443 | 8 .083140404 | 2 .20623 | 0 .03286 | 0 . 161991821 |
| TRIAP1 | -0 .264704711 | 7 .873091692 | -2 .2061 | 0 .03287 | 0 . 161991821 |
| ZNHIT6 | -0 .257029732 | 6 .961050172 | -2 .2011 | 0 .03325 | 0 . 163796986 |
| TMEM165 | 0 .415176816 | 6 .634979075 | 2 .20073 | 0 .03328 | 0 . 1638721 |
| RP3-507I15 . 1 | -0 .233481038 | 1 1 .41818119 | -2 .2003 | 0 .03331 | 0 . 163936898 |
| ABL1 | 0 .30268196 | 8 .825851447 | 2 .20021 | 0 .03332 | 0 . 163936898 |
| TIMP4 | -0 .548222601 | 5 . 167977852 | -2 . 1999 | 0 .03334 | 0 . 163989474 |
| LRIF1 | 0 .406814728 | 5 .704552607 | 2 . 19893 | 0 .03341 | 0 . 164287837 |
| AURKAIP1 | 0 .338841304 | 9 .431721402 | 2 . 1986 | 0 .03344 | 0 . 164347081 |
| GSTM2 | -0 .411047453 | 9 . 147775714 | -2 . 1979 | 0 .03349 | 0 . 164478988 |
| ANXA2 | 0 .335011605 | 13 .6817896 | 2 . 1979 | 0 .03349 | 0 . 164478988 |
| MILR1 | 0 .504336142 | 3 .791373477 | 2 . 19706 | 0 .03355 | 0 . 164729315 |
| PCOLCE | 0 .471954413 | 8 .744939417 | 2 . 19566 | 0 .03366 | 0 . 165190102 |
| OAZ1 | 0 .217031403 | 12 .28195077 | 2 . 19523 | 0 .0337 | 0 . 16528756 |
| TNMD | -0 .586725724 | 4 .410986744 | -2 . 1946 | 0 .03374 | 0 . 165465864 |
| IGF1 | -0 .339414076 | 8 .308015765 | -2 . 1941 | 0 .03378 | 0 . 165508949 |
| PPAP2B | -0 .598252078 | 10 .72107503 | -2 . 194 | 0 .03379 | 0 . 165508949 |
| RFX4 | 0 .581671367 | 2 .712449094 | 2 . 19396 | 0 .03379 | 0 . 165508949 |
| CTNNB1 | -0 .354783364 | 9 .804517888 | -2 . 1937 | 0 .03381 | 0 . 165548881 |
| SRSF11 | -0 .42384525 | 9 .9090596 | -2 . 1935 | 0 .03383 | 0 . 165548881 |
| PKP4 | -0 .214194738 | 9 .22900095 | -2 . 1932 | 0 .03385 | 0 . 165596523 |
| SULT1C2 | 0 .398906549 | 6 .756366392 | 2 . 19253 | 0 .0339 | 0 . 165787916 |
| APOL1 | 0 .594005434 | 6 .753777174 | 2 . 19213 | 0 .03393 | 0 . 165876478 |
| GTPBP4 | 0 .350051335 | 8 .622043164 | 2 . 19155 | 0 .03398 | 0 . 166030409 |
| ANXA3 | -0 .571567134 | 6 .354568243 | -2 . 1914 | 0 .03399 | 0 . 166036094 |
| PTPN1 | 0 .262430123 | 8 .592161414 | 2 . 19082 | 0 .03404 | 0 . 166177569 |
| WDR26 | 0 .359910771 | 8 .620675821 | 2 . 19048 | 0 .03406 | 0 . 166215935 |
| RSU1 | 0 .26299383 | 8 .884197222 | 2 . 19037 | 0 .03407 | 0 . 166215935 |
| CDC5L | 0 .259417334 | 9 .349278685 | 2 . 18938 | 0 .03415 | 0 . 166527008 |

| CLN8 | 0 .285948485 | 7 .601166545 | 2 . 18867 | 0 .0342 | 0 . 166731142 |
| --- | --- | --- | --- | --- | --- |
| ARFIP1 | -0 .322331665 | 7 .82086889 | -2 . 1879 | 0 .03426 | 0 . 166960101 |
| PJA1 | -0 .326315448 | 8 .038363726 | -2 . 1877 | 0 .03428 | 0 . 166971539 |
| RPL6 | -0 .272716701 | 1 1 .44284568 | -2 . 1871 | 0 .03432 | 0 . 167124782 |
| CDKN2AIP | -0 .388460725 | 6 .296403779 | -2 . 1866 | 0 .03436 | 0 . 167256055 |
| GRSF1 | -0 .294494631 | 9 . 1 10216711 | -2 . 1862 | 0 .0344 | 0 . 167348269 |
| DENND1C | 0 .357531071 | 7 .571864087 | 2 . 1852 | 0 .03447 | 0 . 167564852 |
| WDR47 | -0 .424162117 | 6 .760216991 | -2 . 1849 | 0 .0345 | 0 . 167564852 |
| NANS | 0 .264108584 | 8 .619636383 | 2 . 18485 | 0 .0345 | 0 . 167564852 |
| RRAGB | -0 .490623321 | 4 .215228304 | -2 . 1845 | 0 .03453 | 0 . 167564852 |
| OSTF1 | 0 .441645929 | 6 .666048004 | 2 . 1844 | 0 .03454 | 0 . 167564852 |
| SOSTDC1 | -1 .325819975 | 6 .371498551 | -2 . 1843 | 0 .03454 | 0 . 167564852 |
| FGF14 | -0 .571800385 | 4 .814650273 | -2 . 1843 | 0 .03454 | 0 . 167564852 |
| SNAPC1 | -0 .37970568 | 5 .898462428 | -2 . 1843 | 0 .03455 | 0 . 167564852 |
| EIF3M | -0 .280069958 | 10 .40826641 | -2 . 1838 | 0 .03458 | 0 . 167677949 |
| COPS4 | -0 .306772158 | 7 .792158039 | -2 . 1831 | 0 .03464 | 0 . 167838096 |
| NT5M | 0 .454918281 | 5 . 129643955 | 2 . 18303 | 0 .03464 | 0 . 167838096 |
| APBB2 | -0 .230156229 | 9 .291594129 | -2 . 1825 | 0 .03468 | 0 . 167963498 |
| DBP | -0 .290483087 | 7 .878276491 | -2 . 1818 | 0 .03474 | 0 . 168160936 |
| TNP1 | 0 .53366869 | 4 .839041587 | 2 . 18094 | 0 .03481 | 0 . 168354272 |
| ITCH | 0 .247841927 | 8 .201162364 | 2 . 18085 | 0 .03481 | 0 . 168354272 |
| POLDIP2 | 0 .232107022 | 8 .485257608 | 2 . 18072 | 0 .03483 | 0 . 168354272 |
| EMP2 | -0 .417780194 | 9 .254551828 | -2 . 1807 | 0 .03483 | 0 . 168354272 |
| CCL8 | 0 .829555625 | 5 . 184180282 | 2 . 18033 | 0 .03486 | 0 . 168416234 |
| OR3A3 | -0 .479966867 | 6 .805043115 | -2 . 1799 | 0 .03489 | 0 . 168506082 |
| LONRF1 | -0 .569704025 | 6 .928249222 | -2 . 1795 | 0 .03492 | 0 . 168587509 |
| RBX1 | -0 .473285624 | 7 .721227097 | -2 . 1791 | 0 .03495 | 0 . 16867345 |
| PRMT2 | -0 .307920942 | 9 .097207462 | -2 . 1785 | 0 .035 | 0 . 16878496 |
| GRAMD1C | -0 .347859987 | 4 .659147343 | -2 . 1785 | 0 .035 | 0 . 16878496 |
| RBM39 | -0 .380423999 | 9 .611945092 | -2 . 1776 | 0 .03507 | 0 . 169063884 |
| IQCK | -0 .236556923 | 8 .561896493 | -2 . 1773 | 0 .03509 | 0 . 16911 1472 |
| LPCAT1 | 0 .308988262 | 7 .788840909 | 2 . 17704 | 0 .03512 | 0 . 169156435 |
| ZNF331 | -0 .354878064 | 6 .969306203 | -2 . 1763 | 0 .03518 | 0 . 169384265 |
| ARHGAP28 | 0 .32227339 | 6 . 19893224 | 2 . 17454 | 0 .03532 | 0 . 169985486 |
| VWA5A | -0 .291103199 | 8 .333312507 | -2 . 1742 | 0 .03534 | 0 . 170033227 |
| FES | 0 .23746261 | 6 .867228178 | 2 . 17408 | 0 .03535 | 0 . 170033227 |
| AGO1 | -0 .218435652 | 7 .488830759 | -2 . 1723 | 0 .03549 | 0 . 170644658 |
| DPY19L1P1 | 0 .522775579 | 3 .735488818 | 2 . 172 | 0 .03552 | 0 . 170698887 |
| PSMD10 | -0 .40633551 | 7 .397739245 | -2 . 1717 | 0 .03554 | 0 . 170698887 |
| GLYR1 | 0 .209779669 | 8 .50599245 | 2 . 17167 | 0 .03555 | 0 . 170698887 |
| SLC15A3 | 0 .506527281 | 7 . 1 168769 | 2 . 17131 | 0 .03558 | 0 . 170747719 |
| SRC | 0 .317276896 | 6 .801941036 | 2 . 17119 | 0 .03559 | 0 . 170747719 |
| TTC33 | -0 .379094486 | 4 .883927314 | -2 . 171 | 0 .0356 | 0 . 170747719 |
| BORA | 0 .288385732 | 5 .751209525 | 2 . 17078 | 0 .03562 | 0 . 170766979 |
| LPAR6 | -0 .491210228 | 7 .851648215 | -2 . 1707 | 0 .03563 | 0 . 170766979 |
| SLPI | 0 .669248421 | 8 .535874614 | 2 . 16982 | 0 .0357 | 0 . 170931866 |
| VAMP2 | -0 .219289013 | 9 .787811974 | -2 . 1698 | 0 .0357 | 0 . 170931866 |
| TBL3 | 0 .312440007 | 7 . 162901321 | 2 . 16972 | 0 .0357 | 0 . 170931866 |
| CAPN9 | 0 .276832596 | 7 .226596294 | 2 . 16886 | 0 .03577 | 0 . 171197689 |
| ICT1 | 0 .381313571 | 6 .864566566 | 2 . 16833 | 0 .03582 | 0 . 171290167 |
| PRDX2 | -0 .390592726 | 10 .06369656 | -2 . 1682 | 0 .03582 | 0 . 171290167 |
| LINC00312 | -0 .312455975 | 6 .828560652 | -2 . 1681 | 0 .03583 | 0 . 171290167 |
| KIF5B | -0 .221583124 | 10 .00401606 | -2 . 1671 | 0 .03591 | 0 . 171610464 |
| ITPKB | 0 .489317431 | 8 .506436098 | 2 . 16635 | 0 .03598 | 0 . 171844447 |
| PNPLA3 | -0 .440411976 | 5 .660268352 | -2 . 166 | 0 .036 | 0 . 171902315 |
| LXN | -0 .452441958 | 5 .997035909 | -2 . 1649 | 0 .03609 | 0 . 172215269 |

| CA6 | -1 .016860371 | 6 .360720237 | -2 . 1649 | 0 .0361 | 0 . 172215269 |
| --- | --- | --- | --- | --- | --- |
| MTMR2 | -0 .286067252 | 8 .983991241 | -2 . 1643 | 0 .03615 | 0 . 172396654 |
| THAP10 | -0 .381656724 | 6 .276367946 | -2 . 164 | 0 .03617 | 0 . 172431074 |
| EHD4 | 0 .327234415 | 8 .642304211 | 2 . 16373 | 0 .03619 | 0 . 172472526 |
| RNF126 | 0 .278596104 | 7 .995906335 | 2 . 16323 | 0 .03623 | 0 . 172602769 |
| CCL7 | 0 .49556735 | 5 .786859534 | 2 . 16245 | 0 .0363 | 0 . 172839328 |
| UCP2 | 0 .721103694 | 9 .91321023 | 2 . 1613 | 0 .03639 | 0 . 173222119 |
| CCNG1 | -0 .523081192 | 8 .757438768 | -2 . 1609 | 0 .03642 | 0 . 173270413 |
| HMGCS2 | -0 .849119593 | 5 .535866362 | -2 . 1607 | 0 .03644 | 0 . 173270413 |
| SGMS1 | -0 .515337283 | 5 .889198899 | -2 . 1605 | 0 .03645 | 0 . 173270413 |
| GIF | 0 .619032047 | 3 .928643258 | 2 . 16051 | 0 .03645 | 0 . 173270413 |
| GSTK1 | 0 .334280561 | 9 .836627476 | 2 . 15997 | 0 .0365 | 0 . 173306464 |
| STX8 | -0 .312680652 | 7 .596312319 | -2 . 1598 | 0 .03651 | 0 . 173306464 |
| ARC | 0 .566607412 | 4 .755494328 | 2 . 15978 | 0 .03651 | 0 . 173306464 |
| YIPF3 | 0 .239283439 | 8 .629508305 | 2 . 15975 | 0 .03652 | 0 . 173306464 |
| CD101 | 0 .595743551 | 6 .015441332 | 2 . 15904 | 0 .03658 | 0 . 173516256 |
| QSOX1 | 0 .351764929 | 8 .087698965 | 2 . 15803 | 0 .03666 | 0 . 173847509 |
| GART | 0 .266431272 | 8 .754312836 | 2 . 1577 | 0 .03669 | 0 . 173895269 |
| RFX7 | 0 .446357603 | 5 .923675425 | 2 . 15757 | 0 .0367 | 0 . 173895269 |
| YARS | 0 .245699017 | 8 .519426018 | 2 . 15703 | 0 .03674 | 0 . 173968744 |
| TNFRSF10B | 0 .225468879 | 8 .558904771 | 2 . 15702 | 0 .03674 | 0 . 173968744 |
| DNAJC3 | 0 .471189955 | 7 . 1 12252193 | 2 . 15674 | 0 .03677 | 0 . 173968744 |
| FBP1 | -0 .312820897 | 7 .739018329 | -2 . 1566 | 0 .03678 | 0 . 173968744 |
| ZNF430 | -0 .250052029 | 6 .837975813 | -2 . 1565 | 0 .03678 | 0 . 173968744 |
| EPB41L3 | 0 .472959373 | 8 . 1955182 | 2 . 15605 | 0 .03682 | 0 . 17409733 |
| ZNF408 | 0 .32736817 | 6 .276398273 | 2 . 15532 | 0 .03688 | 0 . 174319411 |
| TMEM177 | 0 .324311375 | 6 .840388311 | 2 . 1549 | 0 .03692 | 0 . 17442025 |
| CCNO | 0 .372298749 | 5 .578364002 | 2 . 15459 | 0 .03694 | 0 . 174475435 |
| DHX9 | 0 .347566251 | 8 .349502396 | 2 . 15419 | 0 .03698 | 0 . 17456833 |
| PLEKHG6 | -0 .364553491 | 6 . 126104523 | -2 . 1537 | 0 .03702 | 0 . 174570258 |
| AVPI1 | 0 .309009736 | 8 .533347204 | 2 . 1537 | 0 .03702 | 0 . 174570258 |
| GCC1 | 0 .203081159 | 8 . 159984672 | 2 . 15368 | 0 .03702 | 0 . 174570258 |
| S100PBP | -0 .304285577 | 5 .78495327 | -2 . 153 | 0 .03708 | 0 . 174769341 |
| PTDSS1 | 0 .274454374 | 9 .577778606 | 2 . 15242 | 0 .03713 | 0 . 174934422 |
| RAB2A | 0 .221527662 | 10 .61377368 | 2 . 15189 | 0 .03717 | 0 . 175079223 |
| EIF3K | -0 .235343829 | 12 .00156997 | -2 . 1516 | 0 .03719 | 0 . 175121243 |
| AGGF1 | 0 .220306172 | 8 .238618583 | 2 . 15131 | 0 .03722 | 0 . 175167808 |
| MAP4K1 | 0 .647203612 | 7 .793545878 | 2 . 15116 | 0 .03723 | 0 . 175167808 |
| MTSS1L | -0 .44141739 | 4 .331920372 | -2 . 1502 | 0 .03731 | 0 . 175404368 |
| EMX2 | -0 .486613444 | 5 .391581181 | -2 . 1502 | 0 .03732 | 0 . 175404368 |
| HS3ST1 | -0 .484164169 | 6 .281641812 | -2 . 15 | 0 .03733 | 0 . 175404368 |
| COA1 | 0 .214352071 | 7 .232740116 | 2 . 1499 | 0 .03734 | 0 . 175404368 |
| TARS | 0 .433561879 | 7 .816002607 | 2 . 14915 | 0 .0374 | 0 . 175634643 |
| AKR1C4 | -0 .433798401 | 4 .771721009 | -2 . 1485 | 0 .03746 | 0 . 175730276 |
| SPINT3 | 0 .412247677 | 4 .758666181 | 2 . 14831 | 0 .03747 | 0 . 175730276 |
| HMHA1 | 0 .405705047 | 8 . 151850984 | 2 . 14822 | 0 .03748 | 0 . 175730276 |
| MLXIPL | 0 .355438998 | 5 .498527456 | 2 . 14801 | 0 .0375 | 0 . 175730276 |
| GPR116 | -0 .265886006 | 7 .912205455 | -2 . 1477 | 0 .03752 | 0 . 175730276 |
| GNA11 | -0 .229521595 | 10 .85956525 | -2 . 1476 | 0 .03753 | 0 . 175730276 |
| HSPB6 | -0 .396506024 | 6 .550473544 | -2 . 1476 | 0 .03753 | 0 . 175730276 |
| GNG12 | -0 .344584779 | 9 .715052727 | -2 . 1475 | 0 .03753 | 0 . 175730276 |
| OLA1 | -0 .324789321 | 9 .080896525 | -2 . 1474 | 0 .03755 | 0 . 175730276 |
| BCL11B | -0 .389562574 | 7 . 1 12311745 | -2 . 1472 | 0 .03756 | 0 . 175730276 |
| PUS3 | -0 .216614733 | 7 .624644415 | -2 . 1469 | 0 .03759 | 0 . 175803567 |
| KIAA1033 | -0 .338566553 | 9 .348055827 | -2 . 1454 | 0 .03772 | 0 . 176332231 |
| GSTT1 | 1 .315867069 | 6 .469782554 | 2 . 14412 | 0 .03782 | 0 . 176700945 |

| CPSF6 | 0 .240638739 | 7 .844098654 | 2 . 14406 | 0 .03783 | 0 . 176700945 |
| --- | --- | --- | --- | --- | --- |
| FOXF2 | 0 .478639682 | 6 .496146828 | 2 . 14379 | 0 .03785 | 0 . 176700945 |
| IGLL5 | 1 . 122270822 | 7 .844797756 | 2 . 14368 | 0 .03786 | 0 . 176700945 |
| SELE | 0 .366741944 | 6 .335044723 | 2 . 14363 | 0 .03787 | 0 . 176700945 |
| ATXN1 | -0 .346418834 | 7 .911605458 | -2 . 143 | 0 .03792 | 0 . 176876605 |
| ASF1B | 0 .278848904 | 7 .241428704 | 2 . 14286 | 0 .03793 | 0 . 176876605 |
| KLC2 | 0 .51227175 | 6 .858061413 | 2 . 14226 | 0 .03798 | 0 . 177047989 |
| RPAP1 | -0 .250055453 | 7 .38058391 | -2 . 1415 | 0 .03805 | 0 . 177220368 |
| TOM1L2 | -0 .375975157 | 5 .407296547 | -2 . 1415 | 0 .03805 | 0 . 177220368 |
| ATP10B | 0 .388004068 | 8 .337390727 | 2 . 14082 | 0 .03811 | 0 . 177424909 |
| PPP1CC | -0 .336326671 | 9 . 1 16590253 | -2 . 1399 | 0 .03819 | 0 . 177733314 |
| HAND2-AS1 | 0 .786539633 | 2 .250997573 | 2 . 13921 | 0 .03824 | 0 . 177933824 |
| ZNF271 | -0 .464089695 | 6 .646864331 | -2 . 1389 | 0 .03827 | 0 . 177977842 |
| HDAC5 | -0 .270614866 | 7 .608671888 | -2 . 1374 | 0 .0384 | 0 . 178473506 |
| POP4 | 0 .269534959 | 7 .607587561 | 2 . 13737 | 0 .0384 | 0 . 178473506 |
| PPP2R3A | -0 .38286259 | 8 .333275939 | -2 . 136 | 0 .03852 | 0 . 178965436 |
| OLFML3 | -0 .42943656 | 7 .456816052 | -2 . 1355 | 0 .03857 | 0 . 179047427 |
| PEX11B | -0 .253389441 | 7 .386475969 | -2 . 1354 | 0 .03857 | 0 . 179047427 |
| PDCD5 | 0 .346099074 | 6 .619829963 | 2 . 13493 | 0 .03861 | 0 . 179186964 |
| CD244 | 0 .485896901 | 5 .08128172 | 2 . 13468 | 0 .03864 | 0 . 179221785 |
| DYNC2H1 | -0 .363533094 | 6 .021352135 | -2 . 1344 | 0 .03866 | 0 . 179258701 |
| AKAP6 | -0 .43444631 | 6 . 178260454 | -2 . 1336 | 0 .03873 | 0 . 179457913 |
| REG1A | 0 .503132894 | 5 .888318108 | 2 . 13359 | 0 .03873 | 0 . 179457913 |
| FCER1G | 0 .609724276 | 8 .593235258 | 2 . 13197 | 0 .03887 | 0 . 180047046 |
| GLRX2 | 0 .442056147 | 7 .586316172 | 2 . 13174 | 0 .03889 | 0 . 180070536 |
| CCDC28A | -0 .206727489 | 7 .624048765 | -2 . 1313 | 0 .03893 | 0 . 180170453 |
| STARD13 | -0 .524941766 | 6 .856856657 | -2 . 1312 | 0 .03894 | 0 . 180170453 |
| B4GALT2 | 0 .306804783 | 7 .983269641 | 2 . 13057 | 0 .03899 | 0 . 1803433 |
| ASNSD1 | -0 .343826712 | 7 .294216859 | -2 . 1292 | 0 .03911 | 0 . 180822539 |
| SNORA21 | -0 .501735509 | 5 .299359055 | -2 . 1286 | 0 .03916 | 0 . 181002597 |
| PRRC2B | -0 .335286875 | 9 .231571756 | -2 . 1282 | 0 .0392 | 0 . 181005716 |
| BEX4 | -0 .433684288 | 7 .578150137 | -2 . 128 | 0 .03922 | 0 . 181005716 |
| DNMT3B | 0 .36670798 | 6 .894612436 | 2 . 12797 | 0 .03922 | 0 . 181005716 |
| ATP2A1 | 0 .340364026 | 5 .340935026 | 2 . 12782 | 0 .03923 | 0 . 181005716 |
| YWHAZ | 0 .27860227 | 12 .36474878 | 2 . 12773 | 0 .03924 | 0 . 181005716 |
| TBC1D9B | 0 . 183337003 | 1 1 .04723442 | 2 . 12762 | 0 .03925 | 0 . 181005716 |
| MANF | 0 .424161603 | 8 .434593621 | 2 . 12707 | 0 .0393 | 0 . 181160347 |
| SLC25A36 | -0 .2296583 | 10 .5112993 | -2 . 1268 | 0 .03932 | 0 . 181185693 |
| UROS | 0 .25225502 | 6 .937296392 | 2 . 12609 | 0 .03939 | 0 . 181384725 |
| LOR | 1 .672610261 | 9 .872962222 | 2 . 12603 | 0 .03939 | 0 . 181384725 |
| ACTA1 | -0 .805120596 | 4 .364558566 | -2 . 1255 | 0 .03944 | 0 . 18153666 |
| RNFT2 | 0 .356150985 | 6 .365283492 | 2 . 12518 | 0 .03947 | 0 . 181547501 |
| UGCG | -0 .506435519 | 9 .947442174 | -2 . 1251 | 0 .03947 | 0 . 181547501 |
| NKG7 | 0 .977563031 | 6 .333639671 | 2 . 12398 | 0 .03957 | 0 . 181950974 |
| ESR2 | -0 .28223998 | 7 .515116395 | -2 . 1238 | 0 .03959 | 0 . 181951659 |
| ISG15 | 0 .968275458 | 8 .438614864 | 2 . 12253 | 0 .0397 | 0 . 182408286 |
| TRIL | -0 .279957588 | 7 .716018005 | -2 . 1221 | 0 .03974 | 0 . 182530482 |
| CERS2 | 0 .336409351 | 8 .845234999 | 2 . 12134 | 0 .03981 | 0 . 182757343 |
| DDX41 | 0 .300604326 | 8 .261822981 | 2 . 12057 | 0 .03987 | 0 . 18293916 |
| FAM155B | 0 .593702775 | 5 .348833943 | 2 . 12057 | 0 .03987 | 0 . 18293916 |
| MPI | 0 .300137705 | 6 . 170857833 | 2 . 1 1994 | 0 .03993 | 0 . 183102824 |
| POU3F2 | 0 .605662101 | 4 .347627396 | 2 . 1 1972 | 0 .03995 | 0 . 183102824 |
| PSMD4 | 0 .237588286 | 10 .48868328 | 2 . 1 1968 | 0 .03995 | 0 . 183102824 |
| PRKACB | -0 .444289604 | 8 .342307422 | -2 . 1 187 | 0 .04004 | 0 . 183412323 |
| TGS1 | 0 .469322346 | 6 .877617789 | 2 . 1 1859 | 0 .04005 | 0 . 183412323 |
| ADAR | 0 .24742449 | 10 .0189254 | 2 . 1 1746 | 0 .04015 | 0 . 183788447 |

| CRTAC1 | -1 .064059338 | 8 .259319404 | -2 . 1 173 | 0 .04017 | 0 . 183788447 |
| --- | --- | --- | --- | --- | --- |
| RFC5 | 0 .32224532 | 7 .934922523 | 2 . 1 1718 | 0 .04018 | 0 . 183788447 |
| FBXO7 | -0 .444075774 | 10 . 13565199 | -2 . 1 168 | 0 .04021 | 0 . 183828611 |
| CEACAM1 | -0 .290723479 | 9 .206835639 | -2 . 1 168 | 0 .04021 | 0 . 183828611 |
| TEX12 | 0 .479326319 | 3 .468169045 | 2 . 1 16 | 0 .04028 | 0 . 184072714 |
| PRKCZ | -0 .329330228 | 7 .776258159 | -2 . 1 146 | 0 .04041 | 0 . 184584349 |
| FAM50A | 0 .4707788 | 8 .266156117 | 2 . 1 1394 | 0 .04047 | 0 . 184784018 |
| FAU | -0 .279359742 | 1 1 . 16317072 | -2 . 1 138 | 0 .04048 | 0 . 184787887 |
| RAB29 | 0 .404598525 | 7 .96281016 | 2 . 1 1356 | 0 .0405 | 0 . 184806393 |
| ANGPTL7 | -1 . 12228355 | 6 . 158152066 | -2 . 1 129 | 0 .04056 | 0 . 184987294 |
| IER5 | 0 .317605084 | 8 .59480513 | 2 . 1 1278 | 0 .04057 | 0 . 184987294 |
| ZNF134 | -0 .279804362 | 6 . 153506817 | -2 . 1 124 | 0 .0406 | 0 . 184987294 |
| CACNG3 | 0 .645174008 | 3 .880165417 | 2 . 1 1231 | 0 .04062 | 0 . 184987294 |
| RPS18 | -0 .267112966 | 12 .45531727 | -2 . 1 123 | 0 .04062 | 0 . 184987294 |
| HSD17B7 | -0 .379049983 | 8 .243282763 | -2 . 1 1 18 | 0 .04066 | 0 . 185058336 |
| SPTBN4 | -0 .603822487 | 4 .745484875 | -2 . 1 1 18 | 0 .04067 | 0 . 185058336 |
| APOO | -0 .570683999 | 8 .346481679 | -2 . 1 1 16 | 0 .04068 | 0 . 185058336 |
| ADARB2 | -0 .518510826 | 4 .433970373 | -2 . 1 1 1 1 | 0 .04073 | 0 . 185227168 |
| ATP6V1C1 | 0 .367955833 | 8 .241090393 | 2 . 1 1003 | 0 .04082 | 0 . 18559235 |
| SLIT2 | -0 .334945382 | 7 .351793652 | -2 . 1096 | 0 .04086 | 0 . 185701045 |
| NDUFB6 | -0 .373325845 | 7 .512998221 | -2 . 1088 | 0 .04094 | 0 . 185971824 |
| SF1 | -0 .239231417 | 9 .890624511 | -2 . 1086 | 0 .04095 | 0 . 185971824 |
| RP1-8B22 . 1 | 0 .492345622 | 6 .076868073 | 2 . 10811 | 0 .041 | 0 . 186115044 |
| ACSM3 | -0 .399477384 | 6 .047889998 | -2 . 1075 | 0 .04105 | 0 . 186225634 |
| KLHL29 | -0 .506906742 | 4 .661057011 | -2 . 1075 | 0 .04105 | 0 . 186225634 |
| TRIM2 | -0 .572031543 | 9 .966976692 | -2 . 1074 | 0 .04107 | 0 . 186225634 |
| SLC6A6 | 0 .448216562 | 4 .735908664 | 2 . 107 | 0 .0411 | 0 . 186307916 |
| GINS4 | 0 .733219922 | 4 .702268289 | 2 . 10624 | 0 .04117 | 0 . 186463071 |
| SPDYE2 | -0 .46932271 | 5 .914317902 | -2 . 1062 | 0 .04117 | 0 . 186463071 |
| STAMBP | 0 .322840188 | 7 .639043899 | 2 . 10614 | 0 .04118 | 0 . 186463071 |
| SSH1 | 0 .213016595 | 9 . 1 17897497 | 2 . 10518 | 0 .04126 | 0 . 186790908 |
| MBOAT2 | -0 .249420239 | 7 . 129663155 | -2 . 1046 | 0 .04132 | 0 . 186973433 |
| HNRNPC | -0 .251481435 | 1 1 .2306745 | -2 . 1031 | 0 .04145 | 0 . 187517641 |
| C19orf53 | 0 .224391312 | 9 .647314138 | 2 . 1022 | 0 .04154 | 0 . 187757021 |
| AKAP10 | 0 .276216402 | 7 .263359749 | 2 . 10209 | 0 .04155 | 0 . 187757021 |
| CCDC91 | -0 .570760393 | 6 . 139621057 | -2 . 1019 | 0 .04157 | 0 . 187757021 |
| TMEM243 | -0 .337672324 | 7 .406499738 | -2 . 1019 | 0 .04157 | 0 . 187757021 |
| ZER1 | -0 .21979599 | 7 .999676451 | -2 . 1016 | 0 .04159 | 0 . 187757021 |
| TMEM14B | -0 .444691301 | 7 .792087078 | -2 . 1016 | 0 .0416 | 0 . 187757021 |
| NDFIP1 | -0 .316290519 | 8 .665010358 | -2 . 1 | 0 .04174 | 0 . 18832875 |
| SLC19A1 | 0 .359920439 | 4 .706813237 | 2 .09987 | 0 .04175 | 0 . 18832875 |
| POGLUT1 | -0 .342438516 | 6 . 145074998 | -2 .0992 | 0 .04181 | 0 . 188527203 |
| GK2 | -0 .448173499 | 5 .880762208 | -2 .099 | 0 .04184 | 0 . 188559011 |
| TAPBP | 0 .33224564 | 9 .979074706 | 2 .09842 | 0 .04189 | 0 . 188734902 |
| CDS2 | 0 .270417777 | 7 .939966437 | 2 .09814 | 0 .04192 | 0 . 188785117 |
| SIX2 | 0 .410129214 | 7 .294284389 | 2 .0974 | 0 .04198 | 0 . 188929409 |
| CTSC | 0 .58636497 | 9 .096308363 | 2 .09736 | 0 .04199 | 0 . 188929409 |
| RPRM | 0 .309211654 | 5 .627779475 | 2 .09731 | 0 .04199 | 0 . 188929409 |
| LTB | 1 .079921536 | 7 .517393642 | 2 .09639 | 0 .04208 | 0 . 189162872 |
| RNH1 | 0 .239519665 | 10 .92542805 | 2 .09637 | 0 .04208 | 0 . 189162872 |
| IL21 | -0 .34668771 | 6 .56305326 | -2 .0961 | 0 .0421 | 0 . 189162872 |
| ASIC2 | 0 .37913641 | 5 . 125375211 | 2 .0961 | 0 .0421 | 0 . 189162872 |
| ADAMTSL3 | 0 .273041636 | 6 .453480303 | 2 .09591 | 0 .04212 | 0 . 189176024 |
| MNAT1 | -0 .230667552 | 5 .446651359 | -2 .095 | 0 .04221 | 0 . 189484751 |
| LAPTM4B | 0 .510652334 | 1 1 . 18525231 | 2 .09431 | 0 .04227 | 0 . 189680303 |
| C17orf70 | 0 .235328842 | 7 .341423588 | 2 .09423 | 0 .04228 | 0 . 189680303 |

| ABCA6 | -0 .710750023 | 4 .645396316 | -2 .0937 | 0 .04233 | 0 . 189847718 |
| --- | --- | --- | --- | --- | --- |
| DPH5 | -0 .340879294 | 8 .702061698 | -2 .0934 | 0 .04236 | 0 . 189847718 |
| ACADL | -0 .371843207 | 6 . 150474115 | -2 .0933 | 0 .04236 | 0 . 189847718 |
| CLDN4 | -0 .601586561 | 4 .846774661 | -2 .0922 | 0 .04247 | 0 . 190277396 |
| NMT2 | -0 .23996571 | 7 .780936879 | -2 .092 | 0 .04249 | 0 . 190294973 |
| NOV | 0 .58373706 | 9 .390775452 | 2 .0918 | 0 .04251 | 0 . 190294973 |
| ABCB6 | 0 .283543529 | 6 .731186753 | 2 .09148 | 0 .04254 | 0 . 190363314 |
| GNG4 | 0 .442122786 | 6 .238099642 | 2 .09115 | 0 .04257 | 0 . 19043293 |
| PPP2CA | 0 .263667123 | 8 .519128701 | 2 .09064 | 0 .04262 | 0 . 190581381 |
| TAP2 | 0 .413257125 | 8 .794445451 | 2 .09005 | 0 .04267 | 0 . 190736881 |
| NPAS2 | 0 .246336756 | 8 .332668382 | 2 .08995 | 0 .04268 | 0 . 190736881 |
| GPD1 | -0 .491660529 | 6 .75500672 | -2 .0897 | 0 .04271 | 0 . 190774004 |
| AAAS | 0 .213695266 | 7 .487255984 | 2 .08952 | 0 .04272 | 0 . 190774004 |
| LARP1 | 0 .222068339 | 1 1 .09344721 | 2 .08938 | 0 .04274 | 0 . 190774004 |
| DOCK2 | 0 .787380456 | 5 .66046654 | 2 .08919 | 0 .04275 | 0 . 190783006 |
| RPS12 | -0 .260553836 | 12 .2066748 | -2 .0886 | 0 .04281 | 0 . 190944443 |
| GBP1 | 0 .720164648 | 9 . 181351712 | 2 .08849 | 0 .04282 | 0 . 190944443 |
| IRF1 | 0 .612597016 | 7 .982198448 | 2 .08746 | 0 .04292 | 0 . 191314306 |
| LEPREL4 | -0 .473508656 | 6 .595628963 | -2 .0864 | 0 .04302 | 0 . 191700095 |
| CD4 | 0 .675159748 | 8 .551222408 | 2 .08513 | 0 .04314 | 0 . 192106776 |
| PSTPIP1 | 0 .389016605 | 6 .051406997 | 2 .08511 | 0 .04314 | 0 . 192106776 |
| ZNF665 | -0 .459240413 | 9 .365602637 | -2 .0842 | 0 .04323 | 0 . 192406272 |
| MLN | 0 .427471226 | 7 .28237753 | 2 .08394 | 0 .04325 | 0 . 192458383 |
| CBY1 | -0 .322529344 | 7 .965431592 | -2 .0838 | 0 .04327 | 0 . 192458383 |
| CLASP2 | -0 .548275253 | 7 .923351409 | -2 .0833 | 0 .04331 | 0 . 19258767 |
| GLUD2 | -0 .32255499 | 7 .310424066 | -2 .0831 | 0 .04334 | 0 . 192633108 |
| C11orf57 | -0 .231175746 | 7 .523272088 | -2 .0818 | 0 .04346 | 0 . 193054819 |
| PDCD6IP | 0 .242233877 | 9 .200143143 | 2 .08176 | 0 .04346 | 0 . 193054819 |
| VPREB3 | -0 .329416119 | 5 .7311 12571 | -2 .0813 | 0 .04351 | 0 . 193126343 |
| EML1 | 0 .286005859 | 6 . 123413011 | 2 .08127 | 0 .04351 | 0 . 193126343 |
| SLC25A6 | -0 .23222068 | 12 .24881102 | -2 .0809 | 0 .04355 | 0 . 193192958 |
| PELP1 | 0 .320476284 | 6 .658795325 | 2 .08078 | 0 .04356 | 0 . 193192958 |
| LPL | -0 .573444482 | 8 .686206429 | -2 .0804 | 0 .0436 | 0 . 193192958 |
| P2RY4 | 0 .339628021 | 5 .7238648 | 2 .08012 | 0 .04362 | 0 . 193192958 |
| ATG12 | -0 .226606262 | 7 .94350192 | -2 .0801 | 0 .04363 | 0 . 193192958 |
| TRIB2 | 0 .647299143 | 9 .72146261 | 2 .08001 | 0 .04363 | 0 . 193192958 |
| CS | -0 .241569737 | 10 .02999218 | -2 .0799 | 0 .04364 | 0 . 193192958 |
| TRAM2 | 0 .229865164 | 8 .968220013 | 2 .07983 | 0 .04365 | 0 . 193192958 |
| LEF1 | 0 .465522847 | 9 . 161770069 | 2 .07921 | 0 .04371 | 0 . 193347322 |
| WWP2 | -0 . 19790828 | 8 . 1 18195518 | -2 .0792 | 0 .04371 | 0 . 193347322 |
| WLS | -0 .435648632 | 7 .760843875 | -2 .0786 | 0 .04377 | 0 . 193532116 |
| MPZL2 | 0 .550539228 | 6 . 191772629 | 2 .07799 | 0 .04383 | 0 . 193598499 |
| RNF8 | 0 .239235851 | 7 .494165803 | 2 .07795 | 0 .04383 | 0 . 193598499 |
| C1QTNF1 | 0 .344447583 | 8 .052605406 | 2 .07788 | 0 .04384 | 0 . 193598499 |
| ABCC9 | -0 .301925173 | 7 .027536687 | -2 .0778 | 0 .04385 | 0 . 193598499 |
| PDLIM7 | 0 .270833383 | 8 .441563458 | 2 .07758 | 0 .04387 | 0 . 19360899 |
| CYB5A | -0 .558225602 | 10 .20706408 | -2 .0765 | 0 .04397 | 0 . 193955259 |
| TMEM97 | -0 .493338848 | 10 .27582716 | -2 .0764 | 0 .04398 | 0 . 193955259 |
| WDR5B | -0 .302010211 | 6 .913232137 | -2 .0763 | 0 .04399 | 0 . 193955259 |
| KLF12 | 0 .274576185 | 7 .064640684 | 2 .07612 | 0 .04401 | 0 . 19395855 |
| SH3GLB2 | -0 .303793117 | 6 .915409477 | -2 .0757 | 0 .04405 | 0 . 194080967 |
| TUBB4B | 0 .310525435 | 1 1 .84332752 | 2 .07505 | 0 .04411 | 0 . 194281606 |
| MAPK7 | 0 .231633234 | 7 .579766483 | 2 .07473 | 0 .04414 | 0 . 194351966 |
| PDS5B | -0 .290262599 | 7 .343054453 | -2 .0734 | 0 .04427 | 0 . 194860993 |
| NME4 | 0 .340513675 | 8 .700757583 | 2 .07297 | 0 .04431 | 0 . 194968406 |
| QPCTL | 0 .617858165 | 4 .019992012 | 2 .07255 | 0 .04436 | 0 . 19508312 |

0 .338687188 0 .715521696 0 .382165353 0 .348043667 -0 .222643734 -0 .296093395 0 .289772992 0 .517464943 -0 .345377276 -0 .598575084 -0 .383933271 0 .308613538 -0 .278495215 0 .631949126 0 .432979147 0 .511330591

CCT5 KCNG1 PKN1 ATP6V0D1 NFYB ZNF235 NARFL GCM2 UBE2W SLC44A1 FOCAD MPV17 KIF1B SLC39A2 STAG3 KRT76

LOC100506469 -0 .367436801

SIL1

0 .367998512 -0 .427044941 0 .335165939 -0 .281216033 0 .22631475 -0 .453886596 -0 .351015534 -0 .272397377 0 .720378352 -0 .347602392 0 .719027813 -0 .306983582 -0 .621333919 -0 .29514522 0 .52613997 -0 . 197936607 -0 .281709627 -0 .225829903 -0 .501005069 -0 .481007613 -0 .389270265 0 .622903641 -0 .701555473 0 .486300812 -0 . 183182861 0 .320557753 0 .464595816 0 .368756783 0 .278760379 -0 .606556841 -0 .314308701 0 .558539569 -0 .310153335 0 .264270886 -0 .410037395 0 .334902642 -0 .445830059 0 .480333198 0 .330999997

TTC37

NCLN

INPP1

PRPF31

FAM124B

FZD3

RBM5

HTR5A

DDX17

APLP1

MGST2

GLDC

MMD

NRAS

SON

MAP3K2

NFX1

ZNF721

TMEM38B

WISP3

NKX2-1

MYH8

STMN3

ADD1

RTEL1

PDK1

FLT3

NOP10

ATP2A3

SNX7

SELPLG

BCKDHB

LANCL2

MFSD7

TCEB3-AS1

PPWD1

RASSF8

PAX2

10 .02192093 4 .046138263 7 .384779895 9 .668245772 7 .07253796 5 .998201546 6 .923207004 3 .353669372 5 .982777213 4 .355263926 6 .261889946 8 . 17644229 7 .034004567 6 .298367627 5 .011915509 3 .49101528 7 .018826711 7 .432366714 8 .086751109 7 .561416901 8 . 190900358 8 .880682713 4 . 140657576 5 .769845116 9 .234117319 3 .318608499 1 1 .00211316 3 .216763921 8 .439414938 6 .463360985 6 .501916159 5 .670709091 10 .46915839 5 .715545906 7 .500591043 8 .758435052 4 .833244847 5 .541371085 5 .0675975 5 .952619865 4 .060134796 10 .27744869 8 .476174745 4 .443204018 6 .019921017 9 .397188761 7 .057213894 8 .253934831 7 .706104137 6 .244466172 7 .407883238 7 .587495705 5 .601515769 6 .004019333 4 .528662579 6 . 140373581

2 .0715 2 .07106 2 .07086 2 .07067 -2 .0704 -2 .0701 2 .06975 2 .06792 -2 .0679 -2 .0675 -2 .0673 2 .06679 -2 .066 2 .06538 2 .06499 2 .06436 -2 .0643 2 .06404 -2 .0637 2 .06341 -2 .0625 2 .06231 -2 .0621 -2 .0621 -2 .0617 2 .06147 -2 .0614 2 .0609 -2 .0607 -2 .0607 -2 .06 2 .05965 -2 .059 -2 .059 -2 .0589 -2 .0585 -2 .0578 -2 .0578 2 .0573 -2 .0569 2 .05642 -2 .0564 2 .05618 2 .05582 2 .05572 2 .05446 -2 .054 -2 .054 2 .05384 -2 .0537 2 .05368 -2 .0532 2 .05306 -2 .0525 2 .05234 2 .05211

0 .04446 0 .0445 0 .04452 0 .04454 0 .04457 0 .04459 0 .04463 0 .04481 0 .04482 0 .04485 0 .04487 0 .04492 0 .045 0 .04506 0 .0451 0 .04516 0 .04517 0 .04519 0 .04523 0 .04526 0 .04535 0 .04537 0 .04538 0 .04539 0 .04542 0 .04545 0 .04546 0 .04551 0 .04553 0 .04553 0 .0456 0 .04563 0 .0457 0 .0457 0 .04571 0 .04574 0 .04581 0 .04582 0 .04587 0 .04591 0 .04596 0 .04596 0 .04598 0 .04602 0 .04603 0 .04615 0 .0462 0 .0462 0 .04622 0 .04623 0 .04623 0 .04628 0 .0463 0 .04635 0 .04637 0 .04639

0 . 195465871

0 . 1955847

0 . 195603729

0 . 195616734

0 . 195675603

0 . 195724634

0 . 195810023

0 . 196488294

0 . 196488294

0 . 196560893

0 . 196598921

0 . 196745766

0 . 197023352

0 . 197219118

0 . 197319847

0 . 197475002

0 . 197475002

0 . 197525666

0 . 197618107

0 . 197659043

0 . 197969288

0 . 197969288

0 . 197969288

0 . 197969288

0 . 198044229

0 . 198056489

0 . 198056489

0 . 19816459

0 . 19816459

0 . 19816459

0 . 198402765

0 . 198468627

0 . 198602857

0 . 198602857

0 . 198602857

0 . 198687414

0 . 198865146

0 . 198865146

0 . 199012234

0 . 199109369

0 . 199215844

0 . 199215844

0 . 19922835

0 . 199289481

0 . 199289481

0 . 199771741

0 . 199771741

0 . 199771741

0 . 199771741

0 . 199771741

0 . 199771741

0 . 199898994

0 . 199905409

0 .200081942

0 .200082002

0 .200113077

| TRAPPC8 -0 .331670933 | 7 .894153933 | -2 .0517 | 0 .04643 | 0 .200219265 |
| --- | --- | --- | --- | --- |
| GPAA1 0 .257447463 | 10 .83865454 | 2 .05147 | 0 .04646 | 0 .200219265 |
| CASQ2 -0 .363435641 | 5 .436409649 | -2 .0512 | 0 .04649 | 0 .200219265 |
| THOP1 0 .350507038 | 6 . 171487492 | 2 .05104 | 0 .0465 | 0 .200219265 |
| CTDSP2 -0 .235169264 | 9 .851466246 | -2 .0508 | 0 .04652 | 0 .200219265 |
| ANXA9 0 .443415725 | 7 .071645707 | 2 .05072 | 0 .04653 | 0 .200219265 |
| KIAA1109 -0 .255170374 | 8 .072089824 | -2 .0507 | 0 .04654 | 0 .200219265 |
| ZMYM3 -0 .341473564 | 7 .574628163 | -2 .0506 | 0 .04655 | 0 .200219265 |
| SAP30L 0 .397644711 | 5 .846180339 | 2 .05046 | 0 .04656 | 0 .200219265 |
| PGK1 0 .382574226 | 1 1 . 12755372 | 2 .04976 | 0 .04663 | 0 .200436871 |
| CDC27 0 . 184479674 | 8 .914499512 | 2 .04956 | 0 .04665 | 0 .200436871 |
| LAPTM4A -0 .260769507 | 10 .79980949 | -2 .0495 | 0 .04666 | 0 .200436871 |
| RP5-1 1 18D24 .2 -0 .429781832 | 8 .24344232 | -2 .0492 | 0 .04669 | 0 .20045211 |
| GPR144 0 .528342136 | 4 . 194194207 | 2 .04915 | 0 .04669 | 0 .20045211 |
| APOA4 0 .739434984 | 3 . 194766868 | 2 .04771 | 0 .04684 | 0 .201013274 |
| GUCA1B 0 .349803491 | 5 .56648053 | 2 .0472 | 0 .04689 | 0 .201169121 |
| STK17B 0 .307536455 | 5 .751686498 | 2 .04641 | 0 .04698 | 0 .201452023 |
| JAM2 -0 .356698666 | 6 .670205472 | -2 .0459 | 0 .04703 | 0 .201608008 |
| PTRF -0 .297063553 | 10 .44298913 | -2 .0457 | 0 .04704 | 0 .201610896 |
| IGHD 0 .533637941 | 8 .306560225 | 2 .04553 | 0 .04707 | 0 .201631271 |
| CXCR5 0 .435318855 | 5 .577844586 | 2 .04515 | 0 .0471 | 0 .201730131 |
| HHEX -0 .320895602 | 7 .325678419 | -2 .0446 | 0 .04717 | 0 .20192221 |
| CRYBA2 0 .894959313 | 3 .688790419 | 2 .04291 | 0 .04734 | 0 .202580389 |
| RASAL2 0 .411312845 | 5 .257089889 | 2 .04266 | 0 .04736 | 0 .202625551 |
| INSL6 0 .444604317 | 5 .5686911 19 | 2 .04218 | 0 .04741 | 0 .202767792 |
| HYI -0 .267840686 | 7 .23313463 | -2 .0418 | 0 .04745 | 0 .202864378 |
| SMC4 0 .245521521 | 8 . 145596582 | 2 .04147 | 0 .04749 | 0 .20294378 |
| PCGF3 -0 .21352942 | 8 .903918015 | -2 .0411 | 0 .04752 | 0 .203032366 |
| CACNA1B -0 .409538755 | 4 .486169604 | -2 .0408 | 0 .04755 | 0 .203054675 |
| LHFP -0 .384464398 | 7 .647030096 | -2 .0408 | 0 .04756 | 0 .203054675 |
| ZBTB5 -0 .287184025 | 7 .834059436 | -2 .0404 | 0 .04759 | 0 .203121911 |
| NAGA 0 .236078037 | 8 .579956933 | 2 .03975 | 0 .04766 | 0 .203361327 |
| RENBP 0 .7294411 18 | 6 .776284714 | 2 .03934 | 0 .04771 | 0 .20340305 |
| SLC26A2 -0 .515218455 | 7 . 179492142 | -2 .0393 | 0 .04771 | 0 .20340305 |
| MGP -0 .695690162 | 9 .353381332 | -2 .0392 | 0 .04772 | 0 .20340305 |
| ZNF79 -0 .262820892 | 7 .784952394 | -2 .039 | 0 .04774 | 0 .203410933 |
| CDH13 -0 .461129703 | 4 .489393431 | -2 .0378 | 0 .04787 | 0 .203813805 |
| SNRNP25 0 .242836521 | 7 .567973354 | 2 .03763 | 0 .04788 | 0 .203813805 |
| PNN 0 .240023906 | 10 .64765671 | 2 .03753 | 0 .0479 | 0 .203813805 |
| CYB5B 0 . 175729711 | 8 .311 170628 | 2 .03749 | 0 .0479 | 0 .203813805 |
| TUBG2 0 .22456453 | 6 .900804321 | 2 .0372 | 0 .04793 | 0 .203870334 |
| XPO4 0 .252832682 | 5 .48316928 | 2 .03665 | 0 .04799 | 0 .204025196 |
| HNRNPA0 -0 .205265397 | 9 .89201766 | -2 .0365 | 0 .048 | 0 .204025196 |
| INPP4B 0 .480486561 | 8 .402590383 | 2 .03623 | 0 .04803 | 0 .204094996 |
| ZNF135 -0 .56240007 | 3 .307773059 | -2 .0354 | 0 .04812 | 0 .204334474 |
| GCM1 -0 .292239243 | 4 .996158828 | -2 .0354 | 0 .04812 | 0 .204334474 |
| MSMO1 -0 .881853428 | 8 .27970603 | -2 .0352 | 0 .04814 | 0 .204348076 |
| ZC3H15 -0 .316829382 | 9 .095868642 | -2 .0349 | 0 .04818 | 0 .204400682 |
| PATZ1 -0 .202270043 | 9 .252142487 | -2 .0348 | 0 .04818 | 0 .204400682 |
| MSRA 0 .231974627 | 8 .090250719 | 2 .03443 | 0 .04822 | 0 .204482649 |
| ARHGDIB 0 .619923261 | 9 .67348503 | 2 .03423 | 0 .04824 | 0 .20450482 |
| PREB 0 . 153930809 | 7 .940982671 | 2 .03407 | 0 .04826 | 0 .204507039 |
| HPD 0 .694397036 | 2 . 192418079 | 2 .03387 | 0 .04828 | 0 .204524233 |
| ARPC1A 0 .314995238 | 8 .879271215 | 2 .03358 | 0 .04831 | 0 .204580266 |
| SRD5A3 -0 .429642116 | 4 .608837337 | -2 .0333 | 0 .04834 | 0 .204580266 |
| GNA14 0 .439195455 | 4 .432146146 | 2 .03319 | 0 .04835 | 0 .204580266 |

| UIMC1 | -0 .216304052 | 7 .539506713 | -2 .0331 | 0 .04836 | 0 .204580266 |
| --- | --- | --- | --- | --- | --- |
| PRUNE | -0 . 199761594 | 8 .375922284 | -2 .0329 | 0 .04839 | 0 .204580266 |
| TSPYL5 | -0 .38110356 | 7 .725220613 | -2 .0328 | 0 .0484 | 0 .204580266 |
| ENPP2 | -0 .667601461 | 10 . 10158238 | -2 .0325 | 0 .04842 | 0 .204580266 |
| CD86 | 0 .302376409 | 8 .080965491 | 2 .03251 | 0 .04842 | 0 .204580266 |
| SND1 | 0 . 191839868 | 10 .36854042 | 2 .03203 | 0 .04847 | 0 .204648364 |
| MPHOSPH10 | -0 .331872513 | 8 .091157104 | -2 .0318 | 0 .0485 | 0 .204648364 |
| MYBL1 | 0 .484267109 | 5 .219464714 | 2 .0317 | 0 .04851 | 0 .204648364 |
| FAM60A | -0 .372791143 | 8 .250196373 | -2 .0315 | 0 .04853 | 0 .204648364 |
| MCUR1 | 0 .340955768 | 8 .069086128 | 2 .03146 | 0 .04853 | 0 .204648364 |
| MUC7 | -0 .484552318 | 6 . 124921735 | -2 .0314 | 0 .04854 | 0 .204648364 |
| KIFC1 | 0 .241550987 | 7 .442369564 | 2 .03115 | 0 .04857 | 0 .204648364 |
| SLC35E1 | -0 .357011545 | 10 .20926727 | -2 .0311 | 0 .04857 | 0 .204648364 |
| STXBP3 | -0 .288934971 | 7 .545729491 | -2 .0309 | 0 .0486 | 0 .20469065 |
| LOC100293211 | 0 .655924276 | 6 .30932163 | 2 .03045 | 0 .04864 | 0 .204792981 |
| UBE2NL | 0 .311 136762 | 8 .074957941 | 2 .03034 | 0 .04865 | 0 .204792981 |
| CORT | 0 .54201318 | 6 .605801315 | 2 .03005 | 0 .04868 | 0 .20485329 |
| C17orf75 | -0 .541448825 | 4 .678608494 | -2 .0284 | 0 .04886 | 0 .205409799 |
| ST3GAL6 | -0 .575475949 | 8 .821401815 | -2 .0284 | 0 .04886 | 0 .205409799 |
| SPACA1 | 0 .383835753 | 3 .363909481 | 2 .02834 | 0 .04886 | 0 .205409799 |
| AVP | -0 .468319542 | 4 .812784149 | -2 .0281 | 0 .04889 | 0 .205464693 |
| TMEM144 | -0 .327778614 | 5 .647634873 | -2 .0278 | 0 .04892 | 0 .205509336 |
| MDFI | 0 .427105479 | 5 . 109508078 | 2 .02744 | 0 .04896 | 0 .205591552 |
| SHPK | 0 .297636176 | 6 .913958957 | 2 .02705 | 0 .049 | 0 .205591552 |
| GOLGA1 | 0 .213802023 | 8 . 1 19265648 | 2 .02692 | 0 .04901 | 0 .205591552 |
| HRK | 0 .445099303 | 6 .965638841 | 2 .02688 | 0 .04902 | 0 .205591552 |
| MAGEA10 | 0 .562179964 | 3 .783681597 | 2 .02686 | 0 .04902 | 0 .205591552 |
| HSPBP1 | 0 .268614686 | 8 . 135624645 | 2 .02652 | 0 .04906 | 0 .205594493 |
| GAS2L1 | -0 .41178797 | 9 .28394516 | -2 .0265 | 0 .04906 | 0 .205594493 |
| BRD9 | 0 . 162176888 | 7 .981972635 | 2 .02639 | 0 .04907 | 0 .205594493 |
| PIGV | -0 .230668756 | 7 .615112751 | -2 .0262 | 0 .04909 | 0 .205601128 |
| TRMT61A | 0 .252875858 | 7 .398942895 | 2 .02577 | 0 .04914 | 0 .205733742 |
| SQSTM1 | 0 .304089744 | 10 .50990167 | 2 .02504 | 0 .04922 | 0 .205992872 |
| LEPREL1 | -0 .473125086 | 6 .824618989 | -2 .0248 | 0 .04924 | 0 .206043823 |
| EXO1 | 0 .355492964 | 6 .50689512 | 2 .02408 | 0 .04932 | 0 .20628371 |
| ZNF415 | -0 .316559995 | 5 .578965409 | -2 .0236 | 0 .04937 | 0 .206427235 |
| SNRPD3 | 0 .310760343 | 9 . 126146407 | 2 .02343 | 0 .04939 | 0 .206427235 |
| HEATR3 | 0 .265258658 | 6 . 140481406 | 2 .0233 | 0 .0494 | 0 .206427235 |
| MGRN1 | 0 .261616772 | 8 . 173612459 | 2 .0229 | 0 .04945 | 0 .20648491 |
| CFB | 0 .476403543 | 6 .576346882 | 2 .02287 | 0 .04945 | 0 .20648491 |
| DDX19A | 0 .224824209 | 8 .30424012 | 2 .02264 | 0 .04947 | 0 .206517986 |
| LOC100129361 | 0 .416062755 | 7 .433540518 | 2 .02208 | 0 .04953 | 0 .206701622 |
| CCDC186 | -0 .41572047 | 4 .737387181 | -2 .0219 | 0 .04956 | 0 .206733002 |
| AZGP1 | -0 .69816264 | 8 .222672694 | -2 .0208 | 0 .04967 | 0 .207132226 |
| PLCL1 | 0 .46332234 | 5 . 1 15230968 | 2 .01994 | 0 .04976 | 0 .207445893 |
| ZRSR2 | 0 .231128054 | 9 .341487027 | 2 .01965 | 0 .0498 | 0 .207445893 |
| RUNX3 | 0 .294582463 | 10 .28761316 | 2 .01957 | 0 .0498 | 0 .207445893 |
| F5 | -0 .495122306 | 8 .088956027 | -2 .0194 | 0 .04982 | 0 .207445893 |
| FBXL8 | -0 .364519056 | 4 .659918261 | -2 .0194 | 0 .04983 | 0 .207445893 |
| TOLLIP | 0 .358420255 | 7 .300411292 | 2 .01908 | 0 .04986 | 0 .207501184 |
| NPBWR2 | 0 .44885444 | 3 .808527815 | 2 .01878 | 0 .04989 | 0 .207567259 |
| BUB3 | -0 .217001915 | 10 .05433407 | -2 .0183 | 0 .04994 | 0 .207653254 |
| BRE | 0 . 183883118 | 9 .496077878 | 2 .01815 | 0 .04996 | 0 .207653254 |
| KCNJ8 | -0 .265818788 | 7 .670023871 | -2 .0181 | 0 .04996 | 0 .207653254 |
| SUMO4 | -0 .249714451 | 9 .753894529 | -2 .0178 | 0 .05 | 0 .207749544 |
| SFRP1 | -0 .739321205 | 9 .759140557 | -2 .0163 | 0 .05015 | 0 .208320534 |

| NDUFA4 | -0 .418411212 | 9 .054056363 | -2 .0161 | 0 .05018 | 0 .208347624 |
| --- | --- | --- | --- | --- | --- |
| KLF10 | -0 .282273534 | 8 .576527452 | -2 .0154 | 0 .05026 | 0 .208623495 |
| RNF24 | 0 .262751073 | 8 .961719889 | 2 .01437 | 0 .05037 | 0 .209006908 |
| TMED10 | -0 .286955269 | 10 .84571403 | -2 .0142 | 0 .05039 | 0 .209016375 |
| CASP8 | 0 .258424505 | 7 .350397868 | 2 .01389 | 0 .05042 | 0 .209057567 |
| MSL3 | 0 .214283757 | 7 .545429664 | 2 .0138 | 0 .05043 | 0 .209057567 |
| LY6E | 0 .622278694 | 10 .00292442 | 2 .0133 | 0 .05049 | 0 .20921288 |
| ATP7A | -0 .341084074 | 7 .544895644 | -2 .013 | 0 .05052 | 0 .209304012 |
| GRM5 | 0 .365682922 | 5 .923475006 | 2 .01271 | 0 .05055 | 0 .209342253 |
| ZNF24 | -0 .302747873 | 9 . 123865496 | -2 .0124 | 0 .05059 | 0 .209435662 |
| NUPL1 | 0 .20799429 | 6 .769164815 | 2 .01218 | 0 .05061 | 0 .209444798 |
| AL109706 | -0 .32557888 | 5 .89032101 | -2 .011 | 0 .05074 | 0 .209913675 |
| ZFP64 | 0 .438935972 | 8 .082434764 | 2 .01085 | 0 .05076 | 0 .209913675 |
| YY1 | -0 . 189803952 | 10 .30909758 | -2 .0106 | 0 .05078 | 0 .209942597 |
| RAI14 | 0 .40097866 | 7 .065801594 | 2 .00956 | 0 .0509 | 0 .21036033 |
| LBP | -0 .289053013 | 7 .834504567 | -2 .0093 | 0 .05093 | 0 .210378857 |
| TM2D3 | -0 .307617414 | 7 .923508571 | -2 .0092 | 0 .05093 | 0 .210378857 |
| TRABD | 0 .51239161 | 5 .470889154 | 2 .00874 | 0 .05099 | 0 .210526241 |
| MUM1 | -0 .350465999 | 6 .592673518 | -2 .0078 | 0 .0511 | 0 .210903666 |
| ZBTB39 | -0 .321606302 | 6 . 168928115 | -2 .0074 | 0 .05113 | 0 .210994945 |
| SLC30A3 | -0 .300476169 | 3 .00946215 | -2 .0069 | 0 .05119 | 0 .211 151891 |
| CASK | -0 .24602402 | 8 .379477531 | -2 .0065 | 0 .05124 | 0 .211277125 |
| RALA | 0 .336736469 | 7 .248760798 | 2 .00633 | 0 .05125 | 0 .211277125 |
| ABCC10 | 0 .231054749 | 8 .31020377 | 2 .00571 | 0 .05132 | 0 .211491202 |
| POLR1D | -0 .428158601 | 8 .571271525 | -2 .0052 | 0 .05138 | 0 .211652904 |
| CRISP2 | -0 .601244232 | 3 .255221065 | -2 .0042 | 0 .05149 | 0 .212023547 |
| VIP | -0 .621097923 | 2 .919322667 | -2 .0037 | 0 .05154 | 0 .212187717 |
| BCAR3 | 0 .51015228 | 5 .789917487 | 2 .00304 | 0 .05162 | 0 .212437716 |
| BCL7B | 0 . 199972581 | 7 .705959084 | 2 .00166 | 0 .05177 | 0 .213003112 |
| HDAC11 | -0 .657930592 | 2 .797315052 | -2 .001 | 0 .05185 | 0 .213187698 |
| KLHDC4 | 0 .264966213 | 6 .539604884 | 2 .00095 | 0 .05185 | 0 .213187698 |
| SIX3 | -0 .374518627 | 5 .779660084 | -2 .0006 | 0 .0519 | 0 .213299028 |
| MAN2A1 | -0 .355866774 | 7 .223448669 | -2 .0001 | 0 .05195 | 0 .213460647 |
| GCA | -0 .373017112 | 5 .694296821 | -1 .9993 | 0 .05204 | 0 .213751144 |
| EXOSC7 | -0 . 182956746 | 8 . 198459497 | -1 .9988 | 0 .05209 | 0 .213885731 |
| STIP1 | 0 .317123668 | 8 .363441745 | 1 .99774 | 0 .05221 | 0 .214258051 |
| SLC9A6 | -0 .244010882 | 7 .434292285 | -1 .9977 | 0 .05222 | 0 .214258051 |
| PAPPA | 0 .208802458 | 6 .492062521 | 1 .99724 | 0 .05227 | 0 .214375612 |
| MTHFD1 | 0 .266898621 | 8 .383118182 | 1 .99717 | 0 .05228 | 0 .214375612 |
| SLC25A23 | -0 .423319583 | 6 .057209151 | -1 .9969 | 0 .05231 | 0 .214451137 |
| IFIT2 | 0 .37427777 | 7 .063238739 | 1 .99667 | 0 .05233 | 0 .214463144 |
| GABRQ | 0 .343580624 | 6 .45791148 | 1 .9964 | 0 .05237 | 0 .214520789 |
| CDC42EP3 | -0 .346924692 | 9 .807273714 | -1 .9962 | 0 .05239 | 0 .214559533 |
| CDO1 | -0 .532865692 | 5 .938583709 | -1 .9957 | 0 .05244 | 0 .21468575 |
| ESM1 | 0 .317322712 | 4 .522698202 | 1 .99559 | 0 .05246 | 0 .21468575 |
| POLD4 | 0 .327872929 | 6 .777800118 | 1 .99526 | 0 .05249 | 0 .214766914 |
| SNCB | 0 .885453628 | 4 .339139542 | 1 .99509 | 0 .05251 | 0 .214773301 |
| METRN | 0 .51315957 | 7 .53959306 | 1 .99448 | 0 .05258 | 0 .214960251 |
| WAPAL | -0 .285439187 | 7 .447585938 | -1 .9944 | 0 .05259 | 0 .214960251 |
| HILPDA | -0 .488400607 | 7 .714539808 | -1 .9939 | 0 .05265 | 0 .215114778 |
| SPG11 | -0 .280453092 | 7 .81552717 | -1 .9938 | 0 .05266 | 0 .215114778 |
| UBA1 | 0 .290384074 | 10 .02612446 | 1 .99322 | 0 .05272 | 0 .215291217 |
| HSF1 | 0 .286904337 | 8 .247657335 | 1 .99229 | 0 .05283 | 0 .215622984 |
| ENC1 | 0 .283937731 | 8 .881687816 | 1 .99218 | 0 .05284 | 0 .215622984 |
| GOLT1B | 0 .33396626 | 6 .697936014 | 1 .99205 | 0 .05286 | 0 .215622984 |
| LAMB2 | -0 .296621408 | 8 .897036505 | -1 .9917 | 0 .0529 | 0 .21567858 |

| FLAD1 | 0 .263735234 | 7 .512126463 | 1 .99163 | 0 .05291 | 0 .21567858 |
| --- | --- | --- | --- | --- | --- |
| DSCR3 | -0 .284903715 | 7 .707105145 | -1 .9909 | 0 .05299 | 0 .215932966 |
| FDFT1 | -0 .380146087 | 10 .86262332 | -1 .9907 | 0 .05301 | 0 .215953842 |
| TERT | 0 .529301686 | 4 .455928603 | 1 .98956 | 0 .05314 | 0 .216388683 |
| 5-Mar | -0 .217192584 | 6 .346344451 | -1 .9895 | 0 .05315 | 0 .216388683 |
| ENOPH1 | -0 .309393897 | 7 .697888179 | -1 .9888 | 0 .05323 | 0 .216575881 |
| MCL1 | 0 .247065059 | 10 .70511 177 | 1 .98858 | 0 .05325 | 0 .216575881 |
| ZCCHC8 | -0 .364424096 | 6 .479125918 | -1 .9884 | 0 .05327 | 0 .216575881 |
| AC004692 .5 | -0 .38086105 | 8 .759334521 | -1 .9884 | 0 .05328 | 0 .216575881 |
| RBM4B | -0 .228164635 | 6 .635003119 | -1 .9883 | 0 .05328 | 0 .216575881 |
| PLCB4 | -0 .388621517 | 5 .791776269 | -1 .9877 | 0 .05336 | 0 .216807716 |
| HPGDS | -0 .232537387 | 6 . 18279544 | -1 .9875 | 0 .05337 | 0 .216807716 |
| ST6GAL1 | -0 .331215644 | 8 .881278178 | -1 .9873 | 0 .0534 | 0 .216832381 |
| SLC35B1 | 0 .222096661 | 8 .799627092 | 1 .98704 | 0 .05343 | 0 .216904834 |
| CST5 | 0 .879372697 | 4 .782128727 | 1 .98633 | 0 .05351 | 0 .217165314 |
| KMO | 0 .274419134 | 7 . 160221498 | 1 .98601 | 0 .05355 | 0 .217243614 |
| SHANK2 | -0 .310430434 | 6 .932167681 | -1 .9859 | 0 .05357 | 0 .21724783 |
| ARMCX2 | -0 .491182835 | 6 .21397626 | -1 .9856 | 0 .0536 | 0 .217309029 |
| EIF3B | 0 .224810875 | 10 .88599771 | 1 .98528 | 0 .05363 | 0 .217318744 |
| EPHB2 | 0 .273181758 | 8 .335282962 | 1 .9851 | 0 .05365 | 0 .217318744 |
| SOCS6 | 0 .374340322 | 7 .085915682 | 1 .9851 | 0 .05365 | 0 .217318744 |
| PCM1 | -0 .233134818 | 9 .595529545 | -1 .9848 | 0 .05369 | 0 .217386157 |
| LOC730101 | 0 .393839793 | 5 .797963899 | 1 .98408 | 0 .05377 | 0 .217636008 |
| CMC4 | -0 .270023558 | 9 .062788702 | -1 .984 | 0 .05378 | 0 .217636008 |
| CPNE7 | 0 .394165246 | 7 . 1432756 | 1 .98368 | 0 .05382 | 0 .217700291 |
| EPHX3 | 0 .824206473 | 7 .39830879 | 1 .98343 | 0 .05385 | 0 .217710389 |
| KIAA0040 | 0 .224512453 | 7 .60455819 | 1 .98336 | 0 .05386 | 0 .217710389 |
| FAM3C | -0 .444078204 | 8 .29552277 | -1 .9819 | 0 .05402 | 0 .218310229 |
| UBE2G2 | -0 .235060184 | 9 .024452534 | -1 .9809 | 0 .05414 | 0 .218705524 |
| PLEKHA5 | -0 .419850216 | 8 .432635756 | -1 .9806 | 0 .05417 | 0 .218712793 |
| DSERG1 | -0 .383885838 | 5 .917524274 | -1 .9806 | 0 .05418 | 0 .218712793 |
| MBTD1 | -0 .857573926 | 2 .976281422 | -1 .9805 | 0 .05419 | 0 .218712793 |
| CD37 | 1 .060637268 | 6 .821565935 | 1 .97927 | 0 .05433 | 0 .219205458 |
| PPP2R5B | 0 .284891847 | 7 .474948155 | 1 .97812 | 0 .05446 | 0 .219647102 |
| CNR1 | -0 .421482295 | 6 . 1 1 1316981 | -1 .978 | 0 .05447 | 0 .219647102 |
| TOMM22 | -0 .327190437 | 6 . 133320996 | -1 .9777 | 0 .05452 | 0 .219738795 |
| COASY | 0 .231785765 | 8 .569568089 | 1 .97739 | 0 .05455 | 0 .219738795 |
| UBXN2B | 0 .482532526 | 5 .607028018 | 1 .97729 | 0 .05456 | 0 .219738795 |
| HOXB8 | 0 .479916874 | 4 .259097203 | 1 .97724 | 0 .05457 | 0 .219738795 |
| LRRC14 | 0 .324960236 | 5 .773870084 | 1 .97708 | 0 .05459 | 0 .219746796 |
| TSPYL4 | -0 .285695886 | 7 .748478862 | -1 .9768 | 0 .05462 | 0 .21980139 |
| HSD3B1 | -0 .62296331 | 5 .953678307 | -1 .9766 | 0 .05465 | 0 .219829011 |
| EPHA2 | 0 .378285837 | 7 . 178903831 | 1 .97645 | 0 .05466 | 0 .219829011 |
| UBE3A | -0 .327741901 | 9 .575480865 | -1 .9753 | 0 .05479 | 0 .220283236 |
| LIMD2 | 0 .507834245 | 6 .513353807 | 1 .97517 | 0 .05481 | 0 .220294371 |
| TMEM248 | 0 .296190047 | 8 .35364625 | 1 .97418 | 0 .05493 | 0 .220642648 |
| DONSON | 0 .361519599 | 6 .259487933 | 1 .97413 | 0 .05493 | 0 .220642648 |
| FAM106A | -0 .508747082 | 5 . 192247901 | -1 .9735 | 0 .055 | 0 .22085415 |
| PDPN | 0 .248214146 | 7 .221425611 | 1 .97247 | 0 .05513 | 0 .221187838 |
| SPR | 0 .260615372 | 8 .02732724 | 1 .97241 | 0 .05514 | 0 .221187838 |
| B4GALT3 | 0 .250640108 | 7 .726603962 | 1 .97238 | 0 .05514 | 0 .221187838 |
| GIP | 0 .462753871 | 3 .79905148 | 1 .97194 | 0 .05519 | 0 .221326698 |
| CD247 | 0 .530369019 | 7 .077815638 | 1 .97179 | 0 .05521 | 0 .221326698 |
| UTP11L | 0 .232841692 | 7 .943996374 | 1 .97149 | 0 .05524 | 0 .22136463 |
| LRBA | -0 . 187442919 | 8 .233551308 | -1 .9714 | 0 .05526 | 0 .22136463 |
| CACNA1C | 0 .395989849 | 5 .29152281 | 1 .97126 | 0 .05527 | 0 .22136463 |

| RFPL1S | 0 .546811022 | 4 . 102981635 | 1 .9711 | 0 .05529 | 0 .221373365 |
| --- | --- | --- | --- | --- | --- |
| ACP2 | 0 .208604526 | 8 .467334541 | 1 .96877 | 0 .05557 | 0 .222405254 |
| REPS1 | -0 .420758258 | 5 .045856823 | -1 .9682 | 0 .05563 | 0 .222544643 |
| DENND2D | 0 .489147599 | 6 .770812842 | 1 .96806 | 0 .05565 | 0 .222544643 |
| CRABP2 | 0 .591539901 | 9 .671054608 | 1 .96803 | 0 .05565 | 0 .222544643 |
| SH2B2 | -0 .346977863 | 7 .914520328 | -1 .9675 | 0 .05571 | 0 .222643896 |
| TSC1 | -0 .292720084 | 6 .741105156 | -1 .9675 | 0 .05571 | 0 .222643896 |
| ANKRD12 | -0 .32645916 | 7 .787475703 | -1 .9667 | 0 .05582 | 0 .222900937 |
| PIEZO1 | 0 .313921869 | 8 . 195308256 | 1 .9666 | 0 .05582 | 0 .222900937 |
| ZBTB44 | -0 .48280906 | 5 .325279942 | -1 .9664 | 0 .05585 | 0 .222900937 |
| BNC2 | -0 .342871914 | 7 .749514804 | -1 .9663 | 0 .05586 | 0 .222900937 |
| DLX6 | -0 .3930211 | 4 .595170036 | -1 .9662 | 0 .05587 | 0 .222900937 |
| PAK1IP1 | 0 .567780192 | 6 .206993209 | 1 .96581 | 0 .05592 | 0 .223038489 |
| HGH1 | 0 .290463712 | 9 .42158311 | 1 .96503 | 0 .05601 | 0 .223337403 |
| RNF186 | 0 .54777125 | 4 .271178494 | 1 .96443 | 0 .05608 | 0 .223550575 |
| PEG3 | -0 .441822279 | 6 .527497994 | -1 .964 | 0 .05613 | 0 .223639849 |
| ITFG2 | -0 .515905129 | 4 .91405992 | -1 .964 | 0 .05614 | 0 .223639849 |
| APEH | 0 .251176178 | 7 .804748967 | 1 .9637 | 0 .05617 | 0 .22368968 |
| SEL1L | -0 . 183376747 | 9 .276400997 | -1 .9632 | 0 .05623 | 0 .22384227 |
| NR2F2 | -0 .291121617 | 9 .604560722 | -1 .9626 | 0 .0563 | 0 .224040535 |
| GPR143 | 0 .530772795 | 8 .673065117 | 1 .96252 | 0 .05631 | 0 .224040535 |
| METTL9 | -0 .423190955 | 10 . 18367376 | -1 .9624 | 0 .05633 | 0 .224047811 |
| SNX6 | -0 .201138414 | 8 .089531852 | -1 .9622 | 0 .05635 | 0 .224048518 |
| RUVBL1 | 0 .274837548 | 7 .722972965 | 1 .96148 | 0 .05644 | 0 .224324613 |
| VPS33B | 0 . 174210995 | 8 .829136522 | 1 .96077 | 0 .05652 | 0 .22459538 |
| ACO2 | 0 . 199206712 | 8 .955952066 | 1 .96054 | 0 .05655 | 0 .224631613 |
| HBZ | -0 .288996855 | 6 .234858307 | -1 .96 | 0 .05661 | 0 .224806524 |
| NPTN | -0 .369665686 | 9 .861326173 | -1 .9593 | 0 .0567 | 0 .225074927 |
| DUX1 | 0 .587756472 | 4 .347490825 | 1 .95907 | 0 .05673 | 0 .225124975 |
| ELF4 | 0 .448603689 | 7 .699400455 | 1 .95877 | 0 .05677 | 0 .225196724 |
| LAT | 0 .389662942 | 7 .291281242 | 1 .95861 | 0 .05678 | 0 .225200277 |
| PRSS22 | 0 .622035339 | 6 .82102803 | 1 .95736 | 0 .05694 | 0 .225731125 |
| RTN4 | -0 .319770135 | 12 .46993373 | -1 .9565 | 0 .05704 | 0 .226088024 |
| TSR2 | -0 . 192999519 | 7 .387170651 | -1 .9559 | 0 .05711 | 0 .226285619 |
| CELF1 | -0 .226848978 | 10 . 16465809 | -1 .9555 | 0 .05717 | 0 .226376654 |
| RPLP1 | 0 . 17065894 | 12 .9619214 | 1 .9554 | 0 .05718 | 0 .226376654 |
| SLC6A15 | -0 .350269675 | 7 . 107329898 | -1 .9553 | 0 .05719 | 0 .226376654 |
| NPC2 | 0 .202303097 | 10 .61089699 | 1 .95481 | 0 .05725 | 0 .226531947 |
| GPX3 | -0 .630583185 | 10 .9240016 | -1 .9539 | 0 .05736 | 0 .226882343 |
| IGLV1-44 | 1 .416285492 | 10 .07629522 | 1 .95379 | 0 .05737 | 0 .226882343 |
| NOLC1 | 0 .306187174 | 9 .442486049 | 1 .9533 | 0 .05743 | 0 .227013143 |
| FBXO5 | 0 .289014578 | 5 .386416333 | 1 .95322 | 0 .05744 | 0 .227013143 |
| HOOK1 | -0 .458215237 | 5 .549197972 | -1 .9523 | 0 .05755 | 0 .227371203 |
| MRPL46 | -0 .20694335 | 7 .029254743 | -1 .952 | 0 .05759 | 0 .227451748 |
| GRINA | 0 .353653863 | 8 .771182872 | 1 .95136 | 0 .05767 | 0 .227702525 |
| STARD3 | 0 .255035917 | 8 .25990523 | 1 .95103 | 0 .05771 | 0 .227763833 |
| SSX2B | -0 .666189465 | 4 .225831452 | -1 .9509 | 0 .05772 | 0 .227763833 |
| RRS1 | 0 .335682244 | 7 .701225559 | 1 .95002 | 0 .05783 | 0 .228133865 |
| GLTP | 0 .514745113 | 8 .470121363 | 1 .9496 | 0 .05789 | 0 .228230636 |
| GHRH | 0 .340496335 | 5 .834605886 | 1 .94951 | 0 .0579 | 0 .228230636 |
| CCL4 | 0 .621157927 | 6 .785424604 | 1 .94938 | 0 .05791 | 0 .228230636 |
| EVI2B | 0 .457831286 | 7 .253279631 | 1 .94853 | 0 .05802 | 0 .228545196 |
| SEC62 | -0 .268317845 | 9 .618623842 | -1 .9484 | 0 .05804 | 0 .228545196 |
| XPA | -0 .317473383 | 6 .339197446 | -1 .9483 | 0 .05805 | 0 .228545196 |
| GFPT2 | -0 .283832506 | 6 .262212368 | -1 .9476 | 0 .05813 | 0 .228784752 |
| EPO | 0 .501489632 | 5 .019915267 | 1 .94579 | 0 .05836 | 0 .229609193 |

| FBXW4P1 | -0 .518730347 | 5 .326102302 | -1 .9457 | 0 .05837 | 0 .229609193 |
| --- | --- | --- | --- | --- | --- |
| RP11-348N5 .7 | -0 .470970851 | 4 .013972142 | -1 .9444 | 0 .05853 | 0 .230126015 |
| NAAA | 0 .240361331 | 8 .483686206 | 1 .94431 | 0 .05854 | 0 .230126015 |
| RUNDC3A | 0 .365021886 | 7 . 166576651 | 1 .94394 | 0 .05859 | 0 .230184835 |
| SDS | 0 .322557092 | 6 .347639395 | 1 .94389 | 0 .05859 | 0 .230184835 |
| BACE1 | -0 .318761011 | 6 .960068644 | -1 .9437 | 0 .05861 | 0 .230191613 |
| UBE3C | 0 . 191498986 | 7 .999919259 | 1 .94258 | 0 .05876 | 0 .230685214 |
| MOSPD1 | 0 .277421841 | 7 . 178981737 | 1 .94194 | 0 .05884 | 0 .230812356 |
| LGALS3 | 0 .234944389 | 1 1 .8095295 | 1 .94191 | 0 .05884 | 0 .230812356 |
| NUP43 | -0 .358880448 | 7 .397884565 | -1 .9416 | 0 .05888 | 0 .230812356 |
| OXCT2 | 0 .498136512 | 4 .784284743 | 1 .94154 | 0 .05889 | 0 .230812356 |
| ENOX1 | 0 .319498021 | 5 .876056664 | 1 .94148 | 0 .05889 | 0 .230812356 |
| WDR43 | -0 .301976571 | 6 .477909783 | -1 .9414 | 0 .0589 | 0 .230812356 |
| EFHD1 | -0 .643963444 | 7 .645060224 | -1 .9405 | 0 .05902 | 0 .231215956 |
| ARHGEF5 | -0 .393088336 | 6 .59323908 | -1 .9399 | 0 .05909 | 0 .23142556 |
| ENTPD1-AS1 | -0 .26044879 | 6 .23069323 | -1 .9395 | 0 .05915 | 0 .231556463 |
| SORBS3 | -0 .252710654 | 9 .608726291 | -1 .9393 | 0 .05916 | 0 .231556463 |
| LTBR | 0 .334875777 | 7 .684611817 | 1 .93899 | 0 .05921 | 0 .231651586 |
| PRKCE | -0 .651465012 | 6 .454601367 | -1 .9387 | 0 .05924 | 0 .231708472 |
| ZNF593 | 0 .384530408 | 7 .388098769 | 1 .93832 | 0 .05929 | 0 .231832676 |
| PARP12 | 0 .398205764 | 7 .723796614 | 1 .93754 | 0 .05939 | 0 .232144866 |
| RHOF | 0 .468193748 | 6 .536930676 | 1 .93737 | 0 .05941 | 0 .232156231 |
| DYRK1A | -0 .21157011 | 9 .004133859 | -1 .9368 | 0 .05948 | 0 .232338829 |
| BEND5 | -0 .475374774 | 6 .005219361 | -1 .9367 | 0 .05949 | 0 .232338829 |
| OSMR | 0 .347100075 | 5 .81331395 | 1 .93602 | 0 .05958 | 0 .232604032 |
| CLIC3 | 0 .860053372 | 3 .881166717 | 1 .93587 | 0 .0596 | 0 .232604032 |
| LRIT1 | -0 .501286923 | 4 . 1 1386198 | -1 .9355 | 0 .05965 | 0 .232735742 |
| HYPM | 0 .324624501 | 6 .035225066 | 1 .93528 | 0 .05967 | 0 .232749584 |
| RPARP-AS1 | -0 .259771335 | 6 . 127531345 | -1 .9351 | 0 .0597 | 0 .232783399 |
| NAT9 | -0 .267330979 | 7 .221106432 | -1 .9346 | 0 .05975 | 0 .232923655 |
| SRSF7 | -0 .251514977 | 9 .595044685 | -1 .9339 | 0 .05985 | 0 .233238473 |
| CCNE2 | 0 .338405495 | 5 .662437676 | 1 .93335 | 0 .05992 | 0 .233417093 |
| PHLPP1 | -0 .381886062 | 8 .486065117 | -1 .933 | 0 .05995 | 0 .233478742 |
| TMEM123 | -0 .412137105 | 8 .229349607 | -1 .9329 | 0 .05997 | 0 .233478742 |
| BTN2A1 | 0 .296925549 | 8 .938625548 | 1 .93228 | 0 .06005 | 0 .23372487 |
| ZMAT5 | 0 .427156245 | 4 .834147484 | 1 .9321 | 0 .06008 | 0 .233725838 |
| ST20 | 0 .271814424 | 6 .854953152 | 1 .93199 | 0 .06009 | 0 .233725838 |
| PTPN4 | -0 .30375036 | 6 .575460733 | -1 .9315 | 0 .06015 | 0 .233876027 |
| H1FX | -0 .327792342 | 8 .816717132 | -1 .9296 | 0 .06039 | 0 .234754939 |
| WIPI2 | 0 . 163902036 | 10 .41615503 | 1 .92937 | 0 .06042 | 0 .234771239 |
| XCL1 | 0 .408399805 | 6 .811069813 | 1 .92929 | 0 .06043 | 0 .234771239 |
| PFDN2 | 0 .290348287 | 8 .533883846 | 1 .92912 | 0 .06046 | 0 .234785729 |
| DCTPP1 | -0 .303796023 | 8 .370193903 | -1 .9282 | 0 .06057 | 0 .235170117 |
| MED7 | -0 .276250891 | 7 .935523561 | -1 .9276 | 0 .06065 | 0 .235362648 |
| PAX5 | 0 .359409547 | 6 .450273888 | 1 .92752 | 0 .06066 | 0 .235362648 |
| ITM2C | 0 .290117757 | 8 .512317372 | 1 .92679 | 0 .06075 | 0 .235653522 |
| PES1 | 0 .32223933 | 7 .482687288 | 1 .92631 | 0 .06081 | 0 .235817516 |
| PLA2R1 | -0 .292856239 | 5 .022378189 | -1 .926 | 0 .06086 | 0 .235860129 |
| MAGEA4 | 0 .664379718 | 4 .852513623 | 1 .92593 | 0 .06086 | 0 .235860129 |
| RAB11A | 0 .279703687 | 10 .09429484 | 1 .92469 | 0 .06102 | 0 .236408783 |
| ETS2 | -0 .303508064 | 8 .246000722 | -1 .9238 | 0 .06114 | 0 .236792692 |
| LASP1 | 0 .22488093 | 9 .761735801 | 1 .92354 | 0 .06117 | 0 .236832744 |
| CLEC4E | 0 .465177353 | 5 .973693986 | 1 .92335 | 0 .0612 | 0 .236856167 |
| TMEM9B | -0 .261826299 | 8 .314878279 | -1 .9225 | 0 .06131 | 0 .237227432 |
| NAP1L2 | -0 .390452643 | 4 .243214347 | -1 .9214 | 0 .06145 | 0 .237655508 |
| FST | -0 .337185038 | 7 .934130798 | -1 .9213 | 0 .06147 | 0 .237655508 |

| SAP30 | -0 .306138857 | 8 .05964022 | -1 .9212 | 0 .06148 | 0 .237655508 |
| --- | --- | --- | --- | --- | --- |
| CASP5 | -0 .437098392 | 4 . 190078921 | -1 .9207 | 0 .06154 | 0 .237715977 |
| HEXIM1 | 0 .3655721 | 7 .604593165 | 1 .92061 | 0 .06155 | 0 .237715977 |
| TDG | 0 .326343217 | 6 .403966754 | 1 .92061 | 0 .06155 | 0 .237715977 |
| H6PD | -0 .211866563 | 7 .947532413 | -1 .9204 | 0 .06158 | 0 .237738877 |
| AZI2 | -0 .395978587 | 4 .905148 | -1 .9201 | 0 .06162 | 0 .237850809 |
| KDM4B | 0 . 194443518 | 9 .894796989 | 1 .91938 | 0 .06171 | 0 .237992624 |
| SLC12A8 | 0 .544151976 | 5 .989637028 | 1 .91935 | 0 .06171 | 0 .237992624 |
| CLEC16A | 0 .20504952 | 6 .957278808 | 1 .91933 | 0 .06172 | 0 .237992624 |
| AKR1A1 | -0 .298821905 | 9 . 131443309 | -1 .9189 | 0 .06177 | 0 .238021741 |
| RGL1 | -0 .25602691 | 7 .617161153 | -1 .9189 | 0 .06178 | 0 .238021741 |
| COQ10B | 0 .24695088 | 7 .959998921 | 1 .91881 | 0 .06179 | 0 .238021741 |
| P2RY2 | -0 .479724929 | 4 .894664313 | -1 .9187 | 0 .0618 | 0 .238021741 |
| TSC22D4 | 0 .377974862 | 7 .98739753 | 1 .91844 | 0 .06183 | 0 .238032349 |
| HIST1H2AJ | 0 .33650856 | 5 .414523579 | 1 .91838 | 0 .06184 | 0 .238032349 |
| GRIN2C | 0 .26447541 | 5 .71178522 | 1 .91796 | 0 .0619 | 0 .238105929 |
| KPTN | 0 .347975366 | 6 .763480742 | 1 .91793 | 0 .0619 | 0 .238105929 |
| PRPSAP2 | -0 .262729654 | 6 .78087693 | -1 .9178 | 0 .06192 | 0 .238105929 |
| CD207 | -0 .451308137 | 7 .367559571 | -1 .9175 | 0 .06196 | 0 .238142655 |
| VPS4A | 0 .234831746 | 8 .256350627 | 1 .91729 | 0 .06198 | 0 .238142655 |
| TDRD1 | -0 .504532027 | 4 .932808989 | -1 .9173 | 0 .06198 | 0 .238142655 |
| IL2RG | 0 .348278362 | 9 .32414159 | 1 .91686 | 0 .06204 | 0 .238283071 |
| ZNF350 | -0 .353614756 | 5 .438951203 | -1 .9164 | 0 .06209 | 0 .238423216 |
| CHRNA2 | 0 .37513581 | 6 .389691012 | 1 .91495 | 0 .06229 | 0 .239053516 |
| CDH2 | 0 .459490788 | 7 . 1 1802265 | 1 .91489 | 0 .0623 | 0 .239053516 |
| MIA | 0 .88171643 | 9 .088884573 | 1 .91451 | 0 .06235 | 0 .239128077 |
| CLTA | -0 . 148012181 | 12 .05451831 | -1 .9145 | 0 .06235 | 0 .239128077 |
| HSD17B14 | 0 .366845838 | 6 .871926793 | 1 .91378 | 0 .06244 | 0 .239226701 |
| KANSL1L | -0 .644649234 | 6 .09683151 | -1 .9137 | 0 .06246 | 0 .239226701 |
| ADA | 0 .274164789 | 7 .392264948 | 1 .91368 | 0 .06246 | 0 .239226701 |
| FAM117A | -0 .314243444 | 7 . 144258031 | -1 .9137 | 0 .06246 | 0 .239226701 |
| IL7R | 0 .77428464 | 8 .034494847 | 1 .91326 | 0 .06251 | 0 .239360986 |
| ITPK1 | 0 .329366997 | 9 .499948914 | 1 .91294 | 0 .06255 | 0 .239364867 |
| CCNJ | 0 .362617825 | 4 .848995269 | 1 .91268 | 0 .06259 | 0 .239364867 |
| FBP2 | 0 .41768943 | 2 .276449157 | 1 .91248 | 0 .06261 | 0 .239364867 |
| S1PR1 | -0 .288281025 | 6 .429664201 | -1 .9124 | 0 .06262 | 0 .239364867 |
| DBT | -0 .382112491 | 10 .28766127 | -1 .9124 | 0 .06262 | 0 .239364867 |
| NUS1P3 | -0 .279247417 | 5 .589284905 | -1 .9123 | 0 .06264 | 0 .239364867 |
| SIGLEC9 | 0 .372154445 | 5 .382190628 | 1 .91224 | 0 .06265 | 0 .239364867 |
| BTN2A2 | 0 .256803605 | 7 .997260338 | 1 .9119 | 0 .06269 | 0 .239465493 |
| DBNDD1 | 0 .33381298 | 6 .387233968 | 1 .91147 | 0 .06275 | 0 .239547831 |
| FGF2 | -0 .324659653 | 6 .201615768 | -1 .9114 | 0 .06275 | 0 .239547831 |
| VPS13D | -0 .232168919 | 8 .379101076 | -1 .9106 | 0 .06287 | 0 .239916446 |
| ARL6IP5 | -0 .33122024 | 10 .64233955 | -1 .9101 | 0 .06293 | 0 .240099897 |
| APOL6 | 0 .325915285 | 7 .734501375 | 1 .90894 | 0 .06308 | 0 .240591911 |
| POU3F4 | -0 .337172422 | 6 . 153817629 | -1 .9085 | 0 .06314 | 0 .240676173 |
| GNG13 | -0 .746834173 | 4 .414485867 | -1 .9085 | 0 .06314 | 0 .240676173 |
| DUS2 | 0 .275257137 | 7 .866453932 | 1 .90833 | 0 .06316 | 0 .240681456 |
| SP110 | 0 .318897727 | 9 .782699013 | 1 .90755 | 0 .06327 | 0 .240938744 |
| ARHGAP35 | 0 .281568254 | 8 .776768893 | 1 .9075 | 0 .06327 | 0 .240938744 |
| GEMIN8 | -0 .367386182 | 4 .77350083 | -1 .9074 | 0 .06329 | 0 .240938744 |
| RCBTB2 | -0 .345571655 | 6 .872112203 | -1 .9072 | 0 .06331 | 0 .240938744 |
| ORAI3 | -0 .201613526 | 8 .524359226 | -1 .907 | 0 .06334 | 0 .240938744 |
| SRPK2 | -0 . 1992534 | 9 .722931952 | -1 .907 | 0 .06335 | 0 .240938744 |
| ETV3 | -0 .492219262 | 3 . 129209129 | -1 .9066 | 0 .0634 | 0 .241060259 |
| NUDT11 | -0 .462223712 | 5 .21993628 | -1 .9052 | 0 .06358 | 0 .241618376 |

| TTC28 | -0 .310154411 | 7 .741223182 | -1 .9052 | 0 .06358 | 0 .241618376 |
| --- | --- | --- | --- | --- | --- |
| GEM | -0 .396203141 | 7 .856072887 | -1 .9044 | 0 .06369 | 0 .241957118 |
| HNRNPUL2 | 0 .259306941 | 7 .707944526 | 1 .9041 | 0 .06373 | 0 .241958862 |
| KHDRBS2 | -0 .412684862 | 5 .212114696 | -1 .9041 | 0 .06373 | 0 .241958862 |
| RIPK2 | 0 . 187305579 | 7 .901013941 | 1 .90339 | 0 .06382 | 0 .242153842 |
| NHP2 | 0 . 191695055 | 10 .24136922 | 1 .90326 | 0 .06384 | 0 .242153842 |
| CLEC11A | 0 .446444543 | 8 .928661339 | 1 .90326 | 0 .06384 | 0 .242153842 |
| SMNDC1 | -0 .394106114 | 6 .708432176 | -1 .9027 | 0 .06392 | 0 .24235966 |
| FOXJ3 | -0 .227701556 | 7 .980485086 | -1 .9026 | 0 .06393 | 0 .24235966 |
| DDOST | 0 .254316798 | 10 .53486525 | 1 .90089 | 0 .06416 | 0 .2431064 |
| PIGP | -0 .405516064 | 8 .05797552 | -1 .9006 | 0 .06419 | 0 .2431064 |
| SERPINB9 | 0 .439127061 | 6 .873799022 | 1 .90059 | 0 .0642 | 0 .2431064 |
| DHRS11 | 0 .398436262 | 6 .912454618 | 1 .90052 | 0 .06421 | 0 .2431064 |
| GK3P | 0 .503310694 | 4 .078623818 | 1 .89967 | 0 .06432 | 0 .243468535 |
| NGF | 0 .362781749 | 5 .463937073 | 1 .89901 | 0 .06441 | 0 .243733796 |
| ST5 | 0 .267747118 | 8 .637518843 | 1 .89681 | 0 .06471 | 0 .244655931 |
| FMO6P | -0 .473816226 | 5 .290277176 | -1 .8968 | 0 .06471 | 0 .244655931 |
| CHST2 | 0 .28665673 | 7 .531917855 | 1 .89677 | 0 .06471 | 0 .244655931 |
| ZNF365 | 0 .396525027 | 5 .547559079 | 1 .8963 | 0 .06478 | 0 .244696948 |
| PDSS2 | -0 .450834684 | 5 .585004264 | -1 .8963 | 0 .06478 | 0 .244696948 |
| ERGIC2 | -0 .366445184 | 7 .632045055 | -1 .8963 | 0 .06478 | 0 .244696948 |
| MPST | -0 .228677863 | 8 .045415009 | -1 .896 | 0 .06482 | 0 .244755422 |
| SH3BGR | -0 .344187492 | 5 .793201485 | -1 .895 | 0 .06495 | 0 .24513884 |
| TRIM45 | -0 .337838835 | 5 .213918568 | -1 .8949 | 0 .06496 | 0 .24513884 |
| TMEM87A | -0 .296653647 | 9 .979836824 | -1 .8948 | 0 .06498 | 0 .24513884 |
| ZNF232 | -0 .352740696 | 6 .641844049 | -1 .8945 | 0 .06503 | 0 .245249544 |
| PTPN7 | 0 .299023421 | 7 .581945961 | 1 .89405 | 0 .06508 | 0 .245385976 |
| ENG | 0 .346162784 | 8 .462708582 | 1 .89351 | 0 .06516 | 0 .245555207 |
| LIFR | -0 .463081167 | 4 . 136725196 | -1 .8934 | 0 .06517 | 0 .245555207 |
| FUT2 | 0 .287382401 | 7 .318102013 | 1 .89317 | 0 .0652 | 0 .245617235 |
| UBE2J1 | 0 . 197765328 | 8 .970496123 | 1 .89276 | 0 .06526 | 0 .24575407 |
| REV3L | -0 .433538306 | 6 .811820637 | -1 .8924 | 0 .06531 | 0 .245876496 |
| GADD45GIP1 | 0 .226097979 | 7 .493386844 | 1 .89205 | 0 .06535 | 0 .245970232 |
| ARHGAP25 | 0 .415822265 | 8 .000303939 | 1 .89129 | 0 .06546 | 0 .246239135 |
| DCP2 | -0 .225503252 | 6 .811838133 | -1 .8912 | 0 .06546 | 0 .246239135 |
| GLMN | -0 .372579187 | 5 . 179274856 | -1 .8906 | 0 .06555 | 0 .246499891 |
| IFNGR2 | 0 . 185825735 | 9 .62801894 | 1 .88853 | 0 .06584 | 0 .247489002 |
| STOML2 | -0 .304165578 | 7 .637408224 | -1 .8882 | 0 .06589 | 0 .247539791 |
| MARK3 | 0 .230602611 | 8 . 161879083 | 1 .88815 | 0 .06589 | 0 .247539791 |
| ASNA1 | 0 .218464051 | 7 .814891133 | 1 .88787 | 0 .06593 | 0 .247611716 |
| HIF3A | -0 .442185264 | 6 .873647355 | -1 .8875 | 0 .06597 | 0 .247701716 |
| POU4F3 | 0 .47693489 | 4 . 105616423 | 1 .88617 | 0 .06616 | 0 .248306082 |
| KIAA0408 | 0 .48862695 | 2 .706773334 | 1 .88607 | 0 .06618 | 0 .248306082 |
| CD53 | 0 .375708967 | 9 .225587556 | 1 .88595 | 0 .06619 | 0 .248306082 |
| PTPN3 | -0 .242594665 | 6 .68458706 | -1 .8858 | 0 .06621 | 0 .248306082 |
| MMADHC | -0 .279241232 | 9 .219151188 | -1 .8852 | 0 .06629 | 0 .248464132 |
| PRRC2A | 0 .267588102 | 9 .718734587 | 1 .88522 | 0 .06629 | 0 .248464132 |
| ABI1 | -0 .250601989 | 8 .504365085 | -1 .8847 | 0 .06636 | 0 .248635493 |
| ATAD5 | 0 .392658141 | 5 .70250505 | 1 .88455 | 0 .06639 | 0 .24866377 |
| PTPRN | 0 .371639626 | 5 .417087709 | 1 .88416 | 0 .06644 | 0 .248752292 |
| CRYGA | 0 .419037713 | 5 .636683344 | 1 .88406 | 0 .06645 | 0 .248752292 |
| ZNF551 | -0 .295928831 | 5 .448149009 | -1 .8839 | 0 .06647 | 0 .248752292 |
| KIAA0556 | 0 .228444561 | 7 .739146147 | 1 .88361 | 0 .06652 | 0 .248853836 |
| PAPSS1 | -0 .242724569 | 9 .320578779 | -1 .8831 | 0 .06659 | 0 .249068241 |
| TRAF5 | 0 .306009686 | 7 .235884397 | 1 .88194 | 0 .06675 | 0 .249571515 |
| SSX1 | 0 .297300868 | 7 .03903277 | 1 .88159 | 0 .0668 | 0 .249677459 |

| PHC2 | 0 .306208539 | 9 .307063345 | 1 .88131 | 0 .06684 | 0 .249746839 |
| --- | --- | --- | --- | --- | --- |
| GADD45B | 0 .316861539 | 8 .95320694 | 1 .88101 | 0 .06688 | 0 .249746839 |
| SART1 | 0 .279503937 | 7 .933235269 | 1 .8809 | 0 .06689 | 0 .249746839 |
| TJP3 | -0 .433597723 | 5 .431567705 | -1 .8809 | 0 .0669 | 0 .249746839 |
| TUBA1B | 0 .221298058 | 14 .46891858 | 1 .88058 | 0 .06694 | 0 .249834307 |
| PGAM2 | -0 .704668808 | 3 .532892748 | -1 .8796 | 0 .06708 | 0 .250283353 |
| DNAH6 | 0 .578024818 | 4 .352201271 | 1 .87925 | 0 .06712 | 0 .250304212 |
| KCNJ1 | 0 .513890469 | 6 .257856677 | 1 .87925 | 0 .06712 | 0 .250304212 |
| ROGDI | 0 .243641207 | 8 . 167771901 | 1 .87894 | 0 .06717 | 0 .250387892 |
| PPP1R1A | -0 .562527871 | 6 . 165033998 | -1 .878 | 0 .06729 | 0 .250761317 |
| NMD3 | -0 .281298671 | 7 .678435719 | -1 .8779 | 0 .06731 | 0 .250761317 |
| APC2 | 0 .599544834 | 6 .465362334 | 1 .87765 | 0 .06735 | 0 .250838726 |
| ASAP2 | -0 .406498451 | 6 .82903055 | -1 .8773 | 0 .0674 | 0 .250969712 |
| NCKIPSD | 0 .207684469 | 8 .314516651 | 1 .87688 | 0 .06746 | 0 .251019409 |
| SGPP1 | -0 .36079996 | 5 .228177761 | -1 .8769 | 0 .06746 | 0 .251019409 |
| ZNF544 | -0 . 19332461 | 7 .810032965 | -1 .8766 | 0 .0675 | 0 .251019409 |
| ZC3H4 | -0 .314903203 | 8 .786592535 | -1 .8766 | 0 .0675 | 0 .251019409 |
| C16orf45 | -0 .29581488 | 7 .046405772 | -1 .8762 | 0 .06755 | 0 .251128027 |
| UBE2K | 0 .335016386 | 8 .640307542 | 1 .87585 | 0 .0676 | 0 .251151065 |
| SYTL2 | 0 .24085951 | 5 .789800295 | 1 .87579 | 0 .06761 | 0 .251151065 |
| SPINK5 | 0 .714141523 | 9 .69075369 | 1 .87577 | 0 .06761 | 0 .251151065 |
| ADCK3 | -0 .288904698 | 7 .206708743 | -1 .8752 | 0 .0677 | 0 .251397772 |
| DDRGK1 | 0 .285690348 | 8 .882120422 | 1 .87446 | 0 .0678 | 0 .251688895 |
| CYTH1 | 0 .249523912 | 7 .21012542 | 1 .87423 | 0 .06783 | 0 .251732018 |
| NAP1L3 | -0 .364737989 | 5 .0445539 | -1 .8737 | 0 .0679 | 0 .251753126 |
| CCR10 | 0 .268195094 | 6 .016654795 | 1 .8737 | 0 .0679 | 0 .251753126 |
| EIF4G1 | 0 .262166927 | 10 . 10103683 | 1 .87365 | 0 .06791 | 0 .251753126 |
| ACR | -0 .402539458 | 5 . 174010657 | -1 .8735 | 0 .06793 | 0 .251753126 |
| ACTR6 | -0 .391781439 | 5 .60004557 | -1 .8735 | 0 .06793 | 0 .251753126 |
| STRN | -0 .291957239 | 6 .75096881 | -1 .8732 | 0 .06797 | 0 .251757365 |
| VPS8 | -0 .284652363 | 7 .392262394 | -1 .8732 | 0 .06798 | 0 .251757365 |
| CA5B | -0 .465964468 | 6 . 107778198 | -1 .8726 | 0 .06806 | 0 .251995235 |
| JADE3 | 0 .36198796 | 5 .904979003 | 1 .87197 | 0 .06815 | 0 .252247514 |
| ORC3 | 0 .304493908 | 6 .878507787 | 1 .87145 | 0 .06822 | 0 .252373793 |
| NPDC1 | -0 .378572225 | 7 .778223242 | -1 .8714 | 0 .06823 | 0 .252373793 |
| IGKC | 1 .259851984 | 10 .27859855 | 1 .8713 | 0 .06824 | 0 .252373793 |
| MICU1 | 0 . 167083517 | 8 .779272081 | 1 .87064 | 0 .06834 | 0 .252649621 |
| MDM2 | 0 .454673512 | 7 . 157098534 | 1 .87038 | 0 .06837 | 0 .252707388 |
| NBEAL2 | 0 .383460215 | 6 .373942262 | 1 .86975 | 0 .06846 | 0 .252964785 |
| TNN | -0 .573868959 | 4 .316088555 | -1 .8692 | 0 .06855 | 0 .253199285 |
| B3GNT2 | -0 .367085909 | 6 .40119804 | -1 .8689 | 0 .06858 | 0 .253246246 |
| C5orf22 | 0 .442695588 | 7 .089295859 | 1 .86868 | 0 .06861 | 0 .253304103 |
| RDH8 | 0 .299501557 | 6 .765952463 | 1 .86841 | 0 .06865 | 0 .253310496 |
| THOC7 | -0 .300149608 | 7 .990785304 | -1 .8684 | 0 .06866 | 0 .253310496 |
| MSRB1 | -0 .314900327 | 8 .349601985 | -1 .8674 | 0 .06879 | 0 .25373488 |
| STK10 | 0 .280913658 | 9 .092224177 | 1 .86677 | 0 .06889 | 0 .254013656 |
| NPIPB15 | 0 .302166742 | 6 . 170302192 | 1 .86602 | 0 .06899 | 0 .254331531 |
| SLC12A9 | 0 . 185858667 | 7 .530031462 | 1 .86583 | 0 .06902 | 0 .254359754 |
| BACE2 | 0 .413974127 | 10 .29016656 | 1 .86516 | 0 .06912 | 0 .254634805 |
| TAB2 | -0 .253448796 | 8 .064224605 | -1 .8644 | 0 .06922 | 0 .254869846 |
| IGLJ3 | 1 .427088439 | 8 . 187751318 | 1 .86404 | 0 .06928 | 0 .254869846 |
| ACO1 | -0 .333251418 | 7 .651429348 | -1 .8639 | 0 .0693 | 0 .254869846 |
| CCT6A | 0 .299368272 | 9 .377644732 | 1 .86387 | 0 .0693 | 0 .254869846 |
| ZNF862 | -0 .370932661 | 5 .835906504 | -1 .8638 | 0 .06931 | 0 .254869846 |
| OPA1 | -0 .218838105 | 8 .705288535 | -1 .8637 | 0 .06932 | 0 .254869846 |
| NDUFB7 | -0 .353993394 | 7 . 154009264 | -1 .8637 | 0 .06932 | 0 .254869846 |

| SRSF5 | -0 .269563955 | 10 .21878763 | -1 .8633 | 0 .06939 | 0 .255047847 |
| --- | --- | --- | --- | --- | --- |
| PARP4 | 0 .286110862 | 8 .414259243 | 1 .86298 | 0 .06943 | 0 .255084426 |
| TRPC5 | -0 .697826673 | 2 .858522518 | -1 .8628 | 0 .06945 | 0 .255084426 |
| EDNRA | -0 .38829709 | 8 .099447719 | -1 .8626 | 0 .06949 | 0 .255084426 |
| ALMS1 | -0 .403245106 | 9 .443769211 | -1 .8624 | 0 .06952 | 0 .255084426 |
| PIAS4 | 0 .232863167 | 7 .660758094 | 1 .86235 | 0 .06952 | 0 .255084426 |
| TGFB3 | 0 .259827765 | 6 .929223826 | 1 .86232 | 0 .06953 | 0 .255084426 |
| ZXDC | -0 .395067166 | 5 .276629131 | -1 .8622 | 0 .06954 | 0 .255084426 |
| APBB1IP | 0 .391292007 | 5 .037220327 | 1 .86159 | 0 .06963 | 0 .255310425 |
| C3orf18 | -0 .478257129 | 4 .768740379 | -1 .8615 | 0 .06965 | 0 .255310425 |
| EHBP1L1 | 0 .228916363 | 7 .234682934 | 1 .8609 | 0 .06973 | 0 .255544929 |
| NIT2 | -0 .260728698 | 8 .067621904 | -1 .8606 | 0 .06978 | 0 .255648855 |
| RCOR3 | -0 .356672394 | 5 .67122558 | -1 .8604 | 0 .06981 | 0 .255674921 |
| SEC61B | 0 .225044323 | 10 .29543028 | 1 .85862 | 0 .07006 | 0 .256463791 |
| MSANTD2 | -0 .330919738 | 5 .660217212 | -1 .8586 | 0 .07006 | 0 .256463791 |
| PLA2G2D | 0 .705842314 | 4 .892889114 | 1 .85785 | 0 .07017 | 0 .25678952 |
| TMF1 | -0 . 176513485 | 8 .37590351 | -1 .8574 | 0 .07024 | 0 .256972333 |
| HNF1A | 0 .262731814 | 6 .367597851 | 1 .85672 | 0 .07034 | 0 .257178522 |
| NDUFB5 | -0 .212262342 | 9 .00676511 1 | -1 .8567 | 0 .07034 | 0 .257178522 |
| FHL5 | -0 .399294844 | 5 .485258835 | -1 .8559 | 0 .07046 | 0 .257537023 |
| MMP20 | -0 .49163052 | 3 .925088715 | -1 .8556 | 0 .0705 | 0 .257605828 |
| RPL14 | -0 .202182047 | 12 .5626346 | -1 .8552 | 0 .07056 | 0 .257746193 |
| KLKB1 | 0 .288611353 | 5 .528950805 | 1 .85491 | 0 .0706 | 0 .25782478 |
| TM9SF4 | 0 . 135797936 | 8 .753943573 | 1 .85437 | 0 .07068 | 0 .258042104 |
| SOAT2 | 0 .374672907 | 4 .623319115 | 1 .85354 | 0 .0708 | 0 .258405501 |
| MAGIX | -0 .479032155 | 3 .60628734 | -1 .8532 | 0 .07086 | 0 .258532226 |
| NFKBIA | -0 .396879121 | 10 .01175028 | -1 .8524 | 0 .07097 | 0 .25886137 |
| AGPAT5 | 0 .360760212 | 7 .653371433 | 1 .85223 | 0 .07099 | 0 .258879044 |
| OSER1 | -0 .218074495 | 7 .93067752 | -1 .8518 | 0 .07106 | 0 .259043087 |
| EIF3H | -0 .26158225 | 10 .58601626 | -1 .8515 | 0 .07109 | 0 .259101743 |
| NUP54 | -0 .265916998 | 7 .206541644 | -1 .8513 | 0 .07113 | 0 .259175093 |
| CREBBP | -0 .229778439 | 7 .745912084 | -1 .8506 | 0 .07123 | 0 .259440312 |
| NPC1L1 | 0 .378869999 | 5 .860736674 | 1 .84991 | 0 .07133 | 0 .259744727 |
| JOSD1 | 0 .335221081 | 7 .502916394 | 1 .84958 | 0 .07138 | 0 .259847314 |
| GZMH | 0 .57566961 | 5 .444552895 | 1 .84866 | 0 .07152 | 0 .260265969 |
| DYM | -0 .475265725 | 3 .951853803 | -1 .8478 | 0 .07164 | 0 .260635209 |
| TP73-AS1 | -0 .23641166 | 7 .797106629 | -1 .8469 | 0 .07177 | 0 .26104966 |
| CCHCR1 | 0 .203240029 | 8 .496043942 | 1 .84639 | 0 .07185 | 0 .261262701 |
| LDLR | 0 .298600808 | 8 .8481129 | 1 .84525 | 0 .07202 | 0 .261798763 |
| CDC42 | -0 .313698624 | 10 .44455005 | -1 .8451 | 0 .07204 | 0 .261798763 |
| ARHGEF9 | -0 .259821717 | 6 .990050327 | -1 .8447 | 0 .07211 | 0 .261916364 |
| ZNF174 | 0 .423844079 | 7 .490144705 | 1 .84461 | 0 .07212 | 0 .261916364 |
| DDB1 | -0 .224814066 | 9 .309966985 | -1 .8441 | 0 .07219 | 0 .262096741 |
| MRPL18 | 0 .276239495 | 8 .631823231 | 1 .84386 | 0 .07223 | 0 .262096741 |
| TBX21 | 0 .344443636 | 6 . 153063722 | 1 .84386 | 0 .07223 | 0 .262096741 |
| CST2 | 0 .2611 19341 | 6 .3207804 | 1 .84259 | 0 .07242 | 0 .262706505 |
| NFE2L3 | 0 . 187720113 | 6 .986786314 | 1 .84233 | 0 .07246 | 0 .262767127 |
| C1orf216 | 0 .315020014 | 7 .75195786 | 1 .84211 | 0 .07249 | 0 .262810533 |
| KLF4 | -0 .409109741 | 9 .407880439 | -1 .8415 | 0 .07258 | 0 .263008096 |
| PTGDR | 0 .253249958 | 5 .367341759 | 1 .84142 | 0 .07259 | 0 .263008096 |
| SOX12 | 0 .352264823 | 5 .496031964 | 1 .84129 | 0 .07261 | 0 .263008096 |
| HCK | 0 .442904076 | 6 .794927835 | 1 .84119 | 0 .07263 | 0 .263008096 |
| C16orf80 | -0 .27161711 | 7 .72571614 | -1 .8409 | 0 .07267 | 0 .263059438 |
| SI | 0 .582340081 | 3 . 1 18862525 | 1 .84081 | 0 .07268 | 0 .263059438 |
| SMG9 | -0 .290316698 | 6 .62391861 | -1 .8406 | 0 .07271 | 0 .263071558 |
| EXOC5 | -0 .514816232 | 4 .607144071 | -1 .8405 | 0 .07273 | 0 .263076426 |

| AFG3L2 | 0 . 187134322 | 8 .37532717 | 1 .83989 | 0 .07282 | 0 .26325623 |
| --- | --- | --- | --- | --- | --- |
| SLC22A3 | -0 .4607497 | 3 .025359063 | -1 .8399 | 0 .07282 | 0 .26325623 |
| BHLHB9 | 0 .274167051 | 5 .768035358 | 1 .83973 | 0 .07284 | 0 .263265091 |
| GUSBP11 | 1 .240198485 | 8 .991737717 | 1 .83943 | 0 .07289 | 0 .263352077 |
| MYCL | -0 .279348527 | 7 .6623591 | -1 .8391 | 0 .07293 | 0 .263431435 |
| PAICS | 0 .342356828 | 9 .470902751 | 1 .83829 | 0 .07306 | 0 .263802134 |
| C7 | -0 .744829522 | 4 .570051655 | -1 .8382 | 0 .07308 | 0 .263802134 |
| IRX4 | -0 .462252333 | 6 .861047973 | -1 .8379 | 0 .07312 | 0 .263876037 |
| LGALS13 | -0 .736591909 | 4 .31944107 | -1 .8372 | 0 .07322 | 0 .264176209 |
| TMBIM4 | -0 .408148622 | 9 .245749309 | -1 .837 | 0 .07325 | 0 .264196965 |
| NOL9 | -0 . 158363333 | 7 .8364637 | -1 .8366 | 0 .07332 | 0 .264361063 |
| TRIM68 | -0 . 171743553 | 6 .999579011 | -1 .8359 | 0 .07342 | 0 .264653576 |
| LPAR2 | -0 .322784488 | 6 .65876763 | -1 .8352 | 0 .07353 | 0 .264963426 |
| WDR61 | -0 .221751254 | 9 .025471958 | -1 .8349 | 0 .07358 | 0 .265068026 |
| STRADA | 0 . 16954406 | 9 .320730546 | 1 .8347 | 0 .0736 | 0 .265083704 |
| KLF15 | 0 .272752928 | 6 .495299254 | 1 .83395 | 0 .07372 | 0 .265417849 |
| ZFC3H1 | 0 .219791785 | 7 .980294379 | 1 .8335 | 0 .07378 | 0 .265533709 |
| CDC42BPA | 0 .228809473 | 9 .229033265 | 1 .83346 | 0 .07379 | 0 .265533709 |
| PTGFR | -0 .418866511 | 5 .08540967 | -1 .8322 | 0 .07398 | 0 .266133686 |
| SFRP4 | -0 .325570043 | 6 .203984458 | -1 .8318 | 0 .07404 | 0 .266281492 |
| MYO15B | -0 .231030048 | 8 . 163550248 | -1 .8312 | 0 .07414 | 0 .26655884 |
| PSMB8 | 0 .531277529 | 8 .048995872 | 1 .83097 | 0 .07417 | 0 .266586902 |
| COL7A1 | -0 .43401988 | 8 . 178515736 | -1 .8307 | 0 .0742 | 0 .266641189 |
| PSMD8 | 0 .293373611 | 9 .595296848 | 1 .83034 | 0 .07426 | 0 .266779571 |
| RRAGC | -0 .209188938 | 8 .802742838 | -1 .8296 | 0 .07437 | 0 .267098867 |
| KLHL7 | 0 .297238246 | 6 . 150410698 | 1 .82847 | 0 .07455 | 0 .267652367 |
| MSX1 | 0 .201873088 | 7 .520541697 | 1 .82784 | 0 .07464 | 0 .267757312 |
| AKR1D1 | 0 .319990128 | 4 .831933422 | 1 .82779 | 0 .07465 | 0 .267757312 |
| SEMA4F | 0 .235896413 | 7 .861802278 | 1 .82778 | 0 .07465 | 0 .267757312 |
| WHSC1 | 0 .214599719 | 7 .208602508 | 1 .82764 | 0 .07468 | 0 .267757312 |
| WIPF1 | 0 .284717069 | 8 .827970694 | 1 .82758 | 0 .07469 | 0 .267757312 |
| PITPNM3 | 0 .523764045 | 2 .608437935 | 1 .82719 | 0 .07475 | 0 .267895169 |
| IFI35 | 0 .460286921 | 7 .59149654 | 1 .8269 | 0 .07479 | 0 .267968987 |
| NXN | -0 .316660032 | 8 .807314655 | -1 .8268 | 0 .07481 | 0 .267968987 |
| PTPRN2 | -0 .391572895 | 6 .466847994 | -1 .8264 | 0 .07486 | 0 .268091161 |
| DCTN1 | 0 .26772042 | 9 .079670186 | 1 .82615 | 0 .0749 | 0 .26815798 |
| CNIH3 | 0 .924170678 | 5 .307381341 | 1 .82585 | 0 .07495 | 0 .268245464 |
| FRMD8 | 0 .607627595 | 5 .045190485 | 1 .82514 | 0 .07506 | 0 .268561468 |
| CRK | -0 .202143827 | 9 .249275817 | -1 .8244 | 0 .07518 | 0 .268904752 |
| PRKCSH | 0 .224459514 | 1 1 . 19903464 | 1 .82364 | 0 .07529 | 0 .269158364 |
| BBOX1 | -0 .617869529 | 6 .690991988 | -1 .8236 | 0 .07529 | 0 .269158364 |
| HAPLN1 | 0 .355045947 | 4 .580828487 | 1 .82307 | 0 .07538 | 0 .269171722 |
| CLEC1A | -0 .359555712 | 6 .868608518 | -1 .8231 | 0 .07538 | 0 .269171722 |
| STYK1 | 0 .32097007 | 5 .820991238 | 1 .82297 | 0 .07539 | 0 .269171722 |
| PLEKHB1 | 0 .613531929 | 6 .760850406 | 1 .82291 | 0 .0754 | 0 .269171722 |
| EPS8 | -0 .501511325 | 7 .499857526 | -1 .8229 | 0 .0754 | 0 .269171722 |
| HUWE1 | -0 . 165838836 | 12 .65112349 | -1 .8228 | 0 .07543 | 0 .269171722 |
| RTF1 | -0 .245026245 | 8 .287405171 | -1 .8224 | 0 .07548 | 0 .269171722 |
| TUBA1C | 0 .222279443 | 13 .03160891 | 1 .8223 | 0 .0755 | 0 .269171722 |
| FOXE3 | -0 .731555628 | 4 .427704422 | -1 .8223 | 0 .0755 | 0 .269171722 |
| CSNK2A1 | 0 .348165308 | 9 .781543856 | 1 .82218 | 0 .07552 | 0 .269171722 |
| DHX32 | -0 .208362732 | 7 .699284512 | -1 .8219 | 0 .07555 | 0 .269171722 |
| FAM222B | 0 .33827249 | 8 .21090387 | 1 .8219 | 0 .07556 | 0 .269171722 |
| SEPHS1 | -0 .220911444 | 8 . 134757806 | -1 .8218 | 0 .07558 | 0 .269171722 |
| SH2D2A | 0 .396695093 | 6 .546580025 | 1 .82157 | 0 .07561 | 0 .269171722 |
| IKBKAP | -0 .316337437 | 7 .35243038 | -1 .8215 | 0 .07562 | 0 .269171722 |

| SETBP1 | -0 .363808392 | 6 .588810028 | -1 .8205 | 0 .07577 | 0 .269639992 |
| --- | --- | --- | --- | --- | --- |
| LAMA4 | -0 .305013674 | 8 .948296082 | -1 .8203 | 0 .07581 | 0 .269713806 |
| RAP1GAP | 0 .207685345 | 8 .779725059 | 1 .81861 | 0 .07607 | 0 .270507985 |
| PRPSAP1 | -0 . 18425918 | 8 .277019493 | -1 .8186 | 0 .07608 | 0 .270507985 |
| SMAD1 | -0 .43290707 | 7 .68187408 | -1 .8181 | 0 .07615 | 0 .270649611 |
| USP1 | -0 .351332194 | 8 .477950669 | -1 .818 | 0 .07616 | 0 .270649611 |
| C5orf28 | -0 .411940327 | 5 . 180096813 | -1 .8176 | 0 .07623 | 0 .270764921 |
| OAS1 | 0 .5485425 | 8 .366419472 | 1 .81753 | 0 .07624 | 0 .270764921 |
| CD44 | -0 .300963043 | 13 .06970308 | -1 .8171 | 0 .0763 | 0 .270845247 |
| TTI1 | 0 .274183598 | 6 .818633143 | 1 .81711 | 0 .0763 | 0 .270845247 |
| GGNBP2 | -0 .248665319 | 7 .387549453 | -1 .8169 | 0 .07633 | 0 .270849369 |
| POGZ | -0 .332488842 | 8 . 105926985 | -1 .8168 | 0 .07635 | 0 .270849369 |
| C17orf59 | -0 .287760104 | 6 . 152612946 | -1 .8167 | 0 .07637 | 0 .270849369 |
| LOC100506603 | 0 .217931877 | 6 .049351332 | 1 .81655 | 0 .07639 | 0 .270849369 |
| FRYL | -0 .315996598 | 8 .512343592 | -1 .8156 | 0 .07654 | 0 .271288478 |
| EMILIN1 | 0 .6020387 | 6 .029665407 | 1 .81548 | 0 .07656 | 0 .271288478 |
| F11 | 0 .285874608 | 5 .411937781 | 1 .81526 | 0 .07659 | 0 .271317194 |
| LSM3 | -0 .268594333 | 8 . 103560932 | -1 .8151 | 0 .07661 | 0 .271317194 |
| PSMD11 | 0 . 176904001 | 9 . 106904508 | 1 .81492 | 0 .07664 | 0 .271369165 |
| ELAVL2 | -0 .467516048 | 4 .804527668 | -1 .8144 | 0 .07673 | 0 .271575478 |
| PSMD3 | 0 .274966511 | 8 .88682547 | 1 .81427 | 0 .07675 | 0 .271575478 |
| C1RL | -0 .33138147 | 6 .277319775 | -1 .8136 | 0 .07685 | 0 .271868779 |
| PEX14 | 0 .228778185 | 7 .788152939 | 1 .81231 | 0 .07705 | 0 .272469728 |
| APP | -0 .220287668 | 1 1 .45306723 | -1 .8122 | 0 .07706 | 0 .272469728 |
| MRPS12 | 0 . 169869683 | 8 .435414346 | 1 .81142 | 0 .07719 | 0 .272851537 |
| MAFG | 0 .286394009 | 8 . 163212173 | 1 .81121 | 0 .07723 | 0 .27288937 |
| PPAP2A | -0 .27292587 | 9 .408756033 | -1 .8108 | 0 .07729 | 0 .27302765 |
| PHYH | -0 .239311 123 | 7 . 128069968 | -1 .8098 | 0 .07744 | 0 .273494233 |
| NDUFAF7 | 0 .578382689 | 3 .545641001 | 1 .80947 | 0 .0775 | 0 .273600533 |
| SEMA5A | -0 .282647922 | 8 .562063906 | -1 .8094 | 0 .07751 | 0 .273600533 |
| APPL1 | -0 .241722179 | 6 .98586882 | -1 .8091 | 0 .07755 | 0 .273661031 |
| SMURF2 | -0 .289024335 | 7 .453261817 | -1 .8088 | 0 .07761 | 0 .273778404 |
| CCDC71 | 0 .226441523 | 6 .691668502 | 1 .80847 | 0 .07766 | 0 .273877568 |
| IFT46 | -0 .314194869 | 7 .404033779 | -1 .808 | 0 .07773 | 0 .273993259 |
| EFNB2 | -0 .41475374 | 8 .773363254 | -1 .808 | 0 .07773 | 0 .273993259 |
| LOXL1 | 0 .432737414 | 7 .865490476 | 1 .80758 | 0 .0778 | 0 .274145785 |
| ITGA2 | -0 .350165963 | 7 .048656875 | -1 .8073 | 0 .07784 | 0 .274225667 |
| ANO2 | -0 .371269525 | 4 .678721715 | -1 .8068 | 0 .07793 | 0 .274438797 |
| PPA2 | -0 .294354449 | 8 .064599008 | -1 .8064 | 0 .07799 | 0 .274585129 |
| RP3-522P13 .2 | 0 .373211813 | 5 .908525821 | 1 .80619 | 0 .07802 | 0 .274612395 |
| DUSP10 | 0 .267873193 | 7 .588943941 | 1 .805 | 0 .07821 | 0 .275137596 |
| NOP16 | 0 .222706795 | 9 .021239557 | 1 .80498 | 0 .07821 | 0 .275137596 |
| SET | -0 .223850602 | 10 .21186909 | -1 .8047 | 0 .07825 | 0 .275203585 |
| PLIN2 | -0 .588664748 | 8 .236044761 | -1 .8041 | 0 .07835 | 0 .27543994 |
| CTNNA2 | 0 .544026777 | 4 .701818265 | 1 .80394 | 0 .07838 | 0 .27543994 |
| BTF3 | -0 .207616221 | 12 .57032076 | -1 .8039 | 0 .07839 | 0 .27543994 |
| ZNF207 | 0 .211 1 19531 | 10 .23000674 | 1 .80349 | 0 .07845 | 0 .275577116 |
| MEF2D | 0 .377258365 | 5 .981079703 | 1 .80336 | 0 .07847 | 0 .275577116 |
| MKKS | -0 .249534446 | 7 .548196958 | -1 .8031 | 0 .07851 | 0 .275636455 |
| DIAPH1 | 0 . 17168866 | 9 .893249615 | 1 .80158 | 0 .07876 | 0 .2764246 |
| TPTE | 0 .548976142 | 5 .314049878 | 1 .8014 | 0 .07878 | 0 .276448702 |
| SULT1E1 | -0 .46626284 | 4 .068095797 | -1 .8012 | 0 .07881 | 0 .276468017 |
| POLR2D | 0 .295909751 | 8 . 185708859 | 1 .80092 | 0 .07886 | 0 .276563608 |
| ERLIN1 | -0 .264790691 | 8 .251519595 | -1 .7999 | 0 .07902 | 0 .277052589 |
| RITA1 | 0 .25231021 | 7 .884883247 | 1 .79972 | 0 .07905 | 0 .277080544 |
| GPM6B | -0 .536060408 | 1 1 .62608363 | -1 .7994 | 0 .07911 | 0 .277190889 |

| LSM7 0 .376707143 | 7 .755162153 | 1 .79906 | 0 .07916 | 0 .277296861 |
| --- | --- | --- | --- | --- |
| QRSL1 -0 . 185908614 | 7 .367281884 | -1 .7986 | 0 .07924 | 0 .277462023 |
| CD27 0 .560388175 | 7 .438160346 | 1 .7985 | 0 .07925 | 0 .277462023 |
| TRAPPC12 -0 . 183438204 | 7 .728547411 | -1 .7983 | 0 .07927 | 0 .277470672 |
| EBP -0 .350204004 | 8 .595150327 | -1 .798 | 0 .07933 | 0 .277578684 |
| TNRC6B -0 .274375835 | 7 .904688043 | -1 .7977 | 0 .07938 | 0 .277673048 |
| THTPA -0 .305182812 | 6 .809474884 | -1 .7968 | 0 .07953 | 0 .278103209 |
| BCCIP -0 .24726082 | 6 .252779402 | -1 .7967 | 0 .07954 | 0 .278103209 |
| IGLC1 1 .473064479 | 10 .97802509 | 1 .79538 | 0 .07975 | 0 .278644876 |
| MPP5 -0 .332913542 | 4 .754688641 | -1 .7954 | 0 .07976 | 0 .278644876 |
| PCDHGA10 -0 .505953076 | 3 .894421696 | -1 .7951 | 0 .0798 | 0 .278644876 |
| CNTD2 0 .289839715 | 8 .534544269 | 1 .79482 | 0 .07984 | 0 .278644876 |
| KIF24 0 .36302851 | 5 . 1 15265554 | 1 .79446 | 0 .0799 | 0 .278644876 |
| ANXA2P3 0 .328751452 | 7 .697193715 | 1 .79442 | 0 .07991 | 0 .278644876 |
| SF3B4 0 .278434308 | 8 .424171061 | 1 .79432 | 0 .07992 | 0 .278644876 |
| NRP1 -0 .265487669 | 7 .376710412 | -1 .7943 | 0 .07993 | 0 .278644876 |
| SERPINH1 0 .515342371 | 8 . 187257215 | 1 .79423 | 0 .07994 | 0 .278644876 |
| FCGR1A 0 .424272454 | 4 .430079057 | 1 .79421 | 0 .07994 | 0 .278644876 |
| FMO1 0 .473576234 | 7 .027296093 | 1 .7942 | 0 .07994 | 0 .278644876 |
| DNAJB2 -0 .300818511 | 7 .826353136 | -1 .794 | 0 .07997 | 0 .278679075 |
| UFL1 -0 .286796358 | 7 .700241067 | -1 .7938 | 0 .08001 | 0 .27870692 |
| SMG5 0 .539333865 | 7 .448095945 | 1 .79368 | 0 .08003 | 0 .27870692 |
| FASN -0 .61423853 | 9 .344463615 | -1 .7924 | 0 .08024 | 0 .279353467 |
| SLC25A24 -0 .459347273 | 6 .964074003 | -1 .7917 | 0 .08036 | 0 .279698994 |
| TSPAN31 -0 .236378367 | 8 .801738995 | -1 .7914 | 0 .08039 | 0 .279742522 |
| ECSIT -0 .341587297 | 6 .770635074 | -1 .7906 | 0 .08053 | 0 .280161094 |
| GRAMD3 -0 .477194029 | 7 .082566035 | -1 .7902 | 0 .08059 | 0 .280235131 |
| SLC4A8 0 .397823668 | 4 .034586496 | 1 .79009 | 0 .08061 | 0 .280235131 |
| EPHA7 0 .760892109 | 4 .49717304 | 1 .79003 | 0 .08062 | 0 .280235131 |
| ODF2 0 .546772074 | 5 .242524227 | 1 .78967 | 0 .08068 | 0 .28035731 |
| UBQLN2 -0 .360684152 | 7 .478750292 | -1 .7883 | 0 .08091 | 0 .281072099 |
| TNFSF10 -0 .470060137 | 9 . 1 16759482 | -1 .788 | 0 .08095 | 0 .281126586 |
| IFITM1 0 .432027233 | 1 1 .22168399 | 1 .78791 | 0 .08097 | 0 .281126586 |
| ZYX 0 .243189773 | 10 . 10836726 | 1 .78747 | 0 .08104 | 0 .281281098 |
| ABCG4 0 .384929245 | 6 .063563351 | 1 .78737 | 0 .08106 | 0 .281281098 |
| NOL11 -0 .265305565 | 7 .958965961 | -1 .7872 | 0 .08109 | 0 .281314637 |
| MGAT5 -0 .297653878 | 9 .446975999 | -1 .7869 | 0 .08113 | 0 .281345482 |
| DHX57 -0 .276226724 | 5 .749206374 | -1 .7868 | 0 .08115 | 0 .281345482 |
| TFIP11 0 . 180837343 | 8 .325822306 | 1 .78671 | 0 .08117 | 0 .281345482 |
| MVB12B -0 . 188788917 | 8 .543358839 | -1 .7865 | 0 .0812 | 0 .281367604 |
| PTGER2 -0 .379259046 | 4 .343712882 | -1 .7864 | 0 .08122 | 0 .281367604 |
| HMGCL -0 . 195748018 | 7 .810607534 | -1 .7854 | 0 .08138 | 0 .281849352 |
| RAB1B 0 .222368715 | 10 .30323321 | 1 .78491 | 0 .08146 | 0 .282056446 |
| MANEA -0 .387584986 | 4 .447701213 | -1 .7847 | 0 .0815 | 0 .282108893 |
| NLK 0 . 185890754 | 6 .047673241 | 1 .78375 | 0 .08165 | 0 .282520903 |
| UBE2E3 -0 .418194866 | 9 .342948206 | -1 .7837 | 0 .08166 | 0 .282520903 |
| RP11-499E18 . 1 -0 .26676163 | 5 .933659677 | -1 .7833 | 0 .08172 | 0 .282658031 |
| PBRM1 -0 .26864288 | 7 .968868064 | -1 .783 | 0 .08177 | 0 .282745867 |
| GPN1 -0 . 187489816 | 8 .24605238 | -1 .7829 | 0 .0818 | 0 .28274891 |
| TAF2 0 .313115187 | 6 .598216078 | 1 .78274 | 0 .08182 | 0 .28274891 |
| IPO5 -0 .292495459 | 9 .862835012 | -1 .7824 | 0 .08188 | 0 .282870136 |
| KCNA2 0 .670058092 | 3 .315055059 | 1 .78131 | 0 .08206 | 0 .283412455 |
| VWA9 -0 .205823458 | 7 . 164740411 | -1 .7803 | 0 .08223 | 0 .283929185 |
| LYRM9 -0 .300722828 | 7 . 156434558 | -1 .78 | 0 .08227 | 0 .283986402 |
| TRPC2 0 .375922083 | 6 . 175989357 | 1 .77958 | 0 .08234 | 0 .284171488 |
| SLCO1B3 0 .471863372 | 3 .784951974 | 1 .77942 | 0 .08237 | 0 .28418345 |

| ABI3BP | -0 .458467483 | 4 .036287513 | -1 .7791 | 0 .08243 | 0 .284306835 |
| --- | --- | --- | --- | --- | --- |
| FAM69A | 0 .241960812 | 8 . 132879696 | 1 .77882 | 0 .08247 | 0 .284338711 |
| RASAL1 | 0 .262656885 | 7 .626995419 | 1 .7787 | 0 .08249 | 0 .284338711 |
| SERP1 | -0 .307602797 | 7 .567489744 | -1 .7786 | 0 .08251 | 0 .284338711 |
| SEL1L3 | 0 .350864987 | 7 .676387594 | 1 .77838 | 0 .08254 | 0 .284349474 |
| ZNF107 | -0 .334288073 | 4 .400131095 | -1 .7783 | 0 .08256 | 0 .284349474 |
| CORO1B | 0 .24382434 | 9 .392662363 | 1 .77818 | 0 .08258 | 0 .284349474 |
| MEGF9 | -0 .398640483 | 8 .731741541 | -1 .7773 | 0 .08272 | 0 .284720468 |
| SLC38A10 | 0 .234948792 | 8 .81758953 | 1 .77726 | 0 .08273 | 0 .284720468 |
| ABI2 | 0 . 193099425 | 8 .653482332 | 1 .77698 | 0 .08278 | 0 .284798077 |
| EI24 | -0 .244935494 | 9 .990076039 | -1 .7769 | 0 .0828 | 0 .284798077 |
| SLC22A1 | 0 .43359427 | 4 .392242799 | 1 .77664 | 0 .08283 | 0 .284841547 |
| GNG10 | -0 .372865757 | 9 .024725324 | -1 .7758 | 0 .08298 | 0 .285260679 |
| EHD2 | -0 .270287513 | 8 .555120645 | -1 .7754 | 0 .08304 | 0 .28538899 |
| NBAS | -0 .282079338 | 7 .251622441 | -1 .7751 | 0 .08309 | 0 .285479769 |
| HHLA1 | 0 .510635355 | 4 .919735256 | 1 .7748 | 0 .08314 | 0 .2855888 |
| MAGEA1 | 0 .783723314 | 3 .704353328 | 1 .77397 | 0 .08328 | 0 .285983913 |
| SAT1 | 0 .314712036 | 1 1 .99565091 | 1 .77382 | 0 .08331 | 0 .285995512 |
| GJC1 | 0 .327323684 | 6 .995369403 | 1 .77358 | 0 .08334 | 0 .286052043 |
| KCNAB3 | 0 .422077552 | 4 .735166728 | 1 .77323 | 0 .0834 | 0 .2861101 |
| CYTIP | 0 .667022028 | 6 .273400752 | 1 .77321 | 0 .08341 | 0 .2861101 |
| UBXN4 | -0 .229943884 | 9 .713526475 | -1 .7727 | 0 .08349 | 0 .286316207 |
| CD3E | 0 .566455394 | 7 . 136394244 | 1 .77238 | 0 .08355 | 0 .286433253 |
| SLC22A17 | -0 .336704807 | 7 .01783421 | -1 .7721 | 0 .08359 | 0 .286488287 |
| ERV9-1 | 0 .311224748 | 5 .760046477 | 1 .7713 | 0 .08373 | 0 .286860355 |
| PFKM | -0 .31000121 | 9 . 137261093 | -1 .7712 | 0 .08374 | 0 .286860355 |
| TRIM58 | 0 .679978772 | 2 .874050824 | 1 .77084 | 0 .08381 | 0 .286979562 |
| SOCS2 | -0 . 198152332 | 8 . 164915107 | -1 .7708 | 0 .08382 | 0 .286979562 |
| TRIM5 | 0 .345410897 | 5 . 1 18657578 | 1 .77005 | 0 .08394 | 0 .287309566 |
| ITGA5 | 0 . 190038262 | 8 .715672195 | 1 .7699 | 0 .08397 | 0 .287318557 |
| GP5 | 0 .23682003 | 5 .422175207 | 1 .76941 | 0 .08405 | 0 .287482749 |
| VDAC2 | -0 . 194619151 | 10 .25369906 | -1 .7693 | 0 .08406 | 0 .287482749 |
| FOXN2 | 0 .356376182 | 7 .347339811 | 1 .76883 | 0 .08415 | 0 .287660354 |
| ACBD3 | 0 .249576267 | 8 .586222207 | 1 .76872 | 0 .08416 | 0 .287660354 |
| HIST1H4L | 0 .439867502 | 5 .317395892 | 1 .76863 | 0 .08418 | 0 .287660354 |
| POMT2 | 0 .297884326 | 6 . 133782618 | 1 .76826 | 0 .08424 | 0 .287780467 |
| CDKL5 | 0 .272947041 | 6 .431769693 | 1 .76815 | 0 .08426 | 0 .287780467 |
| MZF1 | -0 .244455737 | 8 .579065621 | -1 .7679 | 0 .08431 | 0 .287867606 |
| FAM13C | -0 .533772539 | 2 .851990724 | -1 .767 | 0 .08446 | 0 .28831344 |
| SNX2 | -0 .271673702 | 8 .40546295 | -1 .7667 | 0 .08451 | 0 .288406754 |
| RPA4 | -0 .448132145 | 4 .436329412 | -1 .7662 | 0 .0846 | 0 .288613291 |
| HIST1H4D | -0 .253068871 | 5 .201008895 | -1 .7656 | 0 .0847 | 0 .288873433 |
| CD1D | 0 .44659274 | 4 .781833182 | 1 .76524 | 0 .08475 | 0 .288994794 |
| GTF2A2 | 0 .270851168 | 8 .39195582 | 1 .76434 | 0 .08491 | 0 .289440293 |
| PHF7 | 0 .272336992 | 7 .229776939 | 1 .76413 | 0 .08494 | 0 .289484572 |
| UTP3 | 0 .301694123 | 7 .404401583 | 1 .76376 | 0 .08501 | 0 .289617147 |
| STAT6 | -0 . 171501346 | 9 .939990561 | -1 .7633 | 0 .08509 | 0 .289763124 |
| RAB33A | 0 .430633329 | 5 .840075849 | 1 .76319 | 0 .0851 | 0 .289763124 |
| F3 | 0 .594899114 | 5 .658121969 | 1 .76311 | 0 .08512 | 0 .289763124 |
| LPO | 0 .34650806 | 4 .480768045 | 1 .76277 | 0 .08518 | 0 .289803792 |
| CDK14 | -0 .363612387 | 7 .232188157 | -1 .7628 | 0 .08518 | 0 .289803792 |
| HOMER2 | 0 .411734755 | 7 .204362114 | 1 .7611 1 | 0 .08546 | 0 .290688387 |
| PTPN20B | -0 .390951593 | 4 .752539589 | -1 .7604 | 0 .08559 | 0 .291028892 |
| CSN2 | 0 .277767462 | 6 . 147334345 | 1 .76013 | 0 .08563 | 0 .291028892 |
| PYHIN1 | -0 .424575298 | 6 .683500968 | -1 .76 | 0 .08565 | 0 .291028892 |
| SNX29P2 | -0 .530417685 | 3 .356369254 | -1 .7599 | 0 .08566 | 0 .291028892 |

| APC | -0 . 196494282 | 8 .512055767 | -1 .7599 | 0 .08568 | 0 .291028892 |
| --- | --- | --- | --- | --- | --- |
| MMP16 | -0 .26039172 | 6 .807275936 | -1 .7585 | 0 .08591 | 0 .291737631 |
| BHLHE40 | 0 .295144058 | 9 .59122862 | 1 .75806 | 0 .08599 | 0 .291922627 |
| GTF2IRD2B | 0 .360773285 | 6 .292130138 | 1 .75737 | 0 .0861 | 0 .292242843 |
| AP1AR | -0 .441859441 | 5 .491229591 | -1 .7569 | 0 .08618 | 0 .292415974 |
| CLU | -0 .734100332 | 9 .712896977 | -1 .7566 | 0 .08623 | 0 .292526833 |
| ZNF611 | -0 .392631319 | 6 . 127153768 | -1 .7563 | 0 .0863 | 0 .292660023 |
| LARS | -0 .433129033 | 8 .220625614 | -1 .7561 | 0 .08632 | 0 .292660023 |
| UBE2V2 | 0 .30712338 | 7 .370473765 | 1 .75556 | 0 .08642 | 0 .292910377 |
| ZMYM1 | -0 .342775145 | 4 .808340645 | -1 .7551 | 0 .0865 | 0 .293074484 |
| AGL | -0 .296619422 | 6 .791949903 | -1 .755 | 0 .08651 | 0 .293074484 |
| HIST3H2A | -0 .382118403 | 8 .610773879 | -1 .7545 | 0 .08659 | 0 .293274539 |
| NOVA2 | 0 .239179817 | 6 .826619799 | 1 .7542 | 0 .08665 | 0 .293322066 |
| POM121C | -0 .377992602 | 8 .680732277 | -1 .7542 | 0 .08665 | 0 .293322066 |
| MPDU1 | 0 .339422121 | 7 .359984224 | 1 .75373 | 0 .08673 | 0 .293436053 |
| DCPS | 0 . 178929867 | 8 . 152747198 | 1 .75367 | 0 .08674 | 0 .293436053 |
| SAR1A | -0 . 195321414 | 8 .937192084 | -1 .7536 | 0 .08676 | 0 .293436053 |
| MON2 | -0 . 176247993 | 7 .803010873 | -1 .7528 | 0 .0869 | 0 .293843238 |
| DNMBP | -0 . 19184668 | 6 .989621268 | -1 .752 | 0 .08703 | 0 .293996106 |
| RP11-680F8 .4 | -0 .529836988 | 4 .58405197 | -1 .752 | 0 .08704 | 0 .293996106 |
| STK4 | 0 .252730074 | 6 . 125057906 | 1 .75193 | 0 .08705 | 0 .293996106 |
| SVEP1 | -0 .274560642 | 8 .001383088 | -1 .7518 | 0 .08706 | 0 .293996106 |
| OLAH | -0 .566590819 | 3 .750451732 | -1 .7518 | 0 .08706 | 0 .293996106 |
| CNKSR2 | 0 .519982417 | 3 .767887415 | 1 .7516 | 0 .0871 | 0 .294012444 |
| COG5 | -0 .207396661 | 8 .792913041 | -1 .7515 | 0 .08712 | 0 .294012444 |
| SNHG17 | 0 .315115247 | 8 .473954553 | 1 .75129 | 0 .08716 | 0 .2940724 |
| PYCARD | 0 .448071133 | 8 .74908046 | 1 .7497 | 0 .08744 | 0 .294866313 |
| WBP11 | 0 . 198135382 | 8 .719141238 | 1 .74967 | 0 .08744 | 0 .294866313 |
| PODXL | -0 .321041643 | 7 .414386632 | -1 .7495 | 0 .08746 | 0 .294866313 |
| EFCAB11 | -0 .233092631 | 7 .566195433 | -1 .749 | 0 .08756 | 0 .295115004 |
| ATXN7L1 | 0 .498464824 | 5 .481545648 | 1 .74854 | 0 .08764 | 0 .295294455 |
| ZNF426 | 0 .357743922 | 5 . 146665896 | 1 .74804 | 0 .08773 | 0 .295463666 |
| HMG20B | 0 .402747872 | 9 .886316713 | 1 .74799 | 0 .08773 | 0 .295463666 |
| HSD3B2 | -0 .359594452 | 5 .557449003 | -1 .7469 | 0 .08793 | 0 .296016954 |
| PTCD3 | -0 .214160901 | 7 .52336997 | -1 .7468 | 0 .08795 | 0 .296016954 |
| C5AR2 | 0 .282237976 | 6 .575021092 | 1 .74627 | 0 .08804 | 0 .29623798 |
| NLRP1 | -0 .275270672 | 7 .39936138 | -1 .7459 | 0 .0881 | 0 .296273797 |
| SPANXB1 | 0 .627945387 | 3 .521870693 | 1 .74588 | 0 .0881 | 0 .296273797 |
| CACNA1F | 0 .591306301 | 5 .238247172 | 1 .74581 | 0 .08812 | 0 .296273797 |
| IL2RA | 0 .294314896 | 6 .97381996 | 1 .74562 | 0 .08815 | 0 .296296136 |
| UCHL1 | -0 .237313913 | 7 .504535684 | -1 .7455 | 0 .08817 | 0 .296296136 |
| GNA12 | 0 . 185758459 | 6 .788926657 | 1 .74478 | 0 .0883 | 0 .296643035 |
| PDZD3 | -0 .312922301 | 5 .624687748 | -1 .7441 | 0 .08842 | 0 .296984939 |
| UQCRC1 | -0 .295301463 | 10 . 12834583 | -1 .7438 | 0 .08847 | 0 .297078955 |
| CALR | 0 .24930687 | 1 1 .36930222 | 1 .74359 | 0 .08851 | 0 .297109192 |
| ANKLE2 | 0 . 193531268 | 7 .677496176 | 1 .74315 | 0 .08859 | 0 .297222895 |
| FGD6 | -0 .371435431 | 4 .93534611 1 | -1 .7431 | 0 .08859 | 0 .297222895 |
| UXT | -0 .298171721 | 9 .090160712 | -1 .7428 | 0 .08864 | 0 .297318535 |
| P2RY10 | 0 .524804272 | 4 .06961109 | 1 .74256 | 0 .08869 | 0 .297401204 |
| AMHR2 | -0 .513313109 | 4 .009352816 | -1 .7421 | 0 .08878 | 0 .297535491 |
| LY6G6C | 0 .59042696 | 8 .947916511 | 1 .74207 | 0 .08878 | 0 .297535491 |
| OAS2 | 0 .361916804 | 7 .871465198 | 1 .74113 | 0 .08894 | 0 .297939807 |
| TRA2A | -0 .327613336 | 8 .200295143 | -1 .7411 | 0 .08895 | 0 .297939807 |
| CIR1 | 0 .245622913 | 6 . 161417042 | 1 .74026 | 0 .0891 | 0 .298235063 |
| GDF3 | 0 .557293248 | 6 .514653571 | 1 .74024 | 0 .0891 | 0 .298235063 |
| MYH7B | -0 .313784656 | 4 .879213233 | -1 .7402 | 0 .0891 | 0 .298235063 |

| RANGRF | 0 .21142075 | 7 .431221676 | 1 .73998 | 0 .08915 | 0 .298298222 |
| --- | --- | --- | --- | --- | --- |
| FAM224A | -0 .530217268 | 4 . 158923658 | -1 .7395 | 0 .08924 | 0 .298453337 |
| EXTL3 | 0 .231063189 | 8 .429213185 | 1 .73942 | 0 .08925 | 0 .298453337 |
| PEX19 | -0 . 186386466 | 8 .421980754 | -1 .7393 | 0 .08927 | 0 .298453337 |
| KIF18A | 0 .286106327 | 4 .871511629 | 1 .73919 | 0 .08929 | 0 .298453337 |
| LRP10 | 0 .235501732 | 10 .00975901 | 1 .73888 | 0 .08934 | 0 .298554471 |
| CASP4 | -0 .266119148 | 8 .247255171 | -1 .7384 | 0 .08942 | 0 .298736621 |
| CLK2 | 0 .356591827 | 9 .060048798 | 1 .73781 | 0 .08953 | 0 .299032521 |
| TUBB | 0 .241276781 | 12 .63462801 | 1 .73708 | 0 .08966 | 0 .299334157 |
| GLI1 | -0 .413166792 | 5 .816570361 | -1 .737 | 0 .08968 | 0 .299334157 |
| FCHSD2 | -0 .345786828 | 5 .705506519 | -1 .7368 | 0 .08971 | 0 .299334157 |
| ACVR1B | -0 .212979466 | 9 .595489156 | -1 .7368 | 0 .08972 | 0 .299334157 |
| ICK | -0 .675077561 | 5 .315055719 | -1 .736 | 0 .08986 | 0 .299719418 |
| PCDHB3 | 0 .246353758 | 6 . 142653185 | 1 .7358 | 0 .08989 | 0 .2997556 |
| MAP2K5 | -0 .30466416 | 8 .518285581 | -1 .7357 | 0 .08992 | 0 .299760139 |
| CDH12 | -0 .310794339 | 4 .029470284 | -1 .7353 | 0 .08999 | 0 .299830675 |
| CYP2C18 | 0 .397712165 | 6 .258697502 | 1 .73527 | 0 .08999 | 0 .299830675 |
| CARF | -0 .333983505 | 4 .011 148974 | -1 .7345 | 0 .09013 | 0 .300220115 |
| ETFB | -0 .275031044 | 8 .717385744 | -1 .7343 | 0 .09015 | 0 .300225202 |
| EXOSC2 | -0 . 188733659 | 7 .06063877 | -1 .7341 | 0 .0902 | 0 .300314509 |
| SLC25A10 | -0 .215519993 | 6 .207855441 | -1 .7336 | 0 .09028 | 0 .300497996 |
| CDK16 | 0 . 180352928 | 9 .537911646 | 1 .73342 | 0 .09032 | 0 .30050735 |
| STIM1 | -0 .217844138 | 6 .759216047 | -1 .7333 | 0 .09033 | 0 .30050735 |
| PREP | 0 .204198532 | 8 .040262312 | 1 .73271 | 0 .09045 | 0 .300718716 |
| RBM42 | 0 .228461216 | 8 .653013232 | 1 .73271 | 0 .09045 | 0 .300718716 |
| PNMA2 | 0 .366690016 | 6 .654150363 | 1 .73251 | 0 .09048 | 0 .300761784 |
| RNF220 | -0 .24681985 | 7 .944453797 | -1 .7323 | 0 .09052 | 0 .300793842 |
| HIRA | 0 .346835467 | 6 .868208901 | 1 .73119 | 0 .09072 | 0 .301300623 |
| TRMT61B | -0 .244318255 | 6 .846123735 | -1 .731 | 0 .09075 | 0 .301300623 |
| MAPKAPK5 | 0 .333204942 | 5 .659074971 | 1 .73102 | 0 .09075 | 0 .301300623 |
| LPIN1 | -0 .227892596 | 8 .916292269 | -1 .7309 | 0 .09076 | 0 .301300623 |
| RGP1 | -0 .23570207 | 7 .383179467 | -1 .7306 | 0 .09082 | 0 .30140334 |
| HBD | -0 .308937635 | 5 .359929069 | -1 .7305 | 0 .09085 | 0 .301416851 |
| PGF | -0 .315396955 | 9 .667933339 | -1 .7296 | 0 .09101 | 0 .301832014 |
| AUH | -0 .251853065 | 6 .701484981 | -1 .7295 | 0 .09102 | 0 .301832014 |
| PRRG4 | -0 .362509899 | 6 .049725778 | -1 .729 | 0 .09112 | 0 .301936637 |
| CYP27B1 | 0 .368851261 | 5 .671457635 | 1 .72889 | 0 .09114 | 0 .301936637 |
| GPC4 | -0 .280836768 | 6 .516699341 | -1 .7288 | 0 .09116 | 0 .301936637 |
| CAND1 | -0 .205948504 | 8 .420249531 | -1 .7288 | 0 .09116 | 0 .301936637 |
| FLJ10038 | -0 .343713768 | 7 .320546685 | -1 .7287 | 0 .09117 | 0 .301936637 |
| FAF2 | 0 . 171080101 | 8 .807697261 | 1 .72778 | 0 .09134 | 0 .302394715 |
| RHBDL1 | 0 .466867492 | 2 .384387804 | 1 .7276 | 0 .09137 | 0 .302423646 |
| USE1 | -0 .23691509 | 7 .838081612 | -1 .7273 | 0 .09143 | 0 .302543313 |
| CTH | -0 .225492495 | 6 .9868168 | -1 .7271 | 0 .09146 | 0 .302556776 |
| ATF7IP | 0 .342796673 | 7 .990291722 | 1 .72698 | 0 .09148 | 0 .302556776 |
| TRAC | 0 .843798005 | 8 .632494633 | 1 .72673 | 0 .09153 | 0 .302570817 |
| CETN2 | 0 .250022546 | 8 .384132967 | 1 .72636 | 0 .09159 | 0 .302570817 |
| KLC1 | -0 .224206041 | 9 .476146828 | -1 .7263 | 0 .09161 | 0 .302570817 |
| LSM14B | 0 .267362127 | 7 . 152323149 | 1 .72623 | 0 .09162 | 0 .302570817 |
| HCG18 | -0 .430187427 | 5 .261080708 | -1 .7258 | 0 .0917 | 0 .302570817 |
| RBBP4 | -0 .236608376 | 9 .070988082 | -1 .7257 | 0 .09171 | 0 .302570817 |
| CDT1 | 0 . 189993324 | 6 .004986115 | 1 .7257 | 0 .09171 | 0 .302570817 |
| OR1D2 | -0 .289170461 | 7 .433382641 | -1 .7257 | 0 .09172 | 0 .302570817 |
| LINC01140 | -0 .273448665 | 6 .383435862 | -1 .7254 | 0 .09177 | 0 .302570817 |
| GDPD5 | 0 .251828673 | 8 .753240339 | 1 .72536 | 0 .09178 | 0 .302570817 |
| GAK | 0 . 178665624 | 9 .411785222 | 1 .72532 | 0 .09178 | 0 .302570817 |

| PTBP3 | 0 . 165953203 | 7 .74922239 | 1 .72521 | 0 .0918 | 0 .302570817 |
| --- | --- | --- | --- | --- | --- |
| WDR12 | 0 . 192202072 | 7 .97826796 | 1 .72513 | 0 .09182 | 0 .302570817 |
| PPP1R7 | 0 . 176866421 | 9 .46586693 | 1 .72499 | 0 .09184 | 0 .302570817 |
| FOLH1B | 0 .309760011 | 5 .414996171 | 1 .72485 | 0 .09187 | 0 .302570817 |
| RAB27A | 0 .311657664 | 10 .28020693 | 1 .72484 | 0 .09187 | 0 .302570817 |
| LMAN1L | 0 .513072933 | 4 .08321536 | 1 .72374 | 0 .09207 | 0 .303138686 |
| PIDD1 | 0 .296023166 | 8 .487682445 | 1 .72363 | 0 .09209 | 0 .303138686 |
| PMVK | -0 .367202678 | 8 .462836973 | -1 .7233 | 0 .09214 | 0 .303190983 |
| GABRB2 | -0 .379617307 | 5 .601658683 | -1 .7231 | 0 .09218 | 0 .303190983 |
| ZNF682 | -0 .434679934 | 3 .52725638 | -1 .7231 | 0 .09218 | 0 .303190983 |
| MXI1 | -0 .386259051 | 9 .349781838 | -1 .7229 | 0 .09222 | 0 .303237251 |
| CNGA1 | -0 .360650999 | 5 .347901482 | -1 .7227 | 0 .09226 | 0 .303260268 |
| TBC1D15 | -0 .217865776 | 7 .259594508 | -1 .7226 | 0 .09227 | 0 .303260268 |
| VGF | 0 .731333051 | 5 .801744082 | 1 .72207 | 0 .09238 | 0 .303449822 |
| CINP | 0 .232045827 | 7 .548619735 | 1 .72193 | 0 .0924 | 0 .303449822 |
| TRIM27 | 0 . 196898201 | 9 .207934501 | 1 .72192 | 0 .0924 | 0 .303449822 |
| RABGGTB | -0 .34028941 | 6 .578547961 | -1 .7217 | 0 .09245 | 0 .303528737 |
| PMPCA | 0 .223970048 | 7 .907061859 | 1 .72024 | 0 .09271 | 0 .304301999 |
| SEMA3E | 0 .534251358 | 4 .235569397 | 1 .71968 | 0 .09281 | 0 .304559244 |
| GOLGA2P5 | 0 .333683352 | 4 .273165961 | 1 .71931 | 0 .09288 | 0 .304701531 |
| CLEC1B | -0 .443494754 | 5 .283810533 | -1 .7189 | 0 .09296 | 0 .304760361 |
| DPYD | -0 .392946691 | 5 .848361174 | -1 .7186 | 0 .09301 | 0 .304760361 |
| LTBP3 | -0 .329660627 | 7 .581635432 | -1 .7186 | 0 .09301 | 0 .304760361 |
| RAD54L | 0 .364981225 | 4 .837973527 | 1 .71856 | 0 .09302 | 0 .304760361 |
| MRPS30 | -0 .232808218 | 7 .996082951 | -1 .7186 | 0 .09302 | 0 .304760361 |
| MMP8 | 0 .416147713 | 5 .429813797 | 1 .71813 | 0 .0931 | 0 .304936447 |
| CHMP1B | 0 . 167694179 | 8 .977488647 | 1 .71758 | 0 .0932 | 0 .30515022 |
| CHORDC1 | -0 .625031772 | 7 .484585314 | -1 .7175 | 0 .09321 | 0 .30515022 |
| RNF146 | -0 . 154569197 | 6 .867406629 | -1 .7173 | 0 .09326 | 0 .30515022 |
| PTPN18 | 0 . 197984694 | 9 .216504964 | 1 .71712 | 0 .09329 | 0 .30515022 |
| BEAN1 | 0 .287181513 | 6 .724632163 | 1 .71701 | 0 .09331 | 0 .30515022 |
| HMMR | 0 . 194255156 | 8 .003745001 | 1 .71698 | 0 .09331 | 0 .30515022 |
| FTSJ3 | 0 .23998423 | 8 .029952127 | 1 .71642 | 0 .09341 | 0 .305323273 |
| CSNK1D | 0 . 169169391 | 9 .69735246 | 1 .7164 | 0 .09342 | 0 .305323273 |
| GTF2H2B | -0 .458146013 | 5 .267615392 | -1 .7163 | 0 .09344 | 0 .305323273 |
| USP2 | 0 .24422508 | 7 .487161234 | 1 .7158 | 0 .09353 | 0 .305542025 |
| UPF2 | -0 .235483437 | 7 .354642737 | -1 .7156 | 0 .09357 | 0 .305592156 |
| YOD1 | 0 .478295315 | 4 .043048966 | 1 .7154 | 0 .0936 | 0 .305625402 |
| CD24 | 0 .727517859 | 10 .76261857 | 1 .71494 | 0 .09369 | 0 .305745513 |
| DHCR24 | -0 .603494536 | 8 .87246763 | -1 .7149 | 0 .09369 | 0 .305745513 |
| KCND3 | -0 .211594323 | 7 .778391753 | -1 .7145 | 0 .09377 | 0 .305922326 |
| TTLL1 | 0 .242576724 | 6 .643264158 | 1 .71438 | 0 .09379 | 0 .305922326 |
| DDX28 | 0 . 166680069 | 7 .933707224 | 1 .71424 | 0 .09382 | 0 .30593004 |
| C4orf6 | 0 .274478868 | 4 .744380264 | 1 .71278 | 0 .09409 | 0 .30668914 |
| IL24 | 0 .384138057 | 4 .596704321 | 1 .71272 | 0 .0941 | 0 .30668914 |
| PTPN12 | -0 .311 178548 | 8 .251369928 | -1 .7125 | 0 .09414 | 0 .306749291 |
| HOXC4 | 0 .227832796 | 7 .763852423 | 1 .71222 | 0 .09419 | 0 .306835919 |
| NDUFA3 | 0 .306267079 | 9 .201621353 | 1 .71176 | 0 .09428 | 0 .306981051 |
| CIAPIN1 | 0 . 182899227 | 9 .03012128 | 1 .71168 | 0 .09429 | 0 .306981051 |
| FABP6 | -0 .638085727 | 3 .306875907 | -1 .7115 | 0 .09432 | 0 .306981051 |
| ADAMTSL2 | -0 .631255756 | 2 .719755644 | -1 .7114 | 0 .09434 | 0 .306981051 |
| ATP8B1 | -0 .431921295 | 6 .530874844 | -1 .7112 | 0 .09438 | 0 .306981051 |
| DLL3 | 0 .331941484 | 5 .964113878 | 1 .711 19 | 0 .09438 | 0 .306981051 |
| GTF2E2 | 0 .268129842 | 6 .867348203 | 1 .71095 | 0 .09443 | 0 .307000856 |
| FXR1 | -0 .319523844 | 9 .341488665 | -1 .7108 | 0 .09446 | 0 .307000856 |
| IDI1 | -0 .398902692 | 8 .789659498 | -1 .7108 | 0 .09446 | 0 .307000856 |

| NPHS2 | 0 .804412731 | 3 .535695891 | 1 .71026 | 0 .09456 | 0 .307218782 |
| --- | --- | --- | --- | --- | --- |
| INPP5B | 0 . 193991957 | 6 . 126023659 | 1 .71014 | 0 .09458 | 0 .307218782 |
| NUFIP1 | 0 .237512821 | 7 .981501609 | 1 .70999 | 0 .09461 | 0 .307222417 |
| CEP112 | 0 .248510513 | 6 .264835435 | 1 .70987 | 0 .09463 | 0 .307222417 |
| DNASE1 | -0 .555593199 | 2 .692492114 | -1 .7088 | 0 .09483 | 0 .307793074 |
| PFN2 | -0 .467160354 | 8 .300033393 | -1 .7085 | 0 .09488 | 0 .307876822 |
| WDR3 | 0 .282089217 | 6 .61686753 | 1 .70807 | 0 .09497 | 0 .308077049 |
| FAM172A | -0 .3131374 | 7 . 176054523 | -1 .7079 | 0 .095 | 0 .30809523 |
| SLC30A5 | 0 .315465379 | 7 .542638575 | 1 .70697 | 0 .09517 | 0 .308584519 |
| PENK | -0 .323063014 | 5 .3511 15581 | -1 .7066 | 0 .09525 | 0 .308744869 |
| PDX1 | 0 .320319325 | 5 .545401973 | 1 .70615 | 0 .09533 | 0 .308925924 |
| ATAD2 | 0 .319395589 | 5 .655662756 | 1 .70558 | 0 .09543 | 0 .309193966 |
| FIP1L1 | 0 .384033753 | 6 .254602525 | 1 .70512 | 0 .09552 | 0 .309392617 |
| YWHAE | -0 .225233596 | 10 .67119447 | -1 .7049 | 0 .09555 | 0 .309424873 |
| FAP | 0 .350265045 | 6 .088000797 | 1 .70447 | 0 .09564 | 0 .309627666 |
| SENP6 | -0 .251361347 | 7 .3497073 | -1 .7043 | 0 .09568 | 0 .309681647 |
| PPME1 | 0 .220962618 | 9 .359745986 | 1 .70409 | 0 .09571 | 0 .309698408 |
| CD38 | 0 .327356727 | 6 .562421275 | 1 .70388 | 0 .09575 | 0 .309700754 |
| MEIS3P1 | -0 .374615348 | 7 .85047538 | -1 .7037 | 0 .09578 | 0 .309700754 |
| PDAP1 | 0 .224138147 | 7 .898928818 | 1 .70358 | 0 .09581 | 0 .309700754 |
| CMC2 | 0 .319150024 | 7 .629798205 | 1 .70357 | 0 .09581 | 0 .309700754 |
| C10orf95 | -0 .308070476 | 2 .699825631 | -1 .7033 | 0 .09586 | 0 .309706954 |
| HSPA1L | 0 .247116262 | 5 .023915308 | 1 .70329 | 0 .09586 | 0 .309706954 |
| PAEP | 0 .447523002 | 4 .903121276 | 1 .70297 | 0 .09593 | 0 .309824413 |
| ZNF835 | 0 .561983486 | 2 .543303669 | 1 .70243 | 0 .09603 | 0 .310074805 |
| S100A3 | 0 .333486214 | 6 .083736569 | 1 .70225 | 0 .09606 | 0 .310101337 |
| KLK10 | 0 .675824389 | 8 .451535483 | 1 .70197 | 0 .09611 | 0 .310188022 |
| PTPN6 | 0 .423579848 | 7 .487382021 | 1 .70185 | 0 .09614 | 0 .310188022 |
| KITLG | 0 .30193812 | 5 .560392771 | 1 .70143 | 0 .09622 | 0 .310362904 |
| ELF3 | -0 .24923385 | 7 . 198668258 | -1 .7013 | 0 .09624 | 0 .310362904 |
| MTHFD2 | 0 .369895626 | 7 . 121179702 | 1 .7008 | 0 .09634 | 0 .310590009 |
| RHOH | 0 .280485194 | 6 .856448655 | 1 .70015 | 0 .09646 | 0 .310795535 |
| KCNK3 | 0 .301248589 | 5 .055112557 | 1 .70005 | 0 .09648 | 0 .310795535 |
| CCNT2 | -0 . 192329883 | 8 .070144035 | -1 .6999 | 0 .0965 | 0 .310795535 |
| ERCC1 | 0 .249491034 | 9 .272838518 | 1 .69978 | 0 .09653 | 0 .310795535 |
| SLC8A1 | 0 .326649119 | 5 .053001785 | 1 .69973 | 0 .09654 | 0 .310795535 |
| TGFB1 | 0 .410641827 | 8 .545306478 | 1 .69968 | 0 .09655 | 0 .310795535 |
| PEX2 | -0 .214116761 | 7 .562325593 | -1 .6994 | 0 .09661 | 0 .310904039 |
| ZNF696 | 0 .390367951 | 4 .001940928 | 1 .69924 | 0 .09663 | 0 .310904039 |
| TCOF1 | -0 .352369597 | 8 .274162507 | -1 .6989 | 0 .09669 | 0 .310997885 |
| ICE2 | -0 .289505236 | 6 .823436924 | -1 .6988 | 0 .09671 | 0 .310997885 |
| ITGAV | -0 .422281766 | 8 .457354558 | -1 .6983 | 0 .09681 | 0 .311248051 |
| PRB4 | -0 .22254572 | 3 .845284234 | -1 .6979 | 0 .09689 | 0 .311403249 |
| R3HDM4 | 0 . 196043931 | 10 .54377943 | 1 .69661 | 0 .09713 | 0 .312064765 |
| MX1 | 0 .628446838 | 9 .281881716 | 1 .69656 | 0 .09714 | 0 .312064765 |
| CILP | -0 .343805047 | 6 .715970783 | -1 .6959 | 0 .09727 | 0 .312409785 |
| RP4-710M16 . 1 | -0 .2714411 1 1 | 7 .842832063 | -1 .6953 | 0 .09739 | 0 .312707101 |
| MCF2L2 | 0 .443910123 | 2 .885893737 | 1 .69474 | 0 .09749 | 0 .312943162 |
| FBXO9 | -0 .233381148 | 9 .720620117 | -1 .6945 | 0 .09754 | 0 .313014736 |
| BHLHE41 | 0 .414980314 | 7 .384851147 | 1 .69407 | 0 .09762 | 0 .313185513 |
| R3HCC1L | -0 .253245766 | 5 .869292528 | -1 .694 | 0 .09764 | 0 .313185513 |
| CORO2B | -0 .734413497 | 8 .504292825 | -1 .6931 | 0 .09781 | 0 .313655496 |
| GDPD3 | 0 .310838204 | 6 .948264833 | 1 .691 | 0 .09821 | 0 .314840156 |
| ADH7 | 0 .26751928 | 4 .781416614 | 1 .69077 | 0 .09825 | 0 .314907396 |
| MTHFD2L | -0 .278859378 | 5 .200481385 | -1 .69 | 0 .0984 | 0 .315225917 |
| PTPN13 | -0 .342327201 | 5 .32184772 | -1 .6899 | 0 .09842 | 0 .315225917 |

| TRDMT1 | -0 .295068267 | 6 .036336101 | -1 .6898 | 0 .09844 | 0 .315225917 |
| --- | --- | --- | --- | --- | --- |
| WDR77 | 0 .242650304 | 8 .968467006 | 1 .68962 | 0 .09847 | 0 .315225917 |
| TUG1 | -0 .241700174 | 9 .881357357 | -1 .6896 | 0 .09848 | 0 .315225917 |
| GPN2 | 0 .203508508 | 6 .635652817 | 1 .68947 | 0 .0985 | 0 .315225917 |
| EN2 | 0 .467281823 | 6 .758673934 | 1 .68934 | 0 .09853 | 0 .315226617 |
| IGK | 1 .388454439 | 4 .553579645 | 1 .68909 | 0 .09858 | 0 .315294243 |
| FBXO40 | 0 .325868583 | 5 .071793041 | 1 .68897 | 0 .0986 | 0 .315294243 |
| CCNT1 | -0 .224432984 | 7 .20851452 | -1 .6888 | 0 .09862 | 0 .315297837 |
| AREG | 0 .359942988 | 6 .032900964 | 1 .68843 | 0 .0987 | 0 .315462378 |
| FCER1A | -0 .390403587 | 7 .400793392 | -1 .6875 | 0 .09887 | 0 .315856926 |
| ADRA2A | -0 .316411212 | 6 .905628393 | -1 .6875 | 0 .09888 | 0 .315856926 |
| CTAG2 | 0 .770508215 | 5 .69809614 | 1 .6873 | 0 .09892 | 0 .31588281 |
| LY86 | 0 .268822765 | 7 .684219147 | 1 .68723 | 0 .09893 | 0 .31588281 |
| ZDHHC8P1 | -0 .306852814 | 4 .954656808 | -1 .6869 | 0 .09899 | 0 .315969615 |
| PPBPP2 | 0 .408581312 | 4 .249084816 | 1 .68683 | 0 .09901 | 0 .315969615 |
| CAPZA2 | -0 .378082028 | 8 .330975688 | -1 .6858 | 0 .09921 | 0 .31652007 |
| RAMP2 | -0 .33054513 | 6 .571415943 | -1 .6848 | 0 .0994 | 0 .31704779 |
| ZNF394 | 0 .220518979 | 7 .003570528 | 1 .68449 | 0 .09947 | 0 .317178067 |
| RHCG | -0 .297070231 | 6 .956526928 | -1 .6843 | 0 .0995 | 0 .317194788 |
| GZMK | 0 .65418138 | 7 .356325507 | 1 .68383 | 0 .09959 | 0 .317426467 |
| SLC7A7 | 0 .271612108 | 7 .075889648 | 1 .68342 | 0 .09968 | 0 .317603393 |
| ALCAM | -0 .410417868 | 8 .694485851 | -1 .6833 | 0 .0997 | 0 .317603794 |
| TNFSF8 | 0 .243049617 | 5 .991974193 | 1 .68299 | 0 .09976 | 0 .317708084 |
| ST8SIA1 | 0 .513835954 | 5 .223636301 | 1 .68272 | 0 .09981 | 0 .317796639 |
| SLC10A1 | -0 .557721425 | 5 . 155441702 | -1 .6823 | 0 .0999 | 0 .317995184 |
| WNT7A | -0 .288555674 | 5 .258794347 | -1 .682 | 0 .09994 | 0 .318058252 |
| EPS15 | -0 .352855657 | 9 . 101888446 | -1 .6814 | 0 . 10006 | 0 .318351437 |
| ATP6V0A4 | -0 .243103773 | 6 .507800419 | -1 .6811 | 0 . 10013 | 0 .318426652 |
| 8-Mar | -0 .230397394 | 6 .509664561 | -1 .6811 | 0 . 10014 | 0 .318426652 |
| ASB8 | -0 . 194649261 | 7 .28411489 | -1 .6806 | 0 . 10022 | 0 .318582899 |
| NEU1 | 0 .291341286 | 7 .735491475 | 1 .68054 | 0 . 10024 | 0 .318582899 |
| PRR11 | -0 .534709684 | 6 .989987273 | -1 .6802 | 0 . 10029 | 0 .318636707 |
| ZBTB7A | -0 .215330478 | 8 .028427706 | -1 .6802 | 0 . 10031 | 0 .318636707 |
| SF3B1 | -0 .212364783 | 1 1 .22149351 | -1 .6799 | 0 . 10037 | 0 .318636707 |
| TP63 | -0 .331581846 | 9 .954993028 | -1 .6798 | 0 . 10037 | 0 .318636707 |
| PMS2P4 | 0 .520110833 | 3 .625596741 | 1 .67981 | 0 . 10038 | 0 .318636707 |
| IGLL3P | 1 .464160295 | 8 .711859176 | 1 .67965 | 0 . 10041 | 0 .318654814 |
| SAC3D1 | 0 .363353813 | 6 .401544911 | 1 .67916 | 0 . 10051 | 0 .318877885 |
| MST1R | -0 .40496699 | 6 .41892659 | -1 .6789 | 0 . 10056 | 0 .318968833 |
| LOC100507630 | 0 .52324684 | 3 .959683735 | 1 .67821 | 0 . 10069 | 0 .319253999 |
| SQRDL | 0 .429930337 | 8 .391398026 | 1 .67811 | 0 . 10071 | 0 .319253999 |
| ARHGAP32 | -0 .226332326 | 8 . 100943848 | -1 .678 | 0 . 10073 | 0 .319253999 |
| B3GALT2 | -0 .366331365 | 4 .87542557 | -1 .6779 | 0 . 10076 | 0 .319279779 |
| SNX19 | -0 .352581472 | 6 .960750369 | -1 .6776 | 0 . 10082 | 0 .319396803 |
| SETD3 | -0 . 150483335 | 8 .311009473 | -1 .6773 | 0 . 10088 | 0 .319485409 |
| TNFSF15 | -0 .524947452 | 4 .240390117 | -1 .677 | 0 . 10094 | 0 .319527559 |
| ABCC5 | -0 .215023819 | 7 .515391071 | -1 .677 | 0 . 10094 | 0 .319527559 |
| DDX27 | 0 .211219736 | 8 .974203448 | 1 .67614 | 0 . 1011 | 0 .319951106 |
| ERAL1 | 0 . 174922293 | 7 .733125229 | 1 .67584 | 0 . 10116 | 0 .319987137 |
| SULT2B1 | 0 .399965284 | 8 .869133223 | 1 .67583 | 0 . 10116 | 0 .319987137 |
| YAP1 | -0 .338507322 | 5 .844710944 | -1 .6751 | 0 . 10131 | 0 .320301192 |
| TUBB2B | 0 .846127807 | 6 .085248674 | 1 .67507 | 0 . 10131 | 0 .320301192 |
| USP20 | -0 . 186276925 | 6 .745738505 | -1 .6748 | 0 . 10137 | 0 .320404723 |
| ZNF423 | -0 .308569209 | 6 .025829837 | -1 .6746 | 0 . 10141 | 0 .320416908 |
| TFPI2 | 0 .441821482 | 5 . 106648245 | 1 .67449 | 0 . 10143 | 0 .320416908 |
| NUDT18 | 0 .306750477 | 5 .289204897 | 1 .67419 | 0 . 10149 | 0 .320527091 |

| HMGCR | -0 .501559301 | 7 .325530619 | -1 .6733 | 0 . 10166 | 0 .320894487 |
| --- | --- | --- | --- | --- | --- |
| IRS2 | -0 .455258695 | 10 . 16661672 | -1 .6732 | 0 . 10167 | 0 .320894487 |
| CYSLTR2 | 0 .376304909 | 5 .432656385 | 1 .67306 | 0 . 10171 | 0 .320894487 |
| SHOX2 | -0 .307212174 | 6 .345690346 | -1 .6729 | 0 . 10173 | 0 .320894487 |
| DNAJA1 | -0 .402090495 | 9 .929734081 | -1 .6728 | 0 . 10176 | 0 .320894487 |
| ACAP2 | -0 .338217859 | 7 .493547605 | -1 .6727 | 0 . 10177 | 0 .320894487 |
| KLK14 | -0 .338499513 | 4 .922303366 | -1 .6727 | 0 . 10178 | 0 .320894487 |
| GABRD | -0 .301412896 | 4 .574257244 | -1 .6725 | 0 . 10183 | 0 .320952226 |
| TFAP2C | -0 .400622016 | 8 .650107974 | -1 .6719 | 0 . 10194 | 0 .321242561 |
| SFT2D2 | 0 . 189674187 | 6 .644314751 | 1 .67161 | 0 . 102 | 0 .32133037 |
| RUVBL2 | 0 .240554442 | 8 .47416811 | 1 .67133 | 0 . 10205 | 0 .321423022 |
| OGN | -0 .555630632 | 2 .476017967 | -1 .6711 | 0 . 10211 | 0 .321518671 |
| LAMP1 | 0 . 179154071 | 12 . 16281931 | 1 .67069 | 0 . 10218 | 0 .32166543 |
| RPS6KC1 | 0 .247825095 | 6 .441485766 | 1 .6703 | 0 . 10226 | 0 .321831153 |
| ROS1 | 0 .249551377 | 6 .261994406 | 1 .6701 | 0 . 1023 | 0 .3218511 13 |
| GPR37L1 | 0 .574386693 | 3 .991621369 | 1 .66997 | 0 . 10232 | 0 .3218511 13 |
| BLCAP | -0 . 194751332 | 9 .26811214 | -1 .6698 | 0 . 10235 | 0 .3218511 13 |
| SERPINA3 | 0 .62645344 | 6 .637235735 | 1 .6696 | 0 . 1024 | 0 .3218511 13 |
| ROR1 | -0 .22920016 | 7 .520335142 | -1 .6694 | 0 . 10243 | 0 .3218511 13 |
| NAV3 | -0 .354720437 | 6 .404191818 | -1 .6693 | 0 . 10245 | 0 .3218511 13 |
| PPP1R12B | -0 .314088205 | 7 .531454844 | -1 .6693 | 0 . 10246 | 0 .3218511 13 |
| ADCY6 | -0 .264927887 | 6 .999258765 | -1 .6692 | 0 . 10247 | 0 .3218511 13 |
| ALG8 | 0 .281011842 | 8 . 189899203 | 1 .66883 | 0 . 10255 | 0 .322026185 |
| RABGAP1L | -0 . 17282699 | 8 .833743879 | -1 .6682 | 0 . 10267 | 0 .322306279 |
| MGA | -0 .434372256 | 5 .912916148 | -1 .6681 | 0 . 1027 | 0 .322321676 |
| RAB9A | 0 .284751161 | 8 .264526646 | 1 .66795 | 0 . 10273 | 0 .32233514 |
| VEGFA | 0 .2423047 | 9 .306346839 | 1 .66769 | 0 . 10278 | 0 .322413536 |
| NELFCD | 0 .228116685 | 8 .463595695 | 1 .6668 | 0 . 10296 | 0 .322892626 |
| INPP5J | 0 .559098291 | 4 .073013061 | 1 .66663 | 0 . 10299 | 0 .322917154 |
| CCDC181 | -0 .329445725 | 4 .265754667 | -1 .6661 | 0 . 10309 | 0 .32304978 |
| CELF2 | -0 .309966212 | 10 .67733731 | -1 .6661 | 0 . 1031 | 0 .32304978 |
| CRYL1 | -0 .375753669 | 8 .008190532 | -1 .666 | 0 . 10312 | 0 .32304978 |
| HSPA6 | 0 .256068313 | 7 .892357223 | 1 .66591 | 0 . 10313 | 0 .32304978 |
| ENPP4 | -0 .325067082 | 5 .823199847 | -1 .6656 | 0 . 10319 | 0 .323150087 |
| PLEKHA8P1 | 0 .350464134 | 5 . 126869336 | 1 .66498 | 0 . 10332 | 0 .323467996 |
| STXBP6 | -0 .42807399 | 7 .513388188 | -1 .664 | 0 . 10352 | 0 .32393822 |
| VAMP4 | -0 .242609799 | 7 .894047372 | -1 .664 | 0 . 10352 | 0 .32393822 |
| PIK3R3 | 0 .304602332 | 7 .225556375 | 1 .6634 | 0 . 10364 | 0 .324211336 |
| FBRS | 0 .242092137 | 6 .662580976 | 1 .66329 | 0 . 10366 | 0 .324211336 |
| BCOR | 0 .257960093 | 5 .907782629 | 1 .6627 | 0 . 10378 | 0 .324498002 |
| IQCH | -0 .434790202 | 3 .870728556 | -1 .6624 | 0 . 10384 | 0 .324618824 |
| KIAA0922 | 0 .201486072 | 6 .489595481 | 1 .66183 | 0 . 10396 | 0 .324889119 |
| LOC145678 | -0 .416567388 | 4 .523268364 | -1 .6614 | 0 . 10404 | 0 .325072287 |
| SPATS2L | 0 . 142669348 | 9 .852430113 | 1 .66105 | 0 . 10411 | 0 .325216637 |
| CKB | -0 .446607497 | 8 . 183841258 | -1 .6607 | 0 . 10419 | 0 .32536527 |
| ABCA4 | 0 .459143159 | 4 .414029585 | 1 .66047 | 0 . 10423 | 0 .325419027 |
| PDGFRA | -0 .252590739 | 8 .734245674 | -1 .6601 | 0 . 1043 | 0 .325547016 |
| ST6GALNAC4 | -0 .23200097 | 7 .853963337 | -1 .66 | 0 . 10432 | 0 .325547016 |
| COL15A1 | 0 .381409744 | 9 . 191592883 | 1 .65936 | 0 . 10445 | 0 .325877643 |
| SLC22A18 | 0 .29639206 | 7 .631647998 | 1 .65896 | 0 . 10453 | 0 .325999554 |
| VILL | -0 .354540346 | 6 . 150953135 | -1 .6589 | 0 . 10454 | 0 .325999554 |
| INS | 0 .458497231 | 3 .919745169 | 1 .65807 | 0 . 10472 | 0 .326400495 |
| IL10RA | 0 .469076799 | 7 .356492062 | 1 .65789 | 0 . 10475 | 0 .326400495 |
| GAS2 | 0 .614642901 | 3 .685481728 | 1 .65783 | 0 . 10476 | 0 .326400495 |
| F2RL3 | 0 .462776246 | 5 .019317204 | 1 .65777 | 0 . 10478 | 0 .326400495 |
| AKTIP | -0 .231046033 | 6 .811 1 10287 | -1 .6575 | 0 . 10483 | 0 .326481975 |

| IFNA17 | -0 .351889715 | 5 .757737567 | -1 .6574 | 0 . 10486 | 0 .32649102 |
| --- | --- | --- | --- | --- | --- |
| CD58 | 0 .380039223 | 8 .727509174 | 1 .65672 | 0 . 10499 | 0 .326820662 |
| DUS1L | 0 .261271536 | 8 .274295754 | 1 .65653 | 0 . 10503 | 0 .326858831 |
| ATG4B | 0 .281062909 | 8 .748878183 | 1 .65637 | 0 . 10506 | 0 .326880045 |
| ATM | -0 .23520527 | 8 .307518811 | -1 .656 | 0 . 10514 | 0 .327034392 |
| DOCK9 | -0 . 193964472 | 8 .205244877 | -1 .6557 | 0 . 10519 | 0 .327052635 |
| TLR5 | -0 .211983152 | 6 .25250874 | -1 .6557 | 0 . 1052 | 0 .327052635 |
| C14orf93 | -0 .493483008 | 5 .425095398 | -1 .6556 | 0 . 10522 | 0 .327052635 |
| CSAD | -0 .38311563 | 5 .890577944 | -1 .6554 | 0 . 10526 | 0 .327082157 |
| ADAMTS8 | -0 .290624699 | 6 .408260429 | -1 .6552 | 0 . 1053 | 0 .327129126 |
| TBX2 | 0 .32187286 | 9 . 1 16824155 | 1 .65474 | 0 . 10539 | 0 .327343797 |
| ZNF74 | -0 .271635466 | 5 .020227972 | -1 .6544 | 0 . 10547 | 0 .327509282 |
| RAB13 | 0 .234064505 | 10 .0487822 | 1 .65392 | 0 . 10556 | 0 .327642492 |
| PYGL | -0 .46616047 | 8 .343853796 | -1 .6539 | 0 . 10557 | 0 .327642492 |
| FAM135A | 0 .476826664 | 2 .707781834 | 1 .65362 | 0 . 10562 | 0 .32769808 |
| GPR18 | 0 .432980107 | 4 .866374901 | 1 .65354 | 0 . 10564 | 0 .32769808 |
| UBXN1 | -0 .224870968 | 9 .496123235 | -1 .6533 | 0 . 10568 | 0 .327764015 |
| MORN1 | 0 .498154276 | 3 .708004438 | 1 .6521 | 0 . 10593 | 0 .32819789 |
| LAIR2 | 0 .335003989 | 5 .316657917 | 1 .6521 | 0 . 10593 | 0 .32819789 |
| LYL1 | 0 .33741952 | 5 .576874773 | 1 .65208 | 0 . 10594 | 0 .32819789 |
| ATP11B | -0 .340081914 | 6 . 1 14551376 | -1 .652 | 0 . 10596 | 0 .32819789 |
| ABAT | 0 .200890953 | 7 . 168971322 | 1 .65177 | 0 . 106 | 0 .32819789 |
| GAL | -0 .816352062 | 7 .605708602 | -1 .6518 | 0 . 106 | 0 .32819789 |
| SETD8 | 0 . 185156144 | 5 .988686782 | 1 .65173 | 0 . 10601 | 0 .32819789 |
| PHF2 | -0 . 18814273 | 8 .096517389 | -1 .6505 | 0 . 10625 | 0 .328814266 |
| FRS3 | 0 .216894914 | 6 .281338714 | 1 .65051 | 0 . 10626 | 0 .328814266 |
| ITGA3 | 0 .430236157 | 8 .514127753 | 1 .6503 | 0 . 1063 | 0 .32884479 |
| PWAR5 | -0 .498918804 | 4 .545807011 | -1 .6502 | 0 . 10632 | 0 .32884479 |
| RPL36AL | -0 .222893931 | 10 .4207811 1 | -1 .6496 | 0 . 10646 | 0 .329176738 |
| GRIN2B | -0 .289547009 | 6 . 146102864 | -1 .6491 | 0 . 10655 | 0 .329386909 |
| LY96 | 0 .60099673 | 5 .651661824 | 1 .64897 | 0 . 10658 | 0 .329386909 |
| SREK1 | -0 .219412524 | 7 .616216297 | -1 .6481 | 0 . 10676 | 0 .329872007 |
| IGFLR1 | 0 .264316373 | 7 .334391457 | 1 .64769 | 0 . 10684 | 0 .330035544 |
| NPAP1 | 0 .236419481 | 6 .453071129 | 1 .64719 | 0 . 10694 | 0 .330246769 |
| MTO1 | -0 . 188708281 | 7 .453464299 | -1 .6471 | 0 . 10696 | 0 .330246769 |
| TAAR5 | 0 .356583988 | 5 .052368245 | 1 .64605 | 0 . 10718 | 0 .330840673 |
| FBXL6 | 0 .215891639 | 6 .841239147 | 1 .64578 | 0 . 10723 | 0 .330930058 |
| AAK1 | 0 .210553412 | 8 .994425137 | 1 .64516 | 0 . 10736 | 0 .331243993 |
| CLDN15 | 0 .364222775 | 6 .630212597 | 1 .64503 | 0 . 10739 | 0 .331246222 |
| EVPL | -0 .587022007 | 7 .775269004 | -1 .6448 | 0 . 10743 | 0 .331285602 |
| PLVAP | 0 .289886818 | 8 .699256718 | 1 .64454 | 0 . 10749 | 0 .331397932 |
| DCT | -0 .767105325 | 12 .34468426 | -1 .644 | 0 . 10761 | 0 .331694168 |
| PSORS1C2 | 0 .764037351 | 6 .49825642 | 1 .64289 | 0 . 10783 | 0 .332166361 |
| TOP2B | -0 .303555549 | 7 .959422449 | -1 .6429 | 0 . 10784 | 0 .332166361 |
| MTERF3 | 0 .357172813 | 6 .285826528 | 1 .64283 | 0 . 10785 | 0 .332166361 |
| SLAMF1 | 0 .296307707 | 6 .098794479 | 1 .64254 | 0 . 10791 | 0 .332270095 |
| MAP2K2 | 0 .21660756 | 9 .787648775 | 1 .64229 | 0 . 10796 | 0 .332348557 |
| FGF6 | 0 .520398001 | 5 .396364626 | 1 .64199 | 0 . 10802 | 0 .332398292 |
| ATXN7 | -0 .245457527 | 7 .229744358 | -1 .642 | 0 . 10803 | 0 .332398292 |
| ARL6IP1 | -0 .27772955 | 9 .253798276 | -1 .6418 | 0 . 10807 | 0 .33243583 |
| HSPB3 | -0 .225512122 | 5 .527017738 | -1 .6412 | 0 . 1082 | 0 .332753302 |
| CDH8 | 0 .294619408 | 5 .969376702 | 1 .64062 | 0 . 10831 | 0 .333013801 |
| ZCCHC24 | -0 .310703882 | 10 .03605464 | -1 .6403 | 0 . 10836 | 0 .333087308 |
| FLCN | -0 .321963864 | 4 .981818113 | -1 .6402 | 0 . 10838 | 0 .333087308 |
| STEAP1 | 0 .244185976 | 6 .601196315 | 1 .63997 | 0 . 10844 | 0 .333118381 |
| CAMK2G | -0 . 158942684 | 8 .66241435 | -1 .6399 | 0 . 10845 | 0 .333118381 |

| CXCL12 | -0 .468869646 | 10 . 15560506 | -1 .6393 | 0 . 10859 | 0 .33346652 |
| --- | --- | --- | --- | --- | --- |
| RBL2 | -0 .243102948 | 8 .736241444 | -1 .6391 | 0 . 10863 | 0 .333522919 |
| HAUS2 | -0 .369966672 | 9 .596150469 | -1 .6389 | 0 . 10866 | 0 .333522919 |
| GABRA4 | -0 .325594226 | 5 .910729795 | -1 .6385 | 0 . 10874 | 0 .333674128 |
| KIF22 | 0 . 143408585 | 7 .28527816 | 1 .63844 | 0 . 10876 | 0 .333674128 |
| SEZ6L2 | 0 .387154669 | 6 .42283238 | 1 .63776 | 0 . 1089 | 0 .333972557 |
| RSRC2 | -0 . 173029745 | 8 .888677302 | -1 .6377 | 0 . 10891 | 0 .333972557 |
| TBC1D2B | -0 . 166204755 | 8 .050173653 | -1 .6372 | 0 . 10901 | 0 .334208415 |
| GALNT7 | -0 .269528687 | 6 .494901026 | -1 .637 | 0 . 10906 | 0 .334254895 |
| TAF5 | -0 .261802027 | 5 .389456665 | -1 .6362 | 0 . 10923 | 0 .334696921 |
| FGF17 | -0 .321073469 | 6 .405087983 | -1 .6356 | 0 . 10935 | 0 .334983352 |
| ZNF304 | -0 .242632429 | 5 .645361384 | -1 .6353 | 0 . 10942 | 0 .335131274 |
| HERC1 | -0 .255456914 | 7 .276627117 | -1 .6349 | 0 . 10949 | 0 .335190055 |
| MYO1F | 0 .249739058 | 7 .912519063 | 1 .63494 | 0 . 1095 | 0 .335190055 |
| CLPS | -0 .757985805 | 6 .206653167 | -1 .6344 | 0 . 1096 | 0 .335245271 |
| CCNB2 | 0 .450449655 | 7 .983422422 | 1 .63438 | 0 . 10961 | 0 .335245271 |
| RAB40B | -0 .286391437 | 7 .280332269 | -1 .6342 | 0 . 10966 | 0 .335245271 |
| LIPA | 0 .294738594 | 9 .0867163 | 1 .63412 | 0 . 10967 | 0 .335245271 |
| UBE2L6 | 0 .420041712 | 9 . 122490178 | 1 .63408 | 0 . 10968 | 0 .335245271 |
| CCL17 | 0 .331940487 | 3 .888844208 | 1 .63404 | 0 . 10969 | 0 .335245271 |
| HSF2 | -0 .290580707 | 5 .582722276 | -1 .634 | 0 . 1097 | 0 .335245271 |
| TRIM23 | -0 . 198248569 | 7 .075436957 | -1 .6337 | 0 . 10976 | 0 .335313574 |
| RP1-263J7 .2 | 0 .534566953 | 3 .704621293 | 1 .63361 | 0 . 10978 | 0 .335313574 |
| C1GALT1 | 0 .256874854 | 6 .586255919 | 1 .63341 | 0 . 10982 | 0 .335362457 |
| SHB | 0 .425387229 | 7 .704179892 | 1 .63295 | 0 . 10991 | 0 .335547534 |
| PDE9A | -0 .603689455 | 3 .413748639 | -1 .6329 | 0 . 10993 | 0 .335547534 |
| LGR5 | -0 .368639324 | 4 .937900154 | -1 .6326 | 0 . 10999 | 0 .335654821 |
| LOC100131532 | 0 .718764636 | 3 .063157493 | 1 .63174 | 0 . 1 1017 | 0 .335911442 |
| GREB1L | -0 .309717335 | 5 .277744164 | -1 .6316 | 0 . 1 1019 | 0 .335911442 |
| NECAB3 | 0 .207065729 | 6 .928763359 | 1 .63162 | 0 . 1 102 | 0 .335911442 |
| CDCA3 | 0 .245246127 | 6 . 129426715 | 1 .6316 | 0 . 1 102 | 0 .335911442 |
| GNA15 | 0 .523753237 | 6 .926040499 | 1 .63155 | 0 . 1 1021 | 0 .335911442 |
| CXCL5 | 0 .25812587 | 5 .693709202 | 1 .63096 | 0 . 1 1034 | 0 .336208444 |
| ITGA4 | 0 .326499295 | 5 .934252608 | 1 .6307 | 0 . 1 1039 | 0 .336290998 |
| COG2 | -0 .253923398 | 7 .717786611 | -1 .6303 | 0 . 1 1047 | 0 .336442349 |
| CD8A | 0 .756753245 | 7 .329203671 | 1 .63013 | 0 . 1 1051 | 0 .336496847 |
| PIN4 | -0 . 155100116 | 9 .386091567 | -1 .6296 | 0 . 1 1063 | 0 .336642458 |
| CDKN1A | 0 .416370282 | 9 .696268977 | 1 .62954 | 0 . 1 1064 | 0 .336642458 |
| RP11-286E11 .2 | -0 .413689964 | 3 .70025358 | -1 .6295 | 0 . 1 1064 | 0 .336642458 |
| SEPP1 | -0 .594417063 | 9 .507371027 | -1 .6293 | 0 . 1 1069 | 0 .336708167 |
| SLC16A2 | -0 .292718829 | 5 .836118673 | -1 .6288 | 0 . 1 108 | 0 .336954598 |
| GPR1 | -0 .567604038 | 4 .437150661 | -1 .6286 | 0 . 1 1084 | 0 .337002991 |
| PAX7 | 0 .6811 12048 | 3 .240377711 | 1 .62836 | 0 . 1 1089 | 0 .337068714 |
| PXDC1 | 0 .229176962 | 8 .276202336 | 1 .62819 | 0 . 1 1092 | 0 .337096802 |
| ALX3 | -0 .311267619 | 4 .863914189 | -1 .628 | 0 . 1 1096 | 0 .337128551 |
| NCAPH | 0 .386761924 | 5 .33067345 | 1 .62786 | 0 . 1 1 1 | 0 .337151734 |
| LYN | 0 .311 128573 | 8 .814063804 | 1 .62723 | 0 . 1 1 1 13 | 0 .337474969 |
| GPR15 | 0 .277268428 | 6 .646954661 | 1 .62641 | 0 . 1 1 13 | 0 .337869047 |
| CKAP5 | 0 .239898818 | 8 .25232021 | 1 .62637 | 0 . 1 1 131 | 0 .337869047 |
| MIPEP | -0 .222030034 | 8 . 176957052 | -1 .6261 | 0 . 1 1 136 | 0 .337890028 |
| EIF3F | -0 .255714991 | 10 .73921389 | -1 .626 | 0 . 1 1 139 | 0 .337890028 |
| DLST | -0 .214114274 | 8 .376588415 | -1 .626 | 0 . 1 1 14 | 0 .337890028 |
| CHD7 | 0 .262241314 | 6 .721909684 | 1 .62508 | 0 . 1 1 159 | 0 .338376586 |
| BMP8A | 0 .289075633 | 5 .690147905 | 1 .62477 | 0 . 1 1 165 | 0 .338492913 |
| GRHPR | -0 . 197532713 | 10 .46518831 | -1 .6245 | 0 . 1 1 172 | 0 .338606634 |
| USP18 | 0 .538494902 | 5 .454703779 | 1 .62433 | 0 . 1 1 175 | 0 .338615197 |

| LPCAT4 | 0 .218614235 | 7 .46849073 | 1 .62411 | 0 . 1 1 179 | 0 .338675139 |
| --- | --- | --- | --- | --- | --- |
| PDPK1 | 0 .276615331 | 6 .424516562 | 1 .62387 | 0 . 1 1 185 | 0 .338754904 |
| GABRA6 | 0 .535112623 | 3 .709575688 | 1 .62361 | 0 . 1 1 19 | 0 .338820032 |
| ADTRP | -0 .254891058 | 7 .51679673 | -1 .6235 | 0 . 1 1 192 | 0 .338820032 |
| CORO1A | 0 .635649807 | 7 .393345728 | 1 .62317 | 0 . 1 12 | 0 .338962907 |
| ITGA10 | 0 .265609629 | 7 .476988838 | 1 .62296 | 0 . 1 1204 | 0 .339014625 |
| LPPR4 | -0 .636933854 | 8 .240828941 | -1 .6226 | 0 . 1 1211 | 0 .339154075 |
| C7orf26 | 0 . 1 15109979 | 8 .696546374 | 1 .6214 | 0 . 1 1238 | 0 .339867605 |
| AP3B2 | -0 .317494742 | 4 .683974022 | -1 .6208 | 0 . 1 1251 | 0 .340200344 |
| HAND2 | 0 .423008796 | 4 .268308857 | 1 .62011 | 0 . 1 1265 | 0 .340351723 |
| HTR1B | -0 .263089125 | 5 .673528751 | -1 .6201 | 0 . 1 1266 | 0 .340351723 |
| ALDH1B1 | 0 .2263107 | 7 .052153519 | 1 .61986 | 0 . 1 1271 | 0 .340351723 |
| DSCC1 | 0 .308960535 | 6 . 157005043 | 1 .61971 | 0 . 1 1274 | 0 .340351723 |
| TVP23B | -0 .334541206 | 6 .98185707 | -1 .6197 | 0 . 1 1275 | 0 .340351723 |
| RBM23 | -0 . 161864448 | 8 .875740284 | -1 .6196 | 0 . 1 1276 | 0 .340351723 |
| DHRS12 | -0 .249948855 | 6 .78848946 | -1 .6192 | 0 . 1 1284 | 0 .340351723 |
| SDHD | -0 .266704309 | 8 .035445671 | -1 .6191 | 0 . 1 1287 | 0 .340351723 |
| ADO | -0 . 197102969 | 9 .070384223 | -1 .619 | 0 . 1 129 | 0 .340351723 |
| UST | -0 .399407738 | 6 .529447251 | -1 .6188 | 0 . 1 1295 | 0 .340351723 |
| C8orf44 | -0 .359157303 | 4 .416271547 | -1 .6187 | 0 . 1 1295 | 0 .340351723 |
| CCL11 | 0 .251326947 | 5 .358911729 | 1 .61874 | 0 . 1 1295 | 0 .340351723 |
| CLINT1 | -0 .202846824 | 8 .753398379 | -1 .6186 | 0 . 1 1298 | 0 .340351723 |
| COL5A3 | 0 .234781384 | 8 .462557493 | 1 .61846 | 0 . 1 1301 | 0 .340351723 |
| OR7C2 | 0 .482343843 | 3 .588840647 | 1 .61841 | 0 . 1 1302 | 0 .340351723 |
| KPNB1 | 0 . 191025783 | 1 1 .69038809 | 1 .61827 | 0 . 1 1305 | 0 .340351723 |
| OAZ3 | 0 .204926442 | 6 .441170894 | 1 .61827 | 0 . 1 1305 | 0 .340351723 |
| STMN1 | 0 .478349055 | 9 .647747126 | 1 .61826 | 0 . 1 1305 | 0 .340351723 |
| MSLN | -0 .338801814 | 3 .424661307 | -1 .6181 | 0 . 1 1309 | 0 .340375583 |
| GSR | 0 .286806429 | 5 .969178241 | 1 .61785 | 0 . 1 1314 | 0 .340457512 |
| CHERP | 0 . 166704231 | 8 .259327397 | 1 .61766 | 0 . 1 1318 | 0 .340496583 |
| USP9Y | -0 .371488673 | 5 .081516429 | -1 .6171 | 0 . 1 133 | 0 .340781729 |
| TOP3A | 0 . 174293783 | 7 .798077228 | 1 .61588 | 0 . 1 1357 | 0 .34136534 |
| MEOX1 | -0 .506667351 | 6 .58820758 | -1 .6158 | 0 . 1 1358 | 0 .34136534 |
| SURF1 | -0 .246876116 | 8 .874587017 | -1 .6157 | 0 . 1 1361 | 0 .34136534 |
| CASC1 | 0 .380719775 | 4 .200913237 | 1 .61568 | 0 . 1 1361 | 0 .34136534 |
| TUBA4A | 0 .508092327 | 10 .04800006 | 1 .61558 | 0 . 1 1363 | 0 .34136534 |
| CBL | -0 .458234808 | 4 .999959676 | -1 .6154 | 0 . 1 1368 | 0 .341400498 |
| TOR1A | 0 . 136416629 | 8 .552925551 | 1 .61527 | 0 . 1 137 | 0 .341400498 |
| SFXN1 | 0 .406263308 | 5 .000487746 | 1 .61478 | 0 . 1 1381 | 0 .34164269 |
| ZNF236 | -0 .236671075 | 8 .541564662 | -1 .6139 | 0 . 1 1399 | 0 .342101354 |
| ACTA2 | -0 .37022299 | 10 .20319318 | -1 .6137 | 0 . 1 1403 | 0 .342158096 |
| SYN3 | -0 .509676153 | 4 .094935887 | -1 .6136 | 0 . 1 1407 | 0 .342183362 |
| MAST2 | 0 . 143794385 | 8 .779978387 | 1 .61307 | 0 . 1 1418 | 0 .342427879 |
| MMP12 | 0 .282648288 | 6 .406616959 | 1 .61292 | 0 . 1 1421 | 0 .342443875 |
| ALDH5A1 | -0 .221067052 | 6 .480261097 | -1 .6123 | 0 . 1 1435 | 0 .342789363 |
| ROCK1 | -0 .22844212 | 8 .477747967 | -1 .6119 | 0 . 1 1444 | 0 .34297655 |
| CEP170B | -0 .285327163 | 8 .879674511 | -1 .6114 | 0 . 1 1454 | 0 .343092677 |
| LRRC47 | -0 .202417259 | 8 .840730246 | -1 .6114 | 0 . 1 1454 | 0 .343092677 |
| TFDP1 | 0 .243712332 | 8 .449579931 | 1 .61032 | 0 . 1 1478 | 0 .343737518 |
| SUCLA2 | -0 .314872374 | 6 .908840371 | -1 .6092 | 0 . 1 1502 | 0 .344359472 |
| SNTG2 | -0 .44641279 | 2 .838820824 | -1 .6091 | 0 . 1 1504 | 0 .344359472 |
| TMEM126B | -0 .233556271 | 8 .20322514 | -1 .6088 | 0 . 1 1512 | 0 .344508879 |
| TAF5L | 0 . 161514963 | 7 .476936688 | 1 .60846 | 0 . 1 1519 | 0 .344627603 |
| ACADS | 0 . 169534272 | 6 .538454019 | 1 .6082 | 0 . 1 1524 | 0 .344638452 |
| RNF13 | -0 .230290348 | 9 .475769693 | -1 .6082 | 0 . 1 1525 | 0 .344638452 |
| CENPE | 0 .378459418 | 4 .624616096 | 1 .60798 | 0 . 1 1529 | 0 .344646109 |

| C16orf95 | -0 .207797018 | 7 .520678232 | -1 .6079 | 0 . 1 1532 | 0 .344646109 |
| --- | --- | --- | --- | --- | --- |
| DDX6 | -0 .22619457 | 7 .081397989 | -1 .6078 | 0 . 1 1533 | 0 .344646109 |
| DHX16 | 0 . 180771356 | 8 .582072823 | 1 .60752 | 0 . 1 1539 | 0 .344752045 |
| FGF4 | -0 .252060213 | 7 . 152718186 | -1 .6073 | 0 . 1 1545 | 0 .344820902 |
| PCNA | 0 .342809186 | 8 .278466517 | 1 .60716 | 0 . 1 1547 | 0 .344820902 |
| SLC2A11 | 0 .285520376 | 6 .691814372 | 1 .60646 | 0 . 1 1563 | 0 .345168872 |
| PPP4C | 0 .21926241 | 7 .858905882 | 1 .60638 | 0 . 1 1564 | 0 .345168872 |
| DCTN2 | -0 . 183684256 | 9 .778336164 | -1 .6062 | 0 . 1 1568 | 0 .345203349 |
| PTPRZ1 | -0 .494926091 | 8 .266274772 | -1 .6057 | 0 . 1 1578 | 0 .345426011 |
| BPHL | -0 .21700868 | 6 .974326397 | -1 .6047 | 0 . 1 1601 | 0 .346011427 |
| ITGB8 | -0 .314261211 | 5 .266055095 | -1 .6045 | 0 . 1 1605 | 0 .346047062 |
| PTGIS | -0 .258303069 | 8 . 121473196 | -1 .6044 | 0 . 1 1609 | 0 .346076944 |
| ECHDC1 | -0 .395197609 | 6 .80261542 | -1 .6042 | 0 . 1 1612 | 0 .346112184 |
| PDE4A | -0 . 196139567 | 8 .557298641 | -1 .6037 | 0 . 1 1624 | 0 .346322367 |
| ASL | 0 . 151183179 | 7 .47782028 | 1 .60338 | 0 . 1 1631 | 0 .346322367 |
| GALC | -0 .211 182758 | 7 .70690127 | -1 .6033 | 0 . 1 1632 | 0 .346322367 |
| TRMT11 | -0 .299794006 | 5 .992537635 | -1 .6031 | 0 . 1 1637 | 0 .346322367 |
| NPAS3 | -0 .408798724 | 5 .915722112 | -1 .6031 | 0 . 1 1637 | 0 .346322367 |
| ZNF143 | -0 . 193574697 | 6 .063751693 | -1 .603 | 0 . 1 1638 | 0 .346322367 |
| CRYGEP | 0 .462672901 | 4 . 180146107 | 1 .60301 | 0 . 1 1639 | 0 .346322367 |
| CXCR2 | 0 .373845401 | 5 .017252013 | 1 .60278 | 0 . 1 1644 | 0 .346389858 |
| ZC3HAV1 | 0 . 18837883 | 8 .55053018 | 1 .60261 | 0 . 1 1648 | 0 .346419115 |
| TECR | 0 .318920664 | 9 .423063488 | 1 .60236 | 0 . 1 1653 | 0 .346505274 |
| SPOCK1 | 0 .480724606 | 7 . 1 12245758 | 1 .60158 | 0 . 1 167 | 0 .346933984 |
| ACADM | -0 .475576723 | 7 .546528392 | -1 .6014 | 0 . 1 1674 | 0 .346946552 |
| ETV1 | -0 .31192791 | 9 . 184516293 | -1 .6013 | 0 . 1 1677 | 0 .346955389 |
| SLCO2B1 | 0 .27212697 | 8 .30864785 | 1 .60115 | 0 . 1 168 | 0 .346968562 |
| MVK | -0 . 184256702 | 9 .957922364 | -1 .6008 | 0 . 1 1688 | 0 .347116369 |
| NT5C2 | -0 .247649459 | 7 .85153059 | -1 .6006 | 0 . 1 1693 | 0 .347181746 |
| FBXO28 | -0 . 160576033 | 8 .311396766 | -1 .6005 | 0 . 1 1695 | 0 .347181746 |
| KAT8 | -0 . 169671955 | 7 .897498971 | -1 .6001 | 0 . 1 1704 | 0 .347352535 |
| CNIH4 | -0 .355424647 | 5 .943265401 | -1 .5999 | 0 . 1 1707 | 0 .347352535 |
| TNPO1 | -0 .202693033 | 12 .05205206 | -1 .599 | 0 . 1 1728 | 0 .347910626 |
| CEBPE | 0 .296600806 | 5 .219586284 | 1 .59885 | 0 . 1 1731 | 0 .347912695 |
| SALL2 | -0 .227054134 | 6 .78276987 | -1 .5981 | 0 . 1 1748 | 0 .348343771 |
| CCNH | 0 .227149648 | 7 .762969821 | 1 .59784 | 0 . 1 1754 | 0 .348415278 |
| RAB28 | -0 .258147402 | 6 .433265878 | -1 .5974 | 0 . 1 1764 | 0 .348516246 |
| LMO4 | -0 .245546119 | 9 .649624115 | -1 .5971 | 0 . 1 177 | 0 .348516246 |
| EDA | -0 .256376087 | 8 . 19273916 | -1 .5969 | 0 . 1 1774 | 0 .348516246 |
| DXO | 0 . 181042592 | 8 .326102957 | 1 .5969 | 0 . 1 1775 | 0 .348516246 |
| AC004941 .5 | 0 .305168525 | 6 .064099572 | 1 .59688 | 0 . 1 1775 | 0 .348516246 |
| NPEPPS | -0 .246829109 | 9 .064083462 | -1 .5968 | 0 . 1 1776 | 0 .348516246 |
| RFXANK | 0 .241608244 | 6 .846775854 | 1 .5968 | 0 . 1 1777 | 0 .348516246 |
| IGSF9B | -0 .462858047 | 5 .990384449 | -1 .5967 | 0 . 1 1779 | 0 .348516246 |
| SNTA1 | 0 . 19634275 | 7 .817894955 | 1 .5964 | 0 . 1 1786 | 0 .348575942 |
| PPFIBP2 | -0 .273575497 | 7 .795835264 | -1 .5963 | 0 . 1 1788 | 0 .348575942 |
| TBC1D19 | -0 .373431863 | 5 .555853793 | -1 .5962 | 0 . 1 179 | 0 .348575942 |
| HAP1 | -0 .437720954 | 5 .816673248 | -1 .5959 | 0 . 1 1798 | 0 .348729336 |
| SLC39A1 | 0 .309891857 | 7 .239909024 | 1 .59498 | 0 . 1 1817 | 0 .349218035 |
| ERCC8 | -0 .260714469 | 7 .428155368 | -1 .5949 | 0 . 1 182 | 0 .349218035 |
| LINC00094 | 0 .279489764 | 6 .3247975 | 1 .59474 | 0 . 1 1823 | 0 .349233673 |
| WDR76 | 0 . 199663328 | 5 .260566301 | 1 .59396 | 0 . 1 184 | 0 .349665938 |
| PIGT | 0 .230313239 | 8 .740053563 | 1 .59333 | 0 . 1 1855 | 0 .350002493 |
| ART3 | -0 .373184211 | 4 .581196302 | -1 .593 | 0 . 1 1862 | 0 .350028804 |
| VPRBP | 0 .215502596 | 7 .285618821 | 1 .59295 | 0 . 1 1863 | 0 .350028804 |
| PVRIG | 0 .62028934 | 6 .499617503 | 1 .59292 | 0 . 1 1864 | 0 .350028804 |

| HIST1H3A | 0 .313730964 | 5 . 1 16662594 | 1 .59249 | 0 . 1 1873 | 0 .350229318 |
| --- | --- | --- | --- | --- | --- |
| ANKMY2 | -0 .238468191 | 7 .315431753 | -1 .5922 | 0 . 1 188 | 0 .350353934 |
| LUC7L3 | -0 .368603395 | 9 .90520597 | -1 .5918 | 0 . 1 1889 | 0 .350448282 |
| FSCN3 | 0 .305321742 | 7 .242897655 | 1 .59179 | 0 . 1 1889 | 0 .350448282 |
| CABP1 | 0 .246041656 | 6 .073359507 | 1 .59148 | 0 . 1 1896 | 0 .350516099 |
| CRYBB2 | 0 .484538805 | 3 .868119231 | 1 .59124 | 0 . 1 1902 | 0 .350516099 |
| APBA3 | 0 .200767288 | 8 . 189835187 | 1 .59122 | 0 . 1 1902 | 0 .350516099 |
| FCGR2C | 0 .23708348 | 7 .814793344 | 1 .59119 | 0 . 1 1903 | 0 .350516099 |
| CNOT1 | -0 . 158388661 | 9 .33610382 | -1 .591 | 0 . 1 1906 | 0 .350534745 |
| KCNH4 | -0 .382192735 | 5 .846808768 | -1 .5908 | 0 . 1 1911 | 0 .35059472 |
| APOF | -0 .424421278 | 3 .826652816 | -1 .5903 | 0 . 1 1923 | 0 .35085768 |
| NDUFS6 | 0 .235831961 | 9 .894527442 | 1 .58981 | 0 . 1 1934 | 0 .351099955 |
| GPR63 | -0 .293928919 | 4 .29544551 | -1 .589 | 0 . 1 1952 | 0 .351393448 |
| KCNA3 | 0 .347525322 | 4 .824135104 | 1 .5889 | 0 . 1 1954 | 0 .351393448 |
| NUP210 | 0 .205828245 | 8 .743607672 | 1 .58872 | 0 . 1 1959 | 0 .351393448 |
| LIMK1 | 0 . 195351733 | 7 .605185322 | 1 .58866 | 0 . 1 196 | 0 .351393448 |
| STEAP4 | 0 .389637972 | 3 .695151463 | 1 .58862 | 0 . 1 1961 | 0 .351393448 |
| IDI2-AS1 | -0 .443720299 | 6 .923757893 | -1 .5886 | 0 . 1 1962 | 0 .351393448 |
| RRP8 | 0 . 159194738 | 7 .790693543 | 1 .58847 | 0 . 1 1964 | 0 .351393448 |
| ELOVL6 | 0 .236467586 | 6 .26443636 | 1 .58833 | 0 . 1 1967 | 0 .351393448 |
| RRAD | -0 .34262416 | 5 .863208109 | -1 .5883 | 0 . 1 1969 | 0 .351393448 |
| TRAPPC11 | -0 .278787372 | 6 .836814444 | -1 .5878 | 0 . 1 1979 | 0 .351565555 |
| ZC3H3 | 0 .223349372 | 7 .288273938 | 1 .58769 | 0 . 1 1982 | 0 .351565555 |
| RPLP0 | -0 . 171378537 | 14 .65393899 | -1 .5875 | 0 . 1 1985 | 0 .351565555 |
| SUCO | -0 .323049023 | 7 .015585754 | -1 .5875 | 0 . 1 1986 | 0 .351565555 |
| MITF | -0 .366742399 | 10 .36349701 | -1 .5872 | 0 . 1 1994 | 0 .351707013 |
| B4GALNT1 | -0 .24629613 | 5 .550324274 | -1 .5868 | 0 . 12001 | 0 .351789774 |
| ICE1 | -0 .311842025 | 7 .23970261 | -1 .5868 | 0 . 12002 | 0 .351789774 |
| COLEC12 | -0 .312840952 | 7 .033148813 | -1 .5865 | 0 . 12009 | 0 .351902917 |
| EIF4G2 | 0 .22029545 | 10 .9987035 | 1 .58629 | 0 . 12014 | 0 .351964923 |
| CASP8AP2 | -0 .237758664 | 5 .775254606 | -1 .586 | 0 . 1202 | 0 .352062652 |
| SOCS1 | 0 .267532752 | 7 .860442705 | 1 .58568 | 0 . 12027 | 0 .352172714 |
| SRP72 | -0 . 174470181 | 10 .6363236 | -1 .5856 | 0 . 12029 | 0 .352172714 |
| MAN1A1 | -0 .382536832 | 7 .397032195 | -1 .5853 | 0 . 12036 | 0 .352300221 |
| LEPROTL1 | 0 . 194601939 | 9 . 187039057 | 1 .58456 | 0 . 12053 | 0 .352678448 |
| CEP72 | 0 .286461059 | 6 .391480853 | 1 .58447 | 0 . 12055 | 0 .352678448 |
| RBBP6 | -0 .25315925 | 8 .473185919 | -1 .5842 | 0 . 1206 | 0 .35275796 |
| SDHC | 0 . 194160701 | 10 .46087594 | 1 .58399 | 0 . 12066 | 0 .352834773 |
| NUPR1 | -0 .268036809 | 9 .300677831 | -1 .5837 | 0 . 12073 | 0 .352958717 |
| TXNL1 | -0 . 190513456 | 9 .227145144 | -1 .5834 | 0 . 12078 | 0 .353039361 |
| SLC23A2 | 0 .318544956 | 8 .283036417 | 1 .58283 | 0 . 12092 | 0 .353358915 |
| ZNF211 | -0 . 181604568 | 6 . 178873439 | -1 .5822 | 0 . 12107 | 0 .35370375 |
| ANP32D | -0 .286503985 | 4 .533554297 | -1 .5819 | 0 . 12113 | 0 .353808784 |
| WDR19 | -0 .26249705 | 6 .555293525 | -1 .5816 | 0 . 1212 | 0 .353881549 |
| PLK3 | 0 .23648361 | 6 .707196542 | 1 .58152 | 0 . 12122 | 0 .353881549 |
| LILRB4 | 0 .292257848 | 6 .686732602 | 1 .58143 | 0 . 12124 | 0 .353881549 |
| GATA6 | -0 .353103522 | 4 . 131098051 | -1 .581 | 0 . 12134 | 0 .353956303 |
| NMB | 0 . 182837607 | 7 .627509091 | 1 .5808 | 0 . 12139 | 0 .353956303 |
| UBR5 | -0 . 187685118 | 8 .866236317 | -1 .5807 | 0 . 1214 | 0 .353956303 |
| TGFBRAP1 | 0 .204898577 | 8 .443577801 | 1 .58073 | 0 . 1214 | 0 .353956303 |
| GOSR2 | 0 .200571868 | 8 .816392049 | 1 .58058 | 0 . 12144 | 0 .353956303 |
| MARCO | 0 .393417577 | 5 . 108836977 | 1 .5805 | 0 . 12146 | 0 .353956303 |
| ABCC8 | 0 .267860857 | 5 .817985011 | 1 .58046 | 0 . 12146 | 0 .353956303 |
| TFRC | 0 .472833305 | 9 .520776027 | 1 .58022 | 0 . 12152 | 0 .35399712 |
| JUNB | 0 .46898032 | 8 .316721247 | 1 .58008 | 0 . 12155 | 0 .35399712 |
| HDAC1 | 0 .250533643 | 8 .262594589 | 1 .57994 | 0 . 12158 | 0 .35399712 |

| TUSC3 | -0 .480408949 | 8 .403764965 | -1 .5799 | 0 . 12159 | 0 .35399712 |
| --- | --- | --- | --- | --- | --- |
| ERP29 | -0 . 17147948 | 9 .724308931 | -1 .5798 | 0 . 12163 | 0 .354016354 |
| SSR2 | -0 .245617522 | 9 .306497824 | -1 .5792 | 0 . 12175 | 0 .354287158 |
| WFS1 | 0 .211344576 | 7 .969602942 | 1 .57879 | 0 . 12185 | 0 .354349121 |
| HTR1E | -0 .492974199 | 3 .95011856 | -1 .5787 | 0 . 12186 | 0 .354349121 |
| CLNS1A | -0 .326246375 | 8 .0811 12539 | -1 .5787 | 0 . 12188 | 0 .354349121 |
| KDM6A | -0 . 165888604 | 7 .246265765 | -1 .5786 | 0 . 12188 | 0 .354349121 |
| CCDC85C | 0 .322136719 | 5 .583113541 | 1 .57835 | 0 . 12195 | 0 .354461138 |
| SCARF1 | -0 .273790262 | 5 .616573021 | -1 .5781 | 0 . 122 | 0 .354477213 |
| OSGIN2 | 0 .231871308 | 6 .793718815 | 1 .57808 | 0 . 12201 | 0 .354477213 |
| HAGH | 0 . 176362785 | 7 .516689086 | 1 .57782 | 0 . 12207 | 0 .354566548 |
| ABHD5 | -0 .315720803 | 8 .234620423 | -1 .5776 | 0 . 12212 | 0 .354629594 |
| GNAZ | -0 . 1906803 | 6 .963283949 | -1 .5774 | 0 . 12218 | 0 .354667309 |
| SNCAIP | -0 .437568572 | 4 .256179266 | -1 .5772 | 0 . 12221 | 0 .354667309 |
| PAX9 | -0 .38028679 | 6 . 140394377 | -1 .5772 | 0 . 12222 | 0 .354667309 |
| ANAPC13 | -0 . 153497775 | 9 .389874363 | -1 .5757 | 0 . 12256 | 0 .355548472 |
| SMPD3 | 0 .517046883 | 5 .238394854 | 1 .57562 | 0 . 12258 | 0 .355548472 |
| DOK2 | 0 .30881272 | 7 .076511593 | 1 .57534 | 0 . 12264 | 0 .355610587 |
| ETV5 | -0 .504916224 | 10 .64080203 | -1 .5753 | 0 . 12266 | 0 .355610587 |
| SPTLC2 | 0 . 131816713 | 8 .759712745 | 1 .57507 | 0 . 12271 | 0 .355672207 |
| PARP2 | -0 . 160238254 | 9 .500837169 | -1 .5749 | 0 . 12275 | 0 .355707892 |
| ADH6 | 0 .32841707 | 5 .75236716 | 1 .57477 | 0 . 12277 | 0 .355707892 |
| M6PR | 0 .324217499 | 7 .899167597 | 1 .57443 | 0 . 12285 | 0 .355852756 |
| COQ7 | -0 . 144504184 | 8 .613327997 | -1 .5742 | 0 . 12291 | 0 .355872669 |
| LRRC40 | -0 .287663162 | 7 .224647474 | -1 .5742 | 0 . 12292 | 0 .355872669 |
| ZNF345 | -0 .425754213 | 4 .796845986 | -1 .5738 | 0 . 12299 | 0 .356018502 |
| COQ4 | -0 .218780256 | 7 .861606286 | -1 .5732 | 0 . 12314 | 0 .356355056 |
| KLK1 | -0 .343379806 | 6 .870666783 | -1 .5728 | 0 . 12322 | 0 .356517606 |
| TMX4 | -0 .42681019 | 9 .505765687 | -1 .5725 | 0 . 12329 | 0 .356626609 |
| CDC123 | 0 .200463974 | 8 .272450789 | 1 .57239 | 0 . 12332 | 0 .356640417 |
| ESF1 | -0 .275185445 | 7 .09305878 | -1 .5719 | 0 . 12343 | 0 .356880489 |
| TRIM24 | -0 .20125998 | 7 .20989511 1 | -1 .5712 | 0 . 12359 | 0 .357154701 |
| HEATR1 | 0 . 190277915 | 8 .763461008 | 1 .57118 | 0 . 12361 | 0 .357154701 |
| CD9 | -0 .305290794 | 10 .72597462 | -1 .5711 | 0 . 12362 | 0 .357154701 |
| RAPGEF2 | -0 .239630558 | 8 .593523234 | -1 .5709 | 0 . 12367 | 0 .3572444 |
| APEX2 | 0 .225565059 | 8 .292998407 | 1 .57035 | 0 . 1238 | 0 .357521422 |
| IRS4 | -0 .317491607 | 5 .476985281 | -1 .5702 | 0 . 12384 | 0 .357564321 |
| LOC57399 | 0 .336615332 | 3 .896135294 | 1 .56981 | 0 . 12392 | 0 .357694177 |
| TNS4 | -0 .353519634 | 5 . 132834721 | -1 .5697 | 0 . 12394 | 0 .357694177 |
| G6PD | 0 .322638914 | 7 .774508464 | 1 .56922 | 0 . 12406 | 0 .357950001 |
| KDSR | -0 . 171699036 | 8 .88609138 | -1 .569 | 0 . 12412 | 0 .358022746 |
| PCYT2 | 0 . 146703571 | 7 .46806923 | 1 .56846 | 0 . 12424 | 0 .35823467 |
| NBR1 | -0 .210979287 | 9 .226889762 | -1 .5683 | 0 . 12427 | 0 .35823467 |
| NRXN2 | 0 .372785187 | 5 .867500386 | 1 .56831 | 0 . 12427 | 0 .35823467 |
| EFNA3 | -0 .804911 1 1 1 | 7 .805809473 | -1 .5679 | 0 . 12436 | 0 .35840055 |
| EGR2 | 0 .31403016 | 6 .29948271 | 1 .56766 | 0 . 12442 | 0 .358446867 |
| PRDX3 | -0 . 160609724 | 9 .764607246 | -1 .5676 | 0 . 12443 | 0 .358446867 |
| VAC14 | 0 . 194485775 | 8 .020099952 | 1 .5675 | 0 . 12446 | 0 .358446867 |
| SUPV3L1 | -0 . 157463922 | 7 .343296325 | -1 .5669 | 0 . 1246 | 0 .358727475 |
| IFNGR1 | -0 .266974162 | 9 .075213093 | -1 .5668 | 0 . 12462 | 0 .358727475 |
| TYR | 0 .452653923 | 1 1 . 12839716 | 1 .56645 | 0 . 12471 | 0 .358909603 |
| BPNT1 | 0 .370977036 | 4 .653615278 | 1 .56597 | 0 . 12482 | 0 .359074369 |
| CTSK | 0 .386490607 | 10 .22646768 | 1 .56592 | 0 . 12483 | 0 .359074369 |
| CYHR1 | 0 .213723563 | 7 .889266214 | 1 .56583 | 0 . 12485 | 0 .359074369 |
| NPC1 | 0 . 183808341 | 8 .258299439 | 1 .56498 | 0 . 12505 | 0 .359527522 |
| AIFM1 | 0 . 195747762 | 7 . 126355396 | 1 .56492 | 0 . 12507 | 0 .359527522 |

| MTRF1 | -0 .323322811 | 5 . 144308172 | -1 .5641 | 0 . 12526 | 0 .359930034 |
| --- | --- | --- | --- | --- | --- |
| LYPD3 | 0 .613867912 | 10 .70807705 | 1 .56408 | 0 . 12526 | 0 .359930034 |
| UQCR11 | -0 .320470403 | 8 .741989972 | -1 .5635 | 0 . 12541 | 0 .36024397 |
| COQ2 | 0 .318788625 | 5 .808533651 | 1 .5633 | 0 . 12545 | 0 .36024397 |
| PSMB6 | 0 .206659181 | 9 .555628107 | 1 .56322 | 0 . 12547 | 0 .36024397 |
| MLST8 | 0 .262001497 | 6 .950475404 | 1 .56312 | 0 . 12549 | 0 .36024397 |
| MRPS18C | -0 .280529412 | 4 .732181604 | -1 .5628 | 0 . 12557 | 0 .360382029 |
| AGPAT1 | -0 .217125041 | 10 .00935421 | -1 .5623 | 0 . 12568 | 0 .360639607 |
| ZCCHC10 | -0 .322149924 | 5 . 164692947 | -1 .5621 | 0 . 12574 | 0 .360707452 |
| F10 | -0 .378489439 | 6 .372350619 | -1 .5616 | 0 . 12584 | 0 .360916113 |
| IL7 | -0 .304395475 | 4 .083343057 | -1 .5615 | 0 . 12588 | 0 .360958004 |
| RAB9BP1 | 0 .368397067 | 4 .285843835 | 1 .56124 | 0 . 12593 | 0 .361021086 |
| QTRTD1 | 0 .305741217 | 6 .09988909 | 1 .56055 | 0 . 12609 | 0 .361393464 |
| SEMA4A | 0 .316393313 | 8 .208722759 | 1 .56045 | 0 . 12612 | 0 .361393464 |
| CCDC51 | -0 . 156614935 | 8 . 167336168 | -1 .5598 | 0 . 12627 | 0 .361736192 |
| SIRPG | 0 .404014436 | 6 .255542603 | 1 .55964 | 0 . 12631 | 0 .361736192 |
| HCRT | -0 .281593686 | 5 .281555742 | -1 .5595 | 0 . 12634 | 0 .361736192 |
| EMD | 0 .200438839 | 8 .37846326 | 1 .55945 | 0 . 12635 | 0 .361736192 |
| HOXA11 | 0 .272723264 | 7 .023071056 | 1 .55868 | 0 . 12654 | 0 .36217552 |
| NUDC | 0 . 163045819 | 9 .593657715 | 1 .55853 | 0 . 12657 | 0 .36219334 |
| DCLRE1A | -0 .242312714 | 5 .601654068 | -1 .558 | 0 . 1267 | 0 .362430373 |
| MYO16 | -0 .430276915 | 4 .211881263 | -1 .5579 | 0 . 12671 | 0 .362430373 |
| POLD3 | -0 . 146557544 | 7 .406736137 | -1 .5571 | 0 . 12691 | 0 .362822069 |
| MYL5 | -0 . 191138932 | 6 .524344686 | -1 .5571 | 0 . 12692 | 0 .362822069 |
| MOSPD2 | -0 .23603653 | 7 .934958238 | -1 .5569 | 0 . 12695 | 0 .362822069 |
| QKI | -0 .212346969 | 10 .42888015 | -1 .5569 | 0 . 12696 | 0 .362822069 |
| KCNJ6 | -0 .473335737 | 4 .424363783 | -1 .5567 | 0 . 127 | 0 .36283781 |
| ZNF205 | 0 .400408602 | 5 .071725169 | 1 .55643 | 0 . 12707 | 0 .362961228 |
| HLA-DOA | 0 .225136964 | 7 .627472914 | 1 .55609 | 0 . 12715 | 0 .36310959 |
| CXCL14 | -0 .482174048 | 1 1 .60711 1 17 | -1 .5559 | 0 . 1272 | 0 .363161596 |
| RIMBP2 | -0 .294516829 | 4 .705015377 | -1 .5553 | 0 . 12733 | 0 .36345483 |
| ANXA4 | -0 .284773275 | 9 .544628533 | -1 .5547 | 0 . 12749 | 0 .363639659 |
| KCNK13 | 0 .229431331 | 5 .506428435 | 1 .55467 | 0 . 12749 | 0 .363639659 |
| SNRPD2 | -0 .242862021 | 10 .42618074 | -1 .5545 | 0 . 12752 | 0 .363639659 |
| CNOT3 | 0 .26061781 | 7 . 1 18810176 | 1 .5545 | 0 . 12753 | 0 .363639659 |
| ITSN1 | -0 . 134702781 | 9 .080520577 | -1 .5545 | 0 . 12754 | 0 .363639659 |
| RIBC2 | 0 .582557596 | 2 .526702051 | 1 .55316 | 0 . 12785 | 0 .36443946 |
| FUT8 | -0 .322979989 | 6 .433049928 | -1 .5523 | 0 . 12805 | 0 .364866777 |
| BC069739 | -0 .351715944 | 3 .508367799 | -1 .5523 | 0 . 12806 | 0 .364866777 |
| HIP1 | 0 . 18048392 | 7 .562309227 | 1 .55203 | 0 . 12812 | 0 .364918253 |
| BRCC3 | 0 . 120953914 | 7 .733970118 | 1 .55197 | 0 . 12813 | 0 .364918253 |
| TH | -0 .463114789 | 3 .757970639 | -1 .5513 | 0 . 12828 | 0 .36521101 |
| CDK18 | 0 .461602925 | 4 .901298886 | 1 .55129 | 0 . 1283 | 0 .36521101 |
| CCDC59 | -0 .359903443 | 6 .028965258 | -1 .5506 | 0 . 12845 | 0 .365570563 |
| THOC5 | 0 . 146735427 | 7 .332769093 | 1 .55032 | 0 . 12853 | 0 .365705848 |
| TARBP1 | -0 .207823666 | 7 .637554551 | -1 .5502 | 0 . 12857 | 0 .365716202 |
| RNF219 | -0 . 198322608 | 6 .089848126 | -1 .5499 | 0 . 12862 | 0 .365716202 |
| MED27 | -0 . 177852281 | 9 .286504035 | -1 .5499 | 0 . 12863 | 0 .365716202 |
| USP3 | -0 . 161590836 | 7 .798141966 | -1 .5498 | 0 . 12865 | 0 .365716202 |
| PRMT5 | -0 .328475158 | 7 .70136858 | -1 .5497 | 0 . 12869 | 0 .365741813 |
| PRKCDBP | 0 .274414359 | 9 .2229802 | 1 .5493 | 0 . 12877 | 0 .365904503 |
| MIR1244-3 | -0 . 133450928 | 10 .21539173 | -1 .5491 | 0 . 12883 | 0 .365980308 |
| HIST1H2AM | 0 .307372566 | 5 .518834164 | 1 .54843 | 0 . 12898 | 0 .366323449 |
| PPM1G | 0 .203930844 | 7 .500795581 | 1 .54833 | 0 . 12901 | 0 .366323449 |
| TSPAN14 | -0 . 194234486 | 8 .689206587 | -1 .5479 | 0 . 12912 | 0 .3665622 |
| SYDE1 | -0 .200092154 | 9 .591697537 | -1 .5469 | 0 . 12935 | 0 .367136139 |

| RPL3L | 0 .482829703 | 4 .678902205 | 1 .54672 | 0 . 12939 | 0 .367169297 |
| --- | --- | --- | --- | --- | --- |
| NAT10 | 0 .331160373 | 7 .908259619 | 1 .54634 | 0 . 12948 | 0 .367246592 |
| SLC39A14 | 0 .278922355 | 7 .591353132 | 1 .54629 | 0 . 1295 | 0 .367246592 |
| LTC4S | -0 .562790567 | 3 .645310164 | -1 .5462 | 0 . 12952 | 0 .367246592 |
| PSMA5 | 0 .200874938 | 8 .688444008 | 1 .54607 | 0 . 12955 | 0 .367246592 |
| ZNF16 | -0 . 166058556 | 6 .724735891 | -1 .546 | 0 . 12957 | 0 .367246592 |
| CSTA | 0 .861034271 | 10 .33062112 | 1 .54524 | 0 . 12975 | 0 .367684425 |
| AC074212 .6 | 0 .740765685 | 2 .61056569 | 1 .54417 | 0 . 13001 | 0 .368335339 |
| DKK3 | -0 .438052166 | 9 .231702297 | -1 .5436 | 0 . 13014 | 0 .368624602 |
| CDHR2 | -0 .23023937 | 6 .773529693 | -1 .5432 | 0 . 13024 | 0 .368817839 |
| SIN3B | 0 .228156039 | 7 .483813588 | 1 .54291 | 0 . 13032 | 0 .368952892 |
| ALPK1 | -0 .360869748 | 7 . 183092743 | -1 .5427 | 0 . 13036 | 0 .368991815 |
| UCHL3 | 0 .346120442 | 7 .72681163 | 1 .54156 | 0 . 13064 | 0 .369708645 |
| ABCG1 | 0 .210002037 | 7 .707858089 | 1 .54092 | 0 . 1308 | 0 .370060718 |
| SLC24A3 | -0 .32181691 | 6 .779332708 | -1 .5408 | 0 . 13082 | 0 .370060718 |
| RDH16 | 0 .601913916 | 4 .294879741 | 1 .54053 | 0 . 13089 | 0 .370166514 |
| PPP1R13B | -0 .214819045 | 6 .43574803 | -1 .5393 | 0 . 1312 | 0 .370953488 |
| MTCH2 | 0 . 186195453 | 9 .056003688 | 1 .53881 | 0 . 13131 | 0 .371102807 |
| ERCC4 | 0 .321292655 | 6 .804318162 | 1 .53881 | 0 . 13131 | 0 .371102807 |
| ARRB2 | 0 .427098661 | 7 . 1 1934351 | 1 .53841 | 0 . 13141 | 0 .371295252 |
| NPM3 | -0 .206817112 | 6 .932868267 | -1 .5382 | 0 . 13147 | 0 .371347236 |
| AATF | 0 . 193309065 | 8 .985607728 | 1 .53809 | 0 . 13149 | 0 .371347236 |
| FAXDC2 | -0 .251711578 | 9 .859342393 | -1 .5379 | 0 . 13153 | 0 .371370996 |
| KRTAP1-1 | 0 .486329726 | 5 .5342066 | 1 .53782 | 0 . 13155 | 0 .371370996 |
| SNRPG | -0 .302440619 | 7 .834096348 | -1 .5375 | 0 . 13164 | 0 .371490666 |
| ITPR1 | -0 .214334172 | 7 .296045648 | -1 .5372 | 0 . 13169 | 0 .371490666 |
| ARID3A | 0 .267010751 | 5 .277686178 | 1 .53719 | 0 . 13171 | 0 .371490666 |
| SIGIRR | 0 .28578126 | 8 .785000329 | 1 .53716 | 0 . 13172 | 0 .371490666 |
| MYBL2 | 0 .231068875 | 7 .992170741 | 1 .53682 | 0 . 1318 | 0 .371643626 |
| TRPC7 | 0 .308145949 | 2 . 105305598 | 1 .53602 | 0 . 13199 | 0 .372038785 |
| AUNIP | 0 .288027196 | 6 .845249928 | 1 .53582 | 0 . 13204 | 0 .372038785 |
| SLC26A3 | 0 .273867385 | 5 .302440598 | 1 .53581 | 0 . 13205 | 0 .372038785 |
| A2M | 0 .30402986 | 9 .999970477 | 1 .53576 | 0 . 13206 | 0 .372038785 |
| B3GAT1 | 0 .299657786 | 4 .656985454 | 1 .53518 | 0 . 1322 | 0 .37235511 1 |
| FBXL12 | 0 . 144019798 | 7 .957577987 | 1 .53505 | 0 . 13223 | 0 .372360905 |
| TRIM32 | -0 . 154274932 | 7 .390399505 | -1 .5347 | 0 . 13231 | 0 .372494843 |
| NMUR1 | 0 .510155845 | 3 .202453428 | 1 .53454 | 0 . 13236 | 0 .372544576 |
| PDZRN3 | -0 .535641524 | 6 .633135506 | -1 .5344 | 0 . 13239 | 0 .372547513 |
| ATPIF1 | 0 .285231797 | 8 . 163496754 | 1 .53419 | 0 . 13244 | 0 .372547513 |
| ZNF669 | 0 .211834521 | 7 .023528341 | 1 .53418 | 0 . 13245 | 0 .372547513 |
| CFLAR | -0 . 199565353 | 10 .88723244 | -1 .5337 | 0 . 13256 | 0 .372732151 |
| KIF21B | -0 .493127751 | 3 .474249302 | -1 .5337 | 0 . 13257 | 0 .372732151 |
| CYP11B1 | -0 .24511 1052 | 4 .747735014 | -1 .5333 | 0 . 13267 | 0 .372934878 |
| ABO | 0 .266633114 | 5 .485289765 | 1 .53276 | 0 . 13279 | 0 .373190432 |
| CYP3A4 | -0 .385146172 | 7 .527788835 | -1 .5321 | 0 . 13297 | 0 .373589569 |
| RBP4 | -0 .40537027 | 4 .808662166 | -1 .5319 | 0 . 133 | 0 .373610193 |
| TKTL1 | 0 .337107784 | 5 .670473235 | 1 .53122 | 0 . 13317 | 0 .373960466 |
| LRRC8D | -0 .234553923 | 8 .40263547 | -1 .531 | 0 . 13323 | 0 .373960466 |
| AC068039 .4 | -0 .375783496 | 7 .725184708 | -1 .5308 | 0 . 13327 | 0 .373960466 |
| DESI1 | 0 . 159268542 | 8 .046895861 | 1 .53063 | 0 . 13332 | 0 .373960466 |
| FAM155A | 0 .338920095 | 4 .629610156 | 1 .53059 | 0 . 13333 | 0 .373960466 |
| ALDOB | 0 .243766636 | 8 . 126439526 | 1 .53042 | 0 . 13337 | 0 .373960466 |
| KDELC1 | 0 .240748204 | 5 .486893503 | 1 .53038 | 0 . 13338 | 0 .373960466 |
| DAXX | 0 . 185576446 | 8 .016858373 | 1 .53031 | 0 . 1334 | 0 .373960466 |
| CHD4 | 0 . 168261925 | 10 .55406649 | 1 .53024 | 0 . 13342 | 0 .373960466 |
| HCRTR1 | -0 .649418618 | 3 .621049788 | -1 .5302 | 0 . 13343 | 0 .373960466 |

| COG4 | -0 .246115193 | 7 .438037227 | -1 .5297 | 0 . 13356 | 0 .374255869 |
| --- | --- | --- | --- | --- | --- |
| NIN | 0 .381557823 | 3 .808614146 | 1 .52938 | 0 . 13363 | 0 .374361825 |
| NRN1 | -0 .433837389 | 7 .543235796 | -1 .5292 | 0 . 13368 | 0 .374417162 |
| ZNF528 | 0 .431514917 | 6 .018538917 | 1 .52878 | 0 . 13378 | 0 .374608834 |
| RAC2 | 0 .564011591 | 8 .854705108 | 1 .52819 | 0 . 13392 | 0 .374938733 |
| PPAP2C | -0 .303307749 | 6 .387720448 | -1 .528 | 0 . 13398 | 0 .375013396 |
| RPL10L | -0 .525307146 | 5 .705981532 | -1 .5273 | 0 . 13415 | 0 .375406723 |
| ERICH1 | -0 .493170676 | 2 .651913847 | -1 .5265 | 0 . 13434 | 0 .375840374 |
| LACTB2 | 0 .360346169 | 5 .216998654 | 1 .52639 | 0 . 13437 | 0 .375851823 |
| CHAT | -0 .465748506 | 4 .355851422 | -1 .5257 | 0 . 13453 | 0 .376214598 |
| C11orf63 | -0 .250210473 | 5 . 187719931 | -1 .5247 | 0 . 13478 | 0 .376786941 |
| CHST12 | 0 .275733104 | 7 .515946955 | 1 .52468 | 0 . 13479 | 0 .376786941 |
| NIPSNAP3B | -0 .239071174 | 5 . 175189879 | -1 .5246 | 0 . 13482 | 0 .376788901 |
| ZDHHC18 | 0 .234141689 | 7 .226530628 | 1 .52407 | 0 . 13495 | 0 .377046078 |
| KCNMB1 | -0 .202907711 | 6 .81730177 | -1 .5239 | 0 . 13498 | 0 .377046078 |
| HMBOX1 | -0 .315490708 | 8 . 1 10679564 | -1 .5237 | 0 . 13505 | 0 .377162348 |
| PABPN1 | -0 .242455335 | 8 . 147624507 | -1 .5233 | 0 . 13514 | 0 .37734235 |
| HOXA6 | -0 .297336114 | 5 .658809095 | -1 .5231 | 0 . 13518 | 0 .37734235 |
| EFR3B | -0 .256507378 | 7 . 143707843 | -1 .523 | 0 . 1352 | 0 .37734235 |
| KNTC1 | 0 .287108949 | 6 .639900058 | 1 .52281 | 0 . 13526 | 0 .377421244 |
| CREB3 | -0 .216519711 | 7 .385934967 | -1 .5225 | 0 . 13533 | 0 .377522891 |
| INHBE | -0 .373992326 | 4 .20204577 | -1 .5223 | 0 . 13539 | 0 .377573905 |
| CLCN2 | 0 .226430703 | 5 .937178451 | 1 .52223 | 0 . 13541 | 0 .377573905 |
| EZH2 | 0 .401335857 | 5 .851276986 | 1 .5214 | 0 . 13561 | 0 .378036883 |
| TLR4 | -0 .395869986 | 6 . 14452296 | -1 .5213 | 0 . 13563 | 0 .378036883 |
| ZNF500 | -0 . 171251017 | 8 .690542046 | -1 .5209 | 0 . 13575 | 0 .378273588 |
| GBE1 | -0 .337189826 | 7 .768488591 | -1 .5204 | 0 . 13587 | 0 .378295897 |
| PCDHB13 | 0 .2725868 | 6 .220171694 | 1 .52036 | 0 . 13587 | 0 .378295897 |
| ENPEP | -0 .283132488 | 5 .234275414 | -1 .5203 | 0 . 13588 | 0 .378295897 |
| FBXL15 | -0 .210697143 | 6 .210191287 | -1 .5203 | 0 . 13588 | 0 .378295897 |
| KREMEN2 | -0 .274890772 | 6 .333172777 | -1 .5202 | 0 . 13591 | 0 .378295897 |
| MKNK1 | -0 . 148898985 | 8 .254764215 | -1 .5199 | 0 . 136 | 0 .378452367 |
| DOLPP1 | -0 . 180911045 | 7 .636428476 | -1 .5198 | 0 . 13602 | 0 .378452367 |
| TOX | 0 .274772585 | 5 .89986875 | 1 .51915 | 0 . 13618 | 0 .37879998 |
| GFRA3 | -0 .329155546 | 5 .885995113 | -1 .5187 | 0 . 13629 | 0 .378977094 |
| PIGK | -0 .306642879 | 6 .377136692 | -1 .5187 | 0 . 1363 | 0 .378977094 |
| ZNF571 | -0 .363203625 | 3 .889412871 | -1 .5185 | 0 . 13633 | 0 .378979246 |
| DROSHA | -0 .222409486 | 8 .009433717 | -1 .5182 | 0 . 13642 | 0 .379129449 |
| ASAP1-IT1 | -0 .396251908 | 5 .48083453 | -1 .5172 | 0 . 13667 | 0 .379743169 |
| CNTNAP2 | 0 . 180238028 | 6 .652086098 | 1 .51708 | 0 . 1367 | 0 .379743169 |
| BCL2L1 | 0 . 166892569 | 8 .30011 1901 | 1 .51584 | 0 . 13701 | 0 .380524171 |
| SPI1 | 0 .50374496 | 6 .314931665 | 1 .51562 | 0 . 13707 | 0 .380524171 |
| KCNMB4 | -0 .239626217 | 6 .811749118 | -1 .5156 | 0 . 13707 | 0 .380524171 |
| UBN1 | 0 . 172330686 | 9 .847932243 | 1 .51519 | 0 . 13718 | 0 .380729552 |
| RSAD1 | -0 . 192309995 | 7 .457871739 | -1 .5147 | 0 . 13729 | 0 .380916432 |
| PROSER1 | 0 . 165471961 | 7 .661326813 | 1 .51464 | 0 . 13731 | 0 .380916432 |
| FOXK2 | 0 . 177006205 | 7 .46703379 | 1 .51456 | 0 . 13733 | 0 .380916432 |
| CD63 | 0 .216021136 | 1 1 .94445345 | 1 .51358 | 0 . 13758 | 0 .381438815 |
| PPP1R16B | 0 .443784576 | 6 .98352188 | 1 .51358 | 0 . 13758 | 0 .381438815 |
| TAF1B | 0 .21762344 | 6 .518306678 | 1 .51302 | 0 . 13772 | 0 .381744818 |
| MAP3K6 | -0 .275945677 | 7 .250227971 | -1 .5127 | 0 . 1378 | 0 .381884605 |
| GAD2 | -0 .220135443 | 7 .359837459 | -1 .5126 | 0 . 13784 | 0 .381885832 |
| MDC1 | 0 .211622638 | 7 .738593431 | 1 .51192 | 0 . 138 | 0 .382265266 |
| SMG8 | -0 . 135613894 | 7 .58958616 | -1 .5116 | 0 . 13808 | 0 .382386175 |
| QDPR | 0 .435334433 | 9 . 129611544 | 1 .51101 | 0 . 13823 | 0 .382734422 |
| RNF11 1 | -0 .226608656 | 6 .393551706 | -1 .5105 | 0 . 13836 | 0 .383000465 |

| TIMM13 | 0 .204038107 | 8 .773683511 | 1 .50988 | 0 . 13852 | 0 .383328329 |
| --- | --- | --- | --- | --- | --- |
| APLP2 | -0 . 132954202 | 1 1 .82102868 | -1 .5098 | 0 . 13854 | 0 .383328329 |
| OSTM1 | -0 .453729831 | 7 .865202745 | -1 .5093 | 0 . 13866 | 0 .383575605 |
| STMN2 | -0 .305736203 | 7 . 131523475 | -1 .5091 | 0 . 13871 | 0 .383575605 |
| MRPS35 | -0 . 182156228 | 8 .216775467 | -1 .5091 | 0 . 13872 | 0 .383575605 |
| SLC25A1 | -0 .231327665 | 8 .38792803 | -1 .5088 | 0 . 13879 | 0 .383579691 |
| PA2G4 | 0 . 194245828 | 10 .05955945 | 1 .50882 | 0 . 13879 | 0 .383579691 |
| PSMG2 | -0 . 160552721 | 9 .373780447 | -1 .5087 | 0 . 13881 | 0 .383579691 |
| PIGO | -0 . 142692301 | 7 .953106455 | -1 .5083 | 0 . 13892 | 0 .383764068 |
| SOX1 | 0 .432010845 | 2 .707390893 | 1 .50823 | 0 . 13894 | 0 .383764068 |
| FRS2 | -0 .533422833 | 3 .650270372 | -1 .5079 | 0 . 13902 | 0 .383828942 |
| ANKRD36B | -0 .403960681 | 6 .554101963 | -1 .5079 | 0 . 13903 | 0 .383828942 |
| NCK1 | -0 . 196584401 | 8 .537261167 | -1 .5077 | 0 . 13907 | 0 .383876634 |
| SIK2 | -0 .287756884 | 6 .798608949 | -1 .5075 | 0 . 13913 | 0 .383948625 |
| PQLC3 | -0 .269154264 | 5 .464466828 | -1 .5073 | 0 . 13918 | 0 .384010178 |
| ZFAND3 | 0 . 145868103 | 8 .725329326 | 1 .50643 | 0 . 1394 | 0 .384531045 |
| S100A6 | 0 .295436166 | 1 1 .71645904 | 1 .50577 | 0 . 13957 | 0 .384910473 |
| HMGXB3 | 0 . 170187758 | 8 .813072042 | 1 .50532 | 0 . 13969 | 0 .385143002 |
| ARHGAP12 | -0 .298614852 | 5 .670961187 | -1 .5049 | 0 . 13978 | 0 .385312677 |
| RPS11 | -0 .574986061 | 9 .248800743 | -1 .5048 | 0 . 13981 | 0 .385312677 |
| STX16 | -0 .201948508 | 9 .048221412 | -1 .5044 | 0 . 13992 | 0 .38547926 |
| MTG1 | 0 . 171367668 | 7 .258711842 | 1 .50429 | 0 . 13995 | 0 .38547926 |
| VCPKMT | -0 .249212012 | 5 .390274648 | -1 .5042 | 0 . 13997 | 0 .38547926 |
| DHPS | -0 . 180830872 | 9 .25638795 | -1 .504 | 0 . 14002 | 0 .38547926 |
| FAM178A | -0 .201389959 | 7 .075361943 | -1 .504 | 0 . 14003 | 0 .38547926 |
| OR6A2 | -0 .396617702 | 5 .959817625 | -1 .5039 | 0 . 14005 | 0 .38547926 |
| ZSCAN9 | 0 .279817758 | 6 .615220061 | 1 .50375 | 0 . 14009 | 0 .385488469 |
| WBP5 | 0 . 199494439 | 8 .769454152 | 1 .50319 | 0 . 14023 | 0 .385799504 |
| RANBP1 | 0 .22911 1399 | 9 .054353538 | 1 .50295 | 0 . 1403 | 0 .3858174 |
| CUL9 | -0 .284778513 | 5 .68567581 | -1 .5029 | 0 . 1403 | 0 .3858174 |
| CLGN | 0 .362486284 | 3 .649611 157 | 1 .50263 | 0 . 14038 | 0 .385941218 |
| BCL2L11 | 0 .20726864 | 5 .826377512 | 1 .50244 | 0 . 14043 | 0 .385941218 |
| RRAS2 | -0 .218726779 | 7 .748810556 | -1 .5024 | 0 . 14044 | 0 .385941218 |
| ZNF471 | -0 .417168796 | 5 .240791451 | -1 .5022 | 0 . 14048 | 0 .385966374 |
| SMG6 | -0 .243507172 | 6 .777940863 | -1 .5018 | 0 . 14058 | 0 .38616187 |
| EPN3 | 0 .468936107 | 6 .388052368 | 1 .50165 | 0 . 14063 | 0 .386218372 |
| CASC5 | -0 .352909879 | 3 .545377943 | -1 .5014 | 0 . 14069 | 0 .386295202 |
| FNTA | -0 . 18519691 | 9 .744986518 | -1 .5011 | 0 . 14076 | 0 .386382184 |
| ZNF354A | -0 .285849437 | 5 .491260173 | -1 .5009 | 0 . 14081 | 0 .386382184 |
| MS4A1 | 0 .38114142 | 7 . 150762044 | 1 .50094 | 0 . 14081 | 0 .386382184 |
| MORC1 | 0 .49030787 | 2 .962150484 | 1 .49915 | 0 . 14127 | 0 .387560376 |
| SPRY2 | -0 .539628695 | 7 .743943013 | -1 .4987 | 0 . 1414 | 0 .387775746 |
| PRF1 | 0 .417795689 | 6 .938049533 | 1 .49851 | 0 . 14144 | 0 .387775746 |
| C11orf80 | -0 .288718882 | 6 .401968093 | -1 .4985 | 0 . 14144 | 0 .387775746 |
| PLS1 | -0 .315598242 | 5 .30719212 | -1 .4982 | 0 . 14151 | 0 .387864714 |
| PGGT1B | 0 .328026356 | 4 .666636094 | 1 .49768 | 0 . 14165 | 0 .388181634 |
| DAB1 | 0 .639507419 | 3 .251402915 | 1 .49756 | 0 . 14169 | 0 .388181686 |
| CHD2 | -0 .376350245 | 6 .963196087 | -1 .4974 | 0 . 14172 | 0 .38820019 |
| SLC11A1 | 0 .228807531 | 8 .503585907 | 1 .49702 | 0 . 14183 | 0 .388399074 |
| MOCS2 | -0 .294393782 | 6 .542074501 | -1 .4967 | 0 . 1419 | 0 .388525343 |
| KRT9 | 0 .557039166 | 6 .806000492 | 1 .49629 | 0 . 14202 | 0 .388745488 |
| GPR45 | 0 .559583079 | 3 .786230637 | 1 .49602 | 0 . 14208 | 0 .388850985 |
| KRT38 | -0 .213263785 | 6 .641600836 | -1 .4957 | 0 . 14217 | 0 .389002878 |
| XRCC5 | -0 . 189171875 | 10 .49710906 | -1 .495 | 0 . 14234 | 0 .38938401 |
| CFTR | -0 .247033566 | 6 .867311 193 | -1 .4941 | 0 . 14258 | 0 .38995422 |
| SLMO2 | 0 .377003208 | 6 .863723085 | 1 .49394 | 0 . 14263 | 0 .389991332 |

| RSPH6A | 0 .279308573 | 6 .256457864 | 1 .49367 | 0 . 1427 | 0 .390102584 |
| --- | --- | --- | --- | --- | --- |
| PCDHB11 | -0 .523516917 | 3 . 163000704 | -1 .4934 | 0 . 14277 | 0 .390204182 |
| CCZ1B | 0 .277307225 | 5 .572030693 | 1 .49323 | 0 . 14281 | 0 .390204182 |
| GPRC5B | -0 .287437835 | 8 .604834273 | -1 .4931 | 0 . 14285 | 0 .390204182 |
| AGRN | 0 .260646481 | 1 1 . 10229317 | 1 .49297 | 0 . 14288 | 0 .390204182 |
| MRPL15 | 0 .282332413 | 8 .272453292 | 1 .49293 | 0 . 14289 | 0 .390204182 |
| CHST8 | 0 .204643916 | 6 .397663984 | 1 .49278 | 0 . 14293 | 0 .39022818 |
| PCDHA9 | 0 .43675383 | 6 .308213507 | 1 .49264 | 0 . 14297 | 0 .390238216 |
| DCHS2 | -0 .344430806 | 3 .39729797 | -1 .4921 | 0 . 1431 | 0 .39052222 |
| AKAP9 | -0 . 194287717 | 7 .450661572 | -1 .4918 | 0 . 14319 | 0 .390683468 |
| ATHL1 | 0 .316560785 | 6 .410503147 | 1 .49161 | 0 . 14323 | 0 .390719155 |
| SNX17 | -0 .267264901 | 7 .916526327 | -1 .4913 | 0 . 14332 | 0 .390879379 |
| APBA2 | 0 .376062787 | 7 .38676104 | 1 .49102 | 0 . 14339 | 0 .39096934 |
| SYCE1L | -0 .243605346 | 3 .876591784 | -1 .4906 | 0 . 14349 | 0 .391156731 |
| TET3 | -0 .233441826 | 5 .872327735 | -1 .4903 | 0 . 14357 | 0 .391292411 |
| CALCOCO2 | -0 . 144013892 | 8 .787384901 | -1 .4899 | 0 . 14369 | 0 .391492896 |
| RAG2 | 0 .499382352 | 3 . 130191029 | 1 .48981 | 0 . 14371 | 0 .391492896 |
| TRBC1 | 0 .716714325 | 10 . 16741355 | 1 .4896 | 0 . 14376 | 0 .39151936 |
| RANBP6 | -0 .232173969 | 5 .938143173 | -1 .4895 | 0 . 14378 | 0 .39151936 |
| HOXB3 | -0 .718006245 | 5 .369887467 | -1 .4887 | 0 . 144 | 0 .392042795 |
| SLC9A3R2 | -0 .505711286 | 3 .748878598 | -1 .4884 | 0 . 14408 | 0 .392139866 |
| PPFIBP1 | 0 . 184744644 | 10 .36899274 | 1 .4883 | 0 . 1441 | 0 .392139866 |
| DNAJC9 | 0 . 152289127 | 8 .559754413 | 1 .48819 | 0 . 14413 | 0 .392139866 |
| REXO4 | -0 . 169798288 | 6 .782704734 | -1 .4875 | 0 . 14433 | 0 .392584693 |
| CD2AP | -0 .464946474 | 6 .309673162 | -1 .4867 | 0 . 14451 | 0 .392863327 |
| EAF2 | 0 .480412195 | 3 .69372158 | 1 .48673 | 0 . 14452 | 0 .392863327 |
| PSME3 | 0 . 147780289 | 10 . 14263877 | 1 .48671 | 0 . 14452 | 0 .392863327 |
| ERGIC3 | -0 .202885929 | 9 .356948223 | -1 .4864 | 0 . 1446 | 0 .392986152 |
| SLC2A9 | 0 .246277685 | 7 .46170143 | 1 .48622 | 0 . 14465 | 0 .393041369 |
| MTHFR | 0 .22577772 | 6 .815387783 | 1 .48589 | 0 . 14474 | 0 .393153217 |
| TNFRSF17 | 0 .366145013 | 4 .816303256 | 1 .48556 | 0 . 14482 | 0 .393153217 |
| PIAS2 | 0 .472407515 | 7 . 105602863 | 1 .48547 | 0 . 14485 | 0 .393153217 |
| GPR50 | -0 .226237956 | 5 .723415787 | -1 .4854 | 0 . 14486 | 0 .393153217 |
| NAA10 | 0 . 158621386 | 8 .31052211 1 | 1 .48529 | 0 . 14489 | 0 .393153217 |
| IST1 | -0 . 15585461 | 8 .882120469 | -1 .4852 | 0 . 14493 | 0 .393153217 |
| MBD2 | 0 . 124932942 | 8 .972095832 | 1 .48512 | 0 . 14494 | 0 .393153217 |
| GTPBP6 | -0 . 195744008 | 8 . 153617141 | -1 .4851 | 0 . 14494 | 0 .393153217 |
| GAS7 | 0 .306765628 | 1 1 .35315014 | 1 .485 | 0 . 14497 | 0 .393153217 |
| STC1 | 0 .227625428 | 7 .345500023 | 1 .48483 | 0 . 14502 | 0 .393181488 |
| FLNA | 0 .245829957 | 1 1 .7848274 | 1 .48472 | 0 . 14505 | 0 .393181488 |
| HGD | -0 .258157839 | 5 .996673386 | -1 .4843 | 0 . 14515 | 0 .393391821 |
| MMS19 | -0 . 183576935 | 8 .762603826 | -1 .4841 | 0 . 14522 | 0 .39347448 |
| MOSPD3 | 0 .230226269 | 7 .609065002 | 1 .48387 | 0 . 14527 | 0 .39353604 |
| RBBP7 | -0 . 197227034 | 9 .413146685 | -1 .4828 | 0 . 14555 | 0 .394130519 |
| ARFGEF1 | 0 .206540968 | 8 .901623948 | 1 .48271 | 0 . 14558 | 0 .394130519 |
| IL4R | 0 .275621894 | 8 .043603598 | 1 .48268 | 0 . 14558 | 0 .394130519 |
| SSR1 | 0 . 150943534 | 10 .89765398 | 1 .48252 | 0 . 14563 | 0 .394162675 |
| 7-Mar | -0 .229961391 | 8 .682689409 | -1 .4823 | 0 . 1457 | 0 .394200626 |
| OPLAH | -0 .263124364 | 6 .011 184185 | -1 .4822 | 0 . 14572 | 0 .394200626 |
| TWF1 | 0 .255396031 | 8 .47817763 | 1 .482 | 0 . 14576 | 0 .394200626 |
| RFC3 | 0 . 194232117 | 7 .531829652 | 1 .48197 | 0 . 14577 | 0 .394200626 |
| ZBTB43 | -0 . 166963326 | 8 .53699548 | -1 .4819 | 0 . 1458 | 0 .394200626 |
| CMKLR1 | 0 . 194556799 | 7 .502720135 | 1 .48167 | 0 . 14585 | 0 .394260776 |
| BARX1 | 0 .264355515 | 4 .692761143 | 1 .4811 1 | 0 . 146 | 0 .394578698 |
| GSK3A | 0 . 170649766 | 10 . 12218961 | 1 .4806 | 0 . 14614 | 0 .394753332 |
| RNF167 | 0 . 14110623 | 8 .456637234 | 1 .48055 | 0 . 14615 | 0 .394753332 |

| PTH1R | -0 . 196492465 | 5 .635928503 | -1 .4805 | 0 . 14616 | 0 .394753332 |
| --- | --- | --- | --- | --- | --- |
| ORC6 | -0 .295578354 | 8 .22639186 | -1 .4801 | 0 . 14627 | 0 .394968387 |
| CYP7B1 | 0 .352283767 | 5 .479217577 | 1 .47976 | 0 . 14636 | 0 .395047899 |
| ERVMER34-1 | 0 .253565555 | 6 .697533618 | 1 .47975 | 0 . 14636 | 0 .395047899 |
| TNFAIP6 | 0 .21236418 | 6 .719159529 | 1 .4794 | 0 . 14646 | 0 .395209229 |
| PPM1B | -0 . 158990904 | 8 .097332841 | -1 .4792 | 0 . 1465 | 0 .3952511 19 |
| EXOC1 | -0 .253066344 | 7 .640912211 | -1 .4789 | 0 . 14658 | 0 .39530008 |
| SMPDL3A | -0 .350756038 | 7 .261661848 | -1 .4789 | 0 . 14658 | 0 .39530008 |
| ELMO1 | 0 .279659998 | 6 .53439729 | 1 .47862 | 0 . 14666 | 0 .395404615 |
| EFHC2 | -0 . 195574681 | 6 .887224247 | -1 .4785 | 0 . 14669 | 0 .395404615 |
| MPPE1 | -0 . 163155479 | 8 .785507113 | -1 .4782 | 0 . 14677 | 0 .395551371 |
| ADNP | -0 .230751748 | 8 . 15343767 | -1 .478 | 0 . 14682 | 0 .395587151 |
| RPL23AP53 | -0 .319844719 | 4 .285097744 | -1 .4775 | 0 . 14695 | 0 .395820668 |
| DCAF17 | -0 .216977224 | 4 .561462897 | -1 .4775 | 0 . 14697 | 0 .395820668 |
| SNPH | 0 .353907081 | 5 . 158360709 | 1 .47734 | 0 . 147 | 0 .395839649 |
| LOC101929272 | 0 .417638115 | 4 .90414647 | 1 .47699 | 0 . 1471 | 0 .395940585 |
| ETNK2 | 0 .242406481 | 6 .697270507 | 1 .47696 | 0 . 14711 | 0 .395940585 |
| NPTX2 | -0 .408589336 | 6 .961088343 | -1 .4767 | 0 . 14718 | 0 .396055673 |
| PRPS2 | -0 .274704684 | 7 .390589729 | -1 .4763 | 0 . 14728 | 0 .396227607 |
| PARP1 | 0 . 167158266 | 8 .770699504 | 1 .47621 | 0 . 14731 | 0 .396227607 |
| PAFAH1B3 | 0 .247916515 | 7 .71859323 | 1 .47572 | 0 . 14744 | 0 .396494888 |
| KIAA0895 | -0 .366978753 | 3 .685591926 | -1 .4754 | 0 . 14753 | 0 .396656262 |
| VPS45 | 0 . 181188819 | 7 .370365533 | 1 .47521 | 0 . 14757 | 0 .396690787 |
| KLHL3 | -0 .323736028 | 5 .524447517 | -1 .475 | 0 . 14763 | 0 .396703381 |
| IKZF1 | 0 . 179246846 | 8 .821185475 | 1 .47496 | 0 . 14764 | 0 .396703381 |
| PLA2G2A | 0 .46791062 | 7 .344010874 | 1 .47464 | 0 . 14773 | 0 .396847333 |
| CREG1 | -0 .333591684 | 9 .276802137 | -1 .4744 | 0 . 14778 | 0 .396882693 |
| WFDC8 | 0 .25288234 | 5 .344743475 | 1 .47431 | 0 . 14782 | 0 .396882693 |
| EAPP | -0 .203635487 | 7 . 1 10981292 | -1 .4742 | 0 . 14783 | 0 .396882693 |
| IGLL1 | 0 .478900429 | 4 .589647608 | 1 .47361 | 0 . 148 | 0 .397249694 |
| GLRA2 | -0 .407992336 | 5 .757318832 | -1 .4734 | 0 . 14805 | 0 .39728387 |
| RP1-217P22 .2 | 0 .510591895 | 3 .44652312 | 1 .47312 | 0 . 14813 | 0 .397432632 |
| CSRP1 | -0 .285788943 | 10 .34885149 | -1 .4727 | 0 . 14826 | 0 .397627807 |
| GPS2 | 0 . 17305142 | 8 .583766888 | 1 .47261 | 0 . 14827 | 0 .397627807 |
| SPCS3 | 0 . 174625234 | 9 .345084591 | 1 .47239 | 0 . 14833 | 0 .397704013 |
| SIX1 | 0 .39343131 | 4 .491710558 | 1 .47213 | 0 . 1484 | 0 .397809136 |
| STAG3L3 | -0 .367293893 | 5 .46790125 | -1 .4715 | 0 . 14858 | 0 .398080718 |
| KIAA0319L | 0 .344204422 | 3 .694469486 | 1 .47143 | 0 . 14859 | 0 .398080718 |
| LRRC36 | 0 .276766583 | 7 . 104553485 | 1 .4714 | 0 . 1486 | 0 .398080718 |
| THSD4 | -0 .282627539 | 5 . 161217558 | -1 .4707 | 0 . 14878 | 0 .398485567 |
| SERPINA5 | -0 .334230055 | 5 .001903604 | -1 .4705 | 0 . 14884 | 0 .398527958 |
| KANSL2 | -0 .223595018 | 7 .015751844 | -1 .4703 | 0 . 14888 | 0 .398527958 |
| BCL7A | -0 .241185288 | 8 .328069701 | -1 .4702 | 0 . 14893 | 0 .398527958 |
| RPL41 | -0 . 198554063 | 12 .7807204 | -1 .4701 | 0 . 14896 | 0 .398527958 |
| ZSCAN5A | -0 .235586647 | 5 .940175277 | -1 .47 | 0 . 14897 | 0 .398527958 |
| CTRC | -0 .24570706 | 7 .00120029 | -1 .47 | 0 . 14899 | 0 .398527958 |
| TCF20 | -0 . 156296103 | 8 .789156182 | -1 .4696 | 0 . 14909 | 0 .398725803 |
| GPR35 | 0 .234286513 | 5 .560141098 | 1 .46891 | 0 . 14927 | 0 .39911 1866 |
| CBFA2T3 | -0 .211817649 | 6 . 132708658 | -1 .4686 | 0 . 14935 | 0 .399243636 |
| KDM4A | -0 . 137062992 | 8 .515249452 | -1 .468 | 0 . 14952 | 0 .399599182 |
| POP7 | 0 . 171203102 | 8 .649369128 | 1 .46782 | 0 . 14956 | 0 .39964141 |
| ERCC6L | 0 .329611 163 | 3 .855809672 | 1 .4673 | 0 . 1497 | 0 .399895689 |
| CD22 | 0 .217666348 | 8 .692447481 | 1 .4672 | 0 . 14973 | 0 .399895689 |
| SUPT7L | 0 . 135904155 | 7 .733628963 | 1 .46692 | 0 . 14981 | 0 .399895689 |
| ABCA2 | 0 . 153993673 | 7 .553911878 | 1 .46689 | 0 . 14982 | 0 .399895689 |
| LIN28A | -0 .348006838 | 4 .634208311 | -1 .4669 | 0 . 14982 | 0 .399895689 |

| KAT2B | -0 .524405282 | 7 .475883389 | -1 .4661 | 0 . 15002 | 0 .400359334 |
| --- | --- | --- | --- | --- | --- |
| ASB1 | -0 . 130769757 | 8 . 158386172 | -1 .4658 | 0 . 1501 | 0 .400389403 |
| PTPLA | -0 .323565062 | 7 .355546081 | -1 .4657 | 0 . 15013 | 0 .400389403 |
| USP22 | 0 . 171359136 | 10 .24278286 | 1 .46568 | 0 . 15014 | 0 .400389403 |
| SLC17A9 | 0 .310506979 | 6 .205986792 | 1 .46561 | 0 . 15016 | 0 .400389403 |
| TEX11 | -0 .206913655 | 5 .723202523 | -1 .4654 | 0 . 15023 | 0 .400488945 |
| TRIB3 | 0 .265785795 | 7 .373495557 | 1 .46477 | 0 . 15039 | 0 .400795375 |
| FKBP3 | -0 .224536237 | 7 .93580341 | -1 .4647 | 0 . 15041 | 0 .400795375 |
| PDK4 | -0 .340244278 | 4 .284406614 | -1 .4644 | 0 . 15049 | 0 .400913687 |
| RBFA | -0 . 159267561 | 7 . 1 13459222 | -1 .464 | 0 . 1506 | 0 .401099086 |
| AKT1 | 0 .205293313 | 9 .366149979 | 1 .46393 | 0 . 15062 | 0 .401099086 |
| IARS | 0 .233752322 | 8 .7886893 | 1 .46372 | 0 . 15067 | 0 .401160045 |
| ZNF384 | -0 .227680042 | 8 .960048407 | -1 .4634 | 0 . 15076 | 0 .401301161 |
| GMCL1 | -0 .215539991 | 5 .64991996 | -1 .4627 | 0 . 15096 | 0 .401736316 |
| TAOK1 | -0 .402256896 | 5 .92957574 | -1 .4622 | 0 . 1511 | 0 .402042855 |
| SPATA7 | -0 .602924287 | 5 .584477989 | -1 .462 | 0 . 15114 | 0 .402044872 |
| PPL | -0 .605398567 | 9 .07816965 | -1 .4618 | 0 . 15119 | 0 .40211 1487 |
| EMC10 | 0 .417229673 | 3 . 151853135 | 1 .46147 | 0 . 15129 | 0 .402272086 |
| SKIV2L2 | -0 .239517342 | 7 .683674449 | -1 .4614 | 0 . 15132 | 0 .402272086 |
| ADORA3 | 0 .265369792 | 6 .918118699 | 1 .46079 | 0 . 15147 | 0 .402604558 |
| AMBRA1 | 0 .234043226 | 7 .938341199 | 1 .46064 | 0 . 15152 | 0 .402629619 |
| TRDV3 | -0 .303207188 | 5 .481640053 | -1 .4604 | 0 . 15159 | 0 .40273654 |
| PLK2 | 0 .375878136 | 7 .366937585 | 1 .45994 | 0 . 15171 | 0 .402956692 |
| HOXC10 | -0 .332830471 | 7 .547648428 | -1 .4598 | 0 . 15173 | 0 .402956692 |
| CDC42EP4 | -0 .211705434 | 9 . 141495514 | -1 .4591 | 0 . 15193 | 0 .403308229 |
| COL18A1 | 0 .251958641 | 10 . 10668982 | 1 .45908 | 0 . 15194 | 0 .403308229 |
| TRIOBP | -0 .230745075 | 9 .868961975 | -1 .459 | 0 . 15196 | 0 .403308229 |
| DNMT3A | 0 .482888742 | 4 . 122943548 | 1 .45856 | 0 . 15209 | 0 .403386866 |
| TOB2 | -0 .30536839 | 8 .416031318 | -1 .4584 | 0 . 15212 | 0 .403386866 |
| EIF2S2 | 0 .223635406 | 9 . 18803516 | 1 .45839 | 0 . 15213 | 0 .403386866 |
| DCAF16 | 0 .2011 17138 | 7 .40142061 | 1 .45834 | 0 . 15215 | 0 .403386866 |
| RALGAPB | 0 . 147196733 | 7 .998596647 | 1 .45825 | 0 . 15217 | 0 .403386866 |
| MAP3K12 | 0 .290555334 | 7 . 153014991 | 1 .45819 | 0 . 15219 | 0 .403386866 |
| IPO4 | 0 .20500549 | 7 .681556083 | 1 .45795 | 0 . 15225 | 0 .403476646 |
| MCAM | 0 .504002106 | 8 .974161262 | 1 .45776 | 0 . 1523 | 0 .403495713 |
| MYO5A | 0 .335816657 | 8 .466565719 | 1 .45762 | 0 . 15234 | 0 .403495713 |
| DMTN | 0 .34734022 | 5 .914561993 | 1 .45753 | 0 . 15237 | 0 .403495713 |
| PAPSS2 | 0 . 193691366 | 8 .526672578 | 1 .45735 | 0 . 15242 | 0 .403495713 |
| CEP70 | -0 .231454153 | 5 .665482899 | -1 .4573 | 0 . 15242 | 0 .403495713 |
| BCL2L13 | 0 .20399811 | 7 .645112355 | 1 .45711 | 0 . 15248 | 0 .403579787 |
| MDM4 | 0 .767262812 | 3 .93511 1318 | 1 .45685 | 0 . 15255 | 0 .403679225 |
| C16orf59 | 0 .290402757 | 4 .88394857 | 1 .45662 | 0 . 15262 | 0 .403760755 |
| IL10RB | -0 . 199837109 | 7 . 1 1 139071 | -1 .4561 | 0 . 15277 | 0 .40407185 |
| FAM49B | 0 .258767746 | 7 .762929901 | 1 .45553 | 0 . 15292 | 0 .404277095 |
| SPO11 | 0 .430755706 | 4 .222826147 | 1 .45547 | 0 . 15294 | 0 .404277095 |
| CAD | 0 . 179434439 | 7 .587513838 | 1 .45545 | 0 . 15294 | 0 .404277095 |
| RAB21 | -0 .214068871 | 6 .743337388 | -1 .4553 | 0 . 15298 | 0 .40428054 |
| CRYBB2P1 | 0 . 19373709 | 5 .611260312 | 1 .45521 | 0 . 15301 | 0 .40428054 |
| ETNPPL | -0 .276289704 | 4 .64331596 | -1 .4548 | 0 . 15312 | 0 .404483291 |
| MAGEB3 | -0 .377389936 | 3 . 199994669 | -1 .4547 | 0 . 15315 | 0 .404483291 |
| UBE2M | 0 .283615486 | 7 .592845918 | 1 .45389 | 0 . 15337 | 0 .404984486 |
| WT1 | 0 .235627165 | 6 . 186385904 | 1 .45333 | 0 . 15352 | 0 .405306858 |
| SIX6 | -0 .238116163 | 5 .360697494 | -1 .4528 | 0 . 15368 | 0 .40563963 |
| MIS18BP1 | 0 .343830725 | 4 .944997345 | 1 .45244 | 0 . 15377 | 0 .405663016 |
| FUT4 | -0 .218644393 | 6 . 1 13943594 | -1 .4524 | 0 . 15378 | 0 .405663016 |
| G3BP2 | -0 .202741031 | 8 .541481588 | -1 .4524 | 0 . 15379 | 0 .405663016 |

| NARF | 0 . 179928769 | 7 .995499029 | 1 .45218 | 0 . 15384 | 0 .405663016 |
| --- | --- | --- | --- | --- | --- |
| AKAP8L | 0 . 193262055 | 7 .988868486 | 1 .45214 | 0 . 15385 | 0 .405663016 |
| SPTBN1 | -0 .229152911 | 1 1 . 12445524 | -1 .4517 | 0 . 15399 | 0 .405925801 |
| LOC102723479 | 0 .3326197 | 4 .900361381 | 1 .45148 | 0 . 15404 | 0 .405938442 |
| CD6 | 0 .290219835 | 8 .769004719 | 1 .45141 | 0 . 15405 | 0 .405938442 |
| ASH2L | -0 . 187192743 | 8 .280123406 | -1 .4511 | 0 . 15413 | 0 .406052242 |
| MYH9 | 0 .203549347 | 10 .82867695 | 1 .45086 | 0 . 15421 | 0 .406169679 |
| ZNF266 | -0 . 178603015 | 7 .402889592 | -1 .4502 | 0 . 1544 | 0 .406581587 |
| MRPL17 | 0 .243779037 | 7 .845624124 | 1 .44979 | 0 . 1545 | 0 .40673019 |
| EVI2A | 0 .40365642 | 6 .32339512 | 1 .44958 | 0 . 15456 | 0 .40673019 |
| CCL23 | 0 .537787797 | 6 .259286983 | 1 .44947 | 0 . 15459 | 0 .40673019 |
| FGA | -0 .261386713 | 6 . 166369363 | -1 .4494 | 0 . 1546 | 0 .40673019 |
| SLC5A12 | 0 .344204429 | 2 .567543016 | 1 .44938 | 0 . 15462 | 0 .40673019 |
| NOD2 | 0 .345951451 | 6 .016088168 | 1 .44928 | 0 . 15465 | 0 .40673019 |
| INSRR | 0 .258763952 | 5 .322908181 | 1 .44876 | 0 . 15479 | 0 .406908048 |
| DAZAP2 | -0 .200471011 | 1 1 .91848642 | -1 .4488 | 0 . 15479 | 0 .406908048 |
| SIPA1L1 | 0 . 149123747 | 7 .760830464 | 1 .44868 | 0 . 15481 | 0 .406908048 |
| MNX1 | 0 .453496957 | 4 .871457486 | 1 .4484 | 0 . 15489 | 0 .407029657 |
| TCF7 | -0 .259776209 | 8 .743564931 | -1 .4476 | 0 . 15512 | 0 .407559689 |
| CD200 | 0 .341624782 | 7 .67976235 | 1 .44703 | 0 . 15527 | 0 .407862187 |
| KIAA1644 | 0 .233386801 | 6 .255556807 | 1 .44676 | 0 . 15535 | 0 .407976695 |
| SARS | -0 . 163421601 | 9 .42116597 | -1 .4465 | 0 . 15541 | 0 .408003159 |
| PDE6H | 0 .37745122 | 4 .358342704 | 1 .44649 | 0 . 15542 | 0 .408003159 |
| RNF113A | -0 . 171348927 | 7 .568517716 | -1 .4463 | 0 . 15547 | 0 .408028101 |
| RHOQ | 0 .286901365 | 1 1 .03946172 | 1 .44601 | 0 . 15556 | 0 .408179473 |
| LOC100288570 | 0 .26264817 | 5 .602520684 | 1 .44523 | 0 . 15577 | 0 .408667485 |
| TAF1 | 0 .212597151 | 6 .803920353 | 1 .44494 | 0 . 15586 | 0 .408733323 |
| PRIM1 | 0 . 177227704 | 6 .738543226 | 1 .44491 | 0 . 15586 | 0 .408733323 |
| ADAM10 | 0 .24992966 | 8 .765785189 | 1 .44433 | 0 . 15603 | 0 .408997041 |
| HSD17B6 | -0 .220551769 | 4 .94916814 | -1 .4443 | 0 . 15603 | 0 .408997041 |
| RWDD1 | -0 . 179582818 | 8 .453979145 | -1 .4441 | 0 . 1561 | 0 .409088428 |
| RP1-130G2 . 1 | -0 .349028868 | 4 .764565113 | -1 .4438 | 0 . 15616 | 0 .409171301 |
| ZNF106 | -0 .525934506 | 8 .834465625 | -1 .4431 | 0 . 15636 | 0 .40951799 |
| FBXL18 | -0 .232188607 | 7 .018978804 | -1 .4431 | 0 . 15636 | 0 .40951799 |
| CCS | 0 .214763758 | 6 .935339297 | 1 .44291 | 0 . 15642 | 0 .409597535 |
| TCF12 | -0 .285133641 | 8 .204715563 | -1 .4428 | 0 . 15646 | 0 .409598897 |
| PPA1 | -0 .227653496 | 9 .561109406 | -1 .4423 | 0 . 15661 | 0 .409871861 |
| HPS1 | 0 . 190814417 | 8 .984945014 | 1 .44212 | 0 . 15665 | 0 .409871861 |
| FN3KRP | 0 . 198332255 | 8 .346400039 | 1 .44207 | 0 . 15666 | 0 .409871861 |
| PRMT1 | 0 . 184033485 | 9 .2055028 | 1 .44112 | 0 . 15693 | 0 .410489511 |
| CROCCP2 | 0 .31554951 | 6 .640763093 | 1 .44033 | 0 . 15715 | 0 .410986122 |
| CLDN18 | 0 .203810999 | 7 .531905764 | 1 .43878 | 0 . 15759 | 0 .41204208 |
| BTBD7 | -0 .428949421 | 5 .667957831 | -1 .4382 | 0 . 15776 | 0 .412342159 |
| PDZD8 | -0 .338365435 | 5 . 168171371 | -1 .4381 | 0 . 15777 | 0 .412342159 |
| HMGB3 | 0 .227881946 | 6 .730018796 | 1 .438 | 0 . 15781 | 0 .412342159 |
| PROM1 | -0 .295112462 | 4 .756362682 | -1 .4378 | 0 . 15786 | 0 .412342159 |
| GLRX3 | 0 . 178855353 | 9 .43776786 | 1 .43779 | 0 . 15787 | 0 .412342159 |
| NFATC3 | -0 . 175373574 | 7 .926621368 | -1 .4375 | 0 . 15795 | 0 .41247433 |
| RP11-403P17 .4 | -0 .391124823 | 6 .917225529 | -1 .4374 | 0 . 15798 | 0 .41247433 |
| LINC00675 | 0 .30981865 | 4 .857708364 | 1 .43699 | 0 . 15809 | 0 .412674963 |
| DYNC1LI2 | -0 .231634341 | 8 .973234548 | -1 .4367 | 0 . 15817 | 0 .412789554 |
| HDAC3 | -0 . 179472118 | 8 .219391413 | -1 .4363 | 0 . 15829 | 0 .413006713 |
| C3orf14 | 0 .226918929 | 7 .897985438 | 1 .43619 | 0 . 15832 | 0 .413006713 |
| NAA40 | 0 . 145735658 | 7 .681648141 | 1 .4359 | 0 . 1584 | 0 .413121822 |
| MPHOSPH9 | 0 .333370862 | 5 .054206053 | 1 .43562 | 0 . 15848 | 0 .413121822 |
| CLIC5 | -0 .226176774 | 7 . 179961037 | -1 .4356 | 0 . 15849 | 0 .413121822 |

| SLC25A3 | -0 . 147150546 | 1 1 . 18919375 | -1 .4353 | 0 . 15857 | 0 .413121822 |
| --- | --- | --- | --- | --- | --- |
| CDC25C | -0 . 184099758 | 6 .959224116 | -1 .4352 | 0 . 15858 | 0 .413121822 |
| GPN3 | -0 .321060387 | 6 .645570505 | -1 .4351 | 0 . 15862 | 0 .413121822 |
| STAM2 | 0 .22410585 | 6 .832015029 | 1 .43501 | 0 . 15865 | 0 .413121822 |
| C14orf159 | -0 .216367322 | 7 .098924935 | -1 .435 | 0 . 15865 | 0 .413121822 |
| CLIC1 | 0 . 196234422 | 10 . 1847575 | 1 .43498 | 0 . 15866 | 0 .413121822 |
| MAPK8IP2 | -0 . 199199366 | 6 .594651531 | -1 .4345 | 0 . 15878 | 0 .413316362 |
| SNAI2 | -0 .376408571 | 8 .750653684 | -1 .4344 | 0 . 15882 | 0 .413316362 |
| NEU3 | 0 .235114709 | 7 .074750045 | 1 .43437 | 0 . 15883 | 0 .413316362 |
| PPIP5K1 | -0 .335453806 | 5 .252769367 | -1 .4341 | 0 . 1589 | 0 .413410536 |
| PPIL6 | 0 .502730942 | 4 .260062557 | 1 .43399 | 0 . 15894 | 0 .413424662 |
| PEX6 | 0 .243419209 | 7 . 175546674 | 1 .43151 | 0 . 15965 | 0 .415176342 |
| OPRK1 | 0 .216898573 | 6 .893675317 | 1 .43129 | 0 . 15971 | 0 .415253591 |
| NOL7 | 0 .239958765 | 9 .9305453 | 1 .43062 | 0 . 1599 | 0 .41565944 |
| LOC202181 | 0 .439835479 | 3 .402802637 | 1 .42993 | 0 . 1601 | 0 .416088563 |
| FAM32A | 0 . 14100739 | 8 .421282232 | 1 .42969 | 0 . 16017 | 0 .416183807 |
| NSFL1C | -0 . 162422168 | 9 .571454539 | -1 .4288 | 0 . 16043 | 0 .416743181 |
| PCDHGA9 | 0 .315427213 | 3 .870596693 | 1 .42839 | 0 . 16053 | 0 .416743181 |
| PLEKHO1 | 0 .305421497 | 6 .747485911 | 1 .42839 | 0 . 16054 | 0 .416743181 |
| SLC26A10 | 0 .481666608 | 6 . 170476166 | 1 .42823 | 0 . 16058 | 0 .416743181 |
| AIM1 | -0 .489866981 | 8 .277373865 | -1 .4282 | 0 . 1606 | 0 .416743181 |
| HSPA9 | 0 .236029205 | 9 .839976486 | 1 .42811 | 0 . 16062 | 0 .416743181 |
| LINC01260 | 0 .218300349 | 6 .637585394 | 1 .42798 | 0 . 16065 | 0 .416743181 |
| ZNF26 | -0 .257958751 | 5 .955992944 | -1 .4279 | 0 . 16069 | 0 .416743181 |
| PHTF1 | -0 .24776228 | 8 .393694785 | -1 .4278 | 0 . 16069 | 0 .416743181 |
| PCDHB17 | 0 .279592049 | 3 .746424144 | 1 .42768 | 0 . 16074 | 0 .416743181 |
| ANAPC5 | 0 . 155530974 | 10 .800512 | 1 .42765 | 0 . 16075 | 0 .416743181 |
| NDUFAF1 | 0 .217759274 | 6 .898419823 | 1 .42748 | 0 . 16079 | 0 .416784707 |
| P2RY14 | -0 .247120124 | 6 .900860978 | -1 .4272 | 0 . 16087 | 0 .416834001 |
| CADPS | 0 .370351494 | 5 . 148483888 | 1 .42684 | 0 . 16098 | 0 .416834001 |
| KCNQ1 | -0 .367563629 | 5 .684659842 | -1 .4268 | 0 . 161 | 0 .416834001 |
| CCL19 | 0 .676917203 | 9 .419457253 | 1 .42671 | 0 . 16102 | 0 .416834001 |
| UBE4A | -0 .236731338 | 8 .30288075 | -1 .4266 | 0 . 16104 | 0 .416834001 |
| DNAJC10 | 0 .315916508 | 6 .659962932 | 1 .42656 | 0 . 16106 | 0 .416834001 |
| TM4SF4 | -0 .323281336 | 5 .345328449 | -1 .4266 | 0 . 16106 | 0 .416834001 |
| KRT36 | 0 . 128885894 | 1 .646419107 | 1 .42634 | 0 . 16112 | 0 .416834001 |
| ASAP3 | -0 .225559356 | 8 .285144072 | -1 .4263 | 0 . 16114 | 0 .416834001 |
| PREPL | -0 .266019825 | 9 .070889057 | -1 .4263 | 0 . 16115 | 0 .416834001 |
| RPL27A | -0 .441351172 | 8 .833213587 | -1 .426 | 0 . 16122 | 0 .416939302 |
| SPIN1 | -0 .314059291 | 6 .970193411 | -1 .4253 | 0 . 16143 | 0 .417405878 |
| CDX4 | 0 .315069236 | 5 .303524743 | 1 .42502 | 0 . 1615 | 0 .41748324 |
| USP39 | 0 . 193956259 | 6 .842605892 | 1 .42492 | 0 . 16153 | 0 .41748324 |
| N4BP2L1 | -0 . 181878832 | 8 .734089305 | -1 .4245 | 0 . 16164 | 0 .417686431 |
| R3HCC1 | -0 . 155604648 | 9 .323907748 | -1 .4235 | 0 . 16193 | 0 .418333294 |
| PRSS50 | -0 .295474346 | 4 .978875309 | -1 .4232 | 0 . 16204 | 0 .418541176 |
| EPB41L4A | -0 .283631593 | 5 .444376898 | -1 .4229 | 0 . 16211 | 0 .418570652 |
| ATP2B1 | -0 .249239033 | 8 .317182448 | -1 .4229 | 0 . 16212 | 0 .418570652 |
| RHO | 0 . 183343052 | 7 .063963326 | 1 .42258 | 0 . 1622 | 0 .418619286 |
| KIAA0513 | 0 .297089503 | 7 .232037274 | 1 .42255 | 0 . 16221 | 0 .418619286 |
| SEC24C | 0 . 150278394 | 8 . 163530478 | 1 .42247 | 0 . 16224 | 0 .418619286 |
| IRF5 | 0 .21181204 | 7 .59413401 | 1 .42225 | 0 . 1623 | 0 .418619286 |
| SOD1 | -0 .213979563 | 10 .35327738 | -1 .4222 | 0 . 1623 | 0 .418619286 |
| GAMT | -0 .326637445 | 6 . 140566845 | -1 .4221 | 0 . 16235 | 0 .418657764 |
| FSTL4 | -0 .300473652 | 6 .765507039 | -1 .4217 | 0 . 16247 | 0 .418842379 |
| RAB11 FIP3 | -0 . 19711 1715 | 7 .462486723 | -1 .4215 | 0 . 16251 | 0 .418842379 |
| PURG | -0 .306865368 | 4 .022137425 | -1 .4215 | 0 . 16252 | 0 .418842379 |

| CRYZ | -0 .356837648 | 6 .883200809 | -1 .4213 | 0 . 16258 | 0 .418906261 |
| --- | --- | --- | --- | --- | --- |
| CSNK1E | 0 .444684343 | 5 .473504981 | 1 .42091 | 0 . 16269 | 0 .419009254 |
| POMT1 | -0 .221313022 | 6 .962350817 | -1 .4209 | 0 . 16269 | 0 .419009254 |
| TP53I11 | 0 .201777089 | 8 .262828874 | 1 .42034 | 0 . 16285 | 0 .419313272 |
| KRT8 | -0 .39546601 | 6 .754607633 | -1 .4203 | 0 . 16287 | 0 .419313272 |
| CLDN5 | -0 .525240732 | 6 .355226841 | -1 .42 | 0 . 16296 | 0 .419455646 |
| METTL5 | 0 . 138168171 | 8 .371158012 | 1 .41975 | 0 . 16302 | 0 .419525748 |
| EIF2AK2 | 0 .223784969 | 9 .471120927 | 1 .41952 | 0 . 16309 | 0 .419609446 |
| CCDC94 | 0 . 177983717 | 6 .843313073 | 1 .41932 | 0 . 16315 | 0 .419671132 |
| HIPK1 | -0 . 198401057 | 8 .569785508 | -1 .4182 | 0 . 16346 | 0 .4203966 |
| TRO | 0 . 178532253 | 7 .243159448 | 1 .41752 | 0 . 16367 | 0 .42073801 |
| IL32 | 0 .744874094 | 8 .015762864 | 1 .41748 | 0 . 16368 | 0 .42073801 |
| CDR1 | 0 .416706828 | 3 .278230411 | 1 .41743 | 0 . 16369 | 0 .42073801 |
| FOXN1 | 0 .325540912 | 2 .725370859 | 1 .4173 | 0 . 16373 | 0 .42074484 |
| MAPK8 | -0 .25989432 | 6 .762057794 | -1 .4172 | 0 . 16377 | 0 .420764505 |
| TGFBI | -0 .330470185 | 9 .854194053 | -1 .4167 | 0 . 16391 | 0 .421030812 |
| DGKZ | 0 .294576122 | 8 .467472822 | 1 .41641 | 0 . 16399 | 0 .421152606 |
| TMEM115 | 0 . 18252766 | 8 . 1 19025336 | 1 .41628 | 0 . 16403 | 0 .421163182 |
| AMPD2 | 0 . 160991998 | 8 .008763021 | 1 .41617 | 0 . 16406 | 0 .421163182 |
| HNRNPA1 | -0 .208520699 | 9 .823744614 | -1 .4159 | 0 . 16413 | 0 .4211879 |
| NIF3L1 | -0 . 192198532 | 7 .340452076 | -1 .4157 | 0 . 16419 | 0 .4211879 |
| H2AFY2 | -0 .441997393 | 6 .269322374 | -1 .4157 | 0 . 1642 | 0 .4211879 |
| LILRA1 | -0 .242893112 | 5 .6000606 | -1 .4157 | 0 . 16421 | 0 .4211879 |
| RFWD3 | 0 .317259909 | 5 .599745463 | 1 .41516 | 0 . 16436 | 0 .421485613 |
| C14orf132 | -0 .41986955 | 7 . 179742294 | -1 .4148 | 0 . 16446 | 0 .421577771 |
| RAB8A | 0 . 172198599 | 9 . 196816271 | 1 .4148 | 0 . 16446 | 0 .421577771 |
| ZDHHC14 | -0 .206282679 | 6 .91701421 | -1 .4144 | 0 . 16459 | 0 .421826933 |
| MCM3AP | 0 . 182597887 | 8 .765447137 | 1 .41408 | 0 . 16467 | 0 .421886712 |
| GORASP2 | 0 . 124918261 | 10 .45089313 | 1 .41405 | 0 . 16468 | 0 .421886712 |
| PDC | -0 .408023493 | 4 .325509711 | -1 .4135 | 0 . 16483 | 0 .422117223 |
| FCN3 | 0 .24888898 | 5 . 100468862 | 1 .4135 | 0 . 16484 | 0 .422117223 |
| MBTPS1 | -0 . 151915594 | 9 . 120925134 | -1 .4134 | 0 . 16487 | 0 .422117223 |
| CXCL11 | 0 .635046859 | 6 . 133708093 | 1 .41326 | 0 . 16491 | 0 .42213322 |
| C2orf72 | 0 .272123778 | 5 .876407493 | 1 .41252 | 0 . 16512 | 0 .422593621 |
| ANXA6 | -0 .247778997 | 8 .882257824 | -1 .4122 | 0 . 16521 | 0 .422715378 |
| FGF20 | -0 .338708482 | 4 .062926119 | -1 .4119 | 0 . 1653 | 0 .422834154 |
| SYNRG | -0 . 15523421 | 8 .966807281 | -1 .4118 | 0 . 16535 | 0 .422834154 |
| POU6F1 | -0 .211759613 | 6 .656495416 | -1 .4117 | 0 . 16535 | 0 .422834154 |
| DND1 | -0 .251091121 | 8 .071026322 | -1 .4116 | 0 . 16541 | 0 .422890332 |
| URM1 | 0 . 192165714 | 8 .501762123 | 1 .41078 | 0 . 16563 | 0 .423380622 |
| NRXN3 | 0 .338719328 | 5 .581779376 | 1 .40985 | 0 . 16591 | 0 .423886503 |
| BYSL | 0 . 193188105 | 7 .882901731 | 1 .40955 | 0 . 166 | 0 .423886503 |
| ATP5B | -0 . 162488474 | 10 .94139064 | -1 .4093 | 0 . 16607 | 0 .423886503 |
| CEBPA | -0 .610845446 | 7 .857156882 | -1 .4093 | 0 . 16607 | 0 .423886503 |
| RP11-560G2 .2 | -0 .293691485 | 5 .060538169 | -1 .4093 | 0 . 16608 | 0 .423886503 |
| CYP11A1 | 0 .338325875 | 4 .746410786 | 1 .40915 | 0 . 16611 | 0 .423886503 |
| CNDP2 | 0 . 190319821 | 9 .043224638 | 1 .40912 | 0 . 16612 | 0 .423886503 |
| ARID1A | -0 . 177968414 | 10 . 13583499 | -1 .4089 | 0 . 16619 | 0 .423886503 |
| PXMP4 | -0 . 160853823 | 8 .443508655 | -1 .4089 | 0 . 16619 | 0 .423886503 |
| POLR2B | 0 . 16706417 | 9 .093904245 | 1 .40889 | 0 . 16619 | 0 .423886503 |
| SPHK2 | -0 .247138416 | 8 .400435972 | -1 .4088 | 0 . 1662 | 0 .423886503 |
| EGR4 | 0 .23462089 | 7 .020389434 | 1 .4087 | 0 . 16625 | 0 .423907336 |
| NEK7 | -0 .368130875 | 7 .410994791 | -1 .4085 | 0 . 16631 | 0 .423998047 |
| PCDH12 | 0 .310647429 | 7 . 130039737 | 1 .40822 | 0 . 16639 | 0 .424094771 |
| TMCO3 | 0 .217905258 | 6 .298719769 | 1 .4077 | 0 . 16654 | 0 .424350422 |
| BTNL3 | 0 .20507762 | 7 .226739015 | 1 .40765 | 0 . 16655 | 0 .424350422 |

| VSNL1 | 0 .625717547 | 8 .326448636 | 1 .40706 | 0 . 16673 | 0 .424709546 |
| --- | --- | --- | --- | --- | --- |
| KLHL24 | -0 .208588706 | 8 .053425423 | -1 .4068 | 0 . 1668 | 0 .424815801 |
| TTC9 | -0 .259832743 | 6 . 109088075 | -1 .4067 | 0 . 16684 | 0 .424825198 |
| BTN1A1 | 0 .293739438 | 5 . 1 14946306 | 1 .40637 | 0 . 16693 | 0 .424879551 |
| HTR4 | 0 .223713296 | 7 .738085535 | 1 .40637 | 0 . 16693 | 0 .424879551 |
| TTC26 | 0 .280122504 | 3 .925697617 | 1 .40619 | 0 . 16699 | 0 .424931278 |
| C16orf3 | 0 .298914744 | 5 .223055114 | 1 .40566 | 0 . 16714 | 0 .425239161 |
| CTBP2 | -0 .214979239 | 1 1 . 12159891 | -1 .4053 | 0 . 16724 | 0 .425347734 |
| LILRB1 | 0 .326683293 | 7 .689668934 | 1 .40529 | 0 . 16725 | 0 .425347734 |
| MED15 | 0 .233099454 | 7 .518245268 | 1 .40499 | 0 . 16734 | 0 .425484134 |
| APPL2 | -0 .279184858 | 7 .233262234 | -1 .4048 | 0 . 16738 | 0 .425510665 |
| KCNAB2 | 0 .31915279 | 8 .648844013 | 1 .40453 | 0 . 16747 | 0 .425658013 |
| ATP1B3 | -0 .204979824 | 10 .59991232 | -1 .4041 | 0 . 1676 | 0 .42588562 |
| FOXD3 | 0 .359994647 | 4 .009030153 | 1 .4039 | 0 . 16766 | 0 .42596153 |
| CSRNP3 | -0 .306539602 | 4 . 181949683 | -1 .4036 | 0 . 16776 | 0 .4261332 |
| DRD3 | 0 .237563293 | 5 .718344652 | 1 .40335 | 0 . 16782 | 0 .426202784 |
| VSX1 | 0 .577065511 | 3 .210065376 | 1 .40248 | 0 . 16808 | 0 .426771718 |
| SMARCA4 | 0 . 179077083 | 1 1 .01145574 | 1 .40217 | 0 . 16818 | 0 .426835107 |
| TMEM19 | -0 .298470956 | 5 .024507077 | -1 .4022 | 0 . 16818 | 0 .426835107 |
| OSM | 0 .274900937 | 2 .272141008 | 1 .40175 | 0 . 1683 | 0 .42706278 |
| ZNF432 | 0 .297388408 | 6 .021109977 | 1 .40154 | 0 . 16836 | 0 .427129088 |
| SGSM2 | -0 . 192464503 | 9 .787574428 | -1 .4012 | 0 . 16847 | 0 .427227366 |
| WDR7 | -0 . 188589042 | 6 .958458019 | -1 .4012 | 0 . 16847 | 0 .427227366 |
| NOX3 | 0 .391941702 | 3 .566289243 | 1 .40083 | 0 . 16857 | 0 .42727799 |
| BLVRB | -0 .221578855 | 8 . 194133157 | -1 .4008 | 0 . 16858 | 0 .42727799 |
| C3AR1 | 0 .271260737 | 6 .409857885 | 1 .40074 | 0 . 1686 | 0 .42727799 |
| HTR2B | 0 .456018831 | 4 .20655723 | 1 .40061 | 0 . 16864 | 0 .42727799 |
| TIPRL | -0 .336189057 | 6 .886597048 | -1 .4005 | 0 . 16866 | 0 .42727799 |
| POU5F1B | -0 .411873704 | 5 .397486542 | -1 .4004 | 0 . 16871 | 0 .427335947 |
| RNF122 | 0 . 178683497 | 6 .89836149 | 1 .40023 | 0 . 16875 | 0 .427338173 |
| LENEP | -0 .527857572 | 4 .764412971 | -1 .3995 | 0 . 16896 | 0 .427752305 |
| TBCC | 0 .206363188 | 6 .942251246 | 1 .39945 | 0 . 16898 | 0 .427752305 |
| DEPDC5 | -0 .304430121 | 4 .360760827 | -1 .3993 | 0 . 16902 | 0 .427777108 |
| C11orf68 | -0 . 136660703 | 8 .770181516 | -1 .3985 | 0 . 16928 | 0 .4282653 |
| PHKG2 | 0 . 144273485 | 7 .518262165 | 1 .39841 | 0 . 16929 | 0 .4282653 |
| LOX | -0 .203448281 | 7 .839859206 | -1 .3983 | 0 . 16932 | 0 .4282653 |
| POT1 | -0 . 152218093 | 7 .079693538 | -1 .3982 | 0 . 16937 | 0 .428299734 |
| KAT2A | -0 . 199476893 | 7 .505998272 | -1 .3974 | 0 . 16958 | 0 .428730729 |
| FAT1 | -0 . 184223818 | 8 .758209171 | -1 .3972 | 0 . 16965 | 0 .428730729 |
| CD69 | 0 .454921326 | 5 .312173485 | 1 .39715 | 0 . 16967 | 0 .428730729 |
| ZNF180 | -0 . 163303907 | 5 .552140845 | -1 .3971 | 0 . 16967 | 0 .428730729 |
| ANXA10 | -0 .373260911 | 3 .951737772 | -1 .3969 | 0 . 16973 | 0 .428780082 |
| MEGF8 | 0 . 165147706 | 6 . 177794379 | 1 .39678 | 0 . 16978 | 0 .428820681 |
| TCF15 | 0 .295732045 | 4 .748861176 | 1 .39656 | 0 . 16985 | 0 .428903134 |
| VHL | -0 .429748902 | 3 .970181985 | -1 .3959 | 0 . 17004 | 0 .42931497 |
| PACSIN2 | -0 .246541428 | 9 .388708721 | -1 .3951 | 0 . 17028 | 0 .429834014 |
| FAM204A | -0 .4347784 | 5 .238549922 | -1 .395 | 0 . 17032 | 0 .429852012 |
| RP11-217B7 .2 | -0 .294309711 | 5 .742110735 | -1 .3948 | 0 . 17036 | 0 .429852607 |
| HADHA | -0 . 151191902 | 10 .83052795 | -1 .3943 | 0 . 17052 | 0 .430125771 |
| VKORC1 | 0 . 191191005 | 9 .420438077 | 1 .39425 | 0 . 17054 | 0 .430125771 |
| NACA2 | -0 .349238312 | 3 .499478914 | -1 .3927 | 0 . 171 | 0 .431199532 |
| KIAA1024 | 0 .380053341 | 4 .677685844 | 1 .39252 | 0 . 17105 | 0 .431199532 |
| MSH3 | -0 . 173208586 | 7 .065070311 | -1 .3924 | 0 . 17108 | 0 .431199532 |
| EOGT | -0 .469619979 | 5 .69240438 | -1 .3923 | 0 . 17112 | 0 .431199532 |
| FARS2 | -0 . 144065898 | 8 .098018593 | -1 .3923 | 0 . 17113 | 0 .431199532 |
| PKIG | -0 .291498106 | 8 .612263343 | -1 .3915 | 0 . 17136 | 0 .431673742 |

| BMP8B | 0 .217759301 | 5 .663981137 | 1 .39128 | 0 . 17143 | 0 .431768335 |
| --- | --- | --- | --- | --- | --- |
| DPM2 | 0 . 185229071 | 7 .29399372 | 1 .39065 | 0 . 17162 | 0 .432155196 |
| RXRB | -0 . 138958035 | 8 .355475173 | -1 .3905 | 0 . 17166 | 0 .432155196 |
| CLPX | 0 .255603909 | 6 .910338875 | 1 .39043 | 0 . 17168 | 0 .432155196 |
| GM2A | 0 . 191465285 | 1 1 . 1 1716294 | 1 .39029 | 0 . 17172 | 0 .43217081 |
| ALG6 | -0 . 181453676 | 7 .584681086 | -1 .39 | 0 . 17182 | 0 .432322781 |
| ZNF324 | -0 . 199353324 | 6 .401785985 | -1 .3895 | 0 . 17197 | 0 .432617789 |
| ADCYAP1R1 | -0 .534018845 | 5 .437072207 | -1 .3892 | 0 . 17207 | 0 .432779419 |
| PITPNA | 0 . 160705808 | 9 .919059242 | 1 .38882 | 0 . 17217 | 0 .432944886 |
| ST8SIA5 | 0 .276910034 | 5 .295898425 | 1 .38825 | 0 . 17234 | 0 .433288732 |
| VGLL4 | -0 .218547187 | 9 .550828768 | -1 .3874 | 0 . 17259 | 0 .433750146 |
| ABCC2 | 0 .246881646 | 6 . 134964961 | 1 .38738 | 0 . 17261 | 0 .433750146 |
| GRIP1 | -0 .430082021 | 4 .310401457 | -1 .3873 | 0 . 17263 | 0 .433750146 |
| SERPINA6 | -0 .410663118 | 4 . 182322011 | -1 .387 | 0 . 17271 | 0 .433782103 |
| DHX30 | 0 . 174743385 | 8 .830974716 | 1 .38703 | 0 . 17271 | 0 .433782103 |
| STOM | -0 .204778021 | 10 . 13805008 | -1 .3869 | 0 . 17275 | 0 .433784139 |
| MED9 | -0 . 1 12024517 | 7 . 167137975 | -1 .3864 | 0 . 17291 | 0 .434021457 |
| RBM12 | -0 . 194583467 | 7 .717794528 | -1 .3862 | 0 . 17295 | 0 .434021457 |
| KBTBD11 | -0 .309999899 | 5 .459028553 | -1 .3862 | 0 . 17297 | 0 .434021457 |
| PMAIP1 | -0 .420844561 | 7 .85252249 | -1 .386 | 0 . 17302 | 0 .434021457 |
| TTC27 | -0 . 140148098 | 6 .883311 186 | -1 .386 | 0 . 17304 | 0 .434021457 |
| VAPA | -0 .208762844 | 9 .700012757 | -1 .3859 | 0 . 17305 | 0 .434021457 |
| UBFD1 | 0 .271284728 | 6 .34036711 | 1 .38561 | 0 . 17314 | 0 .434164077 |
| TLL2 | 0 .248187642 | 6 .058921842 | 1 .3855 | 0 . 17317 | 0 .434164077 |
| PILRA | 0 . 174256137 | 7 .803527667 | 1 .38491 | 0 . 17336 | 0 .434440732 |
| TBX1 | 0 . 153823413 | 6 .273110081 | 1 .38485 | 0 . 17337 | 0 .434440732 |
| HECW1 | 0 .240182682 | 7 .918515538 | 1 .3848 | 0 . 17339 | 0 .434440732 |
| TIMELESS | 0 . 171042151 | 8 .389420622 | 1 .38467 | 0 . 17343 | 0 .434450234 |
| XPO1 | -0 .249277706 | 8 .776511334 | -1 .3844 | 0 . 17351 | 0 .434533581 |
| AHSP | 0 .332523163 | 6 .242373782 | 1 .38433 | 0 . 17353 | 0 .434533581 |
| C8G | 0 .672817431 | 4 .446762871 | 1 .38408 | 0 . 17361 | 0 .434638127 |
| PPCDC | 0 .252570355 | 5 .368632539 | 1 .38369 | 0 . 17372 | 0 .434829743 |
| RAD54B | 0 .220220963 | 5 .25898619 | 1 .38357 | 0 . 17376 | 0 .434829743 |
| PIN1P1 | 0 .245688855 | 6 .679700151 | 1 .38341 | 0 . 17381 | 0 .434829743 |
| ADAM20 | -0 . 181592997 | 6 .428199609 | -1 .3834 | 0 . 17382 | 0 .434829743 |
| MORC3 | -0 .316763828 | 6 .777937566 | -1 .3832 | 0 . 17388 | 0 .434895847 |
| LOC101927770 | 0 .21175936 | 5 .085536023 | 1 .38264 | 0 . 17404 | 0 .435216393 |
| RAB3D | -0 .318301242 | 5 .480987153 | -1 .3823 | 0 . 17415 | 0 .435382612 |
| SH2D3A | -0 .293932598 | 6 .935542501 | -1 .382 | 0 . 17423 | 0 .435453686 |
| TRIM28 | -0 .200246266 | 9 .996367199 | -1 .382 | 0 . 17424 | 0 .435453686 |
| CDON | 0 .479848648 | 2 .955395341 | 1 .38149 | 0 . 17439 | 0 .435744068 |
| ATAT1 | 0 . 187353571 | 7 .81485373 | 1 .3811 | 0 . 17451 | 0 .435913895 |
| MAP2K6 | 0 . 198510006 | 6 .877295024 | 1 .38104 | 0 . 17453 | 0 .435913895 |
| IKBKG | 0 . 177351606 | 8 .759972628 | 1 .38079 | 0 . 17461 | 0 .435943462 |
| PSCA | 0 .25994921 | 5 .48303725 | 1 .38077 | 0 . 17461 | 0 .435943462 |
| GRTP1 | -0 .221170968 | 4 .808596417 | -1 .3803 | 0 . 17475 | 0 .436185125 |
| GSTA1 | -0 .206844841 | 6 .9293311 15 | -1 .3795 | 0 . 17499 | 0 .436599487 |
| PLEKHB2 | 0 .236204878 | 8 .997164218 | 1 .37947 | 0 . 17501 | 0 .436599487 |
| THBS3 | -0 . 165874001 | 7 .633562984 | -1 .3795 | 0 . 17502 | 0 .436599487 |
| STK39 | -0 .221072305 | 6 .76222114 | -1 .3792 | 0 . 1751 | 0 .436719902 |
| GGT5 | 0 . 193076545 | 7 .341230985 | 1 .37854 | 0 . 1753 | 0 .437056284 |
| TBL1X | -0 .206685821 | 8 .80461919 | -1 .3785 | 0 . 1753 | 0 .437056284 |
| ZNF506 | 0 .278933351 | 4 .813917768 | 1 .37833 | 0 . 17536 | 0 .437083508 |
| NEUROD1 | 0 .437868019 | 3 .83992273 | 1 .37818 | 0 . 17541 | 0 .437083508 |
| RAG1 | -0 .317755729 | 4 .077644443 | -1 .3781 | 0 . 17542 | 0 .437083508 |
| GS1-164F24 . 1 | 0 .324928844 | 4 .416251352 | 1 .37798 | 0 . 17547 | 0 .437121007 |

| KLK12 0 .497043363 | 5 .812966713 | 1 .3776 | 0 . 17559 | 0 .437325473 |
| --- | --- | --- | --- | --- |
| H2AFJ -0 .302091962 | 5 . 1 1 1952543 | -1 .3774 | 0 . 17565 | 0 .437405341 |
| CAB39 -0 .250412817 | 7 .95755879 | -1 .3772 | 0 . 17572 | 0 .437478222 |
| KANSL3 -0 .366012928 | 3 .392359958 | -1 .377 | 0 . 17577 | 0 .437521995 |
| TUBB4A 0 .4308616 | 7 .979375396 | 1 .37689 | 0 . 1758 | 0 .437522441 |
| ZNF33B -0 . 198421511 | 5 .92814146 | -1 .3766 | 0 . 17591 | 0 .437646762 |
| SLC26A4 -0 .308551241 | 5 .416799015 | -1 .3765 | 0 . 17592 | 0 .437646762 |
| ACADSB -0 .238363268 | 4 .856741492 | -1 .376 | 0 . 17607 | 0 .437922044 |
| FNDC3A -0 .29266872 | 7 .716266502 | -1 .3759 | 0 . 17612 | 0 .437952379 |
| SLA 0 .283900353 | 8 .856321066 | 1 .37559 | 0 . 1762 | 0 .438082653 |
| PSMA1 0 .210749109 | 1 1 . 10652561 | 1 .37513 | 0 . 17634 | 0 .438344485 |
| XAF1 0 .348652074 | 6 .695053022 | 1 .37451 | 0 . 17654 | 0 .43872892 |
| RNF126P1 0 .317050924 | 4 .936567176 | 1 .3744 | 0 . 17657 | 0 .43872892 |
| TFF3 -0 . 190107123 | 6 .439625923 | -1 .3742 | 0 . 17664 | 0 .438762119 |
| FBLN5 -0 .319193223 | 7 .634359126 | -1 .374 | 0 . 17669 | 0 .438762119 |
| KCNJ3 -0 .292566625 | 5 .652812835 | -1 .374 | 0 . 17669 | 0 .438762119 |
| ZSCAN26 -0 .31021884 | 5 .075422933 | -1 .3739 | 0 . 17674 | 0 .438762119 |
| MAP1LC3C -0 .361280852 | 5 .208662246 | -1 .3738 | 0 . 17676 | 0 .438762119 |
| GAL3ST4 -0 .299612292 | 8 .061410953 | -1 .3736 | 0 . 17682 | 0 .438831702 |
| BDKRB2 0 .27991538 | 6 .066173234 | 1 .37336 | 0 . 17689 | 0 .438914899 |
| DUOX2 -0 .325648517 | 3 .655017703 | -1 .3732 | 0 . 17693 | 0 .438936616 |
| RYR1 -0 .295041581 | 6 .751282327 | -1 .373 | 0 . 17701 | 0 .439045333 |
| RAP2C 0 . 143166392 | 7 .596233772 | 1 .37282 | 0 . 17706 | 0 .439070434 |
| LOC101927051 -0 .38089683 | 3 .541620993 | -1 .3725 | 0 . 17715 | 0 .439167514 |
| DNTTIP2 -0 . 183017323 | 8 .396577195 | -1 .3725 | 0 . 17717 | 0 .439167514 |
| SPTSSA -0 .221396925 | 8 .943446953 | -1 .3723 | 0 . 17721 | 0 .439167514 |
| SP3P 0 .38624689 | 5 . 146214156 | 1 .37224 | 0 . 17723 | 0 .439167514 |
| CNNM4 0 .200885116 | 7 .029891344 | 1 .37207 | 0 . 17729 | 0 .439202941 |
| POP1 0 . 196120178 | 6 .222689335 | 1 .37197 | 0 . 17732 | 0 .439202941 |
| ZNF225 -0 .354307157 | 3 .77433793 | -1 .3715 | 0 . 17747 | 0 .439478298 |
| DSTN -0 .203729346 | 1 1 .31014689 | -1 .3711 | 0 . 1776 | 0 .439653031 |
| DCC 0 .375218122 | 5 . 1 13821079 | 1 .37104 | 0 . 17761 | 0 .439653031 |
| FAM175B -0 . 192308878 | 8 .076650058 | -1 .3709 | 0 . 17766 | 0 .439680259 |
| CHST15 -0 .203494295 | 7 .248412919 | -1 .3708 | 0 . 17769 | 0 .439680259 |
| EPHA4 -0 .369478328 | 7 .294260091 | -1 .3706 | 0 . 17773 | 0 .439693714 |
| GTF2IRD1 -0 . 137653957 | 8 .227337251 | -1 .3704 | 0 . 17781 | 0 .439800894 |
| DNAJC16 -0 . 141217838 | 7 .051322453 | -1 .3699 | 0 . 17796 | 0 .440051925 |
| CHRM2 -0 .276245209 | 5 .987577784 | -1 .3697 | 0 . 17801 | 0 .440051925 |
| USP16 -0 .221328476 | 8 .007135849 | -1 .3697 | 0 . 17802 | 0 .440051925 |
| TTR 0 .200296305 | 6 .736162999 | 1 .36961 | 0 . 17805 | 0 .440051925 |
| GLS -0 . 175823486 | 9 .21838 | -1 .3693 | 0 . 17816 | 0 .440242293 |
| IRF2BP1 -0 . 177039445 | 7 .814091928 | -1 .3684 | 0 . 17843 | 0 .440818529 |
| IFIT3 0 .330052381 | 7 .664196323 | 1 .36789 | 0 . 17858 | 0 .4411 14304 |
| ZNF701 -0 .276511749 | 4 .656814359 | -1 .3675 | 0 . 17871 | 0 .441282878 |
| SLC1A1 0 . 191659418 | 6 .358501272 | 1 .36744 | 0 . 17872 | 0 .441282878 |
| RALGDS -0 . 187055451 | 9 .378350065 | -1 .3671 | 0 . 17882 | 0 .441441311 |
| SPINT2 -0 .51606244 | 8 .527825312 | -1 .3667 | 0 . 17897 | 0 .441711 188 |
| SLCO1C1 -0 .356902832 | 4 .276720542 | -1 .3661 | 0 . 17915 | 0 .442071759 |
| CNGB3 -0 .29623136 | 6 . 146133384 | -1 .3656 | 0 . 1793 | 0 .442292168 |
| LYZ 0 .756553143 | 9 .762035671 | 1 .36556 | 0 . 17931 | 0 .442292168 |
| UNKL -0 .204620202 | 5 .881384766 | -1 .3652 | 0 . 17943 | 0 .442517865 |
| EMX1 0 .612911441 | 3 .355625649 | 1 .36479 | 0 . 17955 | 0 .442713689 |
| KIAA0355 -0 . 152741093 | 7 .456188225 | -1 .3645 | 0 . 17965 | 0 .442871367 |
| TNK1 -0 . 185426002 | 9 . 132932887 | -1 .363 | 0 . 18012 | 0 .443892591 |
| TNFRSF10C -0 .340396967 | 5 . 158853397 | -1 .3629 | 0 . 18013 | 0 .443892591 |
| NOL8 -0 .313753746 | 6 .707689091 | -1 .3627 | 0 . 18019 | 0 .443959265 |

| KIR2DS3 | -0 .416692172 | 3 .730465797 | -1 .3622 | 0 . 18034 | 0 .444239316 |
| --- | --- | --- | --- | --- | --- |
| CLDN16 | -0 .40545948 | 3 .282559201 | -1 .3621 | 0 . 18039 | 0 .444274476 |
| LINC00939 | 0 .444001688 | 3 .215636953 | 1 .36154 | 0 . 18056 | 0 .444607205 |
| TSN | -0 .230473607 | 9 .349185753 | -1 .3614 | 0 . 18062 | 0 .444645312 |
| GOLGA7 | -0 .229648277 | 8 .975339901 | -1 .3612 | 0 . 18068 | 0 .444688003 |
| B3GNTL1 | -0 .345627763 | 6 .692164774 | -1 .3611 | 0 . 1807 | 0 .444688003 |
| MSH6 | -0 .200210933 | 7 .710497322 | -1 .3609 | 0 . 18075 | 0 .444723169 |
| NID2 | 0 .217240152 | 5 .996241151 | 1 .36065 | 0 . 18084 | 0 .444735377 |
| CYP26B1 | -0 .329629764 | 7 .812432395 | -1 .3606 | 0 . 18086 | 0 .444735377 |
| ANKFY1 | -0 . 185630003 | 6 .928434876 | -1 .3605 | 0 . 18089 | 0 .444735377 |
| NCOA2 | -0 . 138318799 | 8 .361135164 | -1 .3605 | 0 . 1809 | 0 .444735377 |
| RNF208 | -0 .356675075 | 6 .959702375 | -1 .3601 | 0 . 18102 | 0 .444942069 |
| N6AMT1 | -0 .563305068 | 3 .767445224 | -1 .3598 | 0 . 1811 | 0 .444957021 |
| TNFSF11 | 0 .218991403 | 5 .556713262 | 1 .35968 | 0 . 18115 | 0 .444957021 |
| F13B | -0 .368552524 | 3 .073904772 | -1 .3594 | 0 . 18123 | 0 .444957021 |
| MTMR1 | -0 . 173887067 | 9 .079899209 | -1 .3593 | 0 . 18126 | 0 .444957021 |
| SCP2 | -0 .261582245 | 10 .02680227 | -1 .3593 | 0 . 18128 | 0 .444957021 |
| NEK1 | -0 . 15655363 | 7 .917198751 | -1 .3592 | 0 . 18131 | 0 .444957021 |
| TTC1 | 0 .215118666 | 7 . 147780377 | 1 .35917 | 0 . 18131 | 0 .444957021 |
| FAM83E | 0 .250847264 | 3 .633182857 | 1 .35917 | 0 . 18131 | 0 .444957021 |
| WDR74 | -0 . 176046531 | 8 .666936216 | -1 .358 | 0 . 18166 | 0 .445682141 |
| CLCN5 | 0 .200936452 | 5 .852432959 | 1 .358 | 0 . 18168 | 0 .445682141 |
| PTPRG | 0 .342530609 | 5 .690373802 | 1 .35751 | 0 . 18183 | 0 .445919951 |
| PCCA | -0 .236290395 | 6 .091099429 | -1 .3575 | 0 . 18184 | 0 .445919951 |
| NDP | 0 .22477703 | 5 .526469339 | 1 .35718 | 0 . 18194 | 0 .445978223 |
| KRTAP1-3 | 0 .603204597 | 4 .001960122 | 1 .35717 | 0 . 18194 | 0 .445978223 |
| CLEC4M | 0 . 159788025 | 7 .307983136 | 1 .35694 | 0 . 18201 | 0 .446067772 |
| PPIA | 0 . 133735622 | 12 .26047447 | 1 .35678 | 0 . 18206 | 0 .446096253 |
| PSMD14 | 0 .267698523 | 8 .299944529 | 1 .3566 | 0 . 18212 | 0 .446096253 |
| RPP38 | 0 . 129161316 | 7 .756350901 | 1 .35656 | 0 . 18213 | 0 .446096253 |
| PRKAR1A | -0 .274851965 | 10 .83816218 | -1 .3562 | 0 . 18224 | 0 .446270348 |
| SLC15A2 | 0 .201589921 | 6 . 137067362 | 1 .356 | 0 . 18231 | 0 .446359865 |
| DSP | -0 .488870381 | 10 .9933389 | -1 .3556 | 0 . 18243 | 0 .446577223 |
| KPNA5 | -0 .282098759 | 4 . 105035457 | -1 .3551 | 0 . 18258 | 0 .446808311 |
| GAB1 | 0 .233221485 | 7 .242223021 | 1 .35508 | 0 . 1826 | 0 .446808311 |
| SEMA4D | 0 .380973598 | 7 .345850704 | 1 .35477 | 0 . 1827 | 0 .446867353 |
| LDHA | 0 .205132151 | 1 1 .53250716 | 1 .35469 | 0 . 18272 | 0 .446867353 |
| TBC1D1 | 0 . 165801782 | 7 .566143207 | 1 .35467 | 0 . 18273 | 0 .446867353 |
| ZNF276 | 0 .362477593 | 4 .964019052 | 1 .35445 | 0 . 1828 | 0 .446947875 |
| NCAPG2 | 0 . 175766484 | 7 .642323047 | 1 .3542 | 0 . 18287 | 0 .447050571 |
| BBS9 | 0 . 140829763 | 7 .86386781 | 1 .35376 | 0 . 18302 | 0 .447308557 |
| THEG | -0 .363487514 | 6 . 154071476 | -1 .3529 | 0 . 18329 | 0 .447903346 |
| ID1 | -0 .291478116 | 8 .359233568 | -1 .3526 | 0 . 18339 | 0 .448059583 |
[truncated: 577,135 more chars]
