# Supplementary material for: Identification of a Seven-Differentially Expressed Gene-Based Recurrence-Free Survival Model for Melanoma Patients
Source: Dis Markers. 2022 Jul 14;2022:3915112. doi: 10.1155/2022/3915112 (PMC9303152; doi:10.1155/2022/3915112)
Supplement: Supplementary Materials — Figure S1: functional enrichment result of overlapped DEGs between GSE98394 and GSE46517. Figure S2: the K-M curves show genes that significantly correlated with PFS. Grouping of samples is based on median gene expression. [file 3915112.f1.zip › Supplementary table 3 (1).docx]

**Supplementary** **table** **3.**Overlapped differentially expressed genes derived from GSE46517 and GSE98394 cohorts.

**Gene** **ID**

**_**

HLF

PHYHIP

RPS15A

TMEM47

FBXW7

ALDH3A2

RNASE4

LETMD1

RPL9

EXPH5

IL11RA

PFDN5

RPL27

FABP7

ADD3

CFH

CHP2

PDZD2

GPR37

GIPC2

PTN

FGFR3

CHL1

PPP1R3C

COBL

PAMR1

AHNAK2

FRZB

EPHX2

SORBS2

ABCA5

TFAP2B

SLC35A1

PDGFD

GATM

KLF5

KRT15

NAALADL1

GREM2

TRPM1

ITIH5

GRIA1

CADM1

BMP4

XYLT1

SYT17

ANKRD36

GATA3

RORA

ABCA8

HSPB2

PRAME

TUBB2A

CTSZ

S100A7

TNFRSF21

SPAG5

SPRR1A

SPRR1B

RGS1

FABP5

KRT6A

IVL

SERPINB3

AKR1B10

SERPINB4

S100A9

PMEL

DFNA5

UPP1

PI3

PHACTR1

TACC3

UBE2S

ZIC1

S100A8

WIPI1

C1QB

KPNA2

ASPM

THY1

MMP11

TIMP2

SPRR2B

KRT16

TNC

WARS

SLC7A5

MICB

MMP1

C2

CEP55

COTL1

MMP3

GDF15

GZMB

PHLDA2

ECM1

LGMN

SPP1

FKBP11

HJURP

S100A1

CHIT1

NEFH

HK2

TMEM158

CXCL2

MMP9

SLAMF7

CD14

ADAMDEC1

C1QA

KRT6B

DSC2

KIF23

TNFRSF1B

APOBEC3G

KIF20A

NDC80

TCN1

C5AR1

CYTL1

CITED1

CXCR4

GPR19

BST2

LCN2

CXCL9

MAGEA6
