# Supplementary figures and images for: Identification of a Seven-Differentially Expressed Gene-Based Recurrence-Free Survival Model for Melanoma Patients
Source: Dis Markers. 2022 Jul 14;2022:3915112. doi: 10.1155/2022/3915112 (PMC9303152; doi:10.1155/2022/3915112)

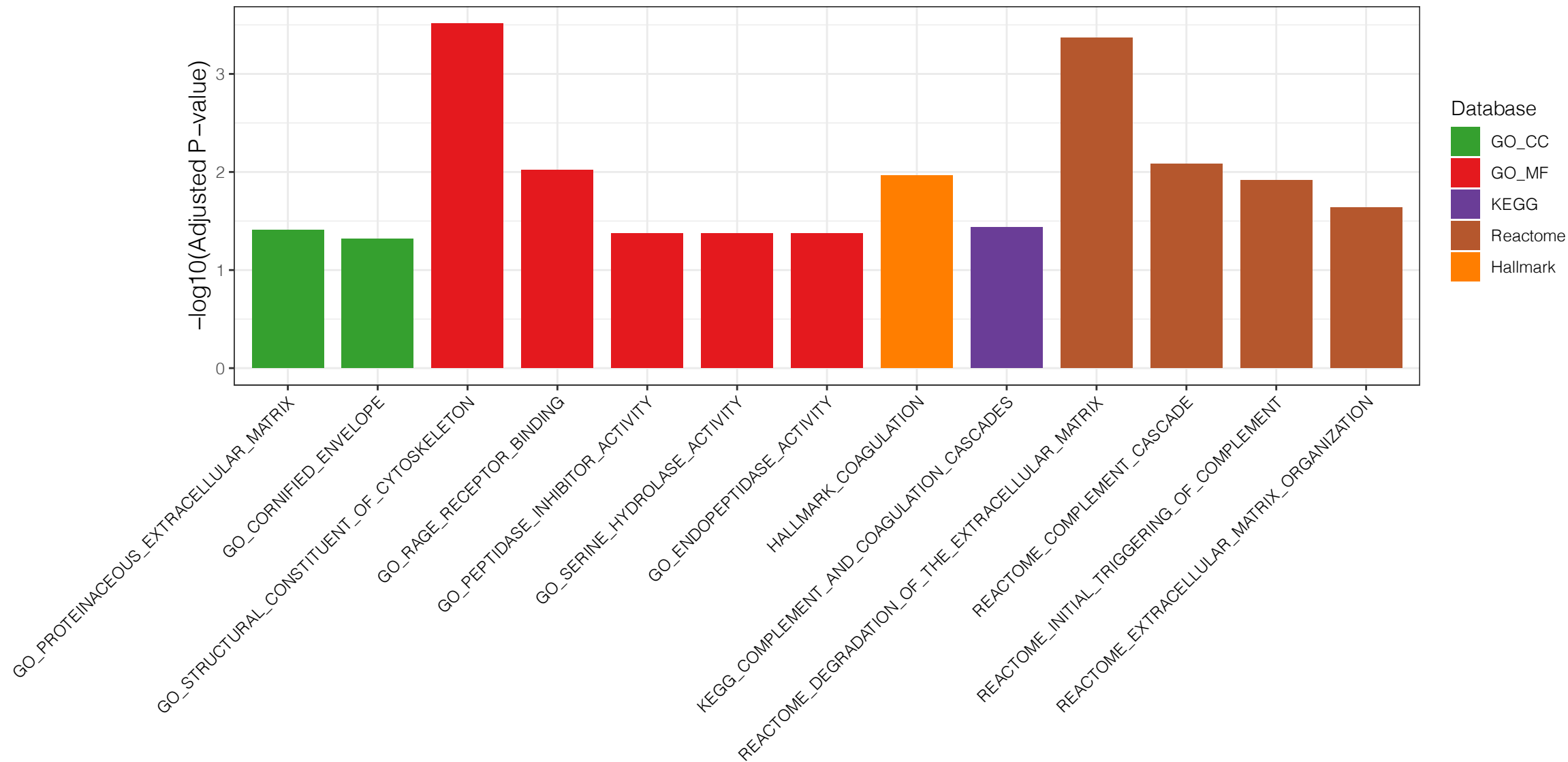

Supplement: Supplementary Materials — Figure S1: functional enrichment result of overlapped DEGs between GSE98394 and GSE46517. Figure S2: the K-M curves show genes that significantly correlated with PFS. Grouping of samples is based on median gene expression. [file 3915112.f1.zip › Figure S1 (1).pdf]

Strata — High expression — Low expression

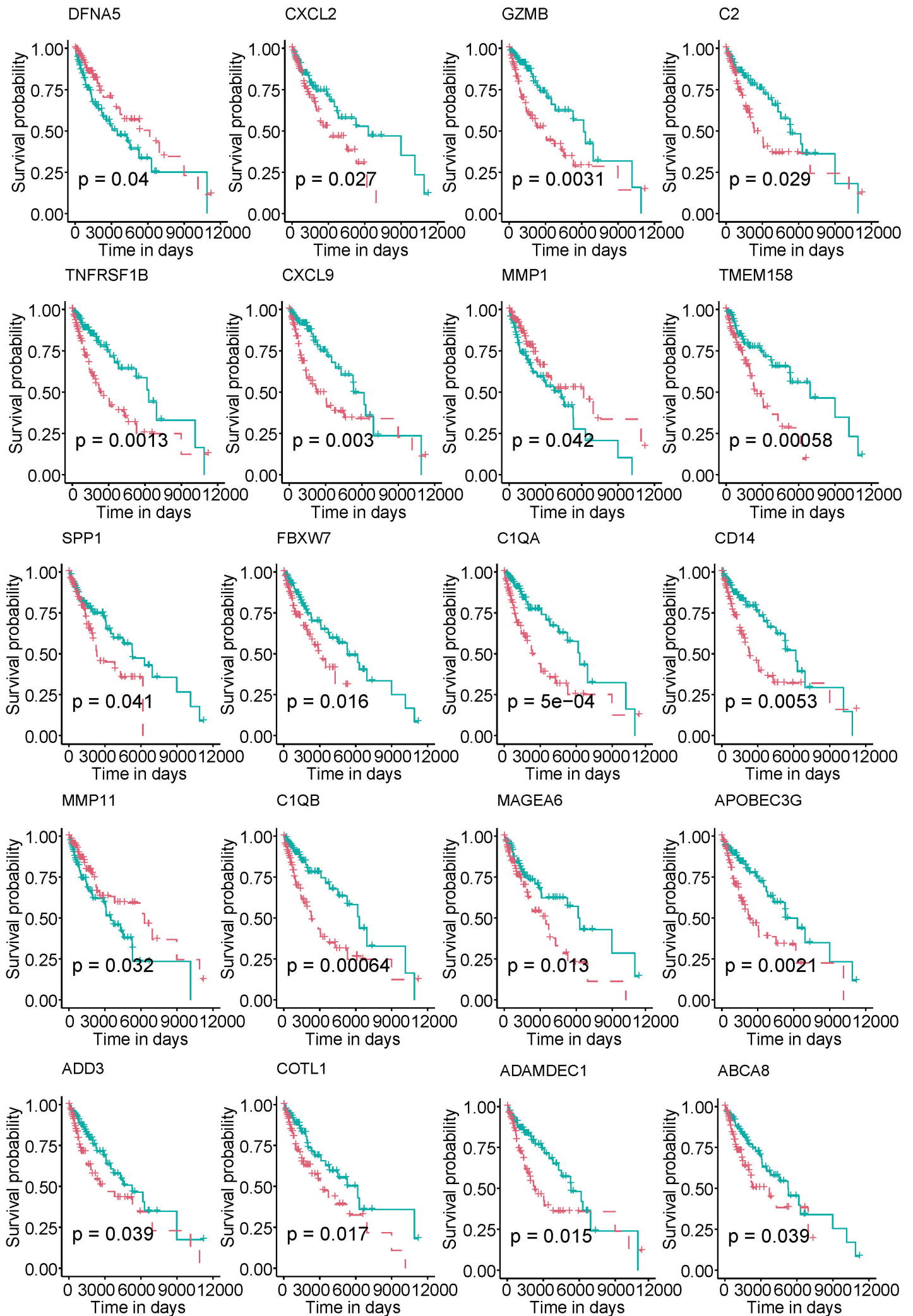

Supplement: Supplementary Materials — Figure S1: functional enrichment result of overlapped DEGs between GSE98394 and GSE46517. Figure S2: the K-M curves show genes that significantly correlated with PFS. Grouping of samples is based on median gene expression. [file 3915112.f1.zip › Figure S2 (1).pdf]
